# Site-switchable mono-*O*-allylation of polyols

**DOI:** 10.1038/s41467-020-19348-x

**Published:** 2020-11-10

**Authors:** Hua Tang, Yu-Biao Tian, Hongyan Cui, Ren-Zhe Li, Xia Zhang, Dawen Niu

**Affiliations:** 1 Department of Emergency, State Key Laboratory of Biotherapy, West China Hospital, and School of Chemical Engineering, Sichuan University, 610041 Chengdu, China; 2 State Key Laboratory of Natural Medicines, China Pharmaceutical University, 210009 Nanjing, China

**Keywords:** Homogeneous catalysis, Synthetic chemistry methodology, Carbohydrate chemistry

## Abstract

Site-selective modification of complex molecules allows for rapid accesses to their analogues and derivatives, and, therefore, offers highly valuable opportunities to probe their functions. However, to selectively manipulate one out of many repeatedly occurring functional groups within a substrate represents a grand challenge in chemistry. Yet more demanding is to develop methods in which alterations to the reaction conditions lead to switching of the specific site of reaction. We report herein the development of a Pd/Lewis acid co-catalytic system that achieves not only site-selective, but site-switchable mono-*O*-allylation of polyols with readily available reagents and catalysts. Through exchanging the Lewis acid additives that recognize specific hydroxyls in a polyol substrate, our system managed to install a versatile allyl group to the target in a site-switchable manner. Our design demonstrates remarkable scope, and is amenable to the direct derivatization of various complex, bioactive natural products.

## Abstract (web summary)

Selective manipulation of one functional group, out of many repeatedly occurring in a substrate, represents a grand challenge in chemistry. Here, the authors report a Pd/Lewis acid cocatalytic system that achieves not only site-selective, but also site-switchable mono-*O*-allylation of polyols.

## Introduction

**Fig. 1:**
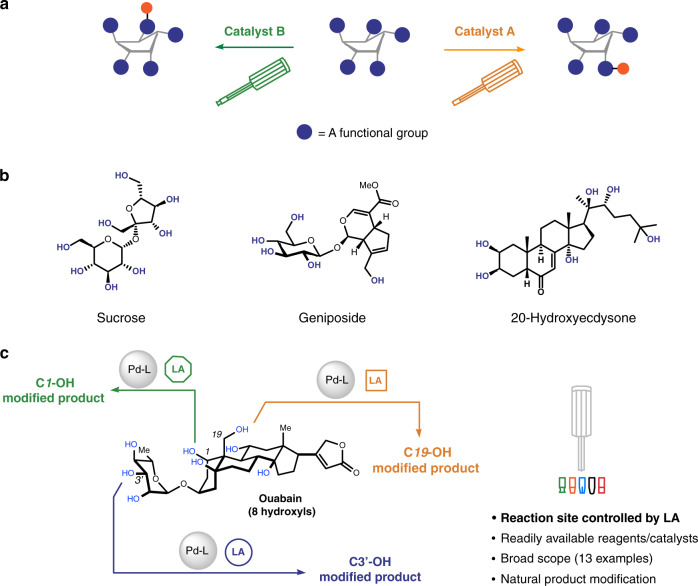
Site-switchable modification of molecules containing multiple identical functional groups. **a** Reagent/catalyst-controlled, site-selective modification of complex molecules: a challenge in synthetic chemistry. **b** Some representative, naturally occurring polyols. **c** Pd/Lewis acid co-catalyzed, site-switchable modification of polyols (this work).

Molecules containing multiple copies of the same functional group are ubiquitous in Nature and in drug candidates. Site-selective transformations—reactions that can manipulate one of these repeating functional groups while keeping others unaffected—provide efficient access to analogues and derivatives of these compounds, thereby facilitating the interrogation and exploitation of their properties^1,2^. The same kind of functional groups tend to undergo similar transformations, however, and differentiation typically only occurs due to subtle steric and electronic environments. Accordingly, to achieve high site-selectivity following a generalizable strategy remains a significant task in chemistry^3–8^. Even more demanding is the development of methods in which alterations to the reaction conditions result in switching of the specific site of reaction (Fig. 1a), since it necessitates the selective modification at positions that are inherently less reactive^9–16^.

Polyhydroxylated natural products, carbohydrates in particular, play critical roles in virtually all biological processes and they are essential components of many pharmaceuticals (Fig. 1b). Rapid access to the derivatives of these compounds holds tremendous opportunities to understand and modulate key biological processes^17–19^. Polyols contain numerous hydroxyl groups that offer excellent opportunities for derivatization. Manipulation of only one or a certain few hydroxyls in the presence of many others in these substrates, however, used to rely heavily on protection/deprotection sequences^20^. Regardless, recent studies have shown great promise and potential for the direct, site-selective modification of polyols. In this realm, various methods^21,22^ have been established to selectively modify the intrinsically most reactive hydroxyl groups within a substrate (substrate control). Recently, systems that can override the intrinsic reactivity preferences and accomplish catalyst-controlled, site-switchable modification of complex polyols have emerged^23–35^. As eminent examples, in a series of landmark studies, the Miller group^23–26^, has identified several oligopeptide-based catalysts that enabled site-switchable modification of complex antibiotics, such as vancomycin, teicoplanin, and erythromycin. In these studies, the oligopeptide catalysts were designed as mimetics of the catalytic domains of enzymes. Kawabata et al.^29–31^ devised chiral pyridine derivatives that were used in the site-selective modification of C4-OH of glucopyranosides as well as natural products such as lanatoside C and avermectin B_2a_. The Tan group^32^ invented a pair of pseudoenantiomeric imidazole-based catalysts that allow site-divergent modification of polyols containing *cis*-1,2-diol moieties, including anticancer agent digitoxin. Nargony and coworkers^33^ employed chiral phosphoric acid catalysts to accomplish site-switchable glycosylation of 6-deoxy erythronolide. Our group^34^ reported the site-divergent *O*-propargylation of various monosaccharides as well as digitoxin employing a pair of chiral Cu-catalysts. These achievements notwithstanding, to develop general systems that can achieve catalyst-controlled, switchable site-selectivity still represents a challenge. In particular, methods capable of introducing a metabolically stable ether bond remain rather limited.

Here we report a strategy that enables site-switchable mono-*O*-allylation of polyols by Pd/Lewis acid co-catalysis^36^. As a distinct feature of our system, the task of activating electrophiles and that of controlling site-selectivity were allocated, respectively, to the Pd-catalyst and the Lewis acid additive. Resembling the role of a gRNA in the Cas/gRNA^37^ system, the Lewis acid additive serves as a guide in our system, and determines the site of reaction in the modification of various polyols (Fig. 1c). Interestingly, such a strategy is akin to the switchable screwdrivers we use in our daily lives. The potential of this principle, however, has not been systematically explored and exploited in chemistry to develop site-selective methodologies. The derivatization of cardiac glycoside ouabain provides an excellent example to illustrate our strategy. As will be discussed in more detail below, using the same Pd-catalyst to activate the electrophile but changing the identity of the specific Lewis acid additive, we accomplish highly selective allylation of the C1-OH, C3’-OH, or C19-OH of ouabain (Fig. 1c).

## Results

### Reaction design

**Fig. 2:**
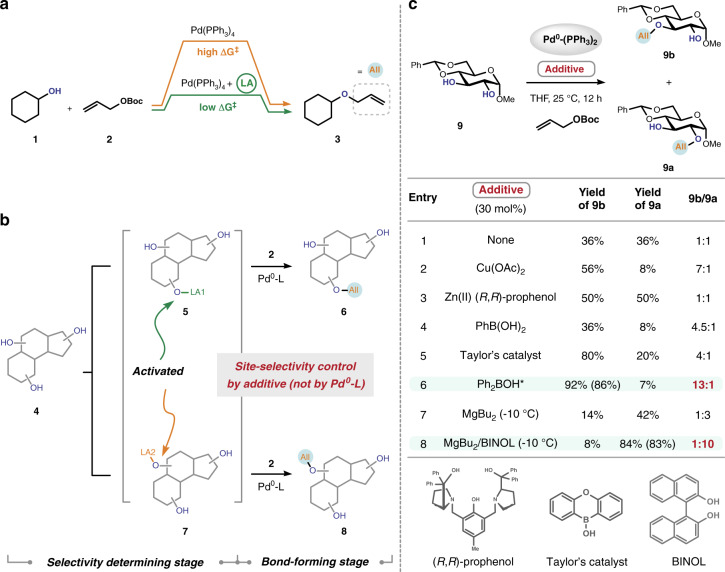
Mechanistic underpinning and reaction design. **a** The Pd-catalyzed *O*-allylation of aliphatic alcohols is accelerated by addition of Lewis acids. **b** Our reaction design: the use of different Lewis acids (LA) to control site-selectivity. **c** Initial realization of site-switchable modification of polyols by exchanging additives. ^‡^Reactions were performed on a 0.2 mmol scale. Product ratios were determined by ^1^H-nuclear magnetic resonance (NMR) analysis of crude reaction mixtures. Yields in parenthesis are isolated yields. Pd^0^-(PPh_3_)_2_ used in this study was generated in situ from Pd_2_dba_3_•CHCl_3_ and PPh_3_. ^*^Generated in situ from the commercial Ph_2_BOCH_2_CH_2_NH_2_. Ac acetyl, Ph phenyl, Boc *t*-butoxycarbonyl, Bu butyl, THF tetrahydrofuran, BINOL 1,1’-bi-2-naphthol, dba dibenzylideneacetone.

We were interested in the selective *O*-allylation of polyols because the allyl group is among the smallest, yet most versatile, units in chemistry^38^. Among all of the allylic alkylation methods established to date, a well-studied and often utilized one is the Pd-catalyzed Tsuji–Trost reaction^39^. Intriguingly, the allylation of aliphatic alcohols by the Tsuji–Trost reaction is often a slow process (**1** + **2** to **3**, Fig. 2a, orange arrow), but it can be accelerated by using Lewis acid additives (Fig. 2a, green arrow, and Supplementary Fig. 2 in Supplementary Information). The Lewis acid additives presumably function through complexing with hydroxyls, which would enhance their acidity and facilitate their deprotonation, thereby increasing their nucleophilicity^39^. Our initial supposition was that the unique reactivity of aliphatic alcohols in the Tsuji–Trost reaction could be leveraged to achieve reagent-controlled site-selectivity during the modification of polyols. Specifically, we reasoned that if a Lewis acid additive (e.g., LA1 or LA2 in Fig. 2b) could be identified to selectively complex with a certain hydroxyl and enhance its reactivity toward the π-allylpalladium intermediates, site-selective functionalization of polyols would result since hydroxyls not interacting with this additive remain largely inert under the conditions (**4** to **6** via **5**, or, **4** to **8** via **7**). More importantly, in this regime, switch of the reaction site could in principle be realized simply by tuning the properties (bulkiness, number of available binding sites, Brønsted basicity, etc.) of the Lewis acid additives. Compared with approaches to achieve selectivity switch through modifying the whole catalytic complex (e.g., Pd-ligand complex here) for each substrate, the advantage of our design lies in the greater ease and extent of changing the characters of Lewis acid additives.

The validity of our design was first established in the site-divergent mono-*O*-allylation of glucopyranoside **9** (Fig. 2c). Compound **9** contains two vicinal, equatorial hydroxyl groups that are similarly reactive toward an electrophilic π-allylpalladium intermediate. Under the standard Pd-catalyzed *O*-allylation conditions and in the absence of any additives, the C2-OH modified product **9a** and the C3-OH modified **9b** were formed in almost equal amounts (entry 1, Fig. 2c). We then attempted adding each of the Lewis acid complexes listed in Fig. 2c individually to the reaction mixture so as to explore their effects on the site-selectivity profile of this transformation (entry 2–7). We found that the use of Ph_2_BOH as an additive resulted in the predominant formation of **9b** in an efficient fashion (entry 6). Notably, the O’Doherty group^40,41^ reported the use of Ph_2_BOH to achieve Pd-catalyzed site-selective glycosylation. More intriguingly, the use of MgBu_2_ as an additive led to the preferential formation of the other isomer **9a**, albeit with moderate selectivity (entry 7). Further optimization of reaction conditions revealed that simultaneous addition of MgBu_2_ with BINOL afforded **9a** in dramatically improved site-selectivity and efficiency (entry 8). Thus, we identified two sets of conditions that install an allyl group onto the two different hydroxyl groups in **9** (see below for a mechanistic rationalization). These conditions employ an identical transition metal complex [i.e., Pd^0^-(PPh_3_)_2_] and differ only in the oxophilic additives utilized [Mg(II) vs. B(III)]. Collectively, these results validated the proof of principle of a strategically distinct approach to achieve reagent-controlled, site-switchable modification of polyols.

### Reaction scope

**Fig. 3:**
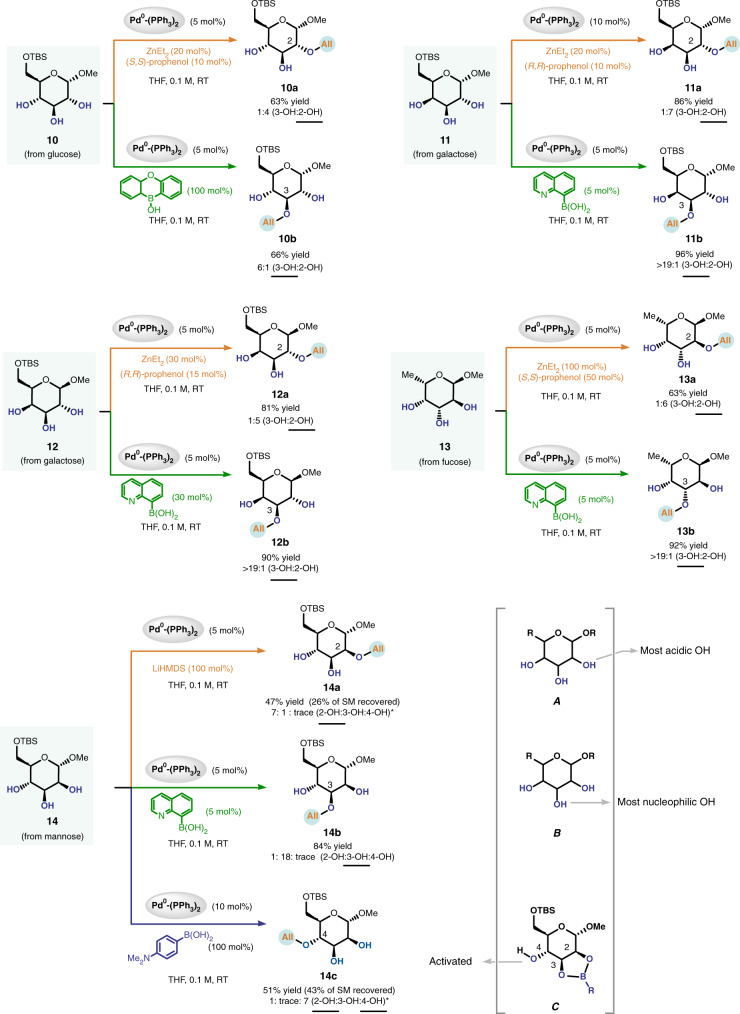
Site-switchable mono-*O*-allylation of monosaccharides. Product ratios were determined by ^1^H NMR analysis of crude reaction mixtures. Reported yields are isolated yields of products that are underlined. * A small amount of diallylated product was formed. See Supplementary Information for experimental details. L ligand.

Following the above protocol, we proceeded to explore the generality of our strategy. We first examined this method in the site-selective modification of various monosaccharides (Fig. 3), including those derived from glucose (**10**), galactose (**11–12**), and fucose (**13**). All of these monosaccharides contain three contiguous secondary hydroxyls. In these cases, the allylation occurs at the C3-OH of the pyranose ring if an organoboron compound^42^ was used as additive (green arrows), but at the C2-OH when ZnEt_2_-prophenol complex^43^ was employed (orange arrows). To rationalize the switchable site-selectivity observed in these cases (**10–13**, Fig. 3) and in the case of **9** (Fig. 2c), we propose that the C3-OH is the most nucleophilic^44^ hydroxyl in each of these substrates, and it therefore most readily complexes with the organoboron compound (cf. structure **A**). The C2-OH, on the other hand, is most readily deprotonated by a base since it is closest to the electron-withdrawing anomeric center (cf. structure **B**).

Each of the three hydroxyls in mannopyranoside **14** could be selectively alkylated in a controllable manner. As can be explained by the structures **A** and **B** shown above, the use of LiHMDS (orange arrow) and quinolin-8-ylboronic acid (green arrow) furnished the C2-OH allylated product **14a** and the C3-OH allylated product **14b**, respectively. The C4-OH allylated product **14c** could be obtained by using 4-dimethylaminophenylboronic acid, which, through formation of a stable boronic ester (cf. structure **C**), deactivates the C2-OH and C3-OH^45^ and simultaneously enhances the reactivity of the C4-OH position.

**Fig. 4:**
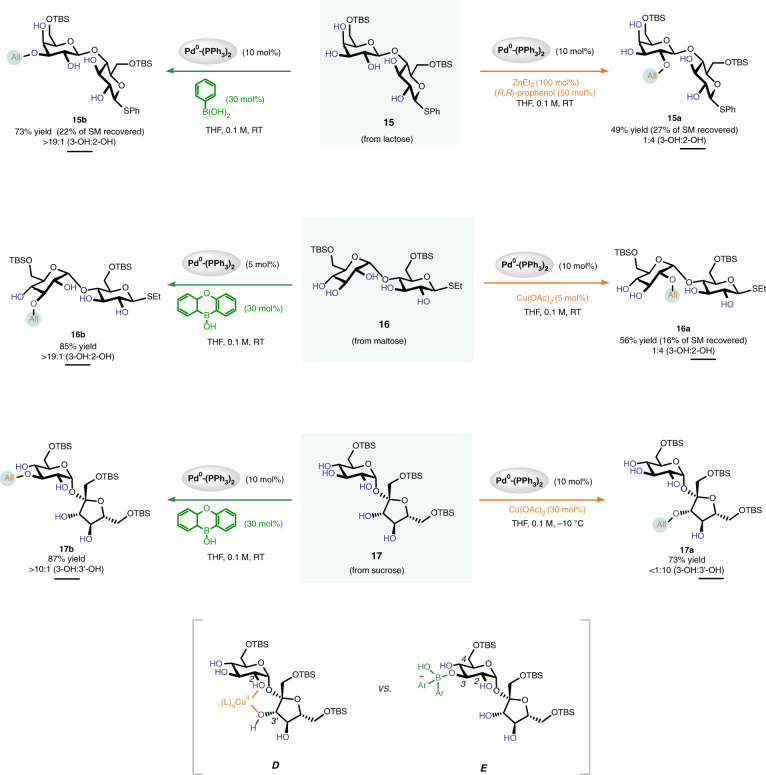
Site-switchable mono-*O*-allylation of disaccharides. Product ratios were determined by ^1^H NMR analysis of reaction mixtures. Reported yields are isolated yields of products that are underlined. See Supplementary Information for experimental details.

We then showed this strategy could be adopted toward the site-switchable allylation of structurally more complex disaccharides (Fig. 4). For example, the lactose derivative **15** contains five free hydroxyl groups. The use of ZnEt_2_-prophenol complex directed the allyl group to the C2-OH of **15** with high selectivity (orange arrow). Switching the additive to phenylboronic acid altered the preferential reaction site to the C3-OH (green arrow). Moving to maltoside **16**, we found the use of Taylor’s catalyst^46^ (10*H*-dibenzo[*b*,*e*][1,4]oxaborinin-10-ol) guided the installation of the allyl group to the C3-OH, while employment of Cu(OAc)_2_ steered the allyl group to the C2-OH instead. It is worth noting that synthetically versatile but chelating thioether groups present in **15** and **16** were well tolerated by our conditions. We further applied this method to the site-divergent modification of fructoside **17**, which contains a pyranoside ring linked to a furanoside ring. We precisely delivered the allyl group to the C3-OH of the pyranoside ring using Taylor’s catalyst as an additive, or to the C3’-OH of the furanoside ring if applying Cu(OAc)_2_, instead. Here, we rationalize that Taylor’s catalyst complexes with the C3-OH of the pyranose ring (see structure **E**), but Cu(OAc)_2_ chelates^47^ with the C2-OH and the C3’-OH of **17** and activates the C3’-OH (cf. structure **D**). It is worth highlighting that, in this case, the selectivity switches between two hydroxyls that are six atoms apart. Therefore, it would be challenging to achieve a selectivity switch between these two hydroxyls through designing Pd-ligand complexes.

### Modification of natural products

**Fig. 5:**
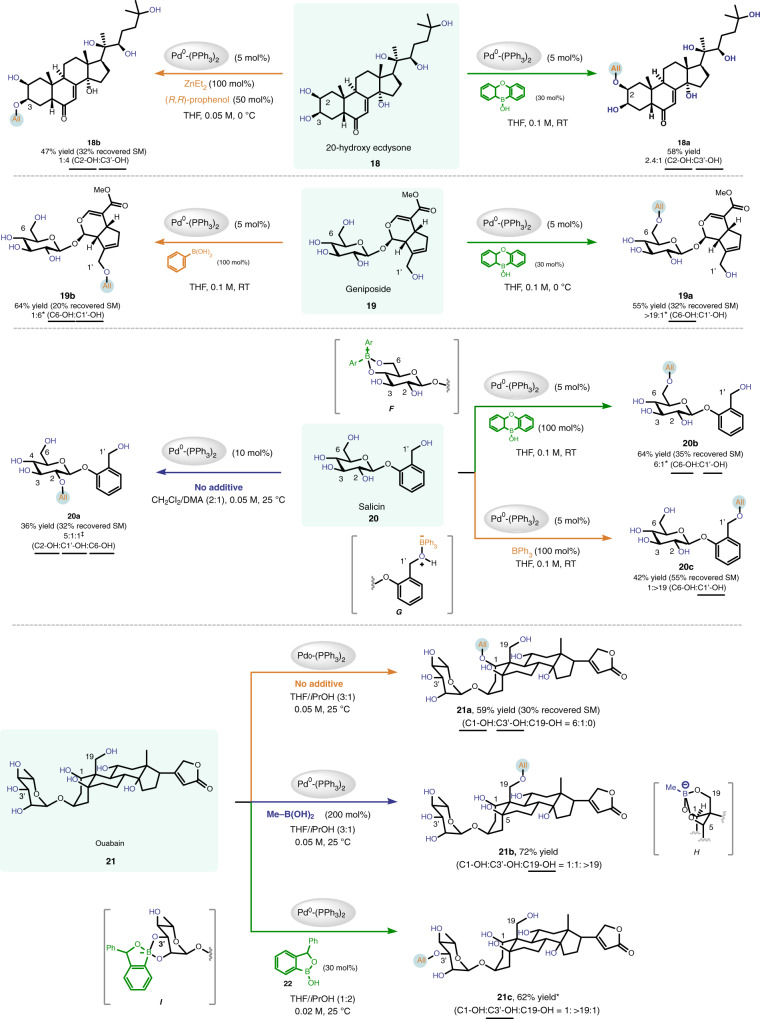
Site-switchable mono-*O*-allylation of polyhydroxylated natural products. Product ratios were determined by ^1^H NMR analysis of crude reaction mixtures. Reported yields are isolated yields of products that are underlined. ^*^A small amount of diallylated product was also formed. ^‡^Small amounts of C3-OH and C4-OH modified products were also formed. DMA, *N,N*-dimethyl acetamide. See Supplementary Information for experimental details.

We continued to apply our strategy to the site-switchable modification of polyhydroxylated natural products (Fig. 5). 20-Hydroxyecdysone (**18**) is an ecdysteroid hormone that controls the metamorphosis and ecdysis of arthropods. This compound contains six free hydroxyls, three of which are secondary. Site-divergent modification of **18** could be achieved. If Taylor’s catalyst was employed as additive, the allylation occurred preferentially at the C2-OH (green arrow), whereas if Zn(II)-Prophenol complex was used, the allylation occurred at the C3-OH (orange arrow). Geniposide (**19**) is an iridoid glycoside that exhibits diverse biological activities. In addition to five free hydroxyls, this compound also contains a sensitive enol ether moiety and an enoate ester group. Employing our strategy, we could selectively install an allyl group to the C6-OH of the glucose ring employing Taylor’s catalyst, or to the C1’-OH with phenylboronic acid as an additive. Here, the site of reaction switches cleanly between two primary hydroxyls.

Following the above principle, we were able to selectively modify up to three different hydroxyl groups within some complex polyols. For example, salicin (**19**) is a natural product that acts as an anti-inflammatory agent in the human body. The C2-OH of the glucopyranoside ring in **19** is intrinsically the most reactive hydroxyl and gets allylated preferentially in the absence of any additives (blue arrow). Intriguingly, applying Taylor’s catalyst as an additive in the standard reaction conditions delivered the allyl group to the C6-OH of **19** (green arrow), presumably via the intermediacy of the ate complex **F**. If the bulky, monovalent BPh_3_ was employed instead (orange arrow), the allylation occurs at the benzylic hydroxyl (C1’-OH) with high site-selectivity (cf. **G**). Thus, three individual hydroxyls within salicin could be selectively modified in a controllable fashion.

Ouabain (**21**) exhibits potent antiproliferative activities against several cancer cell lines at nanomolar concentrations^48,49^. This molecule contains eight free hydroxyls, including one primary, five secondary, and two tertiary ones. When no Lewis acid additive is used, the Pd-catalyzed allylation occurs at the secondary C1-OH to afford **21a** with decent selectivity and efficiency (orange arrow). When we used methylboronic acid as an additive, the primary C19-OH was selectively modified to give product **21b** in high yield (blue arrow). We hypothesize that methylboronic acid simultaneously complexed with the C1-OH, C5-OH and C19-OH of **21** (see structure **H**), and activated the least hindered primary hydroxyl in the Pd-catalyzed allylation reactions. If the oxaborole derivative **22** was employed instead, the site-selectivity changed again. This time the C3’-OH of the sugar ring was modified with high efficiency (green arrow). We assume that the bulky oxaborole **22** will preferentially form an ate complex with the vicinal diol moiety in the sugar ring (see structure **I**), which renders the equatorial C3’-OH most reactive. Lastly, the group that can be transferred by our method is not restricted to the parent allyl unit. For instance, cinnamyl groups bearing various substituents could be installed onto ouabain with good site-selectivities as well, if the corresponding electrophiles are employed (see Supplementary Fig. 3).

### Product derivatization

**Fig. 6:**
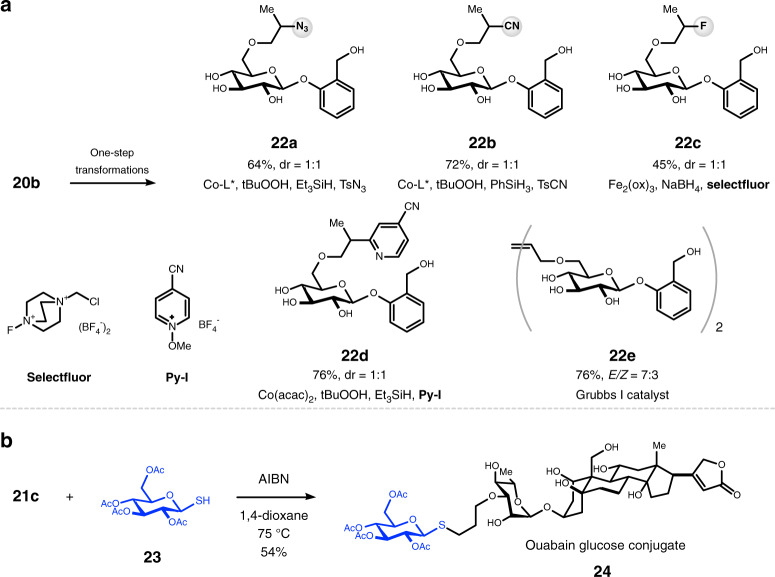
Product derivatization. **a** Derivatization of **20b**. **b** Derivatization of **21c**. Product ratios were determined by ^1^H NMR analysis of crude reaction mixtures. See Supplementary Information for experimental details. L* = (*R,R*)-( − )-*N*,*N*’-Bis(3,5-di-*tert*-butylsalicylidene)-1,2-cyclohexanediamine. ox oxalate.

To further demonstrate the utility of this method as a tool for the late stage functionalization of naturally occurring polyols, we have performed the reactions shown in Fig. 6. We show that the alkene group installed on salicin could be conveniently converted to a variety of other functional groups in one step (Fig. 6a). For instance, we showed that a cobalt-catalyzed hydroazidation reaction^50^ smoothly incorporated an azide group onto **20b**. The resulting product **22a** is thus primed for the venerable azide alkyne coupling reactions^51^ that are extensively utilized in the fields of chemical biology and material science. By using similar hydrofunctionalization reactions of alkenes, we also successfully attached a nitrile^52^ group (**22b**), a fluorine^53^ atom (**22c**), or a pyridine^54^ ring (**22d**) onto the target molecule. Furthermore, a cross-metathesis reaction^55^ readily converted **20b** to a dimeric structure **22e**. Lastly, we prepared a conjugate of ouabain and glucose (**24**) from **21c** and **23**, taking advantage of the potent thiol-ene click reaction^56^. Therefore, when coupled with the synthetic versatility of alkenes, our site-switchable *O*-allylation reaction provides rapid and efficient accesses to derivatives and analogues of complex polyols.

## Discussion

This study showcased the power of a dual catalytic strategy for site-switchable modification of target molecules, a key challenge in chemistry. This design allocates the task of activating electrophiles and that of controlling site-selectivity to, respectively, the Pd-catalyst and the Lewis acid additive. Thus, switch of reaction site was achieved simply through changing the identity of Lewis acid additives, a tactic that has not been systematically explored before. The allyl unit installed by this method could serve as an entry point to various other functional groups. We anticipate that the generality, immediate utility, and the operational ease of this method will inspire further exploration that ultimately leads to precise control of site-selectivity during modification of complex compounds.

## Methods

### Synthesis of 9a

In a N_2_-filled glovebox, *n*Bu_2_Mg (1 M in heptane, Energy Chemicals, Lot DL140184, 45 μL, 0.045 mmol, 0.3 equiv), (±)-BINOL (8.6 mg, Energy Chemicals Lot DI240132, 0.03 mmol, 0.2 equiv) and THF (200 μL) were weighed into a screw capped vial (labeled as **Vial A**) containing a stir bar. The mixture was stirred for 5 min, at which time compound **9** (42.6 mg, 0.15 mmol, 1.0 equiv) was added. The resulting solution was stirred for an additional 30 min.

To another vial, compound **2** (36.4 mg, 0.23 mmol, 1.5 equiv), Pd_2_(dba)_3_•CHCl_3_ (7.8 mg, 7.5 μmol, 0.05 equiv), PPh_3_ (7.8 mg, 0.03 mmol, 0.2 equiv), and THF (200 μL) were added in sequence. The mixture was stirred for 10 min, and then transferred to **Vial A**. An additional portion of THF (2.6 mL) was added to Vial A so that [**9**] was adjusted to 0.05 M. **Vial A** was tightly capped, taken out of the glovebox, and stirred at −10 °C for 12 h (400 rpm). The reaction mixture was then concentrated in vacuo. An aliquot of the residue was taken for ^1^H NMR analysis, which indicated that products **9a** and **9b** were formed in a ratio of ca. 10:1. Flash chromatography (SiO_2_) using petroleum ether/EtOAc (6:1–3:1) as eluent afforded **9a** as a yellow solid (41 mg, 0.127 mmol, 83%).

### Synthesis of 9b

In a N_2_-filled glovebox, compound **9** (56.5 mg, 0.20 mmol, 1.0 equiv) and 2-(diphenylboryloxy)-ethanamine (2.4 mg, Aladdin Lot J1824016, 0.01 mmol, 5 mol%), and THF (500 μL) were weighed into a screw capped vial (labeled as **Vial A**) containing a stir bar. The resulting solution was stirred for an additional 30 min.

To another vial, compound **2** (38.0 mg, 0.24 mmol, 1.2 equiv), Pd_2_(dba)_3_•CHCl_3_ (5.2 mg, 5 μmol, 0.025 equiv), PPh_3_ (5.2 mg, 0.02 mmol, 0.1 equiv), and THF (200 μL) were added in sequence. The mixture was stirred for 10 min, and then transferred to **Vial A**. An additional portion of THF (0.3 mL) was added to Vial A so that [**9**] was adjusted to 0.2 M. **Vial A** was tightly capped, taken out of the glovebox, and stirred at 25 °C (outside temperature) for 12 h (400 rpm). The reaction mixture was then concentrated in vacuo. An aliquot of the residue was taken for ^1^H NMR analysis, which indicated that products **9a** and **9b** were formed in a ratio of 1:13. Flash chromatography (SiO_2_) using petroleum ether/EtOAc (3:1–1:1) as eluent afforded **9b** as a pale yellow oil (55.4 mg, 0.172 mmol, 86%).

## Supplementary information

Supplementary Information

## Data Availability

Additional data supporting the findings described in this paper are available in the Supplementary Information, and also are available from the corresponding author upon reasonable request.
