# Supplementary material for: Site-switchable mono-O-allylation of polyols
Source: Nat Commun. 2020 Nov 10;11:5681. doi: 10.1038/s41467-020-19348-x (PMC7655818; doi:10.1038/s41467-020-19348-x)
Supplement: Supplementary file 1 — Supplementary Information [file 41467_2020_19348_MOESM1_ESM.pdf]

Supplementary Information

Site-switchable mono-*O*-allylation of polyols

Hua Tang[1], Yu-Biao Tian[1], Hongyan Cui[1], Ren-Zhe Li[1], Xia Zhang[1], Dawen Niu*[1,2]

[1]Department of Emergency, State Key Laboratory of Biotherapy, West China Hospital, and School of Chemical Engineering, Sichuan University, Chengdu 610041, China.
[2]State Key Laboratory of Natural Medicines, China Pharmaceutical University, Nanjing 210009, China.

*Correspondence to: niudawen@scu.edu.cn.

Contents of Supplementary Information

1. General Information and Supplementary Figure 1 2

2. Supplementary Figure 2 3

3. Supplementary Figure 3 4

4. Supplementary Methods 5

4.1 Procedures and Characterization Data for Compounds in Figure 2c 5

4.2 Procedures and Characterization Data for Compounds in Figure 3 8

4.3 Procedures and Characterization Data for Compounds in Figure 4 25

4.4 Procedures and Characterization Data for Compounds in Figure 5 34

4.5 Procedures and Characterization Data for Compounds in Figure 6 53

5. NMR spectra 59

6. Supplementary References 250

General Information

Flash chromatography was performed manually, using silica gel purchased from Qindao Haiyang, and mixtures of petroleum ether/ethyl acetate or dichloromethane/methanol as eluting solvents. Anhydrous tetrahydrofuran was freshly distilled from sodium/benzophenone under N_2 or purchased from Energy Chemicals or Adamas and used as received. *i*PrOH was purchased from Energy Chemicals or Adamas and used as received. $Pd(dba)_2·CHCl_3$ was purchased from Adamas (Lot# P1336374) and used as received. PPh_3 was purchased from Adamas (Lot# P1543897) and used as received. Allylic carbonate was prepared according to reported literature procedures.[1] Monosaccharides and disaccharides used in this study, if not commercial, were prepared following reported literature procedures.[2,3] Commercially available Lewis acids and additives were purchased and used as received, with their sources and Lot Nos. specified in the reaction procedures when mentioned. Reaction outcomes of the control experiments where no additive is used were mentioned at the end of the experimental details for each substrate.

All new compounds were characterized by NMR spectroscopy, IR spectroscopy, high-resolution mass spectroscopy (HRMS), and melting point (if solids). NMR spectra were recorded on a Bruker AMX 400 spectrometer and were calibrated using TMS or residual deuterated solvent as an internal reference [$CDCl_3$: 7.26 ppm or 0.00 ppm (TMS) for ^1H NMR and 77.16 ppm for ^{13}C NMR; CD_3OD: 3.31 ppm for ^1H NMR and 49.00 ppm for ^{13}C NMR], and the tabulated data were reported in ppm. All IR spectra were taken on a Thermo Scientific Nicolet iS5 spectrometer (iD5 ATR, diamond). HRMS spectra were recorded on a Waters Q-TOF Premier. Melting points (m.p.) were recorded on an INESA SGW X-4 melting point apparatus. Optical rotations were measured on a Rudolph Research Analytical Autopol VI polarimeter with $[\alpha]_D$ values reported in degrees; concentration (c) is in g/100 mL.

Supplementary Figure 1. Vials used in this study: (A) a 5 mL screw-capped vial; (B) a 10 mL Schlenk tube.

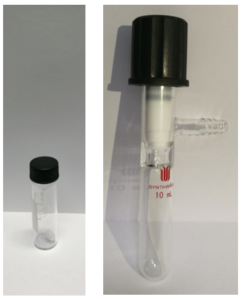

(A) (B)

Supplementary Figure 2: The Pd-catalyzed *O*-allylation of cyclohexanol is accelerated by the use of various Lewis acids.[a]

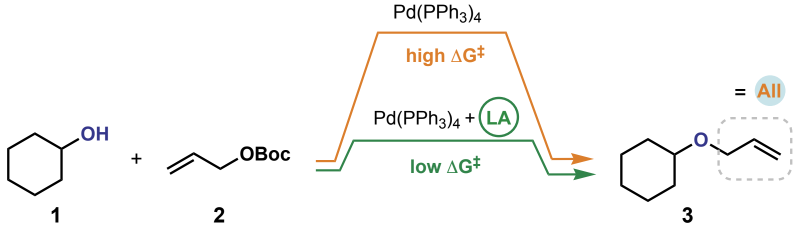

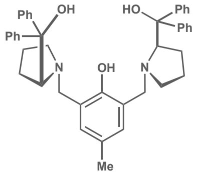

(*R*,*R*)-Prophenol

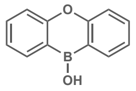

Taylor's catalyst

LA used (30 mol%)	Yield of 3 @ 30 min, RT
none	<5%
Cu(II) bis(2-ethylhexanoate)	71%
Zn(II) (*R*,*R*)-Prophenol complex	78%
$PhB(OH)_2$	52%
Taylor's catalyst	40%
$MgBu_2$	57%

[a] Yields were determined by gas chromatography (GC) analysis of crude reaction mixtures relative to an internal standard (dodecane).

Reaction Procedure:

In a N_2-filled glovebox, a stock solution of cyclohexanol (50 mg, 0.5 mmol) and dodecane (29 mg, 0.17 mmol, internal standard) in anhydrous THF (1 mL) was prepared in a vial labeled as **Vial A**. Another stock solution of allyl carbonate (118 mg, 0.75 mmol, 1.5 equiv), $Pd_2(dba)_3$•$CHCl_3$ (13 mg, 12.5 μmol, 2.5 mol%) and PPh_3 (13 mg, 0.05 mmol, 10 mol% equiv) in anhydrous THF (1 mL) was prepared in a vial labeled as **Vial B**.

A Lewis acid (0.3 equiv) was weighed into a screw capped vial containing a stir bar. Anhydrous THF (200 μL) and 200 μL of the stock solution in **Vial A** were added to this vial, and the resulting mixture was stirred at 25 °C for 30 min. Then, 200 μL of the stock solution in **Vial B** was added. The vial was tightly capped, taken out of the glove box, stirred at 25 ˚C for 30 min (400 rpm stirring). The reaction mixture was then filtered through a column of cotton, diluted with ethyl acetate, and subjected to GC analysis. The yields listed in Supplementary Figure 2 are average of at least two runs.

Supplementary Figure 3: Site-selective installation of cinnamyl groups onto ouabain.[a]

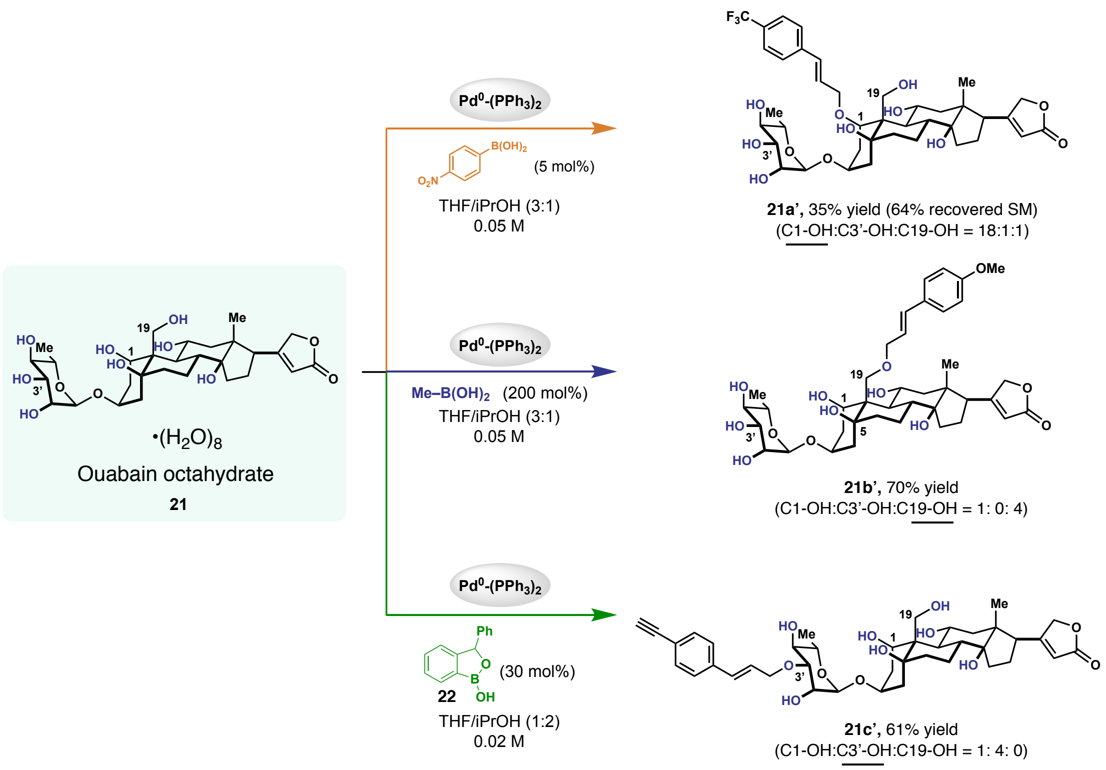

[a] Cinnamyl groups bearing electron-deficient (**21a'**) or electron-rich (**21b'**) aryl rings could be installed. A terminal alkyne group (**21c'**) was also incorporated. In the preparation of **21a'**, addition of 4-nitro phenylboronic acid gave improved yield of product. The connectivity of each product was confirmed by 1D and 2D-NMR analysis.

Supplementary Methods

Procedures and Characterization Data for Compounds in Figure 2c

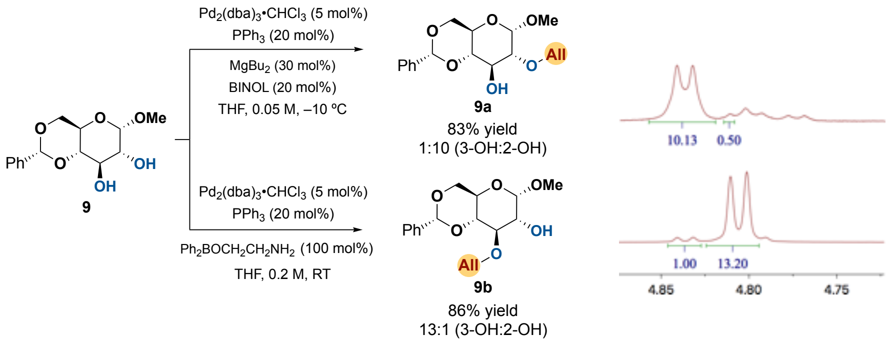

The full NMR spectra of the reaction mixtures were shown below.

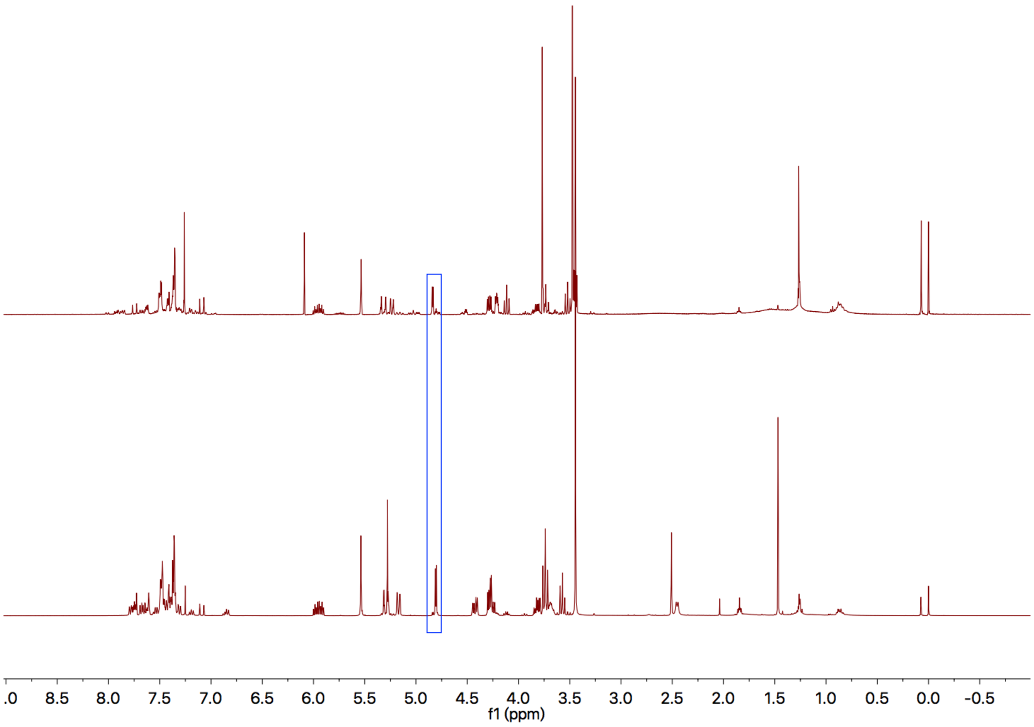

Methyl (*R*)-4,6-*O*-benzylidene-2-*O*-(allyl)-α-*D*-glucopyranoside (9a)

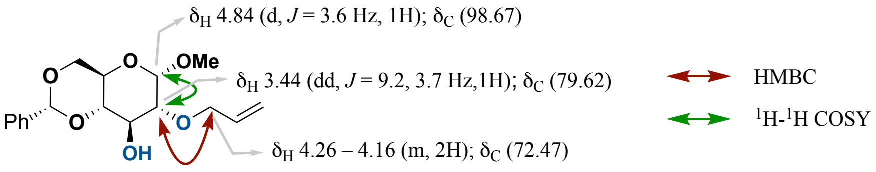

9a

In a N_2-filled glovebox, nBu$_2$Mg (1 M in heptane, Energy Chemicals Lot DL140184, 45 μL, 0.045 mmol, 0.3 equiv), (±)-BINOL (8.6 mg, Energy Chemicals Lot DI240132, 0.03 mmol, 0.2 equiv) and THF (200 μL) were weighed into a screw capped vial (labeled as **Vial A**)

containing a stir bar. The mixture was stirred for 5 min, at which time compound **9**[4] (42.6 mg, 0.15 mmol, 1.0 equiv) was added. The resulting solution was stirred for an additional 30 min.

To another vial, compound **2** (36.4 mg, 0.23 mmol, 1.5 equiv), $Pd_2(dba)_3$•$CHCl_3$ (7.8 mg, 7.5 μmol, 0.05 equiv), PPh_3 (7.8 mg, 0.03 mmol, 0.2 equiv), and THF (200 μL) were added in sequence. The mixture was stirred for 10 min, and then transferred to **Vial A**. An additional portion of THF (2.6 mL) was added to Vial A so that [**9**] was adjusted to 0.05 M. **Vial A** was tightly capped, taken out of the glove box, and stirred at –10 ˚C for 12 h (400 rpm). The reaction mixture was then concentrated in vacuo. An aliquot of the residue was taken for ^{1}H NMR analysis, which indicated that products **9a** and **9b** were formed in a ratio of ca. 10:1. Flash chromatography (SiO_2) using petroleum ether/EtOAc (6:1 to 3:1) as eluent afforded **9a** as a yellow solid (41 mg, 0.127 mmol, 83%).

(±)-BINOL was used as an additive in this work. Interestingly, we found (*S*)-BINOL gave essentially the same result, while (*R*)-BINOL gave only diallylated product.

Mp: 108–110 ºC. **^{1}H NMR (CDCl$_3$, 400 MHz)** δ: 7.54 – 7.46 (m, 2H), 7.40 – 7.31 (m, 3H), 5.53 (s, 1H), 5.32 (dq, *J* = 17.2, 1.5 Hz, 1H), 5.23 (dq, *J* = 10.3, 1.3 Hz, 1H), 4.84 (d, *J* = 3.6 Hz, 1H), 4.28 (dd, *J* = 10.0, 4.6 Hz, 1H), 4.26 – 4.16 (m, 2H), 4.12 (td, *J* = 9.3, 2.2 Hz, 1H), 3.83 (td, *J* = 9.8, 4.6 Hz, 1H), 3.73 (t, *J* = 10.2 Hz, 1H), 3.52 (t, *J* = 9.4 Hz, 1H), 3.48 – 3.40 (m, 1H), 3.44 (s, 3H), and 2.56 (d, *J* = 2.2 Hz, 1H). **^{13}C NMR (CDCl$_3$, 101 MHz)** δ: 137.2, 134.7, 129.3, 128.4, 126.5, 118.3, 102.2, 98.7, 81.4, 79.6, 72.5, 70.3, 69.2, 62.2, and 55.5. **IR (thin film, cm^{-1})**: 3374, 2913, 2828, 1452, 1371, 1096, 981, and 749. **HRMS (DART-TOF)** calculated for $C_{17}H_{22}NaO_6^+$[M+Na]$^+$ m/z 345.1309, found 345.1310. **[α]$_D^{25}$** = 70.7 (c = 0.30, $CHCl_3$).

Methyl (*R*)-4,6-*O*-benzylidene-3-*O*-(allyl)-α-*D*-glucopyranoside (9b)

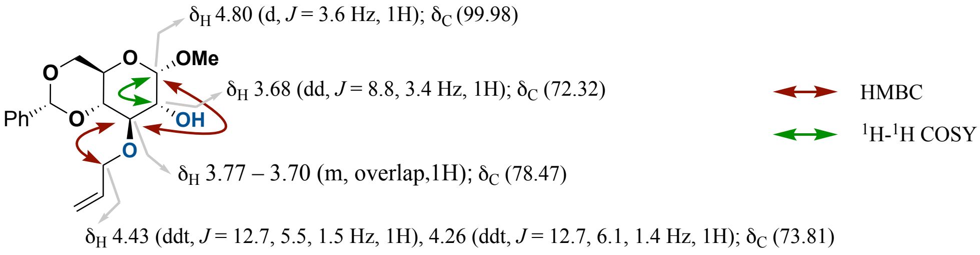

9b

In a N_2-filled glovebox, compound **9** (56.5 mg, 0.20 mmol, 1.0 equiv) and 2-(diphenylboryloxy)-ethanamine (2.4 mg, Aladdin Lot J1824016, 0.01 mmol, 5 mol%), and

THF (500 μL) were weighed into a screw capped vial (labeled as **Vial A**) containing a stir bar. The resulting solution was stirred for an additional 30 min.

To another vial, compound **2** (38.0 mg, 0.24 mmol, 1.2 equiv), $Pd_2(dba)_3$•$CHCl_3$ (5.2 mg, 5 μmol, 0.025 equiv), PPh_3 (5.2 mg, 0.02 mmol, 0.1 equiv), and THF (200 μL) were added in sequence. The mixture was stirred for 10 min, and then transferred to **Vial A**. An additional portion of THF (0.3 mL) was added to Vial A so that [**9**] was adjusted to 0.2 M. **Vial A** was tightly capped, taken out of the glove box, and stirred at 25 ˚C (outside temperature) for 12 h (400 rpm). The reaction mixture was then concentrated in vacuo. An aliquot of the residue was taken for ^{1}H NMR analysis, which indicated that products **9a** and **9b** were formed in a ratio of 1:13. Flash chromatography (SiO_2) using petroleum ether/EtOAc (3:1 to 1:1) as eluent afforded **9b** as a pale yellow oil (55.4 mg, 0.172 mmol, 86%).

^{1}H NMR ($CDCl_3$, 400 MHz) δ: 7.54 – 7.44 (m, 2H), 7.42 – 7.32 (m, 3H), 5.95 (ddt, J = 16.1, 10.5, 5.8 Hz, 1H), 5.54 (s, 1H), 5.29 (dt, J = 17.2, 1.7 Hz, 1H), 5.17 (dt, J = 10.4, 1.4 Hz, 1H), 4.80 (d, J = 3.6 Hz, 1H), 4.43 (ddt, J = 12.7, 5.5, 1.5 Hz, 1H), 4.29 (dd, J = 9.9, 4.5 Hz, 1H), 4.26 (ddt, J = 12.7, 6.1, 1.4 Hz, 1H), 3.81 (td, J = 9.6, 9.1, 4.3 Hz, 1H), 3.77 – 3.70 (m, 2H), 3.68 (dd, J = 8.8, 3.4 Hz, 1H), 3.57 (t, J = 9.1 Hz, 1H), 3.44 (s, 3H), and 2.46 (s, 1H). **^{13}C NMR ($CDCl_3$, 101 MHz)** δ: 137.5, 135.1, 129.0, 128.3, 126.1, 117.4, 101.4, 100.0, 82.0, 78.5, 73.8, 72.3, 69.1, 62.7, and 55.5. **IR (thin film, cm^{-1})**: 3426, 2930, 1451, 1367, 1265, 1141, 1077, 994, 760, and 733. **HRMS (DART-TOF)** calculated for $C_{17}H_{22}NaO_6^+$[M+Na]$^+$ m/z 345.1309, found 345.1315. **$[\alpha]_D^{25}$** =125.2 (c = 0.68, $CHCl_3$).

[When no Lewis acid is used, **9a** and **9b** were obtained in a ratio of 1:1.]

Procedures and Characterization Data for Compounds in Figure 3

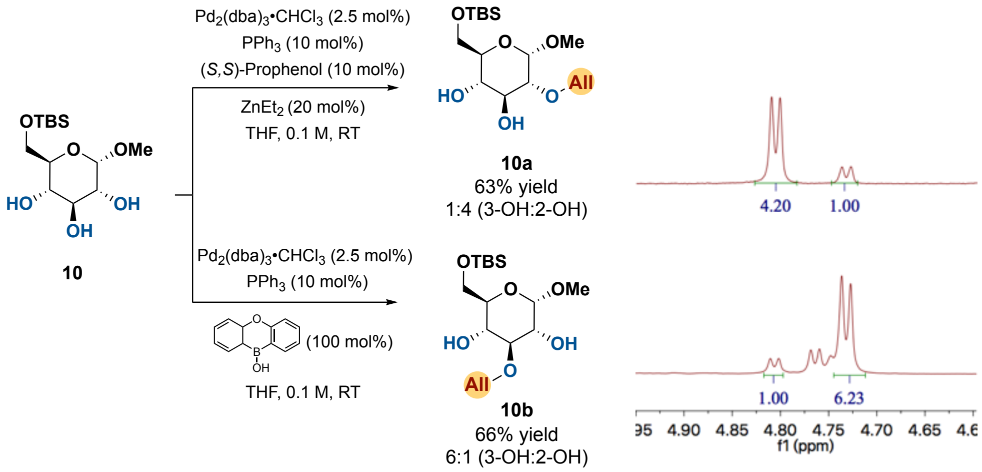

The full NMR spectra of the reaction mixtures were shown below. For the top spectra, NMR was taken after the reaction mixture was passed through a pad of silica gel to remove additives and unreacted starting materials.

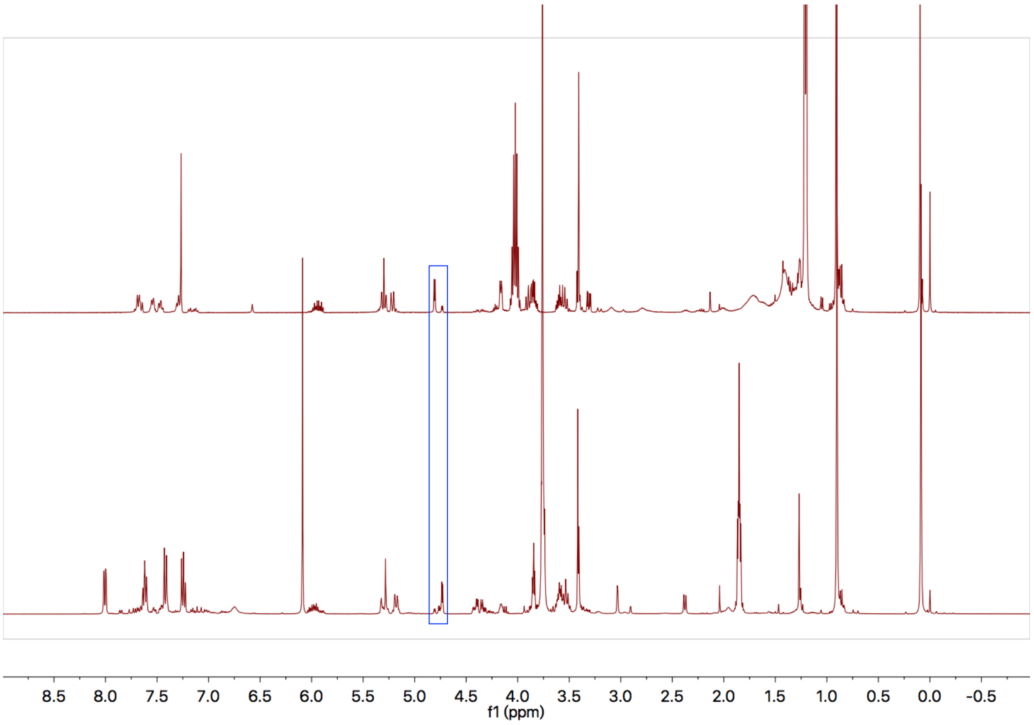

6-(*tert*-Butyldimethylsilyloxyl)-2-*O*-(allyl)-α-*D*-glucopyranoside (10a)

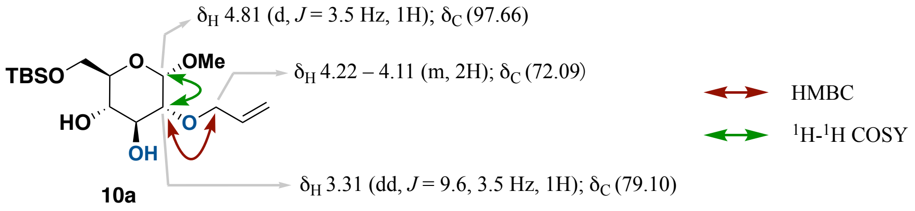

In a N_2-filled glovebox, $ZnEt_2$ (1 M in Toluene, Energy Chemicals Lot FK16010, 40 µL, 0.040 mmol, 0.2 equiv), (*S,S*)-Prophenol (12.7 mg, Sigma Aldrich Lot BGBC1470V, 0.02 mmol, 0.1 equiv) and THF (200 µL) were weighed into a screw capped vial (labeled as **Vial A**) containing a stir bar. The mixture was stirred for 5 min, at which time compound **10** (61.7 mg, 0.20 mmol, 1.0 equiv) was added. The resulting solution was stirred for an additional 30 min.

To another vial, compound **2** (51.0 mg, 0.32 mmol, 1.6 equiv), $Pd_2(dba)_3$•$CHCl_3$ (5.2 mg, 5 µmol, 0.025 equiv), PPh_3 (5.4 mg, 0.02 mmol, 0.1 equiv), and THF (200 µL) were added in sequence. The mixture was stirred for 10 min, and then transferred to **Vial A**. An additional portion of THF (1.6 mL) was added to Vial A so that [**10**] was adjusted to 0.1 M. **Vial A** was tightly capped, taken out of the glove box, and stirred at 25 ˚C (plate temperature) for 12 h (500 rpm). The reaction mixture was then concentrated in vacuo. An aliquot of the residue was taken for ^{1}H NMR analysis, which indicated that products **10a** and **10b** were formed in a ratio of 4:1. Flash chromatography (SiO_2) using petroleum ether/EtOAc (6:1 to 2:1) as eluent afforded **10a** as a yellow oil (43.7 mg, 0.125 mmol, 63%).

^{1}H NMR ($CDCl_3$, 400 MHz) δ: 5.94 (ddt, *J* = 17.4, 10.3, 6.0 Hz, 1H), 5.30 (dq, *J* = 17.2, 1.5 Hz, 1H), 5.22 (dq, *J* = 10.3, 1.3 Hz, 1H), 4.81 (d, *J* = 3.5 Hz, 1H), 4.22 – 4.11 (m, 2H), 3.81 (ddd, J = 9.7, 8.5, 2.0 Hz, 1H), 3.78 (dd, *J* = 10.7, 4.7 Hz, 1H), 3.73 (dd, *J* = 10.7, 4.5 Hz, 1H), 3.60 (td, *J* = 9.8, 5.1 Hz, 1H), 3.55 (td, *J* = 9.7, 9.1, 1.9 Hz, 1H), 3.41 (s, 3H), 3.31 (dd, *J* = 9.6, 3.5 Hz, 1H), 3.02 (d, *J* = 2.1 Hz, 1H), 2.68 (d, *J* = 2.2 Hz, 1H), 0.91 (s, 9H), and 0.10 (s, 6H). **^{13}C NMR ($CDCl_3$, 101 MHz)** δ: 134.8, 118.2, 97.7, 79.1, 73.0, 72.3, 72.1, 70.4, 64.2, 55.2, 26.0, 18.5, -5.3, and -5.3. **IR (thin film, cm^{-1})**: 3414, 2928, 2856, 1462, 1360, 1251, 1146, 1049, 834, and 777. **HRMS (DART-TOF)** calculated for $C_{16}H_{32}NaO_6Si^+$ $[M+Na]^+$ m/z 371.1860, found 371.1867. **$[\alpha]_D^{27}$** = 62.3 (c = 0.31, $CHCl_3$).

6-(*tert*-Butyldimethylsilyloxyl)-3-*O*-(allyl)-α-*D*-glucopyranoside (10b)

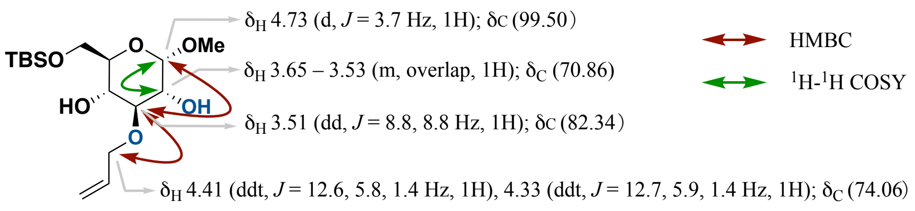

10b

In a N_2-filled glovebox, compound **10** (92.4 mg, 0.30 mmol, 1.0 equiv) and 10*H*-dibenzo[*b*,*e*][1,4]oxaborinin-10-ol[5] (58.8 mg, 0.30 mmol, 1.0 equiv), and THF (500 μL) were weighed into a screw capped vial (labeled as **Vial A**) containing a stir bar. The resulting solution was stirred for an additional 30 min.

To another vial, compound **2** (71.2 mg, 0.45 mmol, 1.5 equiv), $Pd_2(dba)_3$•$CHCl_3$ (7.8 mg, 7.5 μmol, 0.025 equiv), PPh_3 (7.9 mg, 0.03 mmol, 0.1 equiv), and THF (200 μL) were added in sequence. The mixture was stirred for 10 min, and then transferred to **Vial A**. An additional portion of THF (2.3 mL) was added to Vial A so that [**10**] was adjusted to 0.1 M. **Vial A** was tightly capped, taken out of the glove box, and stirred at 25 ˚C for 12 h (400 rpm). The reaction mixture was then concentrated in vacuo. An aliquot of the residue was taken for ^{1}H NMR analysis, which indicated that products **10a** and **10b** were formed in a ratio of 1:6. Flash chromatography (SiO_2) using petroleum ether/EtOAc (6:1 to 3:1) as eluent afforded **10b** as a pale yellow oil (69.0 mg, 0.198 mmol, 66%).

^{1}H NMR ($CDCl_3$, 400 MHz) δ: 5.99 (ddt, *J* = 17.2, 10.4, 5.9 Hz, 1H), 5.31 (dq, *J* = 17.2, 1.6 Hz, 1H), 5.19 (dq, *J* = 10.3, 1.3 Hz, 1H), 4.73 (d, *J* = 3.7 Hz, 1H), 4.41 (ddt, *J* = 12.6, 5.8, 1.4 Hz, 1H), 4.33 (ddt, *J* = 12.7, 5.9, 1.4 Hz, 1H), 3.87 (dd, *J* = 10.7, 4.6 Hz, 1H), 3.82 (dd, *J* = 10.7, 4.4 Hz, 1H), 3.65 – 3.53 (m, 3H), 3.51 (dd, *J* = 8.8, 8.8 Hz, 1H), 3.43 (s, 3H), 2.89 (d, *J* = 1.7 Hz, 1H), 2.16 (d, *J* = 8.1 Hz, 1H), 0.91 (s, 9H), and 0.09 (s, 6H). **^{13}C NMR ($CDCl_3$, 101 MHz)** δ: 135.4, 117.4, 99.5, 82.3, 74.1, 72.5, 72.2, 70.9, 64.3, 55.3, 26.04, 18.47, and -5.28. **IR (thin film, cm^{-1})**: 3431, 2929, 2856, 1463, 1360, 1275, 1258, 1143, 1053, 835, and 750. **HRMS (DART-TOF)** calculated for $C_{16}H_{32}NaO_6Si^+$ $[M+Na]^+$ m/z 371.1860, found 371.1862. **$[\alpha]_D^{27}$** = 70.0 (c = 0.26, $CHCl_3$).

[When no Lewis acid is used, **10a** and **10b** were obtained in a ratio of 2:3]

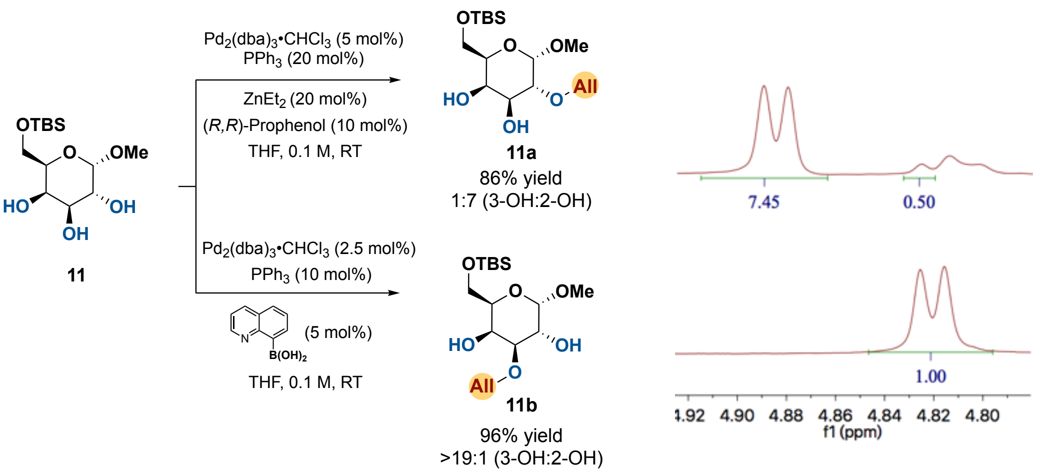

The full NMR spectra of the reaction mixtures were shown below.

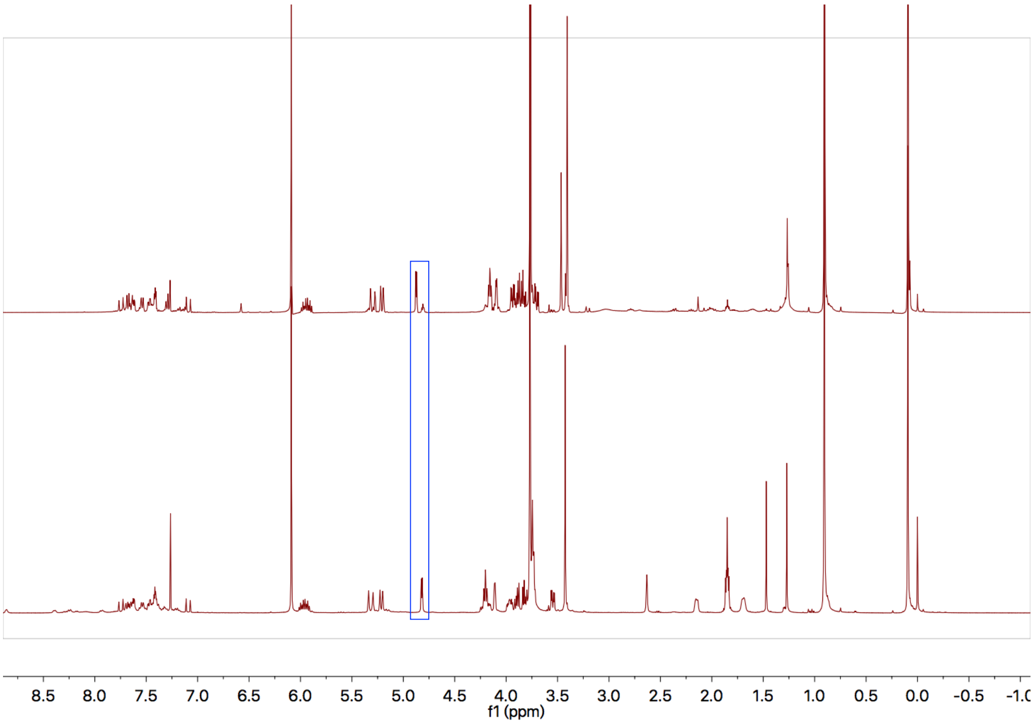

6-(*tert*-Butyldimethylsilyloxyl)-2-*O*-(allyl) -α-*D*-galactopyranoside (11a)

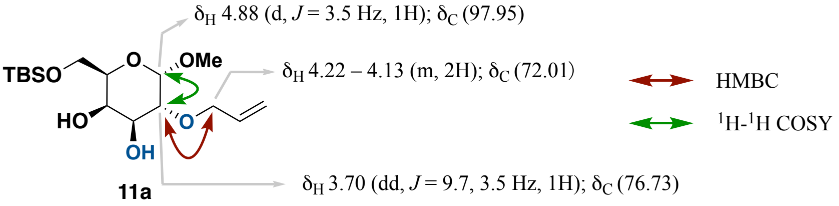

In a N_2-filled glovebox, $ZnEt_2$ (1 M in Toluene, Energy Chemicals Lot FK16010, 40 μL, 0.040 mmol, 0.2 equiv), (*R,R*)-Prophenol (12.7 mg, Sigma Aldrich Lot MKBB9236V, 0.02

mmol, 0.1 equiv) and THF (200 μL) were weighed into a screw capped vial (labeled as **Vial A**) containing a stir bar. The mixture was stirred for 5 min, at which time compound **11** (61.7 mg, 0.20 mmol, 1.0 equiv) was added. The resulting solution was stirred for an additional 30 min.

To another vial, compound **2** (47.5 mg, 0.30 mmol, 1.5 equiv), $Pd_2(dba)_3$•$CHCl_3$ (10.4 mg, 0.01 mmol, 0.05 equiv), PPh_3 (10.4 mg, 0.04 mmol, 0.20 equiv), and THF (200 μL) were added in sequence. The mixture was stirred for 10 min, and then transferred to **Vial A**. An additional portion of THF (1.6 mL) was added to Vial A so that [**11**] was adjusted to 0.1 M. **Vial A** was tightly capped, taken out of the glove box, and stirred at 25 ˚C for 12 h (400 rpm). The reaction mixture was then concentrated in vacuo. An aliquot of the residue was taken for ^{1}H NMR analysis, which indicated that products **11a** and **11b** were formed in a ratio of 7:1. Flash chromatography (SiO_2) using petroleum ether/EtOAc (3:1 to 2:1) as eluent afforded **11a** as a pale yellow oil (59.9 mg, 0.172 mmol, 86%).

^{1}H NMR ($CDCl_3$, 400 MHz) δ: 5.94 (ddt, *J* = 17.2, 10.3, 5.9 Hz, 1H), 5.30 (dq, *J* = 17.2, 1.5 Hz, 1H), 5.21 (dq, *J* = 10.3, 1.3 Hz, 1H), 4.88 (d, *J* = 3.5 Hz, 1H), 4.22 – 4.13 (m, 2H), 4.11 (dd, *J* = 2.9, 1.4 Hz, 1H), 3.94 (dd, *J* = 9.8, 3.3 Hz, 1H), 3.90 (dd, *J* = 10.4, 5.8 Hz, 1H), 3.84 (dd, *J* = 10.5, 5.3 Hz, 1H), 3.76 (td, *J* = 5.5, 1.3 Hz, 1H), 3.70 (dd, *J* = 9.7, 3.5 Hz, 1H), 3.41 (s, 3H), 2.90 (s, 1H), 0.90 (s, 9H), and 0.09 (s, 6H). **^{13}C NMR ($CDCl_3$, 101 MHz)** δ: 134.8, 118.2, 98.0, 76.7, 72.0, 69.7, 69.6, 69.8, 63.2, 55.4, 26.0, 18.4, -5.3, and -5.32. **IR (thin film, cm^{-1})**: 3445, 2928, 2856, 1471, 1359, 1251, 1091, and 837. **HRMS (DART-TOF)** calculated for $C_{16}H_{32}NaO_6Si^+$[M+Na]$^+$ m/z 371.1860, found 371.1862. **$[\alpha]_D^{23}$** = 77.9 (c = 0.34, $CHCl_3$).

6-(*tert*-Butyldimethylsilyloxyl)-3-*O*-(allyl) -α-*D*-galactopyranoside (11b)

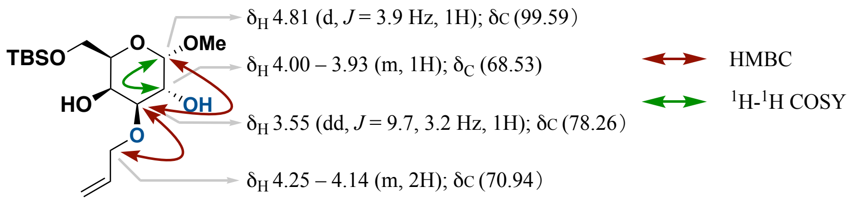

11b

In a N_2-filled glovebox, compound **11** (61.7 mg, 0.20 mmol, 1.0 equiv) and quinoline-8-boronic acid (1.7 mg, Bidepharma Lot BD8489, 0.01 mmol, 0.05 equiv), and THF (500 μL) were weighed into a screw capped vial (labeled as **Vial A**) containing a stir bar. The resulting solution was stirred for an additional 30 min.

To another vial, compound **2** (63.3 mg, 0.40 mmol, 2.0 equiv), $Pd_2(dba)_3$•$CHCl_3$ (5.2 mg, 0.005 mmol, 0.025 equiv), PPh_3 (5.2 mg, 0.02 mmol, 0.1 equiv), and THF (200 μL) were added in sequence. The mixture was stirred for 10 min, and then transferred to **Vial A**. An additional portion of THF (1.3 mL) was added to Vial A so that [**11**] was adjusted to 0.1 M. **Vial A** was tightly capped, taken out of the glove box, and stirred at 25 ˚C for 12 h (400 rpm). The reaction mixture was then concentrated in vacuo. An aliquot of the residue was taken for ^{1}H NMR analysis, which indicated that products **11a** and **11b** were formed in a ratio of <1:19. Flash chromatography (SiO_2) using petroleum ether/EtOAc (3:1 to 1:1) as eluent afforded **11b** as a pale yellow oil (67.4 mg, 0.192 mmol, 96%).

^{1}H NMR ($CDCl_3$, 400 MHz) δ: 5.96 (ddt, *J* = 16.3, 10.8, 5.8 Hz, 1H), 5.32 (dq, *J* = 17.2, 1.6 Hz, 1H), 5.21 (dq, *J* = 10.4, 1.4 Hz, 1H), 4.81 (d, *J* = 3.9 Hz, 1H), 4.25 – 4.14 (m, 2H), 4.10 (d, *J* = 2.7 Hz, 1H), 4.00 – 3.93 (m, 1H), 3.89 (dd, *J* = 10.3, 6.0 Hz, 1H), 3.81 (dd, *J* = 10.3, 5.6 Hz, 1H), 3.73 (t, *J* = 5.8 Hz, 1H), 3.55 (dd, *J* = 9.7, 3.2 Hz, 1H), 3.42 (s, 3H), 2.69 (s, 1H), 2.29 (s, 1H), 0.91 (s, 9H), and 0.09 (s, 6H). **^{13}C NMR ($CDCl_3$, 101 MHz)** δ: 134.7, 117.8, 99.6, 78.3, 70.9, 70.2, 68.5, 66.9, 62.8, 55.3, 25.9, 18.7, -5.3, and -5.4. **IR (thin film, cm^{-1})**: 3458, 2929, 2855, 1471, 1249, 1147, 1088, 1051, 836, and 760. **HRMS (DART-TOF)** calculated for $C_{16}H_{32}NaO_6Si^+$[M+Na]$^+$ m/z 371.1860, found 371.1859. **$[\alpha]_D^{25}$** = 140.3 (c = 1.55, $CHCl_3$).

[When no Lewis acid was used, **11b** and **11a** were obtained with a ratio of 3-*O*:2-*O* = 2.3:1.0.]

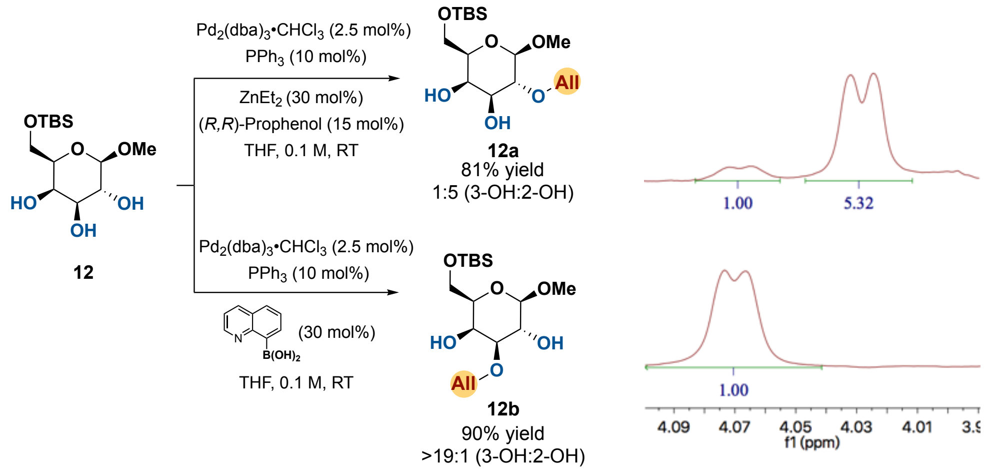

The full NMR spectra of the reaction mixtures were shown below.

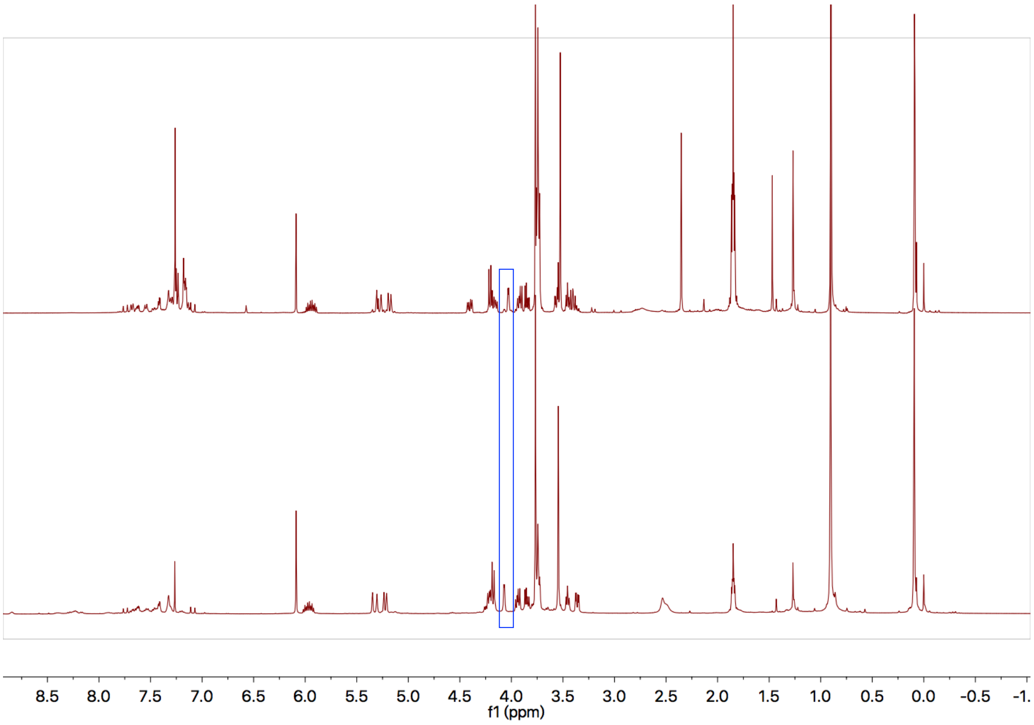

6-(*tert*-Butyldimethylsilyloxyl)-2-*O*-(allyl) -β-*D*-galactopyranoside (12a)

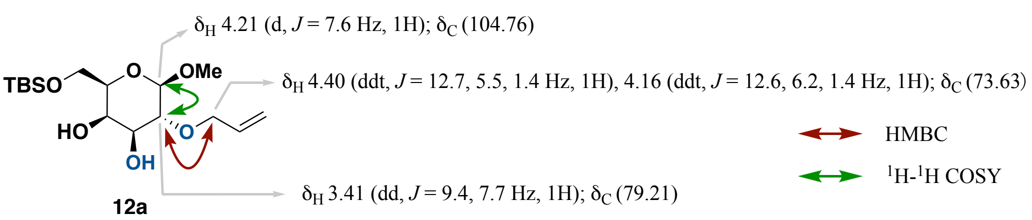

In a N_2-filled glovebox, $ZnEt_2$ (1 M in Toluene, Energy Chemicals Lot FK16010, 60 μL, 0.06 mmol, 0.3 equiv), (*R*,*R*)-Prophenol (19.1 mg, Sigma Aldrich Lot MKBB9236V, 0.03

mmol, 0.15 equiv) and THF (200 µL) were weighed into a screw capped vial (labeled as **Vial A**) containing a stir bar. The mixture was stirred for 5 min, at which time compound **12** (61.7 mg, 0.20 mmol, 1.0 equiv) was added.

To another vial, compound **2** (47.5 mg, 0.30 mmol, 1.5 equiv), $Pd_2(dba)_3$•$CHCl_3$ (5.2 mg, 5 µmol, 0.025 equiv), PPh_3 (5.2 mg, 0.02 mmol, 0.1 equiv), and THF (200 µL) were added in sequence. The mixture was stirred for 10 min, and then transferred to **Vial A**. An additional portion of THF (1.6 mL) was added to Vial A so that [**12**] was adjusted to 0.1 M. **Vial A** was tightly capped, taken out of the glove box, and stirred at 25 ˚C for 12 h (400 rpm, the reaction is sensitive to temperature). The reaction mixture was then concentrated in vacuo. An aliquot of the residue was taken for ^{1}H NMR analysis, which indicated that products **12a** and **12b** were formed in a ratio of 5:1. Flash chromatography (SiO_2) using petroleum ether/EtOAc (6:1 to 3:1) as eluent afforded **12a** as a pale yellow oil (56.5 mg, 0.162 mmol, 81%).

^{1}H NMR ($CDCl_3$, 400 MHz) δ: 5.94 (dddd, J = 16.7, 10.3, 6.2, 5.5 Hz, 1H), 5.29 (dq, J = 17.2, 1.6 Hz, 1H), 5.18 (dq, J = 10.3, 1.3 Hz, 1H), 4.40 (ddt, J = 12.7, 5.5, 1.4 Hz, 1H), 4.21 (d, J = 7.6 Hz, 1H), 4.16 (ddt, J = 12.6, 6.2, 1.4 Hz, 1H), 4.03 (d, J = 3.3 Hz, 1H), 3.92 (dd, J = 10.4, 6.2 Hz, 1H), 3.84 (dd, J = 10.4, 5.3 Hz, 1H), 3.57 (dd, J = 9.4, 3.3 Hz, 1H), 3.53 (s, 3H), 3.46 (ddd, J = 6.3, 5.3, 1.1 Hz, 1H), 3.41 (dd, J = 9.4, 7.7 Hz, 1H), 2.78 (s, 2H), 0.90 (s, 9H), and 0.09 (d, J = 1.6 Hz, 6H). **^{13}C NMR ($CDCl_3$, 101 MHz)** δ: 135.1, 117.5, 104.8, 79.2, 74.5, 73.6, 73.5, 68.7, 62.5, 56.9, 26.0, 18.4, and -5.3. **IR (thin film, cm^{-1})**: 3430, 2929, 2856, 1471, 1388, 1257, 1100, 1091, 837 and 751. **HRMS (DART-TOF)** calculated for $C_{16}H_{32}NaO_6Si^+$[M+Na]$^+$ m/z 371.1860, found 371.1862. **$[\alpha]_D^{27}$** = –3.7 (c = 0.67, $CHCl_3$).

6-(*tert*-Butyldimethylsilyloxyl)-3-*O*-(allyl) -β-*D*-galactopyranoside (12b)

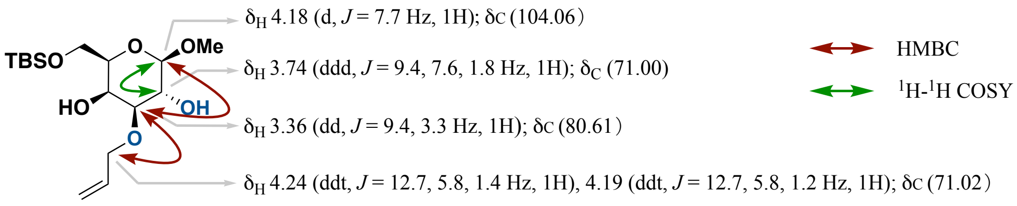

12b

In a N_2-filled glovebox, compound **12** (61.7 mg, 0.20 mmol, 1.0 equiv) and quinoline-8-boronic acid (10.4 mg, Bidepharma Lot BD8489, 0.06 mmol, 0.3 equiv), and THF (500 µL) were weighed into a screw capped vial (labeled as **Vial A**) containing a stir bar. The resulting solution was stirred for an additional 30 min.

To another vial, compound **2** (47.5 mg, 0.3 mmol, 1.5 equiv), $Pd_2(dba)_3$•$CHCl_3$ (5.2 mg, 0.005 mmol, 0.025 equiv), PPh_3 (5.2 mg, 0.02 mmol, 0.1 equiv), and THF (200 μL) were added in sequence. The mixture was stirred for 10 min, and then transferred to **Vial A**. An additional portion of THF (1.3 mL) was added to Vial A so that [**12**] was adjusted to 0.1 M. **Vial A** was tightly capped, taken out of the glove box, and stirred at 25 ˚C for 12 h (400 rpm). The reaction mixture was then concentrated in vacuo. An aliquot of the residue was taken for ^{1}H NMR analysis, which indicated that products **12a** and **12b** were formed in a ratio of <1:19. Flash chromatography (SiO_2) using petroleum ether/EtOAc (5:1 to 2:1) as eluent afforded **12b** as a pale yellow oil (62.8 mg, 0.18 mmol, 90%).

^{1}H NMR ($CDCl_3$, 400 MHz) δ: 5.97 (ddt, *J* = 17.1, 10.3, 5.8 Hz, 1H), 5.33 (dq, *J* = 17.2, 1.6 Hz, 1H), 5.23 (dq, *J* = 10.3, 1.3 Hz, 1H), 4.24 (ddt, *J* = 12.7, 5.8, 1.4 Hz, 1H), 4.19 (ddt, *J* = 12.7, 5.8, 1.2 Hz, 1H), 4.18 (d, *J* = 7.7 Hz, 1H), 4.07 (m, 1H), 3.94 (dd, *J* = 10.3, 6.4 Hz, 1H), 3.85 (dd, *J* = 10.3, 5.5 Hz, 1H), 3.74 (ddd, *J* = 9.4, 7.6, 1.8 Hz, 1H), 3.55 (s, 3H), 3.46 (t, *J* = 6.0 Hz, 1H), 3.36 (dd, *J* = 9.4, 3.3 Hz, 1H), 2.52 (d, *J* = 2.3 Hz, 1H), 2.45 (d, *J* = 2.1 Hz, 1H), 0.90 (s, 9H), and 0.09 (d, *J* = 1.3 Hz, 6H). **^{13}C NMR ($CDCl_3$, 101 MHz)** δ: 134.6, 118.0, 104.1, 80.6, 74.8, 71.0, 71.0, 66.0, 62.4, 57.0, 26.0, 18.4, -5.2, and -5.3. **IR (thin film, cm^{-1})**: 3453, 2929, 2856, 1471, 1388, 1252, 1073, 836, and 778. **HRMS (DART-TOF)** calculated for $C_{16}H_{32}NaO_6Si^+$[M+Na]$^+$ m/z 371.1860, found 371.1886. **$[\alpha]_D^{25}$** = 0.2 (c = 1.46, $CHCl_3$).

[When no Lewis acid was used, **12a** and **12b** were obtained with a ratio of 2.5:1.0.]

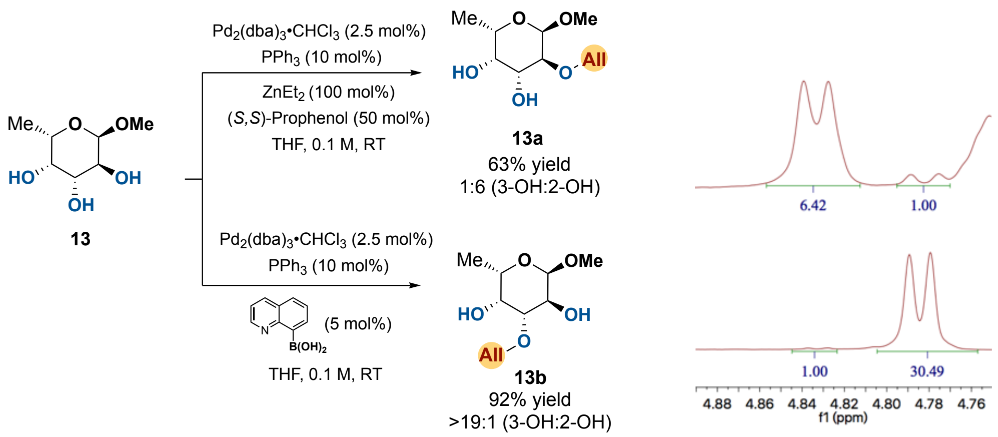

The full NMR spectra of the reaction mixtures were shown below.

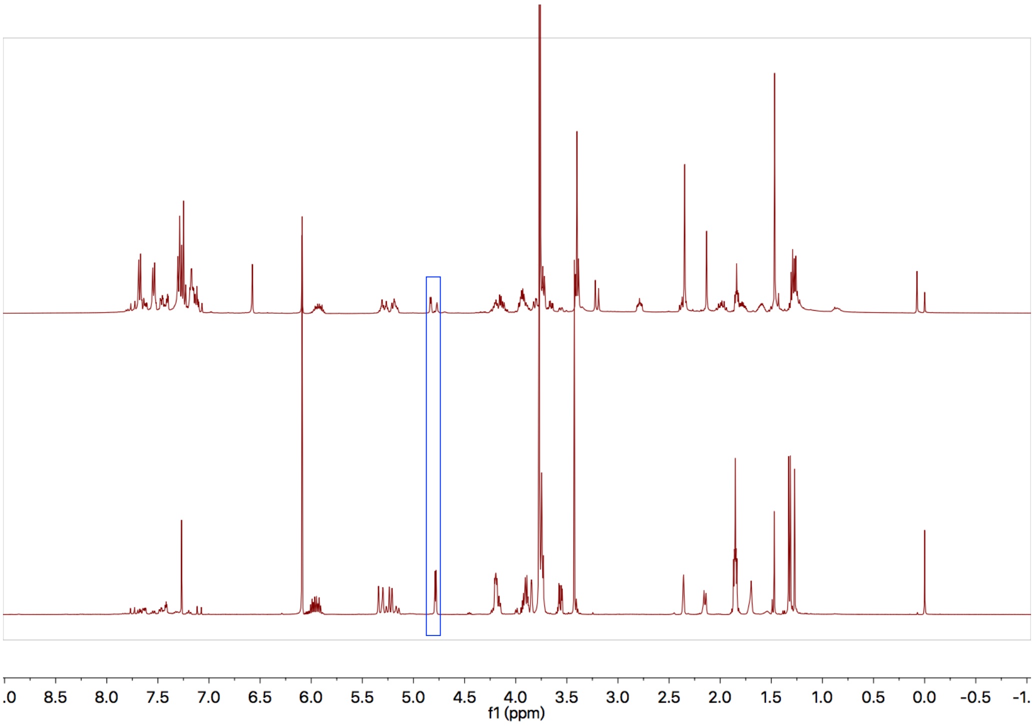

Methyl 2-*O*-(allyl)-α-*L*-fucopyranoside (13a)

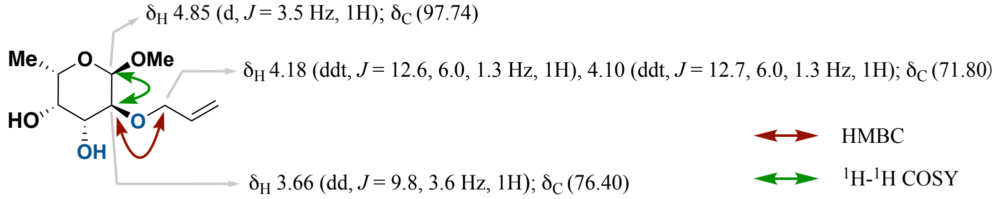

13a

In a N_2-filled glovebox, $ZnEt_2$ (1 M in Toluene, Energy Chemicals Lot FK16010, 300 μL, 0.30 mmol, 1.0 equiv), (*S*,*S*)-Prophenol (96.0 mg, Sigma Aldrich Lot BGBC1470V, 0.15 mmol, 0.5 equiv) and THF (200 μL) were weighed into a screw capped vial (labeled as **Vial A**) containing a stir bar. The mixture was stirred for 5 min, at which time compound **13** (53.4 mg, TCI Lot WOA7G-TJ, 0.30 mmol, 1.0 equiv) was added. The resulting solution was stirred for an additional 30 min.

To another vial, compound **2** (72.0 mg, 0.45 mmol, 1.5 equiv), $Pd_2(dba)_3$•$CHCl_3$ (7.8 mg, 7.5 μmol, 0.025 equiv), PPh_3 (7.9 mg, 0.03 mmol, 0.1 equiv), and THF (200 μL) were added in sequence. The mixture was stirred for 10 min, and then transferred to **Vial A**. An additional portion of THF (2.3 mL) was added to Vial A so that [**13**] was adjusted to 0.1 M. **Vial A** was tightly capped, taken out of the glove box, and stirred at 25 ˚C (plate temperature) for 12 h (400 rpm). The reaction mixture was then concentrated in vacuo. An aliquot of the residue was taken for ^{1}H NMR analysis, which indicated that products **13a** and **13b** were formed in a ratio of 6:1. Flash chromatography (SiO_2) using petroleum ether/EtOAc (3:1 to 1:1) as eluent afforded **13a** as a pale yellow oil (41.7 mg, 0.191 mmol, 63%).

^{1}H NMR ($CDCl_3$, 400 MHz) δ: 5.94 (ddt, *J* = 16.6, 10.3, 6.0 Hz, 1H), 5.30 (dq, *J* = 17.1, 1.6 Hz, 1H), 5.22 (dq, *J* = 10.4, 1.3 Hz, 1H), 4.85 (d, *J* = 3.5 Hz, 1H), 4.18 (ddt, *J* = 12.6, 6.0, 1.3 Hz, 1H), 4.10 (ddt, *J* = 12.7, 6.0, 1.3 Hz, 1H), 3.98 (dd, *J* = 9.9, 3.2 Hz, 1H), 3.86 – 3.81 (m, 1H), 3.66 (dd, *J* = 9.8, 3.6 Hz, 1H), 3.41 (s, 3H), and 1.30 (d, *J* = 6.6 Hz, 3H). **^{13}C NMR ($CDCl_3$, 101 MHz)** δ: 134.7, 118.3, 97.7, 76.4, 71.8, 71.7, 69.6, 65.5, 55.5, and 16.3. **IR (thin film, cm^{-1})**: 3420, 2919, 2839, 1449, 1390, 1134, 1100, 1057, and 974. **HRMS (DART-TOF)** calculated for $C_{10}H_{18}NaO_5^+$[M+Na]$^+$ m/z 241.1046, found 241.1050. **$[\alpha]_D^{23}$** = –25.2 (c = 0.31, $CHCl_3$).

Methyl 3-*O*-(allyl)-α-*L*-fucopyranoside (13b)

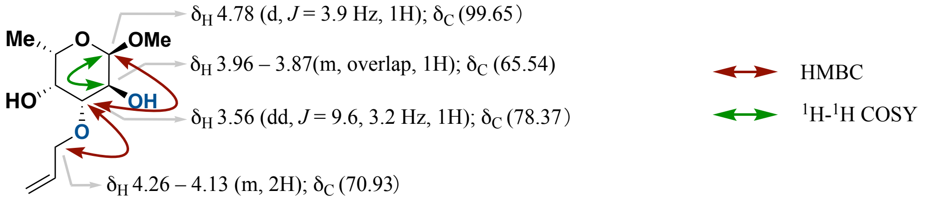

13b

In a N_2-filled glovebox, compound **13** (35.6 mg, 0.20 mmol, 1.0 equiv) and quinoline-8-boronic acid (1.7 mg, Bidepharma Lot BD8489, 0.01 mmol, 0.05 equiv), and THF (500 μL) were weighed into a screw capped vial (labeled as **Vial A**) containing a stir bar. The resulting solution was stirred for an additional 30 min.

To another vial, compound **2** (63.3 mg, 0.40 mmol, 2.0 equiv), $Pd_2(dba)_3$•$CHCl_3$ (5.2 mg, 5 μmol, 0.025 equiv), PPh_3 (5.2 mg, 0.02 mmol, 0.1 equiv), and THF (200 μL) were added in sequence. The mixture was stirred for 10 min, and then transferred to **Vial A**. An additional portion of THF (1.3 mL) was added to Vial A so that [**13**] was adjusted to 0.1 M. **Vial A** was tightly capped, taken out of the glove box, and stirred at 25 ˚C for 12 h (400 rpm). The reaction mixture was then concentrated in vacuo. An aliquot of the residue was taken for ^{1}H NMR

analysis, which indicated that products **13a** and **13b** were formed in a ratio of <1:19. Flash chromatography (SiO_2) using petroleum ether/EtOAc (2:1 to 1:1) as eluent afforded **13b** as a colorless oil (40.1 mg, 0.184 mmol, 92%).

^{1}H NMR ($CDCl_3$, 400 MHz) δ: 5.96 (ddt, *J* = 17.3, 10.3, 5.8 Hz, 1H), 5.32 (dq, *J* = 17.2, 1.6 Hz, 1H), 5.22 (dq, *J* = 10.3, 1.3 Hz, 1H), 4.78 (d, *J* = 3.9 Hz, 1H), 4.26 – 4.13 (m, 2H), 3.96 – 3.87 (m, 2H), 3.85 (dt, *J* = 3.1, 1.4 Hz, 1H), 3.56 (dd, *J* = 9.6, 3.2 Hz, 1H), 3.43 (s, 3H), 2.42 (t, *J* = 1.5 Hz, 1H), 2.22 (d, *J* = 7.8 Hz, 1H), and 1.32 (d, *J* = 6.6 Hz, 3H). **^{13}C NMR ($CDCl_3$, 101 MHz)** δ: 134.7, 117.8, 99.7, 78.4, 70.9, 69.5, 68.2, 65.5, 55.4, and 16.3. **IR (thin film, cm^{-1})**: 3407, 2920, 2851, 1632, 1422, 1360, 1269, 1082, 1043, 956, and 748. **HRMS (DART-TOF)** calculated for $C_{10}H_{18}NaO_5^+$[M+Na]$^+$ m/z 241.1046, found 241.1055. **$[\alpha]_D^{24}$** = –320.9 (c = 0.43, $CHCl_3$).

[When no additive is used, **13b** and **13a** were obtained at a ratio of 3-*O*:2-*O* = 2.0:1.0.]

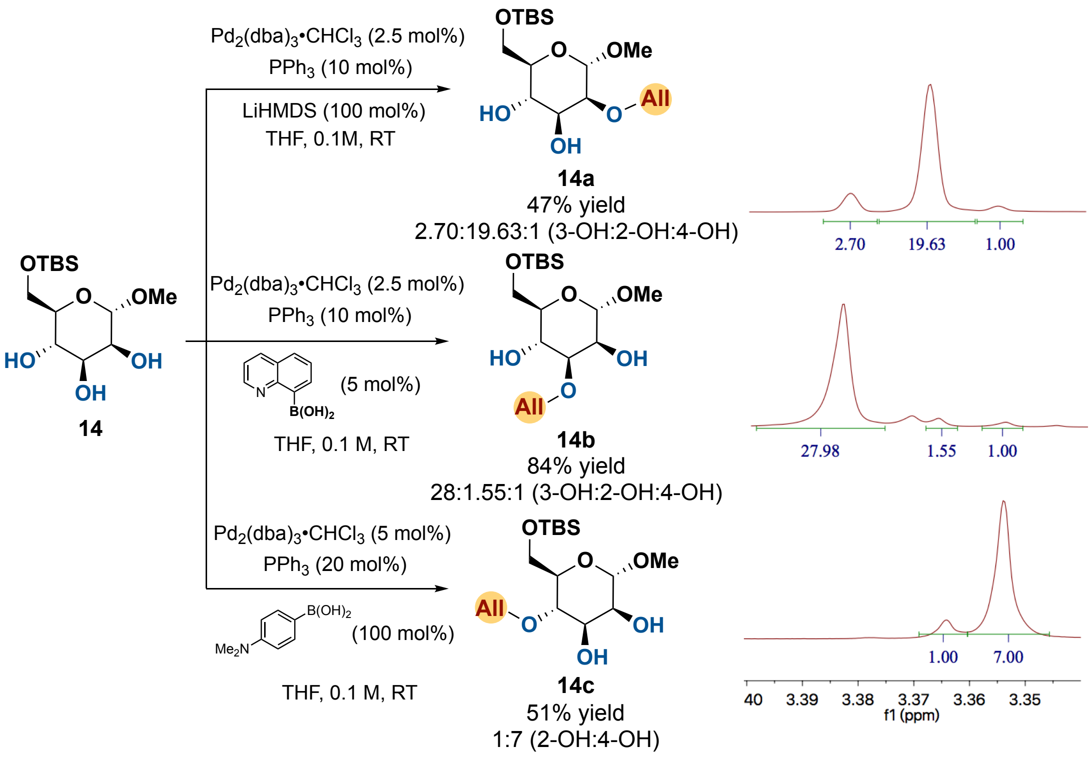

The full NMR spectra of the reaction mixtures were shown below. For the top and bottom spectra , NMR was taken after purification by the reaction mixture was passed through a pad of silica gel to remove additives and unreacted starting materials.

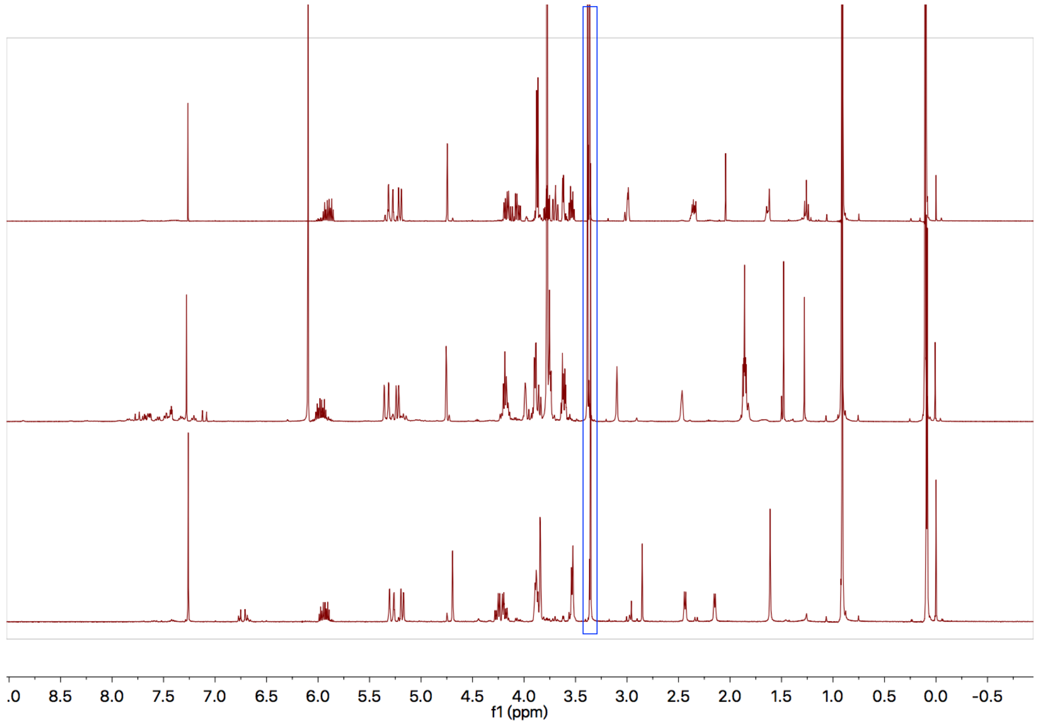

6-(*tert*-Butyldimethylsilyloxyl)-2-*O*-(allyl) -α-*D*-mannopyranoside (14a)

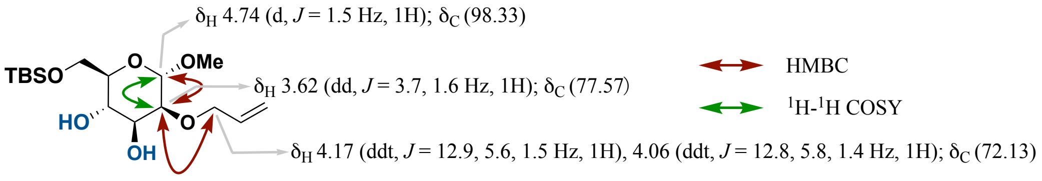

14a

In a N_2-filled glovebox, compound **14** (61.7 mg, 0.20 mmol, 1.0 equiv) and LiHMDS (33.6 mg, Sigma Aldrich Lot SHBH9931, 0.20 mmol, 1.0 equiv), and THF (500 μL) were weighed into a screw capped vial (labeled as **Vial A**) containing a stir bar. The resulting solution was stirred for an additional 3 min.

To another vial, compound **2** (48.0 mg, 0.30 mmol, 1.5 equiv), $Pd_2(dba)_3$•$CHCl_3$ (5.2 mg, 5 μmol, 0.025 equiv), PPh_3 (5.2 mg, 0.02 mmol, 0.1 equiv), and THF (500 μL) were added in sequence. The mixture was stirred for 10 min, and then transferred to **Vial A**. An additional portion of THF (1.0 mL) was added to Vial A so that [**14**] was adjusted to 0.1 M. **Vial A** was tightly capped, taken out of the glove box, and stirred at 20 °C for 3 h (600 rpm). The reaction mixture was then concentrated in vacuo. The residue was passed through a pad of silica gel and then concentrated. An aliquot of the residue was taken for ^{1}H NMR analysis, which indicated that products **14a**, **14b**, and **14c** were formed in a ratio of 19.6:2.7:1.0. Flash chromatography (SiO_2) using petroleum ether/EtOAc (6:1 to 3:1 to 1:1) as eluent afforded diallylated product (14.0 mg, 18%), **14a** as a pale yellow oil (32.9 mg, 0.09 mmol, 47%), and starting material **14** (16.1 mg, 26%).

^{1}H NMR ($CDCl_3$, 400 MHz) δ: 5.90 (ddt, *J* = 17.2, 10.3, 5.6 Hz, 1H), 5.29 (dq, *J* = 17.2, 1.6 Hz, 1H), 5.20 (dq, *J* = 10.3, 1.3 Hz, 1H), 4.74 (d, *J* = 1.5 Hz, 1H), 4.17 (ddt, *J* = 12.9, 5.6, 1.5 Hz, 1H), 4.06 (ddt, *J* = 12.8, 5.8, 1.4 Hz, 1H), 3.91 – 3.83 (m, 2H), 3.82 – 3.73 (m, 1H), 3.69 (td, *J* = 9.3, 1.7 Hz, 1H), 3.62 (dd, *J* = 3.7, 1.6 Hz, 1H), 3.53 (dt, *J* = 9.8, 5.2, Hz, 1H), 3.36 (s, 3H), 0.91 (s, 9H), and 0.10 (s, 6H). **^{13}C NMR ($CDCl_3$, 101 MHz)** δ: 134.4, 117.8, 98.3, 77.6, 72.1, 71.5, 71.3, 70.8, 64.5, 54.9, 26.0, 18.4, and -5.3. **IR (thin film, cm^{-1})**: 3396, 2929, 2856, 1471, 1459, 1269, 1248, 1100, 1052, 836, and 760. **$[\alpha]_D^{14}$** = 5.6 (c = 0.34, $CHCl_3$).

6-(*tert*-Butyldimethylsilyloxyl)-3-*O*-(allyl) -α-*D*-mannopyranoside (14b)

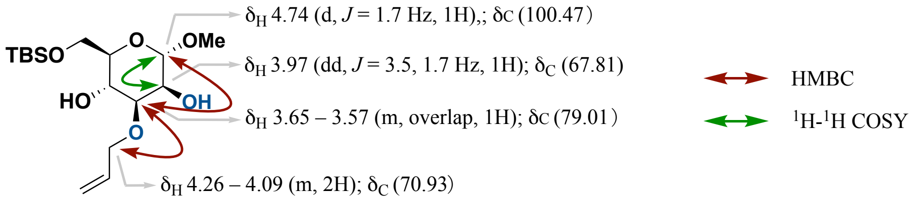

14b

In a N_2-filled glovebox, compound **14** (61.7 mg, 0.20 mmol, 1.0 equiv) and quinoline-8-boronic acid (1.7 mg, Bidepharma Lot BD8489, 0.01 mmol, 0.05 equiv), and THF (500 μL) were weighed into a screw capped vial (labeled as **Vial A**) containing a stir bar. The resulting solution was stirred for an additional 30 min.

To another vial, compound **2** (63.3 mg, 0.40 mmol, 2.0 equiv), $Pd_2(dba)_3$•$CHCl_3$ (5.2 mg, 5 μmol, 0.025 equiv), PPh_3 (5.2 mg, 0.02 mmol, 0.1 equiv), and THF (200 μL) were added in sequence. The mixture was stirred for 10 min, and then transferred to **Vial A**. An additional portion of THF (1.3 mL) was added to Vial A so that [**14**] was adjusted to 0.1 M. **Vial A** was tightly capped, taken out of the glove box, and stirred at 25 ˚C for 12 h (400 rpm). The reaction mixture was then concentrated in vacuo. An aliquot of the residue was taken for ^{1}H NMR analysis, which indicated that products **14a**, **14b**, and **14c** were formed in a ratio of 1.55:28:1. Flash chromatography (SiO_2) using petroleum ether/EtOAc (4:1 to 2:1) as eluent afforded **14b** as a pale yellow oil (58.6 mg, 0.168 mmol, 84%).

^{1}H NMR ($CDCl_3$, 400 MHz) δ: 5.97 (ddt, *J* = 16.2, 10.5, 5.8 Hz, 1H), 5.33 (dq, *J* = 17.3, 1.5 Hz, 1H), 5.22 (dq, *J* = 10.3, 1.3 Hz, 1H), 4.74 (d, *J* = 1.7 Hz, 1H), 4.26 – 4.09 (m, 2H), 3.97 (dd, *J* = 3.5, 1.7 Hz, 1H), 3.93 – 3.82 (m, 2H), 3.83 (d, *J* = 9.4 Hz, 1H), 3.65 – 3.57 (m, 2H), 3.38 (s, 3H), 3.04 (s, 1H), 2.40 (s, 1H), 0.91 (s, 9H), and 0.10 (s, 6H). **^{13}C NMR ($CDCl_3$, 101 MHz)** δ: 134.6, 118.0, 100.5, 79.0, 71.0, 70.8, 69.4, 67.9, 65.0, 55.0, 26.0, 18.4, -5.3 and -5.3. **IR (thin film, cm^{-1})**: 3432, 2928, 2856, 1472, 1248, 1136, 1105, 1049, 833, 778, and 750. **HRMS (DART-TOF)** calculated for $C_{16}H_{32}NaO_6Si^+$[M+Na]$^+$ m/z 371.1860, found 371.1863. **$[\alpha]_D^{25}$** = 46.1 (c = 1.27, $CHCl_3$).

6-(*tert*-Butyldimethylsilyloxyl)-4-*O*-(allyl) -*α*-*D*-mannopyranoside (14c)

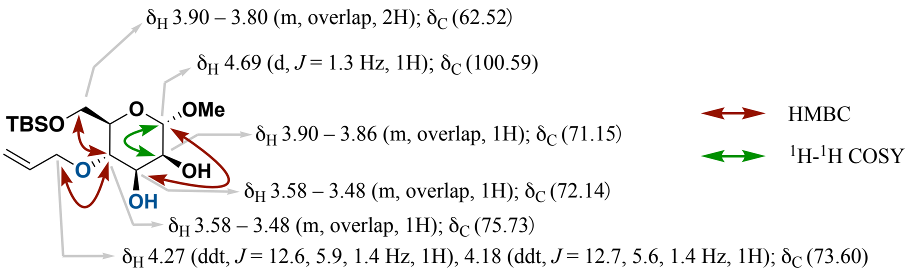

14c

Compound **14** (77.2 mg, 0.25 mmol, 1.0 equiv), 4-(dimethylamino)phenylboronic acid (41.2 mg, TCI Lot OZH3E-IR, 0.25 mmol, 1.0 equiv), and anhydrous THF (1.0 mL) were combined in a screw capped vial (labeled as **Vial A**) containing a stir bar. The mixture was stirred for 30 min and then concentrated under reduced pressure. The resulting material was dried by azeotropic removal of water with small amounts of toluene (1.0 mL × 3) before transferred to a N_2-filled glovebox.

To another vial, compound **2** (63.3 mg, 0.40 mmol,1.6 equiv), $Pd_2(dba)_3$•$CHCl_3$ (13.1 mg, 12.5 μmol, 0.05 equiv), PPh_3 (13.1 mg, 0.05 mmol, 0.2 equiv), and THF (200 μL) were added in sequence under N_2 atmosphere. The mixture was stirred for 10 min, and then transferred to **Vial A**. An additional portion of THF (2.3 mL) was added to Vial A so that [**14**] was adjusted to 0.1 M. **Vial A** was tightly capped, taken out of the glove box, and stirred at 25 ˚C (plate temperature) for 12 h (400 rpm). After that, a solution of glycerol (1 M, aq., 0.5 mL) and Na_2CO_3 (1 M, aq., 0.5 mL) was added and vigorously stirred for additional 30 min, the organic layer was extracted with EtOAc and concentrated. The residue was passed through a pad of silica gel and then concentrated. An aliquot of the residue was taken for ^{1}H NMR analysis, which indicated that products **14a** and **14c** were formed in a ratio of 1:7. Flash chromatography (SiO_2, ca. 3 g) using petroleum ether/EtOAc (3:1 to 1:1) as eluent afforded diallylated product (6.0 mg, 6%), **14c** as a pale yellow oil (44.4 mg, 0.127 mmol, 51%), and starting material **14** (33.4 mg, 43%).

^{1}H NMR ($CDCl_3$, 400 MHz) δ: 5.94 (ddt, *J* = 17.3, 10.3, 5.7 Hz, 1H), 5.28 (dq, *J* = 17.2, 1.7 Hz, 1H), 5.18 (dq, *J* = 10.4, 1.4 Hz, 1H), 4.69 (d, *J* = 1.3 Hz, 1H), 4.27 (ddt, *J* = 12.6, 5.9, 1.4 Hz, 1H), 4.18 (ddt, *J* = 12.7, 5.6, 1.4 Hz, 1H), 3.90 – 3.83 (m, 4H), 3.58 – 3.48 (m, 2H), 3.35 (s, 3H), 2.58 (s, 1H), 2.35 (s, 1H), 0.91 (s, 9H), and 0.09 (d, *J* = 3.2 Hz, 6H).**^{13}C NMR ($CDCl_3$, 101 MHz)** δ: 135.1, 117.3, 100.6, 75.8, 73.6, 72.1, 71.8, 71.2, 62.5, 54.9, 26.1, 26.0, 18.5, -5.0

and -5.2. **IR (thin film, cm^{-1})**: 3421, 290, 2857, 1464, 1269, 1136, 1103, 1058, 973, 836, and 760. **HRMS (DART-TOF)** calculated for $C_{16}H_{32}NaO_6Si^+$[M+Na]$^+$ m/z 371.1860, found 371.1863. **[α]$_D$^{27}** = 69.0 (c = 0.1, $CHCl_3$).

[When no Lewis acid or base was used, **14a**, **14b** and **14c** were obtained at a ratio of 2-*O*:3-*O*:4-*O* = 2.0:1.0:0.4]

Procedures and Characterization Data for Compounds in Figure 4

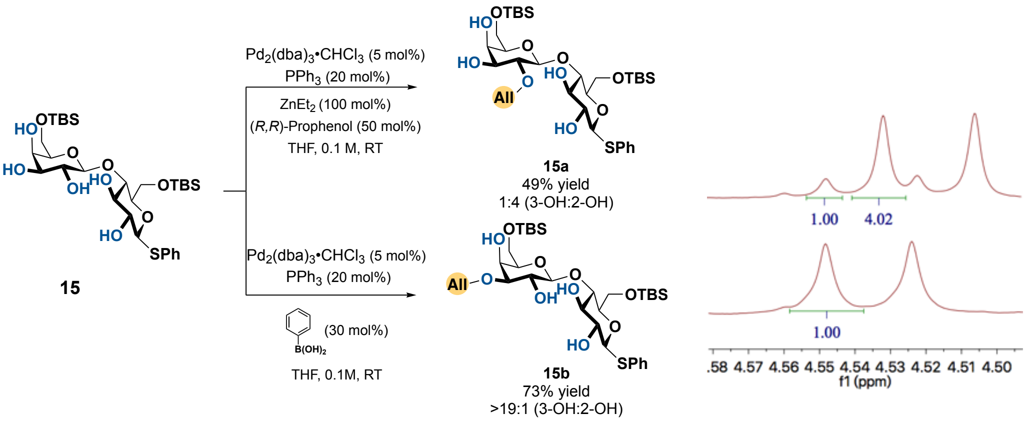

The full NMR spectra of the reaction mixtures were shown below. For the top spectra , NMR was taken after the reaction mixture was passed through a pad of silica gel to remove additives and unreacted starting materials.

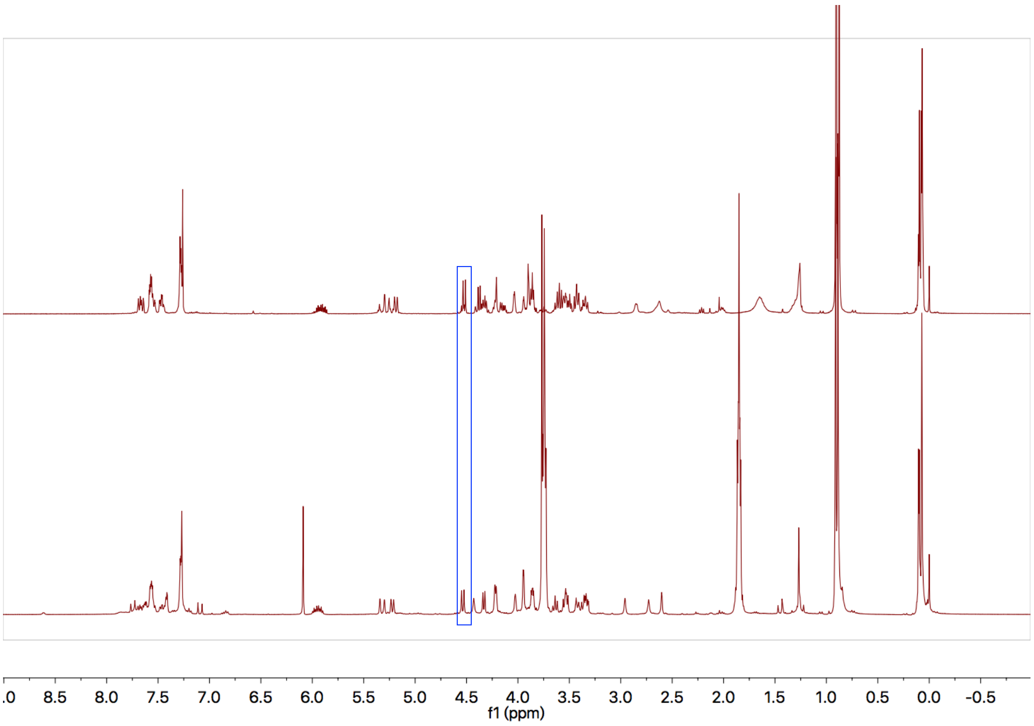

Phenyl 2'-*O*-(allyl)- 6,6'-di (*tert*-Butyldimethylsilyloxyl)-1-thio-β-lactoside (15a)

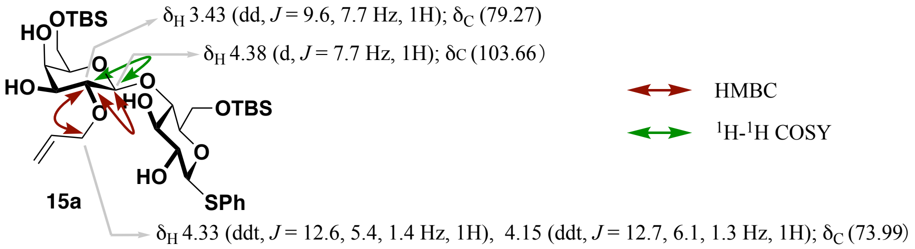

In a N_2-filled glovebox, $ZnEt_2$ (1 M in Toluene, Energy Chemicals Lot FK16010, 100 μL, 0.10 mmol, 1.0 equiv), (*R,R*)-Prophenol (32.0 mg, Sigma Aldrich Lot MKBB9236V, 0.05 mmol, 0.5 equiv) and THF (200 μL) were weighed into a screw capped vial (labeled as **Vial A**) containing a stir bar. The mixture was stirred for 5 min, at which time compound **15**[6] (66.3 mg, 0.10 mmol, 1.0 equiv) was added. The resulting solution was stirred for an additional 30 min.

To another vial, compound **2** (24.0 mg, 0.15 mmol, 1.5 equiv), $Pd_2(dba)_3$•$CHCl_3$ (5.2 mg, 0.005 mmol, 0.05 equiv), PPh_3 (5.2 mg, 0.02 mmol, 0.2 equiv), and THF (200 μL) were added in sequence. The mixture was stirred for 10 min, and then transferred to **Vial A**. An additional portion of THF (600 μL) was added to Vial A so that [**15**] was adjusted to 0.1 M. **Vial A** was tightly capped, taken out of the glove box, and stirred at 25 °C (plate temperature) for 12 h (400 rpm). The reaction mixture was then concentrated in vacuo. The residue was passed through a pad of silica gel and then concentrated. An aliquot of the residue was taken for ^{1}H NMR analysis, which indicated that products **15a** and **15b** were formed in a ratio of 4:1. Flash chromatography (SiO_2) using petroleum ether/EtOAc (3:1 to 1:1 to 1:4) as eluent afforded **15a** as a yellow oil (34.4 mg, 0.049 mmol, 49%) and starting material **15** (18.0 mg, 27%).

^{1}H NMR ($CDCl_3$, 400 MHz) δ: 7.63 – 7.53 (m, 2H), 7.30 – 7.26 (m, 3H), 5.91 (ddt, *J* = 17.2, 10.8, 5.7 Hz, 1H), 5.28 (dq, *J* = 17.2, 1.6 Hz, 1H), 5.19 (dq, *J* = 10.4, 1.3 Hz, 1H), 4.52 (d, *J* = 9.7 Hz, 1H), 4.38 (d, *J* = 7.7 Hz, 1H), 4.33 (ddt, *J* = 12.6, 5.4, 1.4 Hz, 1H), 4.25 – 4.19 (m, 1H), 4.15 (ddt, *J* = 12.7, 6.1, 1.3 Hz, 1H), 4.04 (d, *J* = 3.2 Hz, 1H), 3.91 – 3.84 (m, 3H), 3.65 – 3.57 (m, 2H), 3.57 – 3.52 (m, 1H), 3.49 (td, *J* = 5.9, 1.1 Hz, 1H), 3.46 – 3.39 (m, 1H), 3.43 (dd, *J* = 9.6, 7.7 Hz, 1H), 3.34 (dd, *J* = 9.5, 8.2 Hz, 1H), 2.88 (s, 1H), 2.67 (s, 2H), 0.97 – 0.90 (s, 9H), 0.88 (s, 9H), 0.09 (d, *J* = 7.0 Hz, 1H), and 0.07 (d, *J* = 1.1 Hz, 1H). **^{13}C NMR ($CDCl_3$, 101 MHz)** δ: 134.6, 133.4, 131.8, 129.0, 128.1, 117.7, 103.7, 87.1, 79.5, 79.3, 78.8, 76.1, 74.8, 74.0, 73.5, 71.9, 68.6, 62.3, 61.8, 26.0, 25.9, 18.4, 18.3, -5.0, -5.1, and -5.4. **IR (thin film, cm^{-1})**: 3430, 2928, 2856, 1471, 1462, 1164, 1275, 1259, 1081, 836, 764, and 749. **HRMS (DART-TOF)** calculated for $C_{33}H_{58}NaO_{10}SSi_2^+$ $[M+Na]^+$ m/z 725.3181, found 725.3190. **$[\alpha]_D^{23}$** = –8.1 (c = 0.36, $CHCl_3$).

Phenyl 3'-*O*-(allyl)- 6,6'-di (*tert*-Butyldimethylsilyloxyl)-1-β-lactoside (15b)

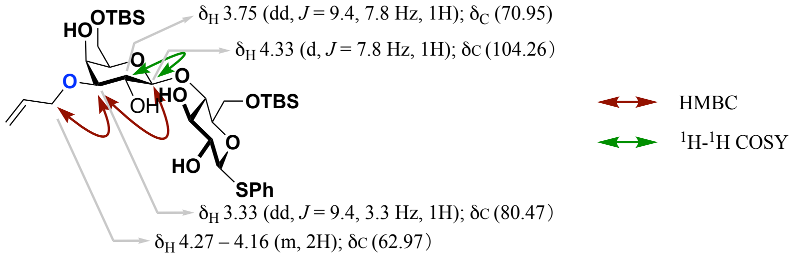

15b

In a N_2-filled glovebox, compound **15** (66.3 mg, 0.10 mmol, 1.0 equiv) and phenylboronic acid (3.7 mg, Macklin Lot C10468538, 0.03 mmol, 0.3 equiv), and THF (500 μL) were weighed into a screw capped vial (labeled as **Vial A**) containing a stir bar. The resulting solution was stirred for an additional 30 min.

To another vial, compound **2** (24.0 mg, 0.15 mmol, 1.5 equiv), $Pd_2(dba)_3$•$CHCl_3$ (5.2 mg, 0.005 mmol, 0.05 equiv), PPh_3 (5.2 mg, 0.02 mmol, 0.2 equiv), and THF (200 μL) were added in sequence. The mixture was stirred for 10 min, and then transferred to **Vial A**. An additional portion of THF (300 μL) was added to Vial A so that [**15**] was adjusted to 0.1 M. **Vial A** was tightly capped, taken out of the glove box, and stirred at 25 °C for 12 h (400 rpm). The reaction mixture was then concentrated in vacuo. An aliquot of the residue was taken for ^{1}H NMR analysis, which indicated that products **15a** and **15b** were formed in a ratio of <1:19. Flash chromatography (SiO_2) using petroleum ether/EtOAc (3:1 to 1:1) as eluent afforded **15b** as a colorless oil (51.0 mg, 0.073 mmol, 73%) and starting material **15** (14.6 mg, 22%).**^{1}H NMR ($CDCl_3$, 400 MHz)** δ: 7.60 – 7.53 (m, 2H), 7.30 – 7.26 (m, 3H), 5.95 (ddt, *J* = 17.3, 10.3, 5.8 Hz, 1H), 5.32 (dq, *J* = 17.2, 1.6 Hz, 1H), 5.23 (dq, *J* = 10.4, 1.3 Hz, 1H), 4.54 (d, *J* = 9.7 Hz, 1H), 4.42 (br s, 1H), 4.33 (d, *J* = 7.8 Hz, 1H), 4.27 – 4.16 (m, 2H), 4.03 (d, *J* = 2.8 Hz, 1H), 3.95 (m, 2H), 3.91 – 3.81 (m, 2H), 3.75 (dd, *J* = 9.4, 7.8 Hz, 1H), 3.64 (t, *J* = 8.7 Hz, 1H), 3.54 (t, *J* = 9.2 Hz, 1H), 3.53 (dd, *J* = 7.2, 6.4 Hz, 1H), 3.42 (dt, *J* = 9.5, 3.0 Hz, 1H), 3.36 (dd, *J* = 9.6, 8.8 Hz, 1H), 3.33 (dd, *J* = 9.4, 3.3 Hz, 1H), 2.90 (s, 1H), 2.67 (s, 1H), 2.57 (s, 1H), 0.91 (s, 9H), 0.89 (s, 9H), 0.10 (d, *J* = 3.1 Hz, 6H), and 0.07 (s, 6H). **^{13}C NMR ($CDCl_3$, 101 MHz)** δ: 134.5, 133.3, 132.0, 129.0, 128.1, 118.1, 104.3, 87.4, 81.1, 80.5, 79.1, 76.5, 75.3, 71.8, 71.3, 71.0, 66.0, 63.0, 62.0, 26.1, 26.0, 18.5, 18.4, -5.0, -5.1, -5.3, and -5.4. **IR (thin film, cm^{-1})**: 3439, 2928, 2856, 1471, 1465, 1275, 1258, 1079, 835, 764, and 749. **HRMS (DART-TOF)** calculated for $C_{33}H_{58}NaO_{10}SSi_2^+$ [M+Na]$^+$ m/z 725.3181, found 725.3180. **$[\alpha]_D^{23}$** = – 14.8 (c = 0.54, $CHCl_3$).

[A complex mixture was formed when no additive is used]

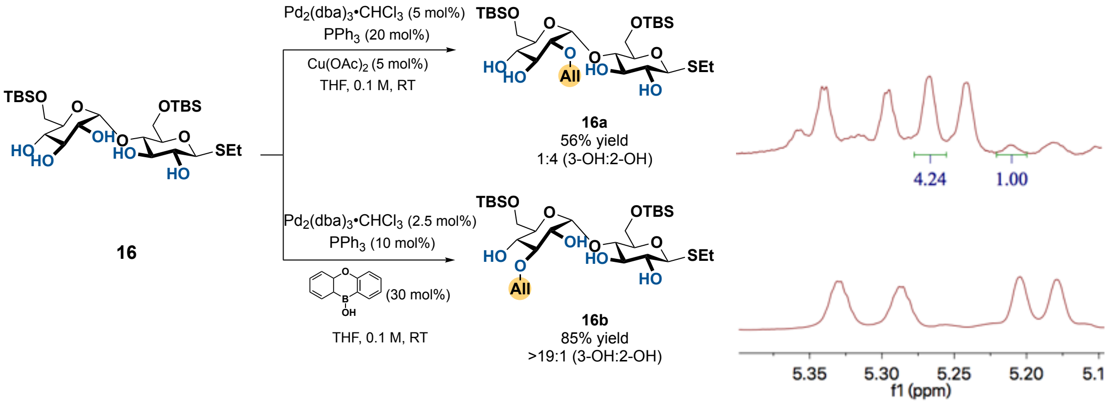

The full NMR spectra of the reaction mixtures were shown below. For the top spectra , NMR was taken after the reaction mixture was passed through a pad of silica gel to remove additives and unreacted starting materials.

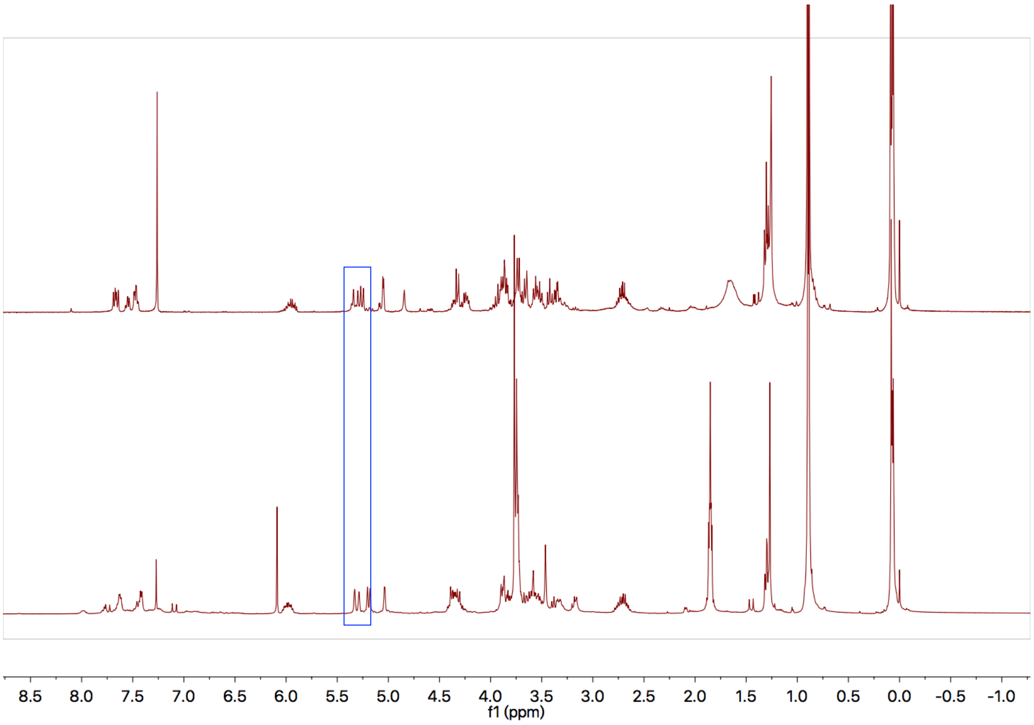

Ethyl-2'-*O*-(allyl)-6,6'-di (*tert*-Butyldimethylsilyloxyl)-1-thiomaltose (16a)

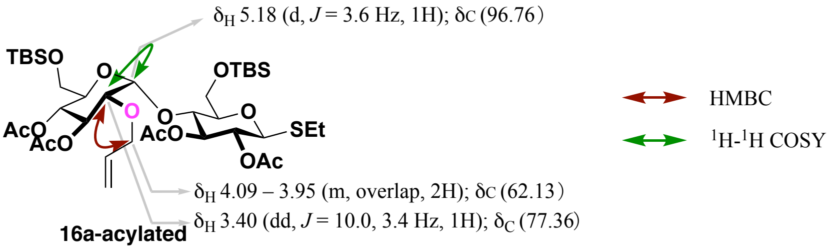

In a N_2-filled glovebox, compound **16**[7] (61.5 mg, 0.10 mmol, 1.0 equiv), $Cu(OAc)_2$ (1.0 mg, Energy Chemicals Lot FI080255, 5 μmol, 0.05 equiv), and THF (500 μL) were weighed into a screw capped vial (labeled as **Vial A**) containing a stir bar. The resulting solution was stirred for an additional 30 min.

To another vial, compound **2** (23.7 mg, 0.15 mmol, 1.5 equiv), $Pd_2(dba)_3$•$CHCl_3$ (5.2 mg, 5 μmol, 0.05 equiv), PPh_3 (5.2 mg, 0.02 mmol, 0.2 equiv), and THF (200 μL) were added in sequence. The mixture was stirred for 10 min, and then transferred to **Vial A**. An additional portion of THF (300 μL) was added to Vial A so that [**16**] was adjusted to 0.1 M. **Vial A** was tightly capped, taken out of the glove box, and stirred at 25 °C for 12 h (400 rpm). The reaction mixture was then concentrated in vacuo. The residue was passed through a pad of silica gel and then concentrated. An aliquot of the residue was taken for ^{1}H NMR analysis, which indicated that products **16a** and **16b** were formed in a ratio of 4:1. Flash chromatography (SiO_2) using petroleum ether/AcOEt (2:1 to 1:1) as eluent afforded **16a** as a light yellow foam (37 mg, 0.056 mmol, 56%) and starting material **16** (9.7 mg, 16%). Product **16a** was further treated with DMAP and Ac_2O to afford the per-acylated product for characterization.

^{1}H NMR ($CDCl_3$, 400 MHz) δ: 5.82 (ddt, *J* = 17.2, 10.3, 5.8 Hz, 1H), 5.30 (t, *J* = 9.2 Hz, 1H), 5.24 (ddt, *J* = 17.6, 1.6, 1.6 Hz, 1H), 5.24 (t, *J* = 9.6 Hz, 1H), 5.18 (ddt, *J* = 10.4, 1.2, 1.2 Hz, 1H), 5.18 (d, *J* = 3.6 Hz, 1H), 4.98 (t, *J* = 9.6 Hz, 1H), 4.92 (t, *J* = 9.6 Hz, 1H), 4.44 (d, *J* = 9.9 Hz, 1H), 4.11 (dd, *J* = 11.9, 2.9 Hz, 1H), 4.09 – 3.95 (m, 3H), 3.94 – 3.86 (m, 2H), 3.67 – 3.57 (m, 2H), 3.40 (dd, *J* = 10.0, 3.4 Hz, 1H), 3.39 – 3.34 (m, 1H), 2.77 – 2.59 (m, 2H), 2.05 (s, 3H), 2.01 (s, 3H), 2.01 (s, 3H), 1.99 (s, 3H), 1.25 (t, *J* = 7.4 Hz, 1H), 0.90 (s, 9H), 0.88 (s, 9H), 0.09 (d, *J* = 5.4 Hz, 6H), and 0.03 (d, *J* = 3.7 Hz, 6H). **^{13}C NMR ($CDCl_3$, 101 MHz)** δ: 170.3, 170.1, 170.0, 169.8, 134.5, 117.9, 96.8, 82.7, 80.0, 77.4, 75.2, 73.8, 72.4, 72.2, 70.9, 70.7, 68.8, 62.2, 62.1, 26.1, 26.0, 23.5, 21.1, 21.0, 21.0, 20.9, 18.5, 18.5, 15.0, -4.9, -5.2, -5.3, and -5.3. **IR (thin film, cm^{-1})**: 2930, 2857, 1753, 1698, 1650, 1401, 1370, 1238, 1149, 1042, 911, 834, and 730. **HRMS (DART-TOF)** calculated for $C_{37}H_{66}NaO_{14}SSi_2^+$ [M+Na]$^+$ m/z 845.3604, found 845.3608. **$[\alpha]_D^{26}$** = 19.7 (c = 0.44, $CHCl_3$).

Ethyl-3'-*O*-(allyl)-6,6'-di (*tert*-Butyldimethylsilyloxyl)-1-thiomaltose (16b)

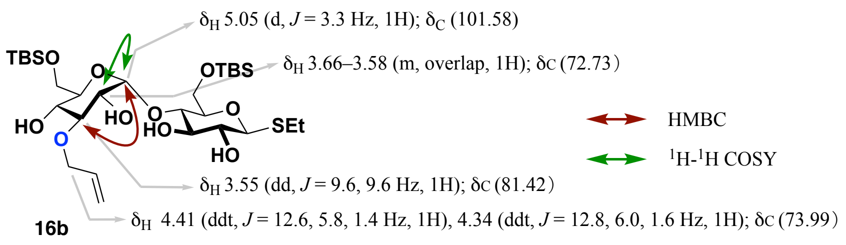

In a N_2-filled glovebox, compound **16** (61.5 mg, 0.10 mmol, 1.0 equiv) and 10*H*-dibenzo[*b*,*e*][1,4]oxaborinin-10-ol[5] (5.9 mg, 0.03 mmol, 0.3 equiv), and THF (500 μL) were weighed into a screw capped vial (labeled as **Vial A**) containing a stir bar. The resulting solution was stirred for an additional 30 min.

To another vial, compound **2** (25.3 mg, 0.16 mmol, 1.6 equiv), $Pd_2(dba)_3$•$CHCl_3$ (2.6 mg, 2.5 μmol, 0.025 equiv), PPh_3 (2.6 mg, 0.02 mmol, 0.2 equiv), and THF (200 μL) were added in sequence. The mixture was stirred for 10 min, and then transferred to **Vial A**. An additional portion of THF (300 μL) was added to Vial A so that [**16**] was adjusted to 0.1 M. **Vial A** was tightly capped, taken out of the glove box, and stirred at 25 ˚C (plate temperature) for 12 h (400 rpm). The reaction mixture was then concentrated in vacuo. An aliquot of the residue was taken for ^{1}H NMR analysis, which indicated that products **16a** and **16b** were formed in a ratio of < 1:19. Flash chromatography (SiO_2) using petroleum ether/EtOAc (2:1 to 1:1) as eluent afforded **16b** as a pale yellow oil (55.4 mg, 0.085 mmol, 85%).

^{1}H NMR ($CDCl_3$, 400 MHz) δ: 5.99 (ddt, *J* = 17.2, 10.3, 5.8 Hz, 1H), 5.32 (dq, *J* = 17.2, 1.7 Hz, 1H), 5.20 (ddt, *J* = 10.4, 2.0, 1.2 Hz, 1H), 5.05 (d, *J* = 3.3 Hz, 1H), 4.98 (s, 1H), 4.41 (ddt, *J* = 12.6, 5.8, 1.4 Hz, 1H), 4.34 (ddt, *J* = 12.8, 6.0, 1.6 Hz, 1H), 4.32 (d, *J* = 9.6 Hz, 1H), 3.89 (dt, *J* = 10.9, 2.8 Hz, 2H), 3.82 (dd, *J* = 11.5, 4.6 Hz, 1H), 3.79 – 3.67 (m, 4H), 3.66–3.51 (m, 3H), 3.55 (t, *J* = 9.6 Hz, 1H), 3.40 (t, *J* = 9.5 Hz, 1H), 3.35 (ddd, *J* = 9.6, 4.5, 1.8 Hz, 1H), 3.15 (d, *J* = 1.8 Hz, 1H), 2.92 (s, 1H), 2.79 – 2.62 (m, 2H), 1.31 (t, *J* = 7.4 Hz, 3H), 0.90 (s, 9H), 0.89 (s, 9H), 0.08 (s, 6H), and 0.06 (d, *J* = 2.9 Hz, 6H). **^{13}C NMR ($CDCl_3$, 101 MHz)** δ: 135.3, 117.4, 101.6, 85.2, 81.4, 80.8, 79.8, 77.8, 74.0, 72.9, 72.7, 72.3, 71.4, 64.6, 62.6, 26.0, 26.0, 23.9, 18.5, 18.4, 15.5, -5.0, -5.1, -5.3 and -5.4. **IR (thin film, cm^{-1})**: 3362, 2928, 2856, 1462, 1360, 1252, 1140, 1074, 1035, 835, and 776. **HRMS (DART-TOF)** calculated for $C_{29}H_{58}NaO_{10}SSi_2^+$ $[M+Na]^+$ m/z 677.3181, found 677.3184. **$[\alpha]_D^{27}$** = 33.3 (c = 0.65, $CHCl_3$). [A complex mixture is formed when no additive is used.]

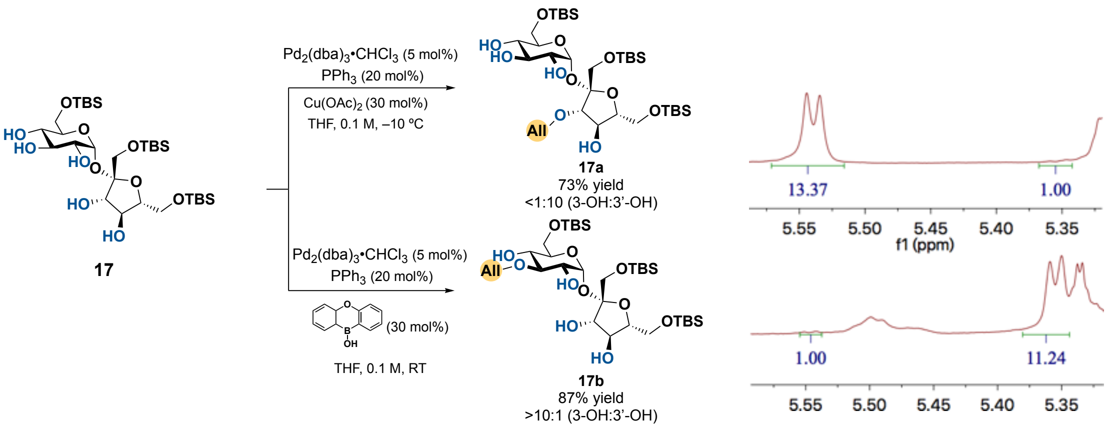

The full NMR spectra of the reaction mixtures were shown below. For the top spectra , NMR was taken after the reaction mixture was passed through a pad of silica gel to remove additives and unreacted starting materials.

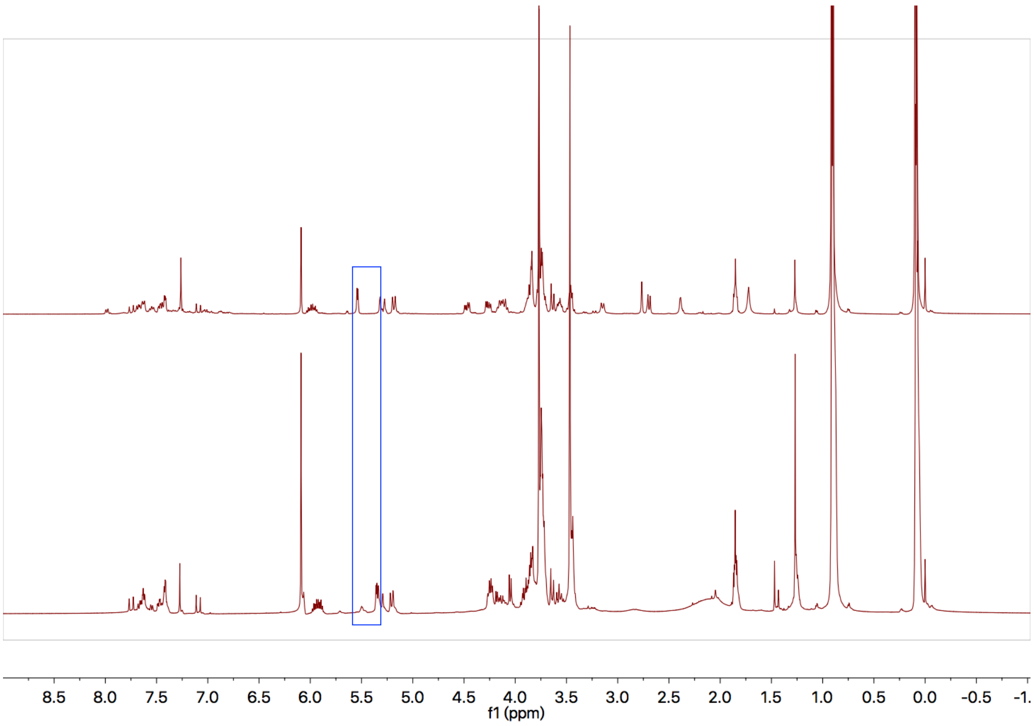

1’, 6’,6-tri (*tert*-Butyldimethylsilyloxyl)-3’-*O*-(allyl)-sucrose (17a)

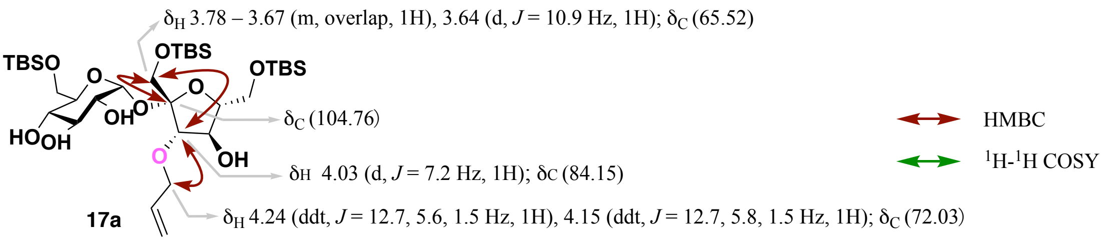

In a N_2-filled glovebox, compound **17** (68.5 mg, 0.10 mmol, 1.0 equiv), $Cu(OAc)_2$ (5.6 mg, Energy Chemicals Lot FI080255, 0.03 mmol, 0.3 equiv), and THF (500 μL) were weighed into a screw capped vial (labeled as **Vial A**) containing a stir bar. The resulting solution was stirred for an additional 30 min.

To another vial, compound **2** (24.0 mg, 0.15 mmol, 1.5 equiv), $Pd_2(dba)_3$•$CHCl_3$ (5.2 mg, 5 μmol, 0.05 equiv), PPh_3 (5.2 mg, 0.02 mmol, 0.2 equiv), and THF (200 μL) were added in sequence. The mixture was stirred for 10 min, and then transferred to **Vial A**. An additional portion of THF (300 μL) was added to Vial A so that [**17**] was adjusted to 0.1 M. **Vial A** was tightly capped, taken out of the glove box, and stirred at 25 ˚C (plate temperature) for 12 h (400 rpm). The reaction mixture was then concentrated in vacuo. The residue was passed through a short pad of silica gel and concentrated. An aliquot of the residue was taken for ^{1}H NMR analysis, which indicated that products **17a** and **17b** were formed in a ratio of >10:1. Flash chromatography (SiO_2) using petroleum ether/EtOAc (3:1 to 1:1) as eluent afforded **17a** as a yellow oil (52.9 mg, 0.073 mmol, 73%).

^{1}H NMR ($CDCl_3$, 400 MHz) δ: 5.93 (dddd, *J* = 16.0, 11.0, 7.2, 4.1 Hz, 1H), 5.36 (d, *J* = 3.8 Hz, 1H), 5.31 (dq, *J* = 17.3, 1.6 Hz, 1H), 5.20 (dq, *J* = 10.4, 1.4 Hz, 1H), 4.27 (m, 1H), 4.24 (ddt, *J* = 12.7, 5.6, 1.5 Hz, 1H), 4.15 (ddt, *J* = 12.7, 5.8, 1.5 Hz, 1H), 4.03 (d, *J* = 7.2 Hz, 1H), 3.95 – 3.82 (m, 3H), 3.78 – 3.67 (m, 5H), 3.64 (d, *J* = 10.9 Hz, 1H), 3.58 (t, *J* = 9.2 Hz, 1H), 3.43 (dd, *J* = 9.4, 3.7 Hz, 1H), 0.96 – 0.84 (m, 27H), and 0.09 (dd, *J* = 4.3, 2.1 Hz, 18H). **^{13}C NMR ($CDCl_3$, 101 MHz)** δ: 134.6, 117.6, 104.8, 92.3, 84.2, 80.9, 77.4, 74.8, 72.3, 72.2, 72.0, 70.9, 65.5, 64.4, 64.3, 26.0, 26.0, 18.5, 18.4, -5.2, -5.3, -5.3, -5.3, and -5.3. **IR (thin film, cm^{-1})**: 3402, 2928, 2857, 1463, 1361, 1254, 1117, 1070, 1004, 835, and 777. **HRMS (DART-TOF)** calculated for $C_{33}H_{68}NaO_{11}Si^+$ $[M+Na]^+$ m/z 747.3962, found 747.3966. **$[\alpha]_D^{27}$** = 14.2 (c = 0.64, $CHCl_3$).

1', 6',6-tri (*tert*-Butyldimethylsilyloxyl)-3-*O*-(allyl)-sucrose (17b)

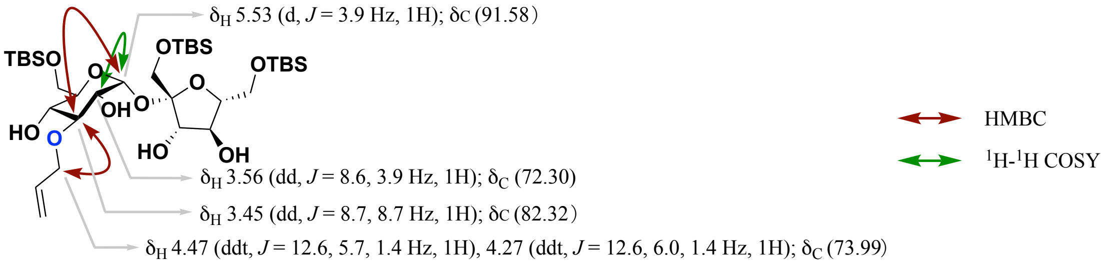

17b

In a N_2-filled glovebox, compound **17** (68.5 mg, 0.10 mmol, 1.0 equiv), 10*H*-dibenzo[*b*,*e*][1,4]oxaborinin-10-ol[5] (6.0 mg, 0.03 mmol, 0.3 equiv)**,** and THF (500 μL) were weighed into a screw capped vial (labeled as **Vial A**) containing a stir bar. The resulting solution was stirred for an additional 30 min.

To another vial, compound **2** (24.0 mg, 0.15 mmol, 1.5 equiv), $Pd_2(dba)_3$•$CHCl_3$ (5.2 mg, 0.005 mmol, 0.05 equiv), PPh_3 (5.2 mg, 0.02 mmol, 0.2 equiv), and THF (200 μL) were added in sequence. The mixture was stirred for 10 min, and then transferred to **Vial A**. An additional portion of THF (300 μL) was added to Vial A so that [**17**] was adjusted to 0.1 M. **Vial A** was tightly capped, taken out of the glove box, and stirred at 25 ˚C for 12 h (400 rpm). The reaction mixture was then concentrated in vacuo. An aliquot of the residue was taken for ^{1}H NMR analysis, which indicated that products **17a** and **17b** were formed in a ratio of <1:10. Flash chromatography (SiO_2) using petroleum ether/EtOAc (3:1 to 2:1) as eluent afforded **17b** as a colorless oil (63.3 mg, 0.087 mmol, 87%).

^{1}H NMR ($CDCl_3$, 400 MHz) δ: 5.98 (ddt, *J* = 16.4, 10.4, 5.8 Hz, 1H), 5.53 (d, *J* = 3.9 Hz, 1H), 5.30 (dq, *J* = 17.2, 1.6 Hz, 1H), 5.18 (dq, *J* = 10.3, 1.4 Hz, 1H), 4.47 (ddt, *J* = 12.6, 5.7, 1.4 Hz, 1H), 4.27 (ddt, *J* = 12.6, 6.0, 1.4 Hz, 1H), 4.18 – 4.05 (m, 2H), 3.91 – 3.81 (m, 4H), 3.80 – 3.69 (m, 3H), 3.64 (d, *J* = 10.8 Hz, 1H), 3.56 (dd, *J* = 8.6, 3.9 Hz, 1H), 3.51 – 3.41 (m, 1H), 3.45 (dd, *J* = 8.7, 8.7 Hz, 1H), 3.17 (dd, *J* = 9.7, 5.0 Hz, 1H), 2.80 (d, *J* = 5.7 Hz, 1H), 2.77 – 2.68 (m, 1H), 2.46 (d, *J* = 13.5 Hz, 1H), 0.91 (s, 9H), 0.91 (s, 9H), 0.90 (s, 9H), 0.10 (s, 6H), 0.10 (s, 3H), 0.09 (s, 3H), and 0.08 (s, 6H). **^{13}C NMR ($CDCl_3$, 101 MHz)** δ: 135.4, 117.3, 104.6, 91.6, 82.3, 81.0, 78.9, 76.3, 74.0, 72.3, 71.1, 65.5, 64.1, 63.4, 26.1, 26.0, 26.0, 18.6, 18.5, 18.4, -5.2, -5.3, -5.3, and -5.3. **IR (thin film, cm^{-1})**: 3411, 2929, 2857, 1672, 1471, 1361, 1256, 1075, 1004, 836, and 750. **HRMS (DART-TOF)** calculated for $C_{33}H_{68}NaO_{11}Si^+$ $[M+Na]^+$ m/z 747.3962, found 747.3967. **$[\alpha]_D^{27}$** = 22.6 (c = 1.30, $CHCl_3$).

[When no additive was used, **17a** and **17b** were obtained with a ratio of 4.0:1.0.]

Procedures and Characterization Data for Compounds in Figure 5

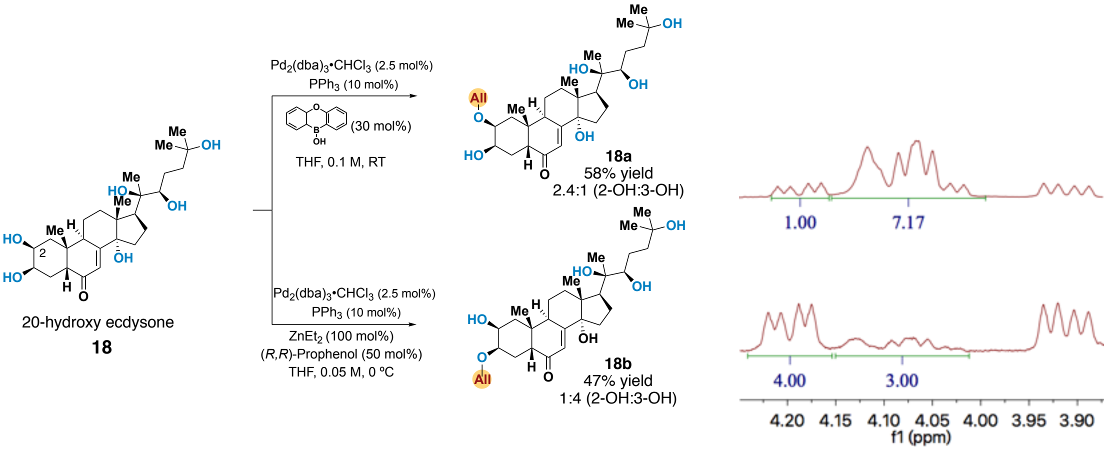

The full NMR spectra of the reaction mixtures were shown below. For the top and bottom spectra, NMR was taken after the reaction mixture was passed through a pad of silica gel to remove additives and unreacted starting materials.

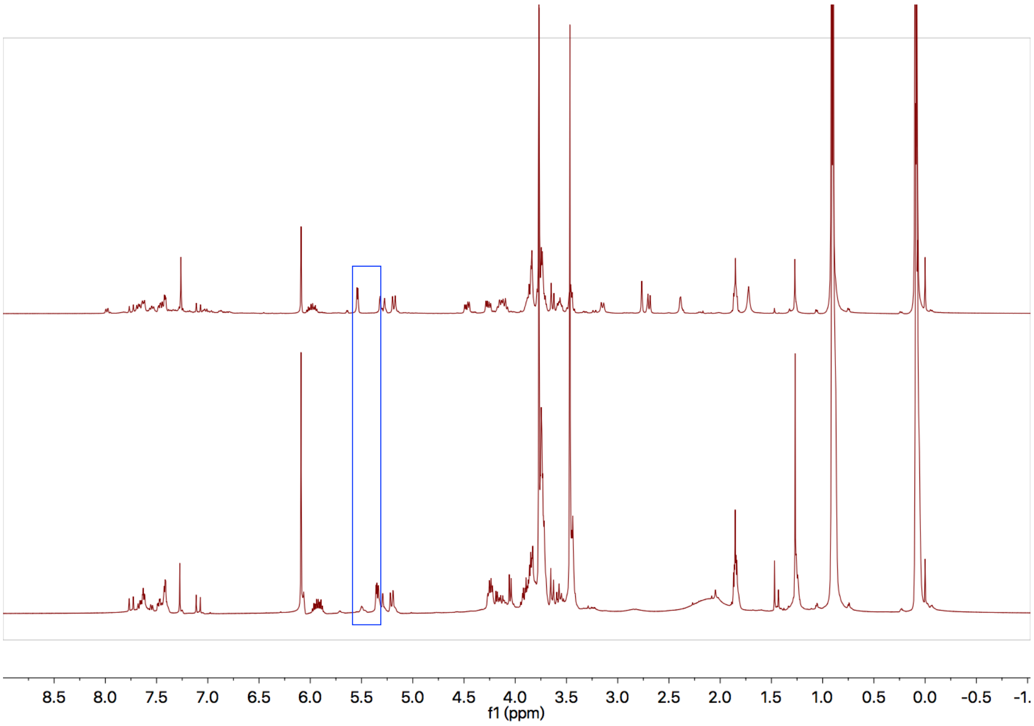

2-*O*-(allyl)-20-hydroxyecdysone (18a)

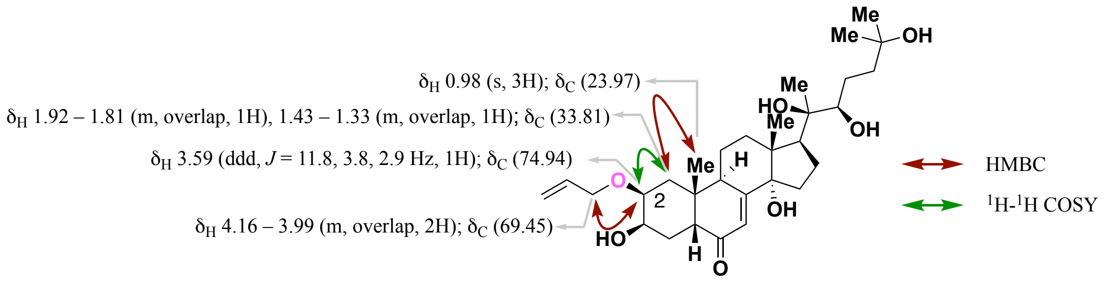

18a

Consistent with this assignment, the chemical shift of C2–H in 20-hydroxyecdysone has moved from δH 3.89 to δH 3.59 after allylation.

In a N_2-filled glovebox, 20-hydroxyecdysone **18** (48.1 mg, SC-Shuyan Lot SY190925, 0.10 mmol, 1.0 equiv), 10*H*-dibenzo[*b*,*e*][1,4]oxaborinin-10-ol[5] (5.9 mg, 0.03 mmol, 0.3 equiv)**,** and THF (500 μL) were weighed into a screw capped vial (labeled as **Vial A**) containing a stir bar. The resulting solution was stirred for an additional 30 min.

To another vial, compound **2** (19.0 mg, 0.12 mmol, 1.2 equiv), $Pd_2(dba)_3$•$CHCl_3$ (2.6 mg, 2.5 μmol, 0.025 equiv), PPh_3 (2.6mg, 0.01 mmol, 0.1 equiv), and THF (200 μL) were added in sequence. The mixture was stirred for 10 min, and then transferred to **Vial A**. An additional portion of THF (300 μL) was added to Vial A so that [**18**] was adjusted to 0.1 M. **Vial A** was tightly capped, taken out of the glove box, and stirred at 25 ˚C (plate temperature) for 12 h (400 rpm). The reaction mixture was then concentrated in vacuo. An aliquot of the residue was taken for ^{1}H NMR analysis, which indicated that products **18a** and **18b** were formed in a ratio of 2.4:1. Flash chromatography (SiO_2) using CH_2Cl_2/MeOH (18:1 to 9:1) as eluent afforded **18a** and **18b** as an inseparable mixture (30.2 mg, 0.058 mmol, **18a**:**18b** = 2.4:1, 58%).

^{1}H NMR ($CDCl_3$, 400 MHz) δ: 5.92 (ddt, *J* = 16.2, 10.7, 5.5 Hz, 1H), 5.84 (d, *J* = 2.5 Hz, 1H), 5.30 (ddd, *J* = 17.2, 3.4, 1.6 Hz, 1H), 5.21 (ddd, *J* = 10.4, 2.9, 1.3 Hz, 1H), 4.16 – 3.99 (m, 3H), 3.59 (ddd, *J* = 11.8, 3.8, 2.9 Hz, 1H), 3.43 (d, *J* = 10.4 Hz, 1H), 2.99 (ddd, *J* = 14.0, 6.4, 2.9 Hz, 1H), 2.60 – 2.44 (m, 3H), 2.37 (t, *J* = 8.8 Hz, 1H), 2.25 – 2.17 (m, 1H), 2.11 (td, *J* = 12.9, 5.1 Hz, 2H), 2.05 – 1.98 (m, 2H), 1.92 – 1.81 (m, 3H), 1.80 – 1.70 (m, 3H), 1.69 – 1.56 (m, 4H), 1.43 – 1.33 (m, 2H), 1.24 (d, *J* = 3.3 Hz, 6H), 1.22 (s, 3H), 0.98 (s, 3H), and 0.87 (s, 3H). **^{13}C NMR ($CDCl_3$, 101 MHz)** δ: 203.8 164.4, 134.8, 134.7, 121.8, 117.3, 84.8, 77.0, 74.9, 70.9, 69.5, 64.9, 50.3, 49.2, 47.4, 41.0, 38.3, 34.0, 33.8, 31.7, 31.3, 30.6, 30.1, 29.3, 26.1, 24.0, 20.9, 20.6, 20.5, and 17.6. **IR (thin film, cm^{-1})**: 3415, 2965, 2877, 1652, 1445, 1380, 1264, 1073, 911, and 800. **HRMS (DART-TOF)** calculated for $C_{30}H_{48}NaO_7^+$[M+Na]$^+$ m/z 543.3292, found 543.3298. **$[\alpha]_D^{23}$** = 54.0 (c = 0.22, $CHCl_3$)

21-*O*-(allyl)-20-hydroxyecdysone (18b)

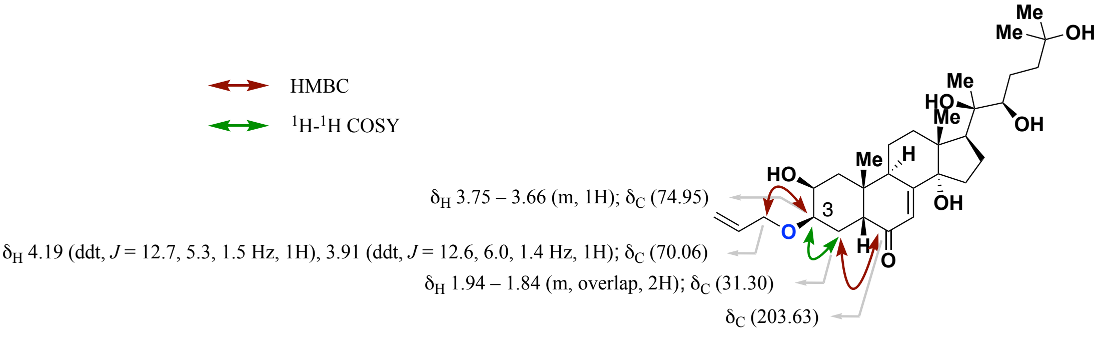

18b

Consistent with this assignment, the chemical shift of C3–H in 20-hydroxyecdysone has moved from δH 4.04 to δH 3.71 after allylation.

In a N_2-filled glovebox, $ZnEt_2$ (1 M in Toluene, Energy Chemicals Lot FK16010, 30 µL, 0.03 mmol, 0.3 equiv), (*R*,*R*)-Prophenol (9.6 mg, Sigma Aldrich Lot MKBB9236V, 0.015 mmol, 0.15 equiv) and THF (200 µL) were weighed into a screw capped vial (labeled as **Vial A**) containing a stir bar. The mixture was stirred for 5 min, at which time compound **21** (48.0 mg, 0.10 mmol, 1.0 equiv) was added. The resulting solution was stirred for an additional 30 min.

To another vial, compound **2** (25.4 mg, 0.16 mmol, 1.6 equiv), $Pd_2(dba)_3$•$CHCl_3$ (2.6 mg, 2.5 µmol, 0.025 equiv), PPh_3 (2.6 mg, 0.01 mmol, 0.1 equiv), and THF (200 µL) were added in sequence. The mixture was stirred for 10 min, and then transferred to **Vial A**. An additional portion of THF (1.6 mL) was added to Vial A so that [**18**] was adjusted to 0.05 M. **Vial A** was tightly capped, taken out of the glove box, and stirred at 0 °C for 12 h (400 rpm). The reaction mixture was then concentrated in vacuo. An aliquot of the residue was taken for ^{1}H NMR analysis, which indicated that products **18a** and **18b** were formed in a ratio of 1:4. Flash chromatography (SiO_2) using CH_2Cl_2/MeOH (27:1 to 18:1 to 9:1) as eluent afforded **18b** as a pale yellow foam (25.0 mg, 0.048 mmol, **18a**:**18b** = 1:4, 47%) and recovered starting material **18 (**15.6 mg, 32%).

^{1}H NMR ($CDCl_3$, 400 MHz) δ: 5.92 (dq, *J* = 16.3, 5.3 Hz, 1H), 5.85 (d, *J* = 2.6 Hz, 1H), 5.33 – 5.17 (m, 2H), 4.19 (ddt, *J* = 12.7, 5.3, 1.5 Hz, 1H), 3.91 (ddt, *J* = 12.6, 6.0, 1.4 Hz, 1H), 3.81 (d, *J* = 11.8 Hz, 1H), 3.75 – 3.66 (m, 1H), 3.43 (d, *J* = 10.3 Hz, 1H), 3.02 (ddd, *J* = 11.2, 7.2, 2.7 Hz, 1H), 2.52 – 2.26 (m, 3H), 2.10 (dd, *J* = 13.2, 4.9 Hz, 1H), 2.07 – 1.98 (m, 3H), 2.07 – 1.98 (m, 3H), 1.98 – 1.82 (m, 5H), 1.80 – 1.69 (m, 3H), 1.68– 1.46 (m, 5H), 1.42 – 1.31 (m, 3H), 1.25 (s, 6H), 1.22 (s, 3H), 0.98 (s, 3H), and 0.87 (s, 3H). **^{13}C NMR ($CDCl_3$, 101 MHz)** δ: 203.6, 164.5, 134.7, 121.8, 117.4, 84.9, 77.4, 77.1, 75.0, 71.0, 70.1, 67.6, 50.6, 49.2, 47.6, 41.0,

38.5, 38.2, 33.9, 31.8, 31.3, 30.1, 29.5, 28.0, 27.4, 26.1, 24.1, 20.9, 20.6, and 17.6. **IR (thin film, cm^{-1})**: 3394, 2964, 2932, 1653, 1446, 1381, 1275, 1260, 1071, 913, 764, and 749. **HRMS (DART-TOF)** calculated for $C_{30}H_{48}NaO_7^+$[M+Na]$^+$ m/z 543.3292, found 543.3300. **$[\alpha]_D^{27}$** = 34.8 (c = 0.37, $CHCl_3$).

[When no additive is used, **18a** and **18b** were obtained at a ratio of ca. 1.0:1.0.]

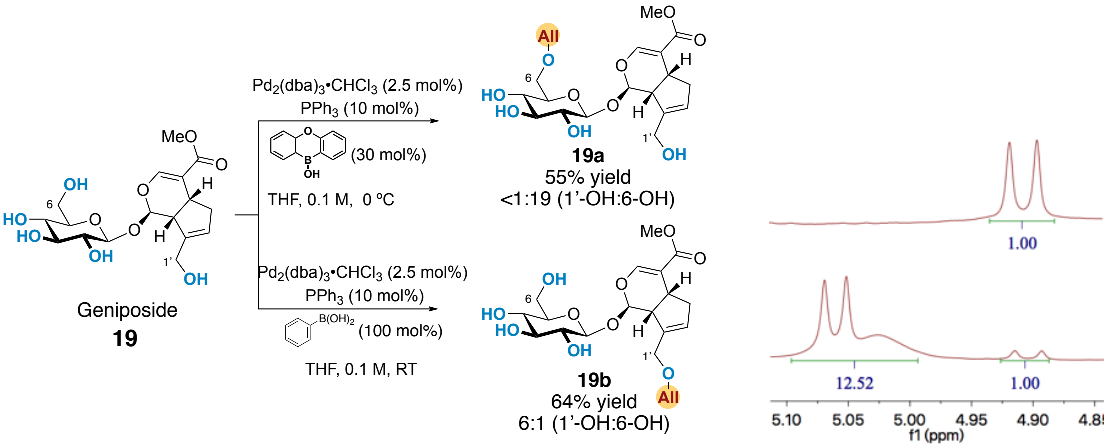

The full NMR spectra of the reaction mixtures were shown below. For the top and bottom spectra , NMR was taken after the reaction mixture was passed through a pad of silica gel to remove additives and unreacted starting materials.

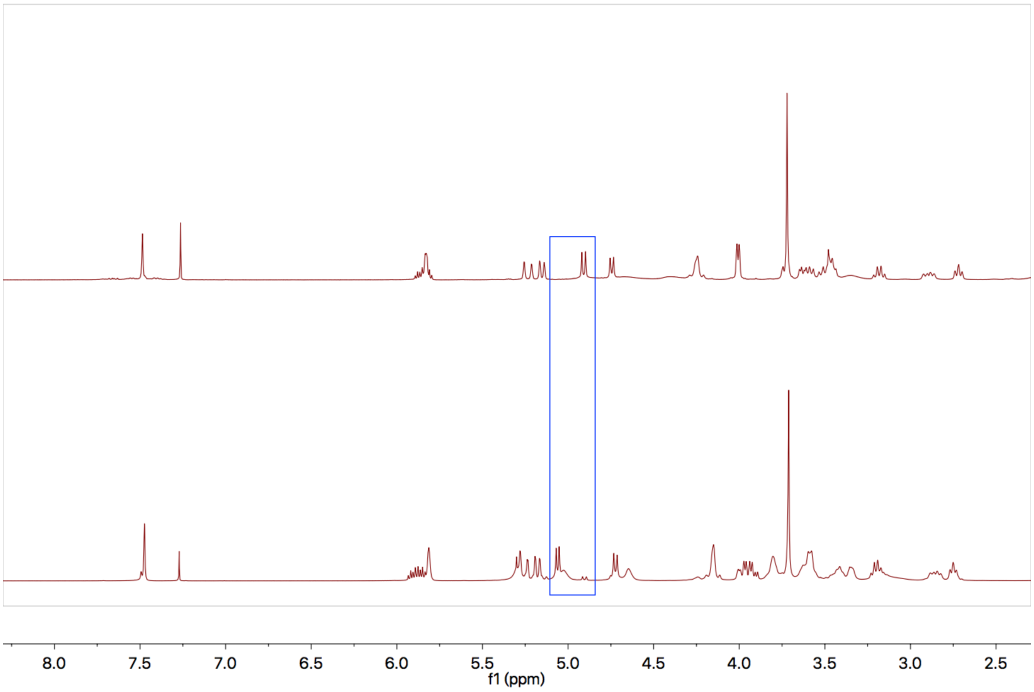

6’-*O*-(allyl)-geniposide (19a)

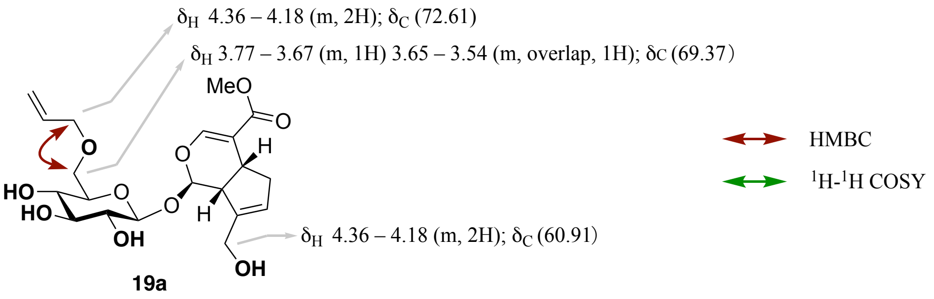

In a N_2-filled glovebox, geniposide **19** (77.6 mg, SC-Shuyan Lot SY190328, 0.20 mmol, 1.0 equiv), 10*H*-dibenzo[*b*,*e*][1,4]oxaborinin-10-ol[5] (11.8 mg, 0.06 mmol, 0.3 equiv), and THF (500 μL) were weighed into a screw capped vial (labeled as **Vial A**) containing a stir bar. The resulting solution was stirred for an additional 30 min.

To another vial, compound **2** (50.6 mg, 0.32 mmol, 1.6 equiv), $Pd_2(dba)_3$•$CHCl_3$ (5.2 mg, 0.005 mmol, 0.025 equiv), PPh_3 (5.2 mg, 0.02 mmol, 0.1 equiv), and THF (200 μL) were added in sequence. The mixture was stirred for 10 min, and then transferred to **Vial A**. An additional portion of THF (1.3 mL) was added to Vial A so that [**19**] was adjusted to 0.1 M. **Vial A** was tightly capped, taken out of the glove box, and stirred at 0 ˚C for 16 h (400 rpm). The reaction mixture was then concentrated in vacuo. An aliquot of the residue was taken for ^{1}H NMR analysis, which indicated that products **19a** and **19b** were formed in a ratio of >19:1. Flash chromatography (SiO_2) using CH_2Cl_2/MeOH (27:1 to 18:1 to 9:1) as eluent afforded diallylated products (5.6 mg, 6%), **19a** as a light yellow oil (46.7 mg, 0.110 mmol, 55%), and starting material **19** (25.1 mg, 32%).

^{1}H NMR ($CDCl_3$, 400 MHz) δ: 7.49 (d, *J* = 1.2 Hz, 1H), 5.84 – 5.79 (br s, 1H), 5.83 (dt, *J* = 17.2, 5.3 Hz, 1H), 5.22 (dq, *J* = 17.2, 1.7 Hz, 1H), 5.14 (dd, *J* = 10.4, 1.6 Hz, 1H), 4.90 (d, *J* = 8.7 Hz, 1H), 4.75 (d, *J* = 7.7 Hz, 1H), 4.36 – 4.18 (m, 2H), 4.00 (m, 2H), 3.77 – 3.67 (m, 1H), 3.72 (s, 3H), 3.65 – 3.54 (m, 2H), 3.53 – 3.42 (m, 3H), 3.17 (q, *J* = 8.6 Hz, 1H), 2.89 (dd, *J* = 15.6, 8.6 Hz, 1H), 2.72 (t, *J* = 8.5 Hz, 1H), and 2.03 (dd, *J* = 16.3, 9.4 Hz, 1H). **^{13}C NMR ($CDCl_3$, 101 MHz)** δ: 167.8, 152.0, 143.2, 134.6, 129.7, 117.3, 111.6, 99.2, 98.3, 76.3, 75.6, 73.3, 72.6, 70.5, 69.4, 60.9, 51.5, 45.3, 39.1, and 36.0. **IR (thin film, cm^{-1})**: 3382, 3005, 2910, 2855, 1703, 1631, 1438, 1277, 1073, 1043, and 749. **HRMS (DART-TOF)** calculated for $C_{20}H_{28}NaO_{10}^+$[M+Na]$^+$ m/z 451.1575, found 451.1578. **$[\alpha]_D^{26}$** = –2.1 (c = 0.91, $CHCl_3$).

10-*O*-(allyl)-geniposide (19b)

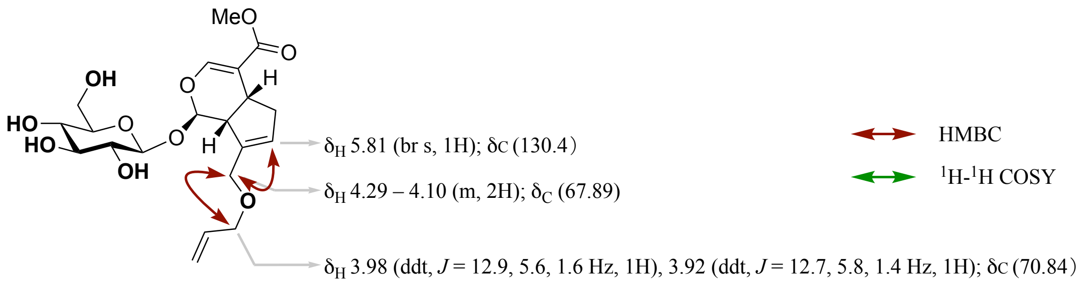

In a N_2-filled glovebox, geniposide **19** (77.6 mg, 0.20 mmol, 1.0 equiv), Phenylboronic acid (24.2 mg, 0.20 mmol, 1.0 equiv), and THF (500 μL) were weighed into a screw capped

vial (labeled as **Vial A**) containing a stir bar. The resulting solution was stirred for an additional 30 min.

To another vial, compound **2** (50.6 mg, 0.32 mmol, 1.6 equiv), $Pd_2(dba)_3 \cdot CHCl_3$ (5.2 mg, 5 μmol, 2.5 mol%), PPh_3 (5.2 mg, 0.02 mmol, 0.1 equiv), and THF (200 μL) were added in sequence. The mixture was stirred for 10 min, and then transferred to **Vial A**. An additional portion of THF (1.3 mL) was added to Vial A so that [**19**] was adjusted to 0.1 M. **Vial A** was tightly capped, taken out of the glove box, and stirred at 25 ˚C (plate temperature) for 12 h (400 rpm). The reaction mixture was then concentrated in vacuo. An aliquot of the residue was taken for ^1H NMR analysis, which indicated that products **19a** and **19b** were formed in a ratio of 1:6. Flash chromatography (SiO_2) using CH_2Cl_2/MeOH (27:1 to 9:1) as eluent afforded diallylated products (11.9 mg, ca. 10%), **19b** as a light yellow oil (54.6 mg, 0.128 mmol, **19a**:**19b** = 1:6, 63.8%), and recovered starting material **19** (15.7 mg, 20%).

^{1}H NMR ($CDCl_3$, 400 MHz) δ: 7.47 (d, *J* = 1.1 Hz, 1H), 5.88 (ddt, *J* = 17.3, 10.4, 5.7 Hz,1H), 5.81 (br s, 1H), 5.26 (dq, *J* = 17.2, 1.7 Hz, 1H), 5.18 (dd, *J* = 10.4, 1.7 Hz, 1H), 5.11 – 4.92 (m, 1H), 5.06 (d, *J* = 7.1 Hz, 1H), 4.83 – 4.65 (m, 1H), 4.72 (d, *J* = 7.8 Hz, 1H), 4.29 – 4.10 (m, 2H), 3.98 (ddt, *J* = 12.9, 5.6, 1.6 Hz, 1H), 3.92 (ddt, *J* = 12.7, 5.8, 1.4 Hz, 1H), 3.86 – 3.74 (m, 2H), 3.71 (s, 3H), 3.67 – 3.53 (m, 2H), 3.50 – 3.38 (m, 2H), 3.38 – 3.29 (m, 1H), 3.21 (q, *J* = 7.8 Hz, 1H), 2.85 (dd, *J* = 16.4, 8.6 Hz, 1H), 2.75 (t, *J* = 7.6 Hz, 1H), and 2.12 (dd, *J* = 16.4, 7.6 Hz, 1H). **^{13}C NMR ($CDCl_3$, 101 MHz)** δ: 167.9, 151.9, 139.9, 134.6, 130.4, 117.6, 111.7, 99.4, 97.4, 76.2, 76.1, 73.3, 70.8, 69.8, 67.9, 61.7, 51.5, 45.9, 38.8, and 34.9. **IR (thin film, cm^{-1})**: 3382, 3005, 2910, 2855, 1703, 1631, 1438, 1277, 1073, 1043, and 749. **HRMS (DART-TOF)** calculated for $C_{20}H_{28}NaO_{10}^+$ $[M+Na]^+$ m/z 451.1575, found 451.1578. **$[\alpha]_D^{26}$** = –2.1 (c = 0.91, $CHCl_3$).

[A complex mixture was formed when no additive is used.]

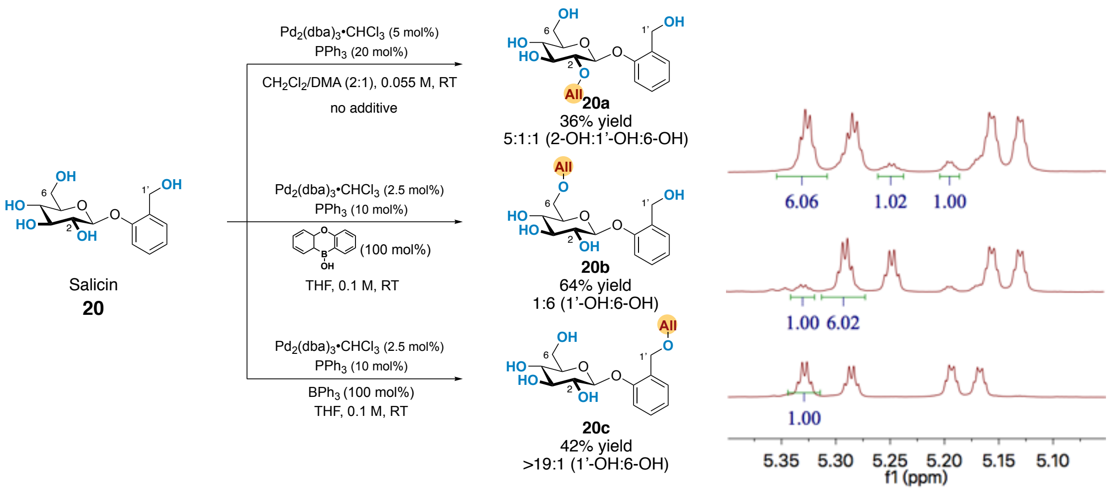

The full NMR spectra of the reaction mixtures were shown below. For all of the spectra , NMR was taken after the reaction mixture was passed through a pad of silica gel to remove additives and unreacted starting materials.

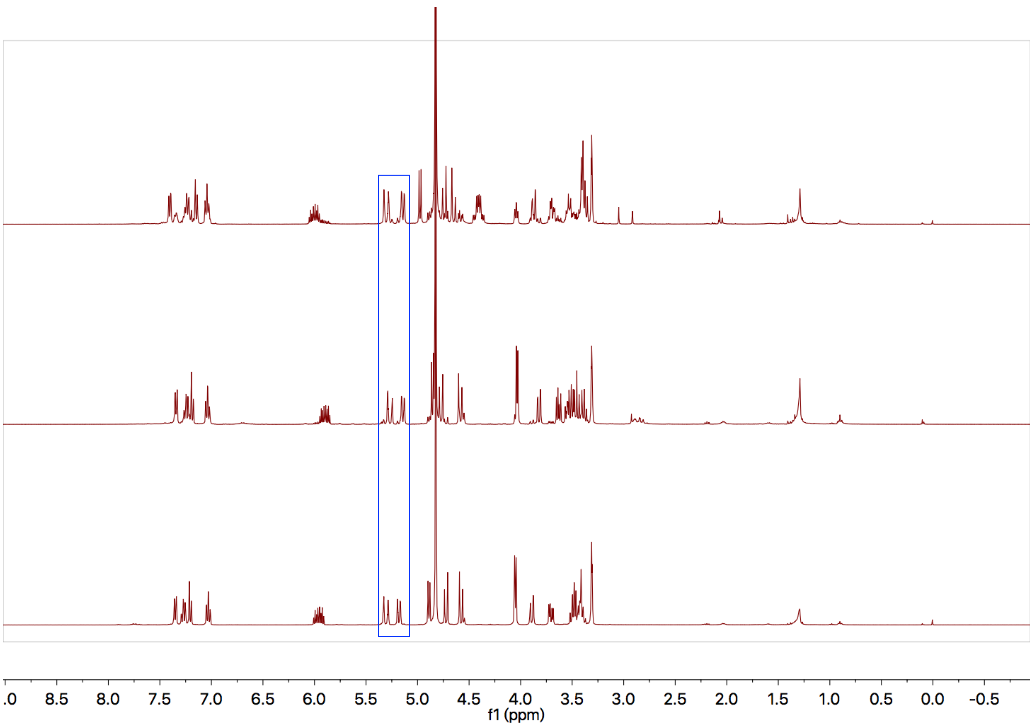

2'-*O*-(allyl)-salicin (20a)

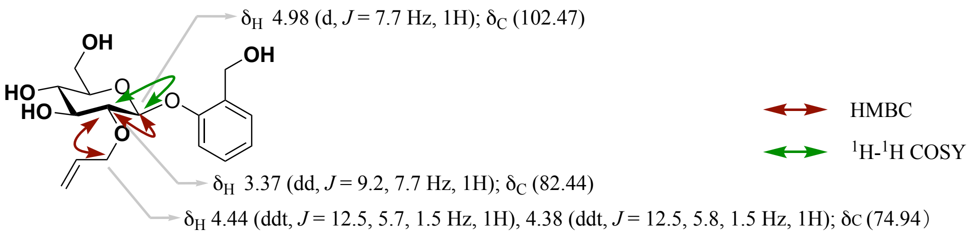

In a N_2-filled glovebox, salicin **20** (57.2 mg, TCI Lot YPHNL-AT , 0.20 mmol, 1.0 equiv), and *N*,*N*-dimethyl acetamide (1.2 mL) were weighed into a Schlenk tube (labeled as **Tube A**) containing a stir bar.

To a screw vial, compound **2** (69.6 mg, 0.44 mmol, 2.2 equiv), $Pd_2(dba)_3 \cdot CHCl_3$ (10.4 mg, 0.01 mmol, 0.05 equiv), PPh_3 (10.4 mg, 0.02 mmol, 0.1 equiv), and CH_2Cl_2 (400 μL) were added in sequence. The mixture was stirred for 10 min, and then transferred to **Tube A**. An additional portion of CH_2Cl_2 (2.0 mL) was added to Tube A so that [**20**] was adjusted to 0.055 M. **Vial A** was tightly capped, taken out of the glove box, and stirred at 25 ˚C for 8 h (400 rpm). The reaction mixture was then concentrated in vacuo. The residue was passed through a pad of silica gel. An aliquot of the residue was taken for ^1H NMR analysis, which indicated that products **20a**, **20b** and **20c** were formed in a ratio of 5:1:1. Flash chromatography (SiO_2) using CH_2Cl_2/MeOH (1:0 to 54:1 to 27:1 to 18:1 to 7:1) as eluent afforded the C3-OH allylated product and C4-OH allylated product as a mixture (6.5 mg, C3-OH: C4-OH = 1:2.5, 10%), **20a** (23.5 mg, 0.072 mmol, **20a:20b:20c** = 5:1:1, 36%), and recovered starting material **20** (18.1 mg, 32%).

^{1}H NMR ($CDCl_3$, 400 MHz) δ: 7.40 (dd, *J* = 7.5, 1.7 Hz, 1H), 7.24 (td, *J* = 7.9, 2.0 Hz, 1H), 7.15 (dd, *J* = 8.3, 1.2 Hz, 1H), 7.04 (td, *J* = 7.4, 1.2 Hz, 1H), 6.00 (ddt, *J* = 17.3, 10.4, 5.7 Hz, 1H), 5.30 (dq, *J* = 17.3, 1.8 Hz, 1H), 5.14 (dq, *J* = 10.4, 1.4 Hz, 1H), 4.98 (d, *J* = 7.7 Hz, 1H), 4.74 (d, *J* = 13.3 Hz, 1H), 4.65 (d, *J* = 13.4 Hz, 1H), 4.44 (ddt, *J* = 12.5, 5.7, 1.5 Hz, 1H), 4.38 (ddt, *J* = 12.5, 5.8, 1.5 Hz, 1H), 3.87 (dd, *J* = 12.1, 1.6 Hz, 1H), 3.69 (dd, *J* = 11.8, 4.8 Hz, 1H), 3.72 – 3.66 (m, 1H), 3.58 – 3.51 (m, 1H), 3.42–3.39 (m, 2H), 3.37 (dd, *J* = 9.2, 7.7 Hz, 1H).
^{13}C NMR ($CDCl_3$, 101 MHz) δ: 156.1, 136.6, 132.0, 129.5, 129.1, 123.6, 117.1, 116.5, 102.5, 82.4, 78.0, 77.8, 74.9, 71.4, 62.5, and 60.4.

6'-*O*-(allyl)-salicin (20b)

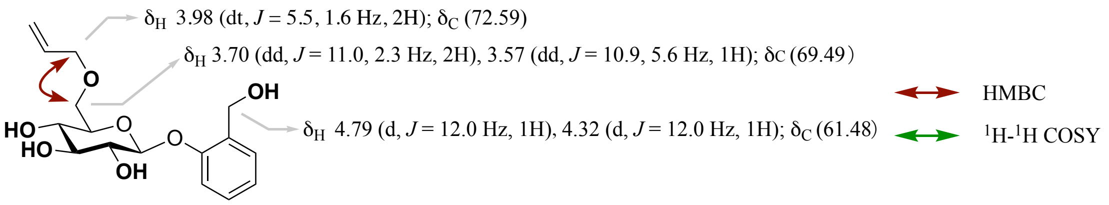

In a N_2-filled glovebox, salicin **20** (57.2 mg, 0.20 mmol, 1.0 equiv), 10*H*-dibenzo[*b*,*e*][1,4]oxaborinin-10-ol[5] (39.2 mg, 0.20 mmol, 1.0 equiv), and THF (500 μL) were weighed into a screw capped vial (labeled as **Vial A**) containing a stir bar. The resulting solution was stirred for an additional 30 min.

To another vial, compound **2** (50.6 mg, 0.16 mmol, 1.6 equiv), $Pd_2(dba)_3$•$CHCl_3$ (5.2 mg, 0.005 mmol, 0.025 equiv), PPh_3 (5.2 mg, 0.02 mmol, 0.1 equiv), and THF (200 μL) were added in sequence. The mixture was stirred for 10 min, and then transferred to **Vial A**. An additional portion of THF (1.3 mL) was added to Vial A so that [**20**] was adjusted to 0.1 M. **Vial A** was tightly capped, taken out of the glove box, and stirred at 25 ˚C for 12 h (400 rpm). The reaction mixture was then concentrated in vacuo. An aliquot of the residue was taken for ^{1}H NMR analysis, which indicated that products **20b** and **20c** were formed in a ratio of 6:1. Flash chromatography (SiO_2) using CH_2Cl_2/MeOH (1:0 to 18:1 to 7:1) as eluent afforded diallylated products (4.0 mg, 5%), **20b** as a light yellow oil (42 mg, 0.128 mmol, 64%), recovered starting material **20** (10.4 mg, 35%).

4-Dimethylaminophenylboronic acid could be used as an additive instead.

^{1}H NMR ($CDCl_3$, 400 MHz) δ: 7.24 – 7.13 (m, 2H), 7.08 (d, J = 8.4 Hz, 1H), 6.97 (t, J = 7.3 Hz, 1H), 5.85 (ddt, J = 15.9, 10.7, 5.5 Hz, 1H), 5.23 (dq, J = 17.2, 1.8 Hz, 1H), 5.13 (ddd, J = 10.4, 3.1, 1.4 Hz, 1H), 4.79 (d, J = 12.0 Hz, 1H), 4.65 (d, J = 7.7 Hz, 1H), 4.32 (d, J = 12.0 Hz, 1H), 3.98 (dt, J = 5.8, 1.6 Hz, 1H), 3.70 (dd, J = 11.0, 2.3 Hz, 2H), 3.61 (dd, J = 8.8, 8.0 Hz, 1H), 3.57 (dd, J = 10.9, 5.6 Hz, 1H), 3.49 (t, J = 8.2 Hz, 1H), 3.45 (t, J = 8.8 Hz, 1H), and 3.40 – 3.30 (m, 1H). **^{13}C NMR ($CDCl_3$, 101 MHz)** δ: 156.8, 134.7, 130.5, 130.0, 129.8, 123.3, 117.2, 117.0, 102.6, 76.5, 75.5, 73.6, 72.6, 70.4, 69.5, and 61.5. **IR (thin film, cm^{-1})**: 3344, 2922, 2876, 1604, 1492, 1455, 1234, 1059, 1005, and755. **HRMS (DART-TOF)** calculated for $C_{16}H_{22}NaO_7^+$ $[M+Na]^+$ m/z 349.1258, found 349.1259. **$[\alpha]_D^{27}$** = –32.2 (c = 0.68, $CHCl_3$).

1-*O*-(allyl)-salicin (20c)

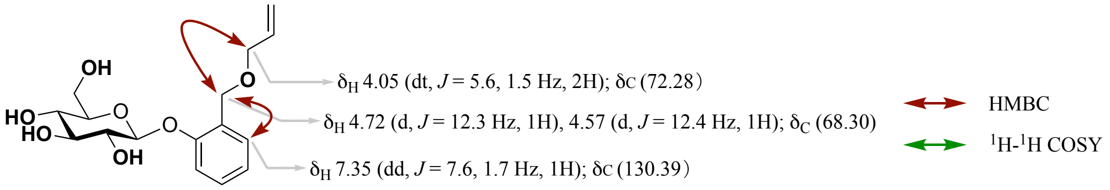

In a N_2-filled glovebox, salicin **20** (114.4 mg, 0.40 mmol, 1.0 equiv), BPh_3 (0.25 M in THF, Sigma Aldrich Lot SHBH1129V, 1.6 mL, 0.40 mmol, 1.0 equiv), and THF (500 μL) were weighed into a screw capped vial (labeled as **Vial A**) containing a stir bar. The resulting solution was stirred for an additional 30 min.

To another vial, compound **2** (101.1 mg, 0.64 mmol, 1.6 equiv), $Pd_2(dba)_3$•$CHCl_3$ (10.4 mg, 0.01 mmol, 0.025 equiv), PPh_3 (10.4 mg, 0.04 mmol, 0.1 equiv), and THF (200 μL) were added in sequence. The mixture was stirred for 10 min, and then transferred to **Vial A**. An

additional portion of THF (1.7 mL) was added to Vial A so that [**20**] was adjusted to 0.1 M. **Vial A** was tightly capped, taken out of the glove box, and stirred at 25 ˚C (plate temperature) for 12 h (400 rpm). The reaction mixture was then concentrated in vacuo. An aliquot of the residue was taken for ^{1}H NMR analysis, which indicated that products **20a**, **20b** and **20c** were formed in a ratio of <1:1:19. Flash chromatography (SiO_2) using CH_2Cl_2/MeOH (27:1 to 18:1 to 7:1) as eluent afforded **20c** as a light yellow oil (54.7 mg, 0.168 mmol, 42%) and recovered starting material **20** (63.0 mg, 55%).

Note: The quality of BPh_3 is critical for the performance of this reaction. Many commercial sources of solid BPh_3 contain boronic acids or borinic acids as impurities. We note the commercial THF solution of BPh_3 gave consistent results.

^{1}H NMR (CD_3OD, 400 MHz) δ: 7.35 (dd, *J* = 7.6, 1.7 Hz, 1H), 7.27 (td, *J* = 7.8, 7.3, 1.8 Hz, 1H), 7.20 (dd, *J* = 8.3, 1.2 Hz, 1H), 7.03 (td, *J* = 7.4, 1.3 Hz, 1H), 5.95 (ddt, *J* = 17.4, 10.8, 5.6 Hz, 1H), 5.31 (dq, *J* = 17.3, 1.7 Hz, 1H), 5.18 (dq, *J* = 10.5, 1.5 Hz, 1H), 4.89 (d, *J* = 7.2 Hz, 1H), 4.72 (d, *J* = 12.3 Hz, 1H), 4.57 (d, *J* = 12.4 Hz, 1H), 4.05 (dt, *J* = 5.6, 1.5 Hz, 2H), 3.89 (dd, *J* = 12.0, 1.9 Hz, 1H), 3.70 (dd, *J* = 12.0, 4.9 Hz, 1H), and 3.52 – 3.36 (m, 4H). **^{13}C NMR (CD_3OD, 101 MHz)** δ: 157.1, 136.0, 130.4, 130.2, 128.9, 123.5, 117.5, 116.8, 103.2, 78.2, 78.0, 75.1, 72.3, 71.4, 68.3 and 62.6. **IR (thin film, cm^{-1})**: 3370, 2920, 2874, 1590, 1491, 1455, 1235, 1072, 1042, and 756. **HRMS (DART-TOF)** calculated for $C_{16}H_{22}NaO_7^+$ $[M+Na]^+$ m/z 349.1258, found 349.1256. **$[\alpha]_D^{27}$** = –11.0 (c = 0.23, $CHCl_3$).

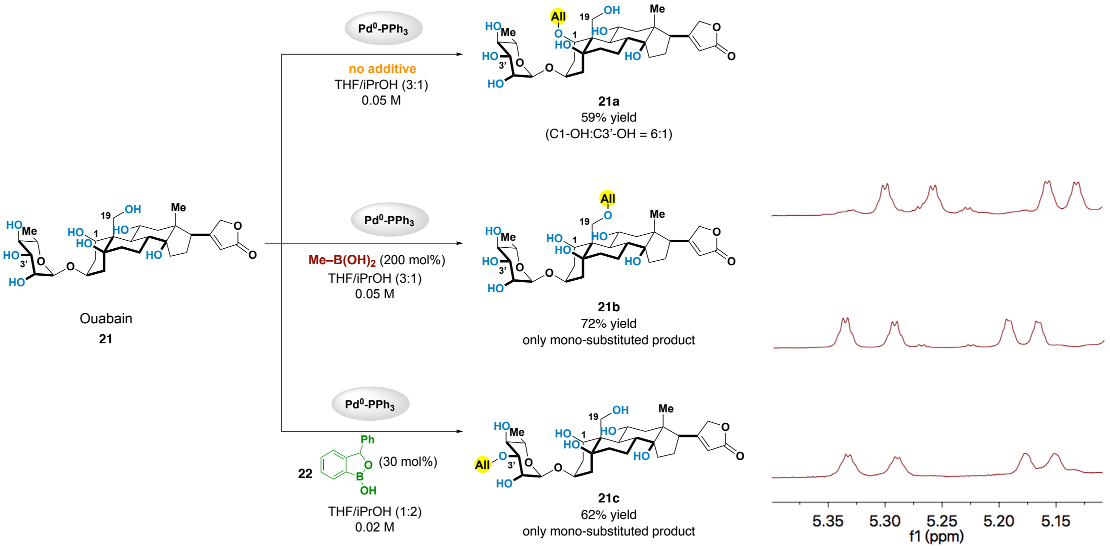

The full NMR spectra of the reaction mixtures were shown below.

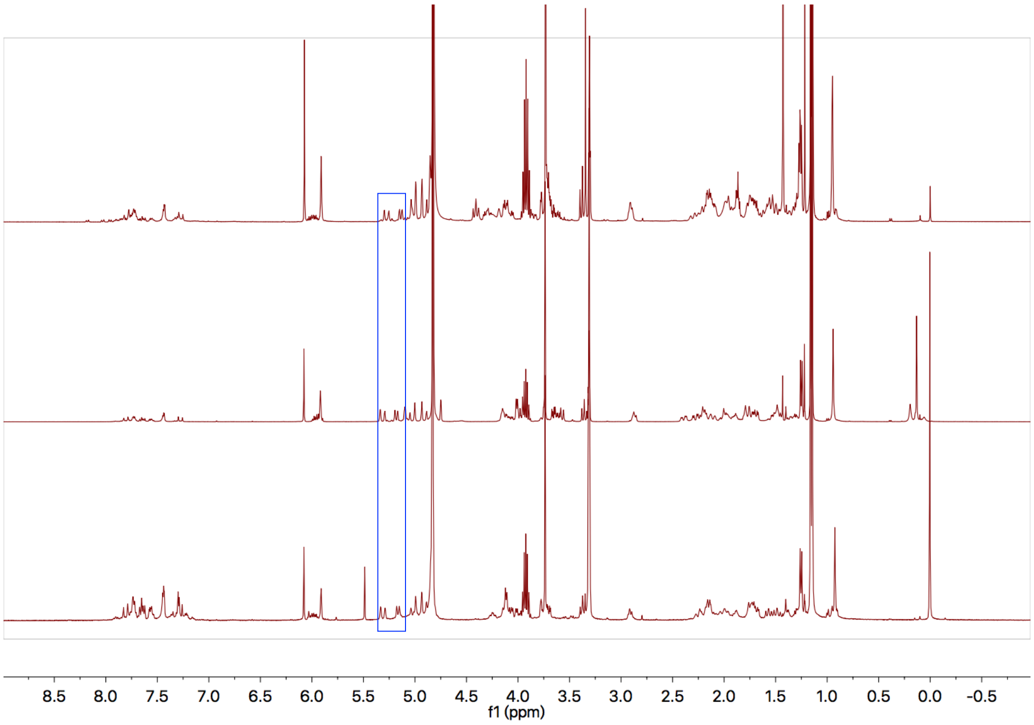

1-*O*-(allyl)- ouabain(21a)

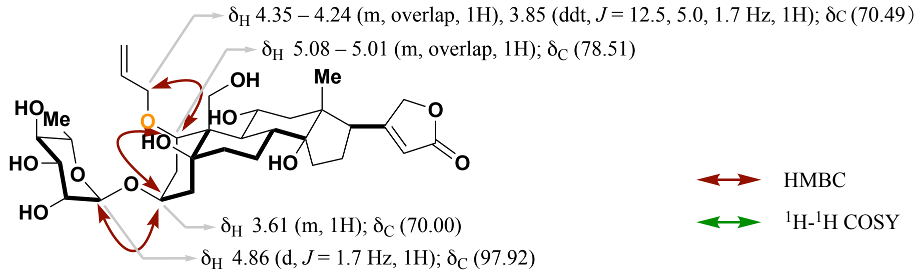

In a N_2-filled glovebox, ouabain octahydrate **21** (36.4 mg, Acros Lot A0396776, 0.05 mmol, 1.0 equiv), and *i*PrOH (250 µL) were weighed into a screw capped vial (labeled as **Vial A**) containing a stir bar.

To another vial, compound **2** (12.0 mg, 0.075 mmol, 1.5 equiv), $Pd_2(dba)_3$•$CHCl_3$ (1.3 mg, 1.3 μmol, 0.025 equiv), PPh_3 (1.3 mg, 0.005 mmol, 0.1 equiv), and THF (200 μL) were added in sequence. The mixture was stirred for 10 min, and then transferred to **Vial A**. An additional portion of THF (550 μL) was added to Vial A so that [**21**] was adjusted to 0.05 M. **Vial A** was tightly capped, taken out of the glove box, and stirred at 25 ˚C for 12 h (400 rpm). The reaction mixture was then concentrated in vacuo. An aliquot of the residue was taken for ^{1}H NMR analysis, which indicated that products **21a**, **21b** and **21c** were formed in a ratio of 6:0:1. Flash chromatography (SiO_2) using CH_2Cl_2/MeOH (1:0 to 9:1 to 7:1) as eluent afforded **21a** as a white foam (18.7 mg, 0.030 mmol, **21a:21c** = 6:1, 59%) and recovered starting material **21** (8.7 mg, ca. 30%).

^{1}H NMR (CD_3OD, 400 MHz) δ: 5.99 (ddt, *J* = 17.3, 10.4, 5.1 Hz, 1H), 5.91 (5.91 (t, *J* = 1.8 Hz, 1H), 5.28 (dq, *J* = 17.2, 1.7 Hz, 1H), 5.14 (dq, *J* = 10.5, 1.6 Hz, 1H), 5.02 (dd, *J* = 18.5, 1.9 Hz, 1H), 5.08 – 5.01 (m, 1H), 4.91 (dd, *J* = 18.4, 1.8 Hz, 1H), 4.86 (d, *J* = 1.7 Hz, 1H), 4.42 (d, *J* = 11.3 Hz, 1H), 4.35 – 4.24 (m, 2H), 4.19 (br s, 1H), 4.12 (d, *J* = 11.2 Hz, 1H), 3.85 (ddt, *J* = 12.5, 5.0, 1.7 Hz, 1H), 3.70 (dd, *J* = 3.5, 1.6 Hz, 1H), 3.66 (dd, *J* = 9.3, 3.4 Hz, 1H), 3.61 (dt, *J* = 9.4, 6.2 Hz, 1H), 3.37 (t, *J* = 9.5 Hz, 1H), 2.93 – 2.88 (m, 1H), 2.35 – 2.26 (m, 1H), 2.21 – 2.06 (m, 4H), 2.02 – 1.87 (m, 3H), 1.66 – 1.45 (m, 3H), 1.80 – 1.66 (m, 3H), 1.39 – 1.35 (m, 1H), 1.35 – 1.31 (m, 3H), 1.31 – 1.28 (m, 2H), 1.27 (d, *J* = 6.2 Hz, 3H), and 0.95 (s, 3H). **^{13}C NMR (CD_3OD, 101 MHz)** δ: 177.6, 177.1, 136.6, 118.0, 116.2, 97.9, 85.6, 78.5, 75.8, 75.3, 74.2, 72.4, 72.3, 70.5, 70.1, 70.0, 69.3, 60.8, 51.7, 51.0, 50.4, 49.9, 48.0, 41.1, 35.7, 33.6, 27.9, 24.2, 18.1, 17.6, and 9.2. **IR (thin film, cm^{-1})**: 3389, 2933, 1730, 1415, 1381, 1046, 981, and 794. **HRMS (DART-TOF)** calculated for $C_{32}H_{48}NaO_{12}^+$[M+Na]$^+$ m/z 647.3038, found 647.3038. **$[\alpha]_D^{25}$** = –22.4 (c = 1.08, MeOH)

19-*O*-(allyl)-ouabain(21b)

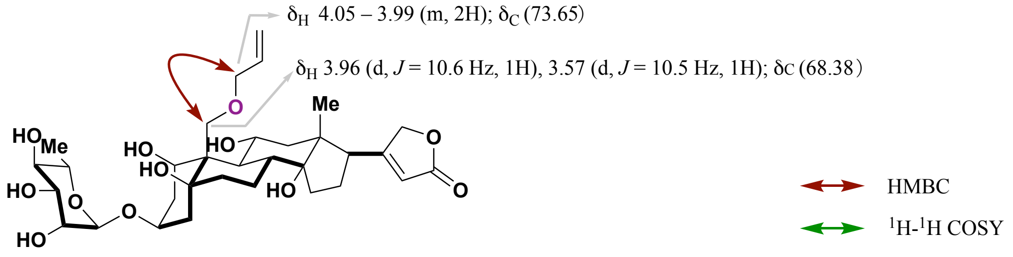

In a N_2-filled glovebox, ouabain octahydrate **21** (36.4mg, 0.05 mmol, 1.0 equiv), methylboronic acid (6.0 mg, Energy Chemicals Lot ED200014, 0.10 mmol, 2.0 equiv), and

*i*PrOH (250 μL) were weighed into a screw capped vial (labeled as **Vial A**) containing a stir bar. The resulting solution was stirred for an additional 30 min.

To another vial, compound **2** (11.9 mg, 0.075 mmol, 1.5 equiv), $Pd_2(dba)_3$•$CHCl_3$ (1.3 mg, 1.3 μmol, 2.5% equiv), PPh_3 (1.3 mg, 0.005 mmol, 0.1 equiv), and THF (200 μL) were added in sequence. The mixture was stirred for 10 min, and then transferred to **Vial A**. An additional portion of THF (550 μL) was added to Vial A so that [**21**] was adjusted to 0.05 M. **Vial A** was tightly capped, taken out of the glove box, and stirred at 25 ˚C for 12 h (400 rpm). The reaction mixture was then concentrated in vacuo. An aliquot of the residue was taken for ^{1}H NMR analysis. Flash chromatography (SiO_2) using CH_2Cl_2/MeOH (15:1 to 10:1) as eluent afforded **21b** as a pale yellow foam (22.5 mg, 0.036 mmol, 72.3%).

^{1}H NMR (CD_3OD, 400 MHz) δ: 5.94 (ddt, *J* = 17.2, 10.4, 5.6 Hz, 1H), 5.92 (t, *J* = 1.6 Hz 1H), 5.31 (dq, *J* = 17.3, 1.7 Hz, 1H), 5.18 (dq, *J* = 10.4, 1.4 Hz, 1H), 5.09 (dd, *J* = 3.9, 2.0 Hz, 1H), 5.02 (dd, *J* = 18.4, 1.8 Hz, 1H), 4.91 (dd, *J* = 18.4, 1.7 Hz, 1H), 4.75 (d, *J* = 1.6 Hz, 1H), 4.18 – 4.12 (m, 1H), 4.09 (dd, *J* = 10.8, 4.4 Hz, 1H), 4.05 – 3.99 (m, 2H), 3.96 (d, *J* = 10.6 Hz, 1H), 3.74 (dd, *J* = 3.4, 1.6 Hz, 1H), 3.65 (dd, *J* = 9.5, 3.5 Hz, 1H), 3.62 – 3.59 (m, 1H), 3.57 (d, *J* = 10.5 Hz, 1H), 3.35 (t, *J* = 9.5 Hz, 1H), 2.87 (dd, *J* = 8.7, 5.4 Hz, 1H), 2.39 (ddd, *J* = 15.6, 5.3, 2.2 Hz, 1H), 2.28 (dd, *J* = 15.6, 4.8 Hz, 1H), 2.24 – 2.16 (m, 2H), 2.15 – 2.06 (m, 1H), 2.05 – 1.93 (m, 2H), 1.90 (dt, *J* = 9.4, 4.5 Hz, 1H), 1.77 (dd, *J* = 15.2, 2.8 Hz, 2H), 1.74 – 1.65 (m, 2H), 1.59 – 1.42 (m, 3H), 1.40 – 1.29 (m, 2H),1.25 (d, *J* = 6.2 Hz, 3H) and 0.94 (s, 3H). **^{13}C NMR (CD_3OD, 101 MHz)** δ: 177.6, 177.1 136.3, 118.1, 117.6, 98.9, 85.5, 75.3, 74.5, 74.2, 73.7, 72.3, 72.2, 71.1, 70.1, 70.1, 69.1, 68.4, 51.8, 51.3, 50.5, 47.2, 45.7, 40.9, 34.8, 34.8, 33.3, 33.2, 28.0, 24.1, 18.0 and 17.5. **IR (thin film, cm^{-1})**: 3393, 2928, 1731, 1390, 1341, 1042, and 672. **HRMS (DART-TOF)** calculated for $C_{32}H_{48}NaO_{12}{}^+$[M+Na]$^+$ m/z 647.3038, found 647.3049. **$[\alpha]_D^{26}$** = –25.1 (c = 0.51, MeOH).

3'-*O*-(allyl)-ouabain (21c)

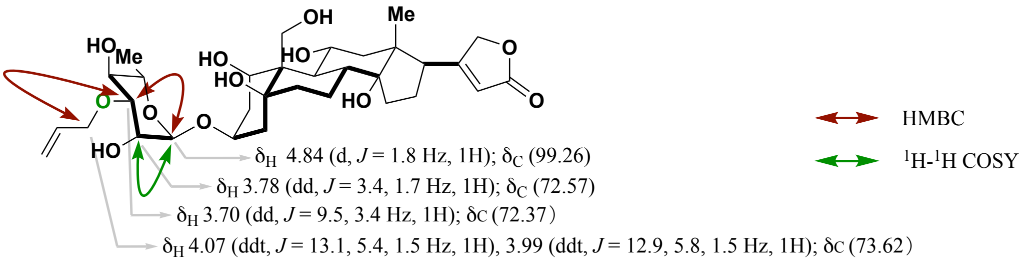

In a N_2-filled glovebox, ouabain octahydrate **21** (36.4mg, 0.05 mmol, 1.0 equiv), 3-phenylbenzo[*c*][1,2]oxaborol-1(3*H*)-ol[8] (3.0 mg, 0.03 mmol, 0.3 equiv), THF (500 μL), and

*i*PrOH (2.0 mL) were weighed into a screw capped vial (labeled as **Vial A**) containing a stir bar. The resulting solution was stirred for an additional 30 min.

To another vial, compound **2** (12.0 mg, 0.075 mmol, 1.5 equiv), $Pd_2(dba)_3$•$CHCl_3$ (1.3 mg, 1.3 μmol, 2.5% equiv), PPh_3 (1.3 mg, 0.005 mmol, 0.1 equiv), and THF (200 μL) were added in sequence. The mixture was stirred for 10 min, and then transferred to **Vial A**. An additional portion of THF (300 μL) was added to Vial A so that [**21**] was adjusted to 0.016 M. **Vial A** was tightly capped, taken out of the glove box, and stirred at 25 ˚C for 12 h (400 rpm). The reaction mixture was then concentrated in vacuo. An aliquot of the residue was taken for ^{1}H NMR analysis. Flash chromatography (SiO_2) using CH_2Cl_2/MeOH (9:1 to 7:1) as eluent afforded diallylated products (2.3 mg, 6%) and **21c** as a white solid (19.4 mg, 0.031 mmol, 62%).

^{1}H NMR (CD_3OD, 400 MHz) δ: 5.99 (ddt, *J* = 17.3, 10.8, 5.6 Hz, 1H), 5.91 (t, *J* = 1.8 Hz, 1H), 5.31 (dq, *J* = 17.2, 1.7 Hz, 1H), 5.16 (dq, *J* = 10.4, 1.4 Hz, 1H), 5.01 (dd, *J* = 18.4, 1.8 Hz, 1H), 4.91 (dd, *J* = 18.3, 1.7 Hz, 1H), 4.84 (d, *J* = 1.8 Hz, 1H), 4.24 (td, *J* = 10.3, 4.4 Hz, 1H), 4.17 – 4.10 (m, 3H), 4.07 (ddt, *J* = 13.1, 5.4, 1.5 Hz, 1H), 3.99 (ddt, *J* = 12.9, 5.8, 1.5 Hz, 1H), 3.78 (dd, *J* = 3.4, 1.7 Hz, C2'-*H*, 1H), 3.74 (dq, *J* = 9.6, 6.5 Hz, C5'-*H*, 1H), 3.70 (dd, *J* = 9.5, 3.4 Hz, C3'-*H*, 1H), 3.35 (dd, *J* = 9.6, 9.6 Hz, C4'-*H*, 1H), 2.91 (t, *J* = 7.2 Hz, 1H), 2.30 – 1.83 (m, 8H), 1.81 – 1.64 (m, 4H), 1.57 (dd, *J* = 11.9, 9.7 Hz, 1H), 1.49 (dd, *J* = 13.4, 10.9 Hz, 1H), 1.43 – 1.27 (m, 3H), 1.25 (d, *J* = 6.3 Hz, 3H), and 0.92 (s, 3H). **^{13}C NMR (CD_3OD, 101 MHz)** δ: 177.6, 177.1, 136.5, 118.0, 117.2, 99.3, 85.7, 75.9, 75.3, 74.2, 73.6, 72.6, 72.4, 72.4, 71.8, 70.4, 69.9, 69.0, 51.6, 50.9, 50.3, 41.2, 41.1, 36.1, 33.4, 27.9, 24.5, 18.0, and 17.5. **IR (thin film, cm^{-1})**: 3407, 2934, 2857, 1734, 1624, 1437, 1275, 1260, 1045, 764, and 749. **Mp**: 219 – 220 ºC. **HRMS (DART-TOF)** calculated for $C_{32}H_{48}NaO_{12}^+$[M+Na]$^+$ m/z 647.3038, found 647.3043. **$[\alpha]_D^{25}$** = –7.3 (c = 1.17, MeOH).

1-*O*-(4-CF_3-cinnamyl)- ouabain(21a')

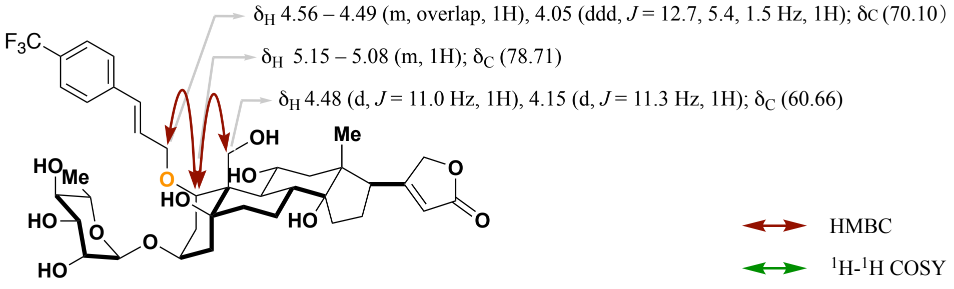

In a N_2-filled glovebox, ouabain octahydrate **21** (29.2 mg, Acros Lot A0396776, 0.04 mmol, 1.0 equiv), 4-nitrophenyl boronic acid (0.33 mg in 200 μL of 1:1 THF/*i*PrOH), and *i*PrOH (100 μL) were weighed into a screw capped vial (labeled as **Vial A**) containing a stir bar. The resulting solution was stirred for an additional 30 min.

To another vial, (*E*)-*tert*-butyl (3-(4-(trifluoromethyl)phenyl)allyl) carbonate (18.1 mg, 0.06 mmol, 1.5 equiv), $Pd_2(dba)_3$•$CHCl_3$ (1.0 mg, 1.0 μmol, 0.025 equiv), PPh_3 (1.0 mg, 0.004 mmol, 0.1 equiv), and THF (200 μL) were added in sequence. The mixture was stirred for 10 min, and then transferred to **Vial A**. An additional portion of THF (300 μL) was added to **Vial A** so that [**21**] was adjusted to 0.05 M. **Vial A** was tightly capped, taken out of the glove box, and stirred at 25 ˚C for 12 h (400 rpm). The reaction mixture was then concentrated in vacuo. Flash chromatography (SiO_2, 3 g) using CH_2Cl_2/MeOH (20:1 to 10:1 to 7:1) as eluent afforded **21a'** as a white foam (11.0 mg, 0.014 mmol, 35%) and recovered starting material **21** (18.7 mg, ca. 64%)

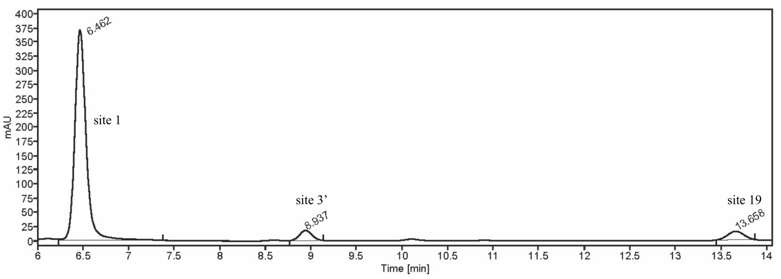

Signal: DAD1A, Sig=220, 4 Ref=off

RT [min]	Type	Width [min]	Area	Height	Area%
6.5	MM m	0.1	3173.9	370	90.1
8.9	MM m	0.1	166.0	18	4.7
13.7	MM m	0.2	184.4	15	5.2
		Sum	3524.3		100.0

^{1}H NMR (CD_3OD, 400 MHz) δ: 7.64 (d, *J* = 8.2 Hz, 2H), 7.58 (d, *J* = 8.3 Hz, 2H), 6.73 (d, *J* = 16.0 Hz, 1H), 6.57 (dt, *J* = 16.1, 5.5 Hz, 1H), 5.91 (d, *J* = 1.8 Hz, 1H), 5.15 – 5.08 (m, 1H), 5.02 (dd, *J* = 18.5, 1.8 Hz, 1H), 4.92 (dd, *J* = 18.6, 1.8 Hz, 1H), 4.88 (d, *J* = 1.3 Hz, 1H), 4.56 – 4.49 (m, 2H), 4.48 (d, *J* = 11.0 Hz, 1H), 4.32 (td, *J* = 10.5, 4.3 Hz, 1H), 4.24 – 4.19 (m, 1H), 4.15 (d, *J* = 11.3 Hz, 1H), 4.05 (ddd, *J* = 12.7, 5.4, 1.5 Hz, 1H), 3.75 – 3.70 (m, 1H), 3.71 (t, *J* = 3.5 Hz, 1H), 3.66 (dt, *J* = 9.5, 6.1 Hz, 1H), 3.40 (t, *J* = 9.2 Hz, 1H), 2.91 (dd, *J* = 8.7, 5.8 Hz, 1H), 2.41 – 2.32 (m, 1H), 2.22 – 2.09 (m, 4H), 2.03 – 1.86 (m, 3H), 1.80 – 1.45 (m, 7H), 1.40 – 1.30 (m, 3H), 1.29 (d, *J* = 6.2 Hz, 3H), and 0.96 (s, 3H). **^{13}C NMR (CD_3OD, 101 MHz)** δ: 177.6, 177.1, 142.4, 131.1, 131.0, 128.1, 127.1, 126.4 (q, *J* = 3.8 Hz), 118.0, 98.0, 85.7, 78.7, 75.9, 75.3, 74.3, 72.6, 72.3, 70.3, 70.1, 70.0, 69.4, 60.7, 51.7, 51.0, 50.4, 50.0, 48.1, 41.2, 35.7,

33.6, 30.8, 27.9, 24.3, 18.1, and 17.6.**HRMS (DART-TOF)** calculated for $C_{39}H_{51}F_3NaO_{12}^+$ $[M+Na]^+$ m/z 791.3225, found 791.3230.

19-*O*-(4-OMe-Cinnamyl)-ouabain(21b')

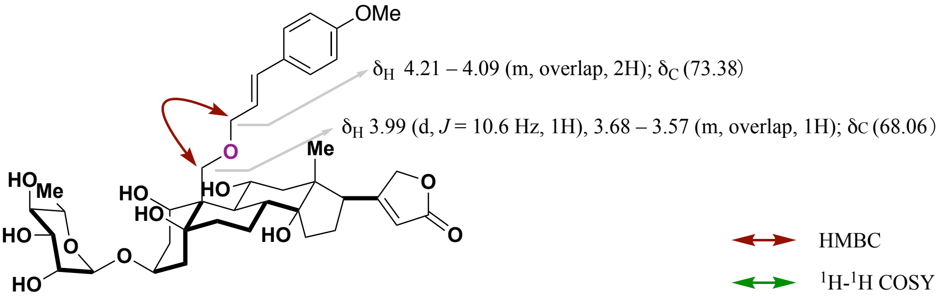

In a N_2-filled glovebox, ouabain octahydrate **21** (29.2 mg, 0.04 mmol, 1.0 equiv), methylboronic acid (4.8 mg, Energy Chemicals Lot ED200014, 0.08 mmol, 2.0 equiv), and *i*PrOH (200 μL) were weighed into a screw capped vial (labeled as **Vial A**) containing a stir bar. The resulting solution was stirred for an additional 30 min.

To another vial, compound (*E*)-*tert*-butyl (3-(4-methoxyphenyl)allyl) carbonate (15.9 mg, 0.06 mmol, 1.5 equiv), $Pd_2(dba)_3$•$CHCl_3$ (1.0 mg, 1.0 μmol, 2.5% equiv), PPh_3 (1.0 mg, 0.004 mmol, 0.1 equiv), and THF (200 μL) were added in sequence. The mixture was stirred for 10 min, and then transferred to **Vial A**. An additional portion of THF (400 μL) was added to Vial A so that [**21**] was adjusted to 0.05 M. **Vial A** was tightly capped, taken out of the glove box, and stirred at 25 ˚C for 12 h (400 rpm). The reaction mixture was then concentrated in vacuo. Flash chromatography (SiO_2) using CH_2Cl_2/MeOH (20:1 to 9:1) as eluent afforded **21b'** as a white foam (20.5 mg, 0.028 mmol, 70%).

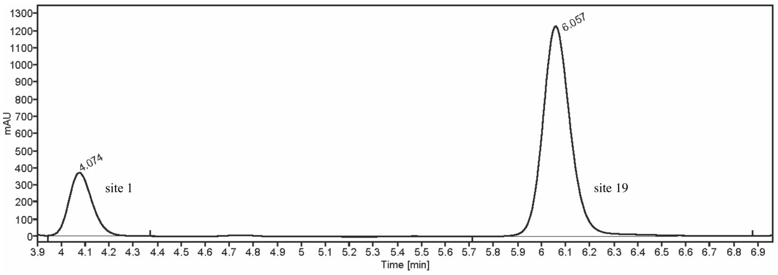

Signal: DAD1A, Sig=220, 4 Ref=off

RT [min]	Type	Width [min]	Area	Height	Area%
4.1	MM m	0.1	2469.4	369	19.9
6.1	MM m	0.1	9926.7	1230	80.1
		Sum	12396.1		100.0

^{1}H NMR (CD$_3$OD, 400 MHz) δ: 7.34 (d, J = 8.8 Hz, 2H), 6.86 (d, J = 8.8 Hz, 2H), 6.59 (d, J = 15.9 Hz, 1H), 6.20 (dt, J = 15.9, 6.2 Hz, 1H), 5.91 (t, J = 1.6 Hz, 1H), 5.13 (dd, J = 3.6, 2.0 Hz, 1H), 5.01 (dd, J = 18.4, 1.8 Hz, 1H), 4.90 (dd, J = 18.4, 1.7 Hz, 1H), 4.74 (d, J = 1.7 Hz,

1H), 4.21 – 4.09 (m, 3H), 3.99 (d, J = 10.6 Hz, 1H), 3.78 (s, 3H), 3.74 (dd, J = 3.5, 1.7 Hz, 1H), 3.64 (dd, J = 9.0, 2.9 Hz, 1H), 3.68 – 3.57 (m, 2H), 3.36 (t, J = 9.5 Hz, 1H), 2.87 (t, J = 7.1 Hz, 1H), 2.40 (ddd, J = 15.7, 5.3, 2.1 Hz, 1H), 2.28 (dd, J = 15.7, 4.8 Hz, 1H), 2.24 – 2.08 (m, 3H), 2.01 (t, J = 10.9 Hz, 3H), 1.89 (q, J = 4.6 Hz, 1H), 1.82 – 1.66 (m, 4H), 1.56 – 1.42 (m, 3H), 1.37 – 1.27 (m, 8H), 1.25 (d, J = 6.2 Hz, 3H), and 0.92 (s, 3H). **^{13}C NMR (CD_3OD, 101 MHz)** δ: 177.6, 177.1, 160.9, 133.8, 130.8, 128.8, 128.8 (q, J = 32.4 Hz), 124.8, 118.1, 115.0, 99.0, 85.5, 75.3, 74.5, 74.2, 73.4, 72.3, 72.2, 71.1, 70.1, 70.1, 69.2, 68.1, 55.7, 51.4, 50.5, 49.9, 47.3, 45.7, 40.9, 34.8, 33.3, 30.7, 28.0, 24.1, 18.0, and 17.5. **HRMS (DART-TOF)** calculated for $C_{39}H_{54}NaO_{13}{}^+$[M+Na]$^+$ m/z 753.3457, found 753.3471.

3’-*O*-(4-alkynyl cinnamyl)- ouabain(21c’)

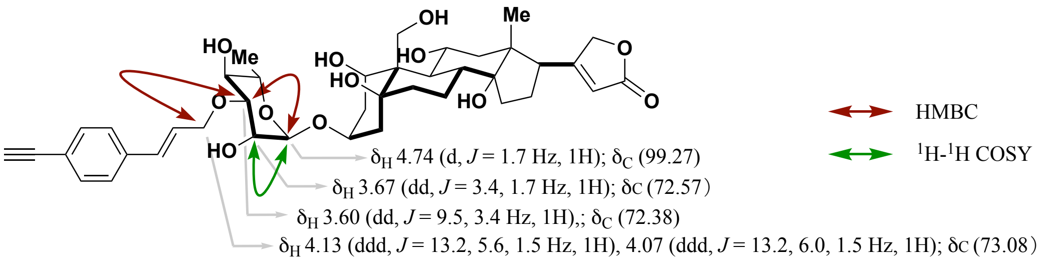

In a N_2-filled glovebox, ouabain octahydrate **21** (29.2 mg, 0.04 mmol, 1.0 equiv), 3-phenylbenzo[*c*][1,2]oxaborol-1(3*H*)-ol (2.5 mg, 0.012 mmol, 0.3 equiv), THF (500 μL), and *i*PrOH (1.6 mL) were weighed into a screw capped vial (labeled as **Vial A**) containing a stir bar. The resulting solution was stirred for an additional 30 min.

To another vial, compound (*E*)-*tert*-butyl (3-(4-ethynylphenyl)allyl) carbonate (15.5 mg, 0.06 mmol, 1.5 equiv), $Pd_2(dba)_3$•$CHCl_3$ (1.0 mg, 1.0 μmol, 2.5% equiv), PPh_3 (1.0 mg, 0.004 mmol, 0.1 equiv), and THF (200 μL) were added in sequence. The mixture was stirred for 10 min, and then transferred to **Vial A**. An additional portion of THF (100 μL) was added to Vial A so that [**21**] was adjusted to 0.016 M. **Vial A** was tightly capped, taken out of the glove box, and stirred at 25 ˚C for 12 h (400 rpm). The reaction mixture was then concentrated in vacuo. Flash chromatography (SiO_2) using CH_2Cl_2/MeOH (18:1 to 9:1) as eluent afforded **21c’** as a white foam (15.2 mg, 0.024 mmol, 61%).

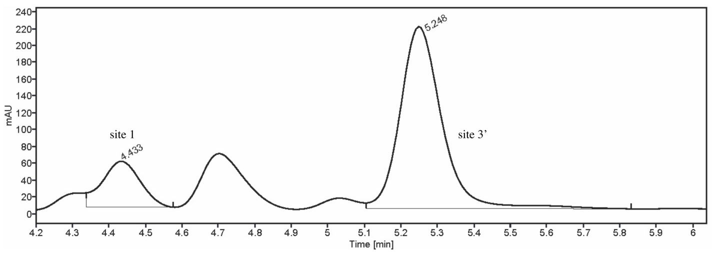

Signal: DAD1B, Sig=254, 4 Ref=off

RT [min]	Type	Width [min]	Area	Height	Area%
4.4	MM m	0.1	394.4	54	18.7
5.2	MM m	0.1	1719.7	216	81.3
		Sum	2114.1		100.0

^{1}H NMR (CD_3OD, 400 MHz) δ: 7.29 (br s, 4H), 6.56 (dt, *J* = 16.0, 1.5 Hz, 1H), 6.34 (dt, *J* = 16.0, 5.8 Hz, 1H), 5.80 (d, *J* = 1.8 Hz, 1H), 4.89 (dd, *J* = 18.4, 1.8 Hz, 1H), 4.79 (dd, *J* = 18.4, 1.8 Hz, 1H), 4.74 (d, *J* = 1.7 Hz, 1H), 4.19 (dd, *J* = 10.3, 4.4 Hz, 1H), 4.13 (ddd, *J* = 13.2, 5.6, 1.5 Hz, 1H), 4.11 – 4.04 (m, 1H), 4.07 (ddd, *J* = 13.2, 6.0, 1.5 Hz, 1H), 4.04 – 4.00 (m, 1H), 3.67 (dd, *J* = 3.4, 1.7 Hz, 1H), 3.64 (dq, *J* = 9.5, 6.4 Hz, 1H), 3.60 (dd, *J* = 9.5, 3.4 Hz, 1H), 3.38 (s, 1H), 3.27 (t, *J* = 9.5 Hz, 1H), 2.80 (t, *J* = 7.2 Hz, 1H), 2.16 (dt, *J* = 14.8, 3.3 Hz, 1H), 2.11 – 2.00 (m, 3H), 2.00 – 1.83 (m, 3H), 1.83 – 1.73 (m, 1H), 1.69 – 1.55 (m, 4H), 1.47 (dd, *J* = 11.9, 9.6 Hz, 1H), 1.38 (dd, *J* = 13.2, 11.2 Hz, 1H), 1.33 – 1.24 (m, 1H), 1.24 – 1.17 (m, 2H), 1.15 (d, *J* = 6.2 Hz, 3H), and 0.80 (s, 3H). **^{13}C NMR (CD_3OD, 101 MHz)** δ: 177.5, 177.1, 138.8, 133.2, 132.3, 129.1, 127.5, 122.8, 118.0, 99.3, 85.6, 84.4, 79.2, 75.9, 75.3, 74.2, 73.1, 72.6, 72.4, 71.8, 70.5, 69.9, 69.0, 51.6, 50.9, 50.4, 49.3, 41.2, 33.4, 27.9, 24.5, 18.0, and 17.5. **HRMS (DART-TOF)** calculated for $C_{40}H_{52}NaO_{12}^{+}$[M+Na]$^{+}$ m/z 747.3351, found 747.3365.

Procedures and Characterization Data for Compounds in Figure 6

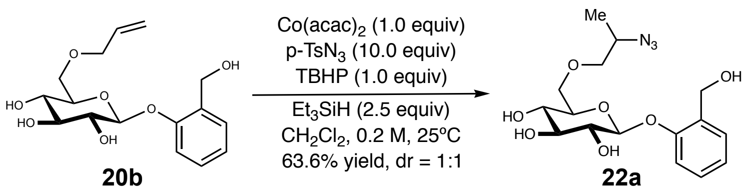

In a N_2-filled glovebox, a 10-mL screw capped vial with a stir bar was charged sequentially with **20b** (65.2 mg, 0.2 mmol, 1.0 equiv), TsN_3 (394.0 mg, Energy Chemicals Lot RN6RRN4R, 2.0 mmol, 10.0 equiv), and cobalt bis(acetylacetonate) (71.2 mg, Energy Chemicals Lot FJ270144, 0.2 mmol, 1.0 equiv). Dichloromethane (1.0 mL), triethylsilane (58 mg, Energy Chemicals Lot FR3RRH2K, 0.5 mmol, 2.5 equiv), and a solution of *tert*-butyl hydroperoxide in decane (5.5 M in decane, Energy Chemicals Lot FA050166, 36 μL, 0.2 mmol, 1.00 equiv) were then added in sequence via syringe. The reaction mixture was stirred for 19 h at 24 °C (400 rpm). The solution was concentrated in vacuo. Flash chromatography (SiO_2) using CH_2Cl_2/MeOH (40:1 to 20:1) as eluent afforded **22a** [53.5 mg (which contains 16.7% hydrogenated product), 0.127 mmol, 63.6%] as a 1:1 mixture of diastereomers as a colorless oil.

^{1}H NMR (MeOD, 400 MHz) δ 7.34 (app dd, *J* = 7.5, 1.6 Hz, 1H), 7.24 (app td, *J* = 7.7, 7.3, 1.7 Hz, 1H), 7.21 – 7.16 (m, 1H), 7.03 (app td, *J* = 7.3, 1.3 Hz, 1H), 4.78 (app d, *J* = 13.0 Hz, 1H), 4.57 (d, *J* = 13.0 Hz, 1H), 3.86 (app dt, *J* = 11.1, 1.8 Hz, 1H), 3.69 – 3.59 (m, 2H), 3.54 (m, 2H), 3.47 (m, 3H), 3.41 – 3.33 (m, 1H), 1.13 (app dd, *J* = 6.7, 2.6 Hz, 3H). **^{13}C NMR (MeOD, 101 MHz)** δ 157.0, 156.9, 132.2, 132.2, 129.9, 129.9, 129.8, 123.8, 117.1, 117.0, 103.2, 103.2, 78.0, 77.3, 77.3, 76.5, 76.4, 75.0, 74.9, 71.8, 71.6, 71.5, 71.4, 61.0, 58.4, 58.3, 16.3, and 16.3. **IR (thin film, cm-1)**:3360, 2961, 2875, 2103, 1604, 1590, 1492, 1455, 1236, 1068, and 756. **HRMS (DART-TOF)** calculated for $C_{16}H_{23}N_3NaO_7^+$ $[M+Na]^+$ m/z 392.1428, found 392.1428.

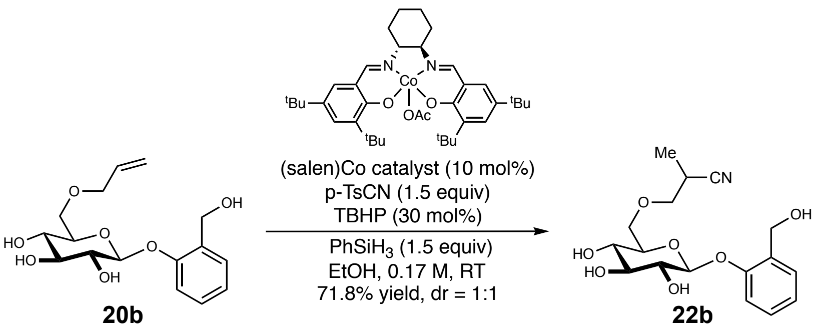

The (salen)Co catalyst[9] (13.2 mg, 0.02 mmol, 0.1 equiv) was dissolved in anhydrous EtOH (800 μL). After 2 min, allylated salicin **20b** (65.2 mg, 0.2 mmol, 1.0 equiv) was added followed by TsCN (57.6 mg, SC-Shuyan Lot SY001647, 0.3 mmol, 1.5 equiv). TBHP (5.5 M solution in decane, Energy Chemicals Lot FA050166, 10 μL, 0.06 mmol, 0.3 equiv) was added to the solution followed by $PhSiH_3$ (32.4 mg, Energy Chemicals Lot RK0RRN7N, 0.3 mmol, 1.5 equiv) and another portion of EtOH (400 μL). The resulting pale yellow, homogeneous solution was stirred at RT for 12 h (400 rpm). After completion the solvent was evaporated and the crude mixture purified by flash chromatography (SiO_2) using CH_2Cl_2/MeOH (40:1 to 20:1) as eluent afforded **22b** [60.1 mg (which contains 24% hydrogenated product), 0.144 mmol, 71.8%, dr = 1:1] as a colorless oil.

^{1}H NMR (MeOD, 400 MHz) δ 7.34 (app dd, *J* = 7.5, 1.6 Hz, 1H), 7.26 (app td, *J* = 7.7, 1.7 Hz, 1H), 7.19 (app dt, *J* = 8.4, 1.6 Hz, 1H), 7.03 (app td, *J* = 7.4, 1.2 Hz, 1H), 4.88 (app d, *J* = 1.7 Hz, 1H), 4.78 (d, *J* = 13.0 Hz, 1H), 4.57 (d, *J* = 13.0 Hz, 1H), 3.88 (app dd, *J* = 11.3, 1.8 Hz, 1H), 3.68 (app ddd, *J* = 11.2, 6.2, 2.7 Hz, 1H), 3.62 – 3.54 (m, 3H), 3.53 – 3.48 (m, 1H), 3.45 (t, *J* = 8.8 Hz, 1H), 3.37 (t, *J* = 8.8 Hz, 1H), 2.96 (hept, *J* = 7.0 Hz, 1H), 1.24 (app dd, *J* = 7.2, 3.7 Hz, 3H). **^{13}C NMR (MeOD,101 MHz)** δ 156.9, 132.2, 132.2, 130.0, 130.0, 129.8, 123.8, 123.8, 122.9, 122.9, 117.1, 117.0, 103.2, 103.1, 78.0, 77.4, 77.3, 75.0, 73.4, 73.3, 71.7, 71.7, 71.5, 60.9, 27.6, 14.9, and 14.8. **IR (thin film, cm-1)**:3378, 2922, 2873, 1604, 1492, 1455, 1235, 1062, 1011, and 760. **HRMS (DART-TOF)** calculated for $C_{17}H_{23}NNaO_7^+$ [M+Na]$^+$ m/z 376.1367, found 376.1366.

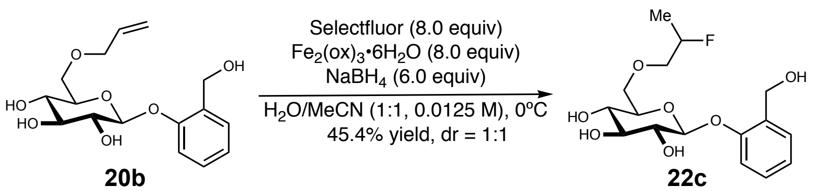

To a 25 mL round bottom flask (RBF) placed in a N_2-filled glovebox, $Fe_2(ox)_3$• $6H_2O$ (774.4 mg, Alfa Aesar Lot T26E001, 1.6 mmol, 8.0 equiv), H_2O (8 mL), and a stir bar were added in sequence. The resulting mixture was stirred to homogeneity (ca. 2 h). The clear yellow solution was then cooled to 0 °C. Selectfluor (566.4 mg, Energy Chemicals Lot GC200313, 1.6 mmol, 8.0 equiv) and MeCN (4.0 mL) were sequentially added to the reaction mixture. Then, **20b** (65.2 mg, 0.2 mmol, 1.0 equiv) and MeCN (4.0 mL) was added to the reaction mixture. The RBF was taken out of the glovebox and cooled at 0 °C. $NaBH_4$ (7.6 mg, 0.2 mmol, 1.0 equiv) was added to the mixture under N_2 atmosphere at 0 °C. After 60 min, the reaction mixture was treated with an additional portion of $NaBH_4$ (7.6 mg). The resulting mixture was stirred for additional 60 min. The addition of $NaBH_4$ was repeated for 6 times. Then the reaction mixture was quenched by addition of 28–30% aqueous ammonia (2.0 mL). The mixture was extracted with 10% MeOH in CH_2Cl_2 and the organic layer was dried over Na_2SO_4 and concentrated under reduced pressure. Flash chromatography (SiO_2) using CH_2Cl_2/MeOH (40:1 to 20:1) as eluent afforded **22c** [38.9 mg (which contains 20% hydrogenated product), 0.091 mmol, 45.4%] as a 1:1 mixture of diastereomers as a colorless oil.

^{1}H NMR (MeOD, 400 MHz) δ 7.34 (app dd, J = 7.6, 1.6 Hz, 1H), 7.24 (app td, J = 7.8, 7.1, 1.6 Hz, 1H), 7.18 (app dd, J = 8.3, 1.5 Hz, 1H), 7.03 (app td, J = 7.4, 1.3 Hz, 1H), 4.86 (app s, 1H, overlap), 4.77 (dd, J = 12.9, 2.8 Hz, 1H), 4.58 (d, J = 13.0 Hz, 1H), 3.88 (app ddd, J = 11.2, 4.0, 1.9 Hz, 1H), 3.67 (app ddd, J = 11.2, 6.2, 2.5 Hz, 1H), 3.62 –3.58 (m, 1H), 3.58 – 3.41 (m, 4H), 3.37 (app td, J = 9.1, 4.1 Hz, 1H), 1.28 (app dd, J = 6.4, 3.4 Hz, 1H), 1.22 (app dd, J = 6.4, 3.3 Hz, 1H). **^{13}C NMR (MeOD, 101 MHz)** δ 157.0, 132.2, 129.9, 129.9, 129.8, 123.8, 123.8, 117.1, 117.1, 103.2, 103.2, 90.6 (d, J = 167.0 Hz), 90.5 (d, J = 167.0 Hz), 78.0, 77.3, 77.2, 75.9, 75.8, 75.7, 75.6, 75.0, 71.8 (d, J = 30.0 Hz), 71.7 (d, J = 30.0 Hz), 61.0, 17.6 (d, J = 22.0 Hz), 17.5 (d, J = 22.0 Hz). **^{19}F NMR (MeOD, 376 MHz)** δ -180.71, and -180.75. **IR (thin film, cm-1)**:3351, 2922, 2875, 2485, 1604, 1590, 1492, 1455, 1235, 1063, and 758. **HRMS (DART-TOF)** calculated for $C_{16}H_{23}FNaO_7^+$ $[M+Na]^+$ m/z 369.1320, found 369.1321.

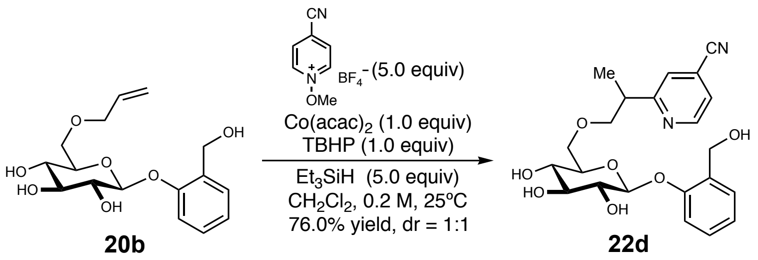

In a N_2-filled glovebox, a 10-mL screw capped vial with a stir bar was charged sequentially with **20b** (48.9 mg, 0.15 mmol, 1.0 equiv), *N*-methoxy 4-cyanopyridinium tetrafluoroborate[10] (166.5 mg, 0.75 mmol, 5.0 equiv), and cobalt bis(acetylacetonate) (53.4 mg, 0.15 mmol, 1.0 equiv). Dichloromethane (750 μL), triethylsilane (87 mg, 0.75 mmol, 5.0 equiv), and a solution of *tert*-butyl hydroperoxide in decane (5.5 M in decane, 27 μL, 0.15 mmol, 1.0 equiv) were then added in sequence via syringe. The reaction vessel was protected from light with aluminum foil. The reaction mixture was stirred for 16 h at 25 °C. The solution was concentrated in vacuo. Flash chromatography (SiO_2) using CH_2Cl_2/MeOH (40:1 to 20:1) as eluent afforded **22d** [56.5 mg (which contains 16.7% hydrogenated product), 0.114 mmol, 76.0%] as a 1:1 mixture of diastereomers as a light yellow oil.

^{1}H NMR (MeOD, 400 MHz) δ 8.65 (app d, *J* = 5.1 Hz, 1H), 7.64 – 7.56 (m, 1H), 7.47 (app ddd, *J* = 6.4, 5.0, 1.5 Hz, 1H), 7.34 (app dq, *J* = 7.6, 1.7 Hz, 1H), 7.24 – 7.17 (m, 1H), 7.08 (app dd, *J* = 8.1, 2.9 Hz, 1H), 7.03 (app td, *J* = 7.4, 2.8 Hz, 1H), 4.84 (m, 1H, overlap), 4.77 (d, *J* = 13.4 Hz, 1H), 4.57 (dt, *J* = 13.0, 4.0 Hz, 1H), 3.82 – 3.72 (m, 2H), 3.68 (app ddd, *J* = 9.6, 7.8, 5.9 Hz, 1H), 3.60 (app td, *J* = 11.1, 6.2 Hz, 1H), 3.49 – 3.40 (m, 3H), 3.28 – 3.23 (m, 1H), 1.26 (app dd, *J* = 7.0, 3.1 Hz, 3H). **^{13}C NMR (MeOD, 101 MHz)** δ 166.8, 166.8, 157.0, 151.1, 151.0, 132.3, 132.3, 129.9, 129.9, 125.8, 125.8, 124.5, 124.5, 123.8, 122.1, 117.7, 117.7, 117.0, 117.0, 103.3, 103.2, 77.9, 77.2, 77.1, 76.6, 76.4, 75.0, 75.0, 71.5, 71.5, 61.0, 43.2, 17.3, and 17.2. **IR (thin film, cm-1)**:3371, 2922, 2875, 1602, 1551, 1492, 1455, 1235, 1064, and 785. **HRMS (DART-TOF)** calculated for $C_{22}H_{26}N_2NaO_7^+$ $[M+Na]^+$ m/z 453.1632, found 453.1635.

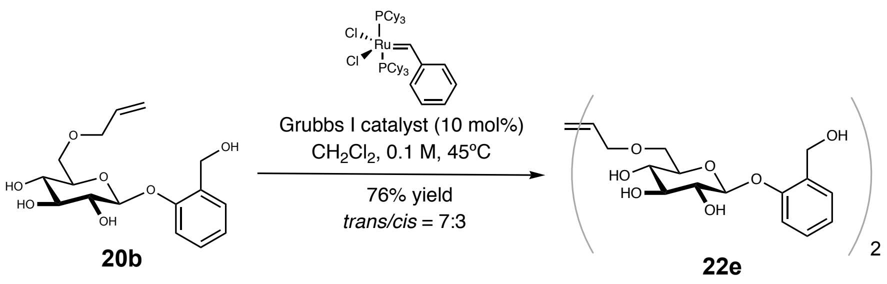

In a N_2-filled glovebox, a screw capped vial with a stir bar was charged sequentially with **20b** (65.2 mg, 0.2 mmol, 1.0 equiv), Grubbs I catalyst (16.7 mg, Energy Chemicals Lot RNR3RHD4, 2.0 mmol, 10 mol%), and dichloromethane (1.0 mL). The mixture was stirred at 45 °C for 16 h. The reaction mixture was then concentrated in vacuo. Flash chromatography (SiO_2) using CH_2Cl_2/MeOH (20:1 to 8:1) as eluent afforded the mixture **22e** (*trans/cis* 7:3) as a colorless oil (47.3 mg, 0.076 mmol, 76%), and recovered starting material **20b** (6.4 mg, 19.6%).

^{1}H NMR (MeOD, 400 MHz) δ 7.33 (app dt, *J* = 7.6, 2.0 Hz, 2H), 7.24 (app td, *J* = 7.8, 1.7 Hz, 2H), 7.20 – 7.13 (m, 2H), 7.02 (app td, *J* = 7.4, 1.2 Hz, 2H), 5.77 (dd, *J* = 3.7, 2.1 Hz, 1.4 H, *cis*), 5.66 (t, *J* = 4.0 Hz, 0.6 H, *trans*), 4.85 (app br s, 2H, overlap), 4.76 (d, *J* = 12.9 Hz, 2H), 4.57 (dd, *J* = 13.0, 1.8 Hz, 2H), 4.09 – 3.99 (m, 4H), 3.79 (dd, *J* = 11.0, 1.9 Hz, 1.54 H), 3.74 (dd, *J* = 11.1, 1.9 Hz, 0.66H), 3.65 – 3.56 (m, 2H), 3.56 – 3.42 (m, 6H), 3.42 – 3.35 (m, 2H). **^{13}C NMR (MeOD, 101 MHz)** δ 157.0, 132.2, 130.4, 130.4, 130.0, 130.0, 129.9, 129.9, 123.8, 117.1, 117.0, 103.2, 103.2, 78.0, 77.9, 77.1, 77.1, 75.0, 74.9, 72.3, 71.5, 71.4, 70.5, 70.4, 68.1, and 61.0. **IR (thin film, cm-1)**:3349, 2911, 2873, 2486, 1604, 1590, 1492, 1455, 1369, 1234, 1062, 1013, and 758. **HRMS (DART-TOF)** calculated for $C_{30}H_{40}NaO_{14}^+$ $[M+Na]^+$ m/z 647.2310, found 647.2311.

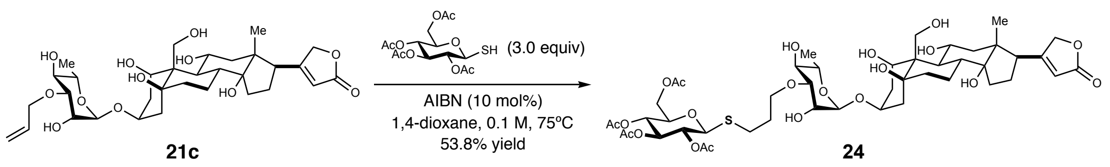

In a N_2-filled glovebox, a screw capped vial with a stir bar was charged with **21c** (62.4 mg, 0.1 mmol, 1.0 equiv), and tetraacetyl thioglucose[11] (109mg, 0.3 mmol, 3 equiv). AIBN (1.6 mg, Adamas beta Lot P1180693, 0.01 mmol, 10 mol%) was added, followed by anhydrous 1,4-dioxane (1.0 mL). The mixture was then heated for 12 h at 75 °C. The solution was concentrated in vacuo. Flash chromatography (SiO_2) using CH_2Cl_2/MeOH (20:1 to 8:1) as eluent afforded **24** as a white foam (53.2 mg, 0.0538 mmol, 53.8%).

^{1}H NMR (MeOD, 400 MHz) δ 5.92 (d, J = 1.9 Hz, 1H), 5.28 (t, J = 9.4 Hz, 1H), 5.04 (d, J = 18.9 Hz, 1H), 5.02 (t, J = 9.6 Hz, 1H), 4.94 (t, J = 9.7 Hz, 1H), 4.93 (dd, J = 18.4, 1.7 Hz, 1H), 4.85 (d, J = 1.9 Hz, 1H), 4.73 (d, J = 10.1 Hz, 1H), 4.25 (dd, J = 12.4, 5.0 Hz, 1H), 4.19 (dd, J = 10.3, 4.4 Hz, 1H), 4.14 – 4.06 (m, 3H), 3.89 (ddd, J = 10.1, 5.0, 2.4 Hz, 1H), 3.80 – 3.70 (m, 2H), 3.70 (dd, J = 9.4, 3.4 Hz, 1H), 3.60 – 3.51 (m, 2H), 3.37 (t, J = 9.5 Hz, 1H), 2.93 (t, J = 7.2 Hz, 1H), 2.88 – 2.73 (m, 2H), 2.25 (dt, J = 15.6, 3.7 Hz, 1H), 2.20 – 2.12 (m, 3H), 2.06 (s, 3H), 2.02 (s, 3H), 2.01 (s, 3H), 1.97 (s, 3H), 1.96 – 1.82 (m, 4H), 1.80 – 1.67 (m, 4H), 1.61 –1.45 (m, 2H), 1.43 –1.34 (m, 1H), 1.31 –1.19 (m, 1H), 1.25 (d, J = 6.2 Hz, 3H), and 0.97 (s, 3H). **^{13}C NMR (MeOD, 101 MHz)** δ 177.6, 177.1, 172.3, 171.6, 171.3, 171.1, 118.1, 99.1, 85.6, 84.8, 76.7, 75.8, 75.3, 75.18, 74.2, 72.5, 72.3, 71.7, 71.4, 70.9, 70.9, 69.9, 69.8, 69.1, 63.4, 51.6, 51.0, 50.5, 41.3, 41.2, 33.4, 31.5, 28.5, 27.9, 24.5, 20.8, 20.7, 20.6, 18.0, and 17.9. **IR (thin film, cm-1)**:3401, 2935, 2881, 2520, 1733, 1623, 1492, 1434, 1368, 1219, 1035, and 909. **HRMS (DART-TOF)** calculated for $C_{46}H_{68}NaO_{21}S^+$ $[M+Na]^+$ m/z 1011.3866, found 1011.3874.

CDCl3, 400.13 MHz

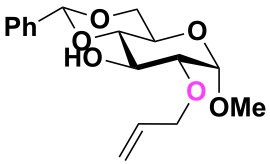

9a

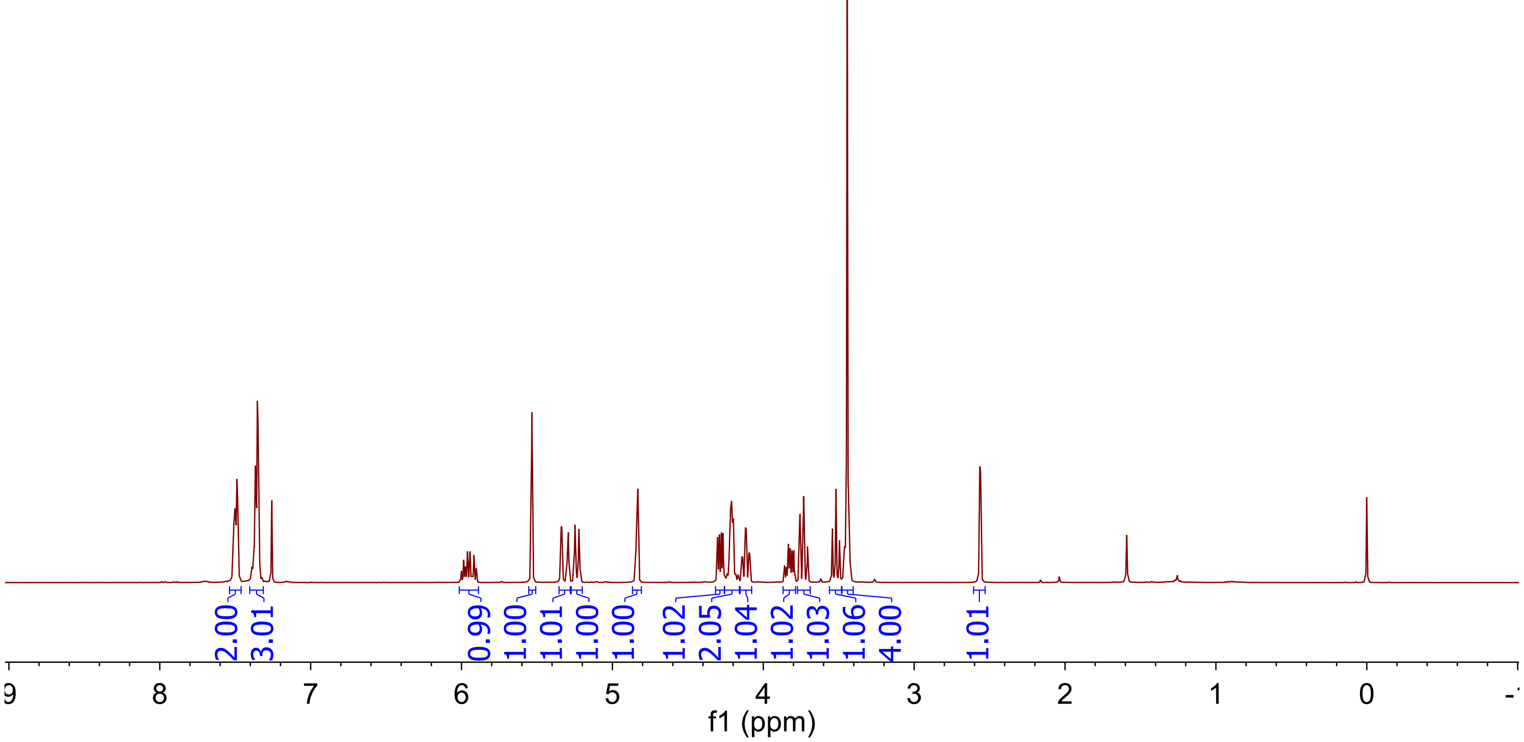

CDCl3, 100.62 MHz

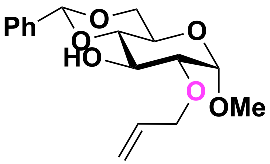

9a

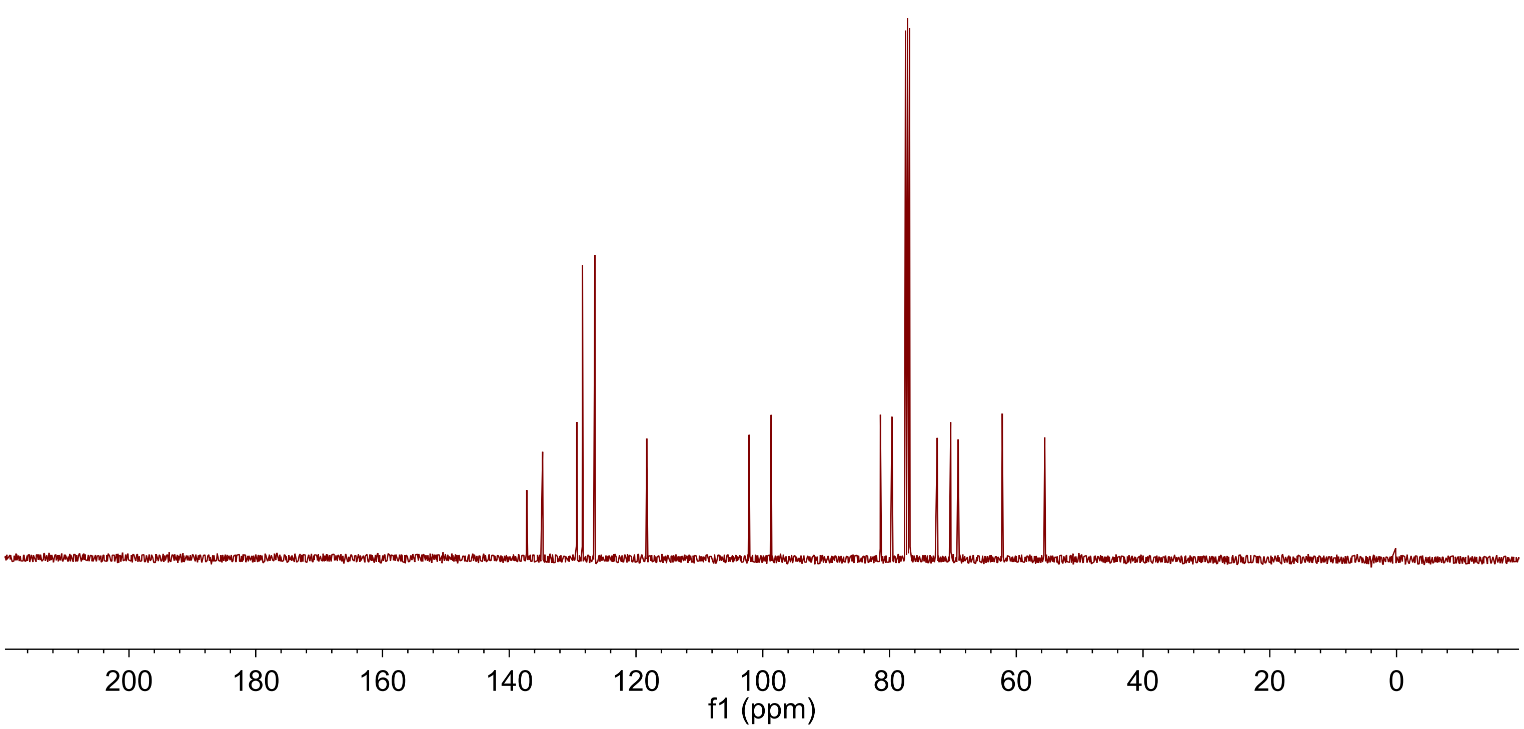

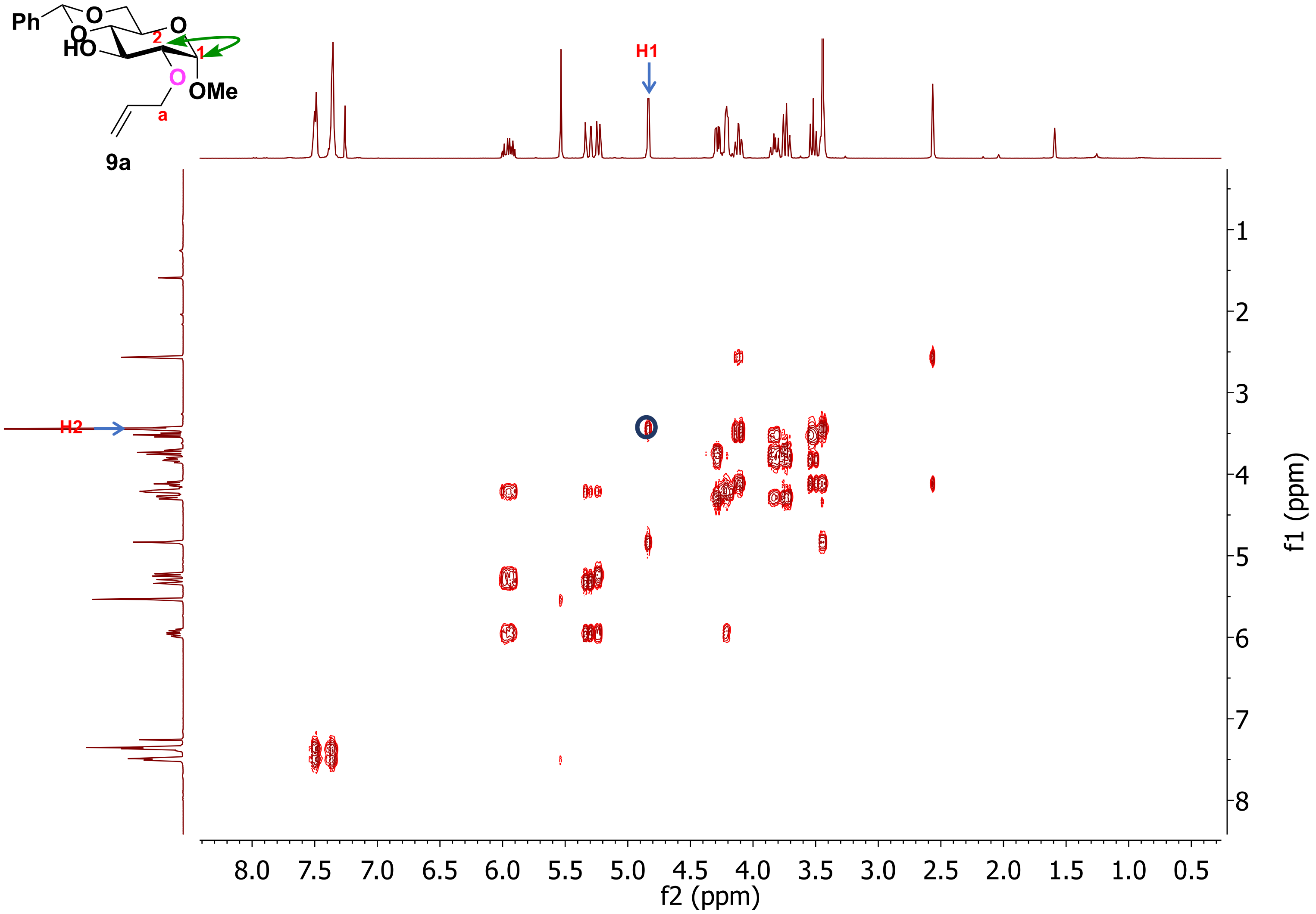
Ph
O
O
O
HO
2
1
O
OMe
a
9a
H1
H2
8.0 7.5 7.0 6.5 6.0 5.5 5.0 4.5 4.0 3.5 3.0 2.5 2.0 1.5 1.0 0.5
f2 (ppm)
1 2 3 4 5 6 7 8
f1 (ppm)

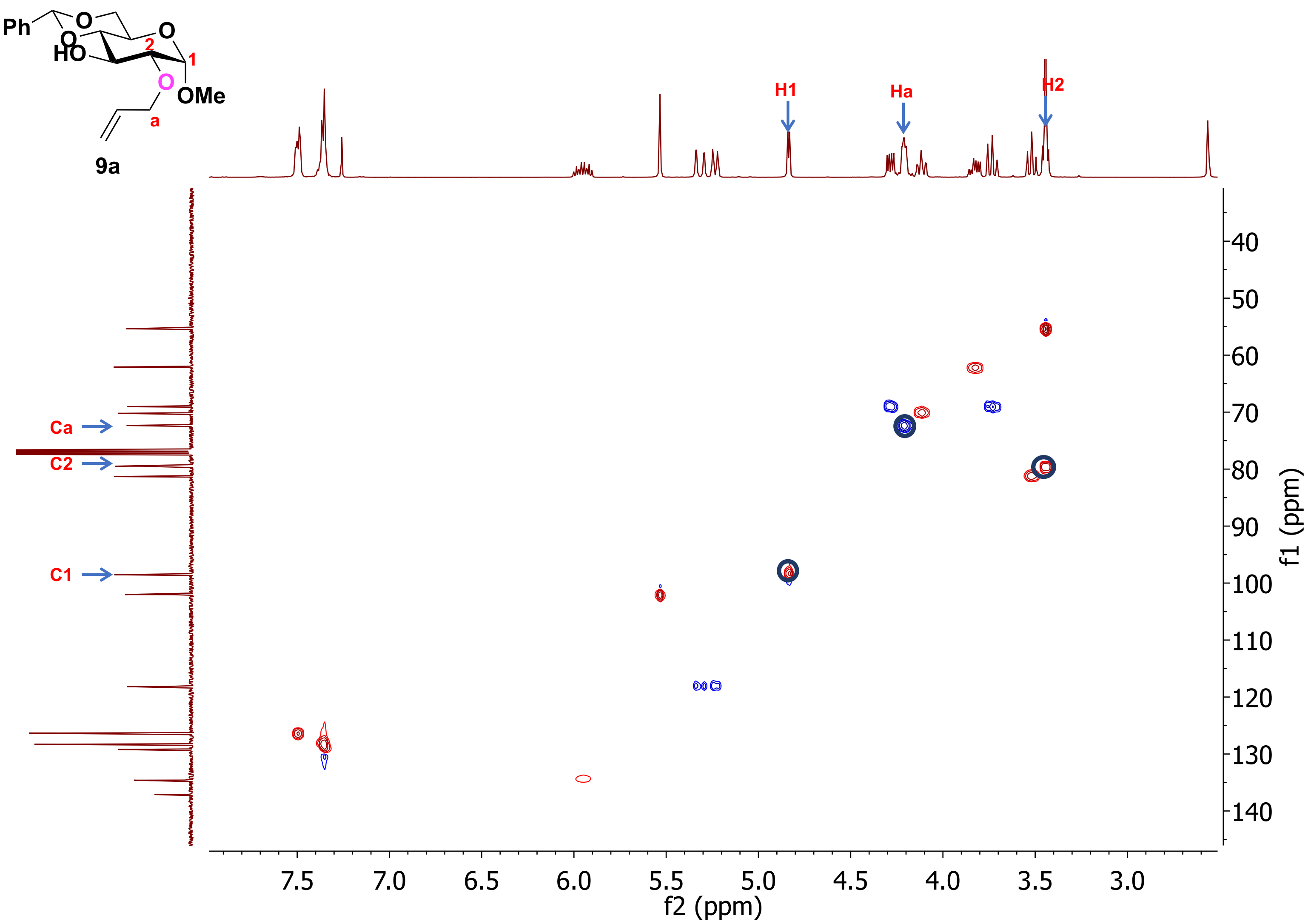
Ph
O
O
O
HO
2
1
O
OMe
a
9a
H1
Ha
H2
Ca
C2
C1
40
50
60
70
80
90
100
110
120
130
140
f1 (ppm)
7.5
7.0
6.5
6.0
5.5
5.0
4.5
4.0
3.5
3.0
f2 (ppm)

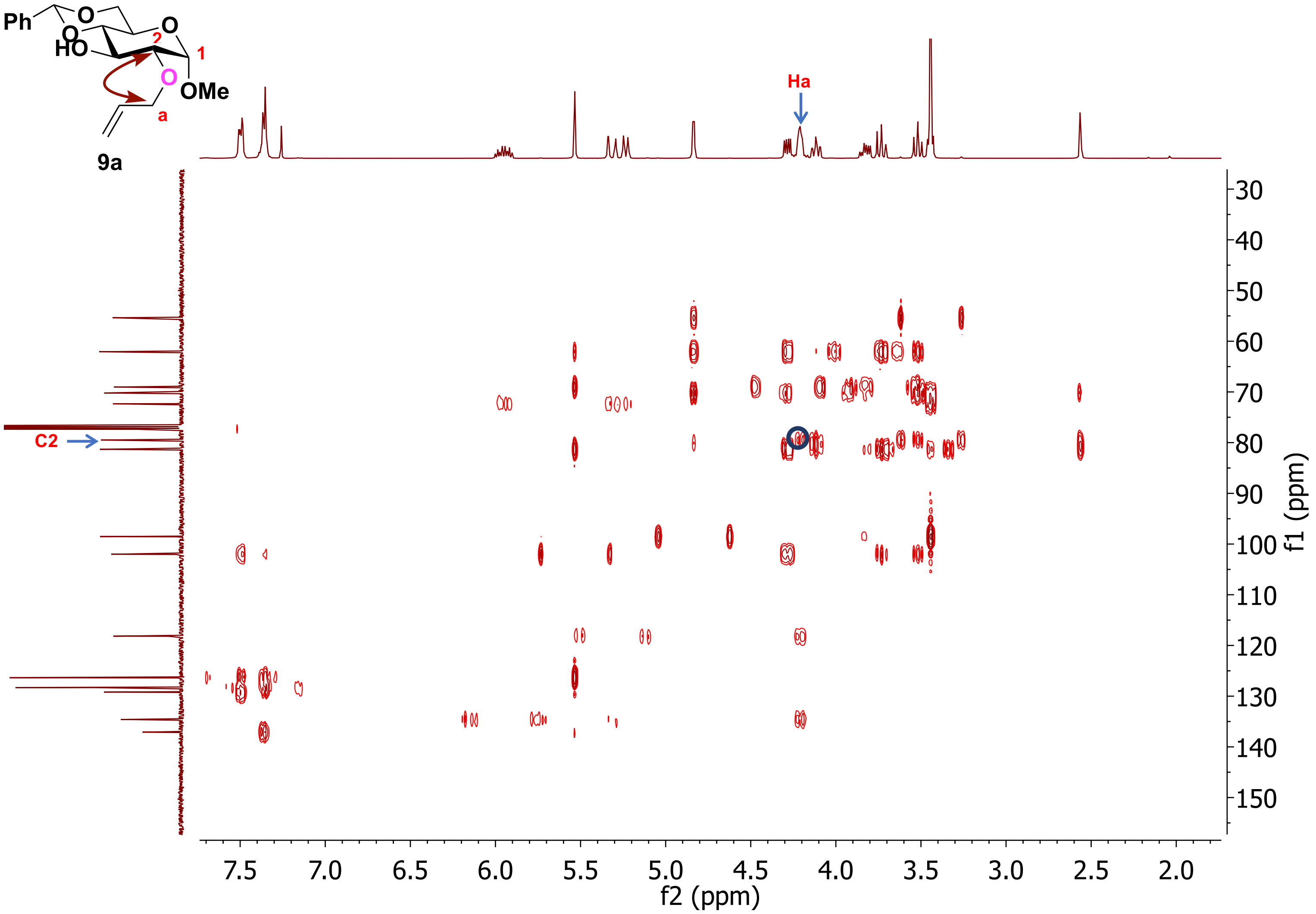

Ph
O
O
O
HO
2
1
O
OMe
a
9a
Ha
C2
30
40
50
60
70
80
90
100
110
120
130
140
150
f1 (ppm)
7.5
7.0
6.5
6.0
5.5
5.0
4.5
4.0
3.5
3.0
2.5
2.0
f2 (ppm)

CDCl3, 400 MHz

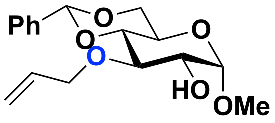

9b

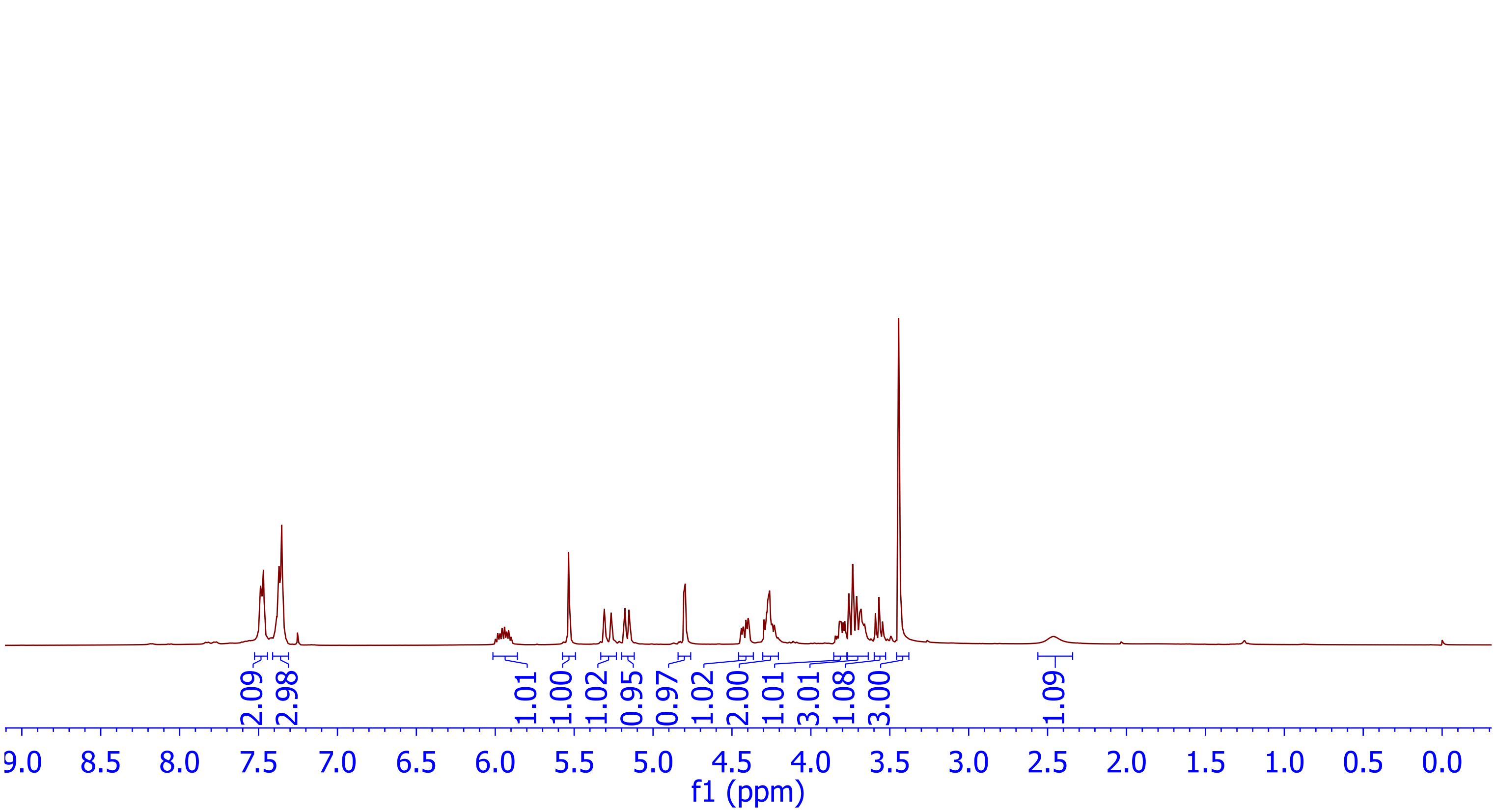

CDCl3, 101 MHz

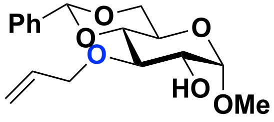

9b

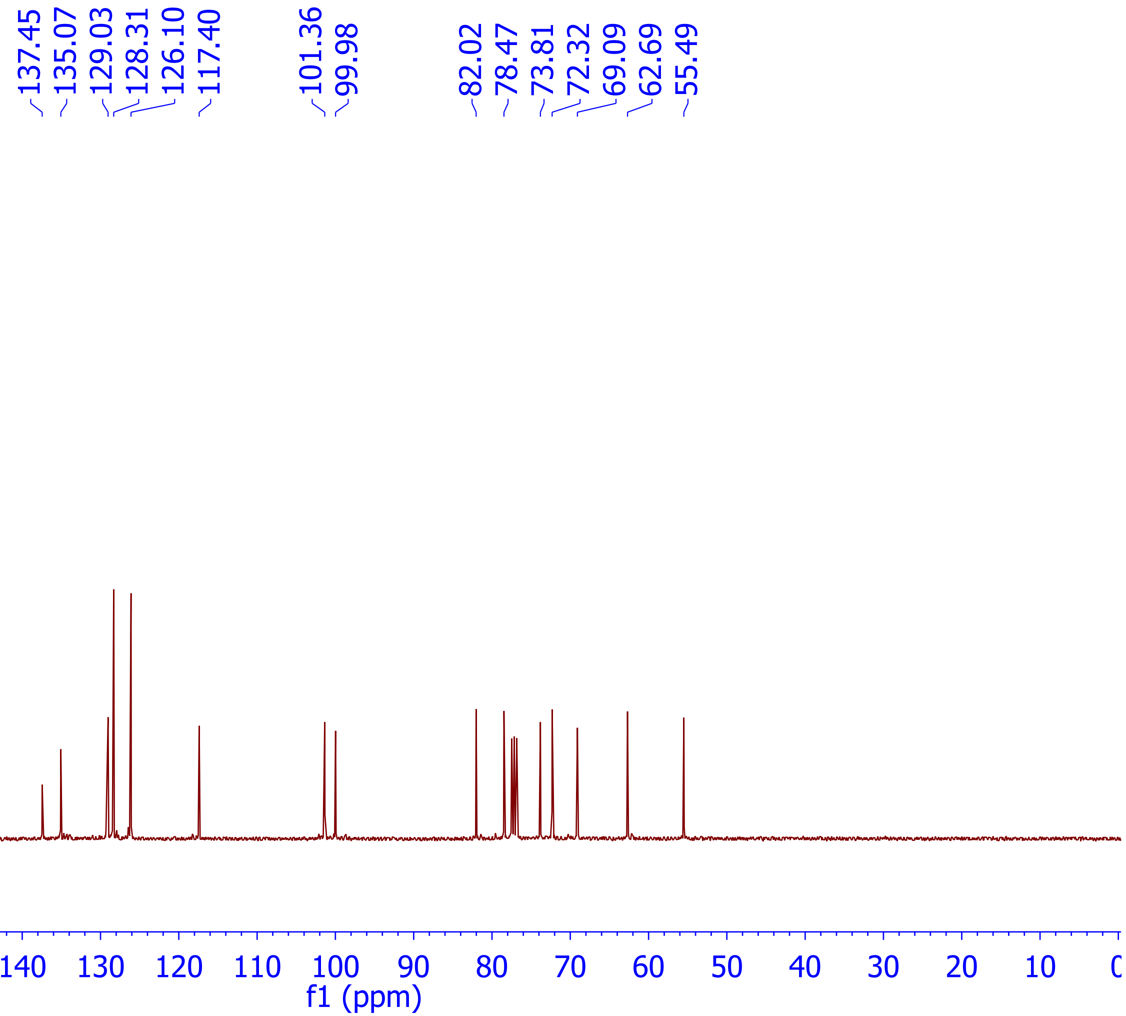

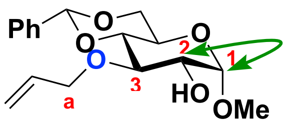

Ph
O
O
O
O
2
1
3
HO
OMe
a

9b

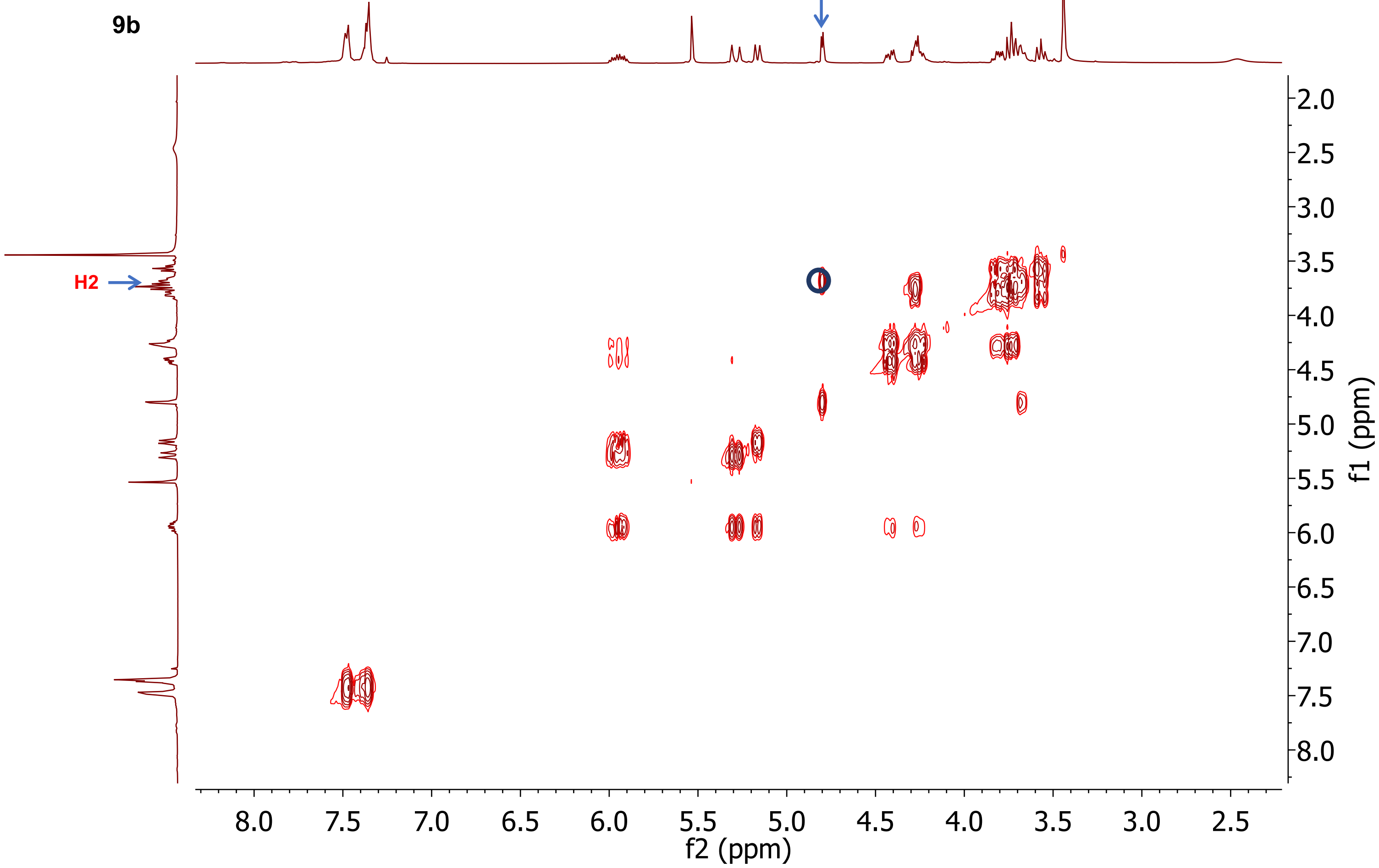

H1
H2
f2 (ppm)
f1 (ppm)
8.0
7.5
7.0
6.5
6.0
5.5
5.0
4.5
4.0
3.5
3.0
2.5
2.0

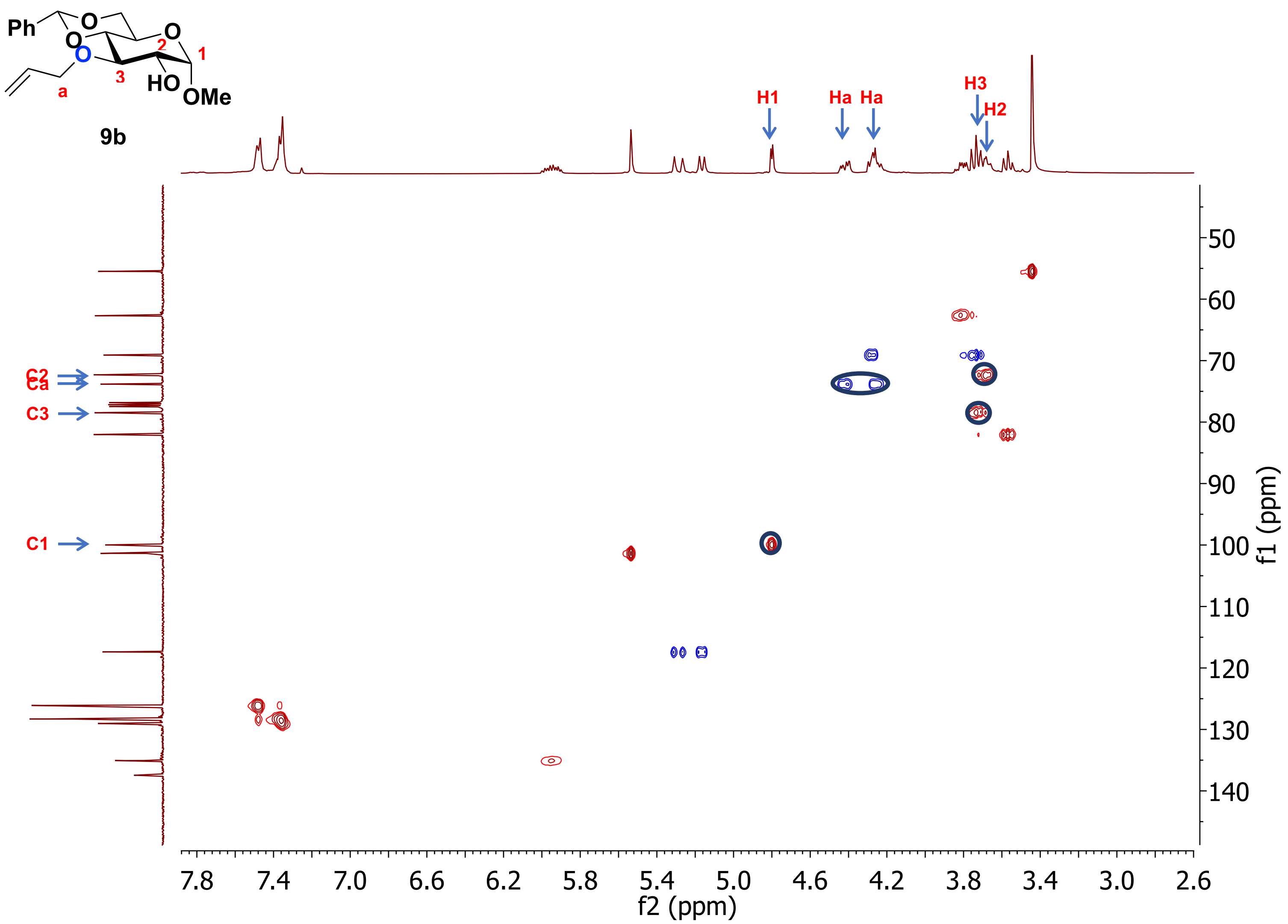
Ph
O
O
O
O
1
2
3
HO
OMe
a
9b
H1
Ha
Ha
H3
H2
C2
Ca
C3
C1
f2 (ppm)
f1 (ppm)

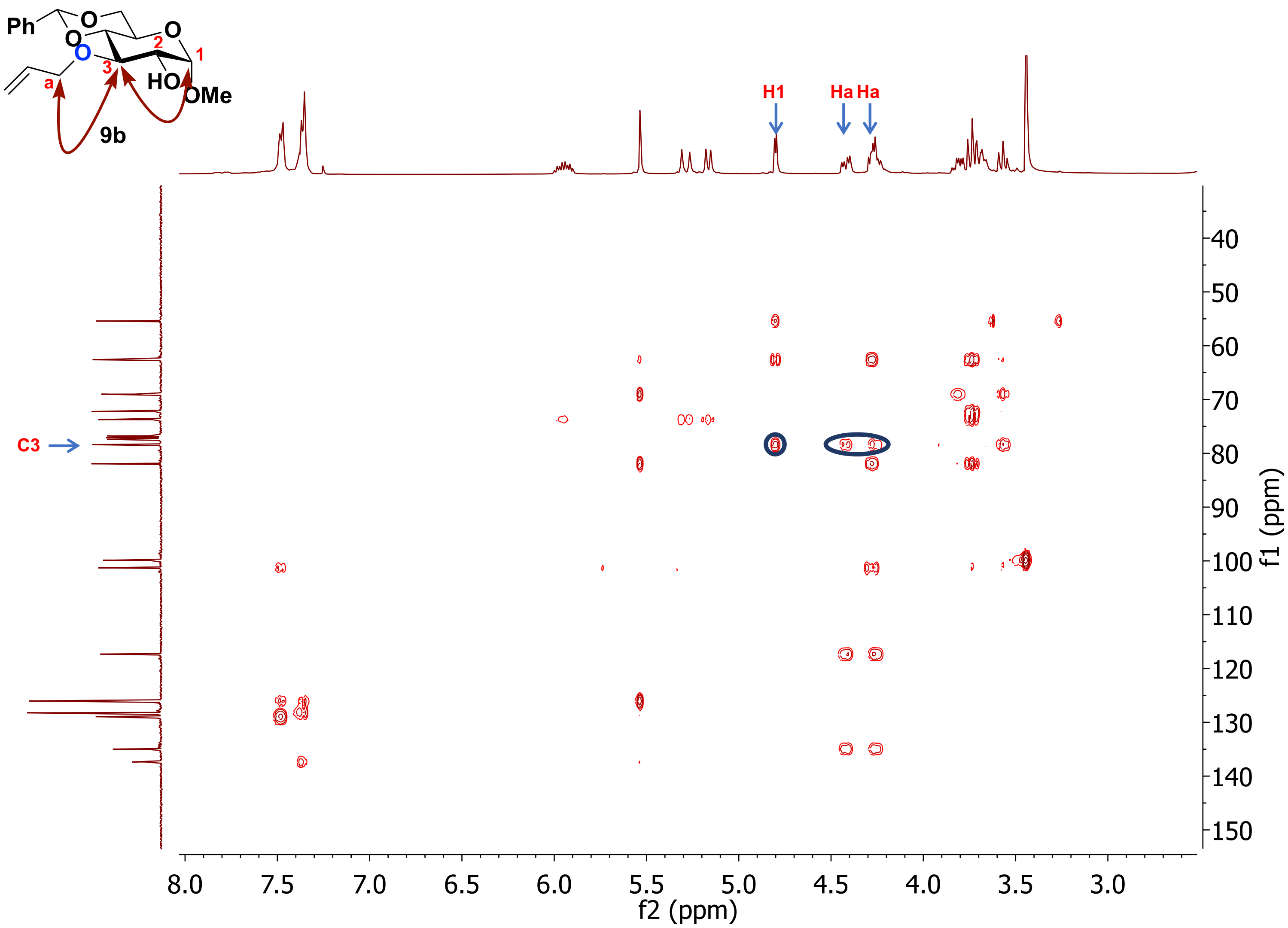
Ph
O
O
O
O
2
1
3
a
HO
OMe
9b
H1
Ha Ha
C3
8.0
7.5
7.0
6.5
6.0
5.5
5.0
4.5
4.0
3.5
3.0
f2 (ppm)
40
50
60
70
80
90
100
110
120
130
140
150
f1 (ppm)

CDCl3, 400.13 MHz

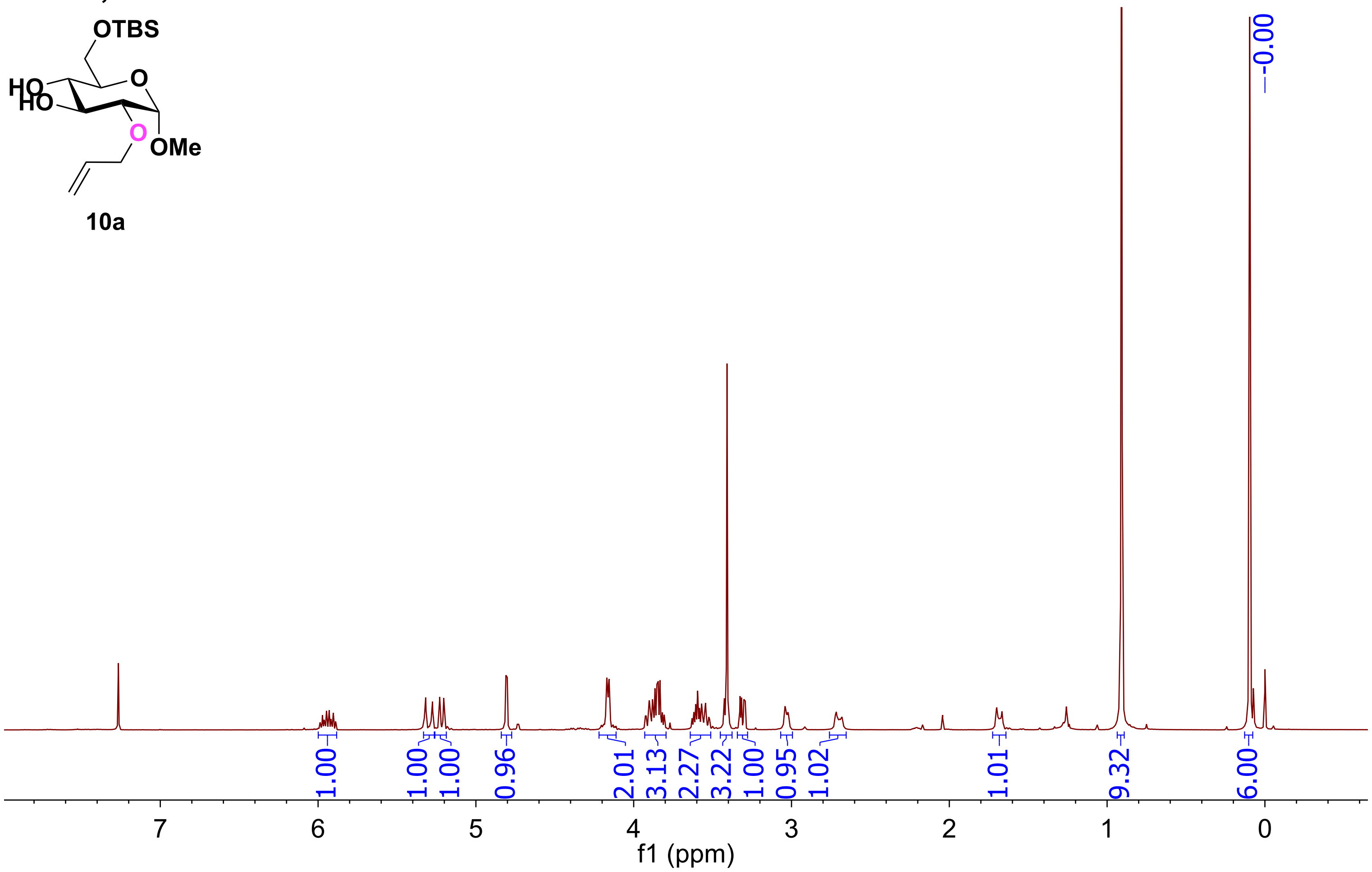

CDCl3, 100.62 MHz

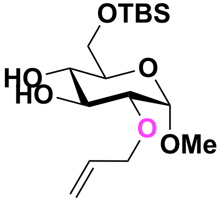

10a

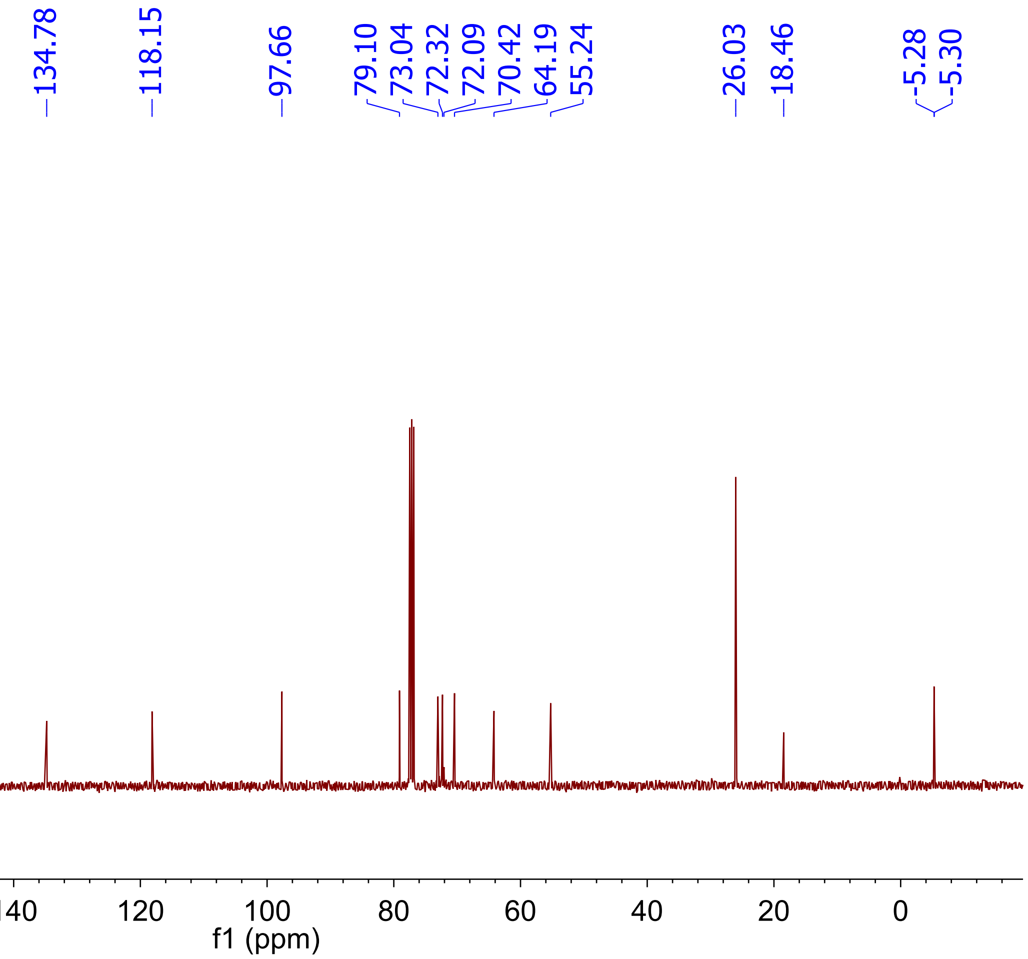

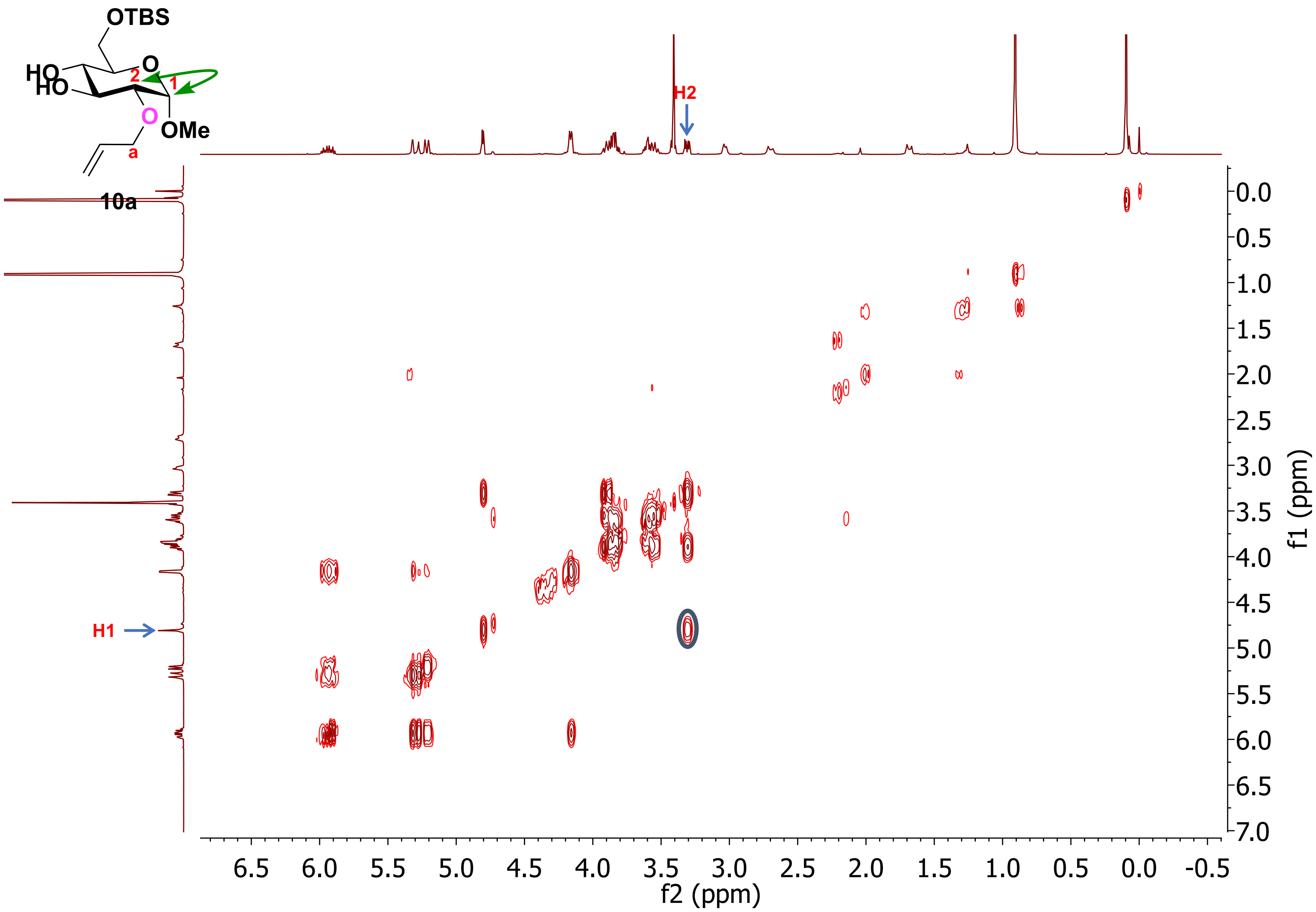
OTBS
HO
HO
O
2
1
O
OMe
a
10a
H2
H1
f2 (ppm)
f1 (ppm)

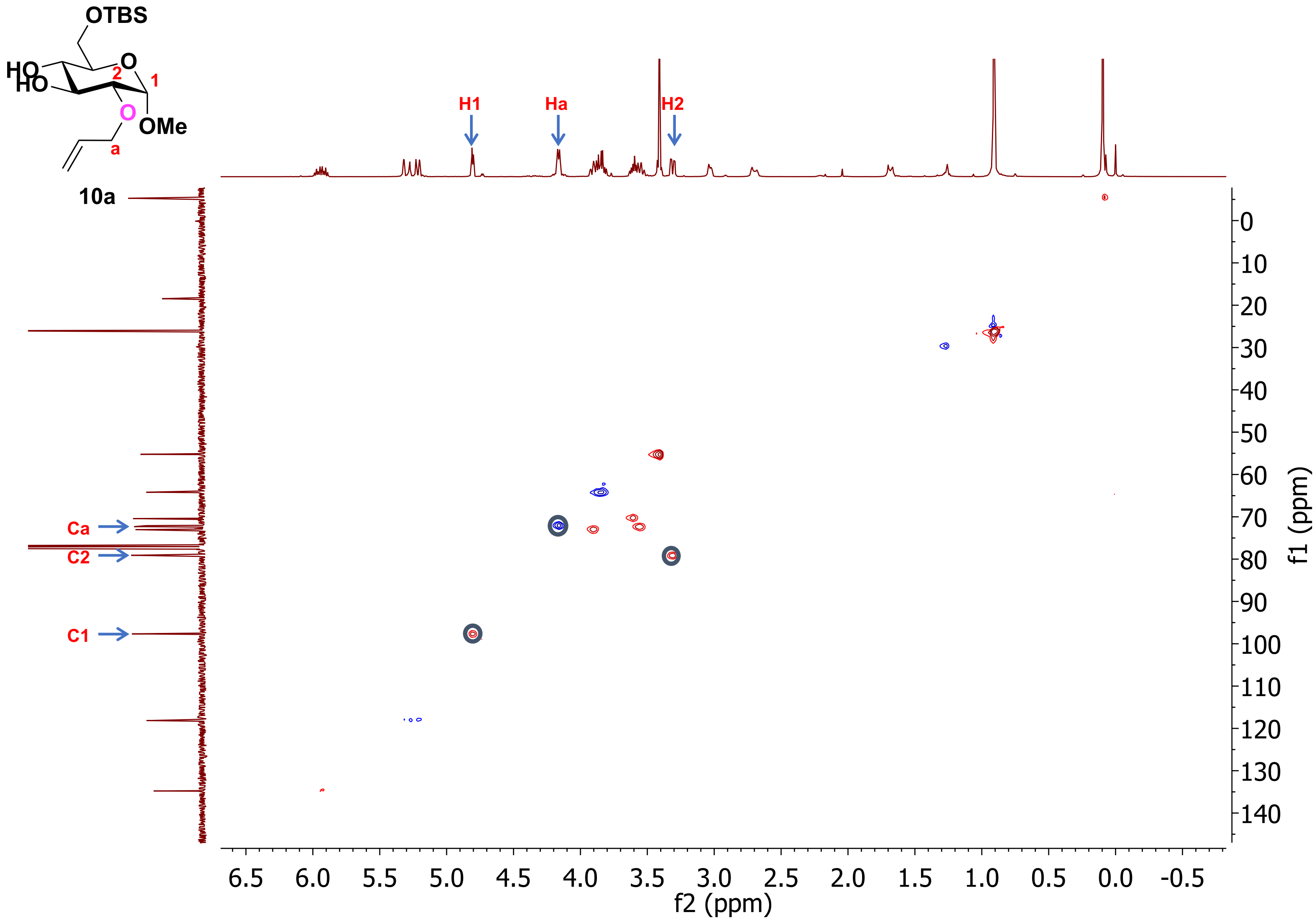
OTBS
HO
HO
O
2
1
O
OMe
a
10a
H1
Ha
H2
Ca
C2
C1
f2 (ppm)
f1 (ppm)

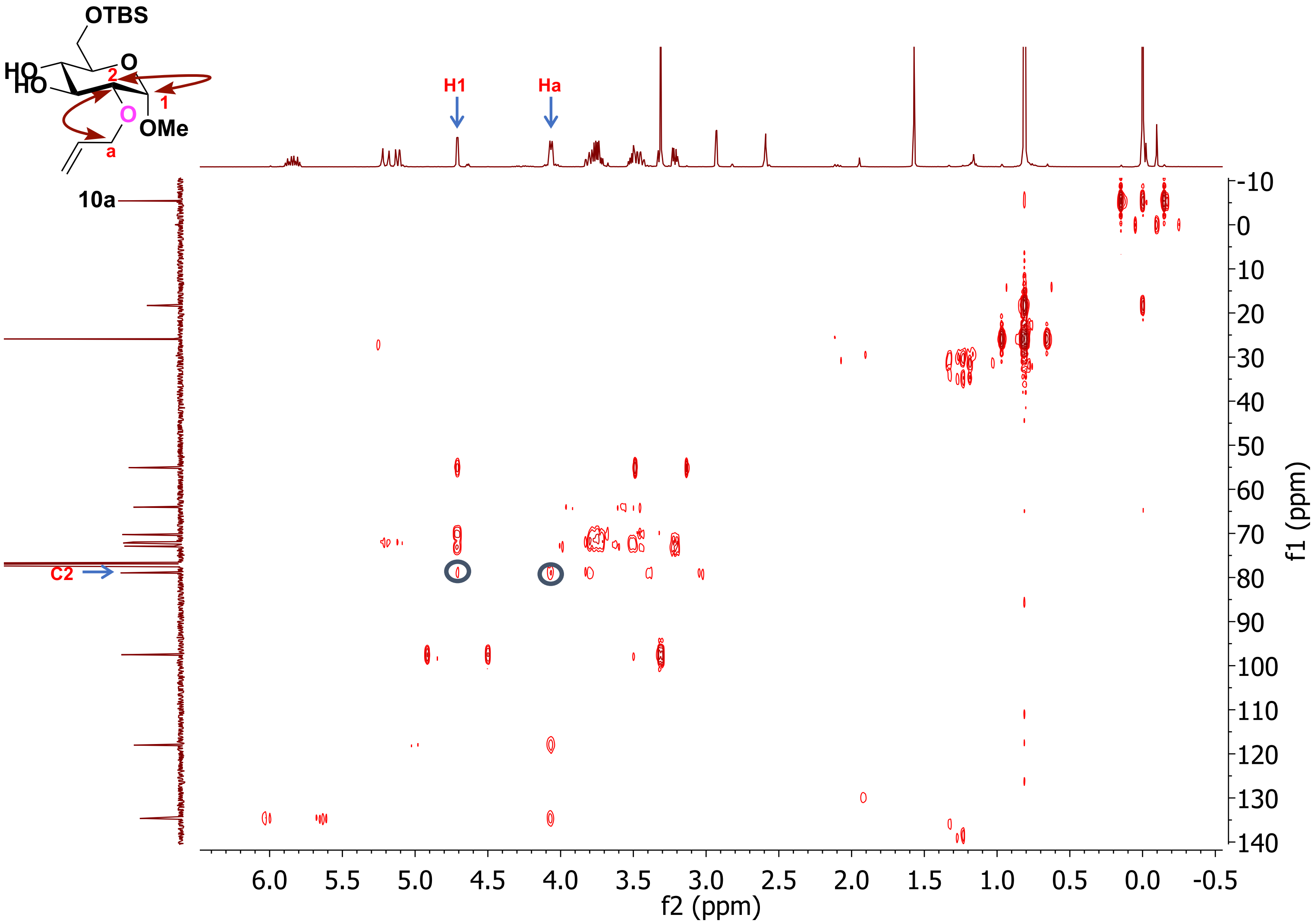
OTBS
HO
HO
O
2
1
O
OMe
a
10a
H1
Ha
C2
f2 (ppm)
f1 (ppm)

CDCl3, 400.13 MHz

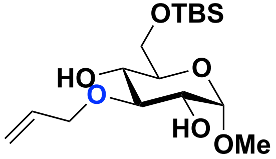

10b

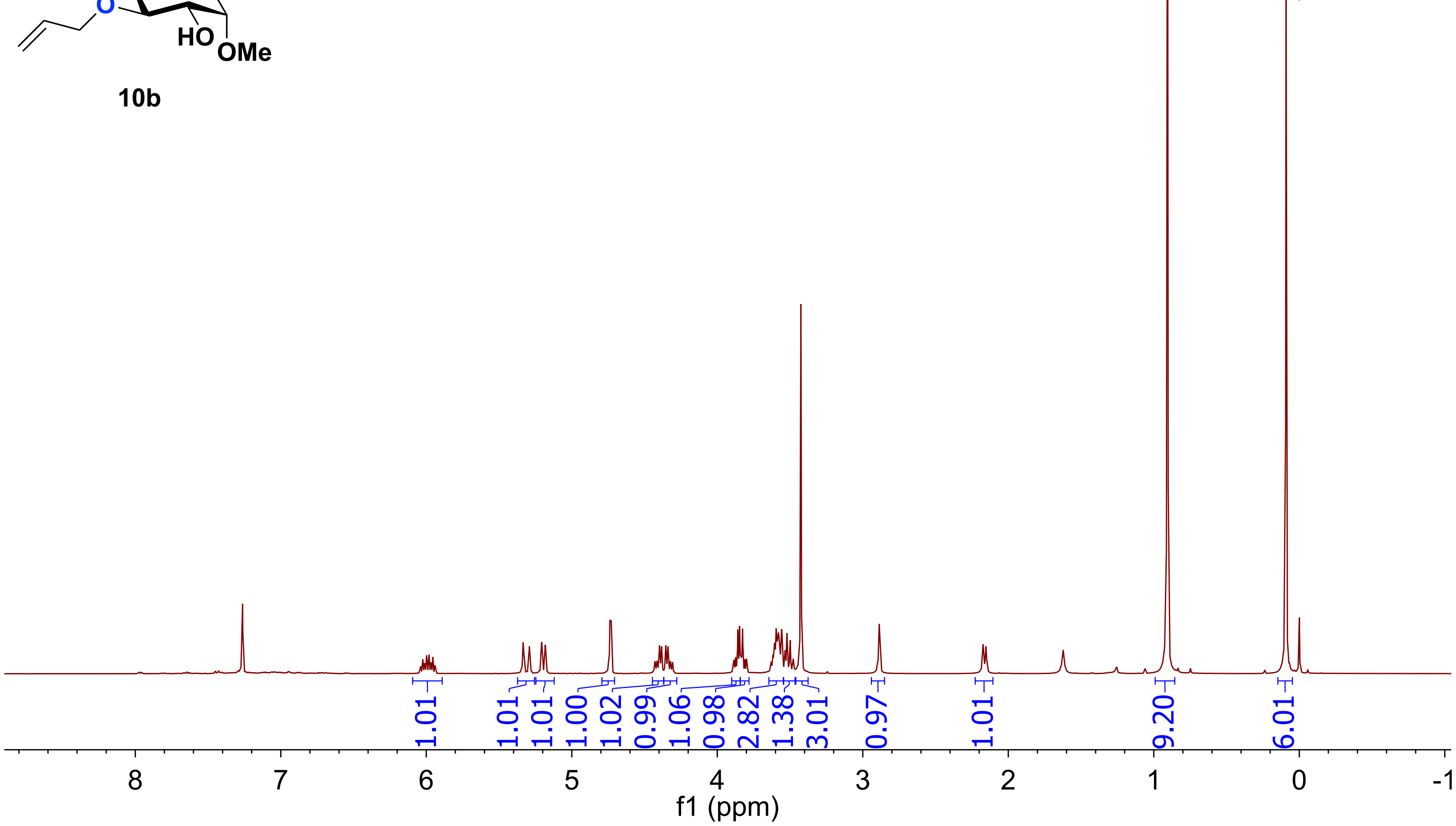

CDCl3, 100.62 MHz

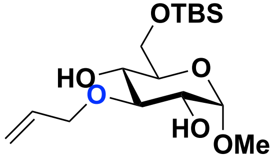

10b

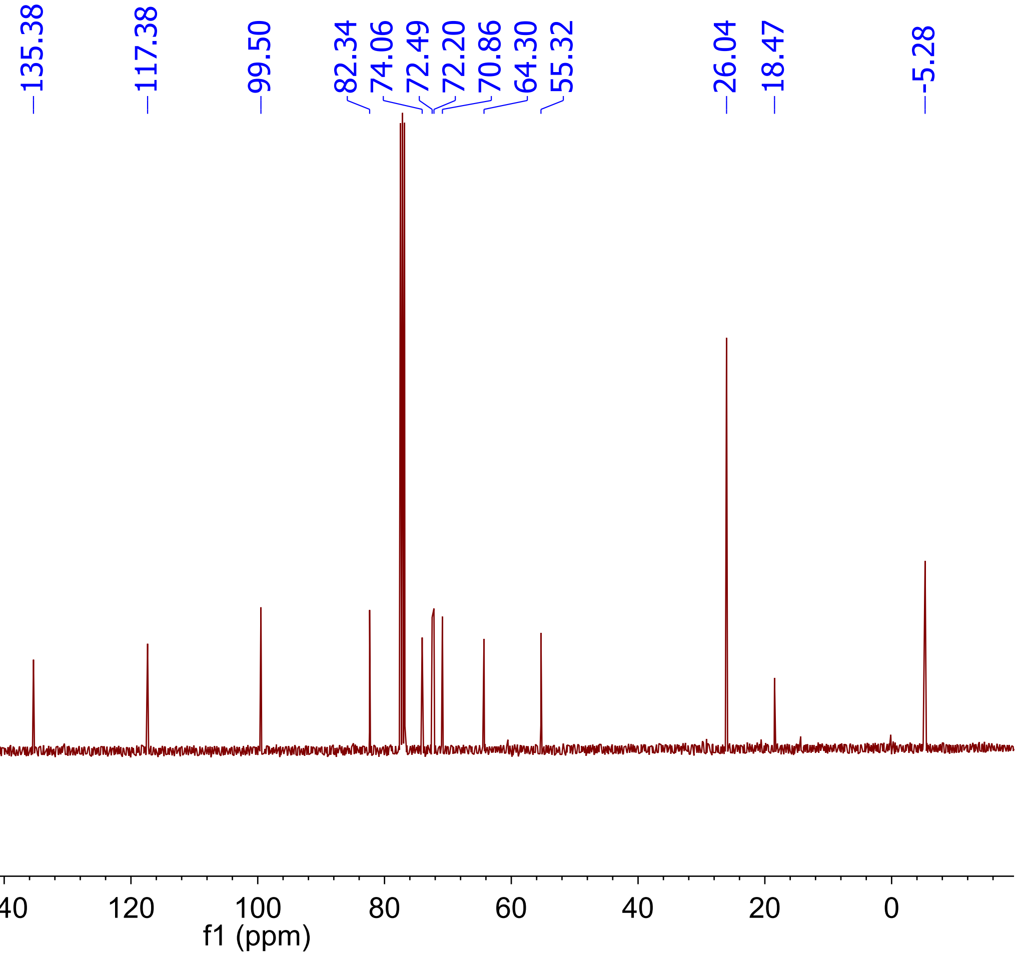

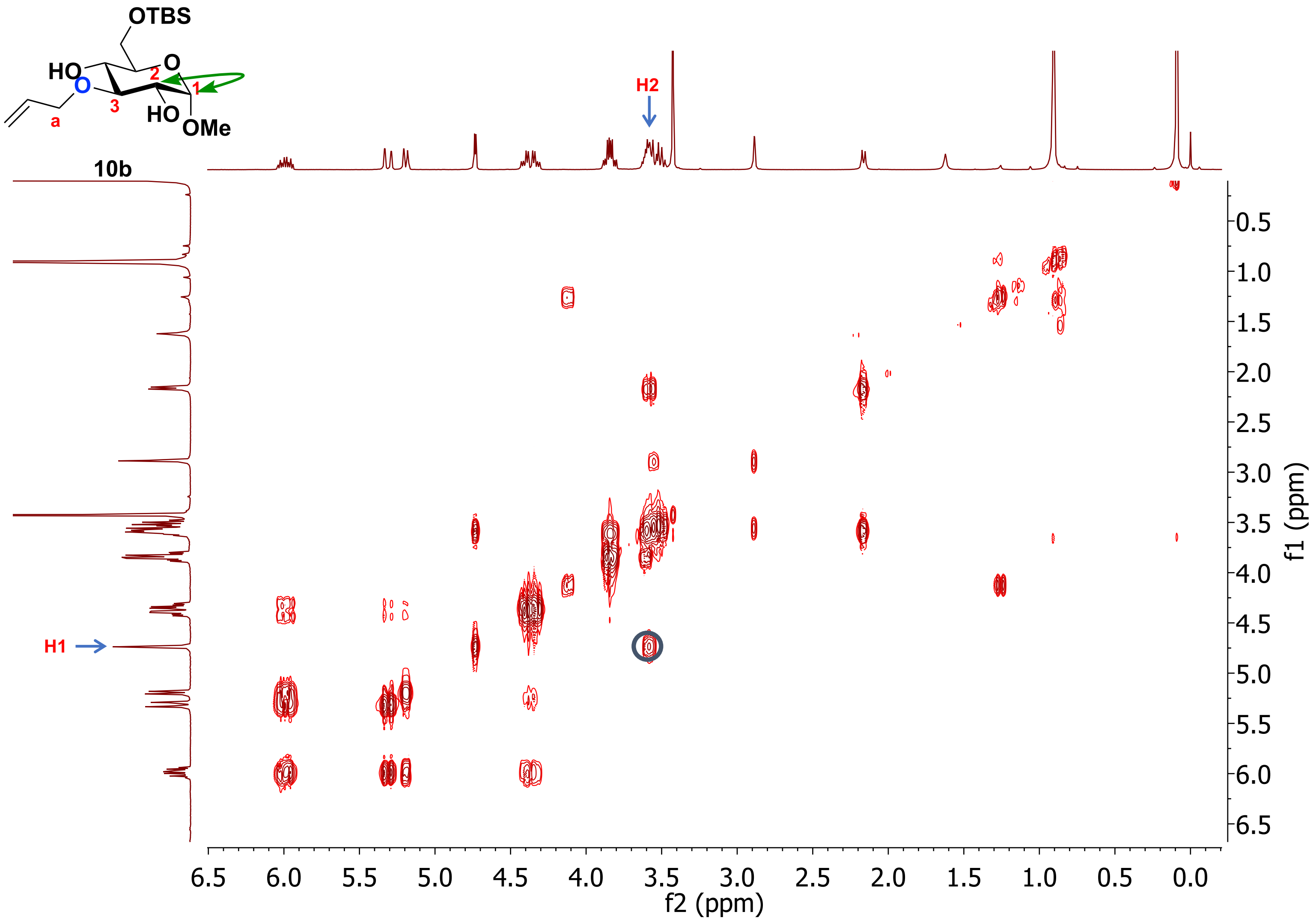
OTBS
HO
O
O
2
1
3
a
HO
OMe
10b
H2
H1
f2 (ppm)
f1 (ppm)

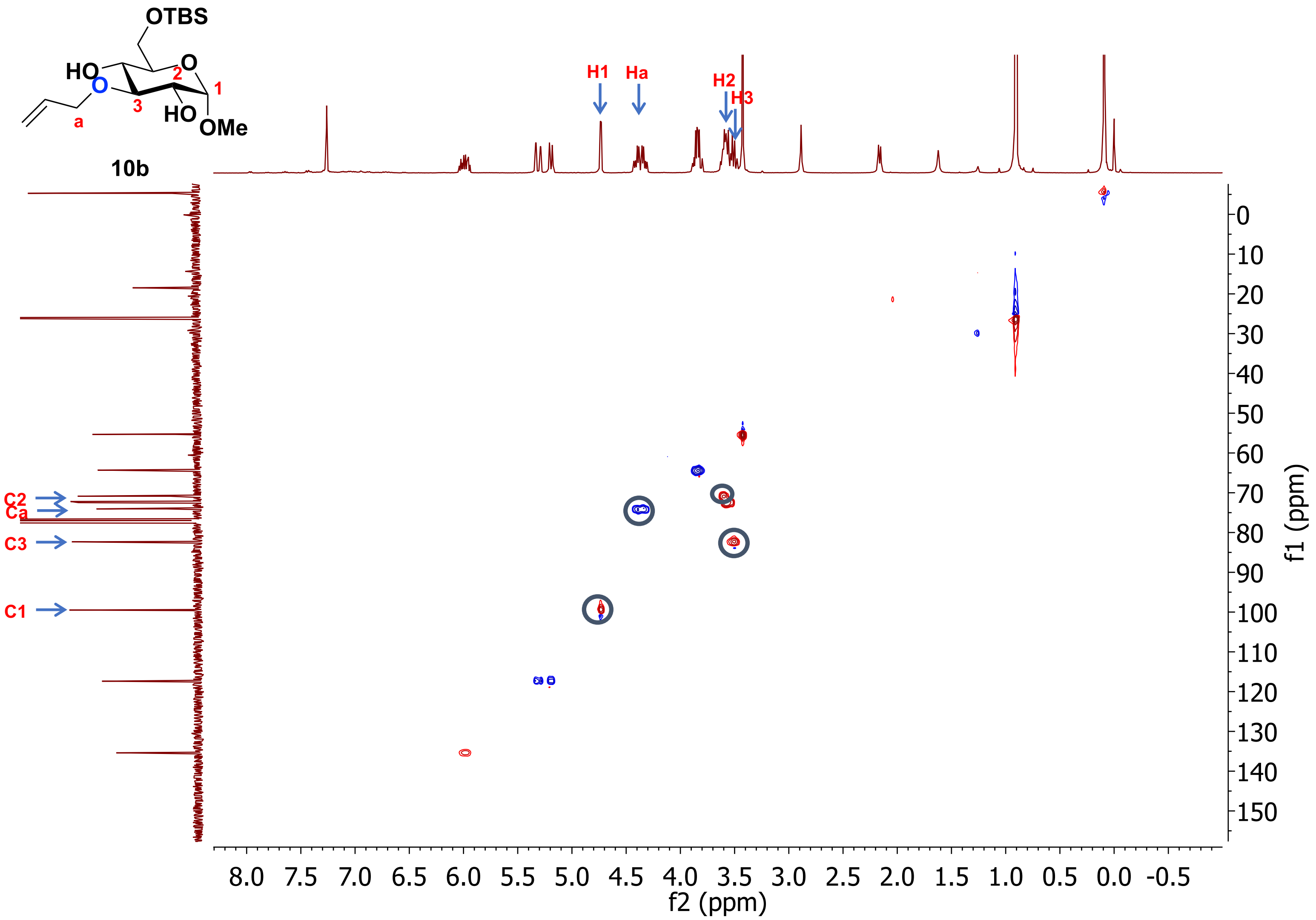
OTBS
HO
O
O
2
1
3
a
HO
OMe
10b
H1
Ha
H2
H3
C2
Ca
C3
C1
f1 (ppm)
f2 (ppm)

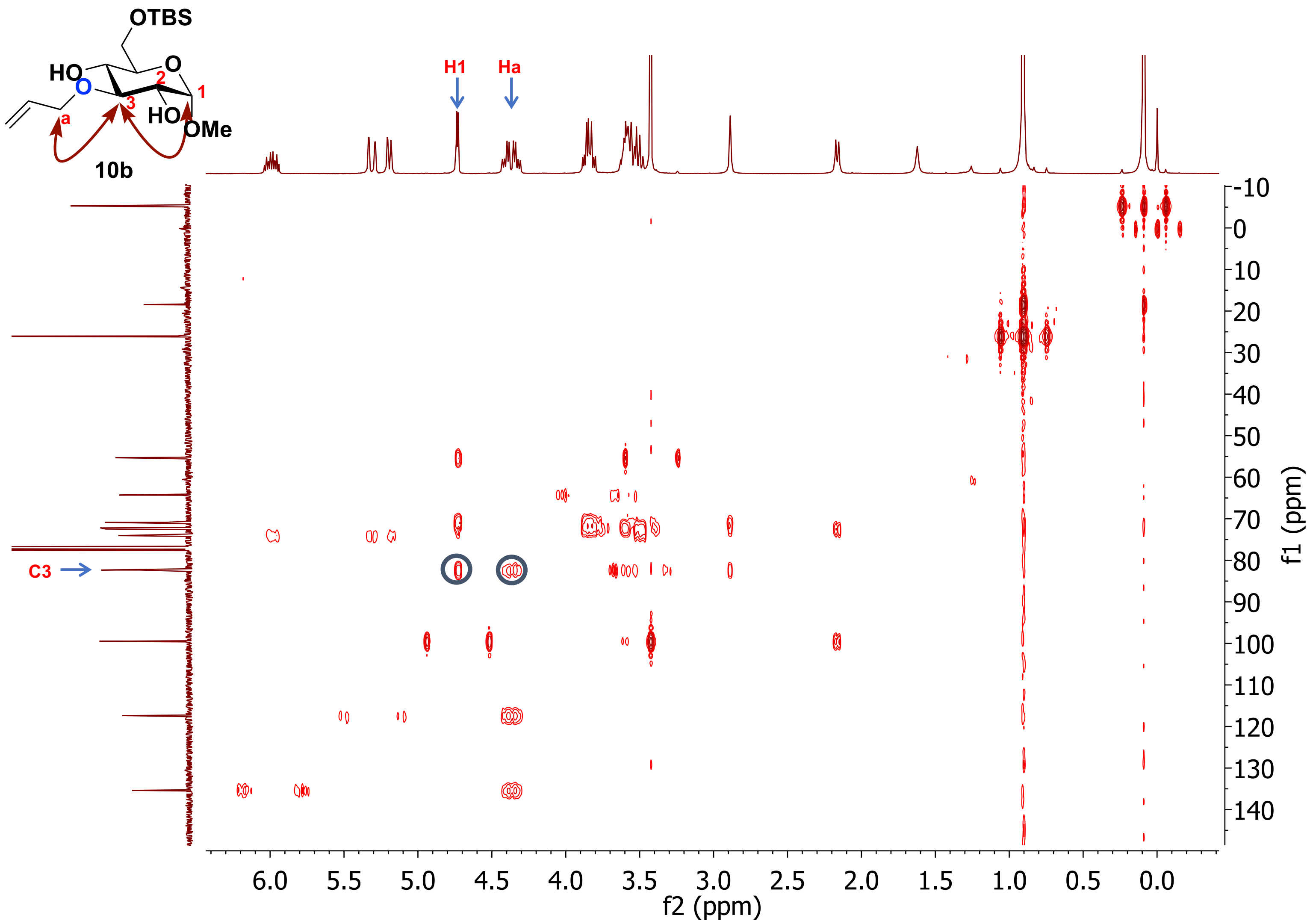
OTBS
HO
O
O
2
3
1
a
HO
OMe
10b
H1
Ha
C3
f2 (ppm)
f1 (ppm)

CDCl3, 400.13 MHz

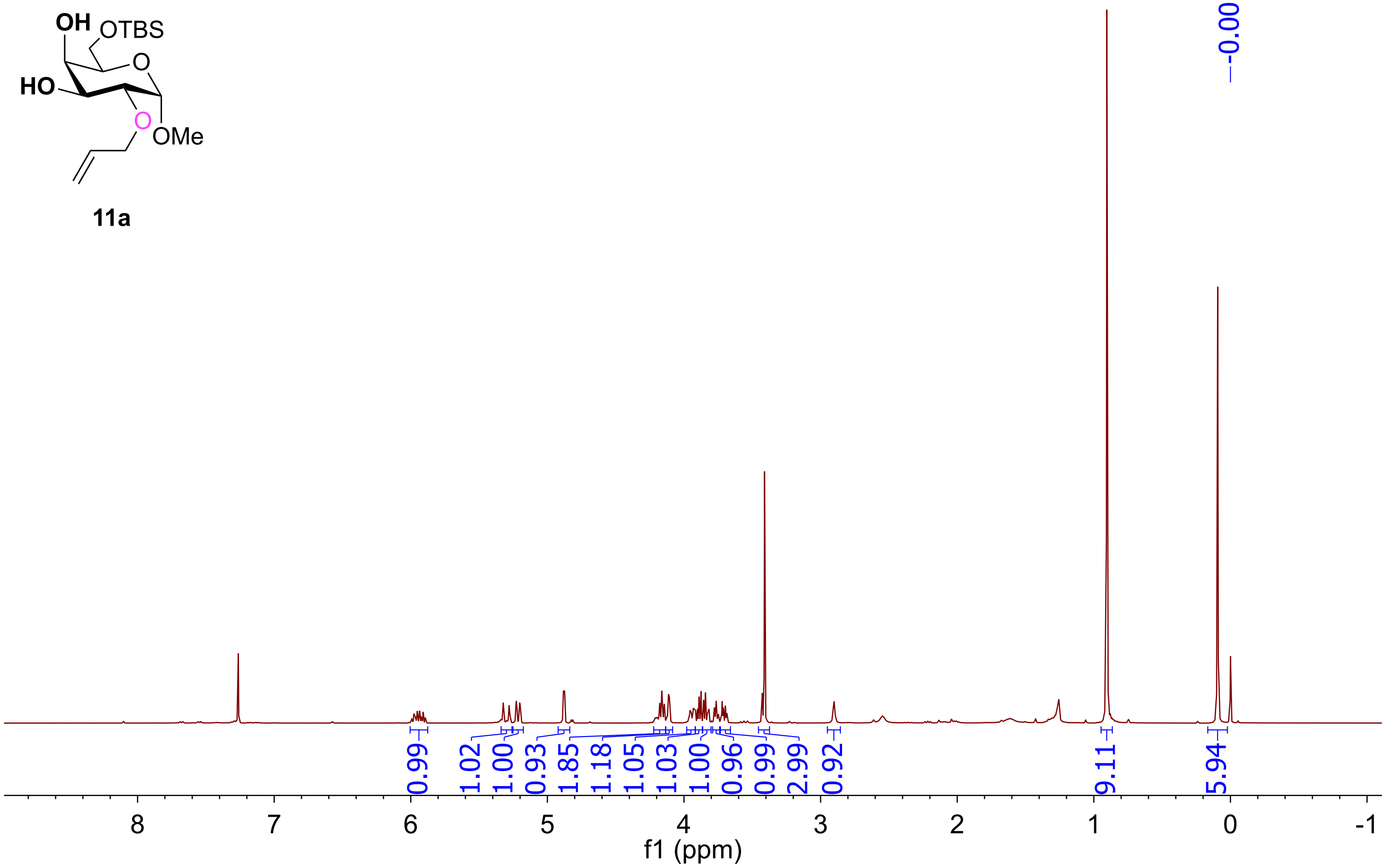

CDCl3, 100.62 MHz

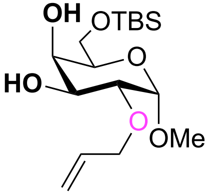

11a

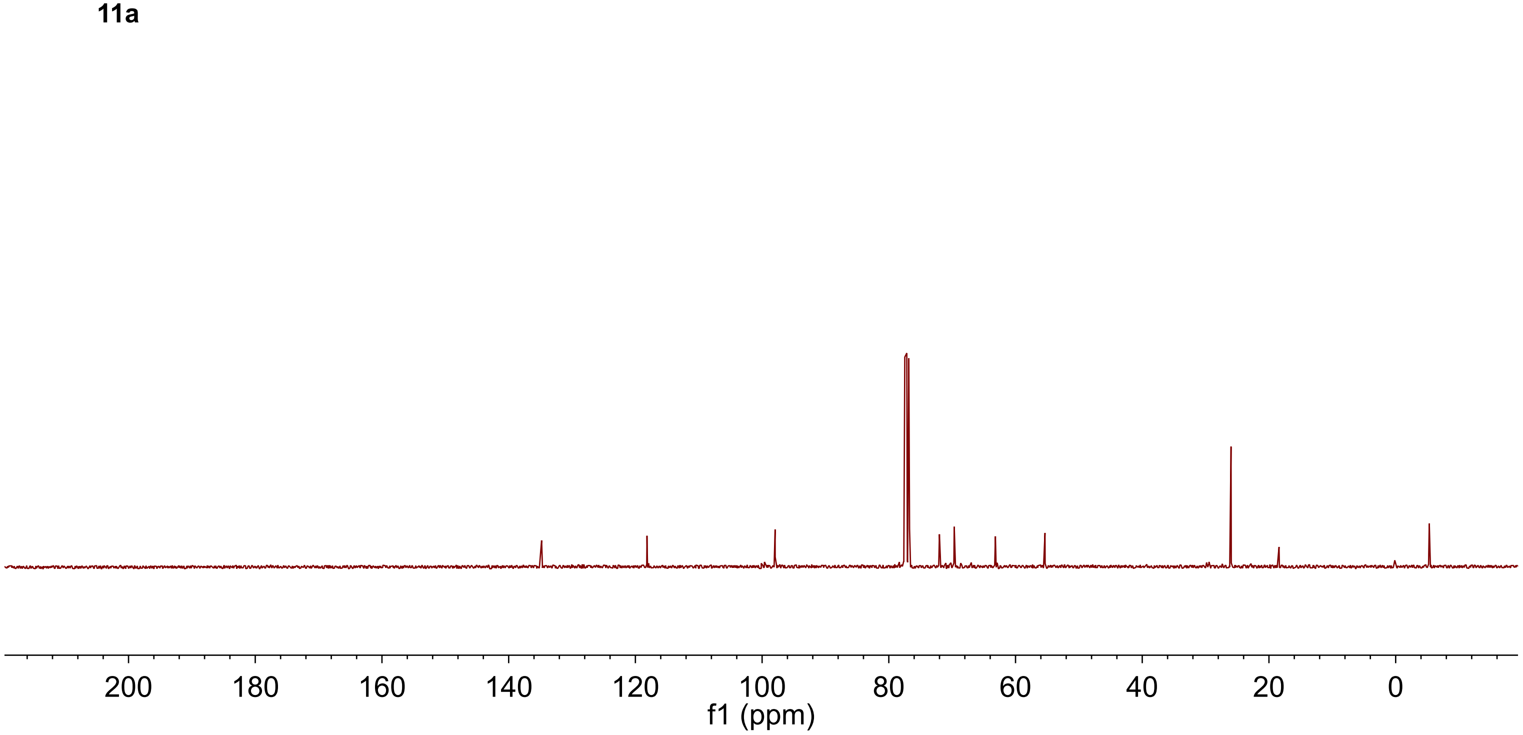

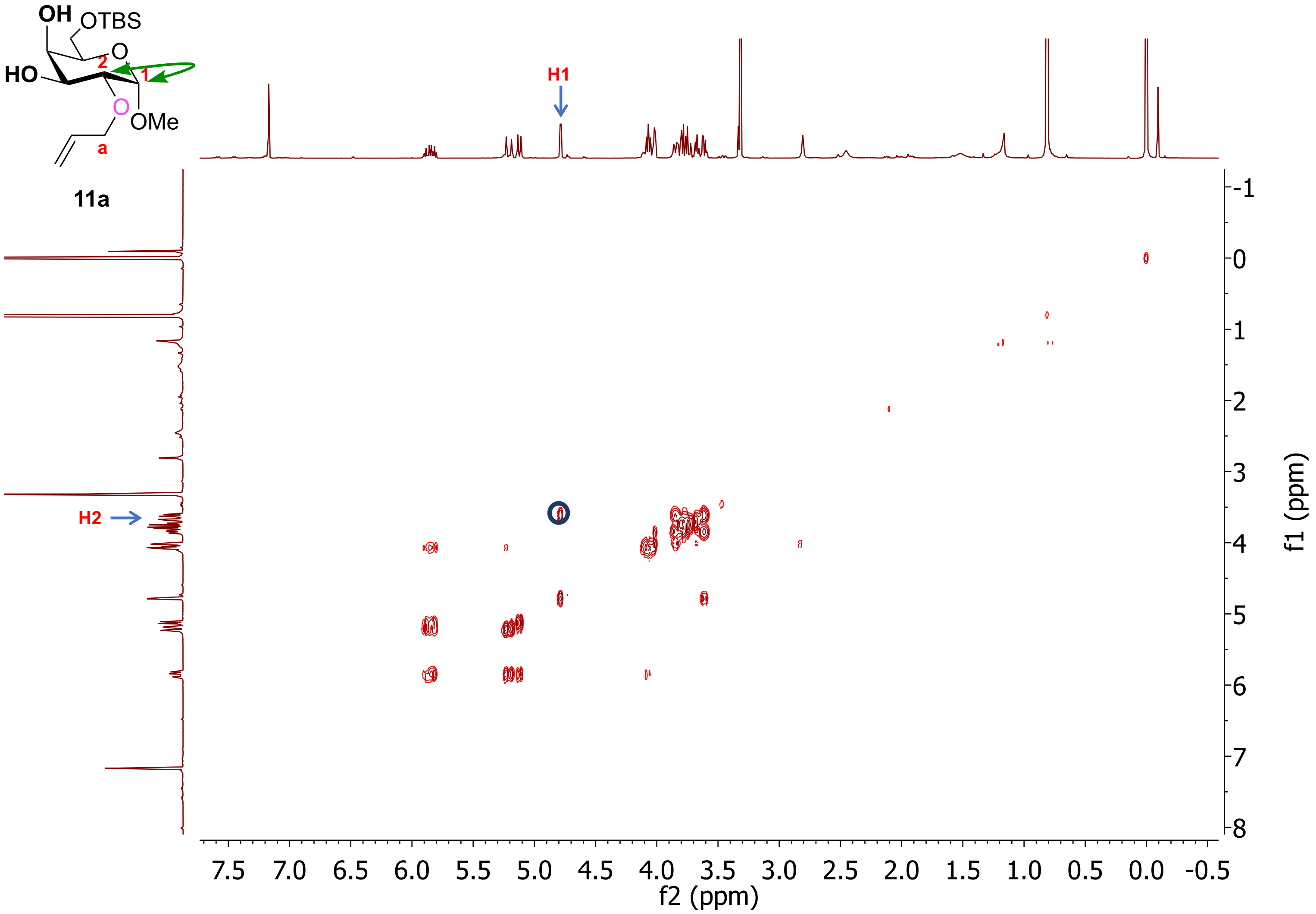
OH
OTBS
O
HO
2
1
O
OMe
a
11a
H1
H2
7.5
7.0
6.5
6.0
5.5
5.0
4.5
4.0
3.5
3.0
2.5
2.0
1.5
1.0
0.5
0.0
-0.5
f2 (ppm)
-1
0
1
2
3
4
5
6
7
8
f1 (ppm)

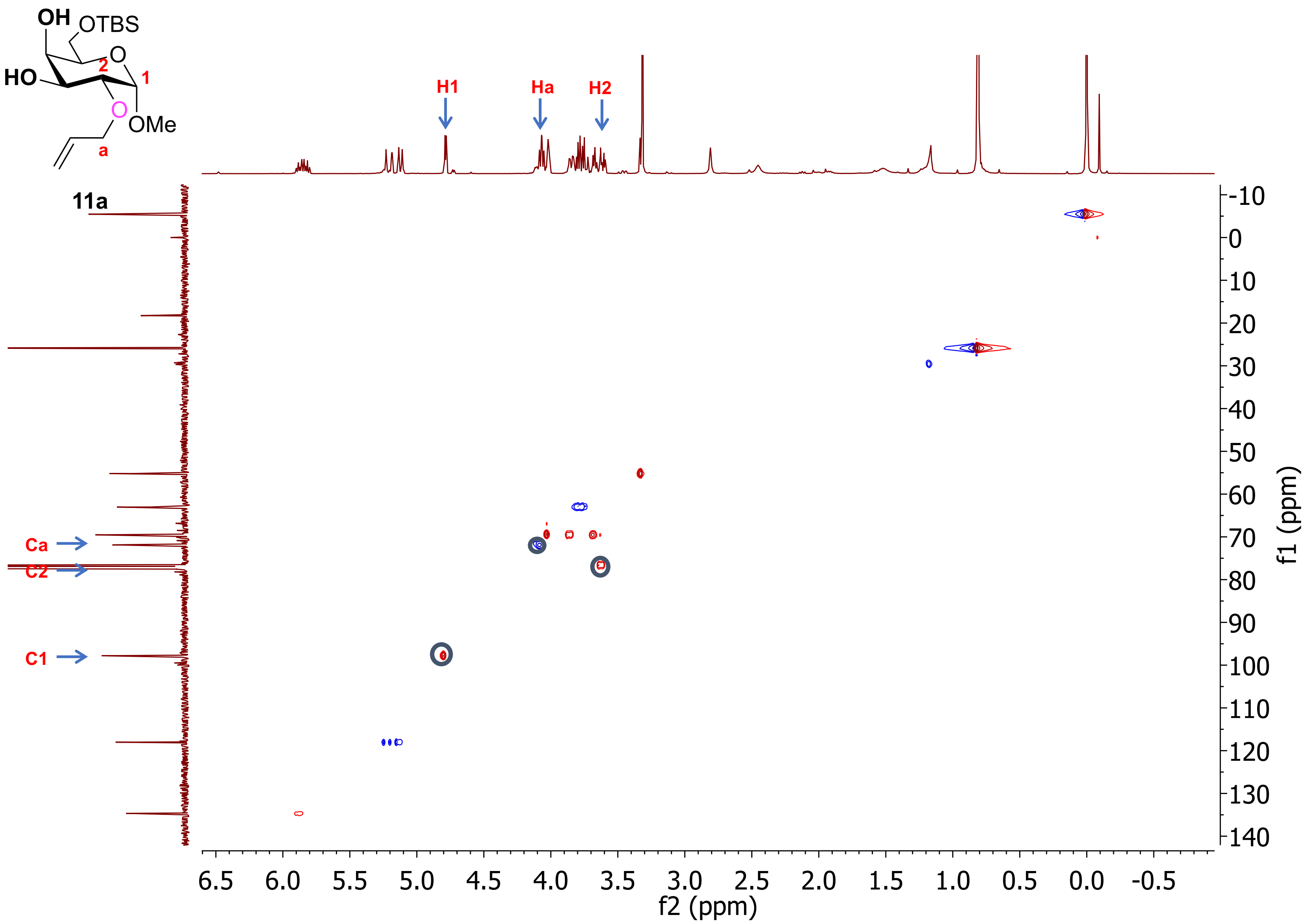
OH
OTBS
HO
O
OMe
1
2
a
11a
H1
Ha
H2
Ca
C2
C1
f2 (ppm)
f1 (ppm)

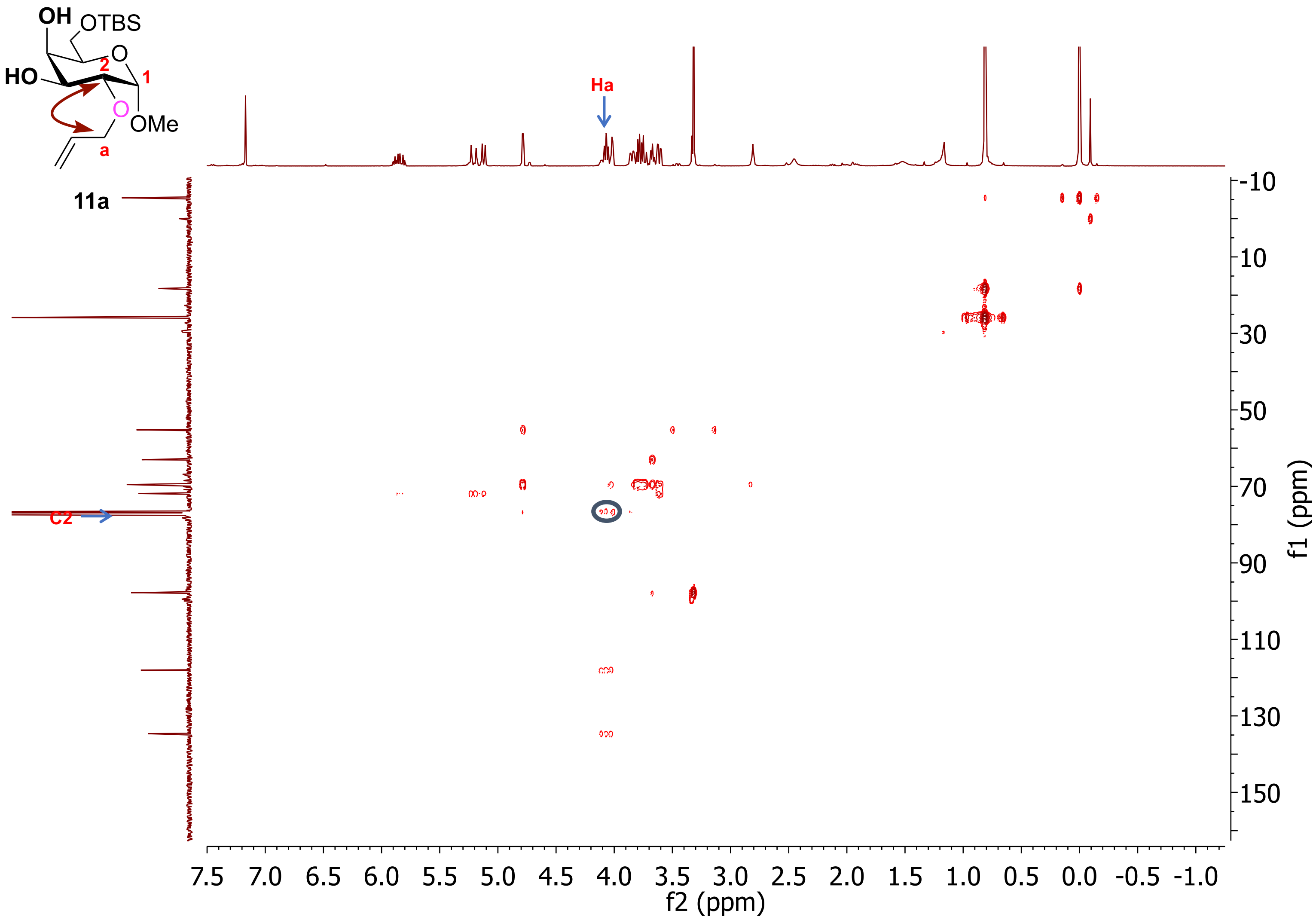
OH
OTBS
O
HO
2
1
O
OMe
a
Ha
11a
C2
f2 (ppm)
f1 (ppm)

CDCl3, 400 MHz

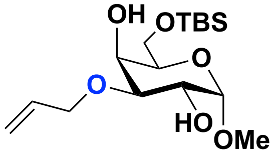

11b

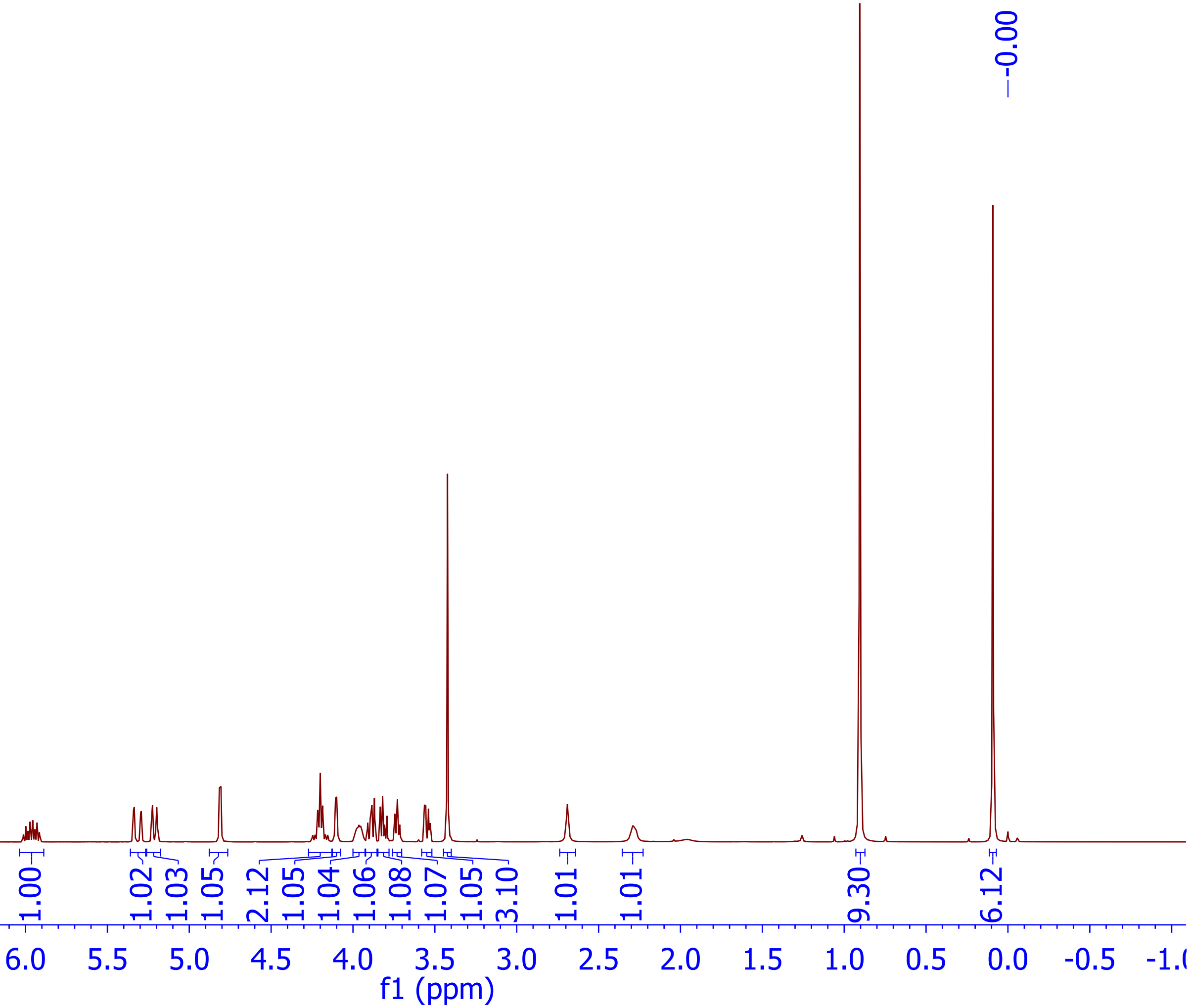

CDCl3, 101 MHz

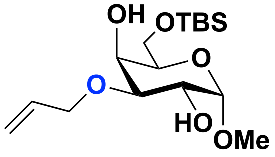

11b

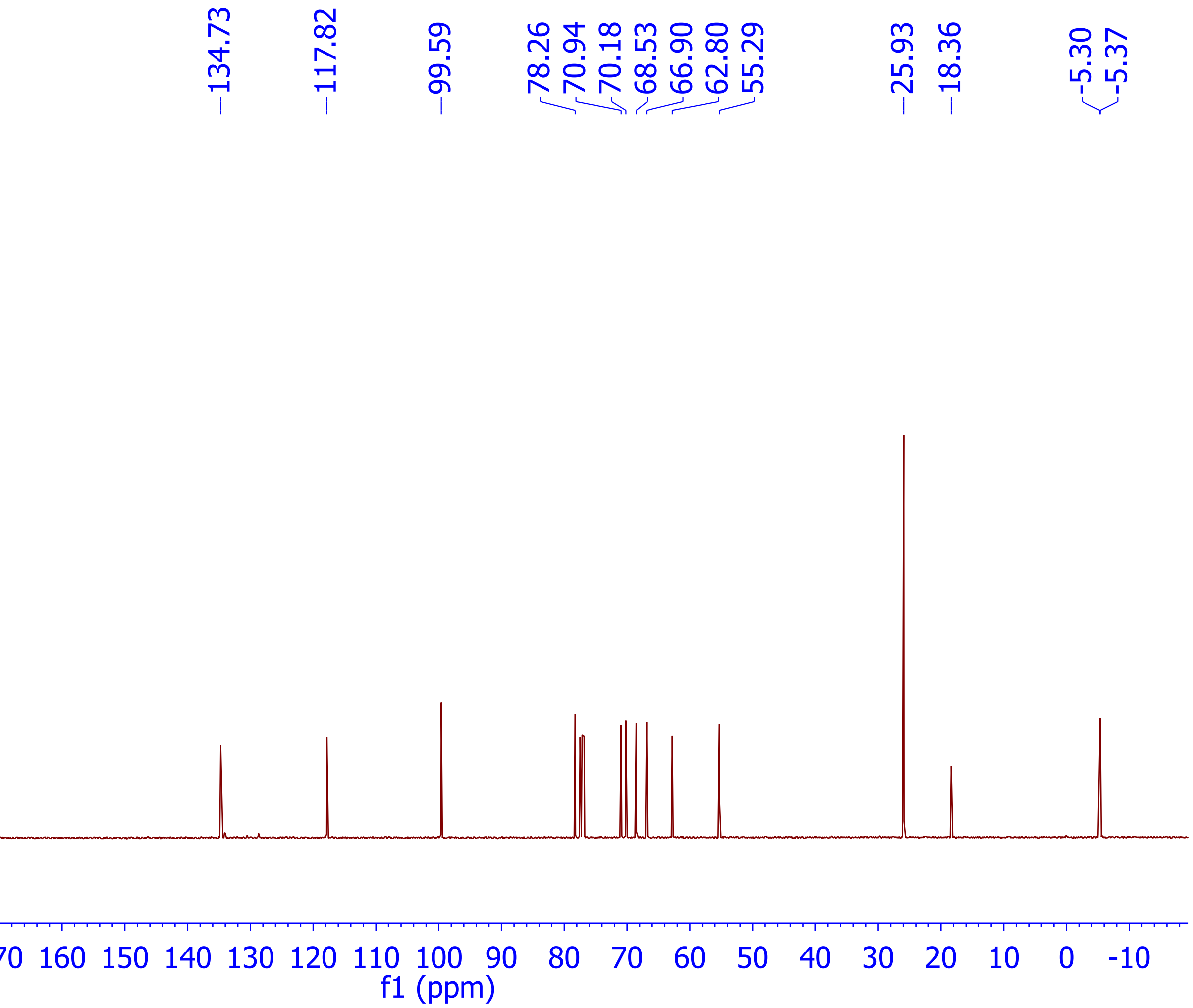

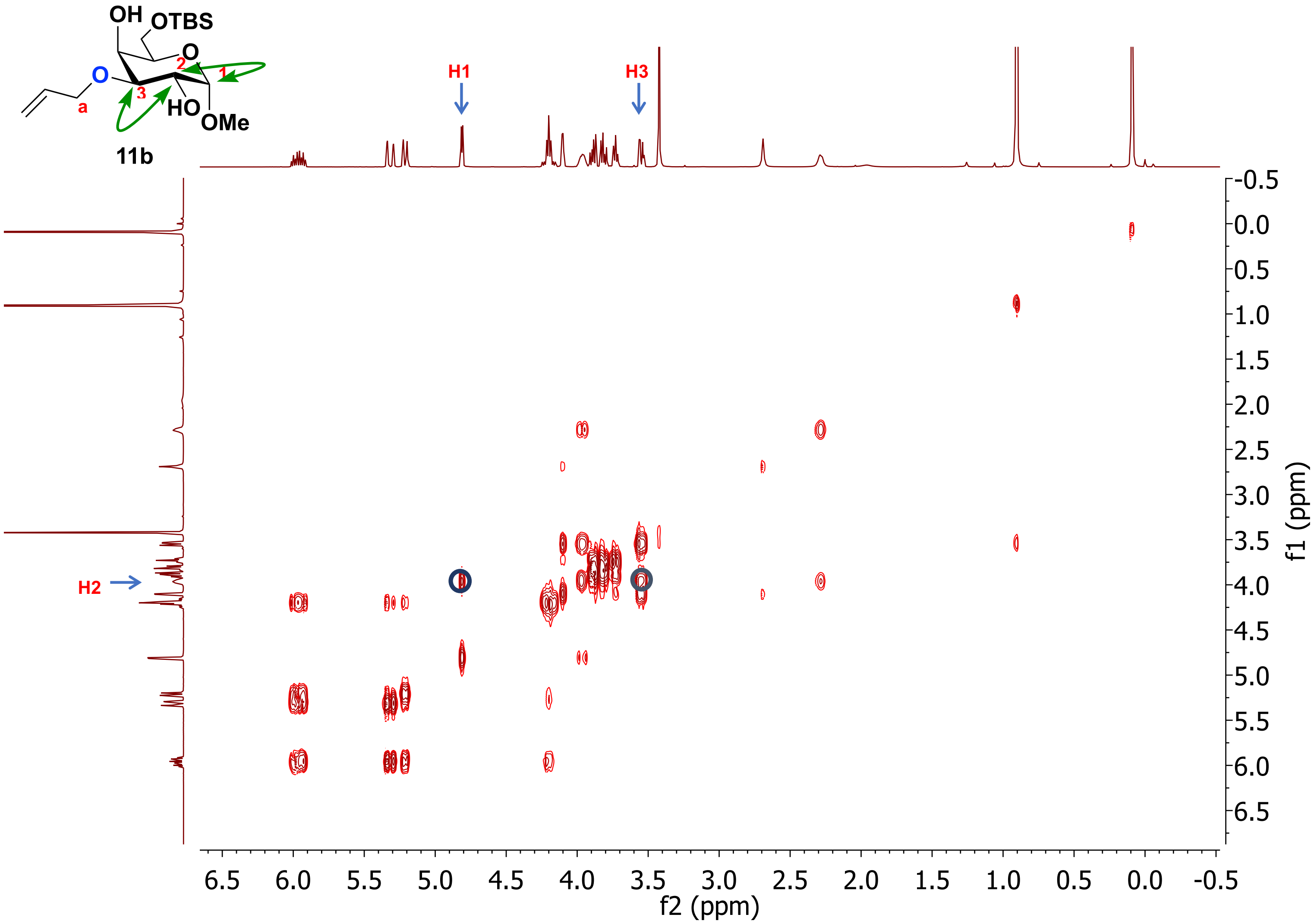
OH
OTBS
O
O
1
2
3
a
HO
OMe
11b
H1
H3
H2
6.5
6.0
5.5
5.0
4.5
4.0
3.5
3.0
2.5
2.0
1.5
1.0
0.5
0.0
-0.5
f2 (ppm)
f1 (ppm)

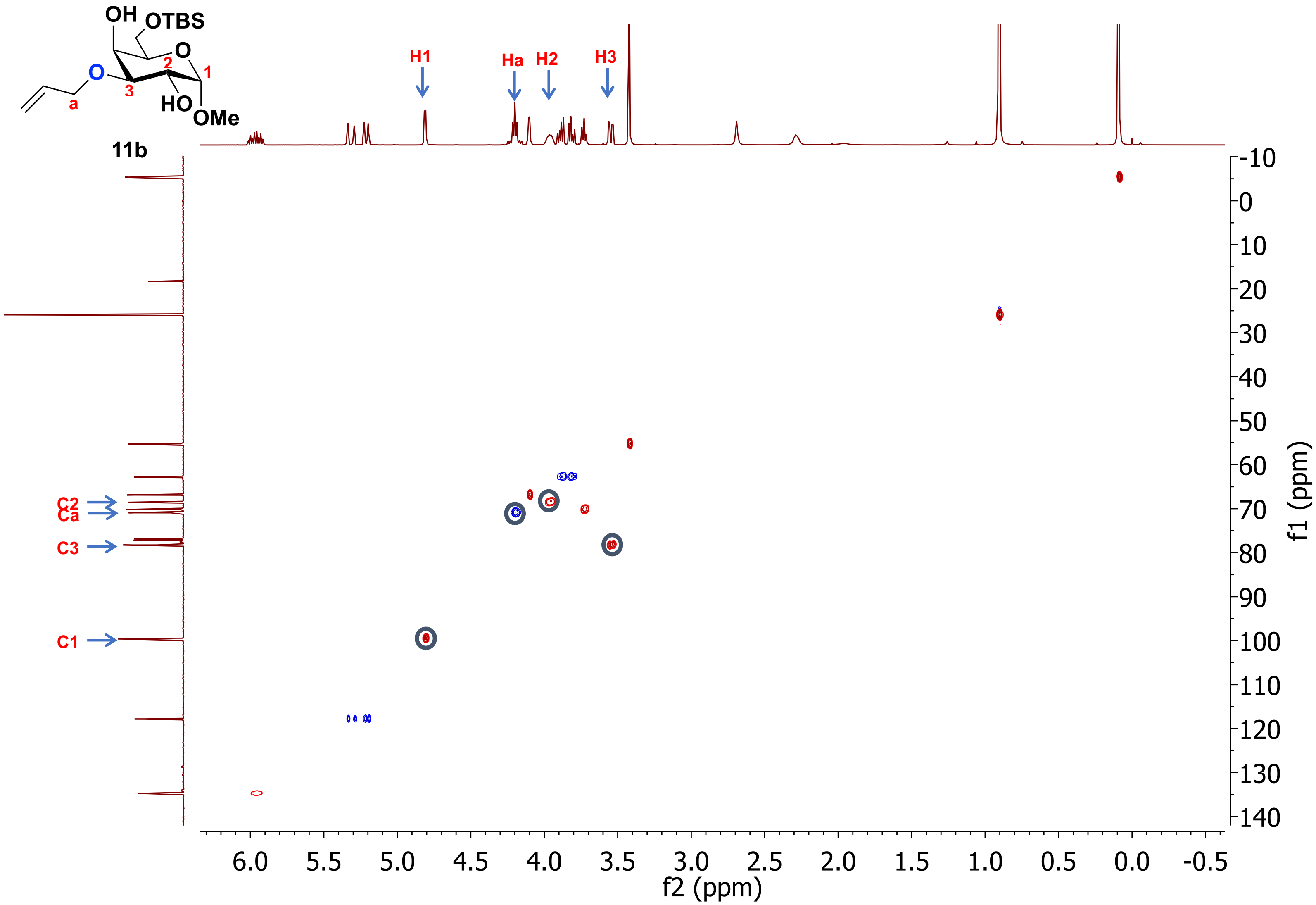
OH
OTBS
O
O
2
1
3
a
HO
OMe
11b
H1
Ha
H2
H3
C2
Ca
C3
C1
f2 (ppm)
f1 (ppm)

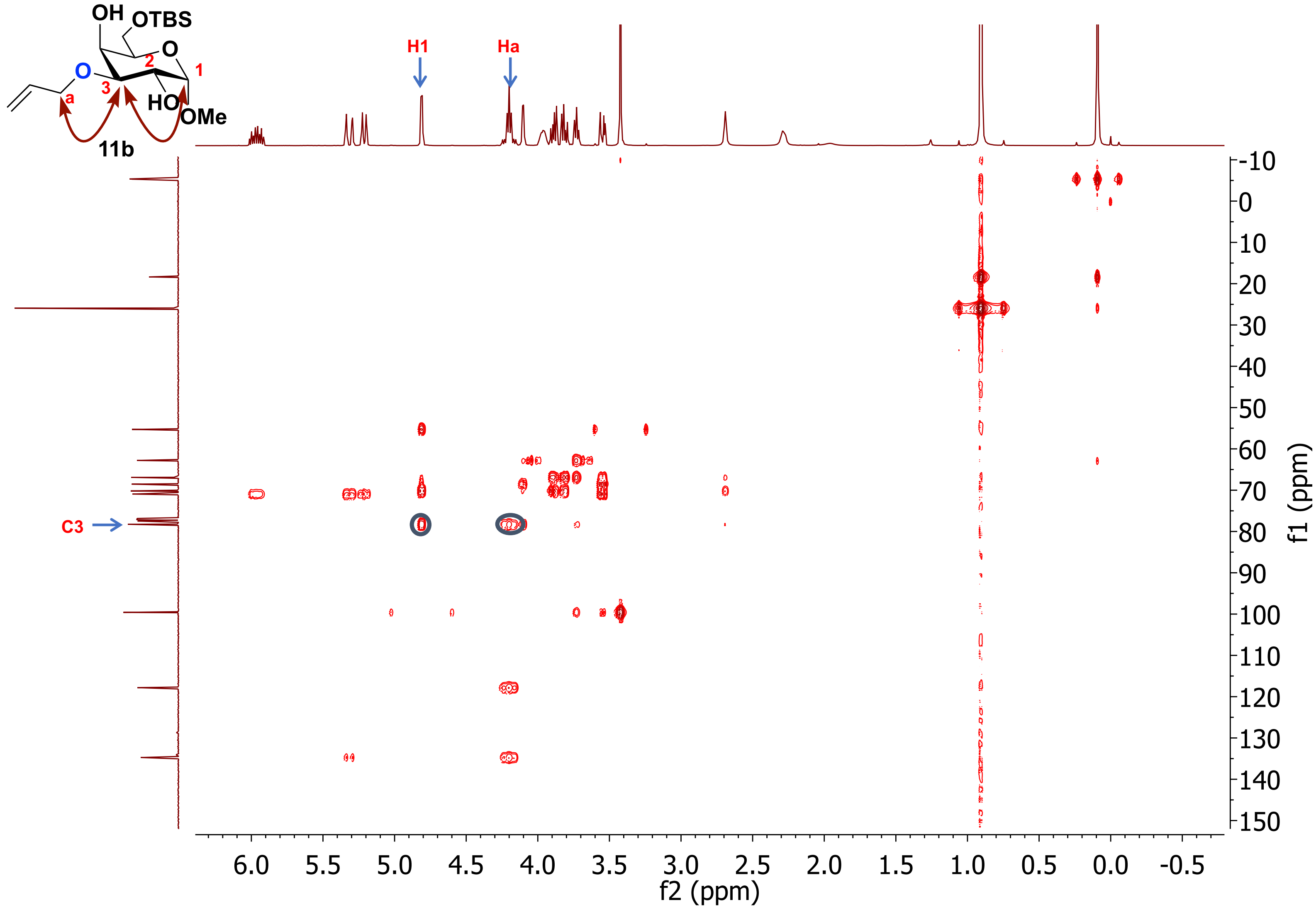

OH
OTBS
O
O
1
2
3
a
HO
OMe
11b
H1
Ha
C3
f2 (ppm)
f1 (ppm)

CDCl3, 400.13 MHz

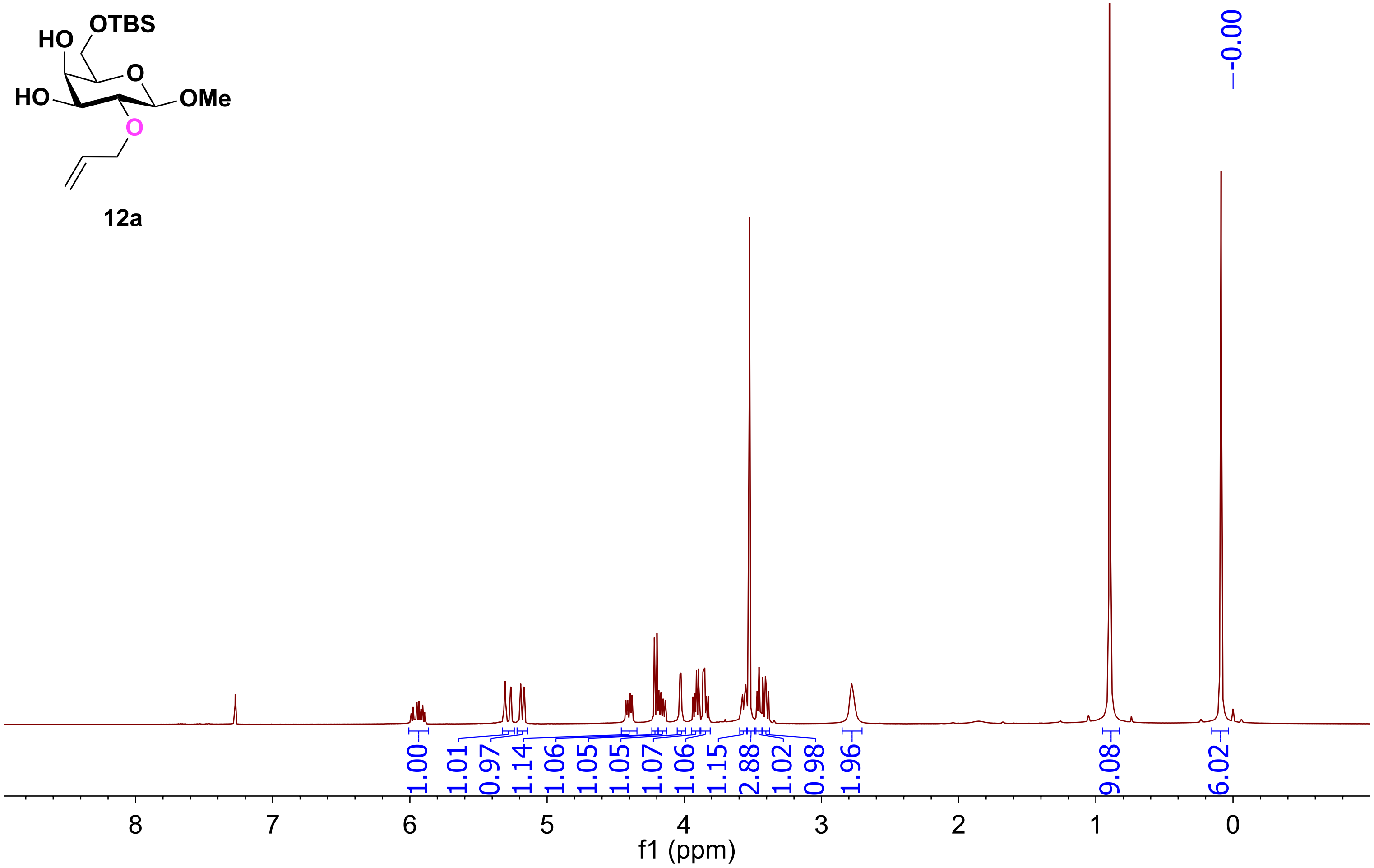

CDCl3, 100.62 MHz

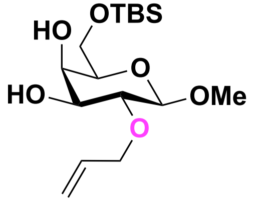

12a

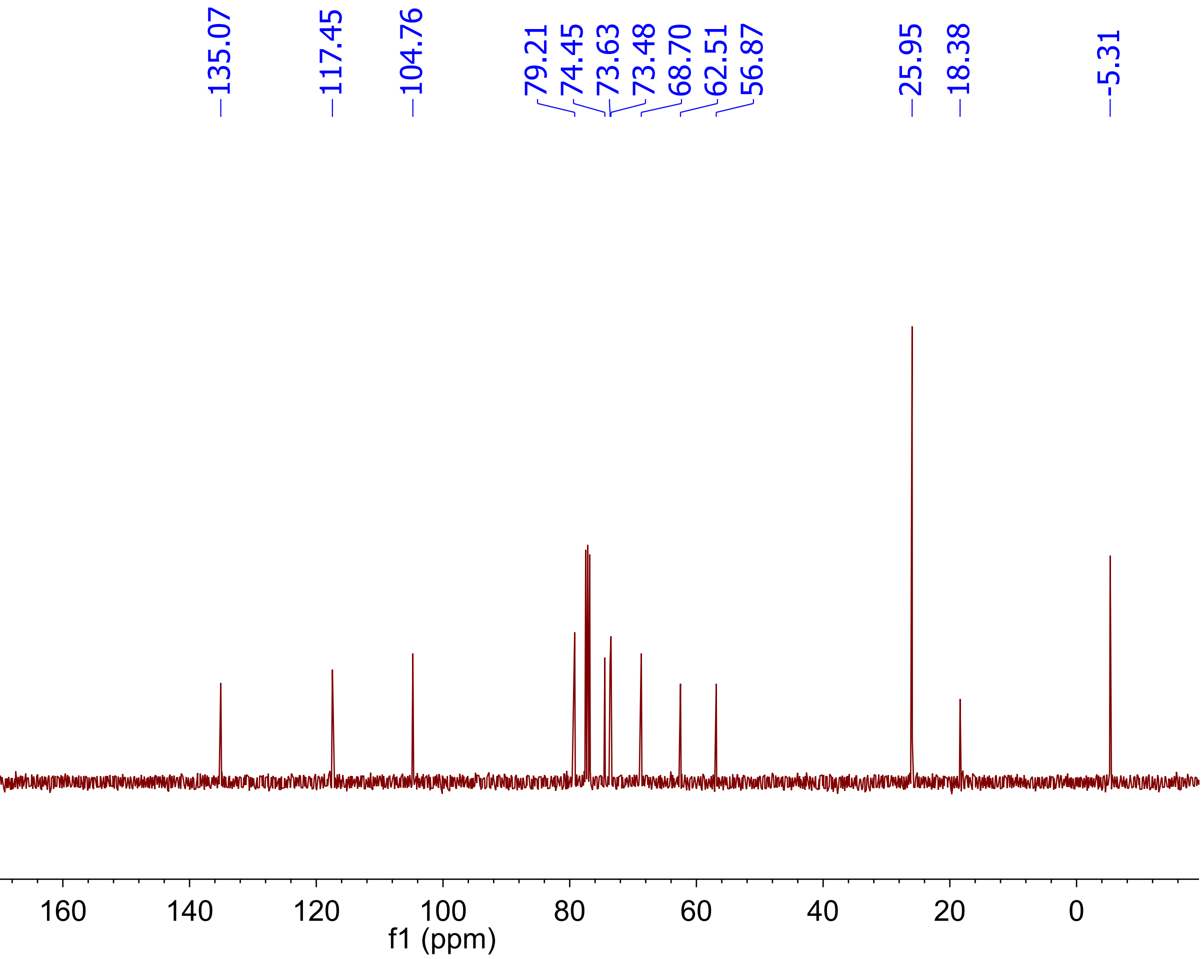

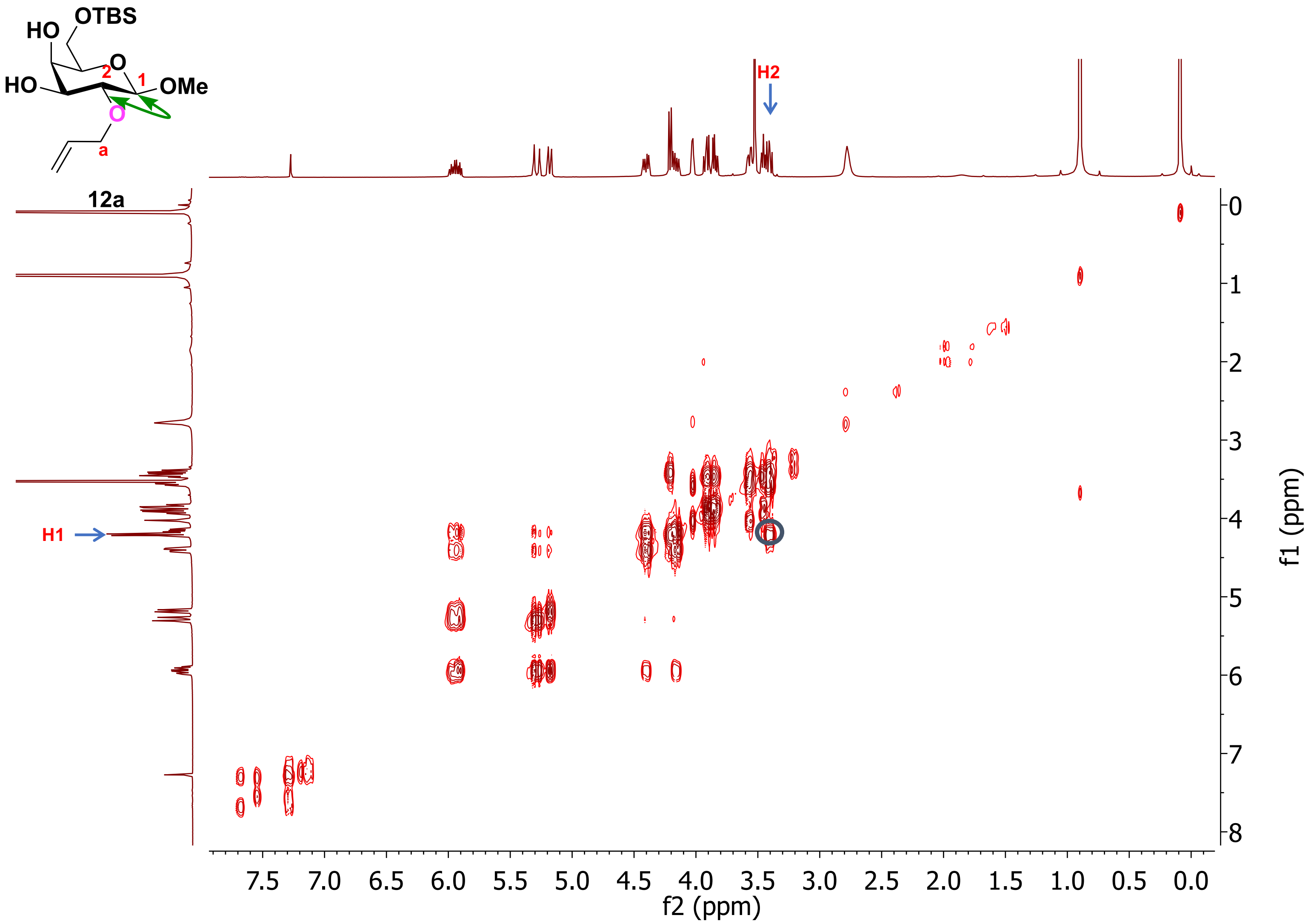
OTBS
HO
HO
O
2
1
OMe
O
a
12a
H2
H1
f2 (ppm)
f1 (ppm)

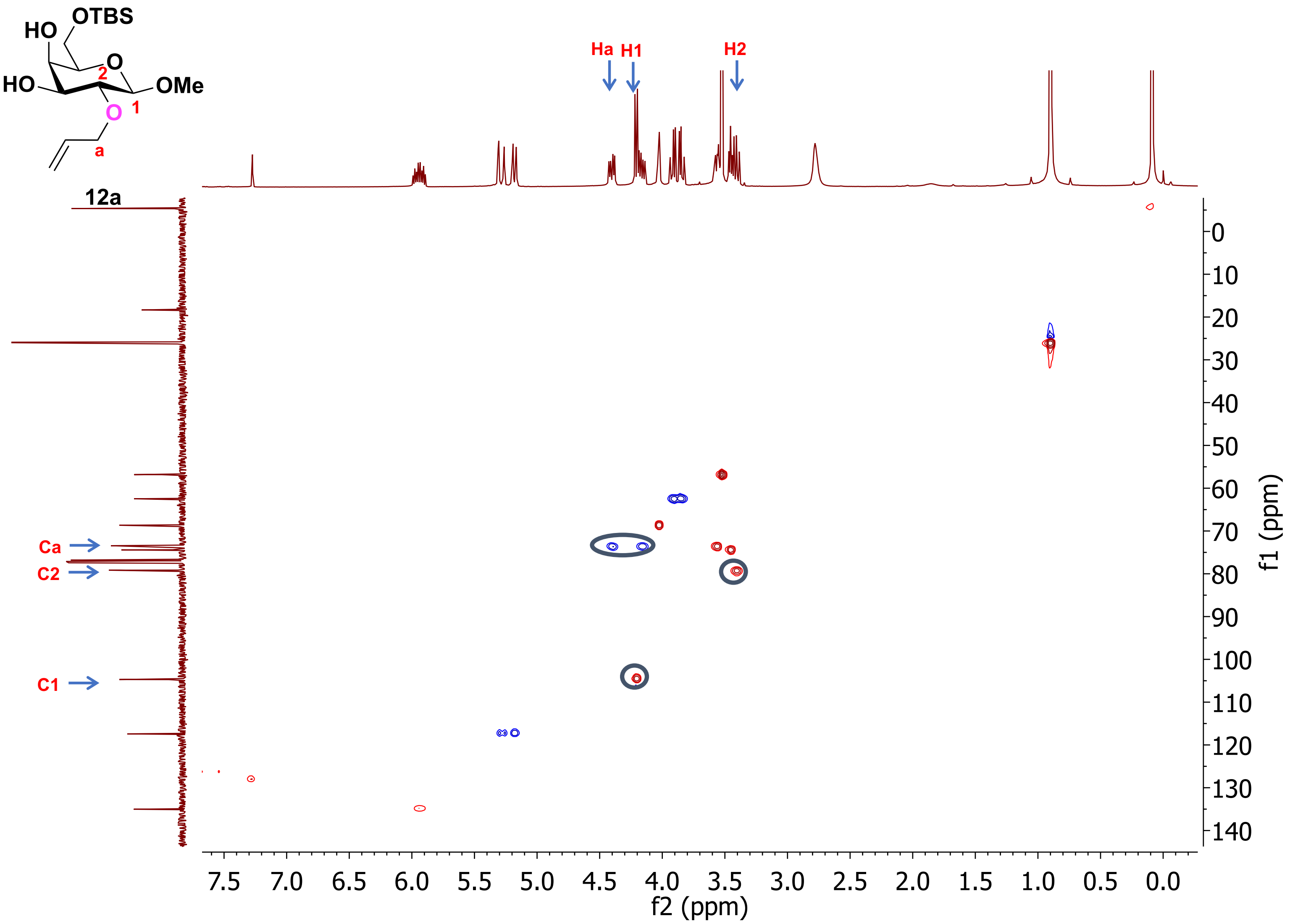

HO
OTBS
HO
O
OMe
2
1
O
a
12a
Ha
H1
H2
Ca
C2
C1
f2 (ppm)
f1 (ppm)

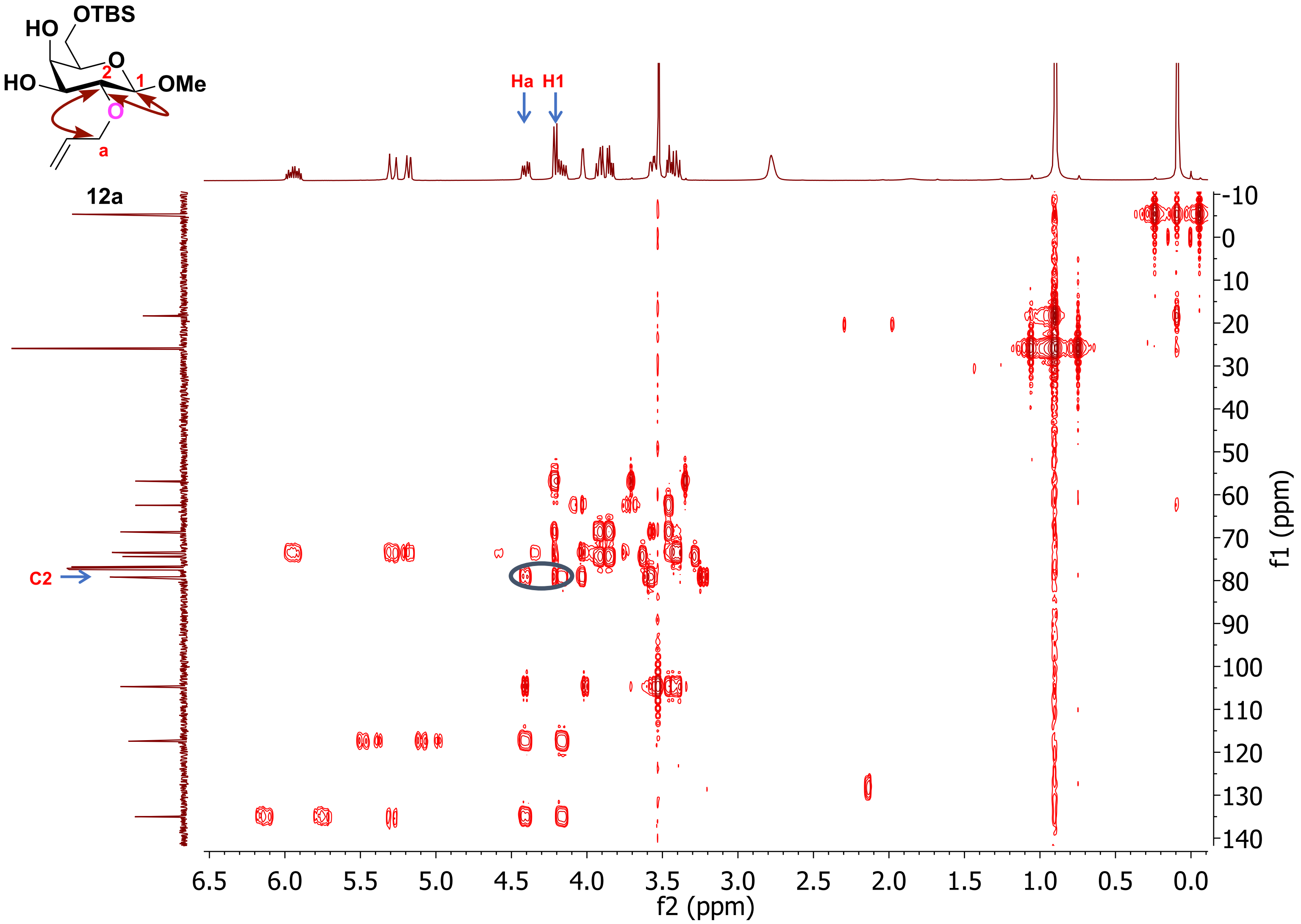

HO
OTBS
O
HO
2
1
OMe
O
a
12a
Ha
H1
C2
f1 (ppm)
f2 (ppm)

CDCl3, 400.13 MHz

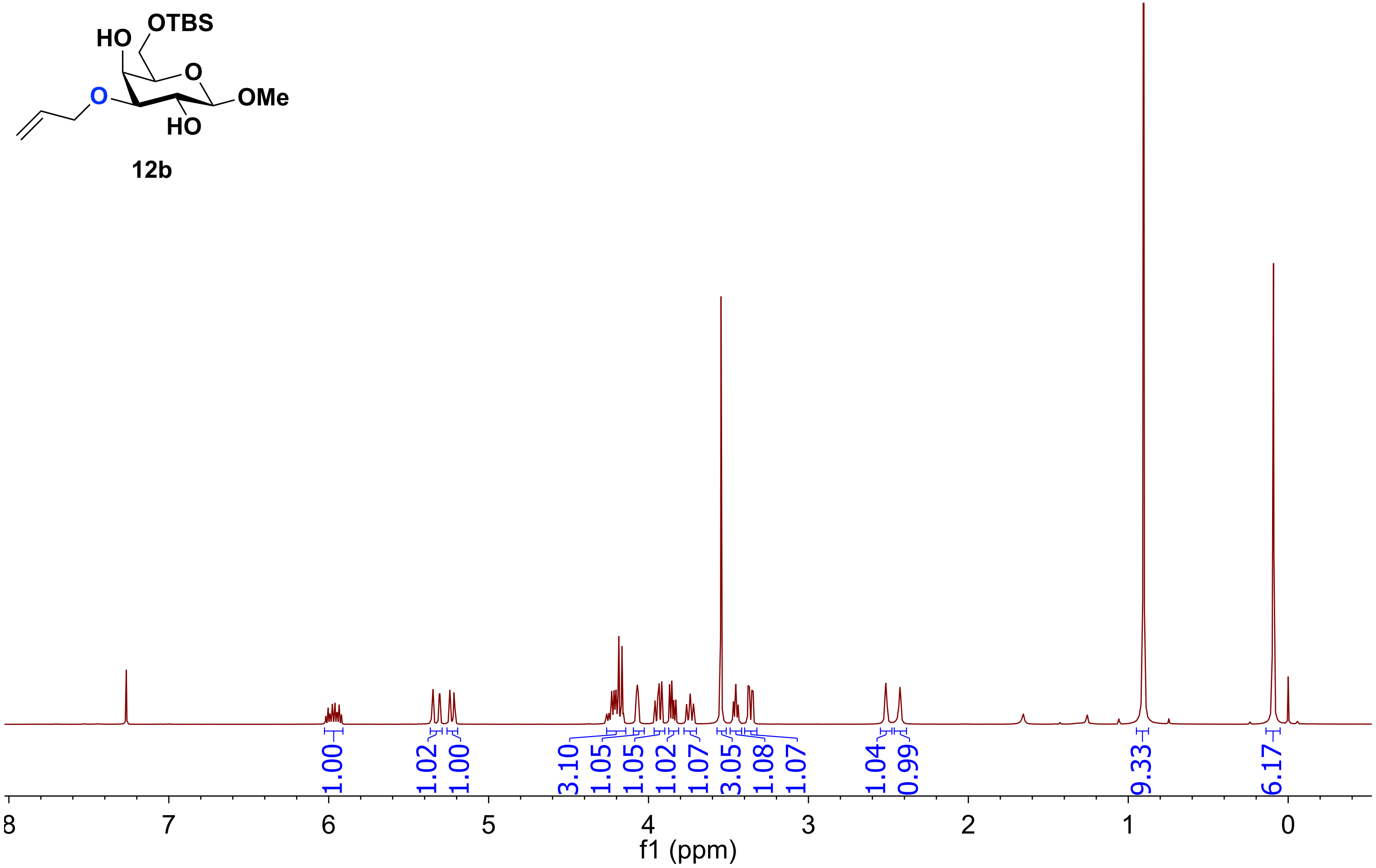

CDCl3, 100.62 MHz

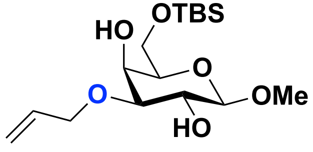

12b

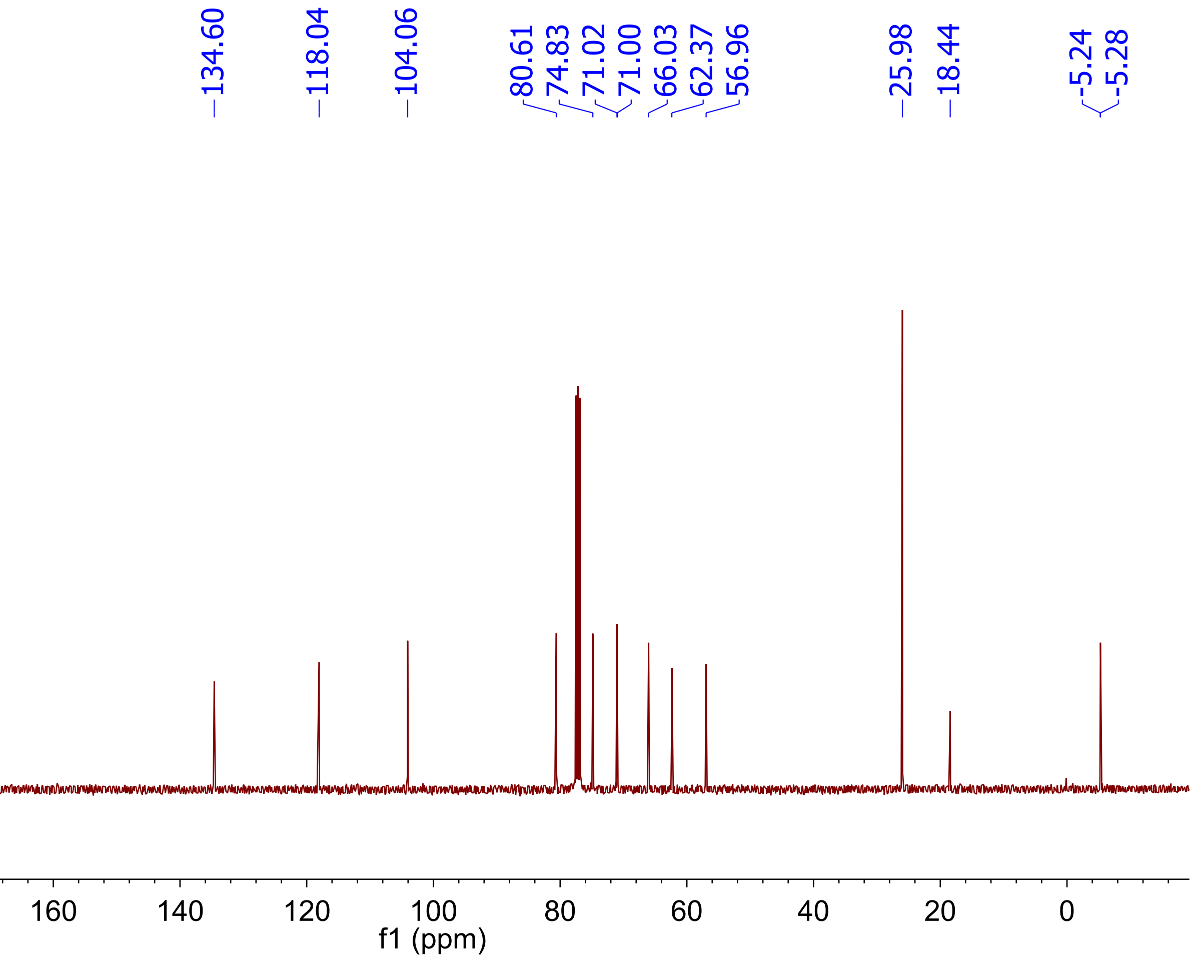

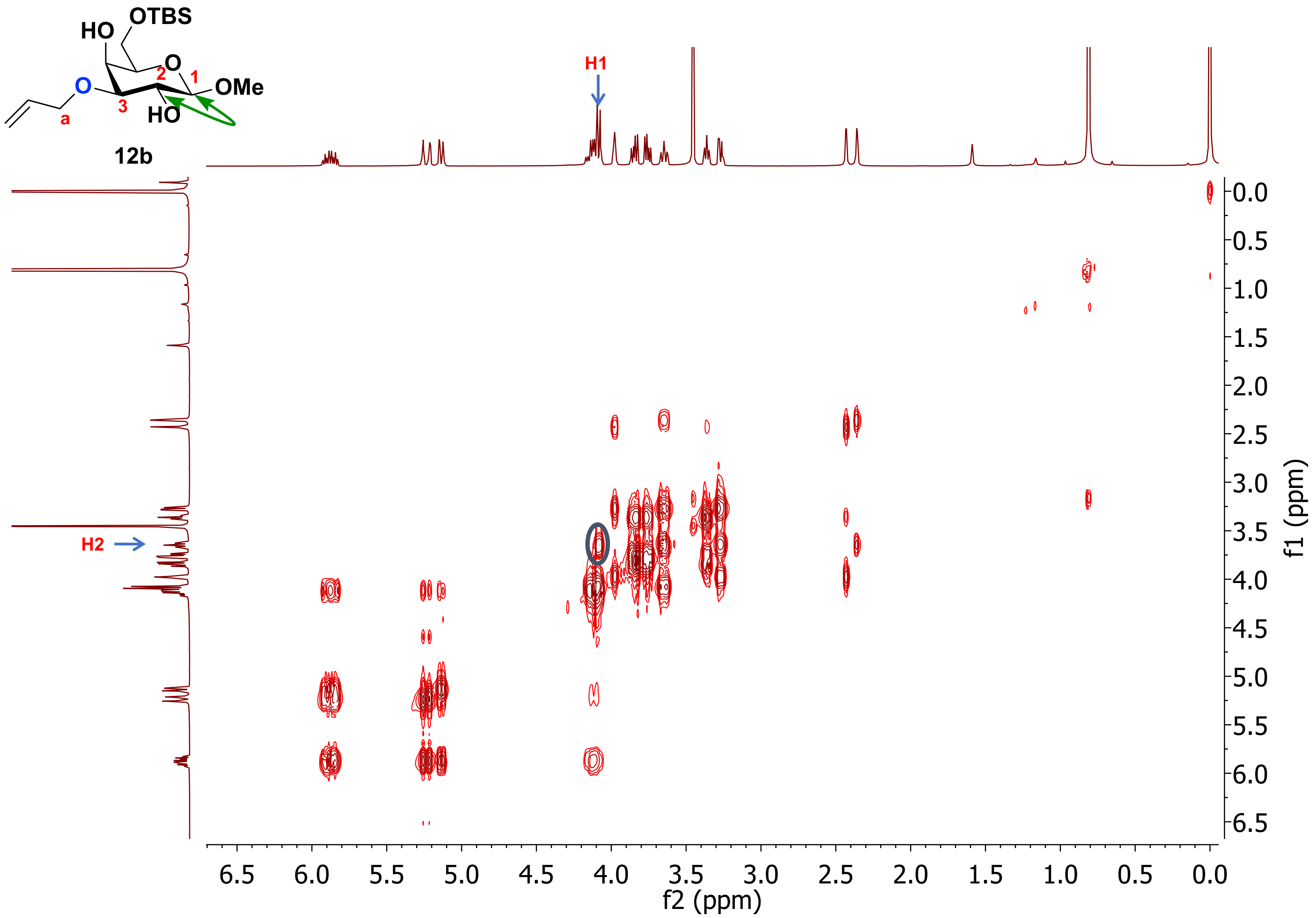
HO
OTBS
O
1
OMe
2
O
3
a
HO
12b
H1
H2
f1 (ppm)
f2 (ppm)
6.5
6.0
5.5
5.0
4.5
4.0
3.5
3.0
2.5
2.0
1.5
1.0
0.5
0.0

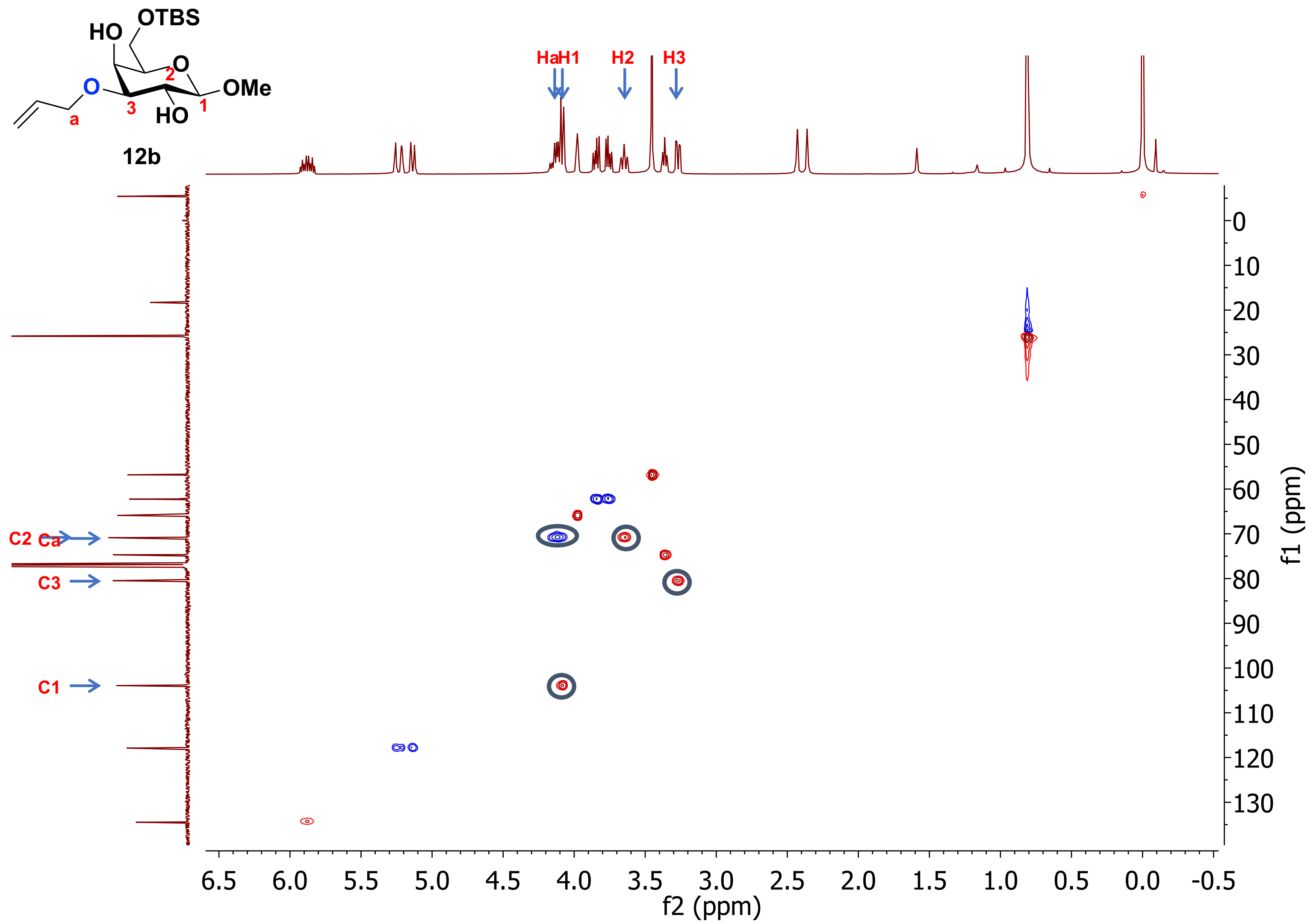
OTBS
HO
O
O
OMe
2
3
1
a
HO
12b
HaH1
H2
H3
C2 Ca
C3
C1
f1 (ppm)
f2 (ppm)

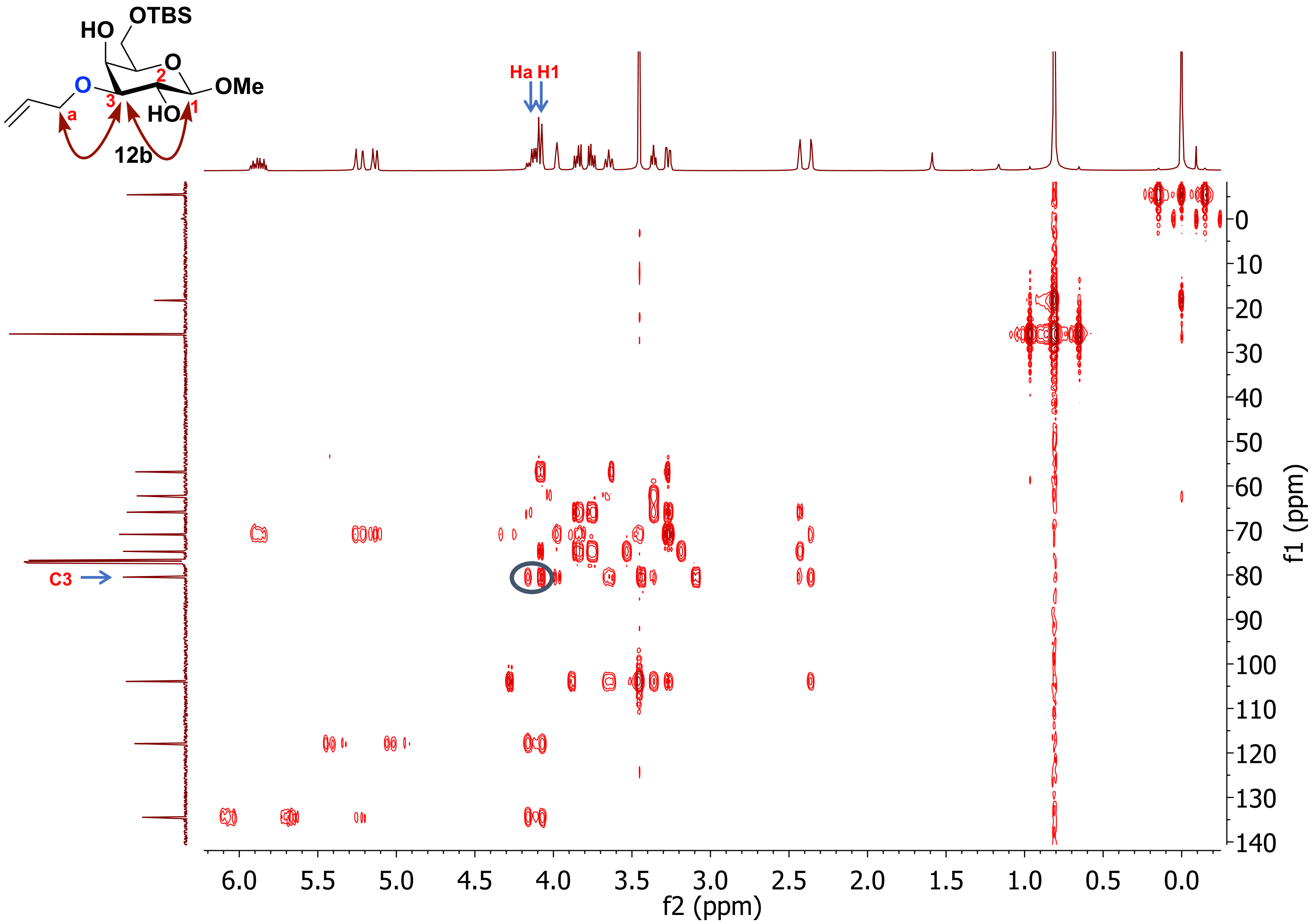

HO
OTBS
O
OMe
O
a
3
2
1
HO
12b
Ha H1
C3
f2 (ppm)
f1 (ppm)

CDCl3, 400.13 MHz

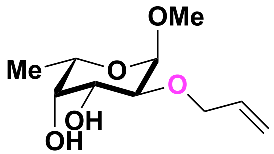

13a

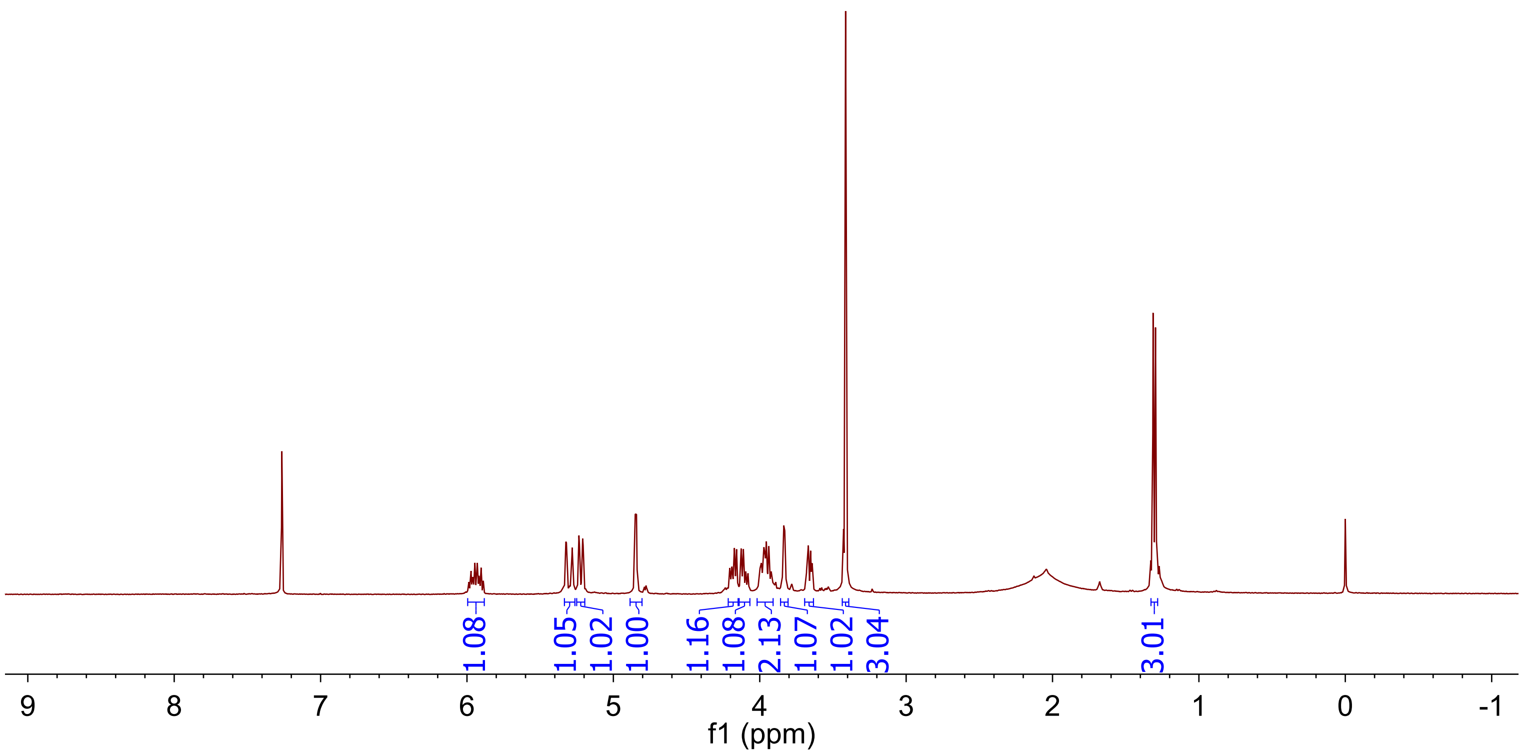

CDCl3, 100.62 MHz

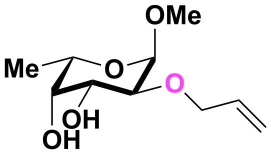

13a

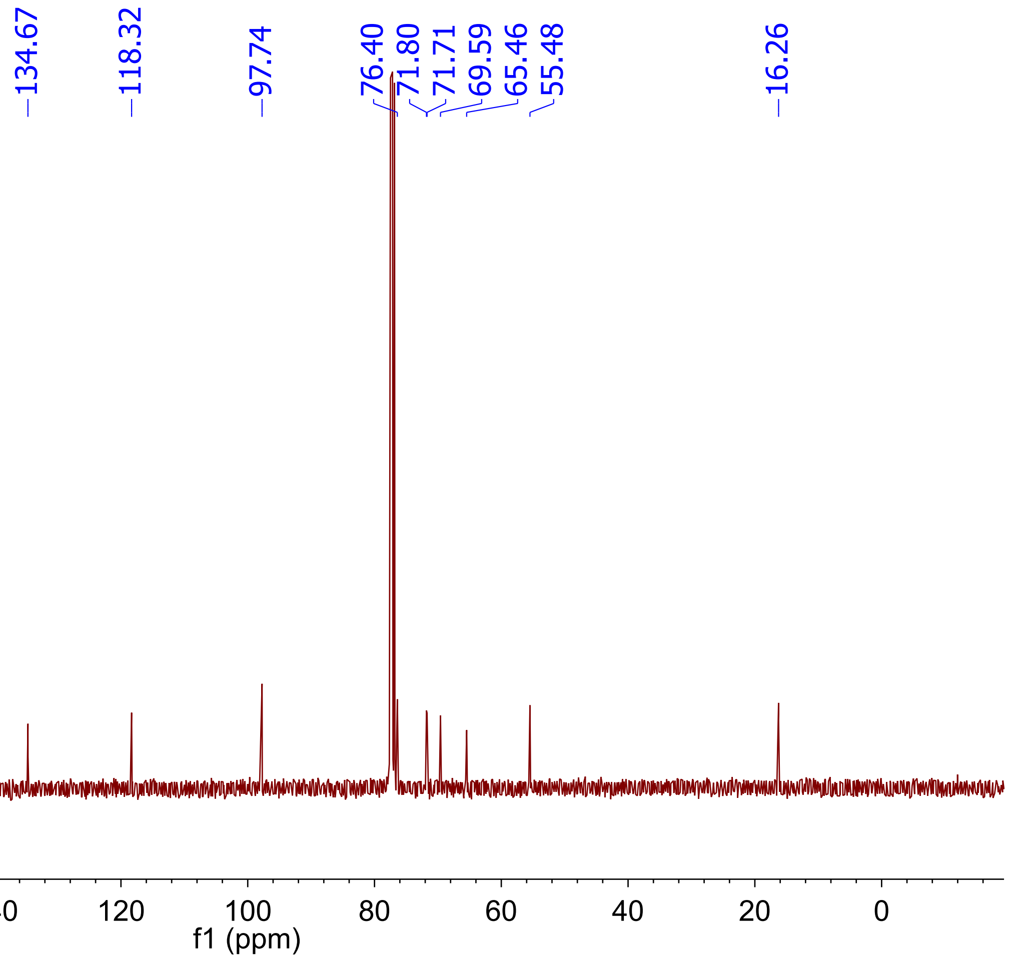

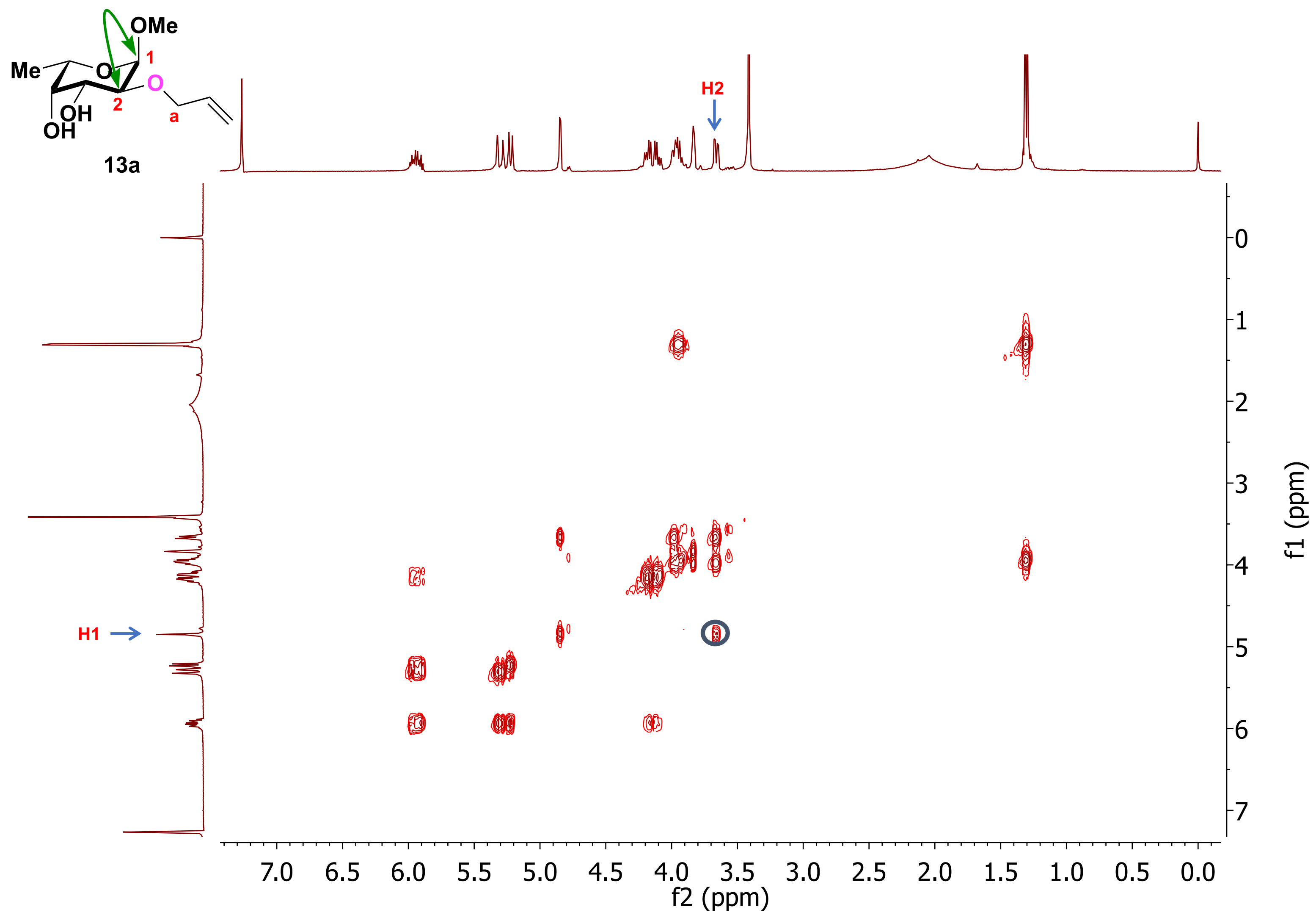
OMe
Me
O
1
2
O
a
OH
OH
13a
H2
H1
f2 (ppm)
f1 (ppm)

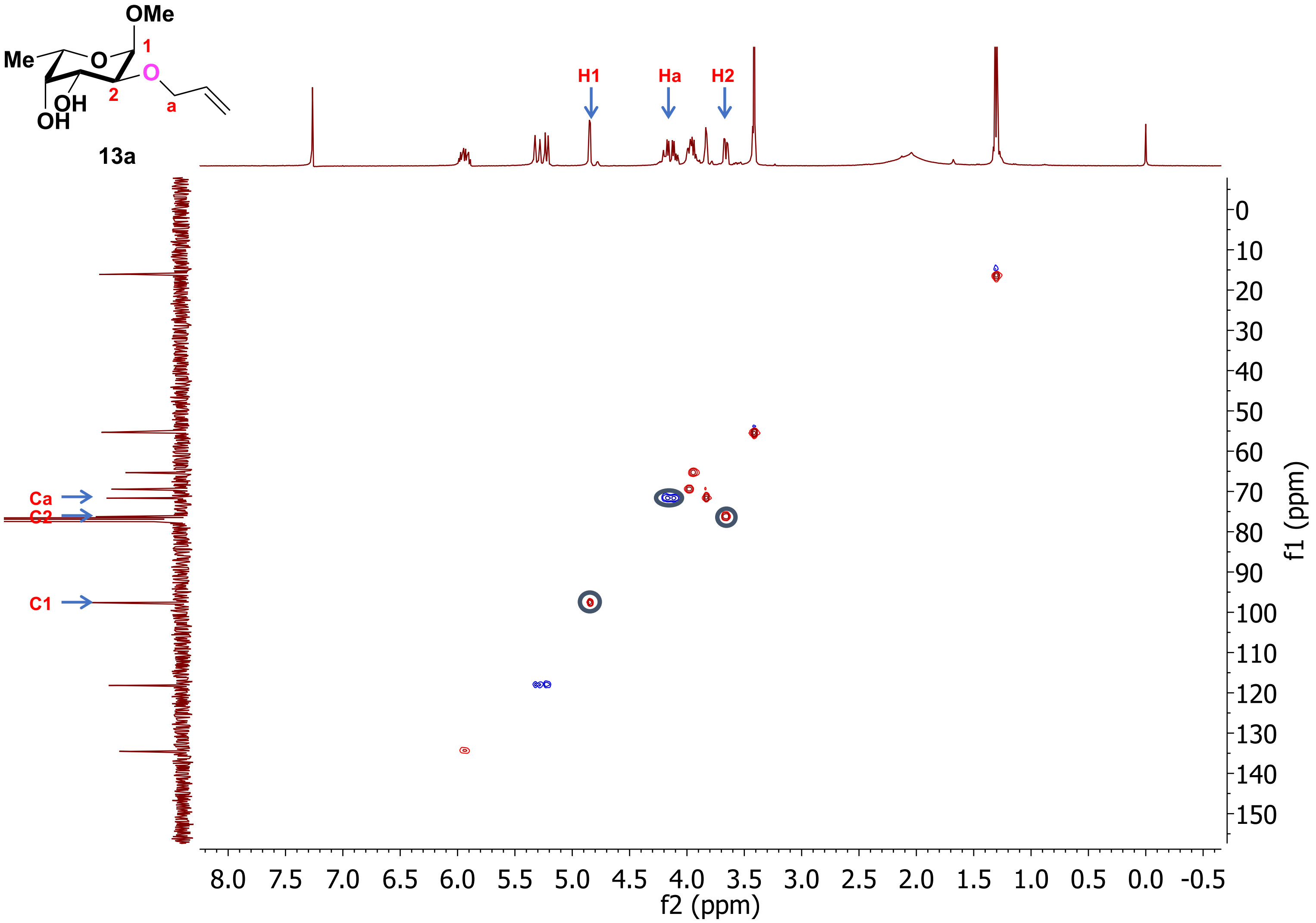

OMe
Me
O
1
2
O
a
OH
OH
13a
H1
Ha
H2
Ca
C2
C1
f2 (ppm)
f1 (ppm)

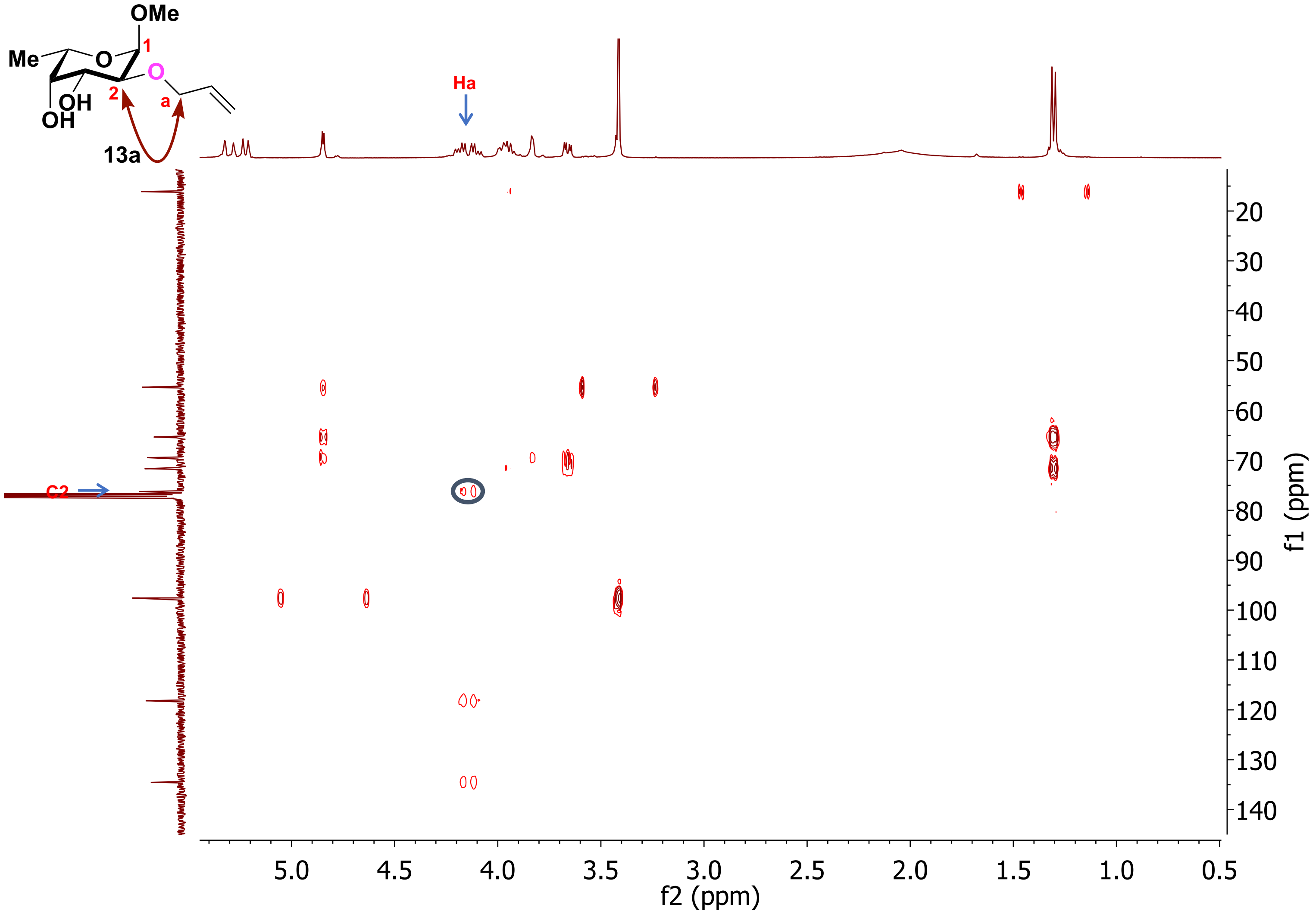
OMe
Me
O
1
O
2
a
OH
OH
13a
Ha
C2
20
30
40
50
60
70
80
90
100
110
120
130
140
f1 (ppm)
5.0
4.5
4.0
3.5
3.0
2.5
2.0
1.5
1.0
0.5
f2 (ppm)

CDCl3, 400.13 MHz

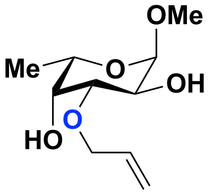

13b

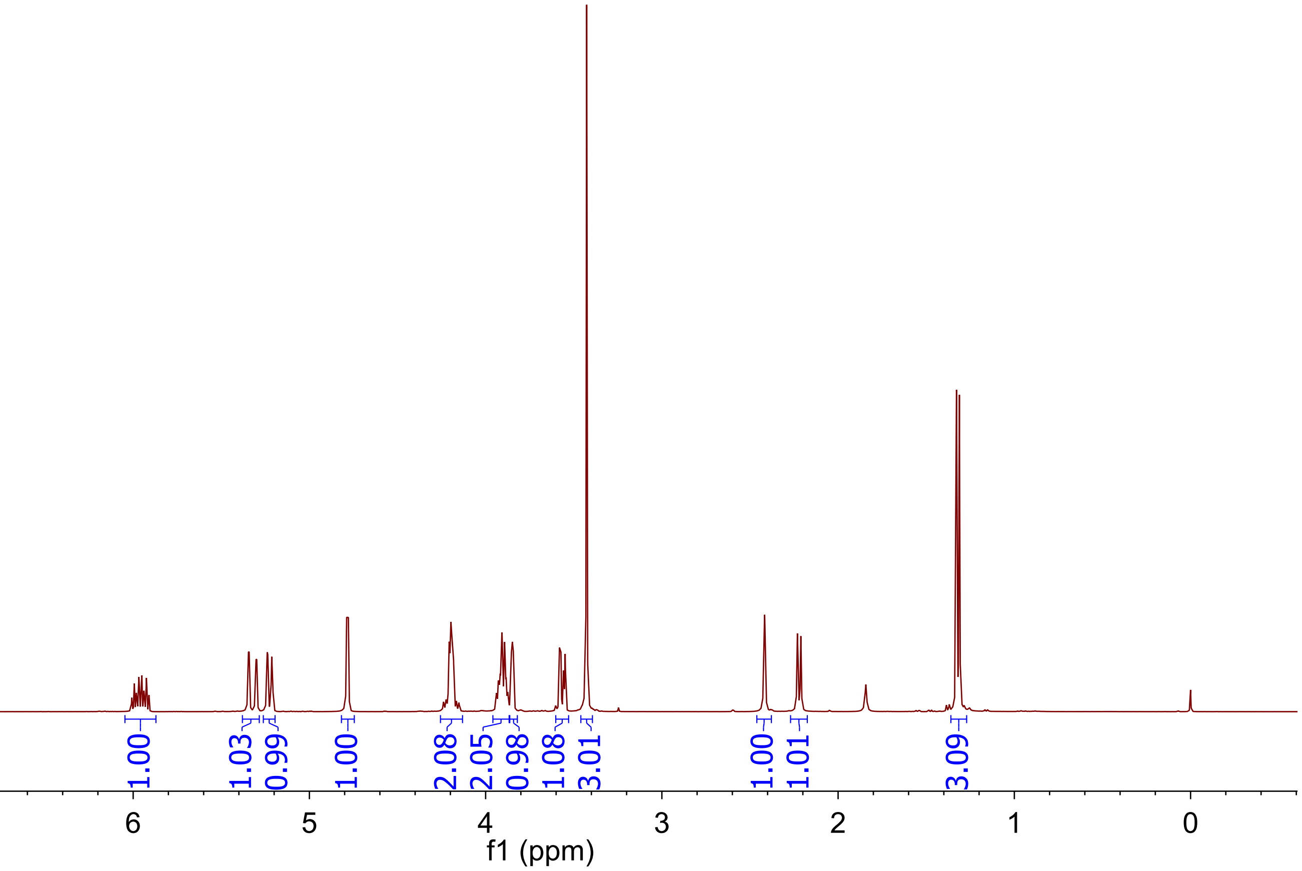

CDCl3, 100.62 MHz

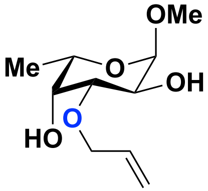

13b

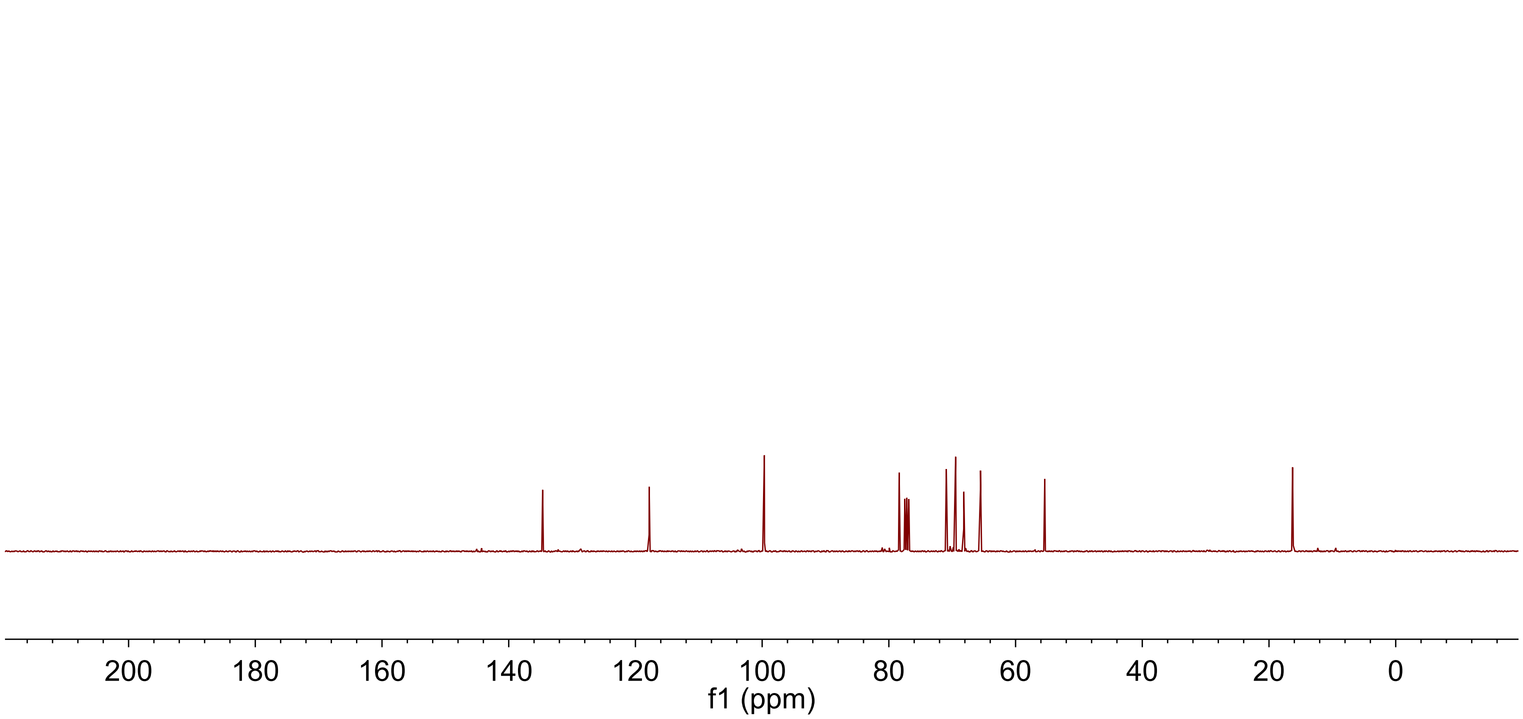

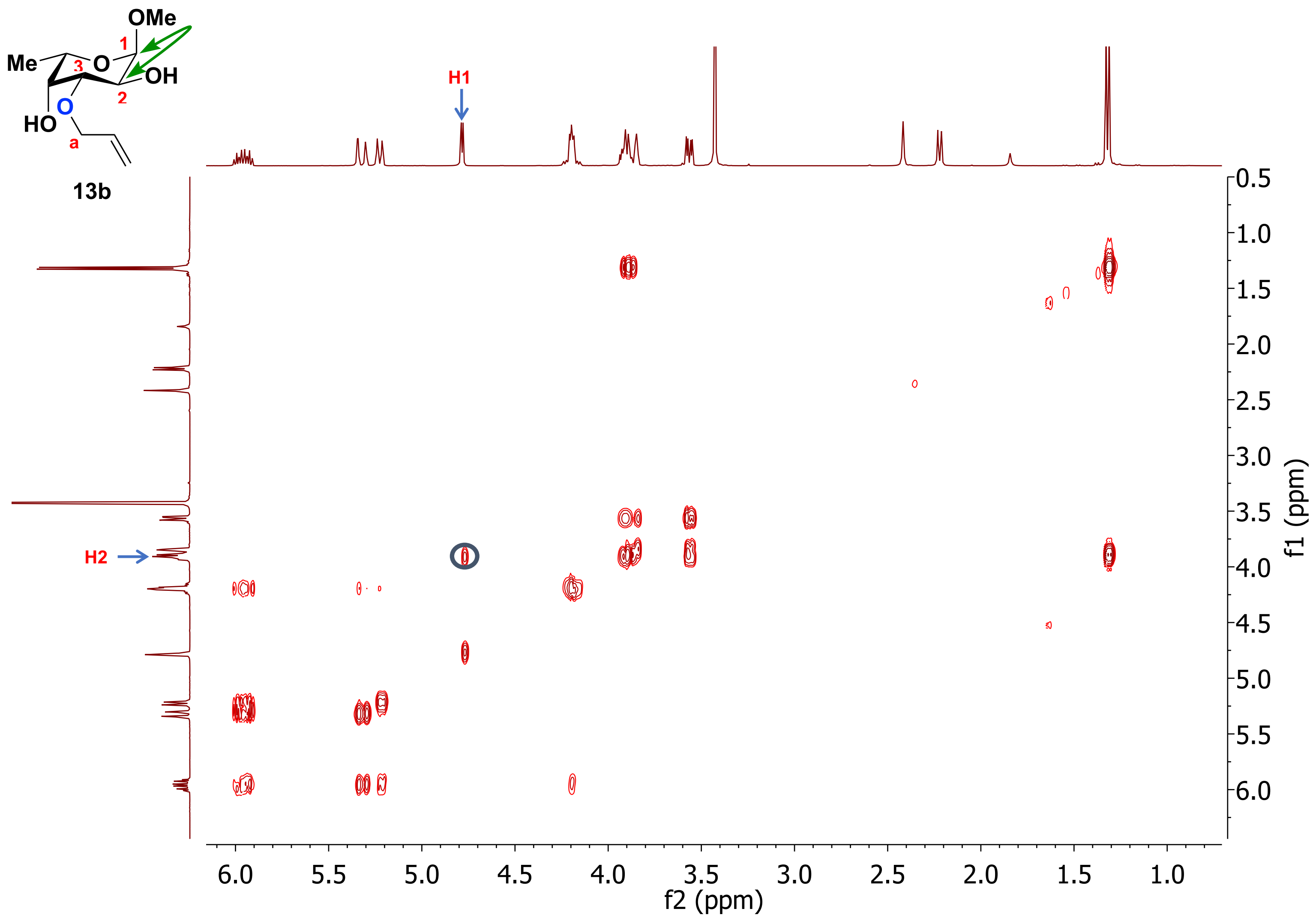
OMe
Me
O
OH
HO
O
1
2
3
a
13b
H1
H2
f2 (ppm)
f1 (ppm)

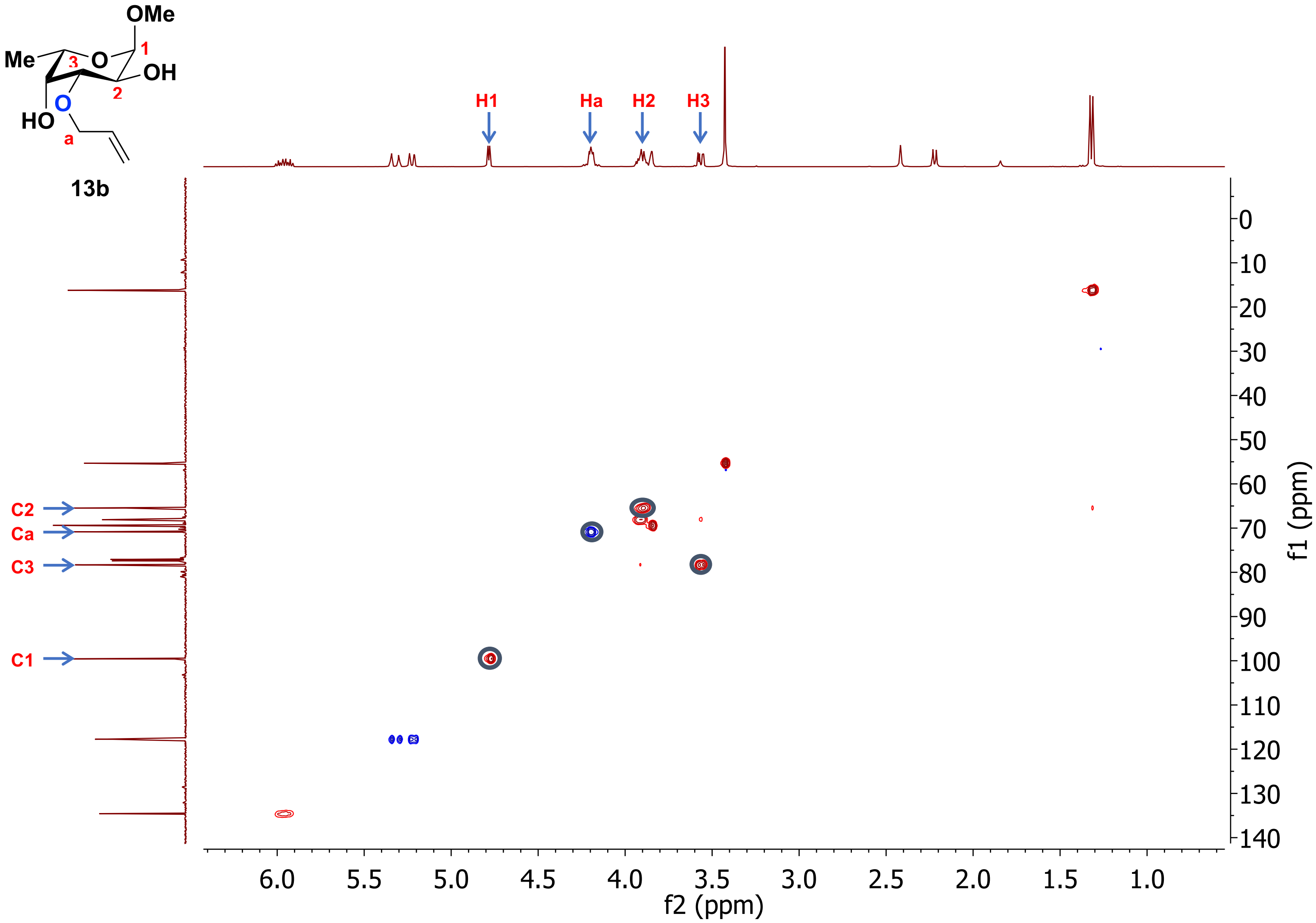

OMe
Me
O
OH
HO
O
1
2
3
a
13b
H1
Ha
H2
H3
C2
Ca
C3
C1
f2 (ppm)
f1 (ppm)

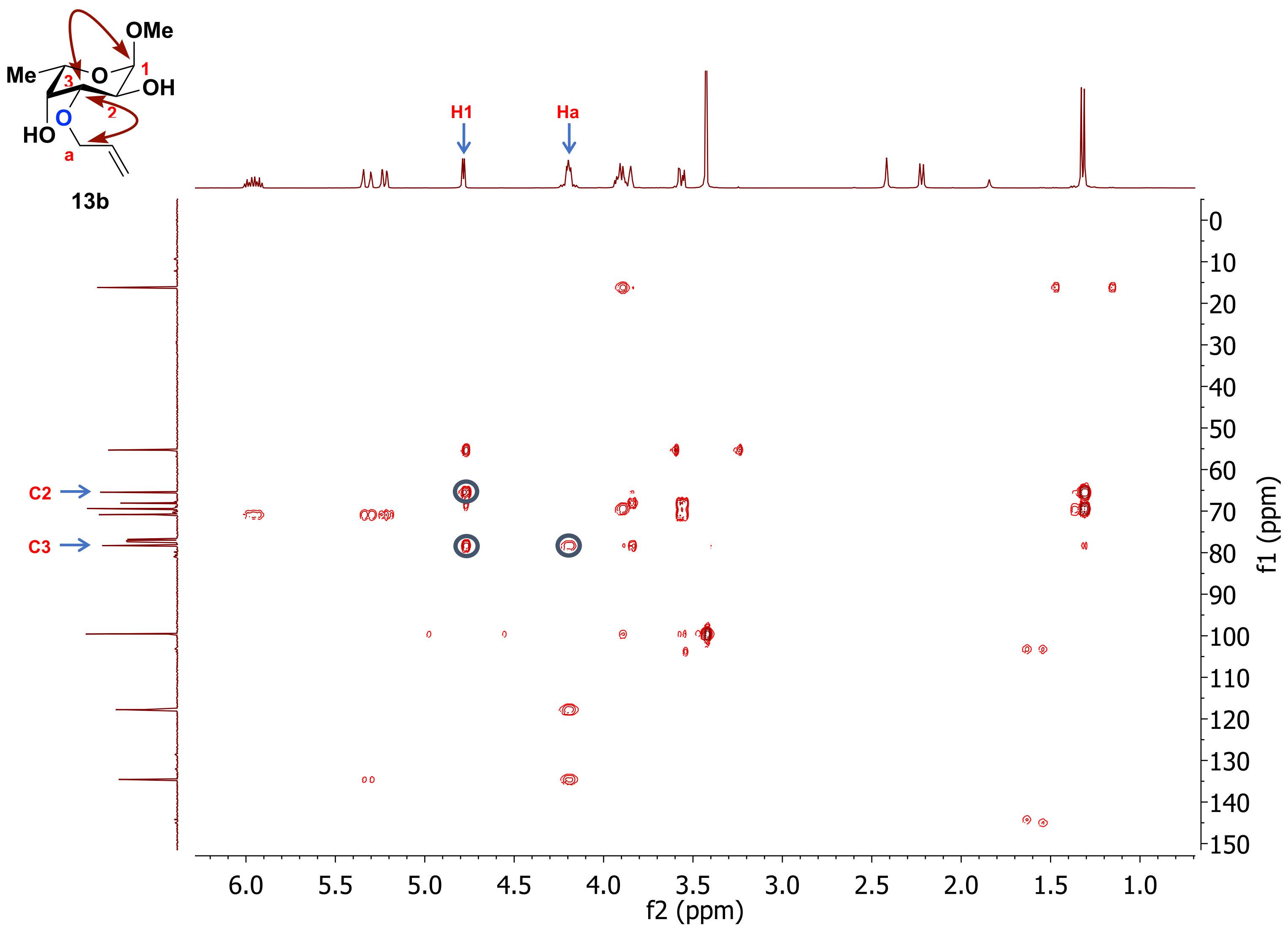
OMe
Me
O
1
OH
3
2
O
HO
a
13b
H1
Ha
C2
C3
6.0
5.5
5.0
4.5
4.0
3.5
3.0
2.5
2.0
1.5
1.0
f2 (ppm)
0
10
20
30
40
50
60
70
80
90
100
110
120
130
140
150
f1 (ppm)

CDCl3, 400.13 MHz

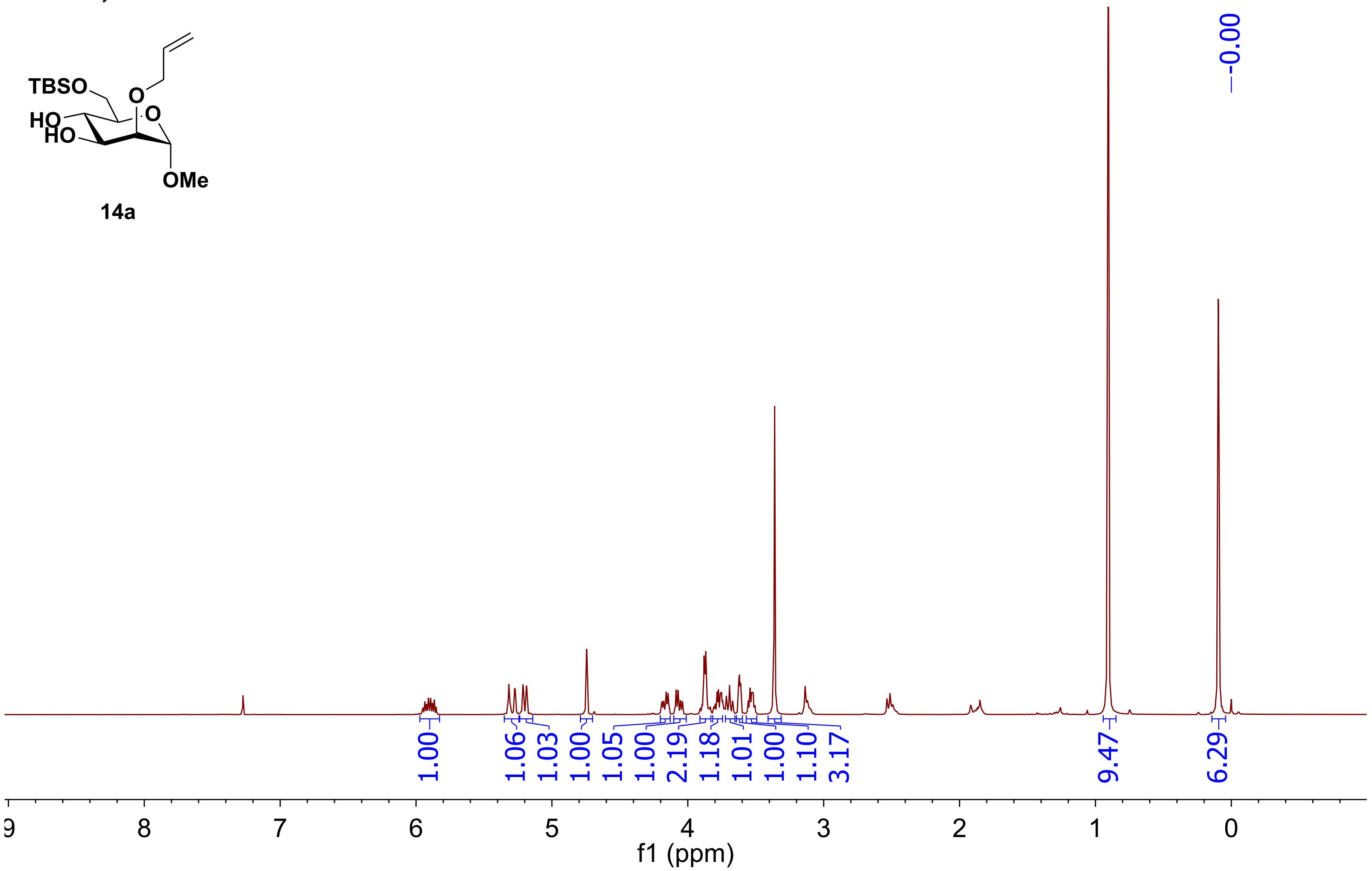

CDCl3, 100.62 MHz

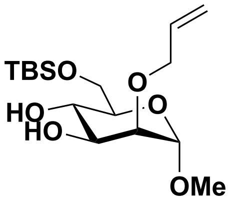

14a

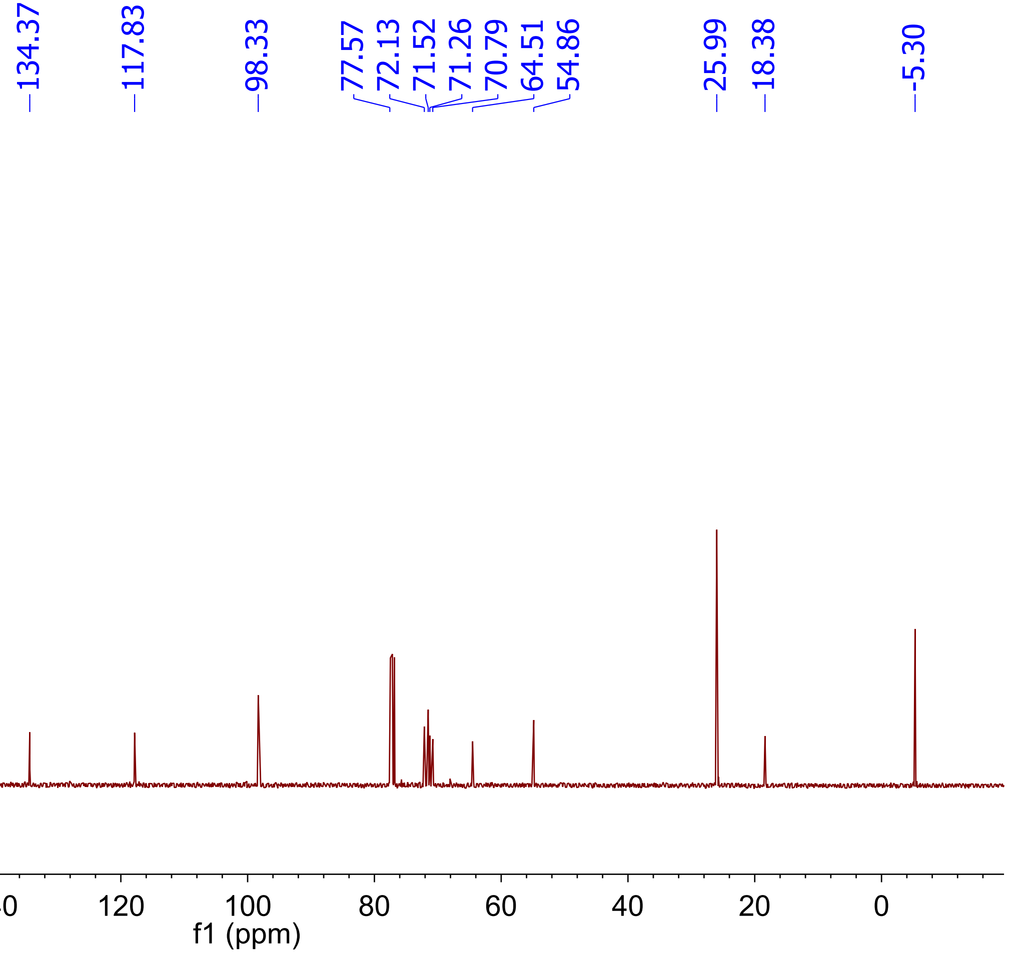

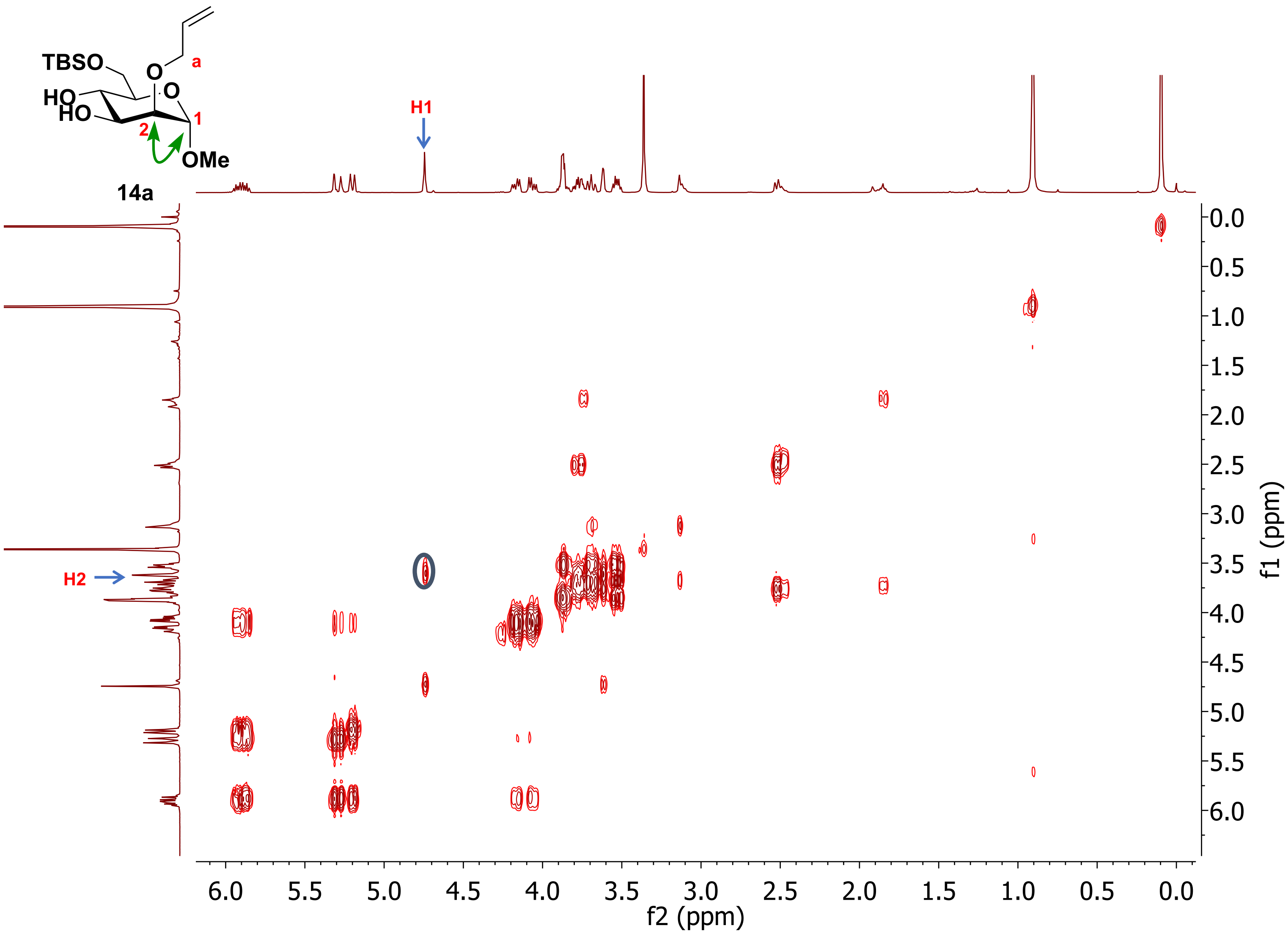
TBSO
O
a
O
HO
HO
1
2
OMe
14a
H1
H2
6.0
5.5
5.0
4.5
4.0
3.5
3.0
2.5
2.0
1.5
1.0
0.5
0.0
f2 (ppm)
f1 (ppm)

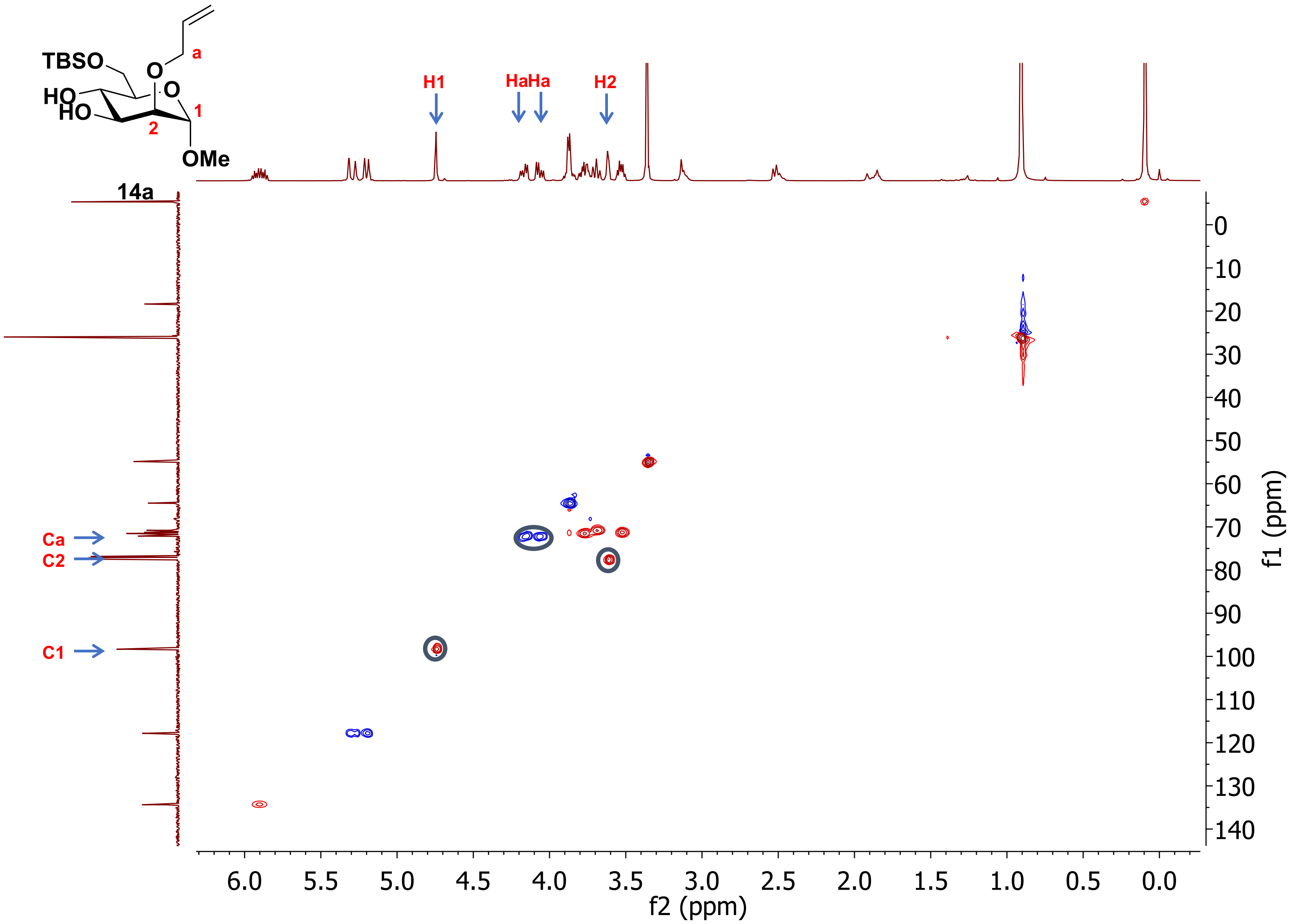
TBSO
HO
HO
O
O
a
1
2
OMe
14a
H1
HaHa
H2
Ca
C2
C1
f2 (ppm)
f1 (ppm)

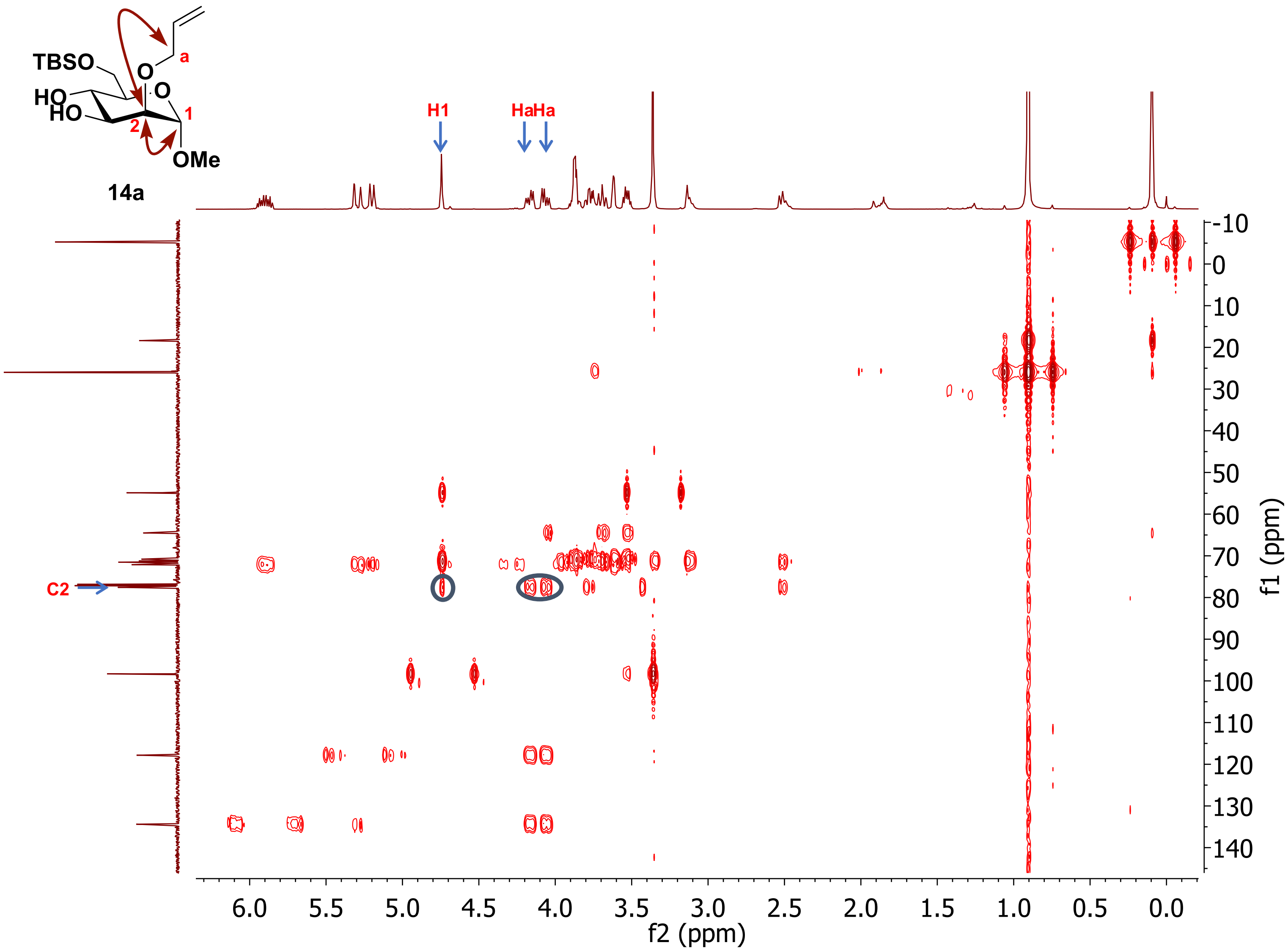

TBSO
HO
HO
O
O
a
1
2
OMe
14a
H1
HaHa
C2
f2 (ppm)
f1 (ppm)

CDCl3, 400 MHz

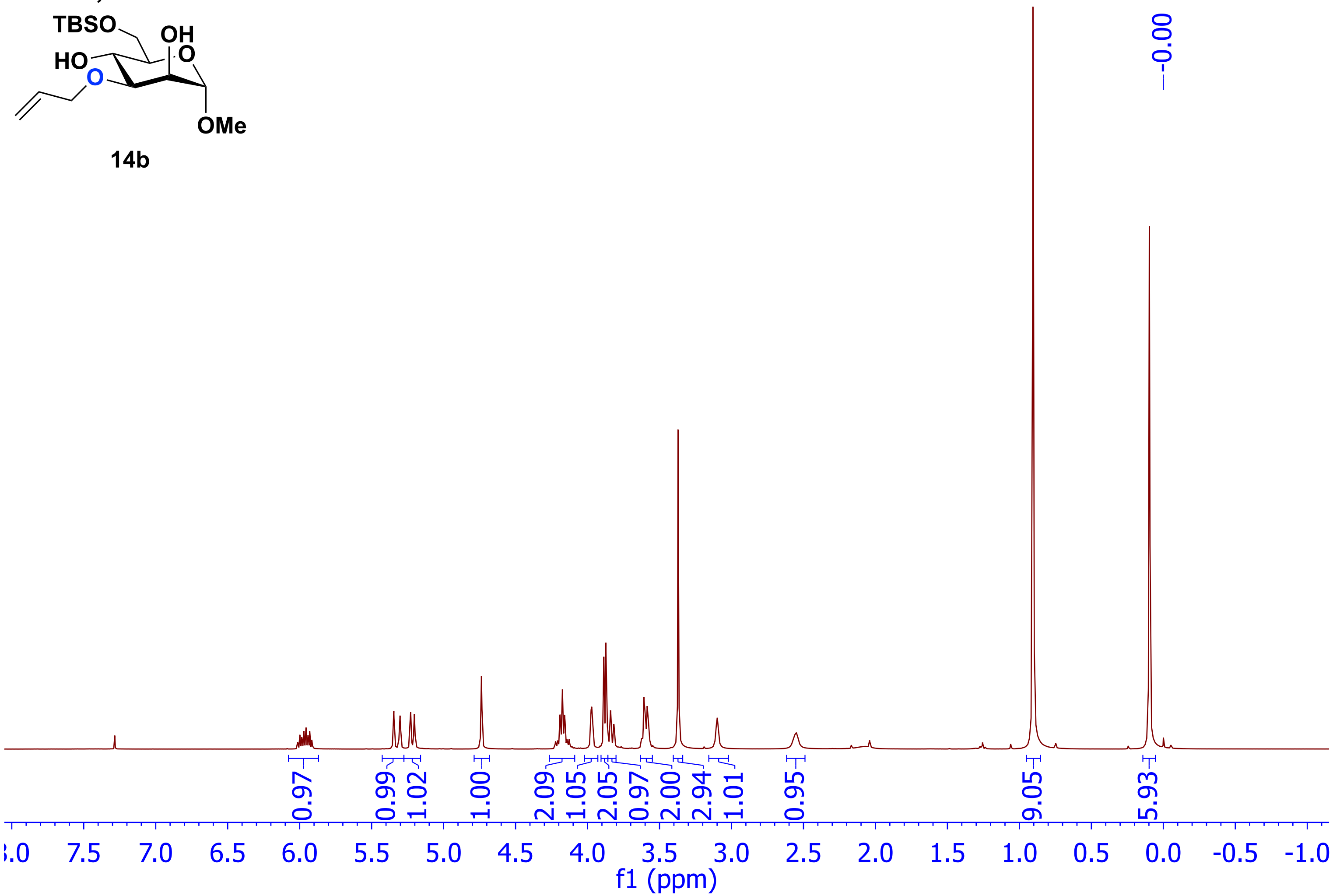

CDCl3, 101 MHz

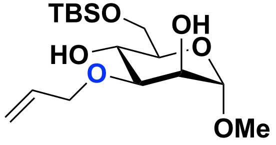

14b

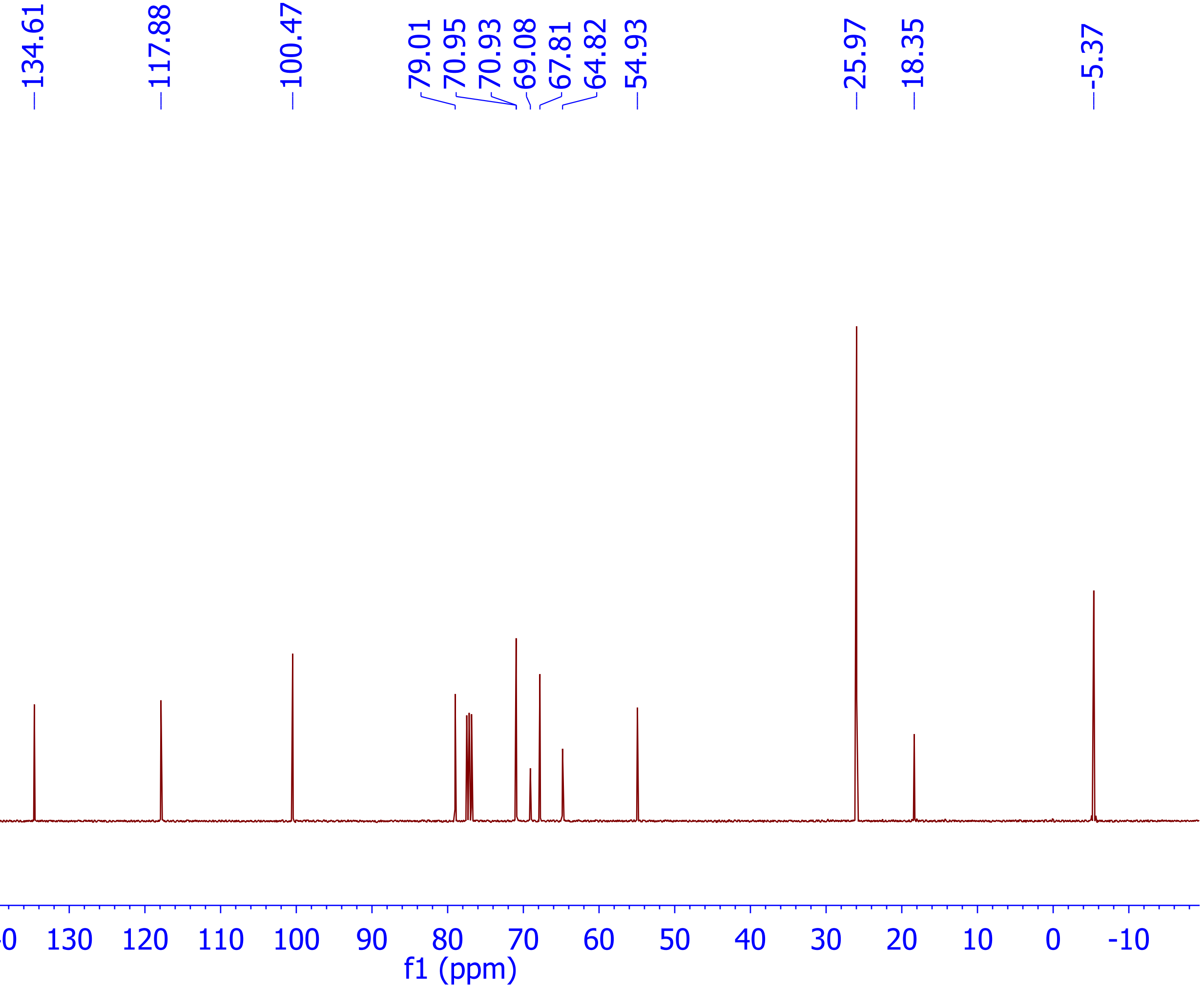

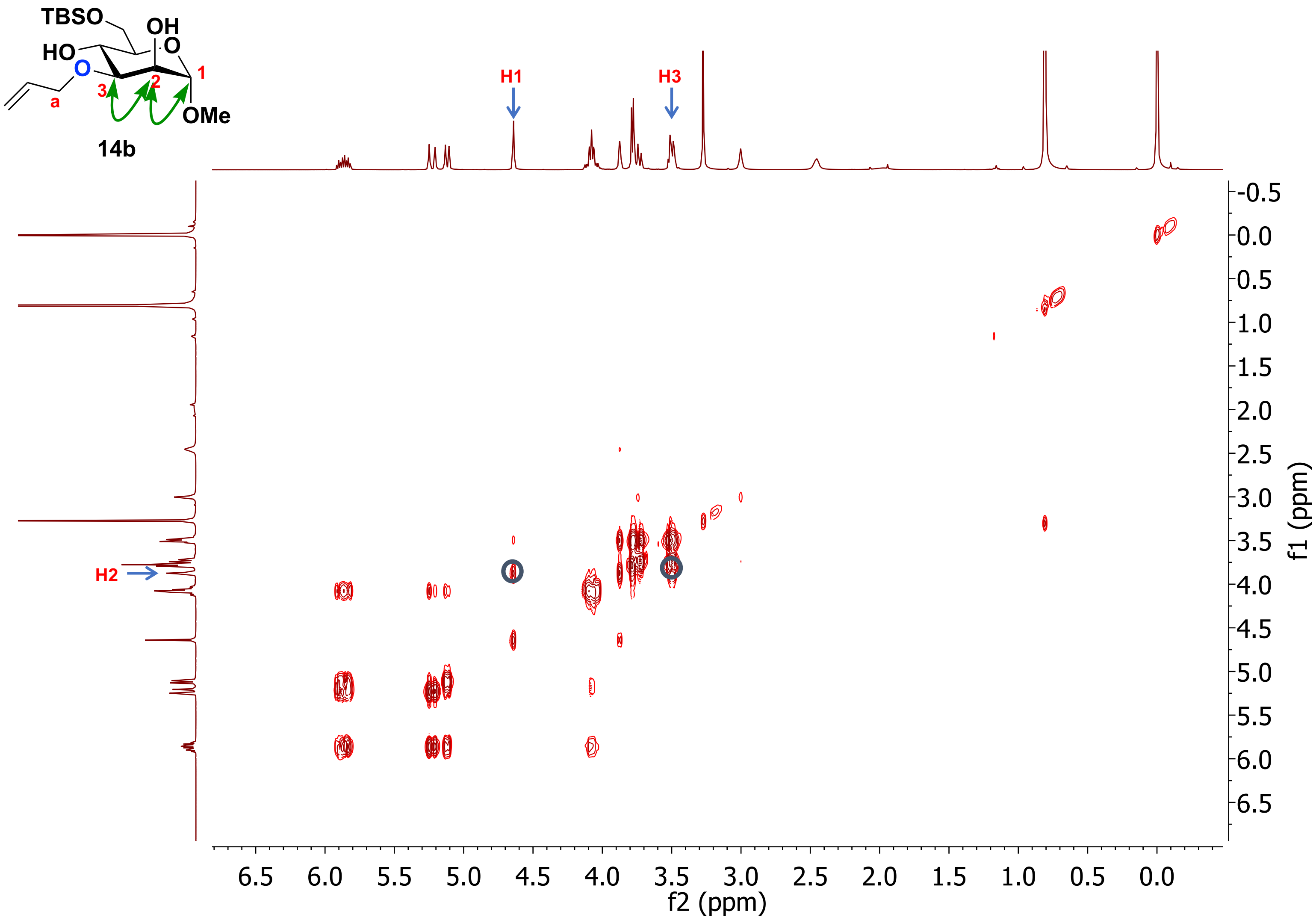
TBSO
OH
HO
O
O
1
2
3
a
OMe
14b
H1
H3
H2
-0.5
0.0
0.5
1.0
1.5
2.0
2.5
3.0
3.5
4.0
4.5
5.0
5.5
6.0
6.5
f1 (ppm)
6.5
6.0
5.5
5.0
4.5
4.0
3.5
3.0
2.5
2.0
1.5
1.0
0.5
0.0
f2 (ppm)

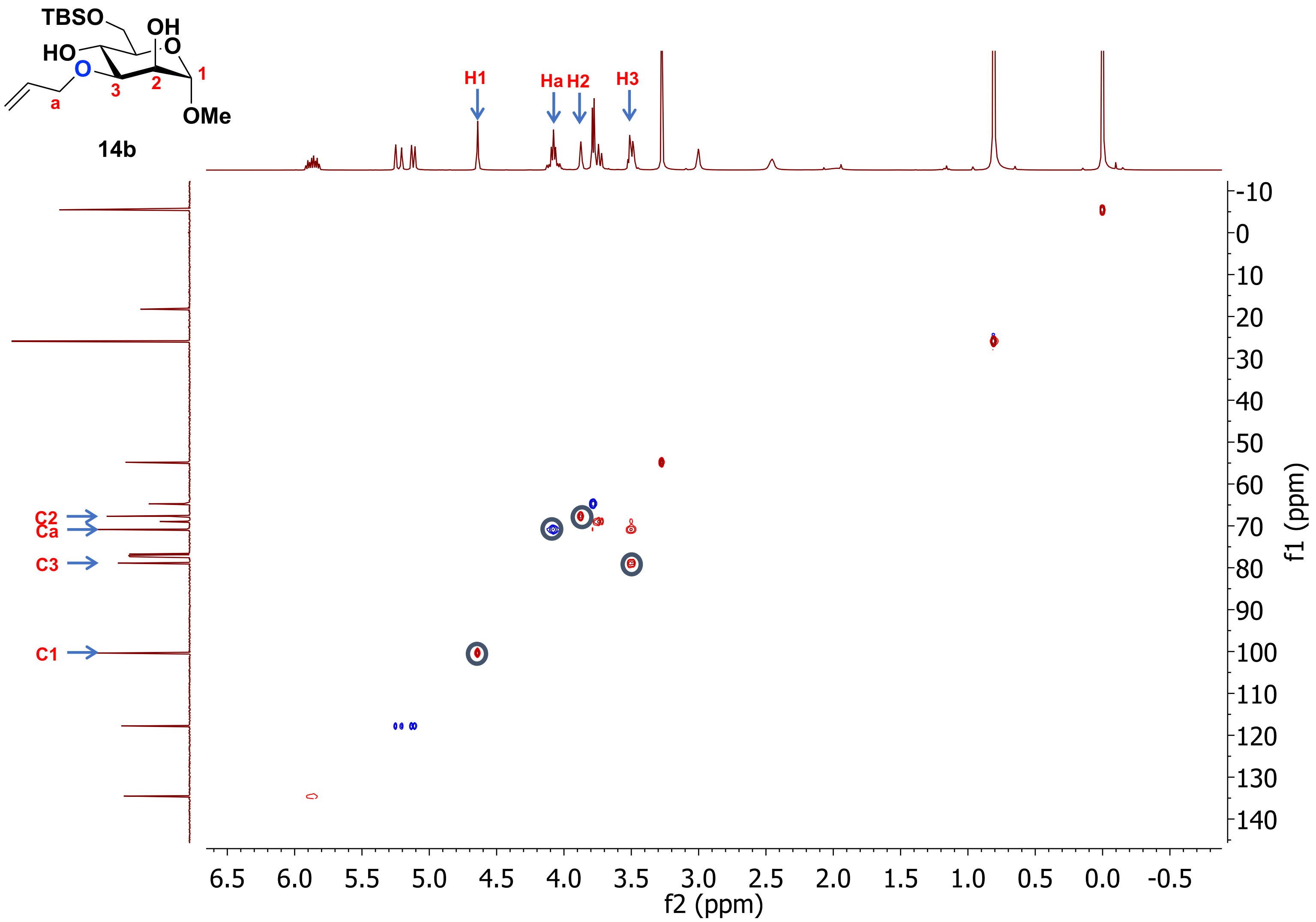
TBSO
OH
HO
O
O
1
3
2
a
OMe
14b
H1
Ha H2
H3
C2
Ca
C3
C1
-10
0
10
20
30
40
50
60
70
80
90
100
110
120
130
140
f1 (ppm)
6.5 6.0 5.5 5.0 4.5 4.0 3.5 3.0 2.5 2.0 1.5 1.0 0.5 0.0 -0.5
f2 (ppm)

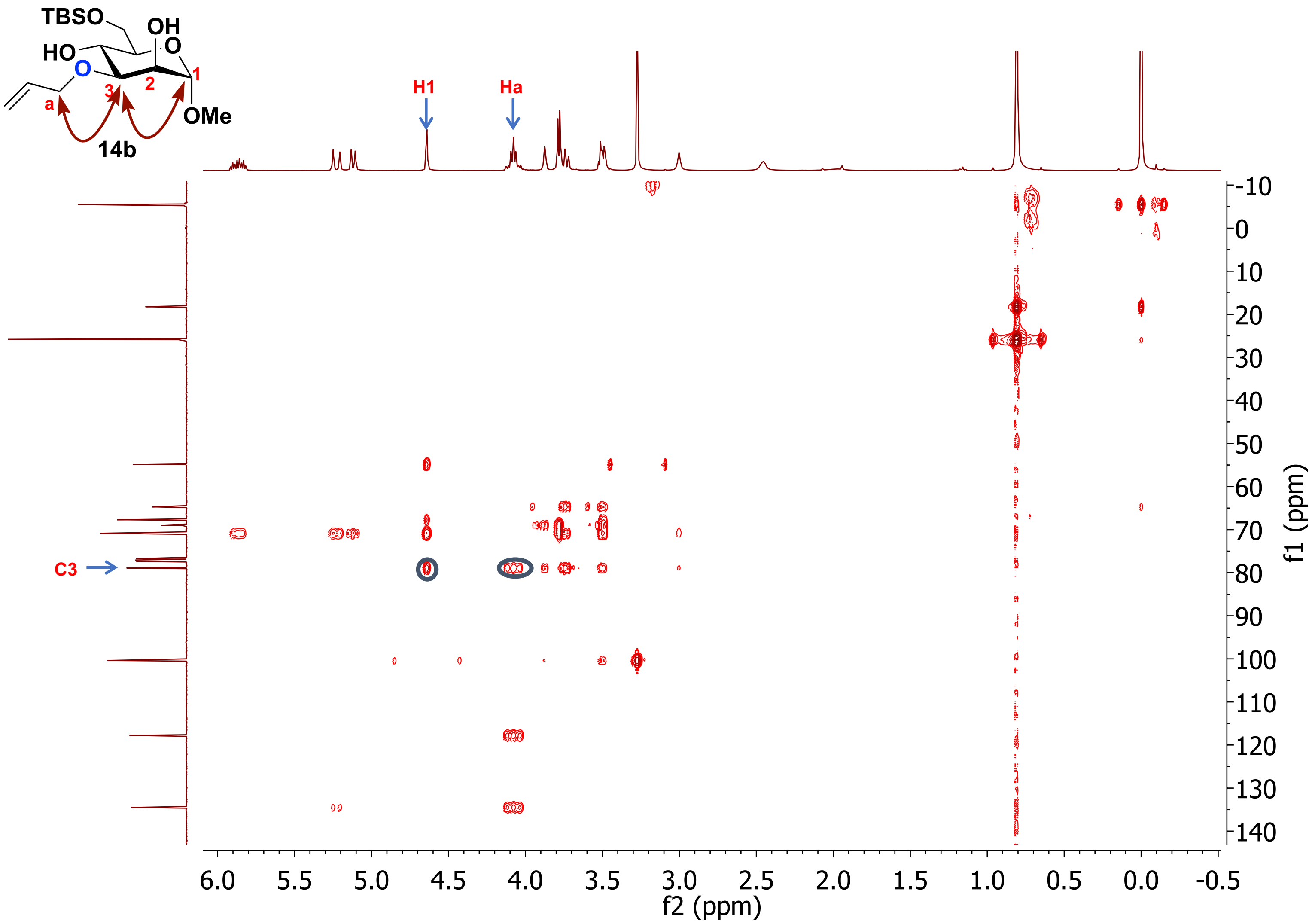

TBSO
OH
HO
O
O
1
2
3
a
OMe
14b
H1
Ha
C3
f1 (ppm)
f2 (ppm)

CDCl3, 400.13 MHz

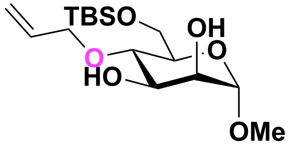

14c

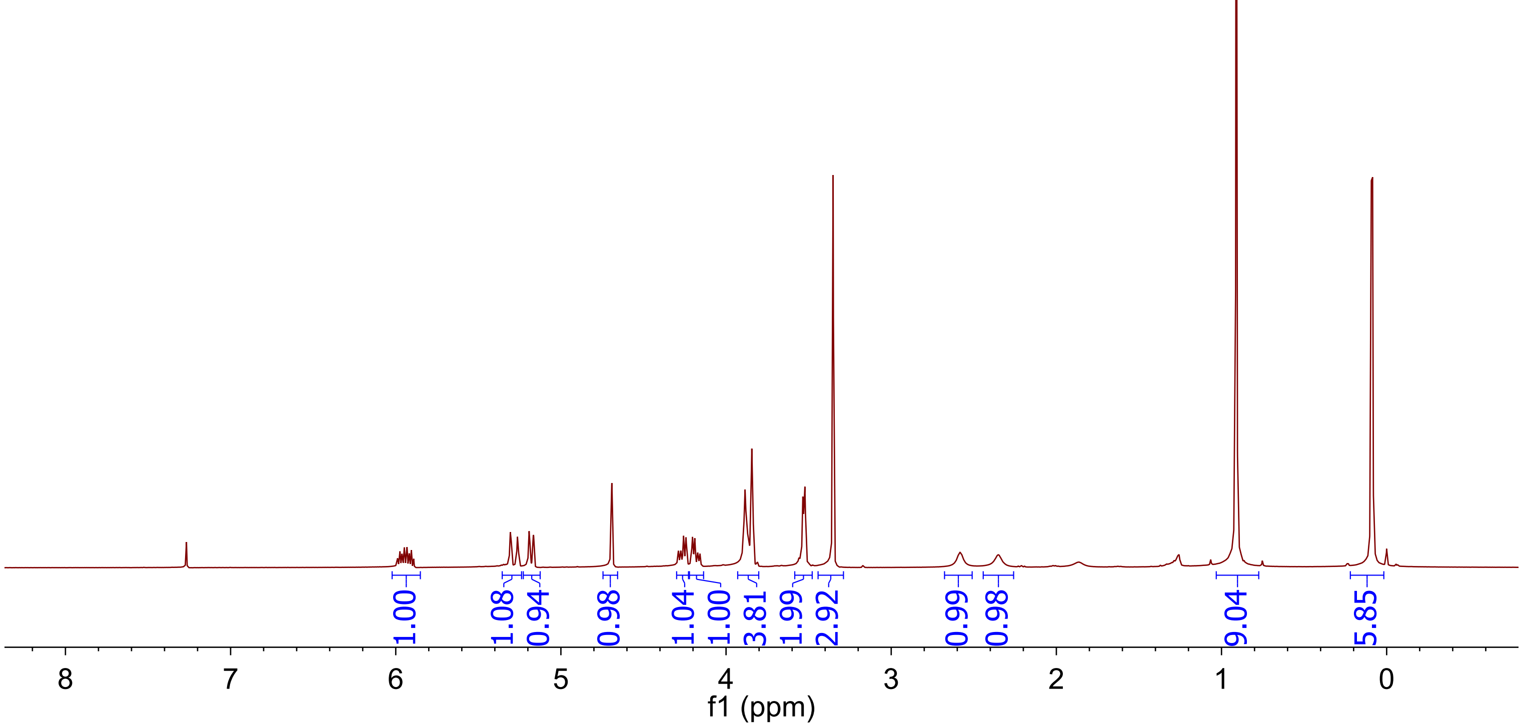

CDCl3, 100.62 MHz

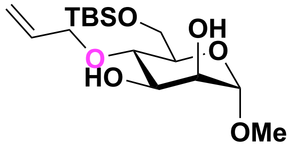

14c

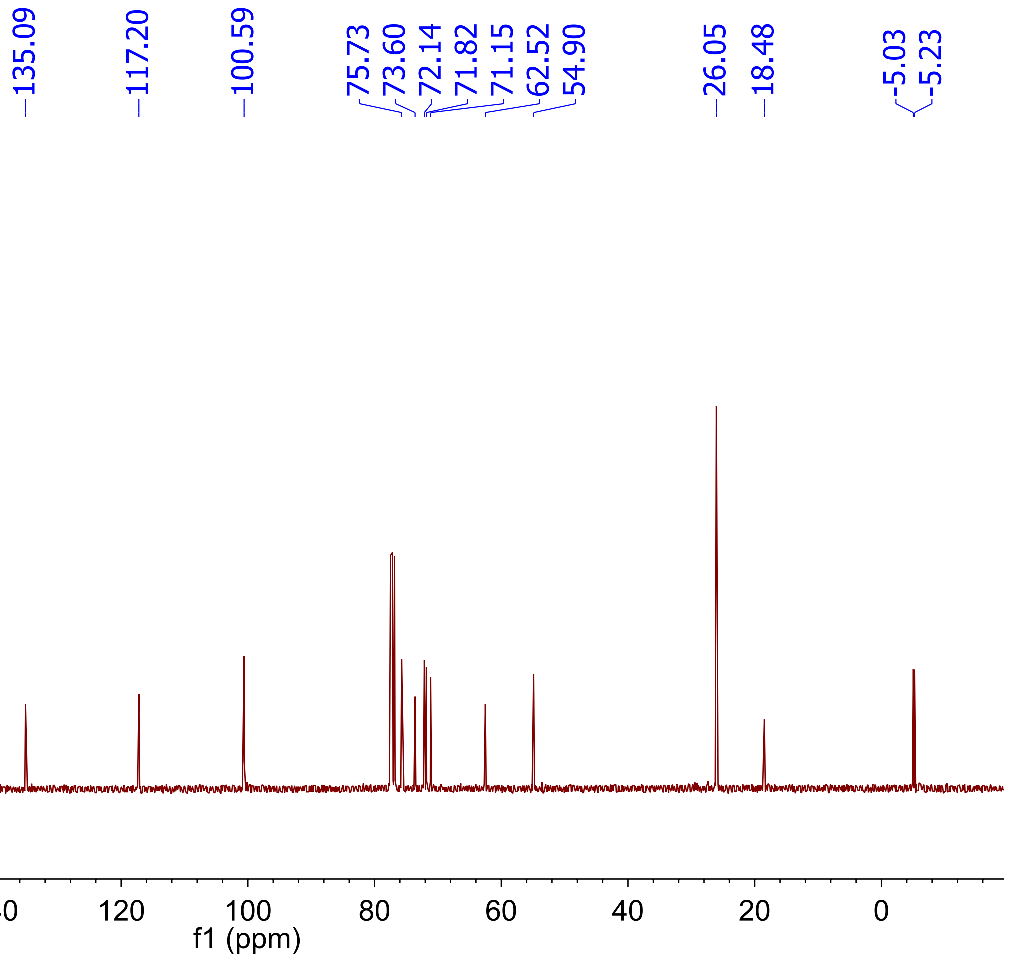

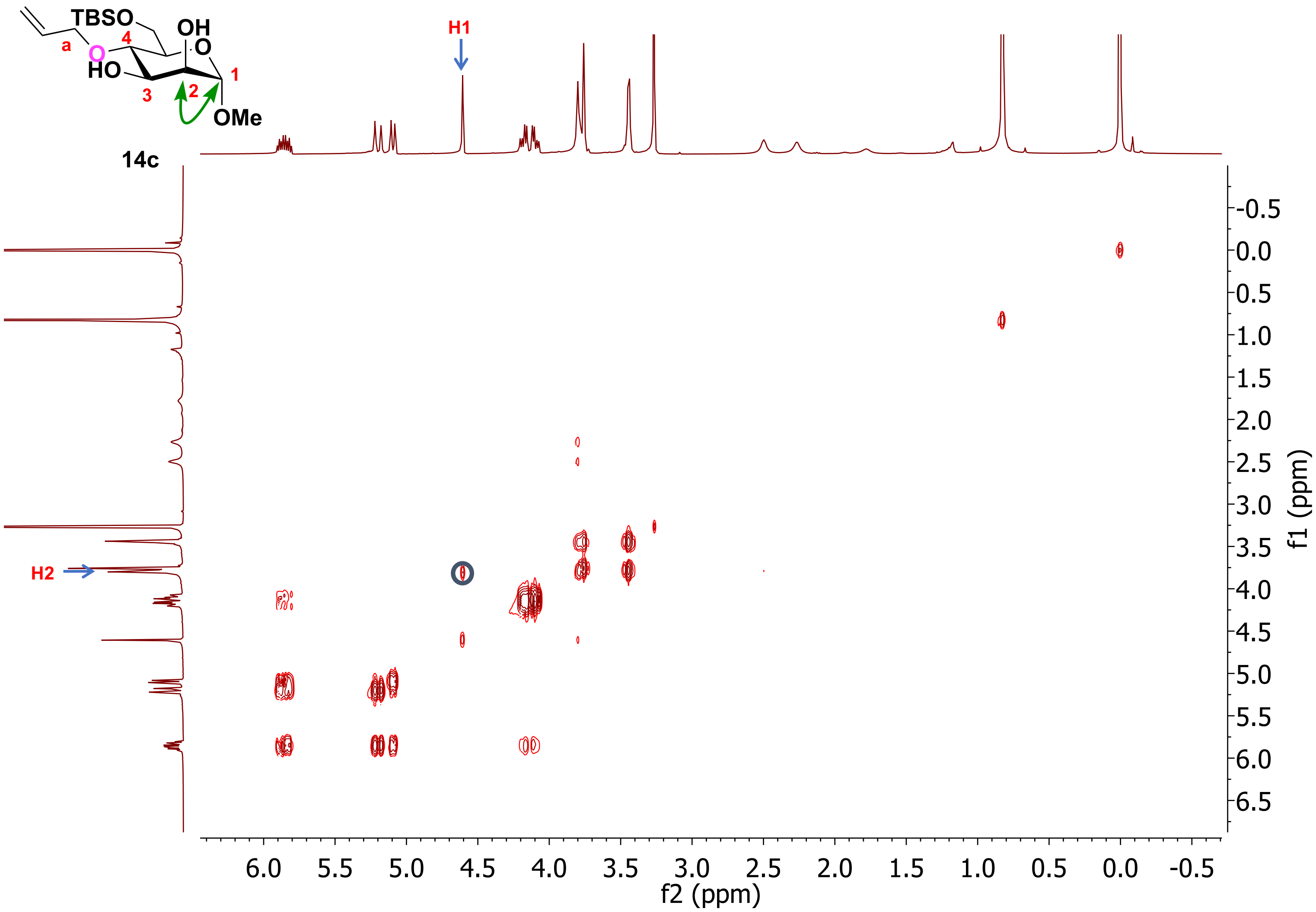
TBSO
OH
O
a
4
HO
1
3
2
OMe
14c
H1
H2
f1 (ppm)
f2 (ppm)

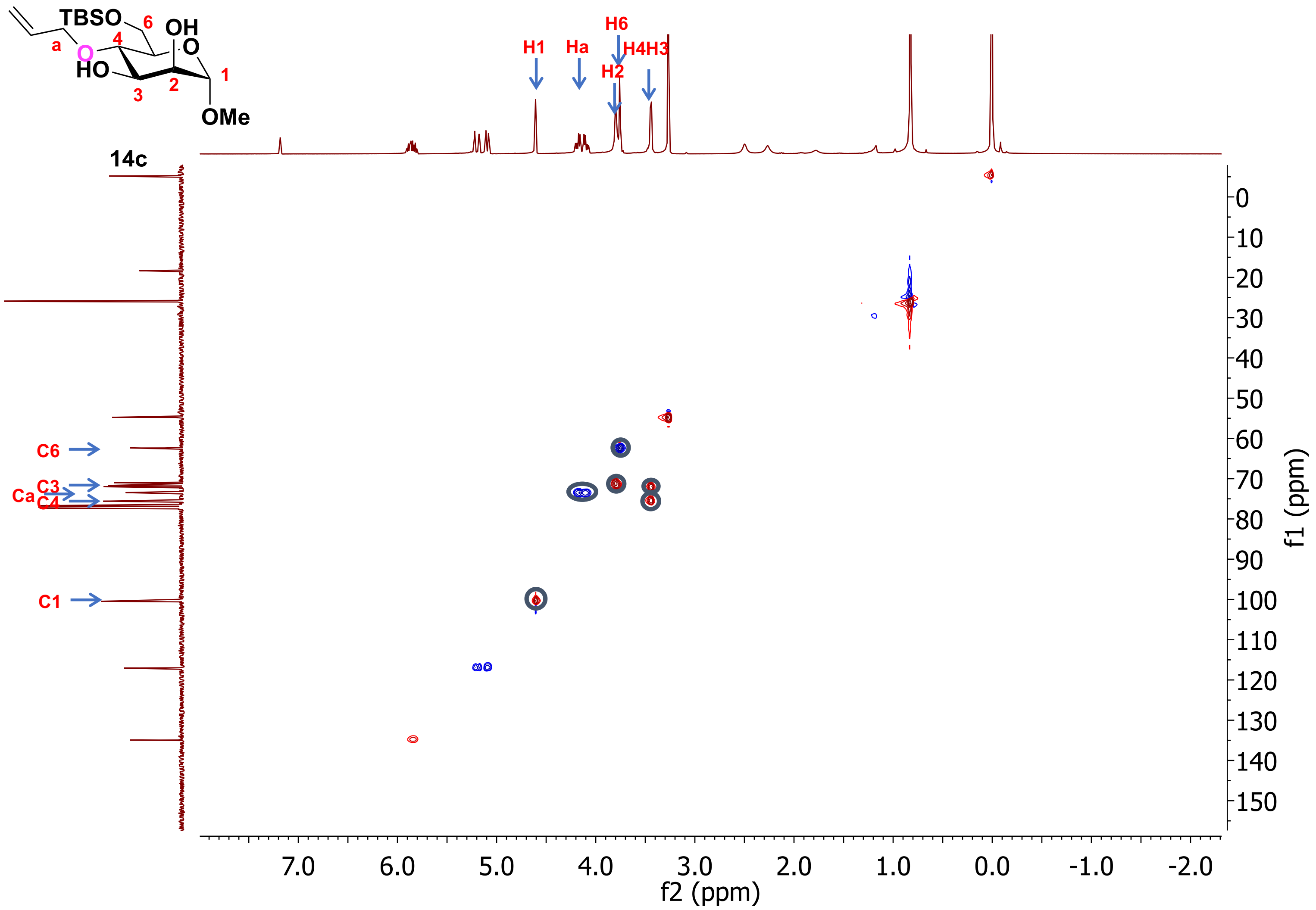

TBSO
OH
O
a
O
4
6
HO
3
2
1
OMe
14c
H1
Ha
H6
H2
H4H3
C6
C3
Ca
C4
C1
f2 (ppm)
f1 (ppm)

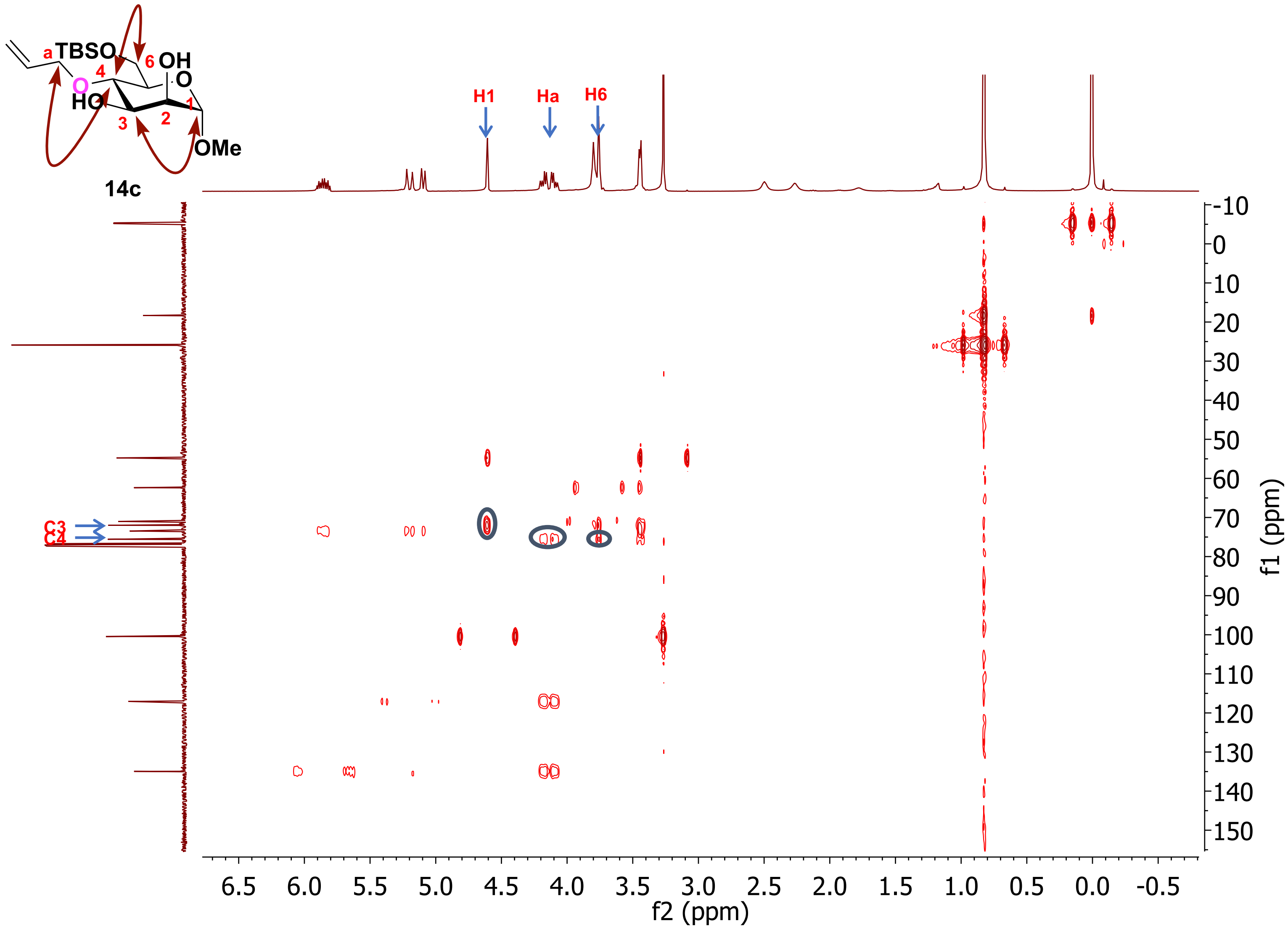

TBSO
OH
O
HO
OMe
a
6
4
1
2
3
14c
H1
Ha
H6
C3
C4
f2 (ppm)
f1 (ppm)

CDCl3, 400.13 MHz

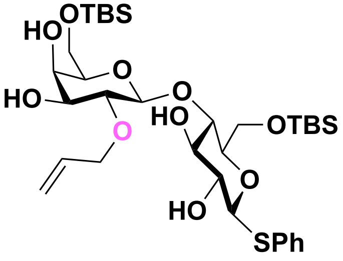

15a

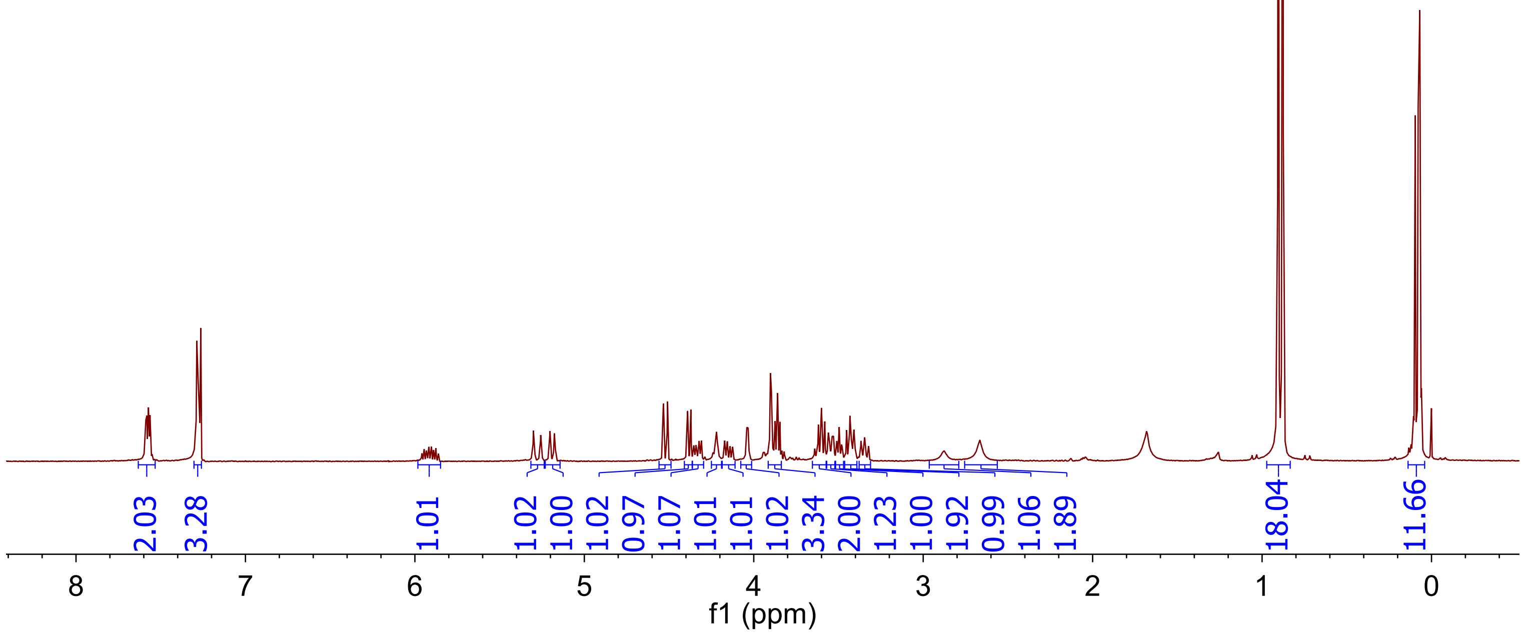

CDCl3, 100.62 MHz

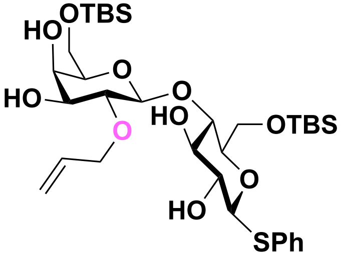

15a

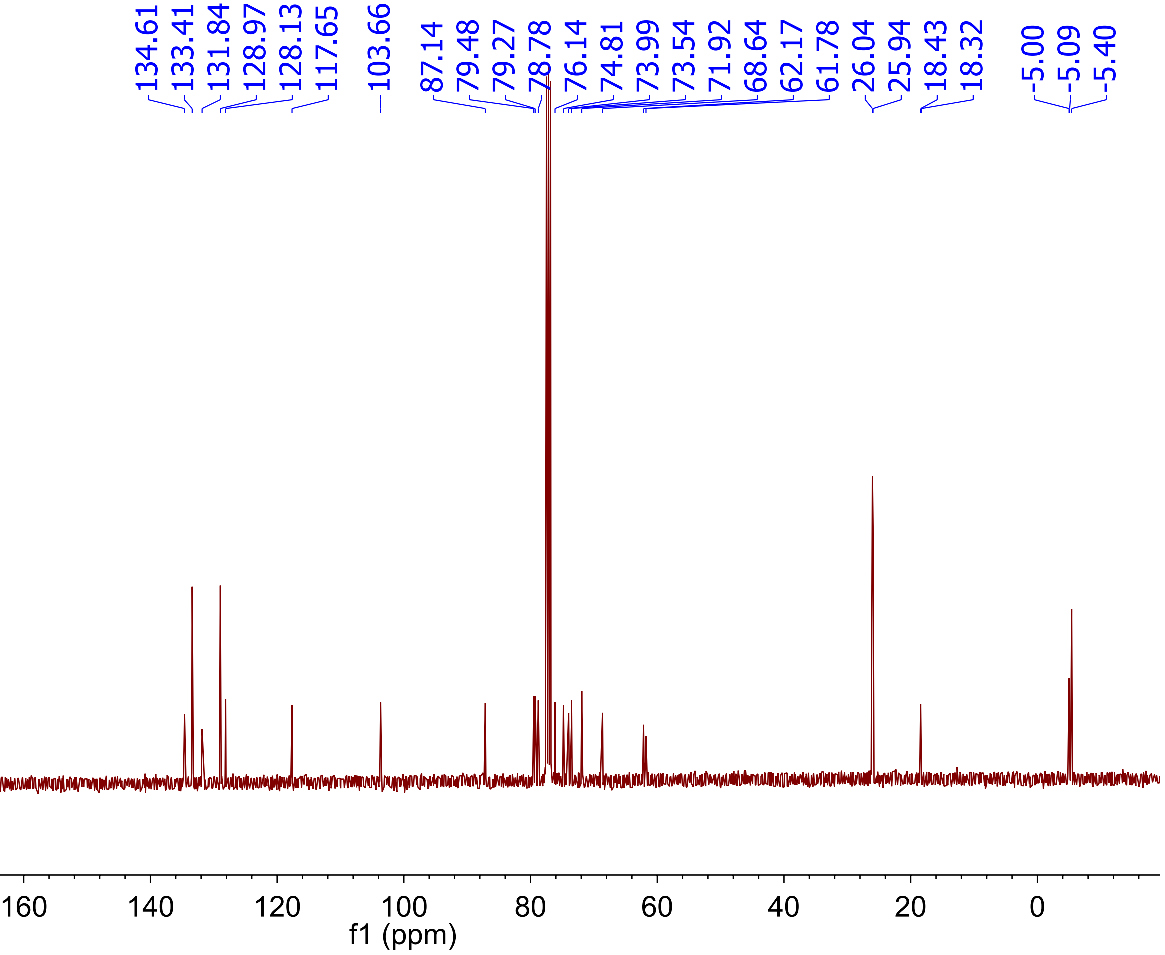

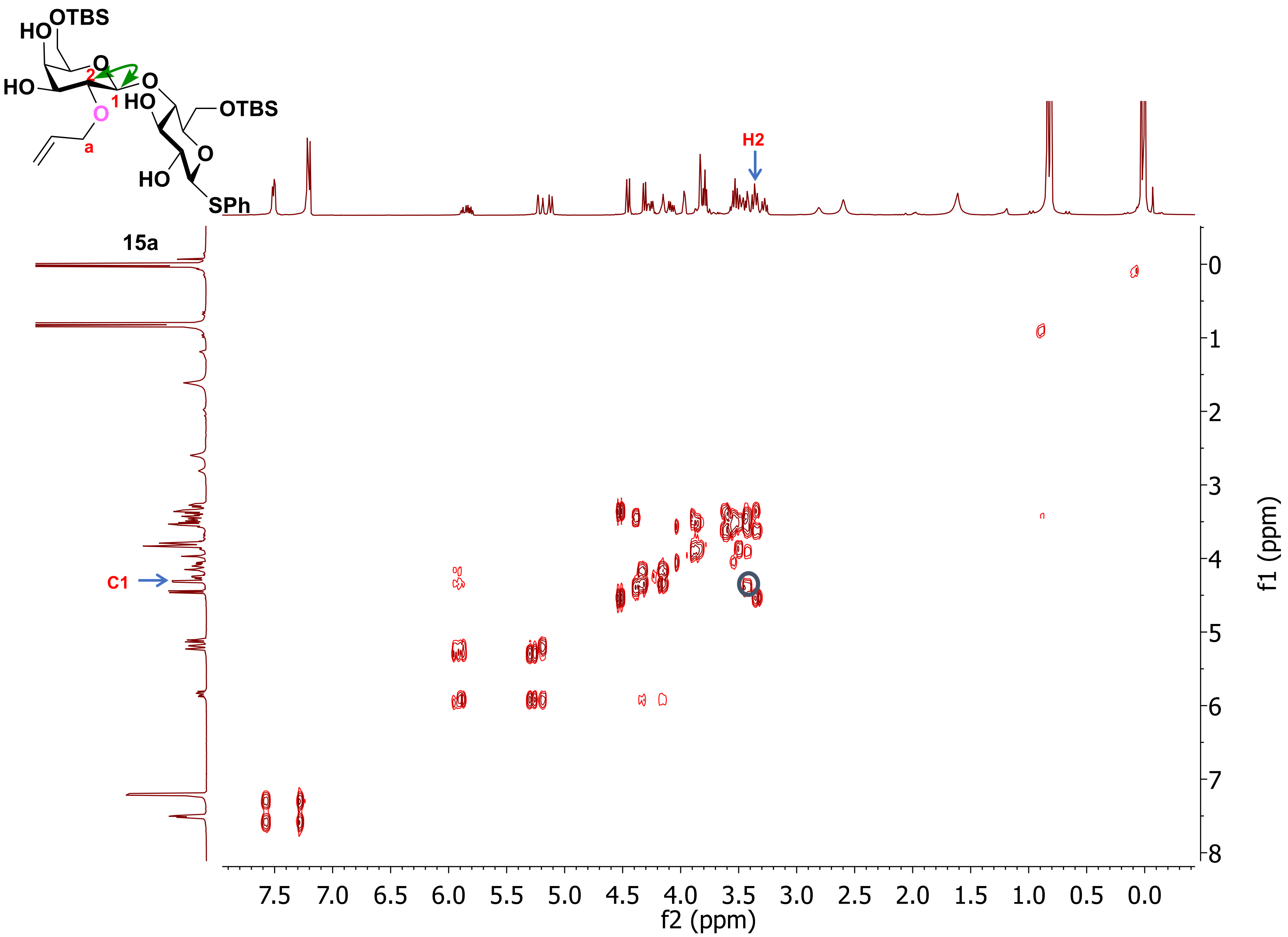
HO
OTBS
O
2
1
HO
O
OTBS
O
HO
a
O
HO
SPh
15a
H2
C1
7.5
7.0
6.5
6.0
5.5
5.0
4.5
4.0
3.5
3.0
2.5
2.0
1.5
1.0
0.5
0.0
f2 (ppm)
0
1
2
3
4
5
6
7
8
f1 (ppm)

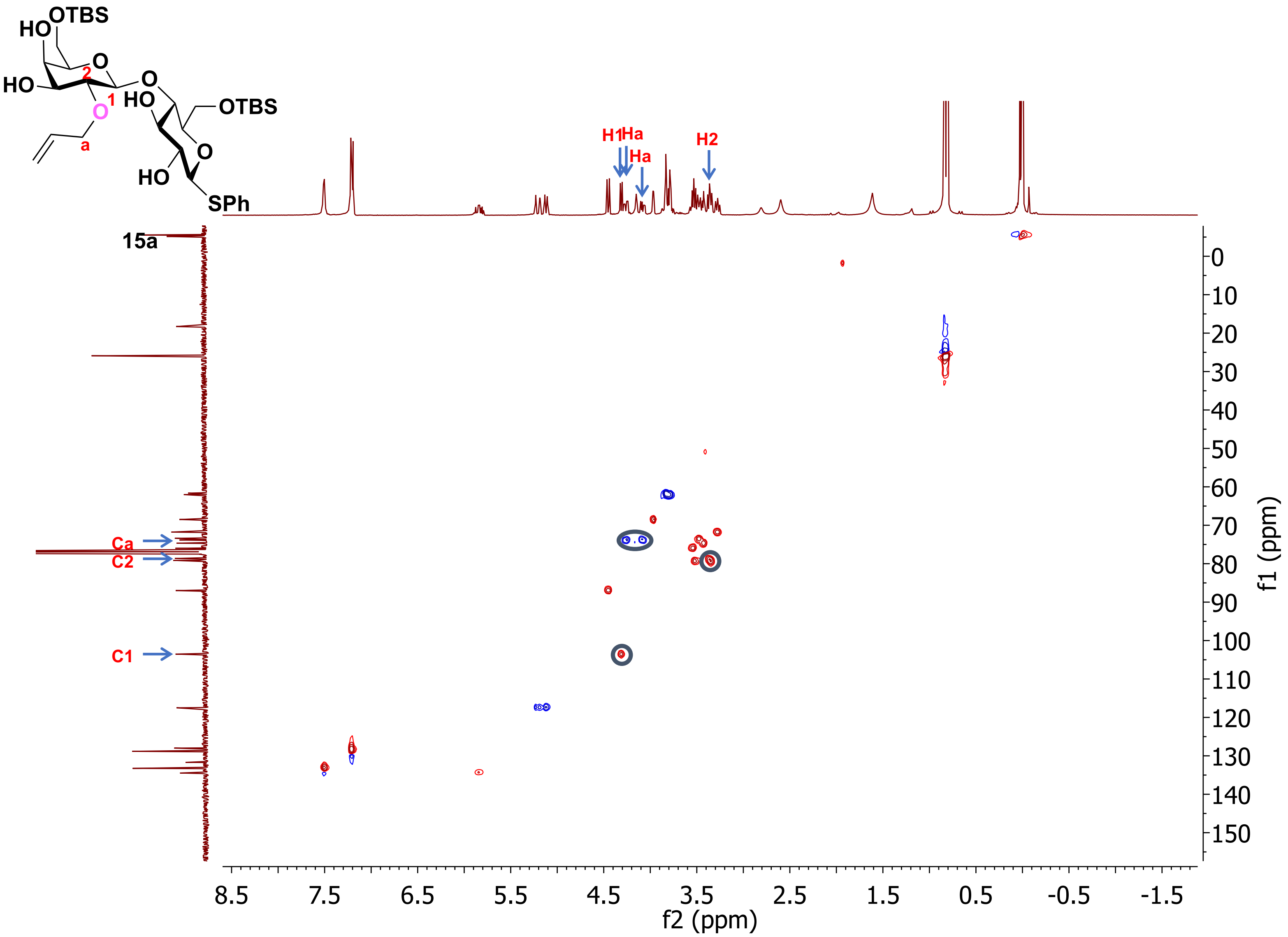

OTBS
HO
O
HO
2
1
O
HO
OTBS
a
O
HO
SPh
15a
H1
Ha
Ha
H2
Ca
C2
C1
8.5
7.5
6.5
5.5
4.5
3.5
2.5
1.5
0.5
-0.5
-1.5
f2 (ppm)
0
10
20
30
40
50
60
70
80
90
100
110
120
130
140
150
f1 (ppm)

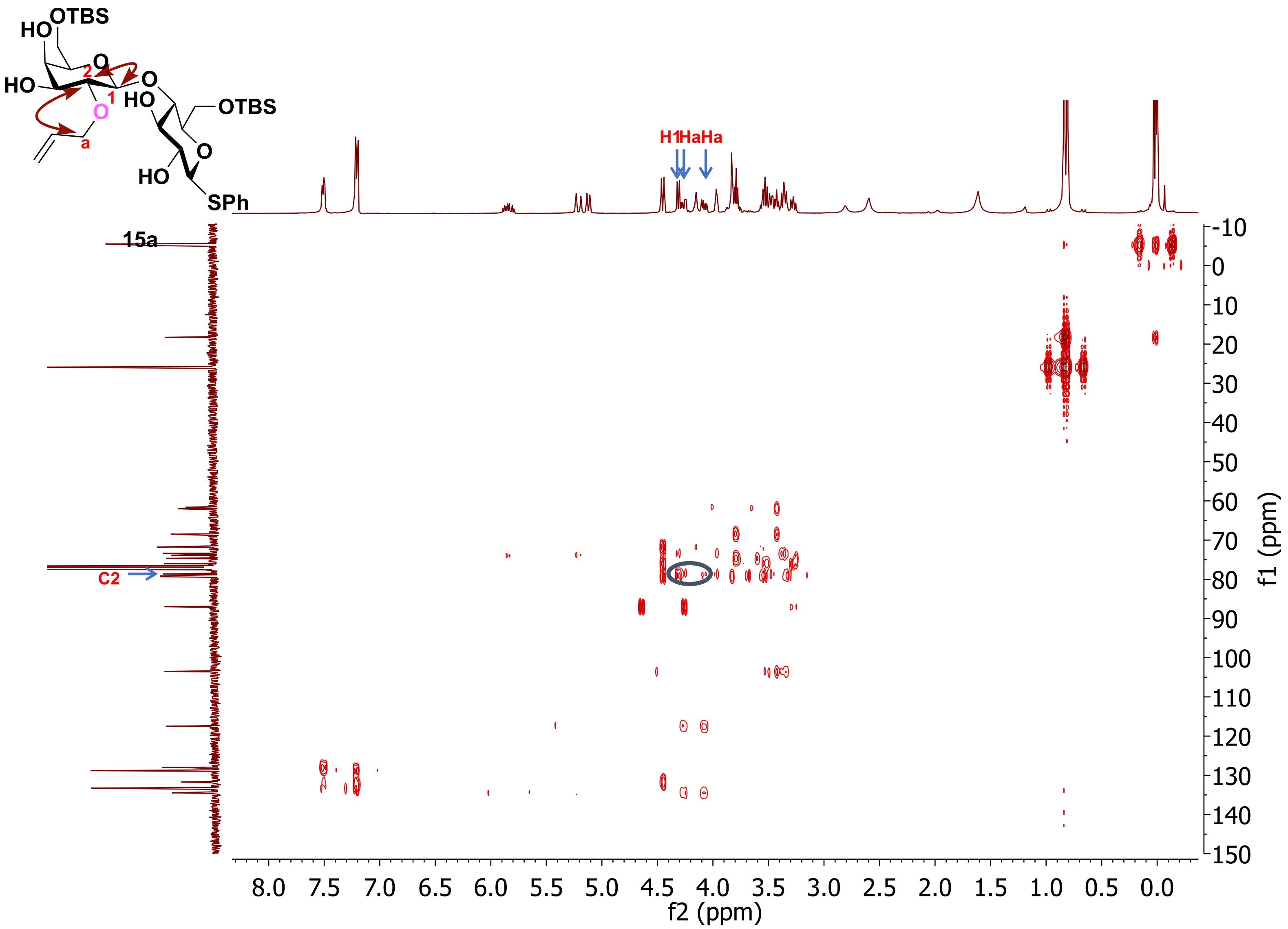

HO
OTBS
O
HO
2
1
O
HO
O
a
OTBS
O
HO
SPh
H1HaHa
15a
C2
f1 (ppm)
f2 (ppm)

CDCl3, 400.13 MHz

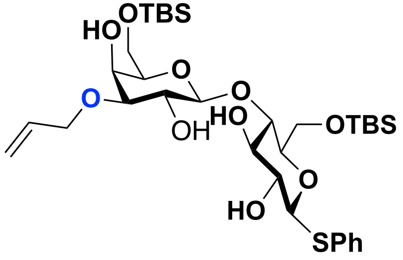

15b

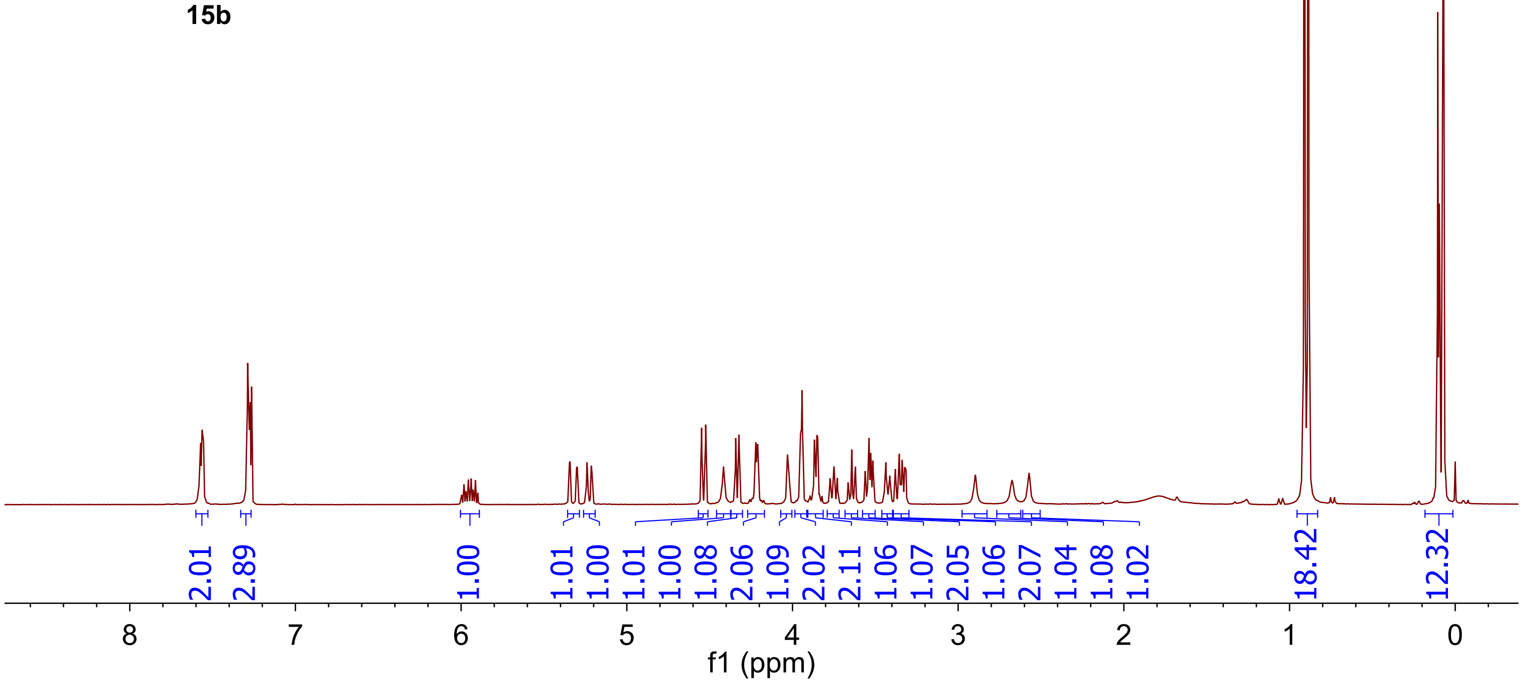

CDCl3, 100.62 MHz

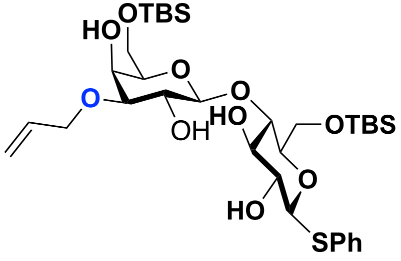

15b

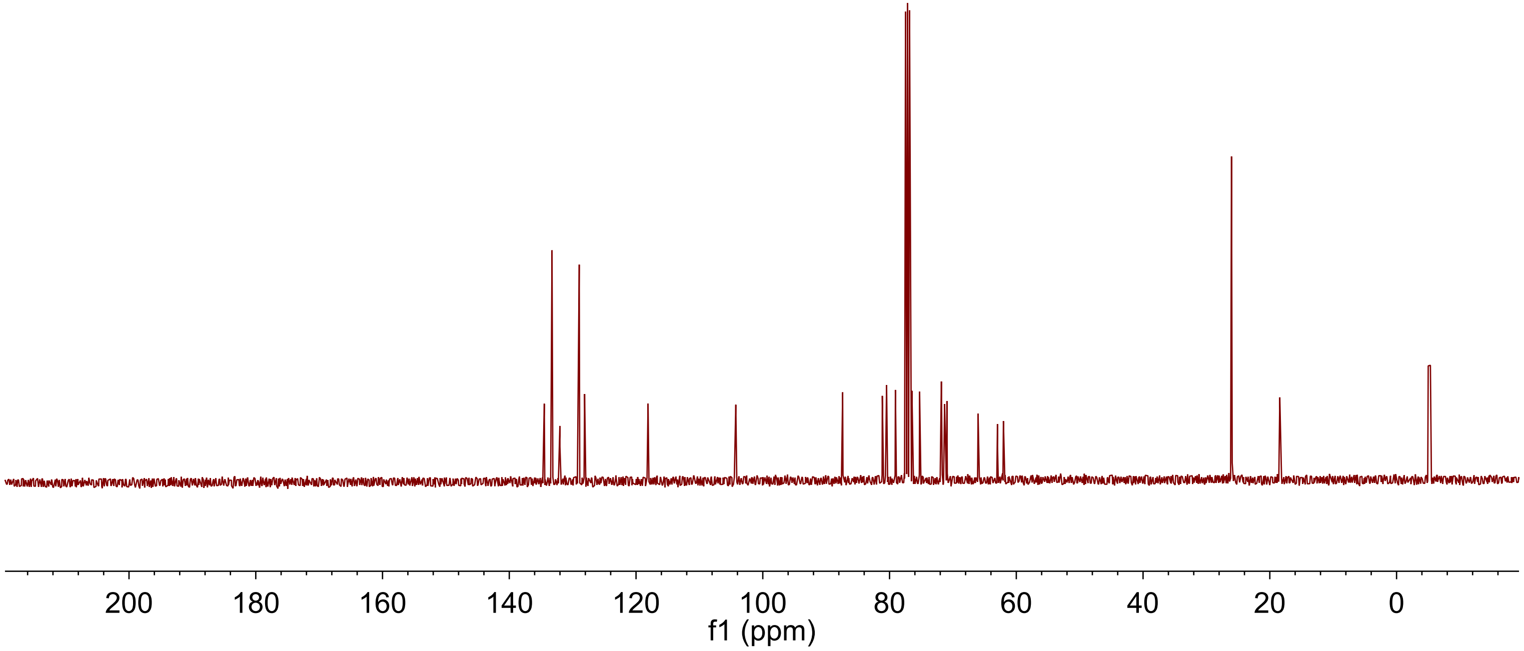

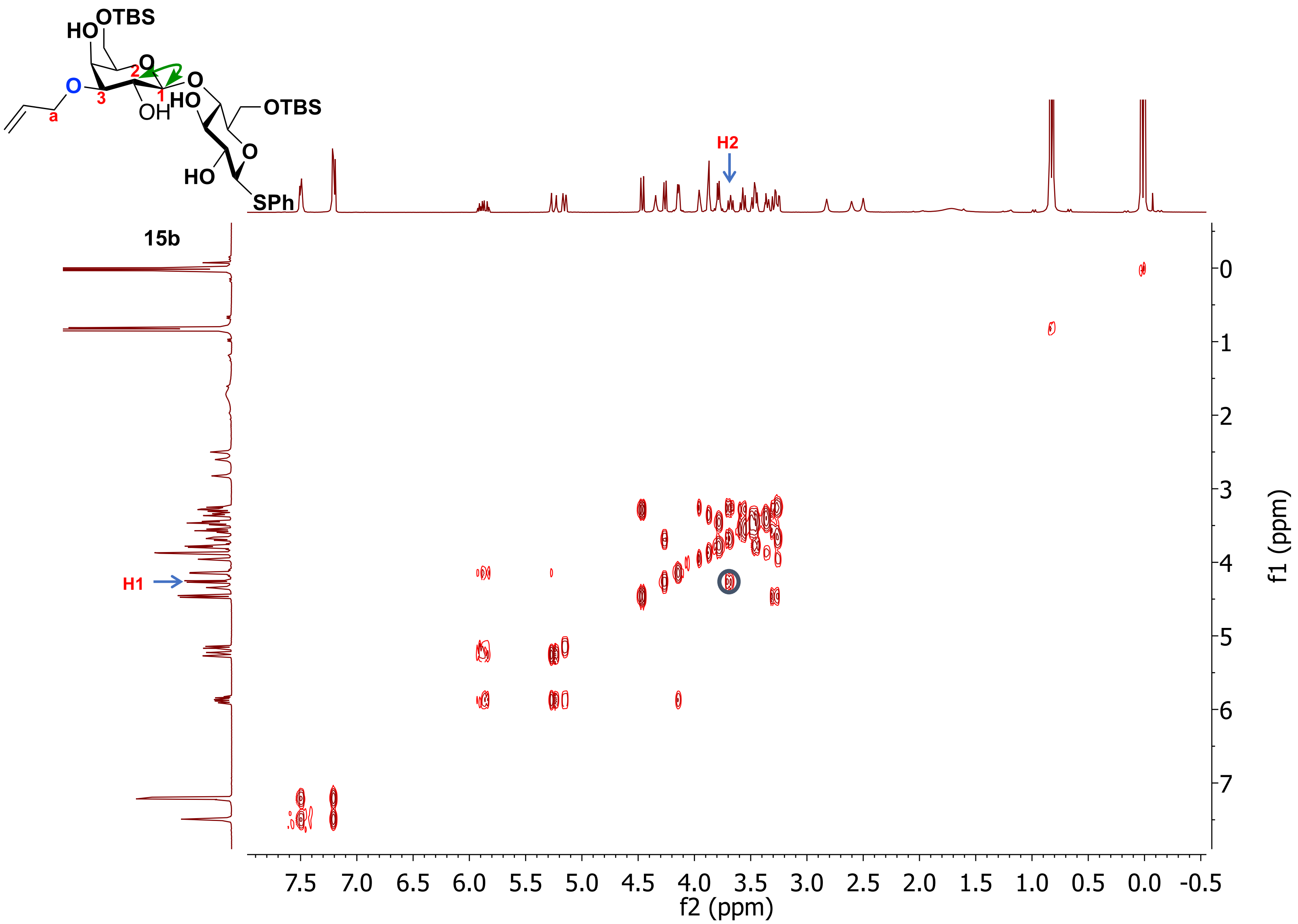

OTBS
HO
O
O
OTBS
HO
O
SPh
HO
OH
a
1
2
3
15b
H2
H1
f2 (ppm)
f1 (ppm)

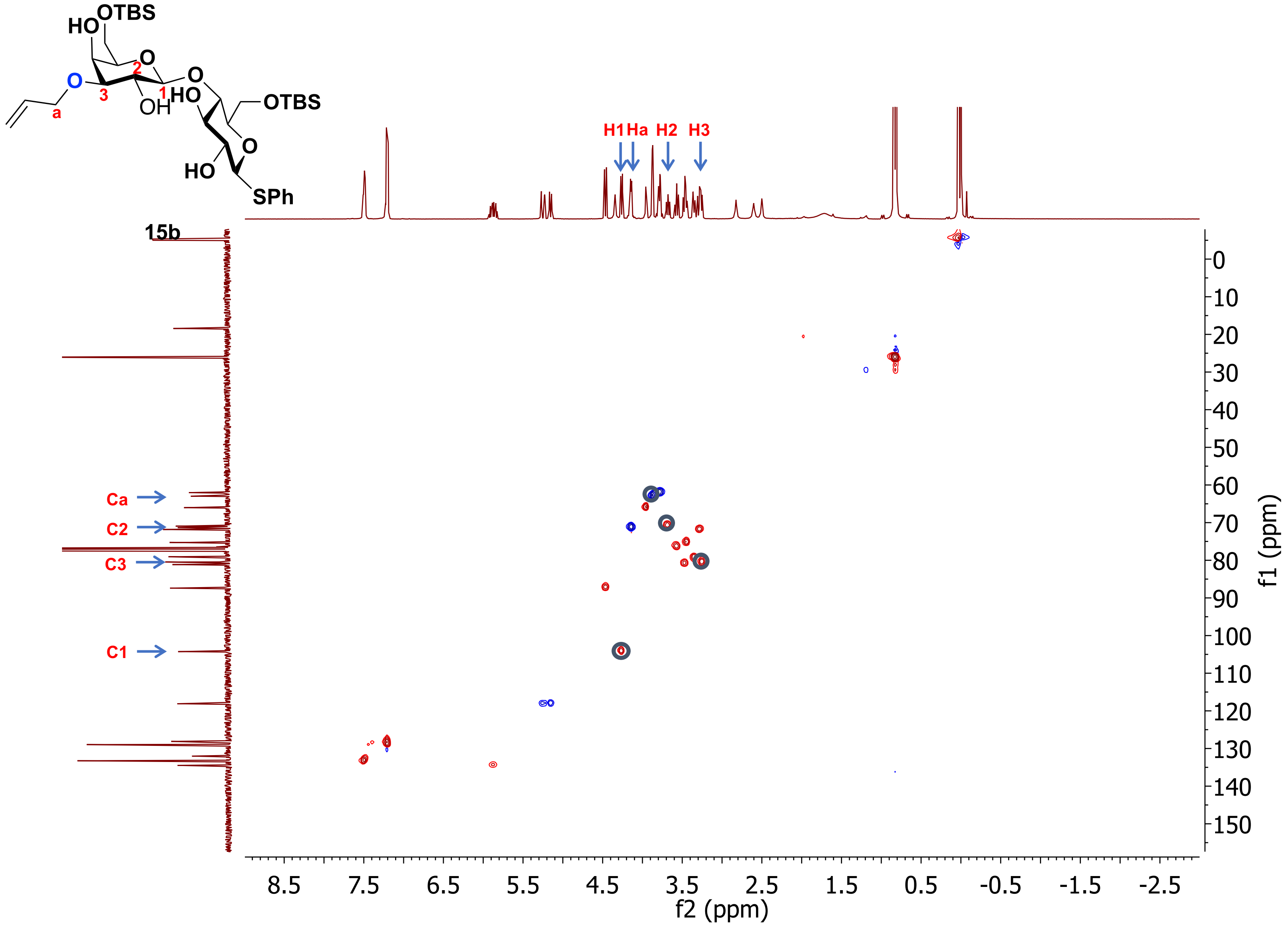
HO
OTBS
O
O
3
2
1
a
OH
HO
O
OTBS
O
HO
SPh
15b
H1
Ha
H2
H3
Ca
C2
C3
C1
f2 (ppm)
f1 (ppm)

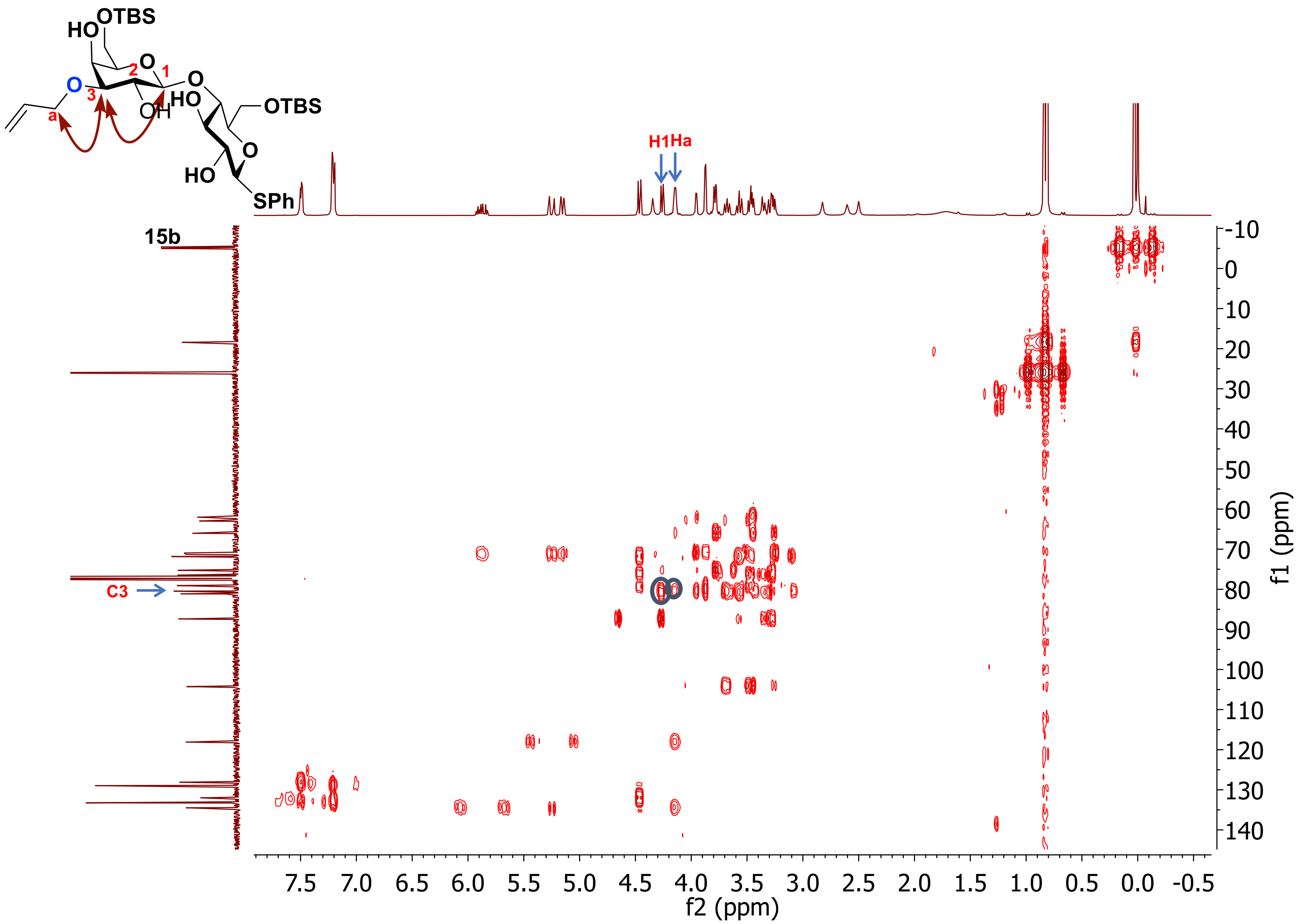

HO
OTBS
O
O
2
1
3
a
OH
HO
O
OTBS
O
HO
SPh
15b
H1
Ha
C3
f2 (ppm)
f1 (ppm)

CDCl3, 400.13 MHz

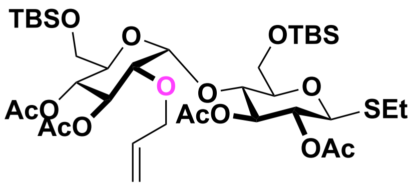

16a-acylated

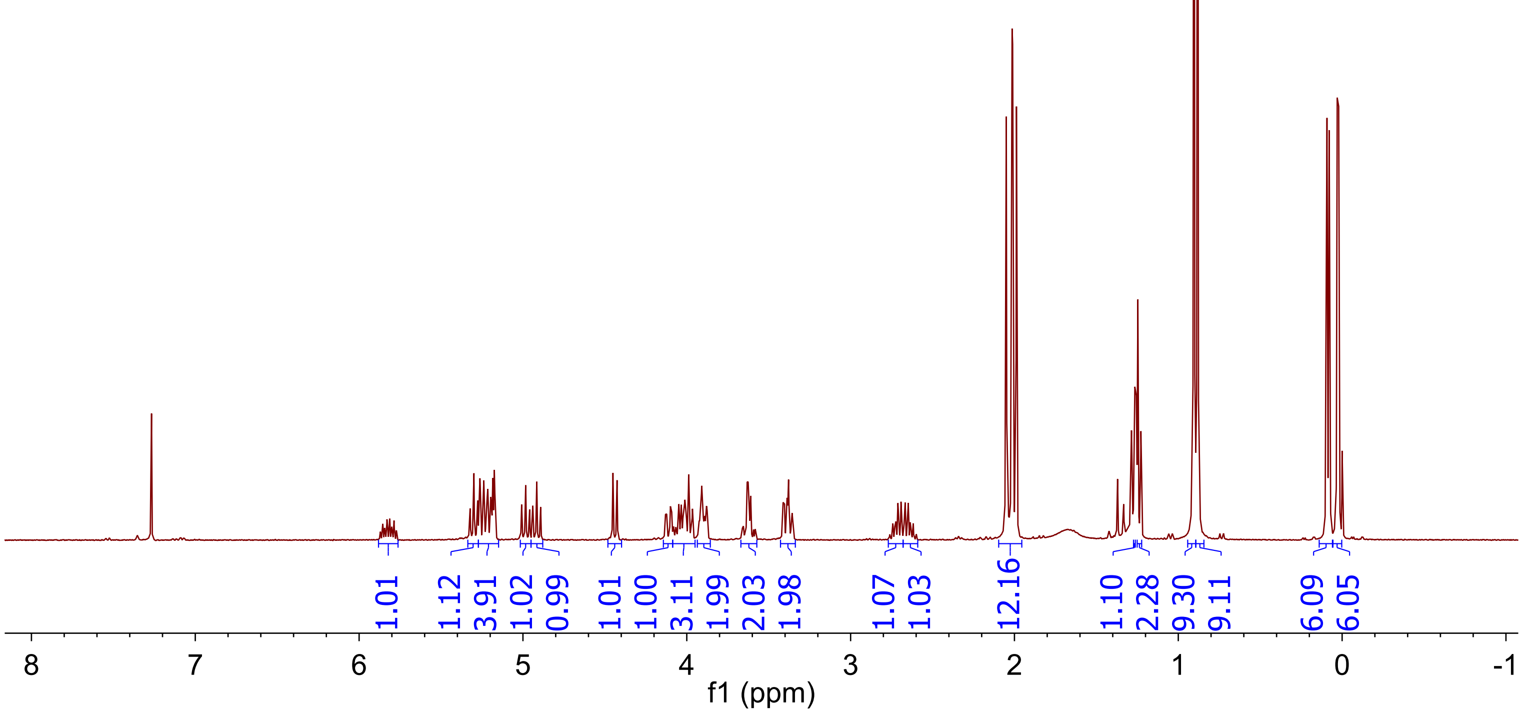

CDCl3, 100.62 MHz

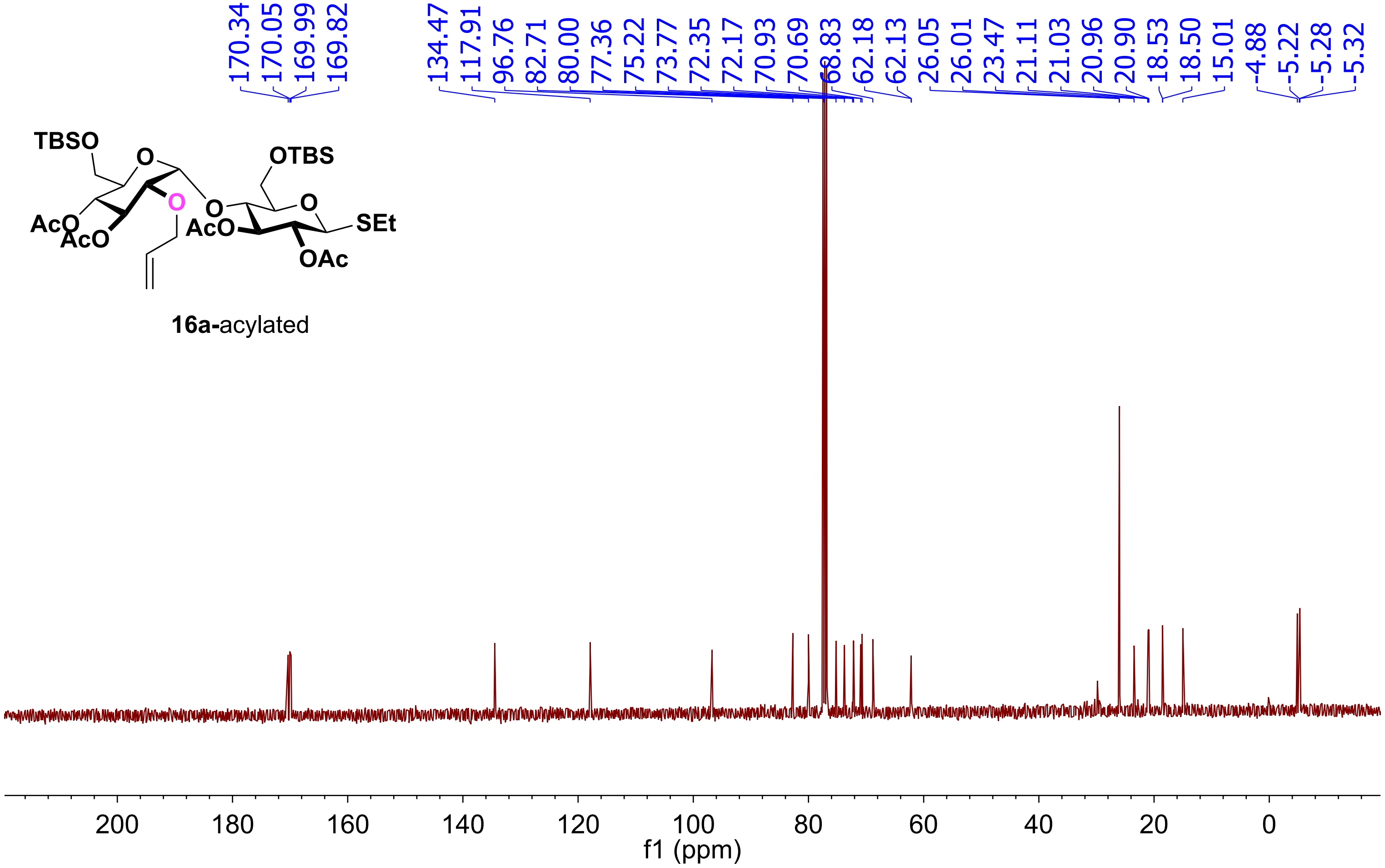

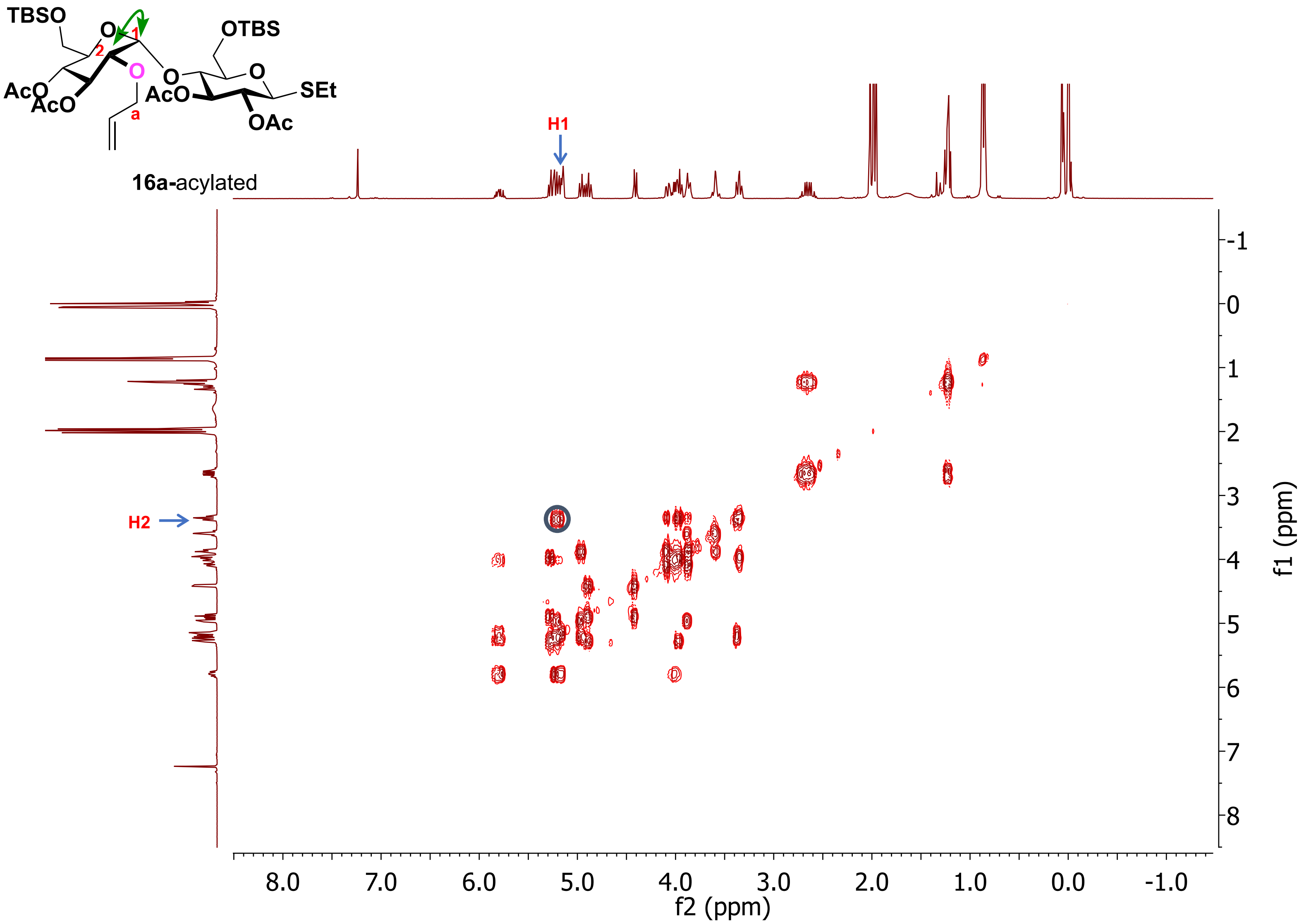
TBSO
OTBS
AcO
AcO
AcO
O
SEt
OAc
1
2
a
16a-acylated
H1
H2
8.0
7.0
6.0
5.0
4.0
3.0
2.0
1.0
0.0
-1.0
f2 (ppm)
-1
0
1
2
3
4
5
6
7
8
f1 (ppm)

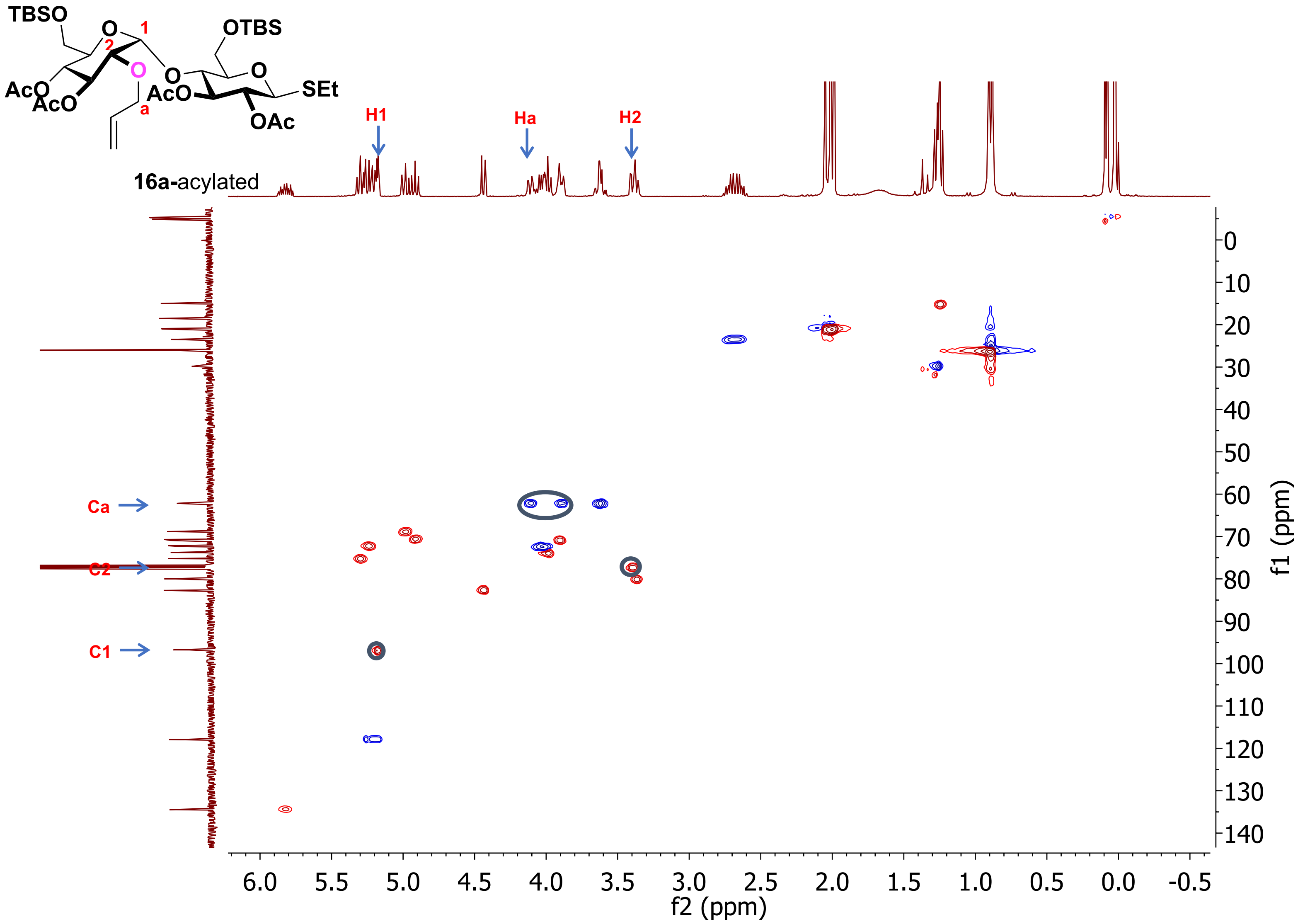
TBSO
OTBS
O
1
2
O
O
AcO
AcO
AcO
a
OAc
SEt
16a-acylated
H1
Ha
H2
Ca
C2
C1
f2 (ppm)
f1 (ppm)

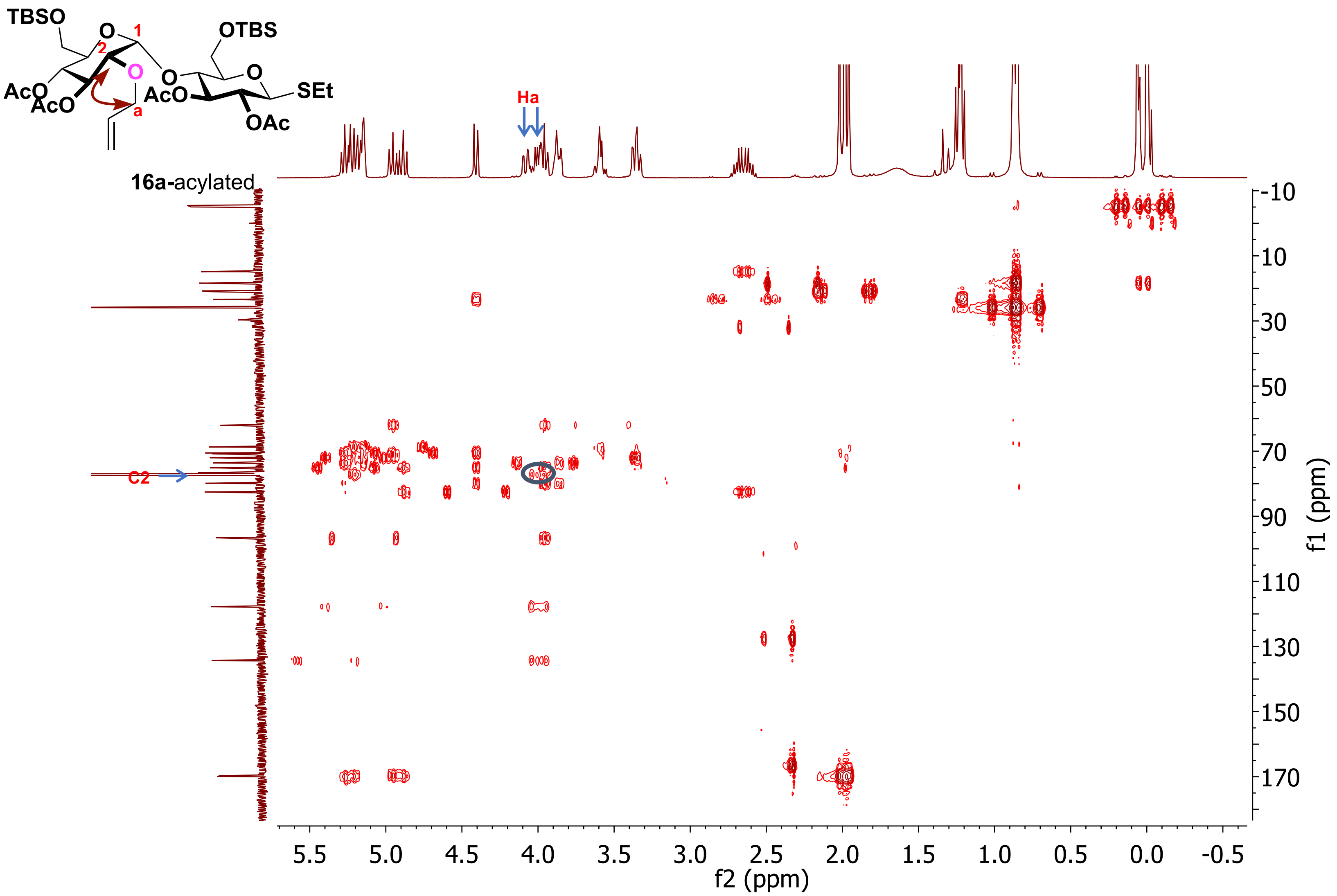
TBSO
OTBS
SEt
OAc
AcO
Ha
16a-acylated
C2
f2 (ppm)
f1 (ppm)

CDCl3, 400.13 MHz

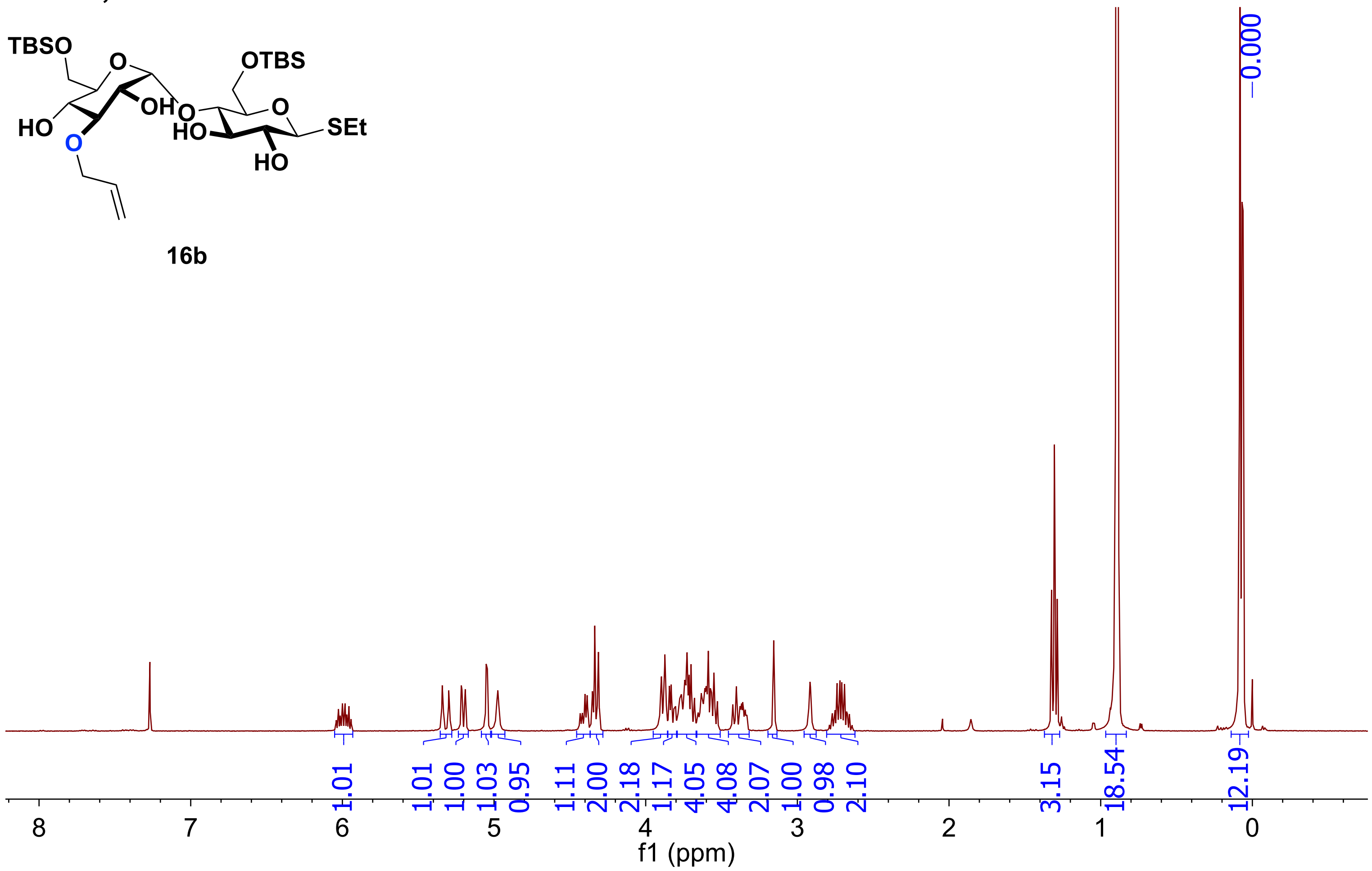

CDCl3, 100.62 MHz

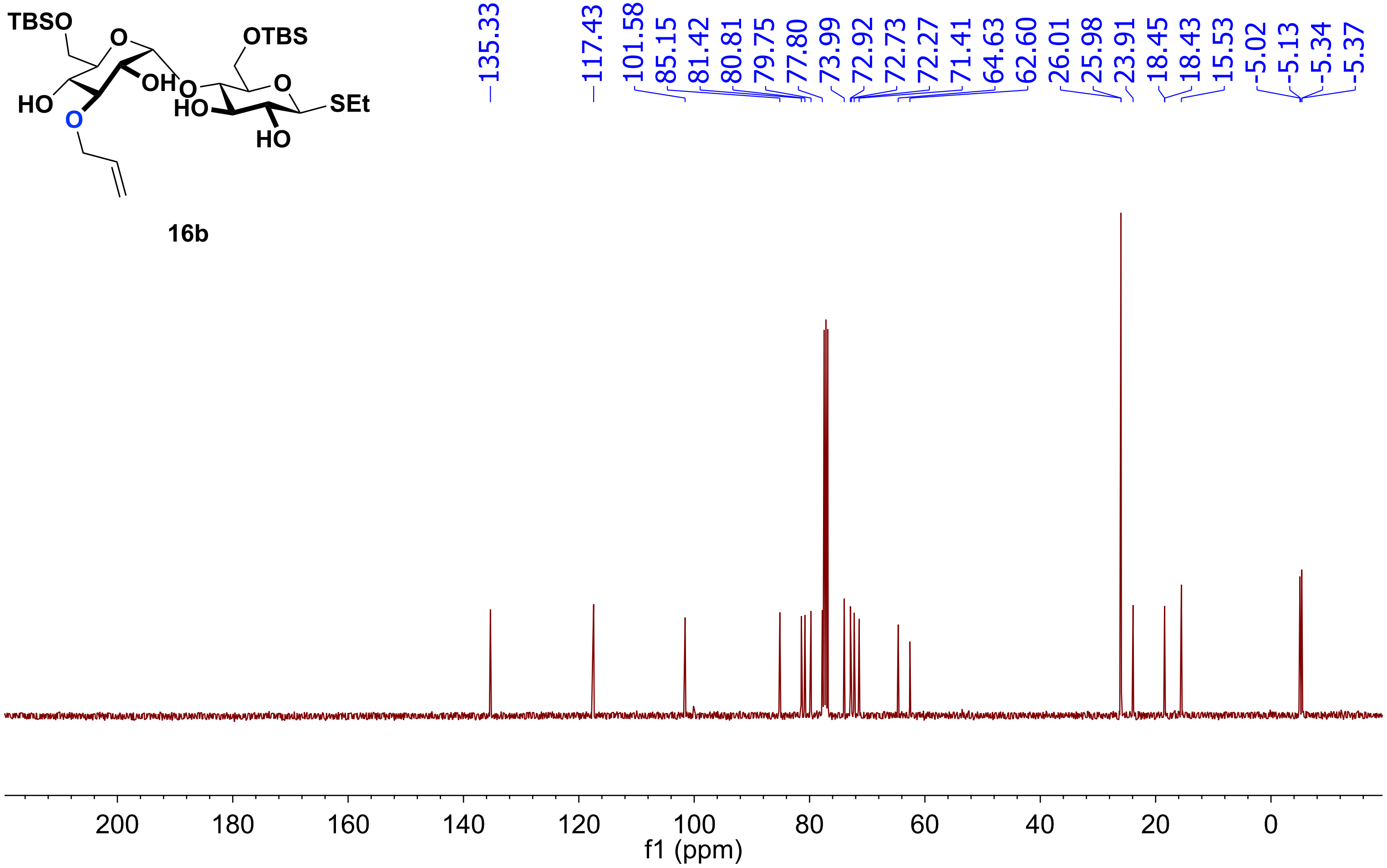

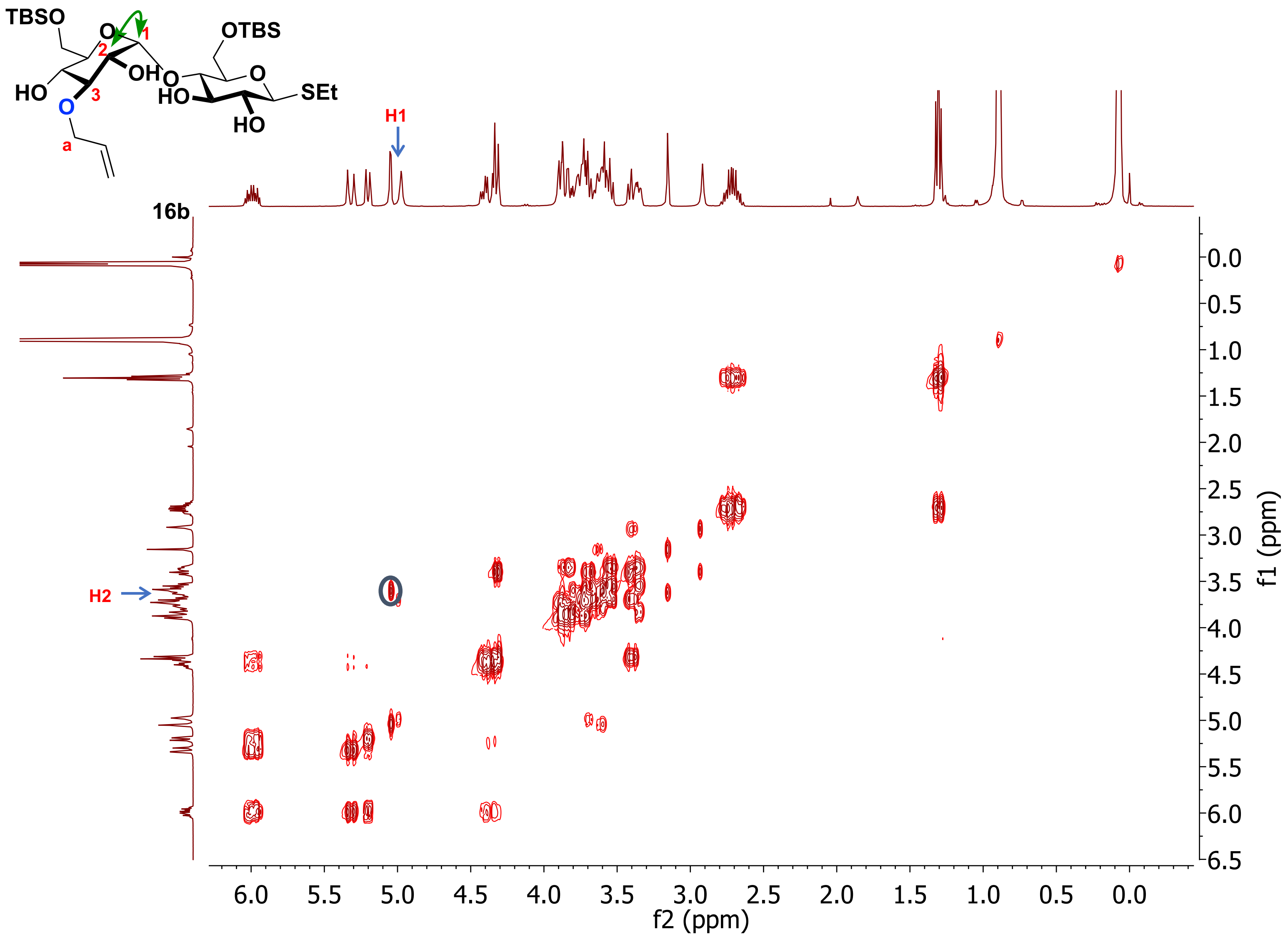

TBSO
O
2
1
OH
O
OTBS
O
SEt
HO
3
HO
O
HO
a
H1
16b
H2
6.0
5.5
5.0
4.5
4.0
3.5
3.0
2.5
2.0
1.5
1.0
0.5
0.0
f2 (ppm)
0.0
0.5
1.0
1.5
2.0
2.5
3.0
3.5
4.0
4.5
5.0
5.5
6.0
6.5
f1 (ppm)

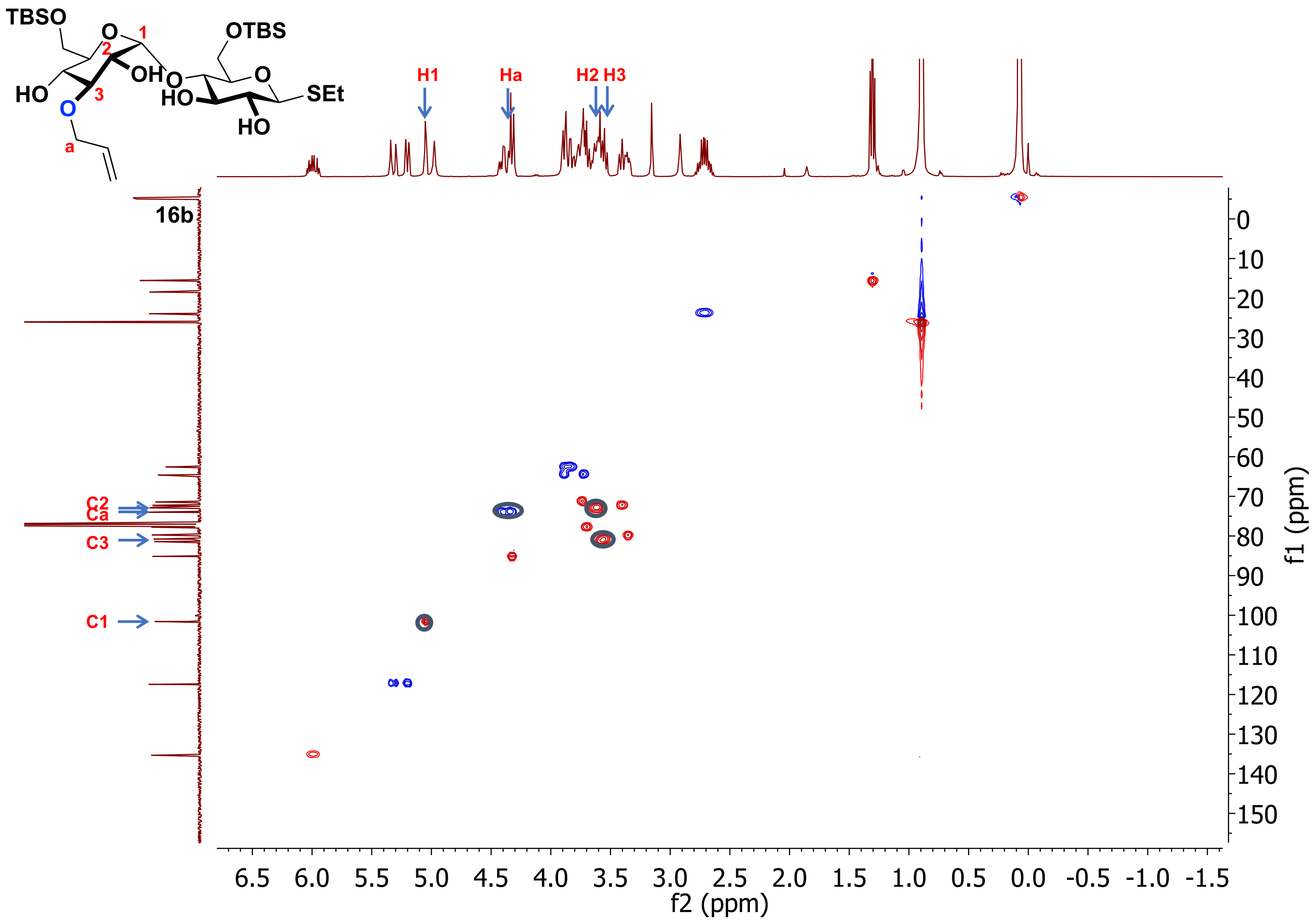

TBSO
O
1
2
OH
O
OTBS
O
SEt
HO
3
O
a
HO
HO
H1
Ha
H2
H3
16b
C2
Ca
C3
C1
6.5
6.0
5.5
5.0
4.5
4.0
3.5
3.0
2.5
2.0
1.5
1.0
0.5
0.0
-0.5
-1.0
-1.5
f2 (ppm)
0
10
20
30
40
50
60
70
80
90
100
110
120
130
140
150
f1 (ppm)

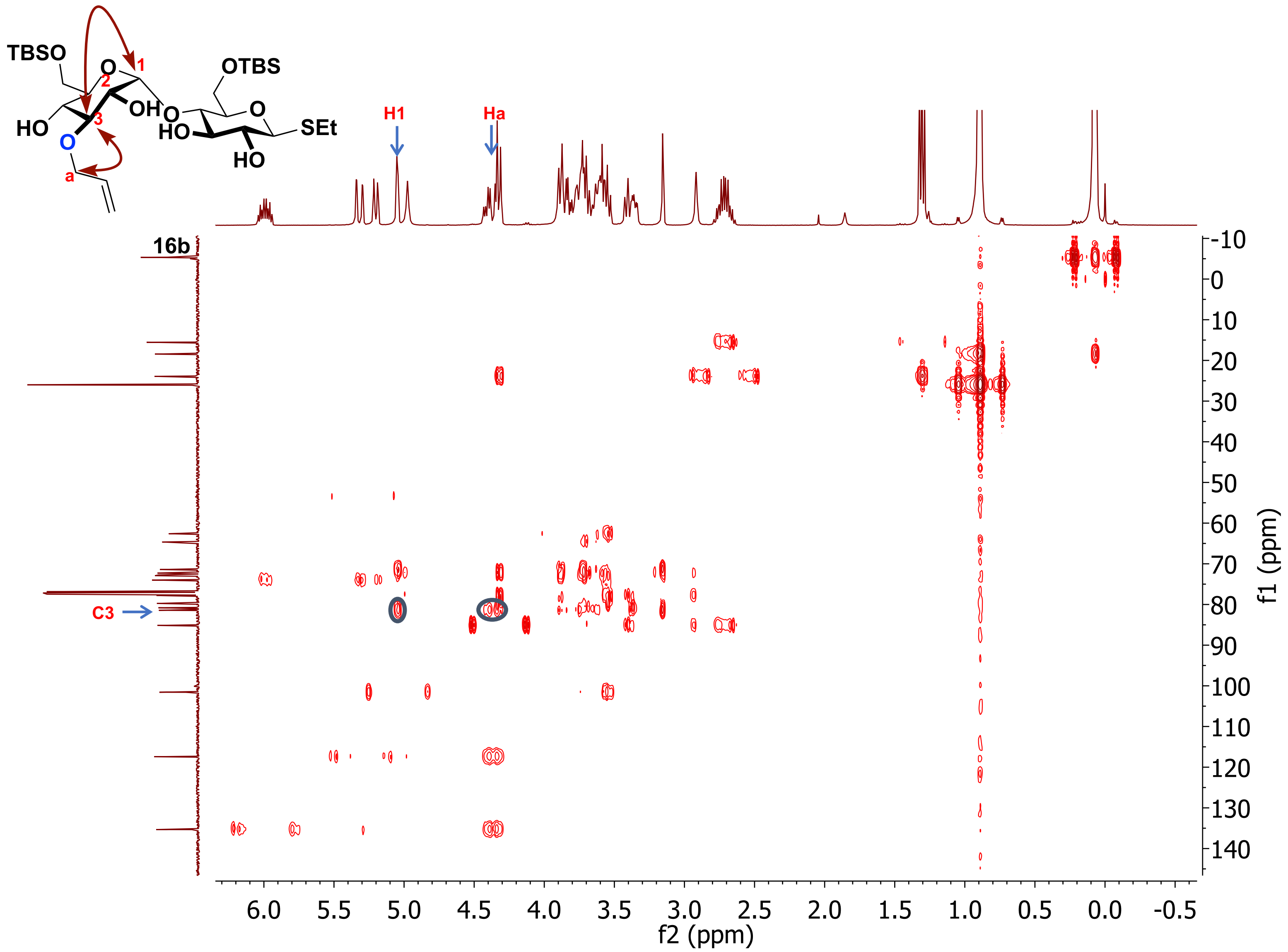
TBSO
O
1
2
OH
O
OTBS
O
HO
3
HO
SEt
O
a
HO
H1
Ha
16b
C3
f2 (ppm)
f1 (ppm)

CDCl3, 400.13 MHz

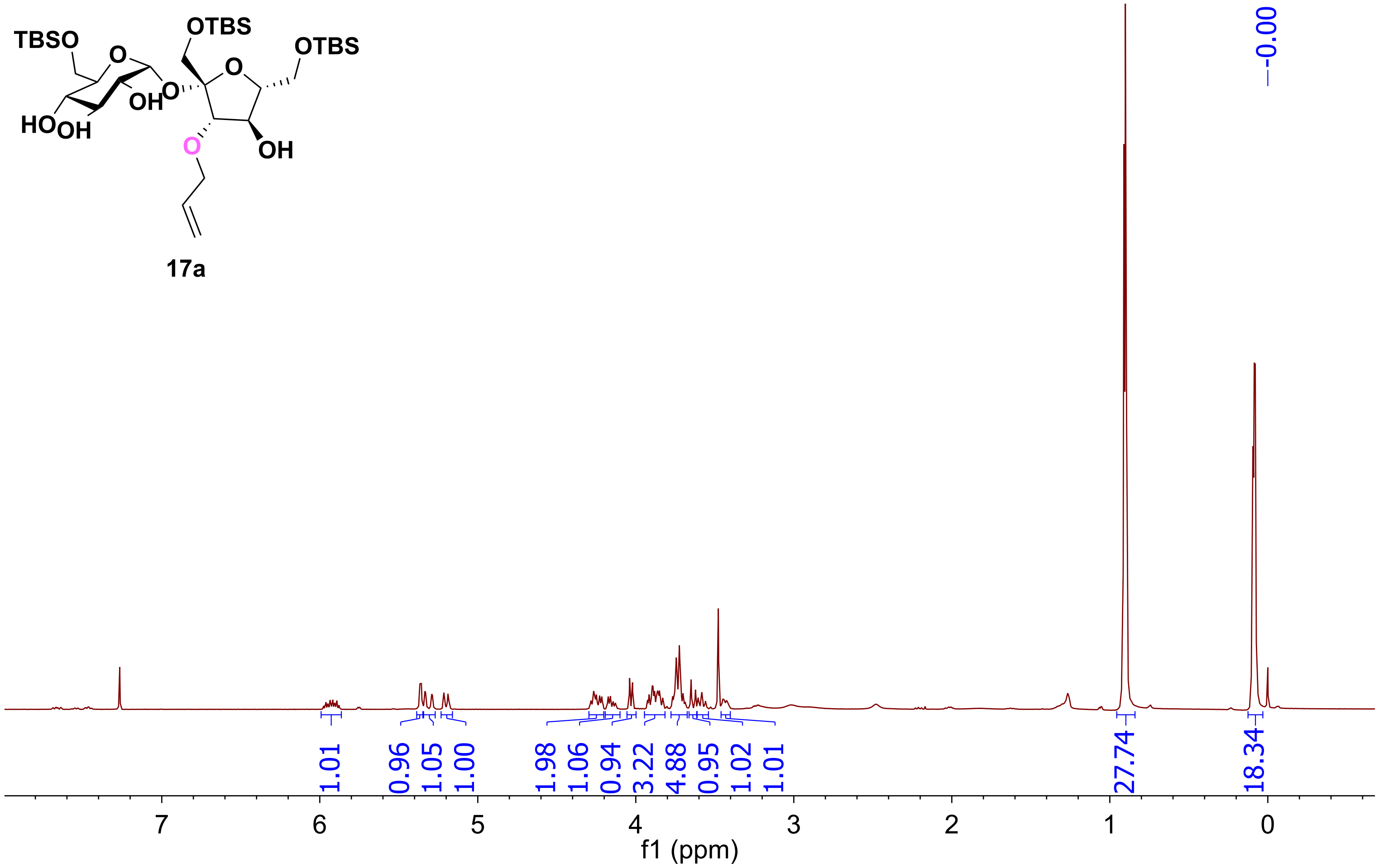

CDCl3, 100.62 MHz

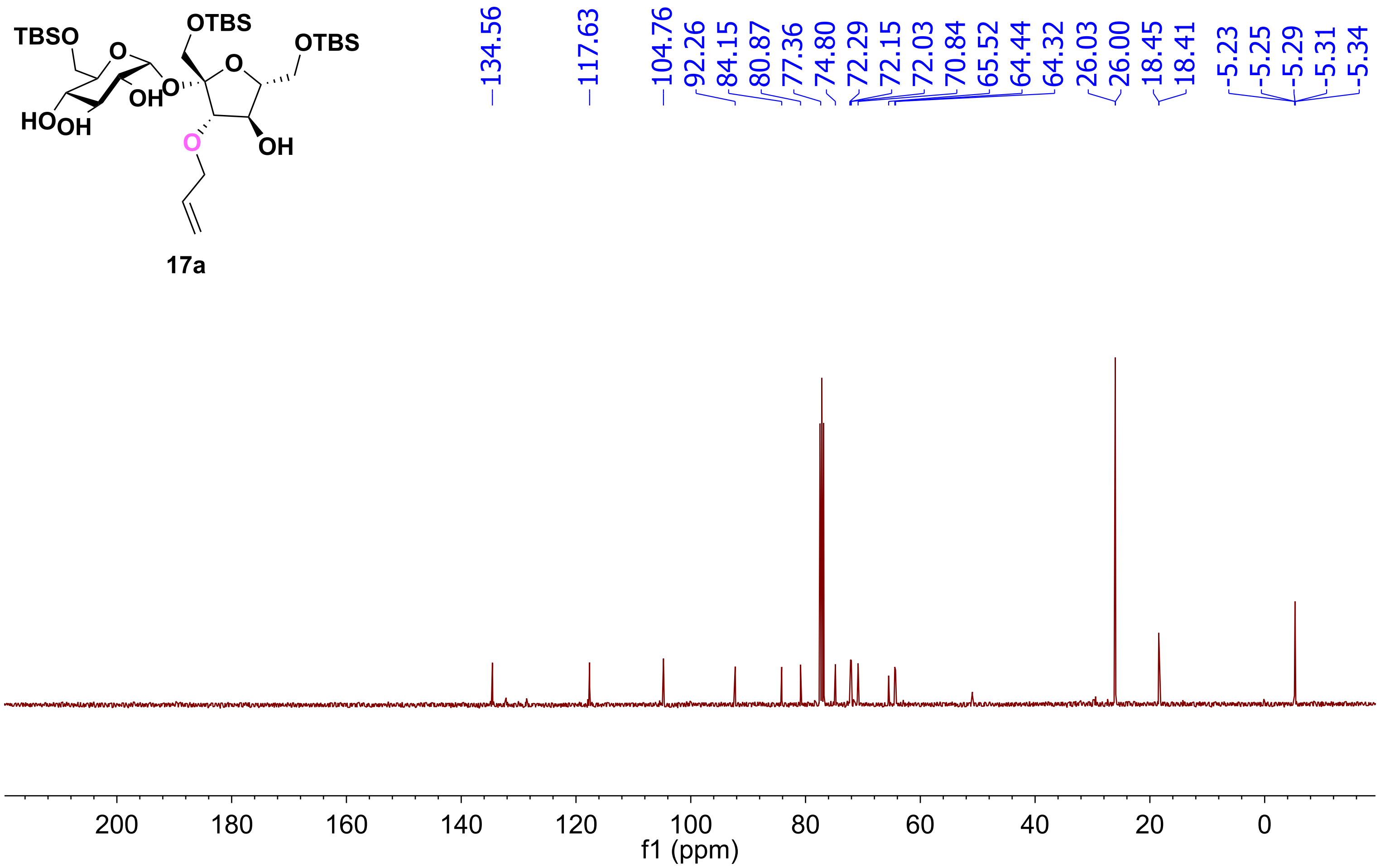

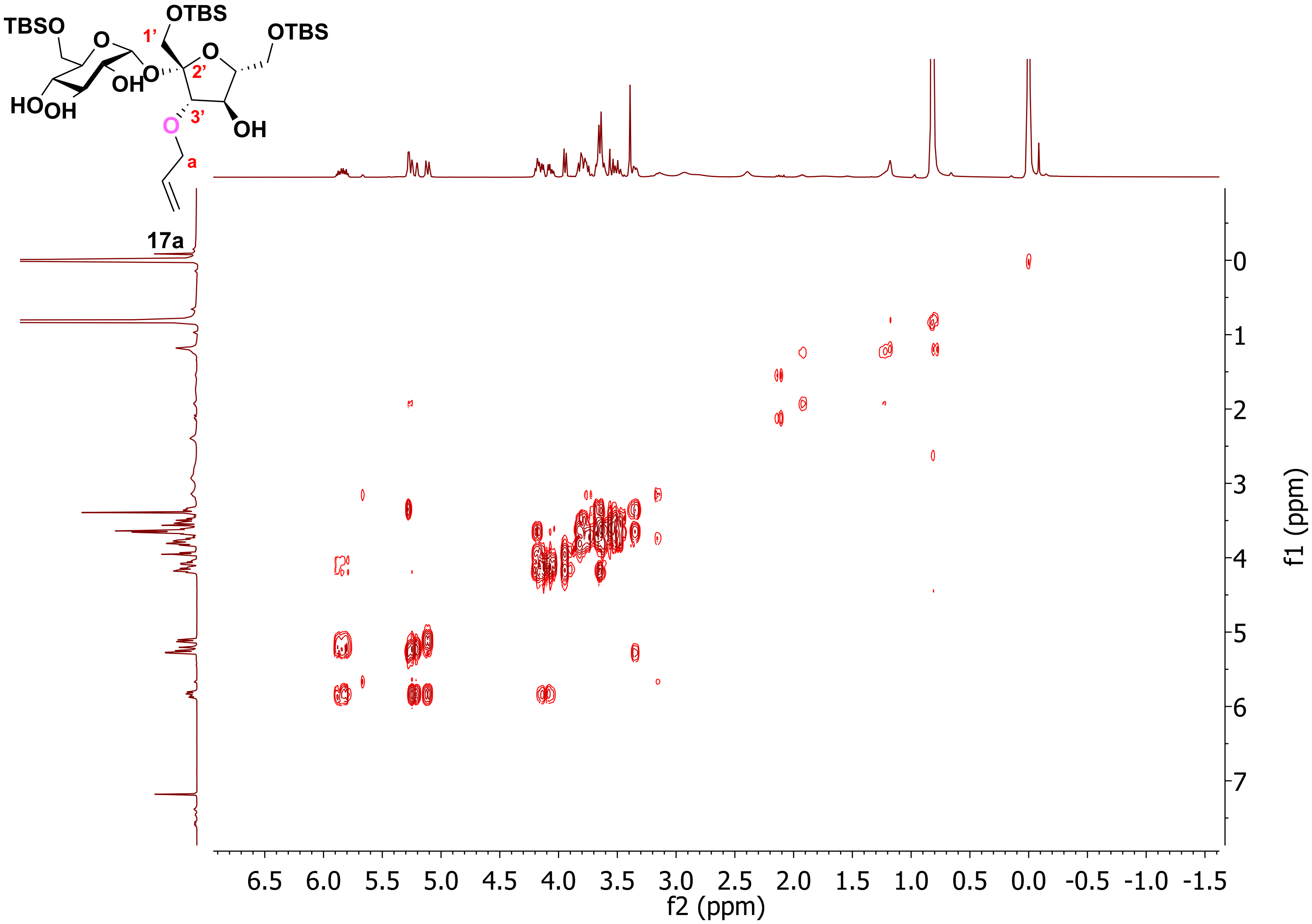
TBSO
OTBS
OTBS
1'
2'
3'
O
OH
HO
OH
OH
OH
a
17a
6.5 6.0 5.5 5.0 4.5 4.0 3.5 3.0 2.5 2.0 1.5 1.0 0.5 0.0 -0.5 -1.0 -1.5
f2 (ppm)
0 1 2 3 4 5 6 7
f1 (ppm)

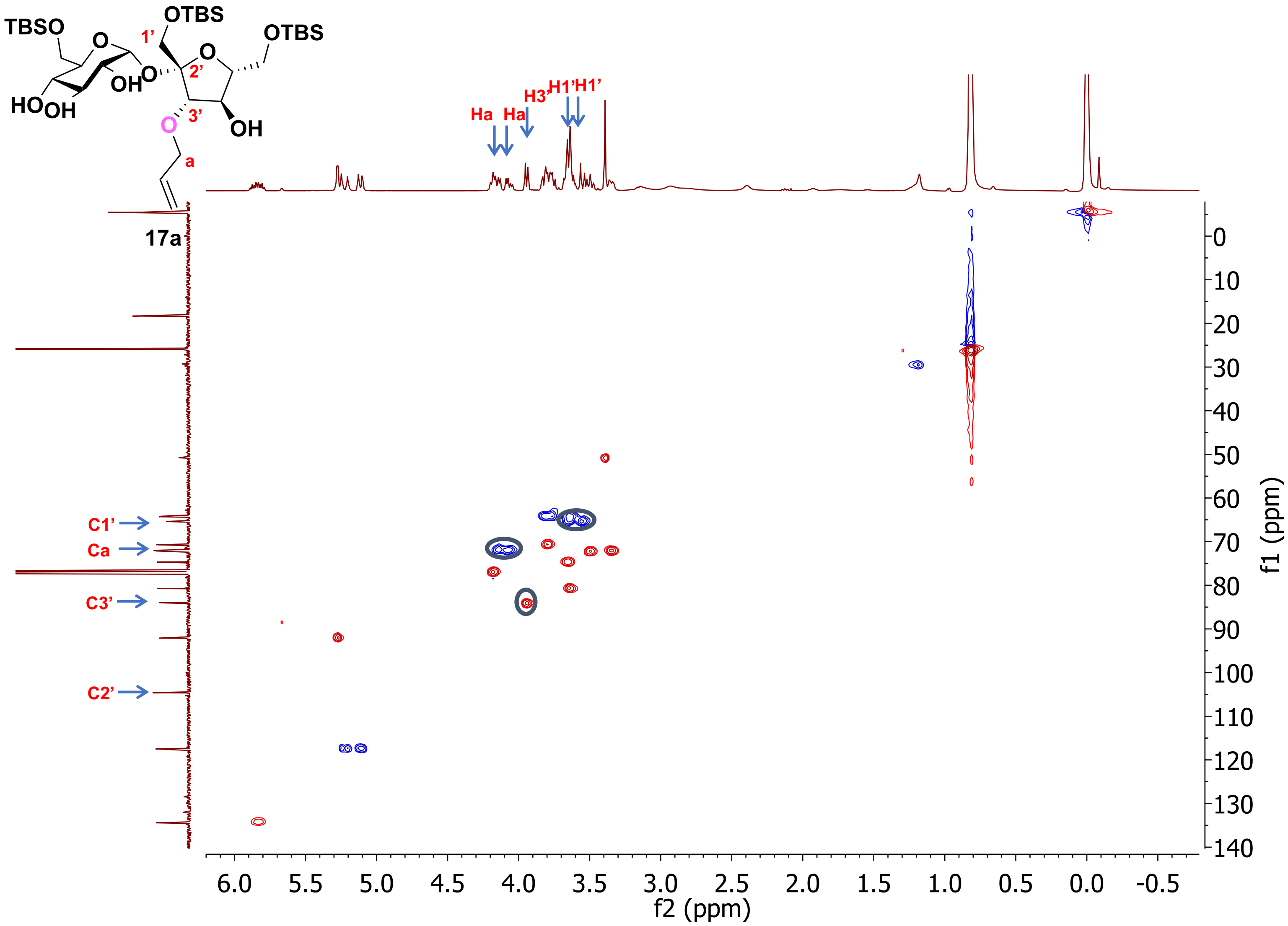

TBSO
OTBS
OTBS
1'
2'
3'
OH
HO
OH
OH
a
17a
Ha
Ha
H3'
H1'
H1'
C1'
Ca
C3'
C2'
f2 (ppm)
f1 (ppm)

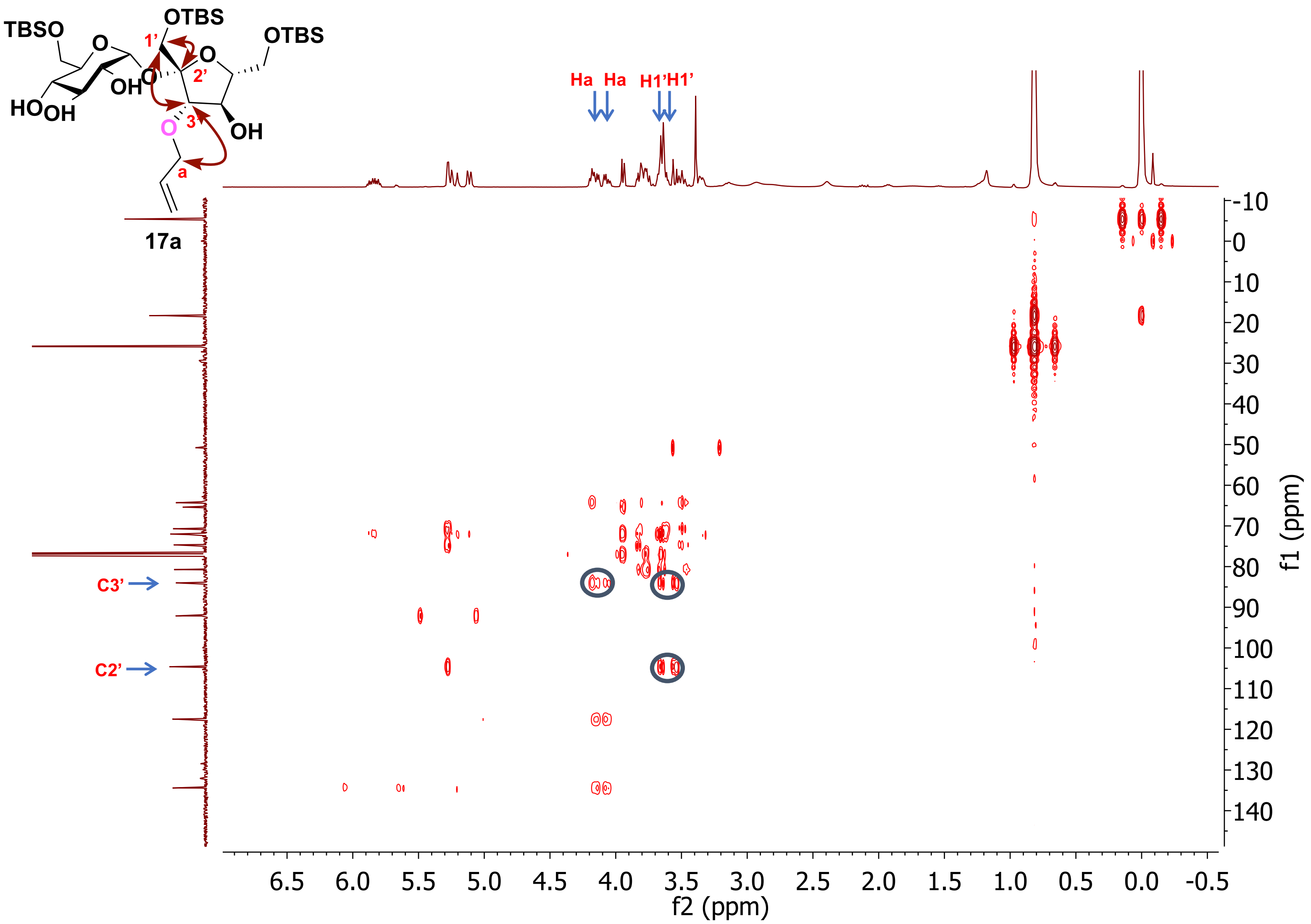

TBSO
OTBS
OTBS
1'
2'
3'
OH
HO
OH
OH
O
a
17a
Ha Ha
H1' H1'
C3'
C2'
f2 (ppm)
f1 (ppm)

CDCl3, 400.13 MHz

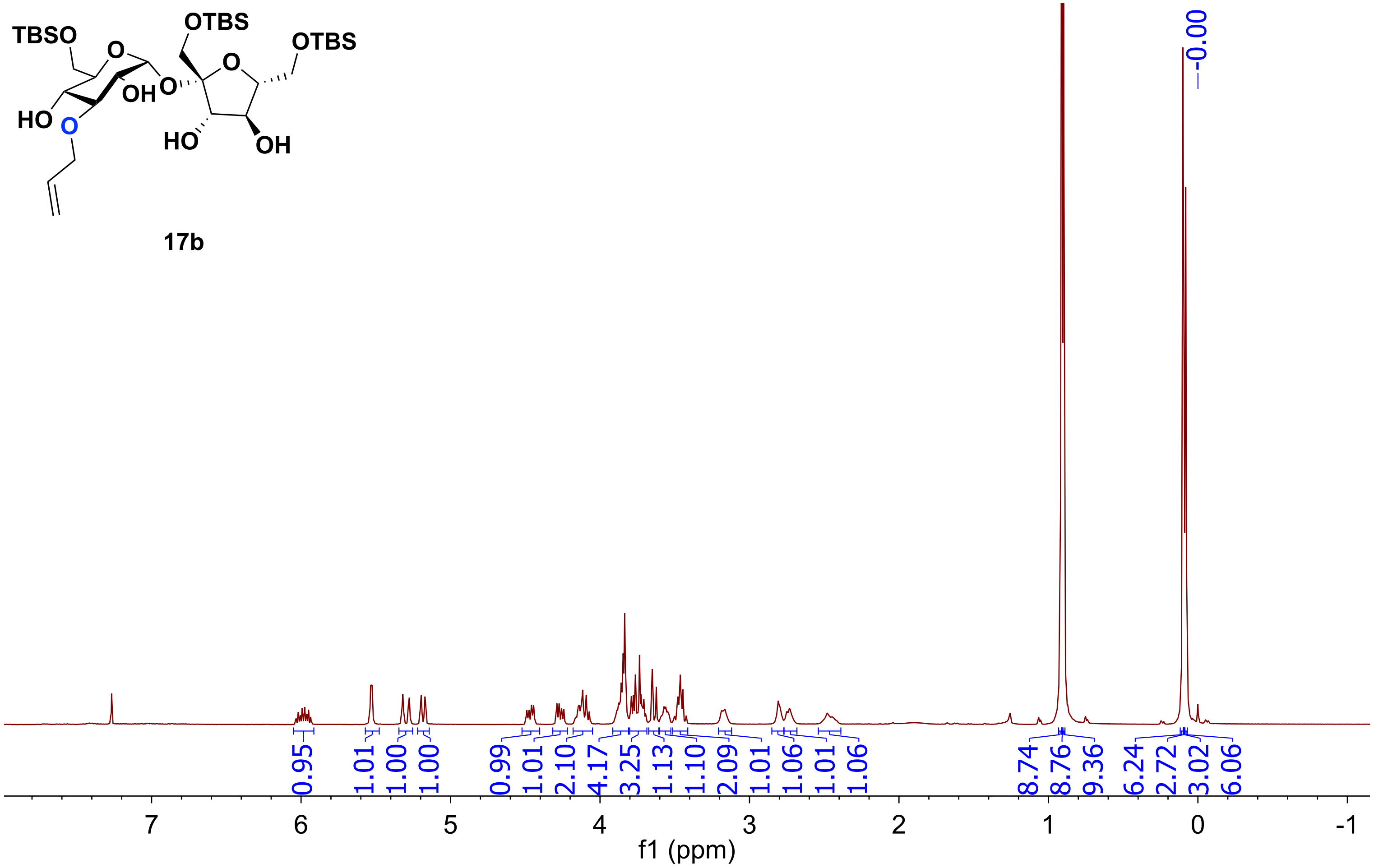

CDCl3, 100.62 MHz

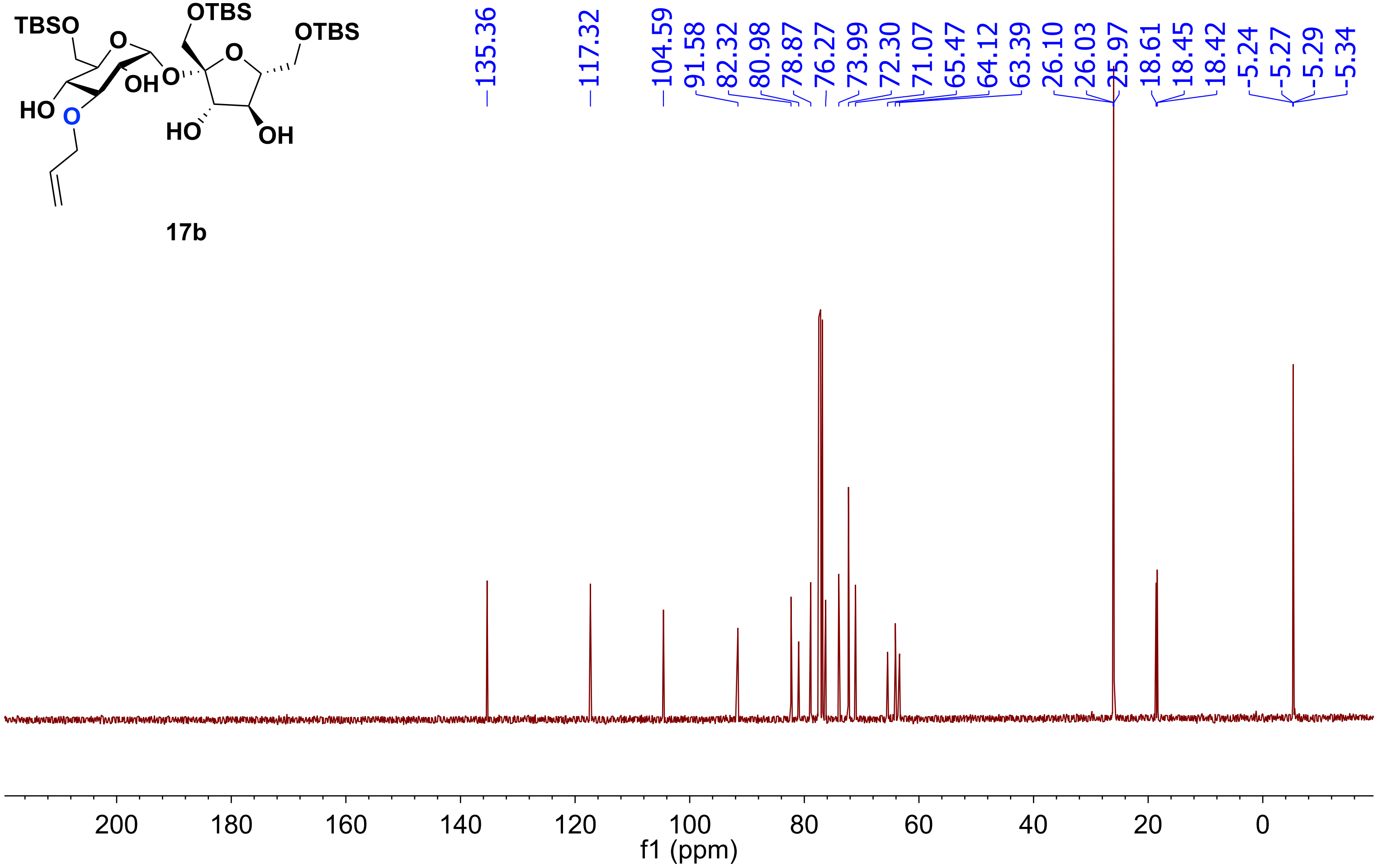

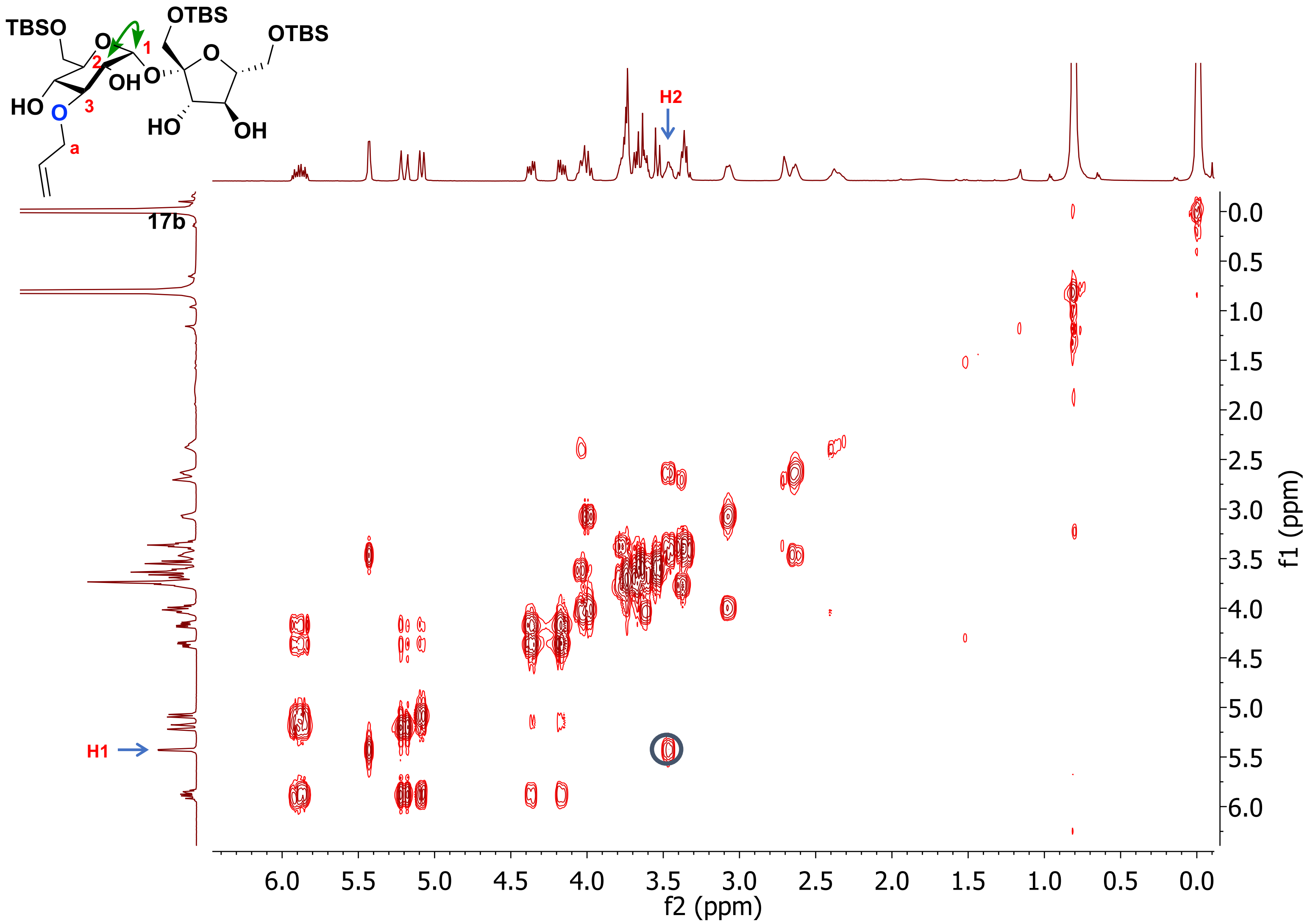
TBSO
OTBS
OTBS
OH
HO
HO
OH
a
1
2
3
17b
H2
H1
f2 (ppm)
f1 (ppm)

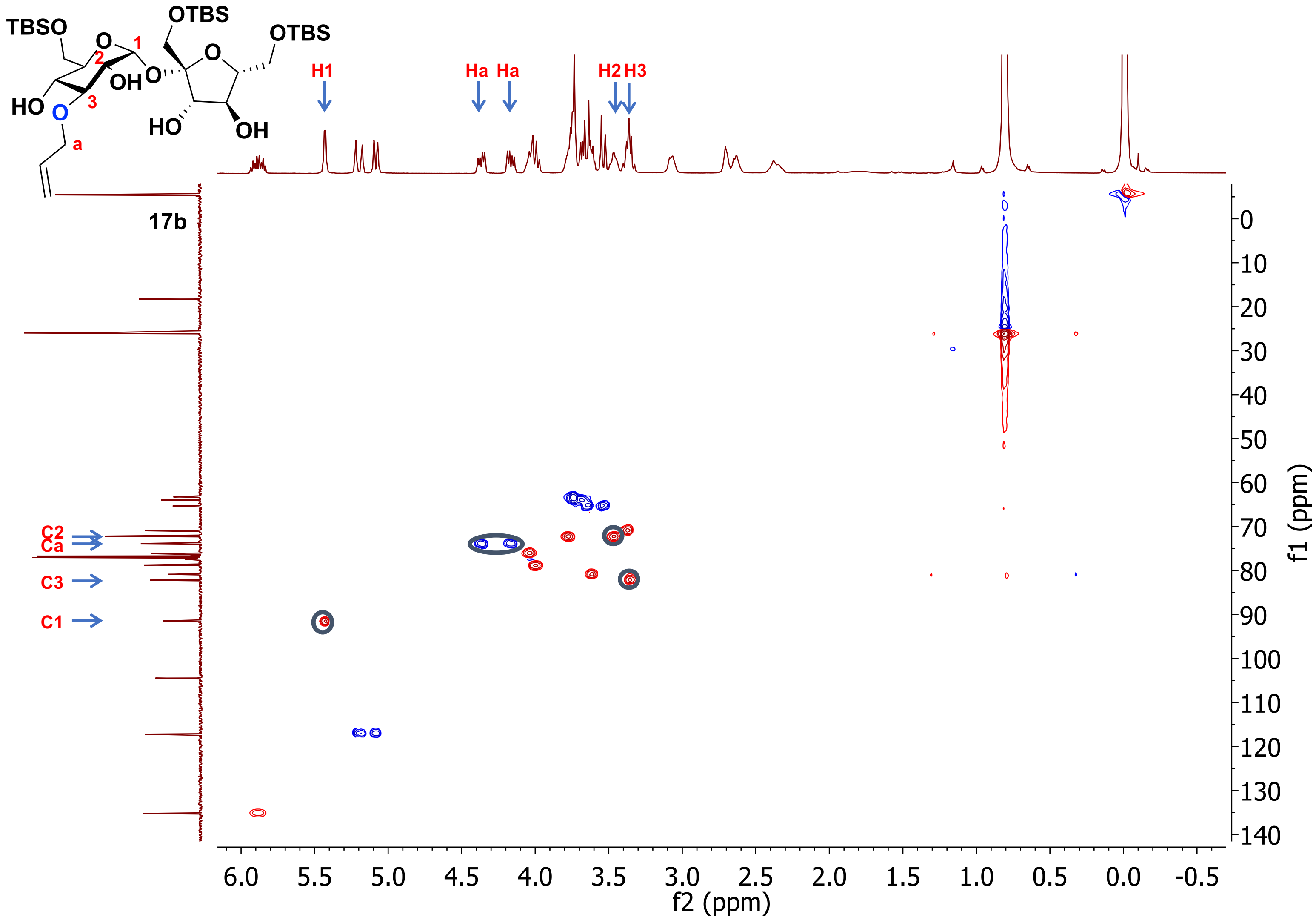
TBSO
OTBS
OTBS
1
2
3
OH
HO
HO
OH
a
17b
H1
Ha Ha
H2 H3
C2
Ca
C3
C1
6.0 5.5 5.0 4.5 4.0 3.5 3.0 2.5 2.0 1.5 1.0 0.5 0.0 -0.5
f2 (ppm)
0 10 20 30 40 50 60 70 80 90 100 110 120 130 140
f1 (ppm)

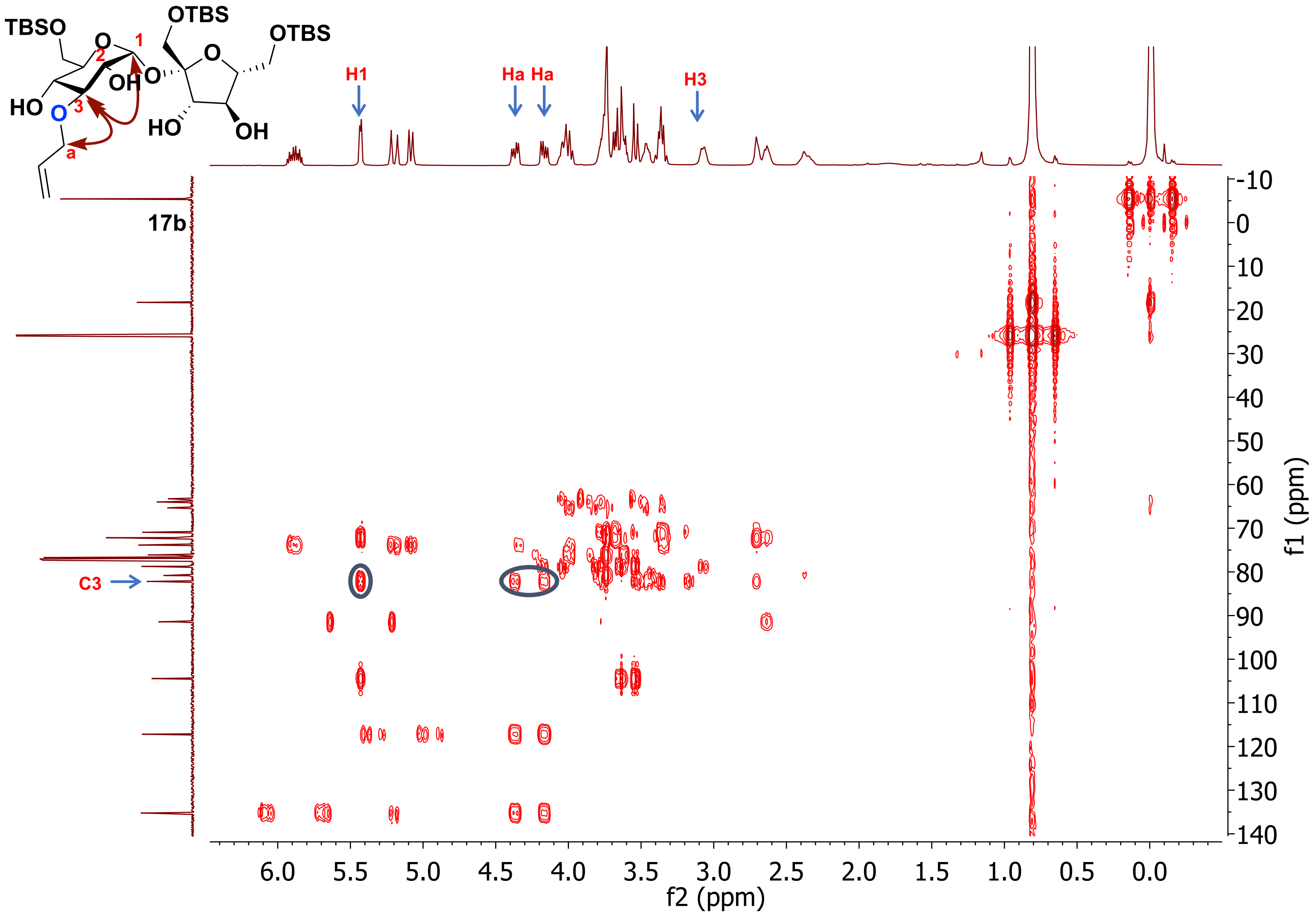

TBSO
OTBS
OTBS
O
1
2
OH
O
HO
O
3
HO
OH
a
17b
H1
Ha
Ha
H3
C3
f2 (ppm)
f1 (ppm)

CDCl3, 400.13 MHz

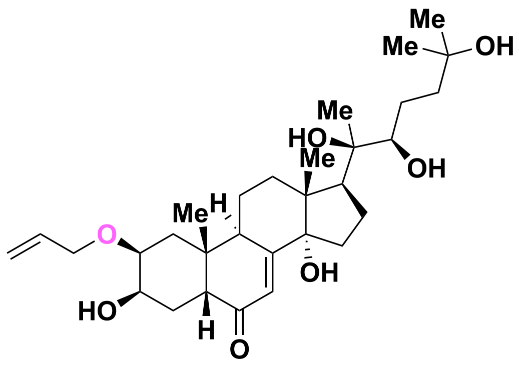

18a

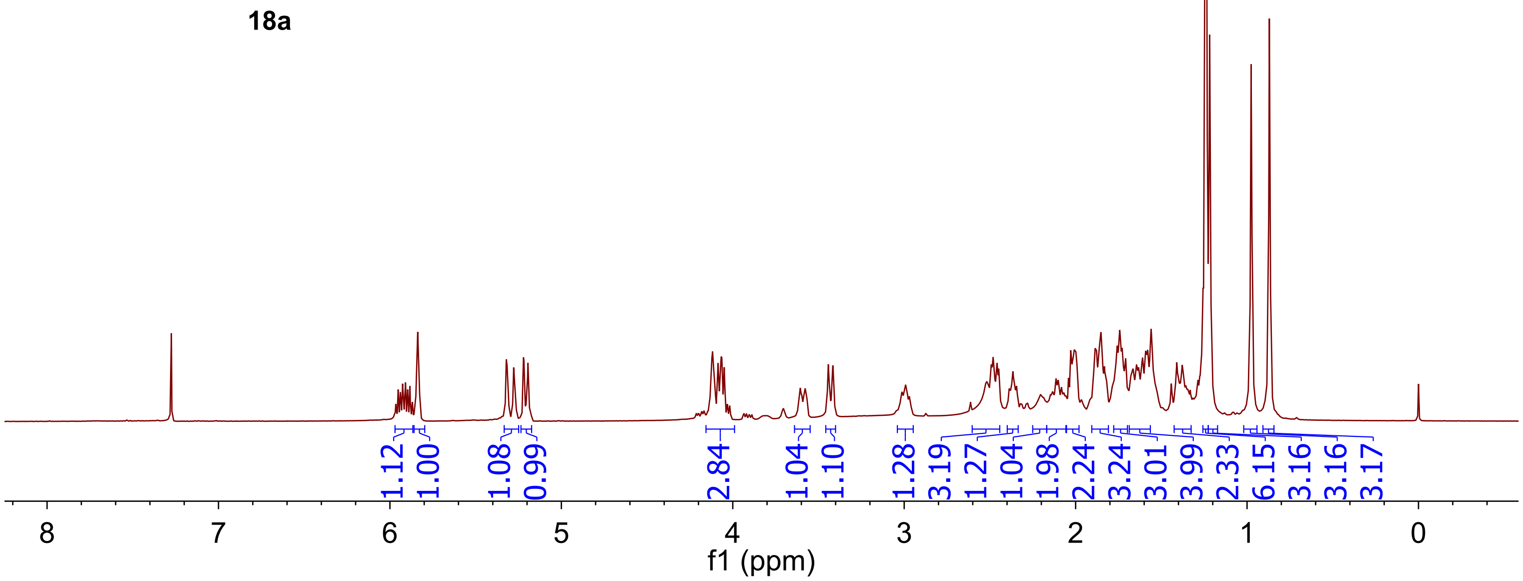

CDCl3, 100.62 MHz

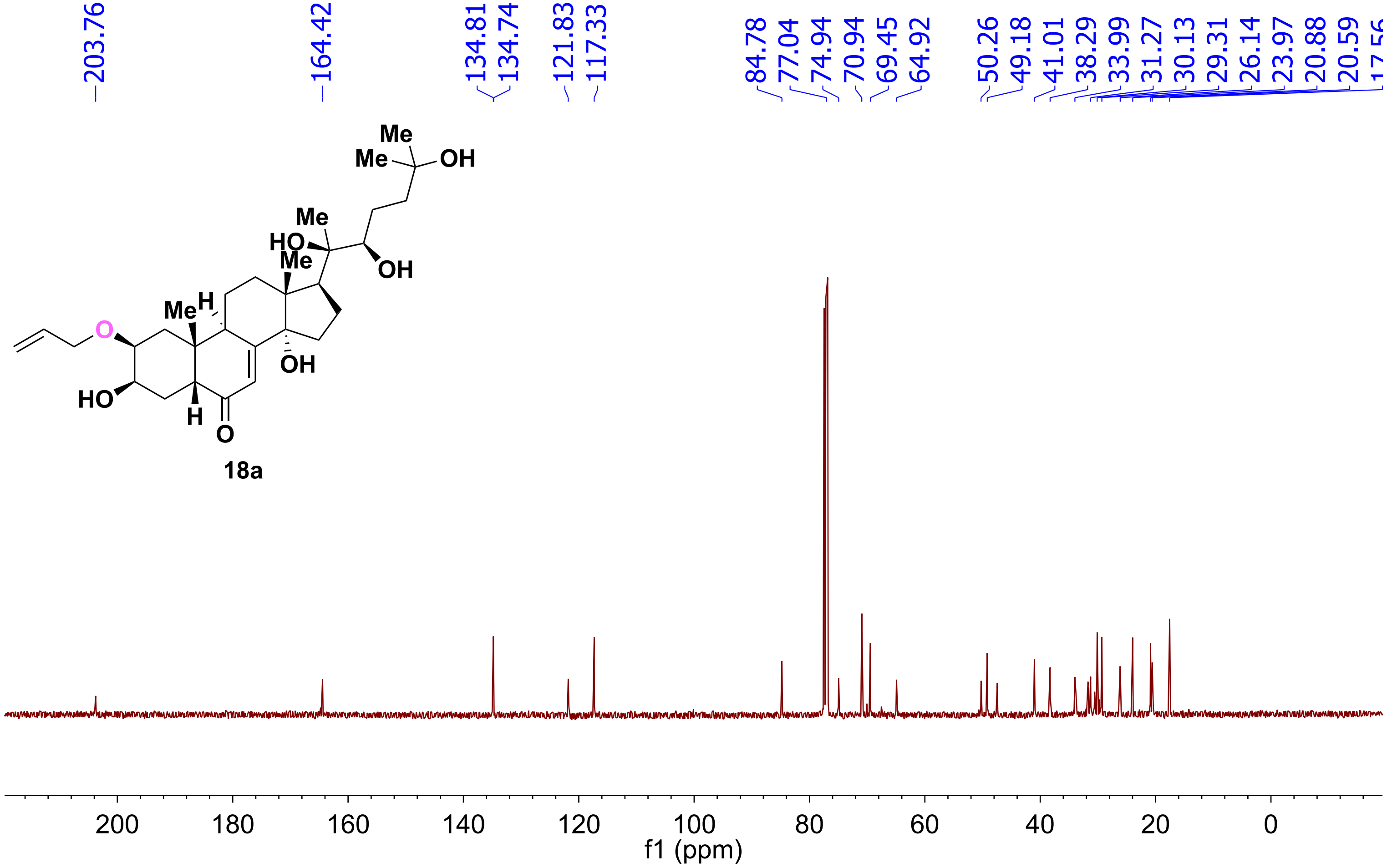

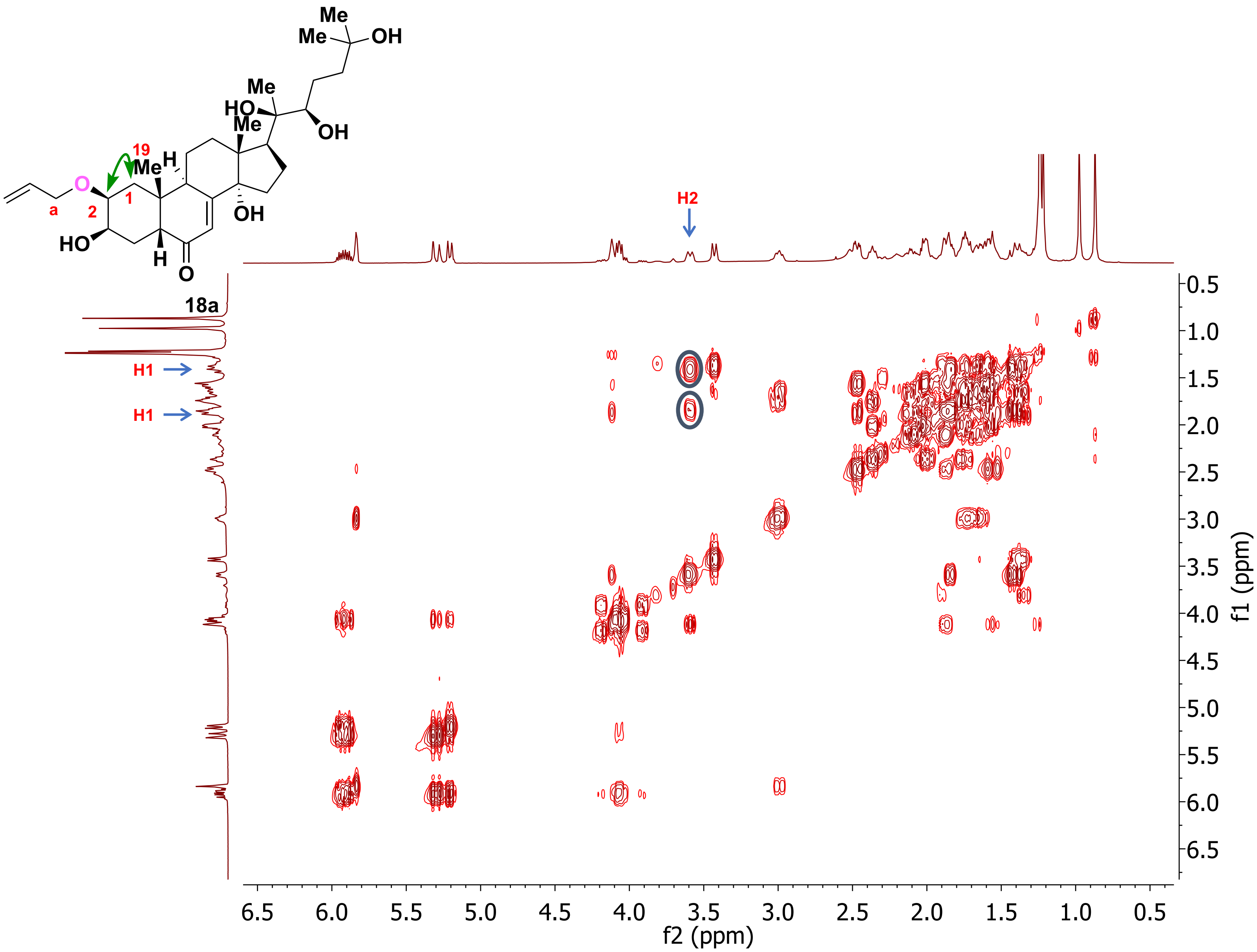

Me
Me
OH
Me
HO
Me
OH
19
Me
H
O
a
2
1
OH
HO
H
O
18a
H2
H1
H1
6.5
6.0
5.5
5.0
4.5
4.0
3.5
3.0
2.5
2.0
1.5
1.0
0.5
f2 (ppm)
f1 (ppm)

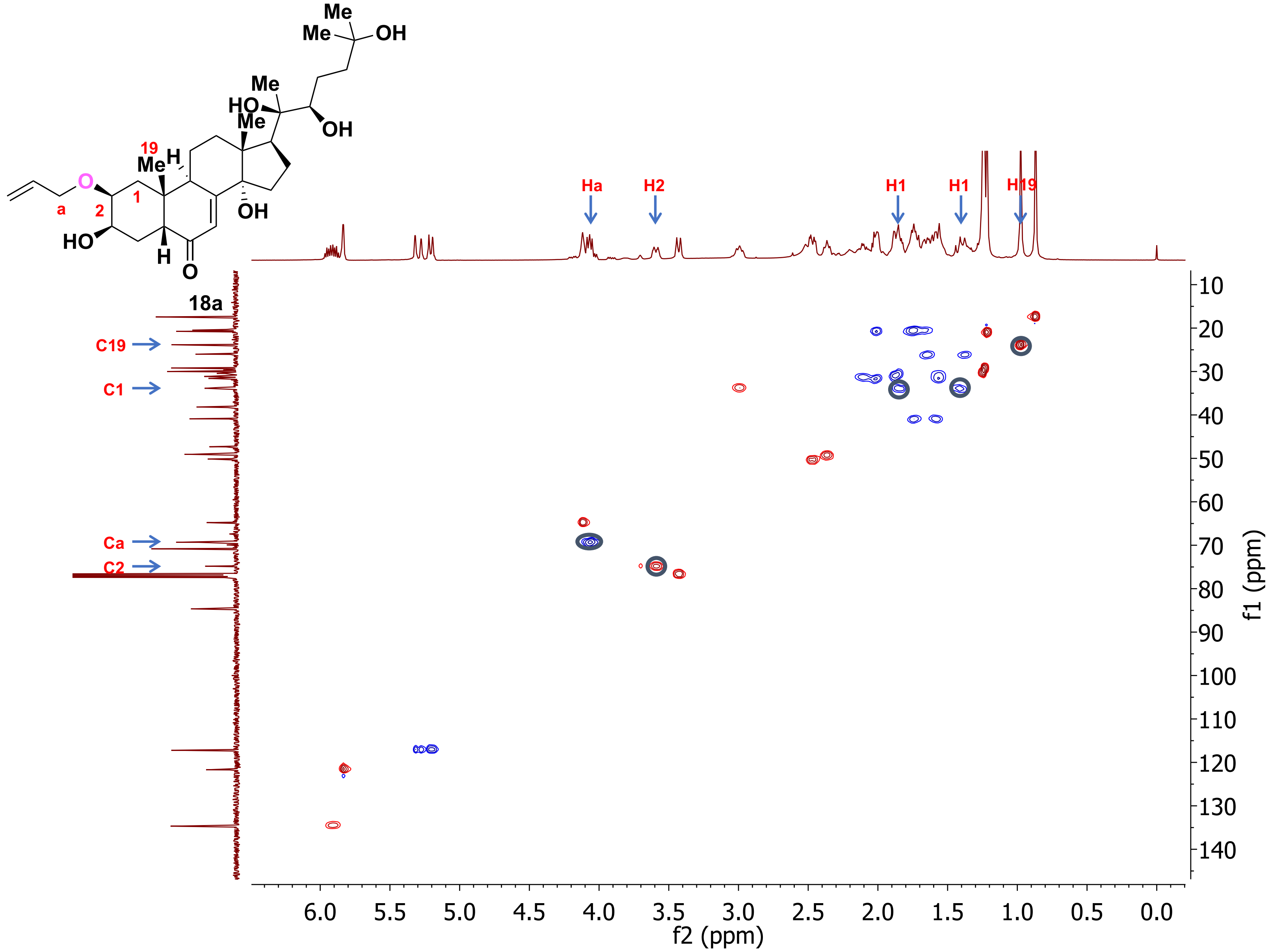

Me
Me
OH
Me
HO
Me
OH
19
Me
H
O
a
2
1
OH
HO
H
O
18a
Ha
H2
H1
H1
H19
C19
C1
Ca
C2
6.0
5.5
5.0
4.5
4.0
3.5
3.0
2.5
2.0
1.5
1.0
0.5
0.0
f2 (ppm)
10
20
30
40
50
60
70
80
90
100
110
120
130
140
f1 (ppm)

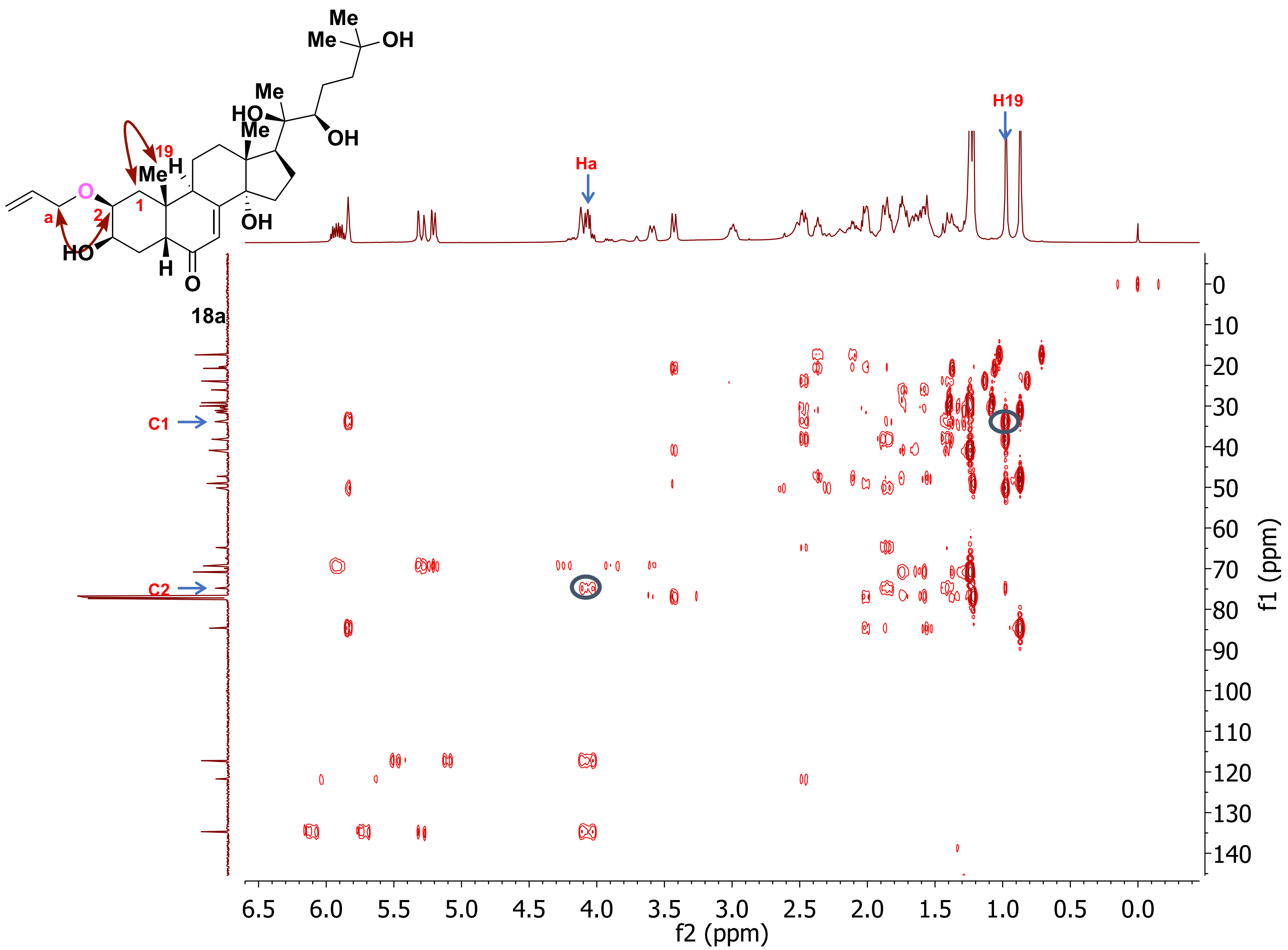
Me
Me
OH
Me
HO
Me
OH
19
Me
H
O
a
2
1
HO
H
O
OH
18a
Ha
H19
C1
C2
f2 (ppm)
f1 (ppm)

CDCl3, 400.13 MHz

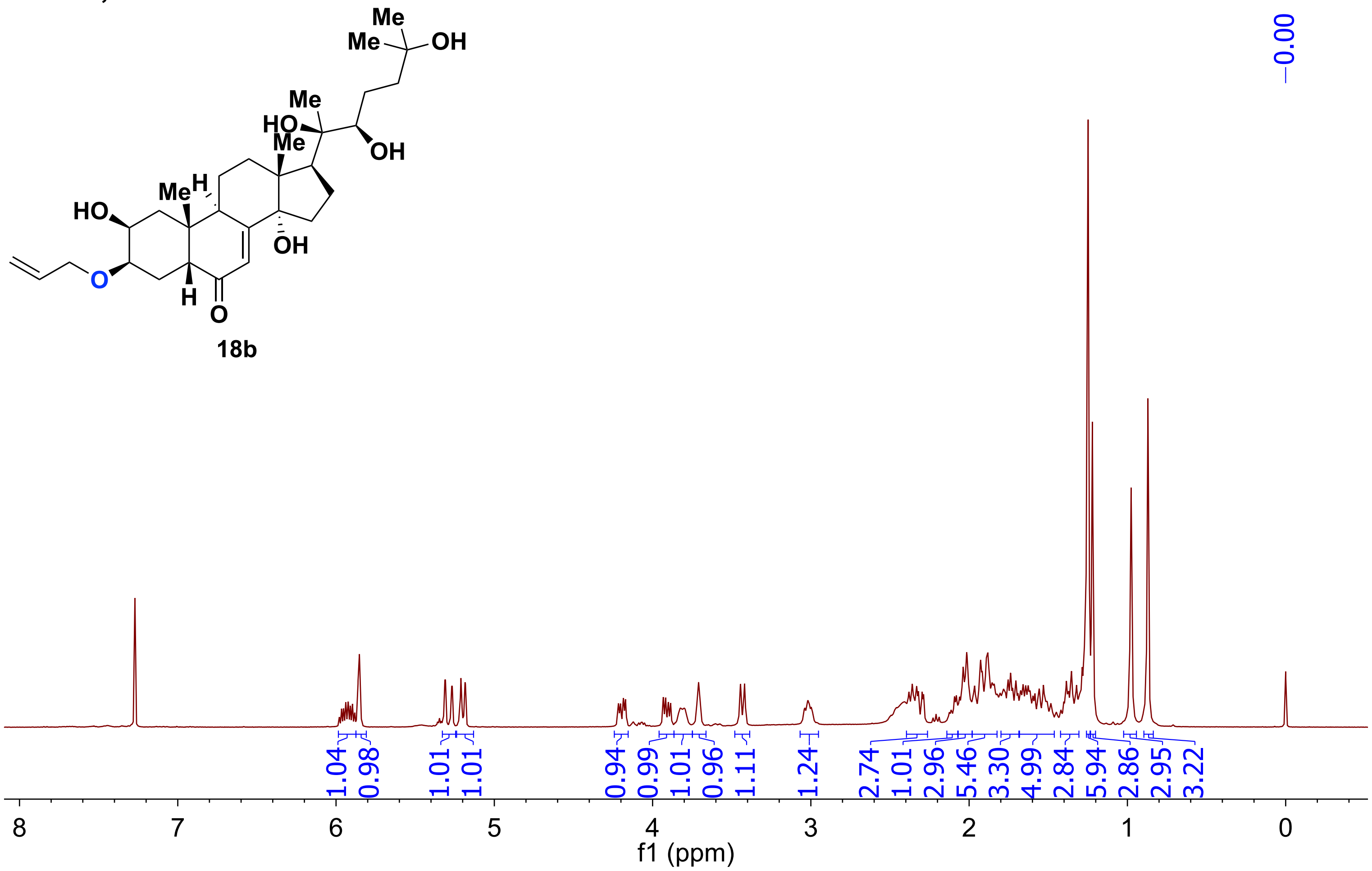

CDCl3, 100.62 MHz

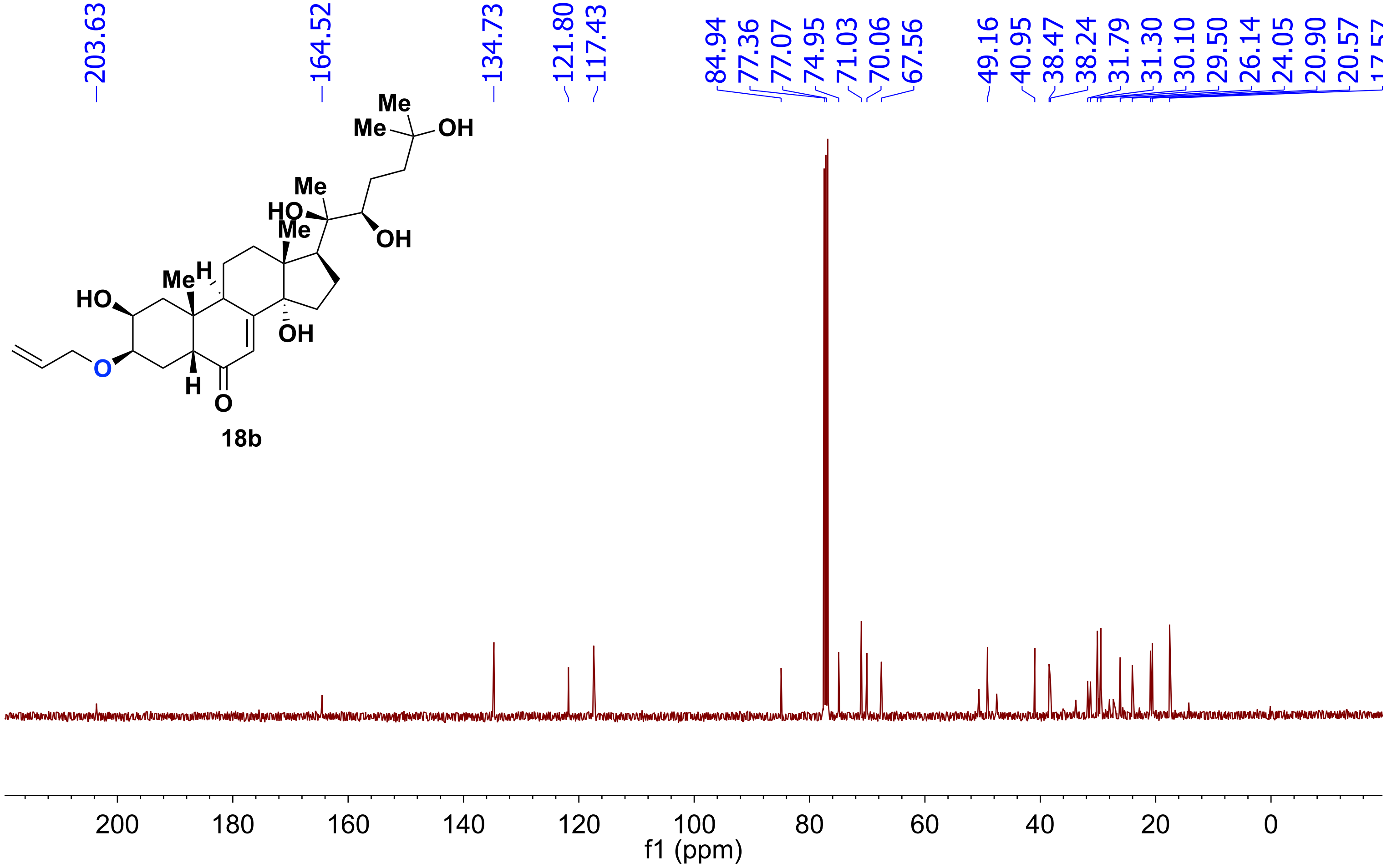

18b

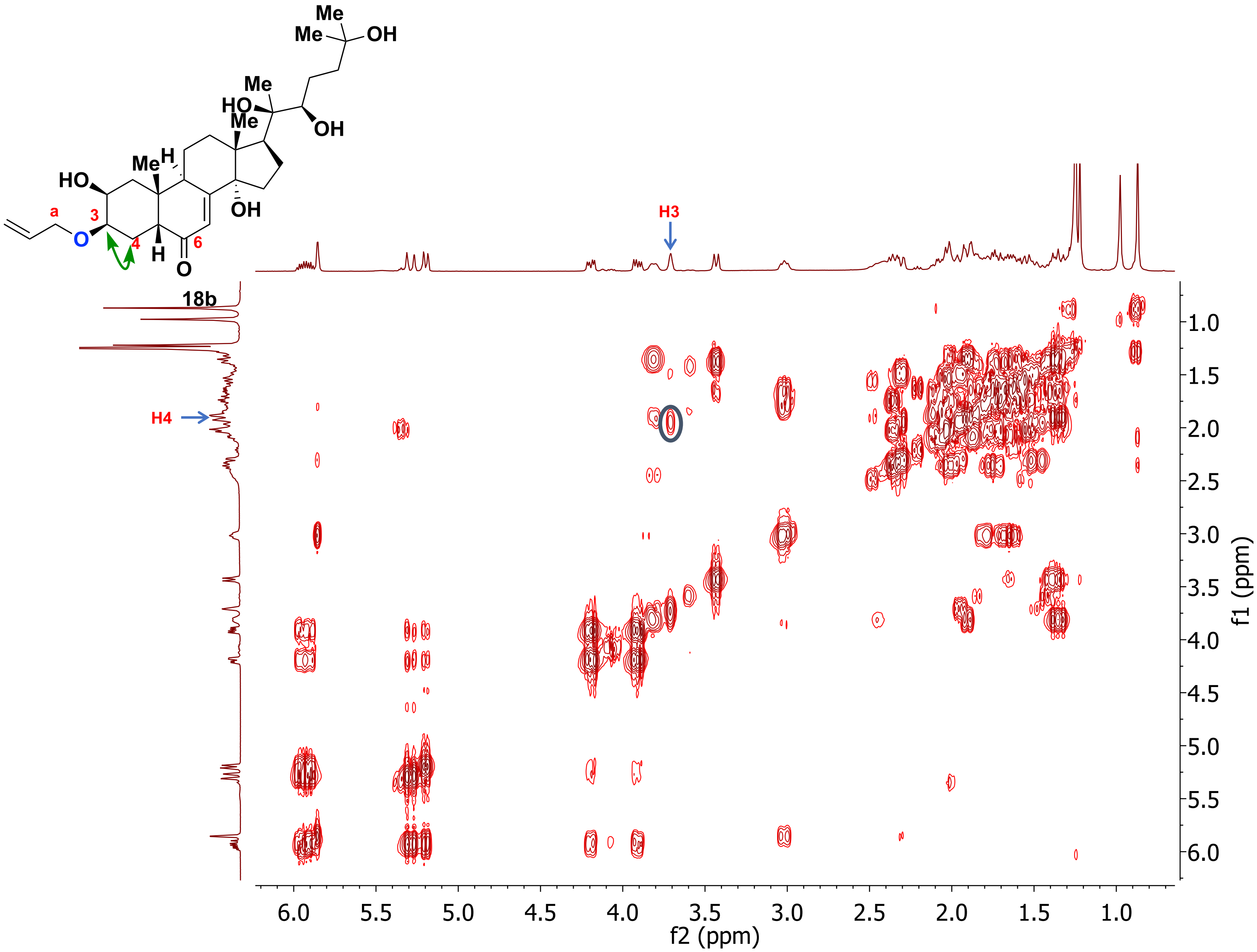
Me
Me
OH
Me
HO
Me
OH
Me
H
HO
a
3
O
4
H
6
O
OH
18b
H3
H4
6.0
5.5
5.0
4.5
4.0
3.5
3.0
2.5
2.0
1.5
1.0
f2 (ppm)
f1 (ppm)

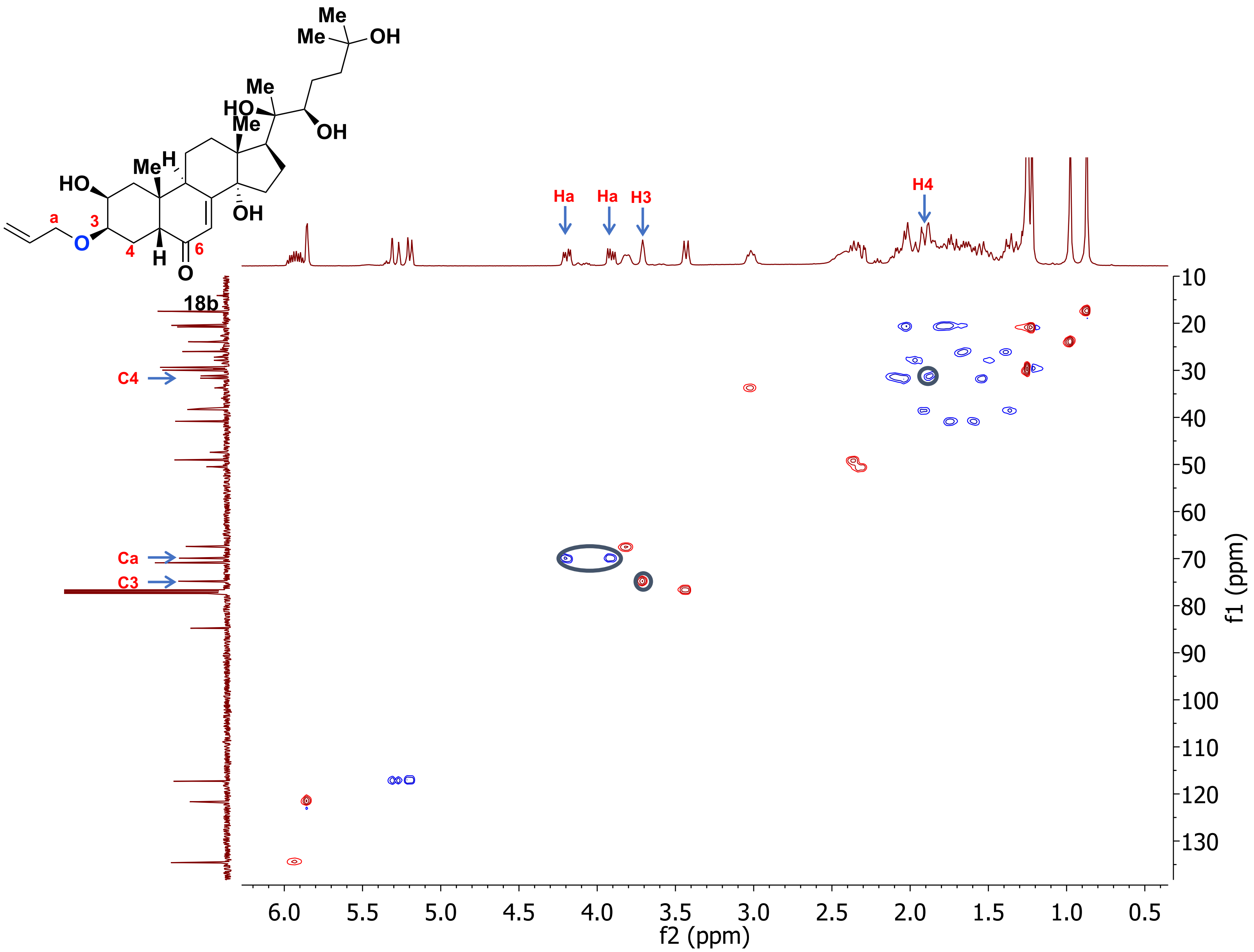
18b
Ha
Ha
H3
H4
C4
Ca
C3
f2 (ppm)
f1 (ppm)

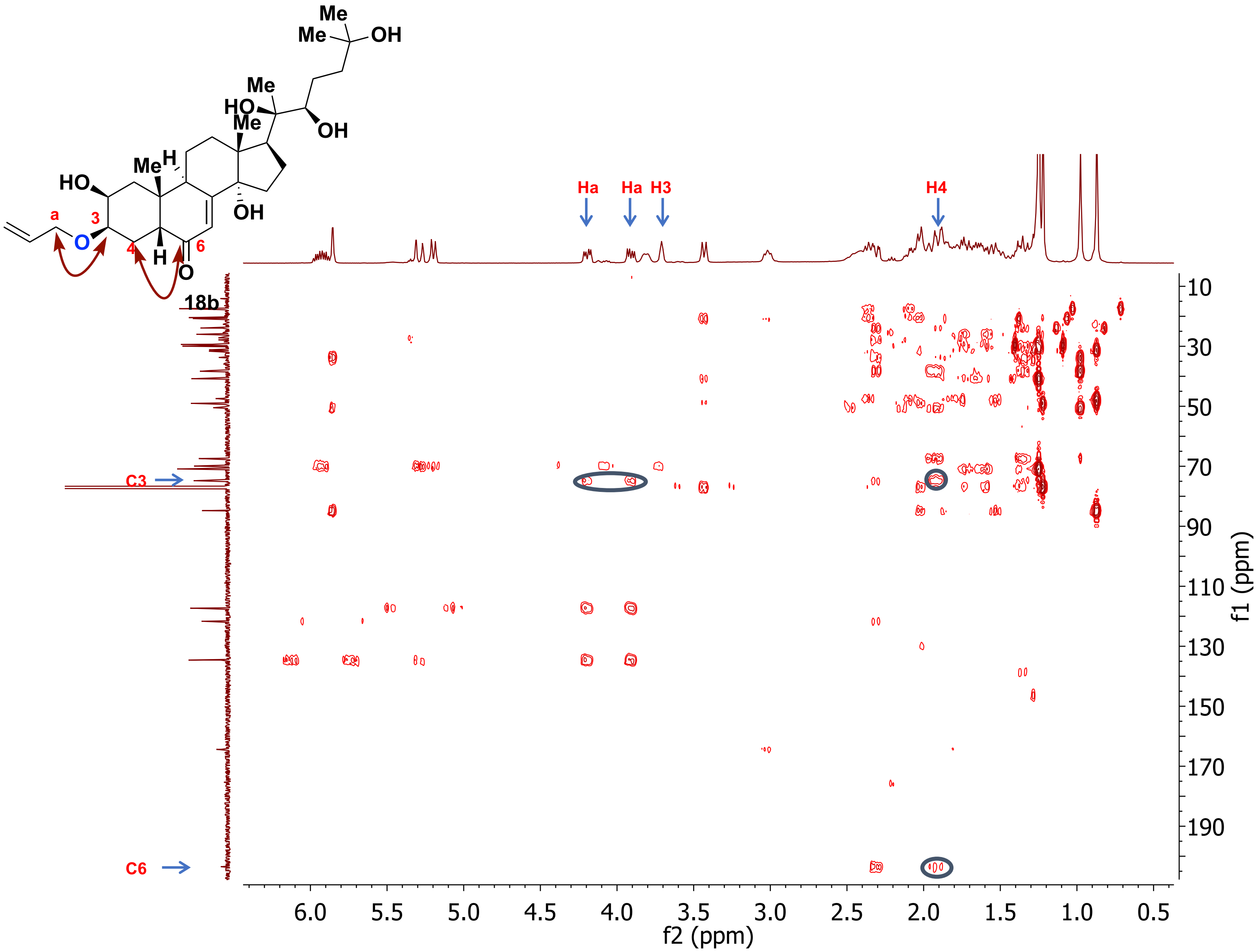
Me
Me
OH
Me
HO
Me
OH
Me
H
HO
a
3
O
4
H
6
O
OH
18b
Ha
Ha
H3
H4
C3
C6
f2 (ppm)
f1 (ppm)

CDCl3, 400.13 MHz

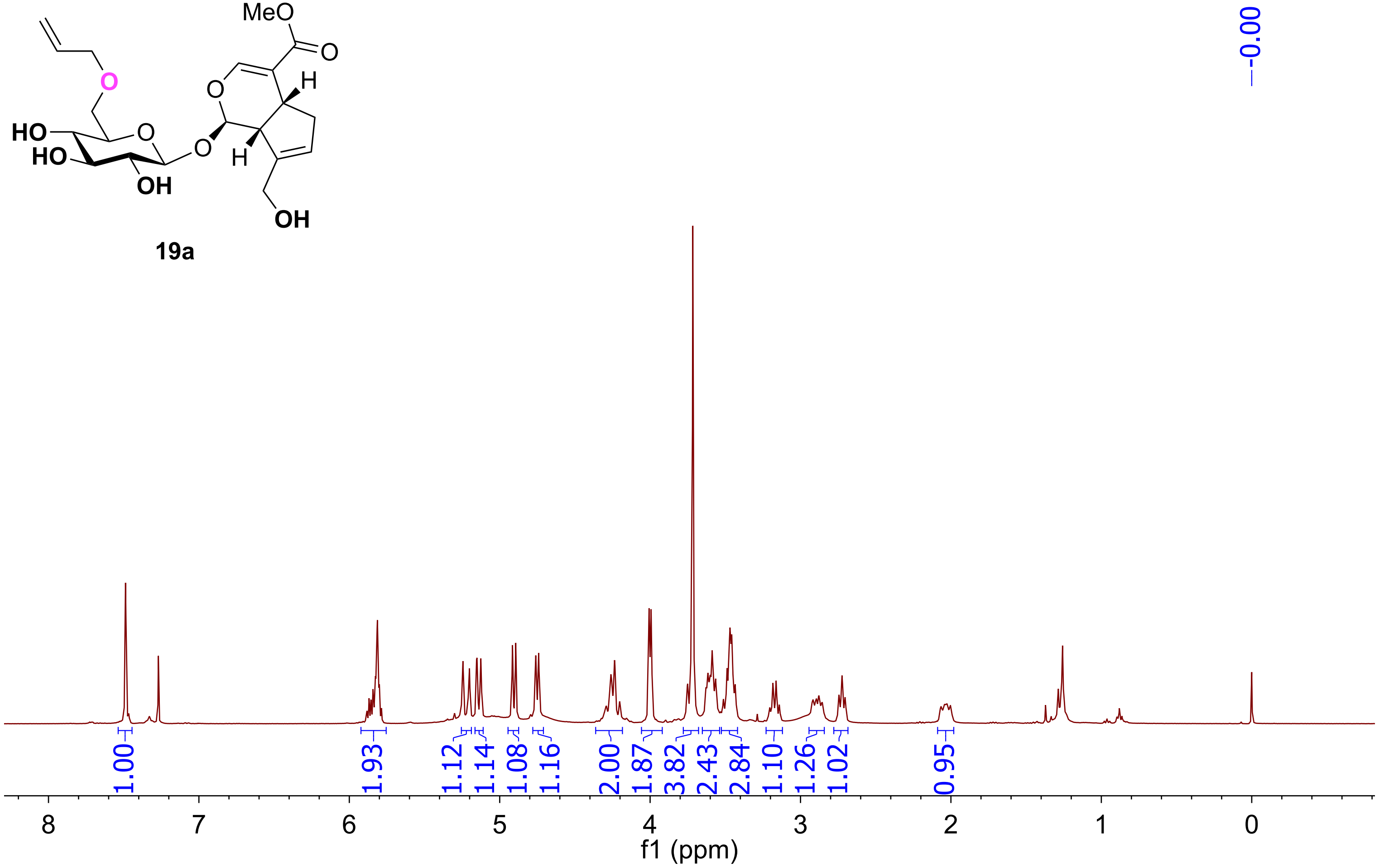

CDCl3, 100.62 MHz

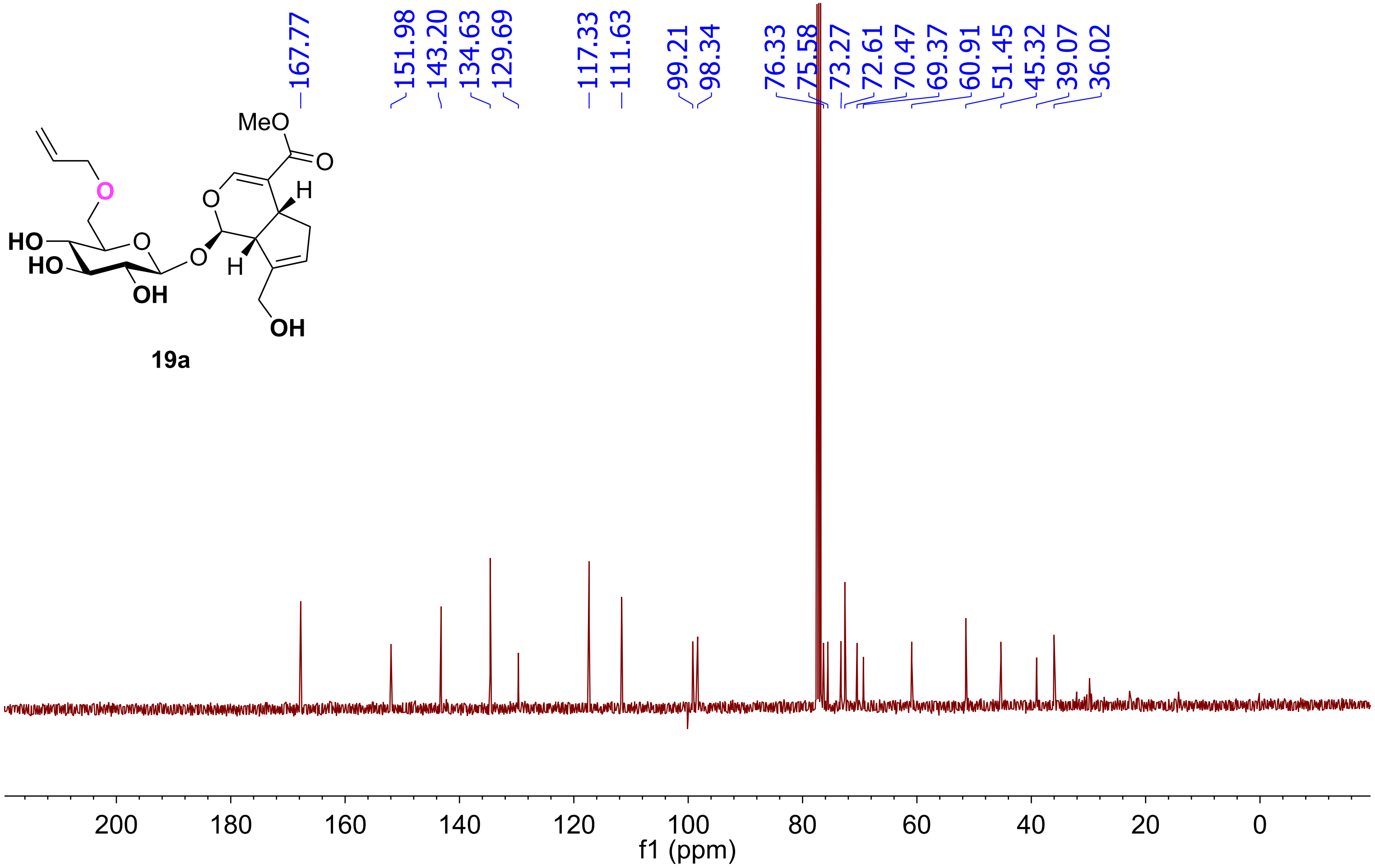

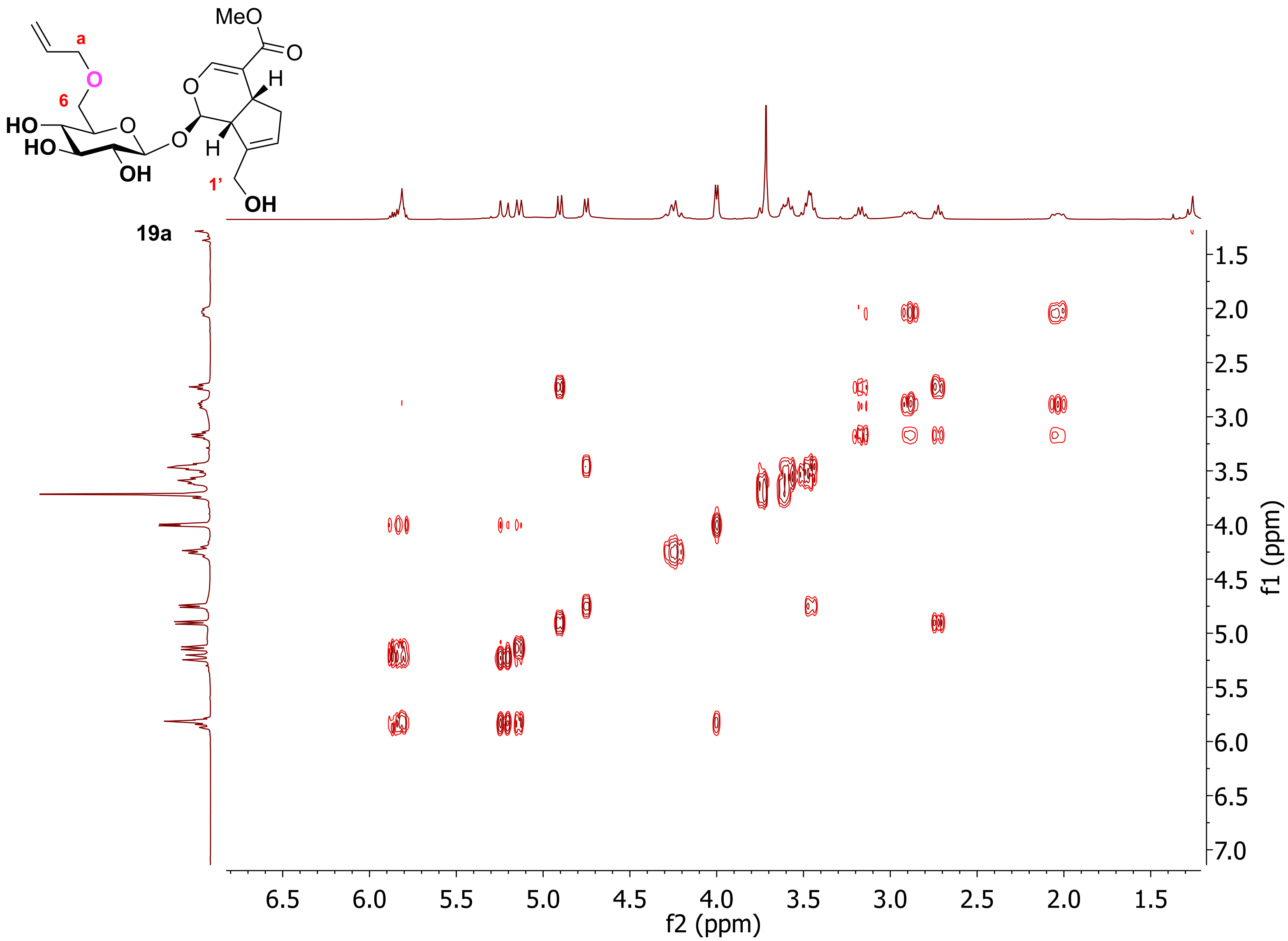
MeO
O
a
O
6
HO
HO
OH
O
O
O
H
H
1'
OH
19a
f2 (ppm)
f1 (ppm)
6.5
6.0
5.5
5.0
4.5
4.0
3.5
3.0
2.5
2.0
1.5
7.0

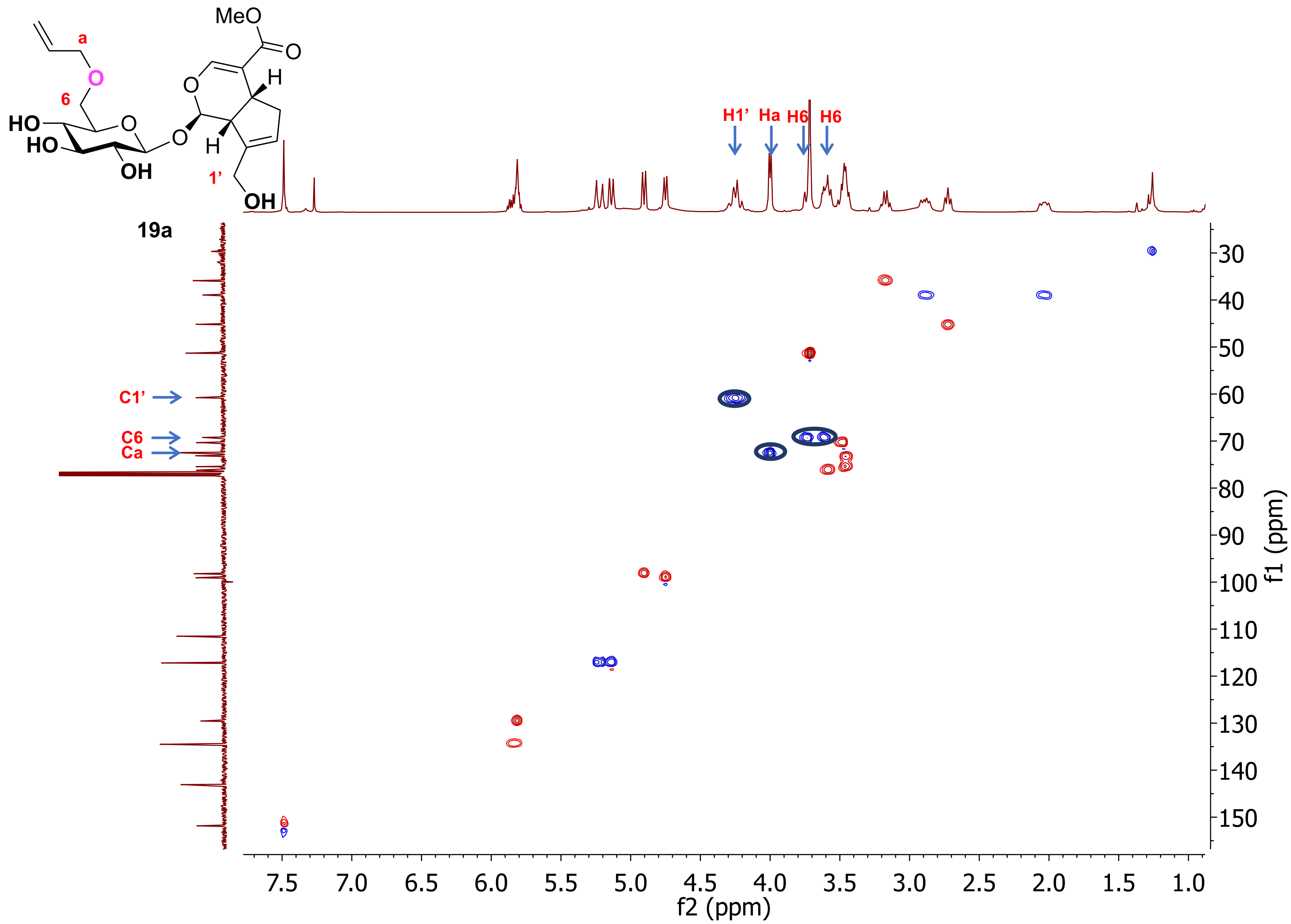

MeO
O
H
O
a
O
6
HO
O
O
HO
H
OH
1'
OH
H1'
Ha
H6
H6
C1'
C6
Ca
30
40
50
60
70
80
90
100
110
120
130
140
150
f1 (ppm)
7.5
7.0
6.5
6.0
5.5
5.0
4.5
4.0
3.5
3.0
2.5
2.0
1.5
1.0
f2 (ppm)

19a

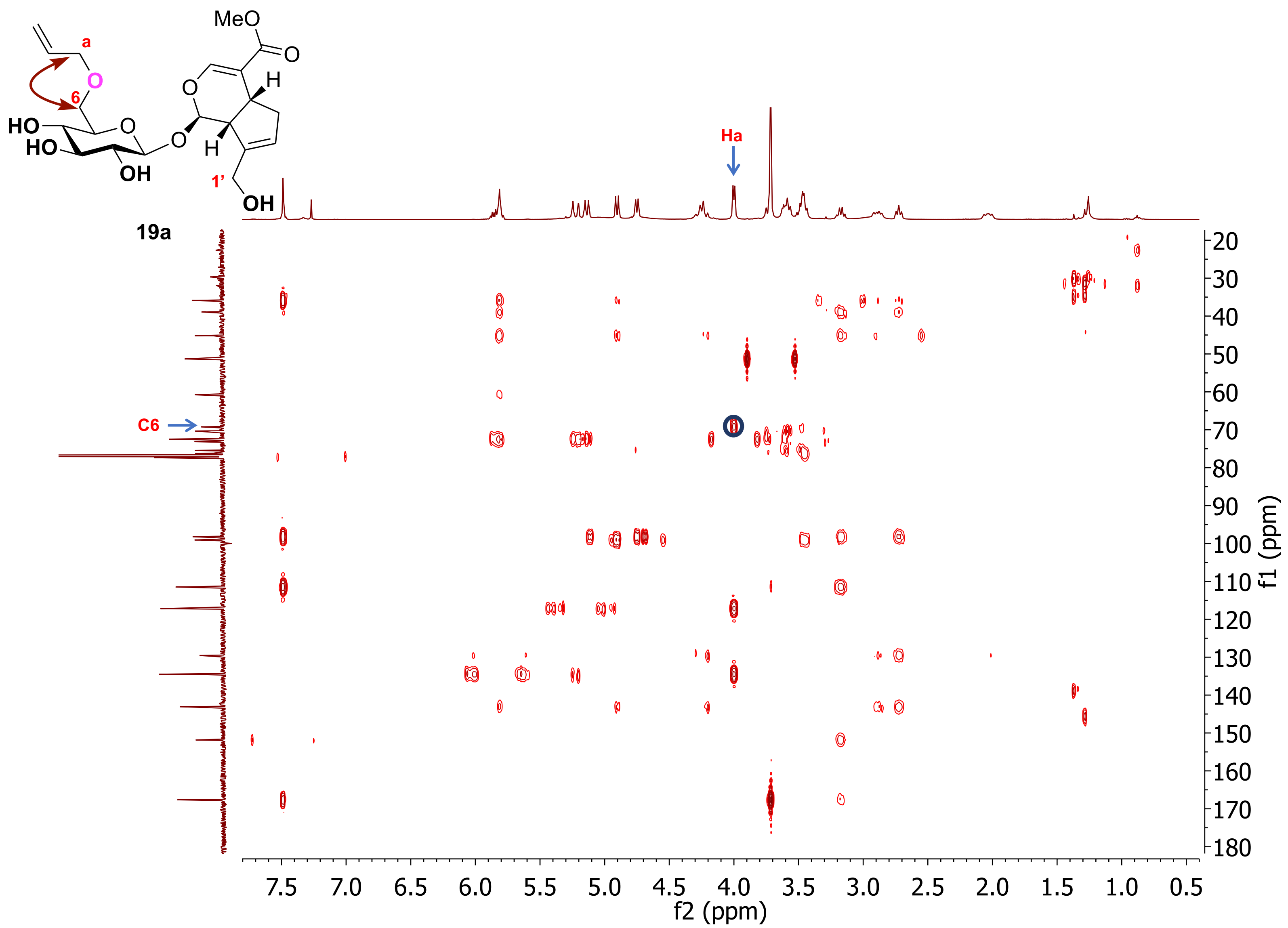
MeO
O
O
H
H
a
O
6
HO
O
HO
OH
O
1'
OH
19a
Ha
C6
f2 (ppm)
f1 (ppm)

CDCl3, 400.13 MHz

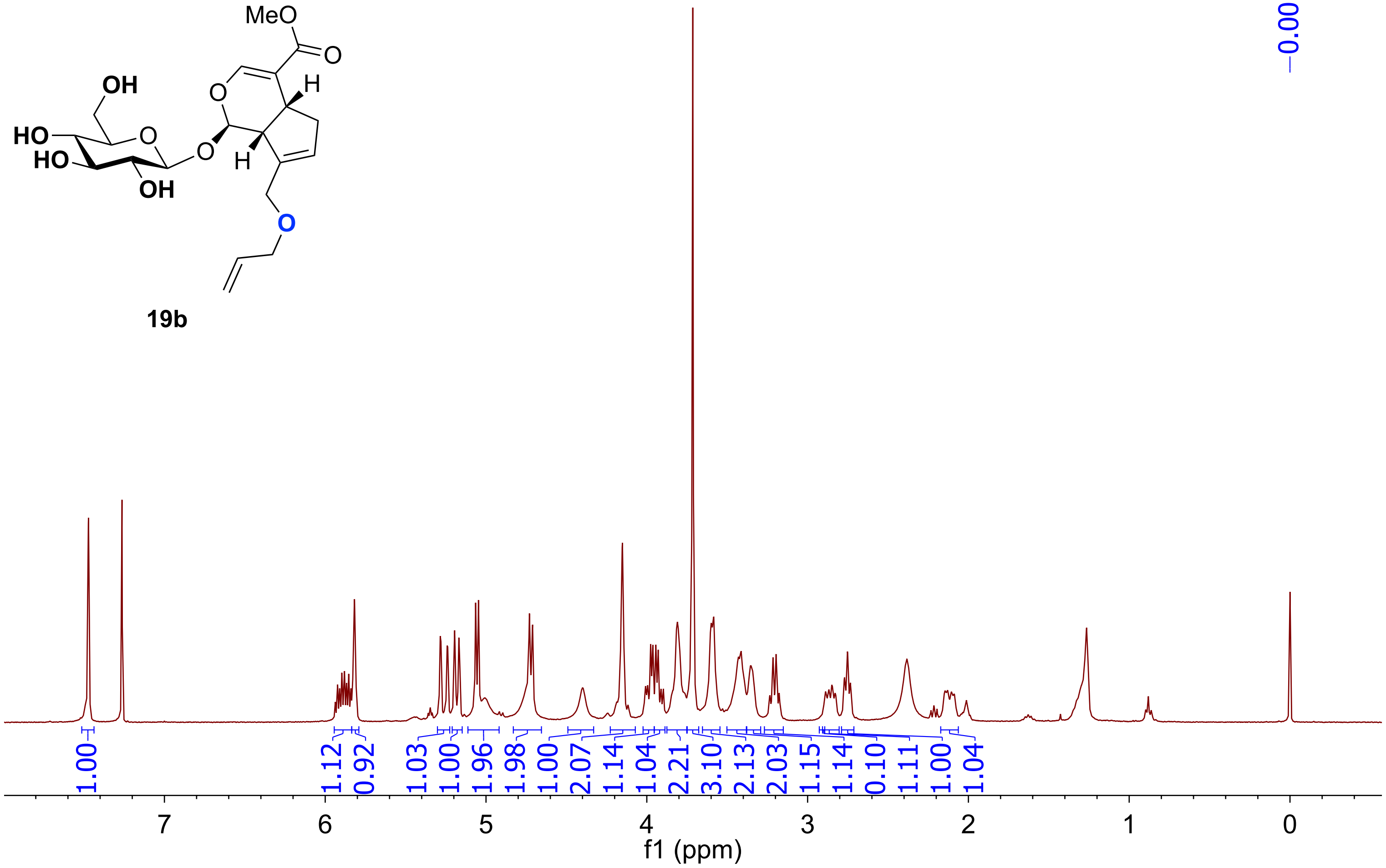

CDCl3, 100.62 MHz

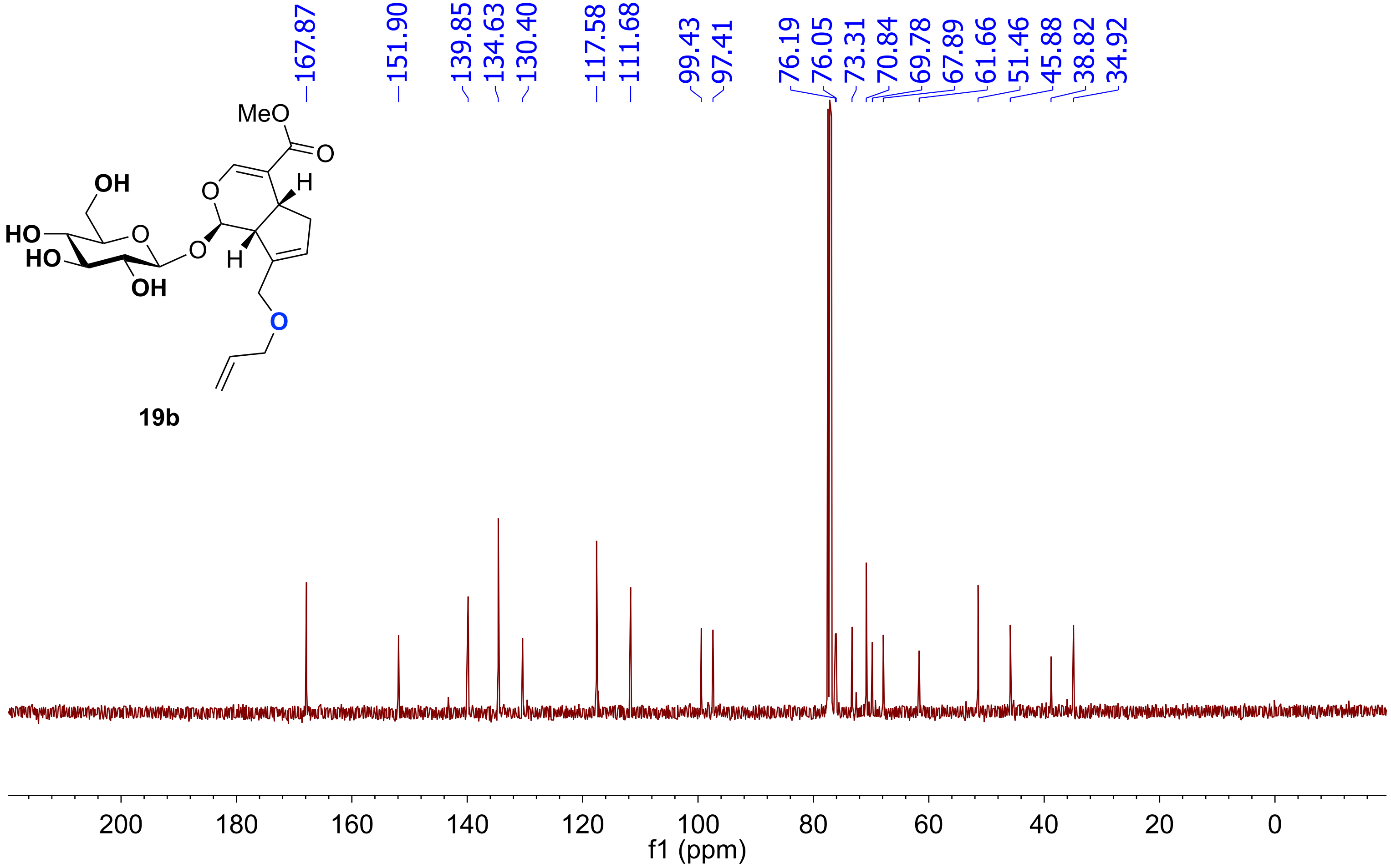

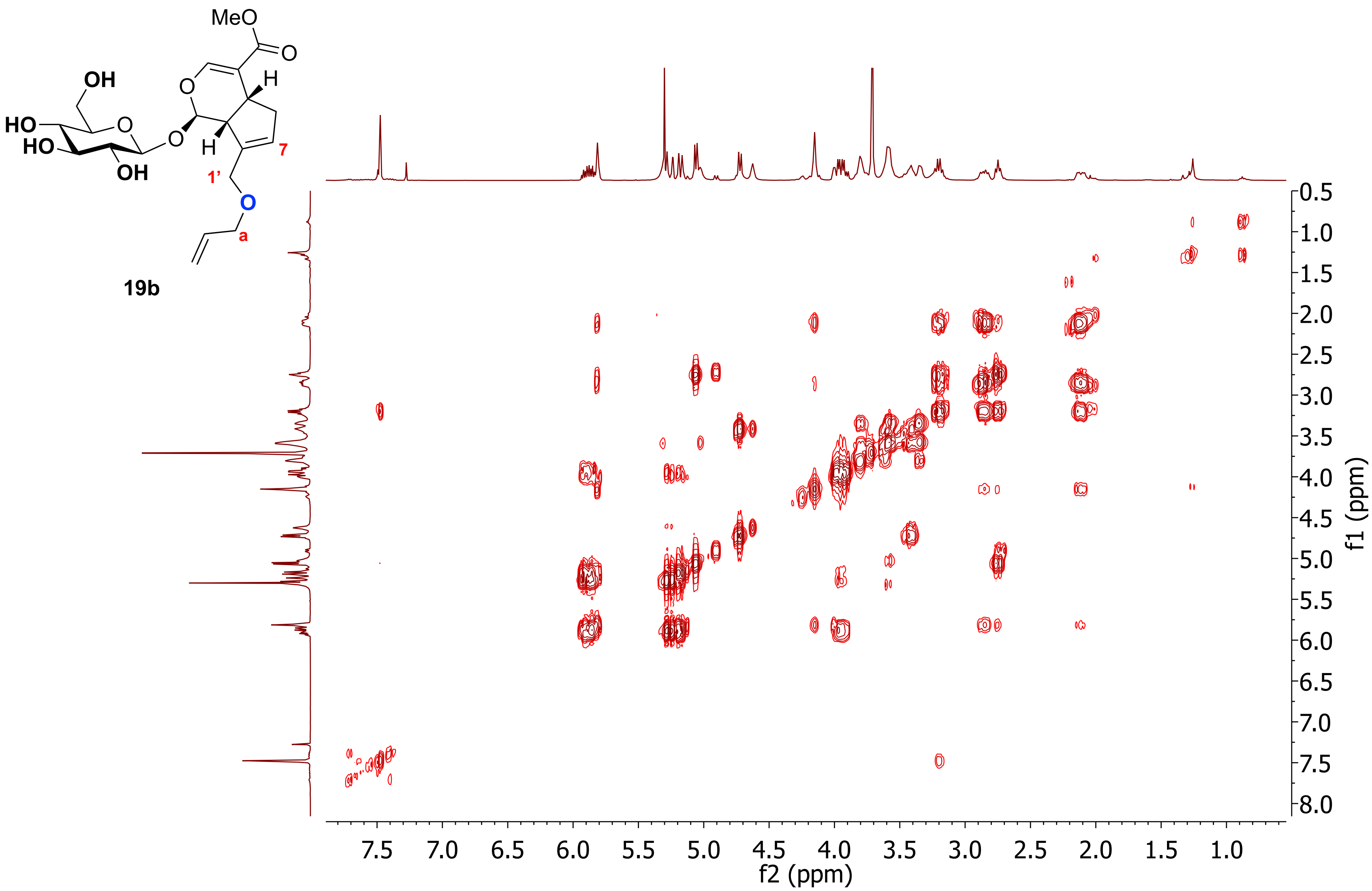
MeO
O
H
OH
HO
HO
OH
O
O
O
H
7
1'
O
a
19b
f2 (ppm)
f1 (ppm)
7.5
7.0
6.5
6.0
5.5
5.0
4.5
4.0
3.5
3.0
2.5
2.0
1.5
1.0
0.5
8.0

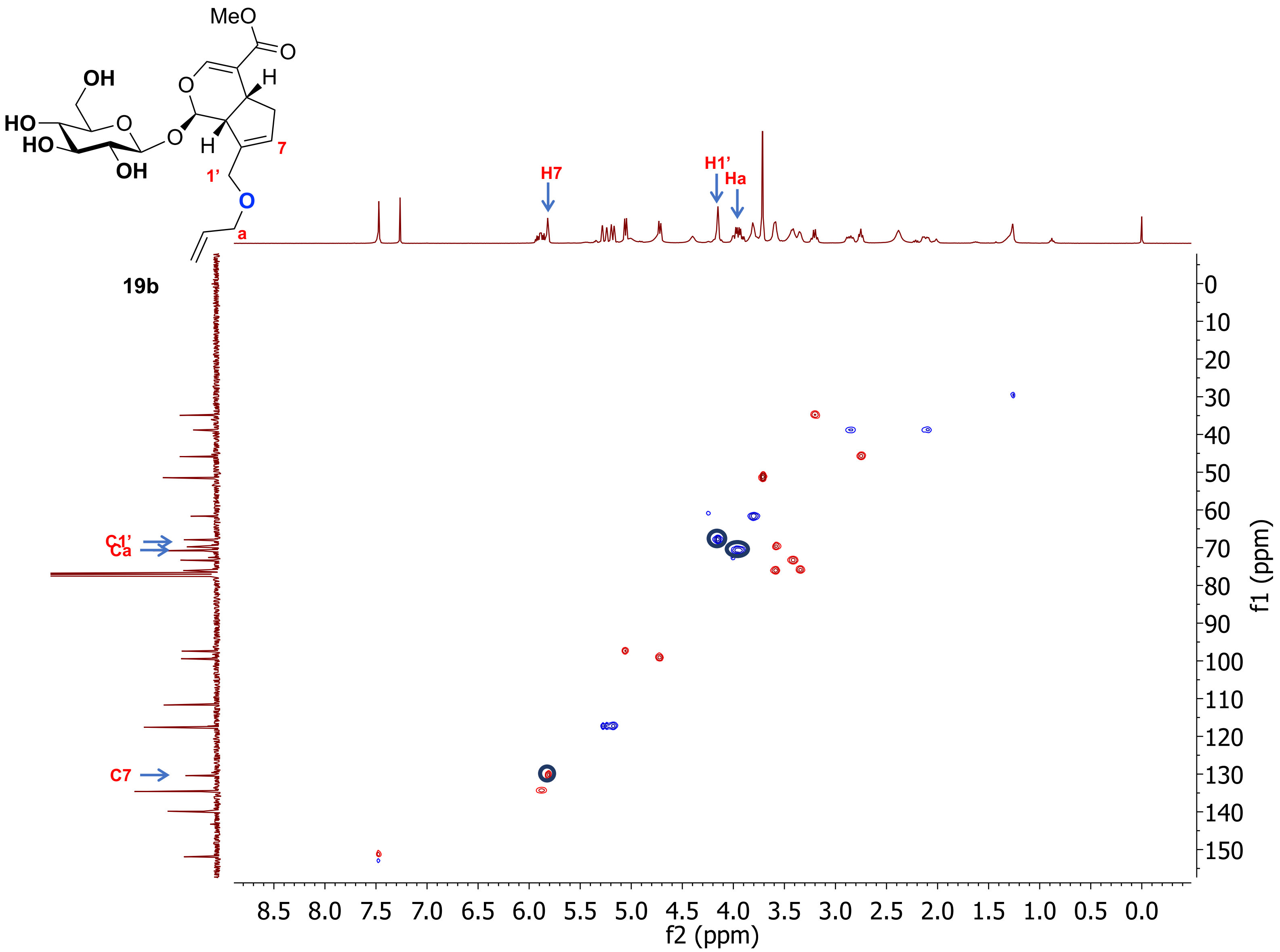
MeO
O
OH
HO
HO
OH
H
7
1'
O
a
19b
H7
H1'
Ha
C1'
Ca
C7
0
10
20
30
40
50
60
70
80
90
100
110
120
130
140
150
f1 (ppm)
8.5 8.0 7.5 7.0 6.5 6.0 5.5 5.0 4.5 4.0 3.5 3.0 2.5 2.0 1.5 1.0 0.5 0.0
f2 (ppm)

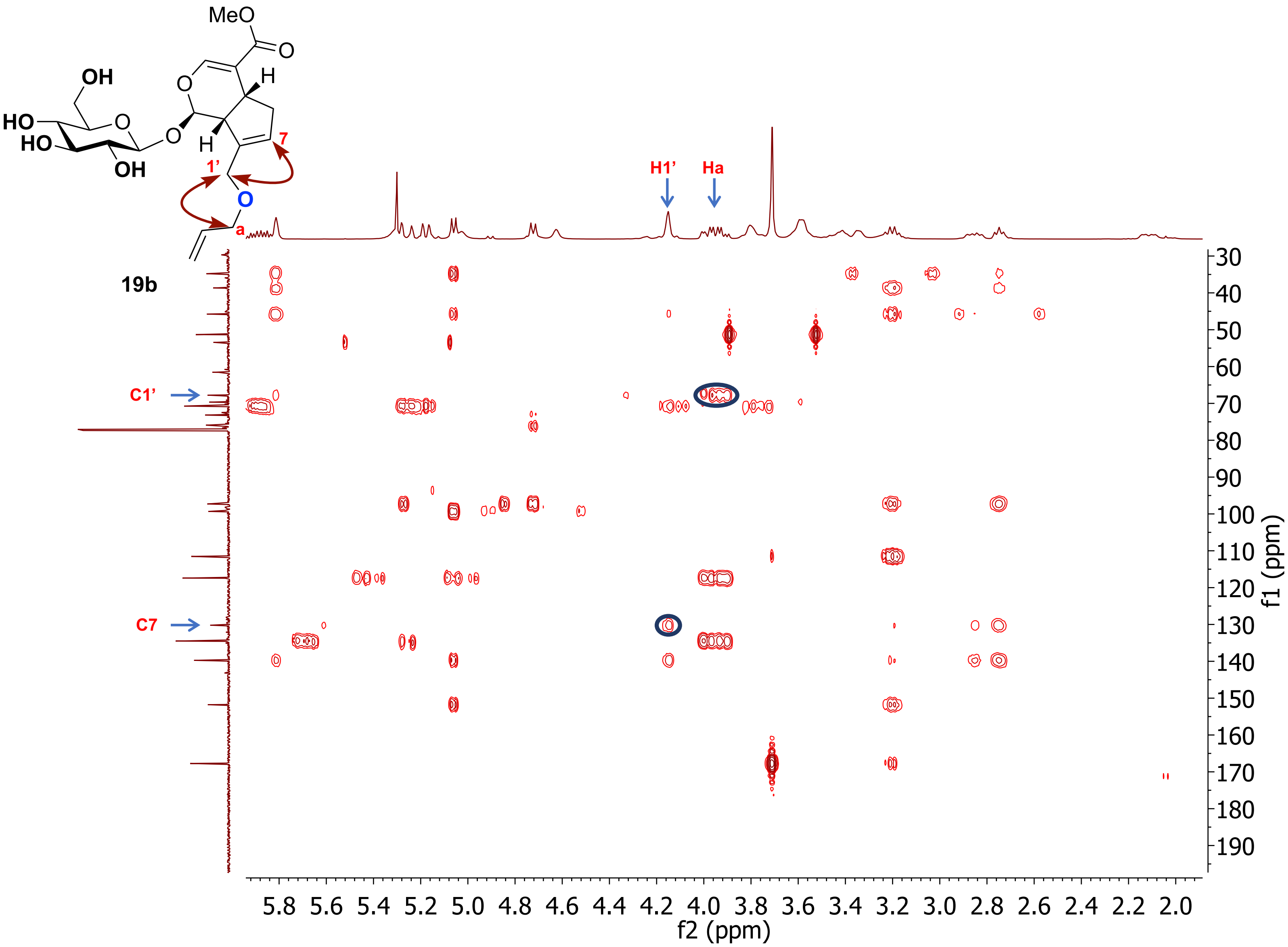
MeO
OH
HO
HO
OH
H
7
1'
a
19b
H1'
Ha
C1'
C7
f1 (ppm)
f2 (ppm)

MeOD, 400.13 MHz

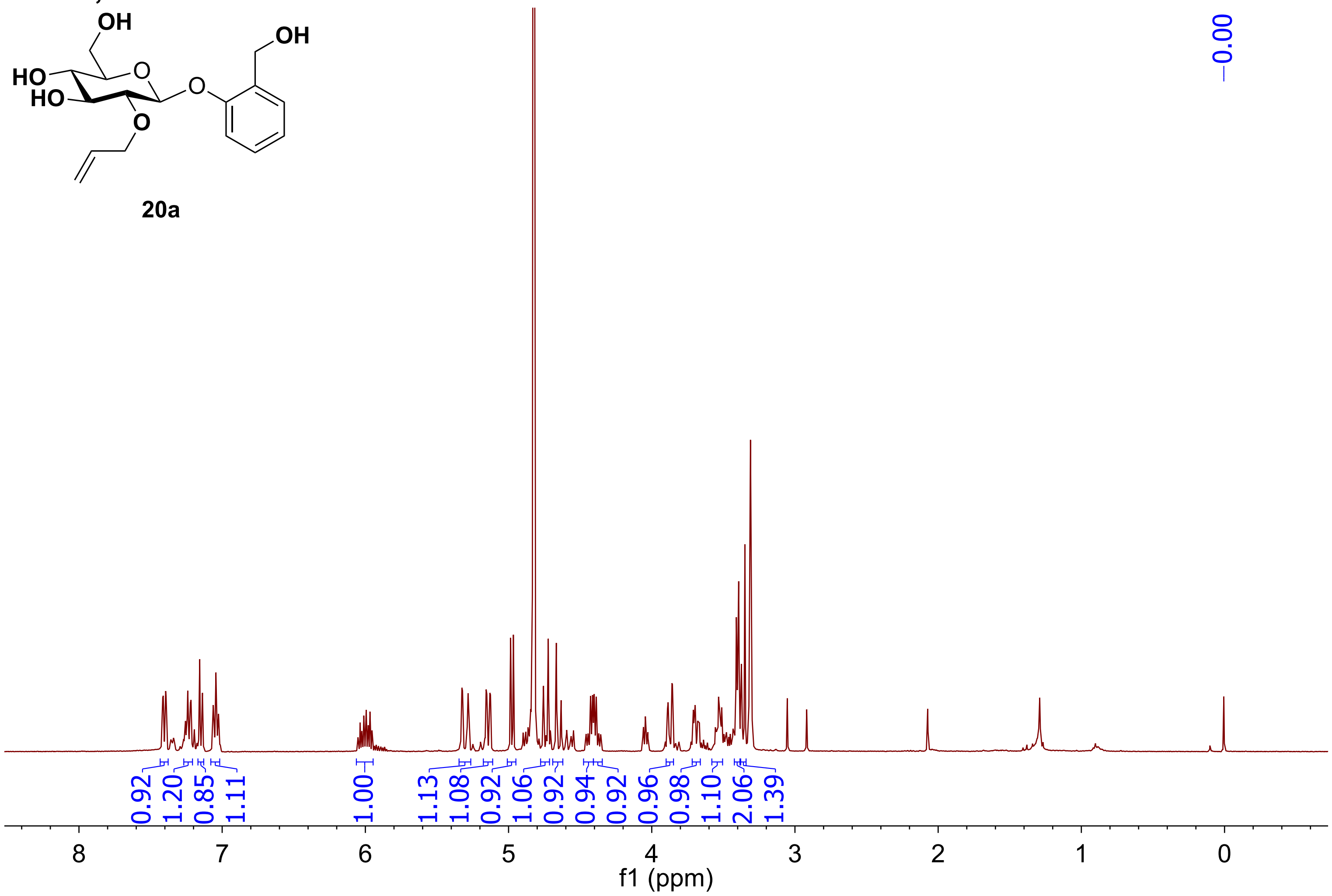

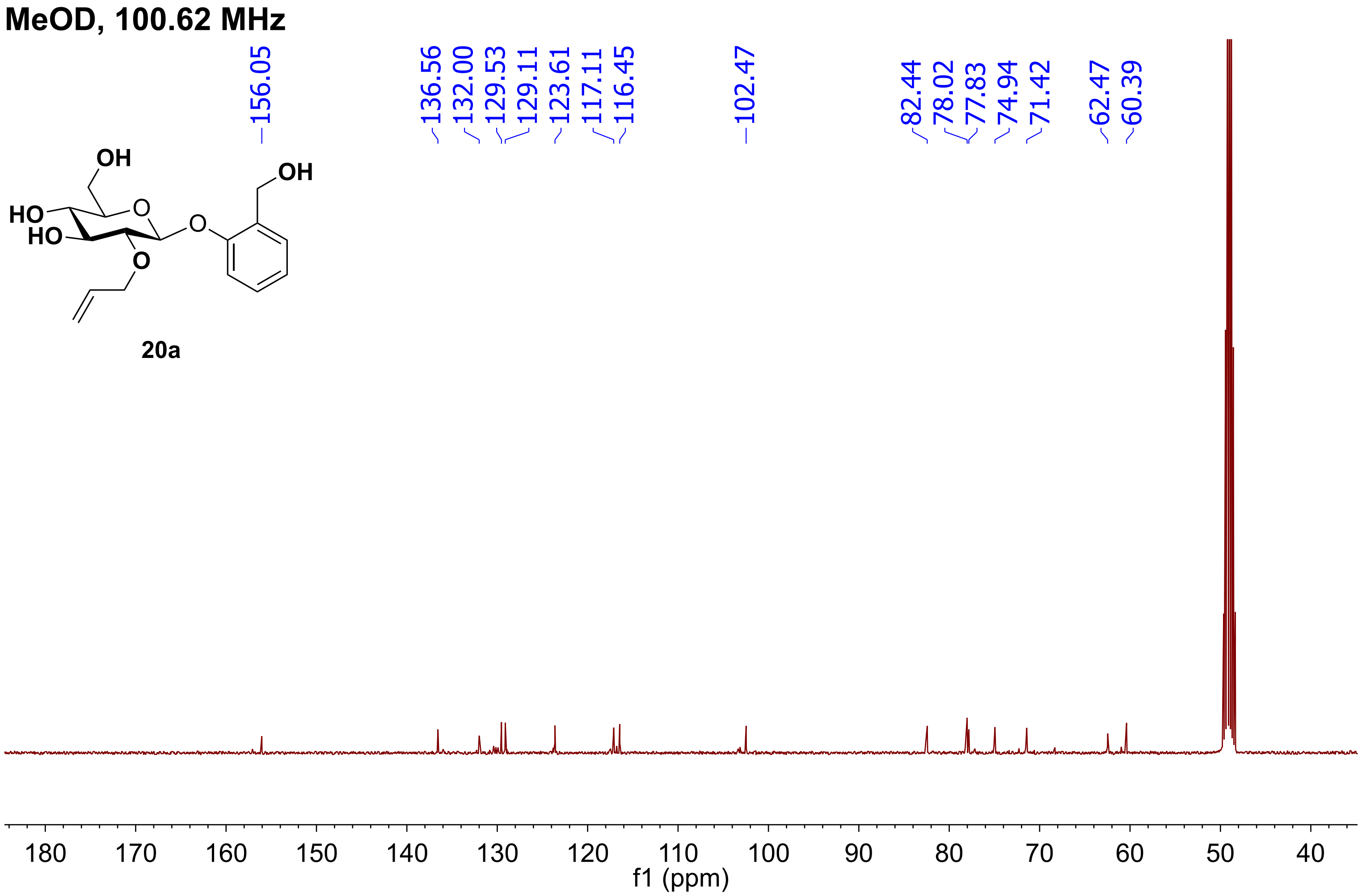
MeOD, 100.62 MHz
156.05
136.56
132.00
129.53
129.11
123.61
117.11
116.45
102.47
82.44
78.02
77.83
74.94
71.42
62.47
60.39
OH
HO
HO
O
O
O
OH
20a
180
170
160
150
140
130
120
110
100
90
80
70
60
50
40
f1 (ppm)

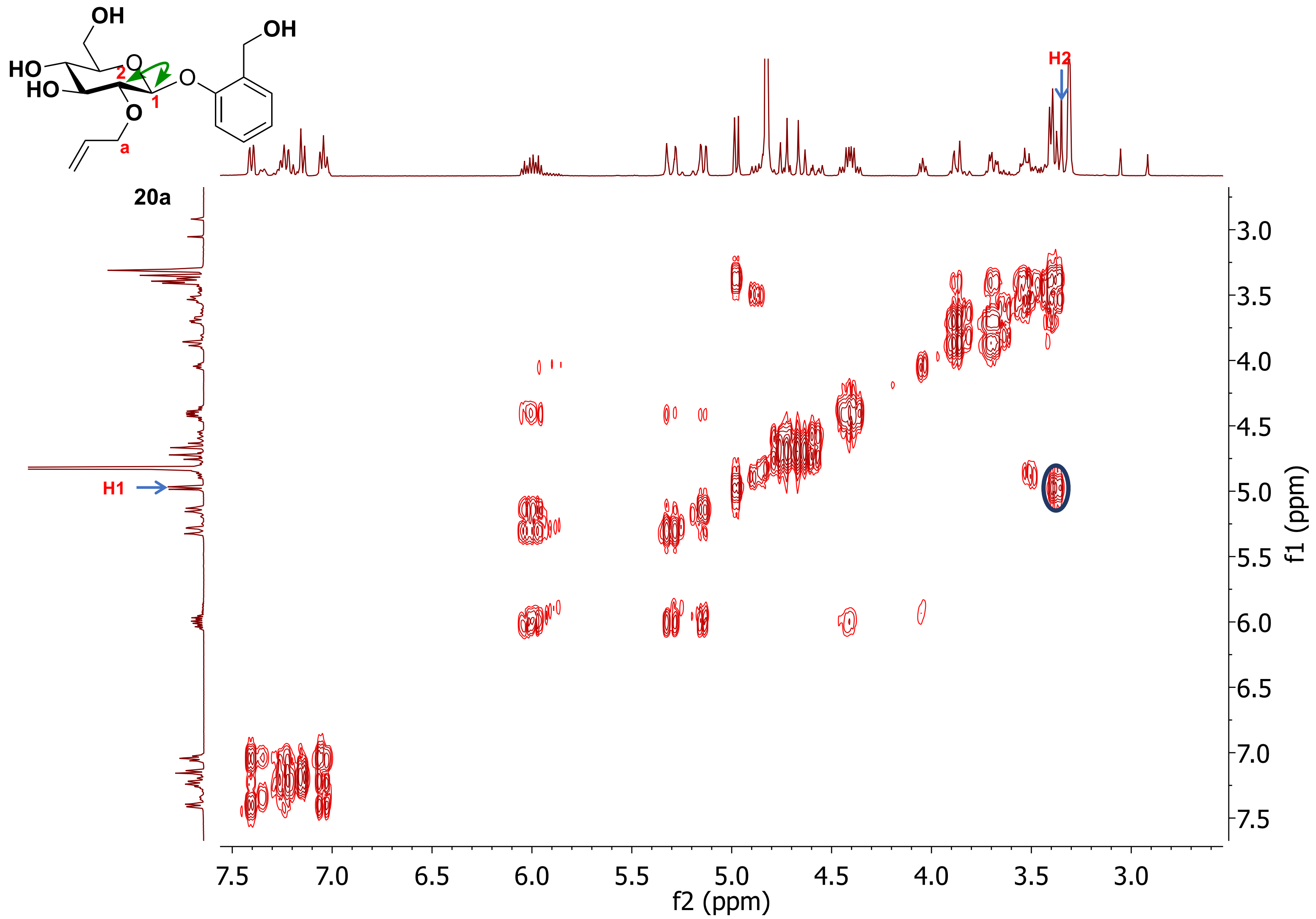
OH
OH
HO
HO
O
O
O
2
1
a
20a
H2
H1
7.5
7.0
6.5
6.0
5.5
5.0
4.5
4.0
3.5
3.0
f2 (ppm)
f1 (ppm)

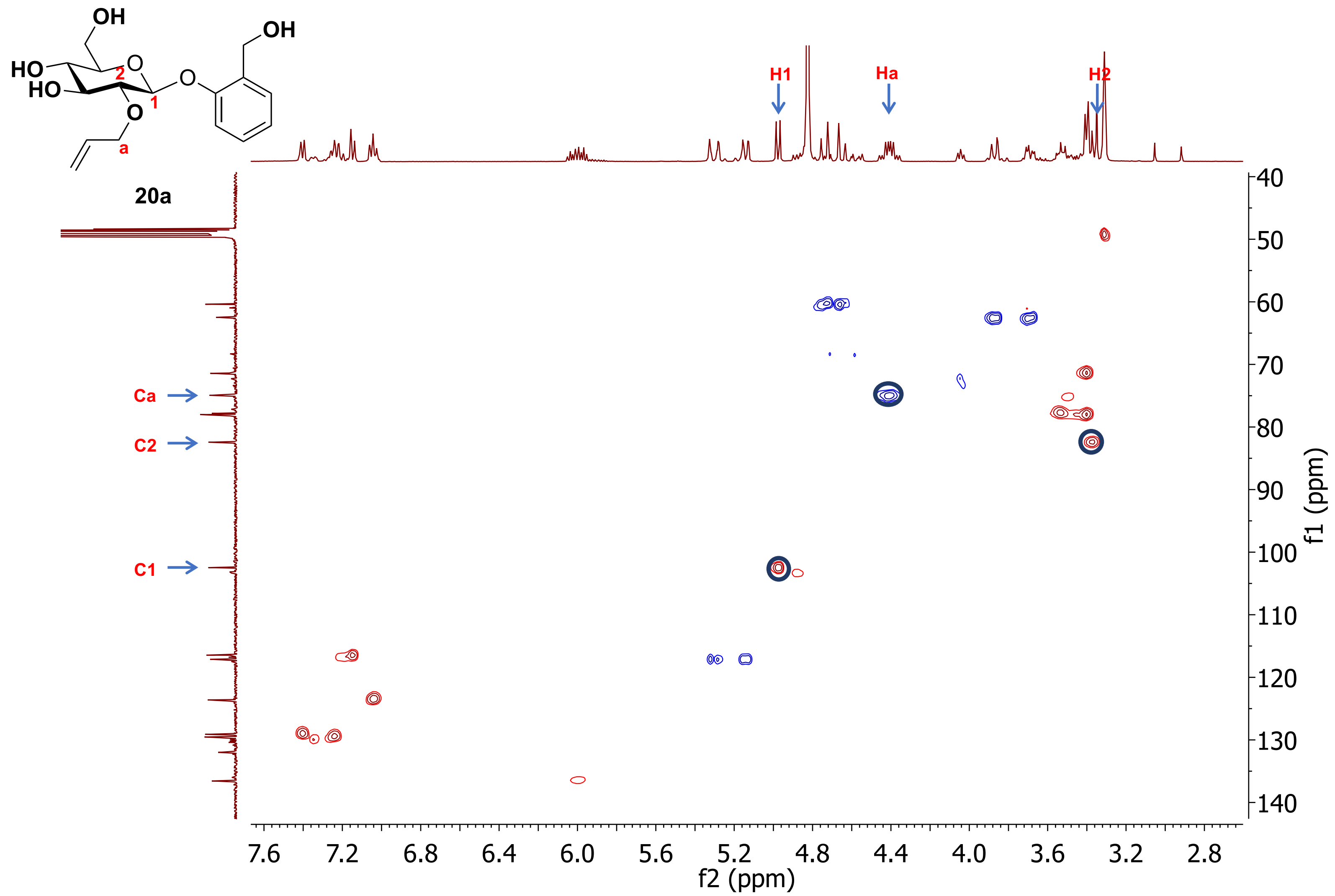
OH
OH
HO
HO
O
O
O
2
1
a
20a
H1
Ha
H2
Ca
C2
C1
f2 (ppm)
f1 (ppm)

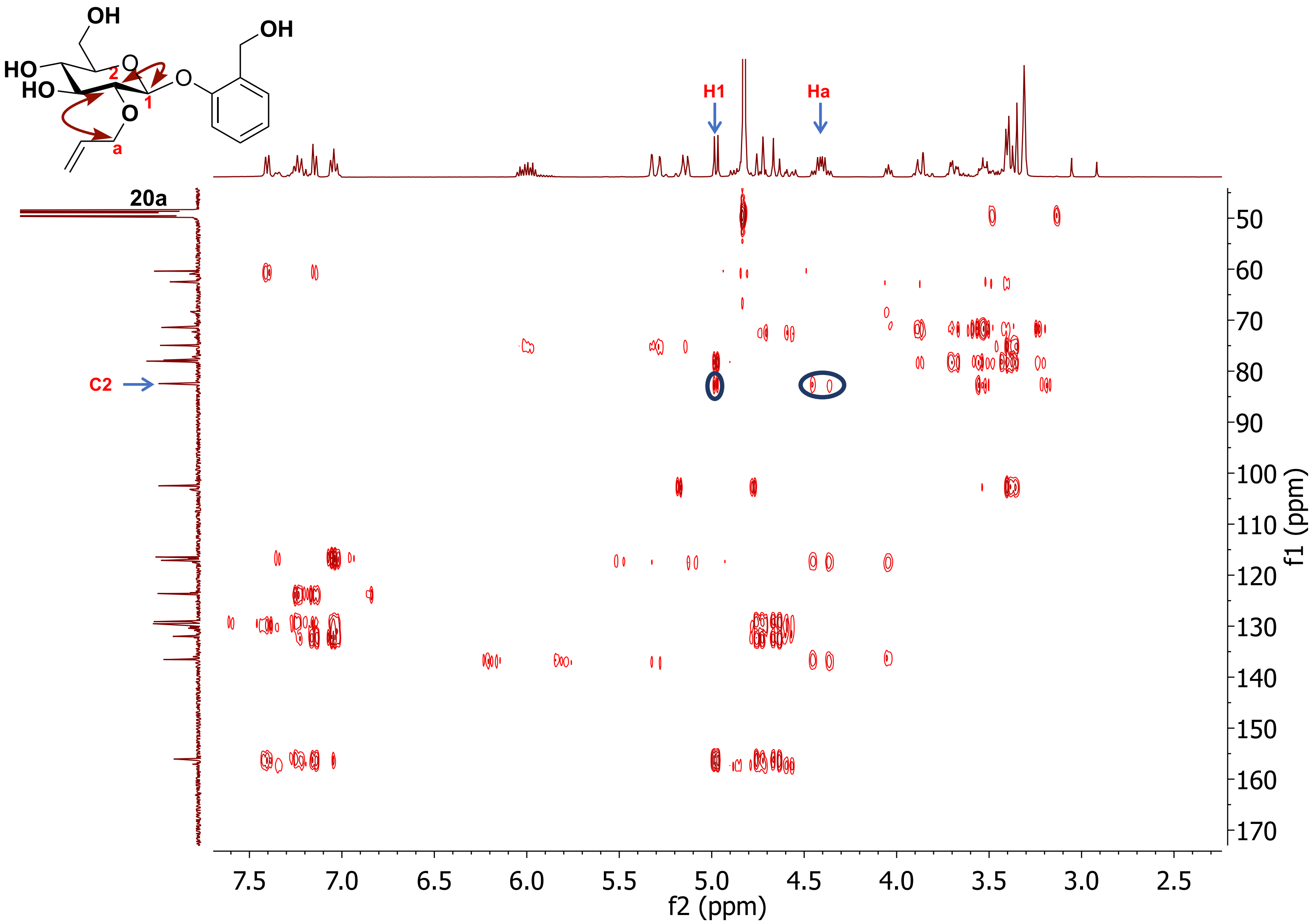
OH
OH
HO
HO
O
O
O
2
1
a
20a
H1
Ha
C2
f2 (ppm)
f1 (ppm)

CDCl3, 400.13 MHz

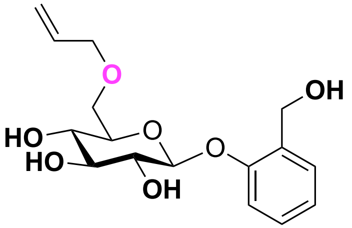

20b

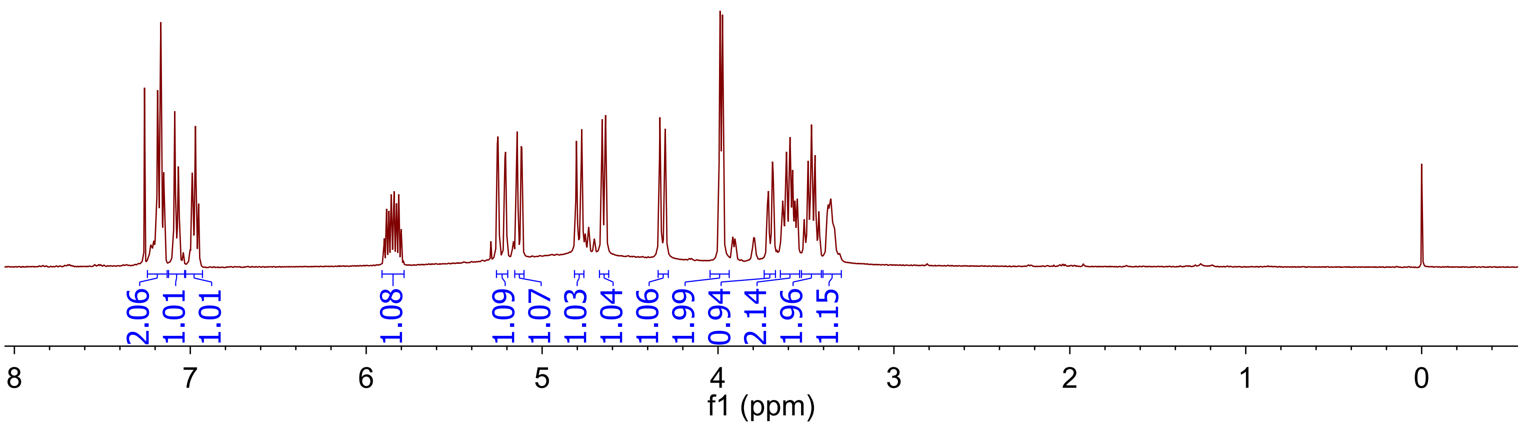

CDCl3, 100.62 MHz

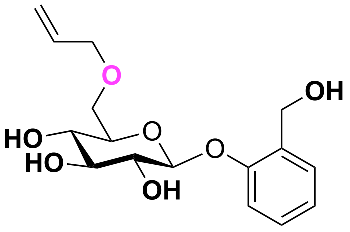

20b

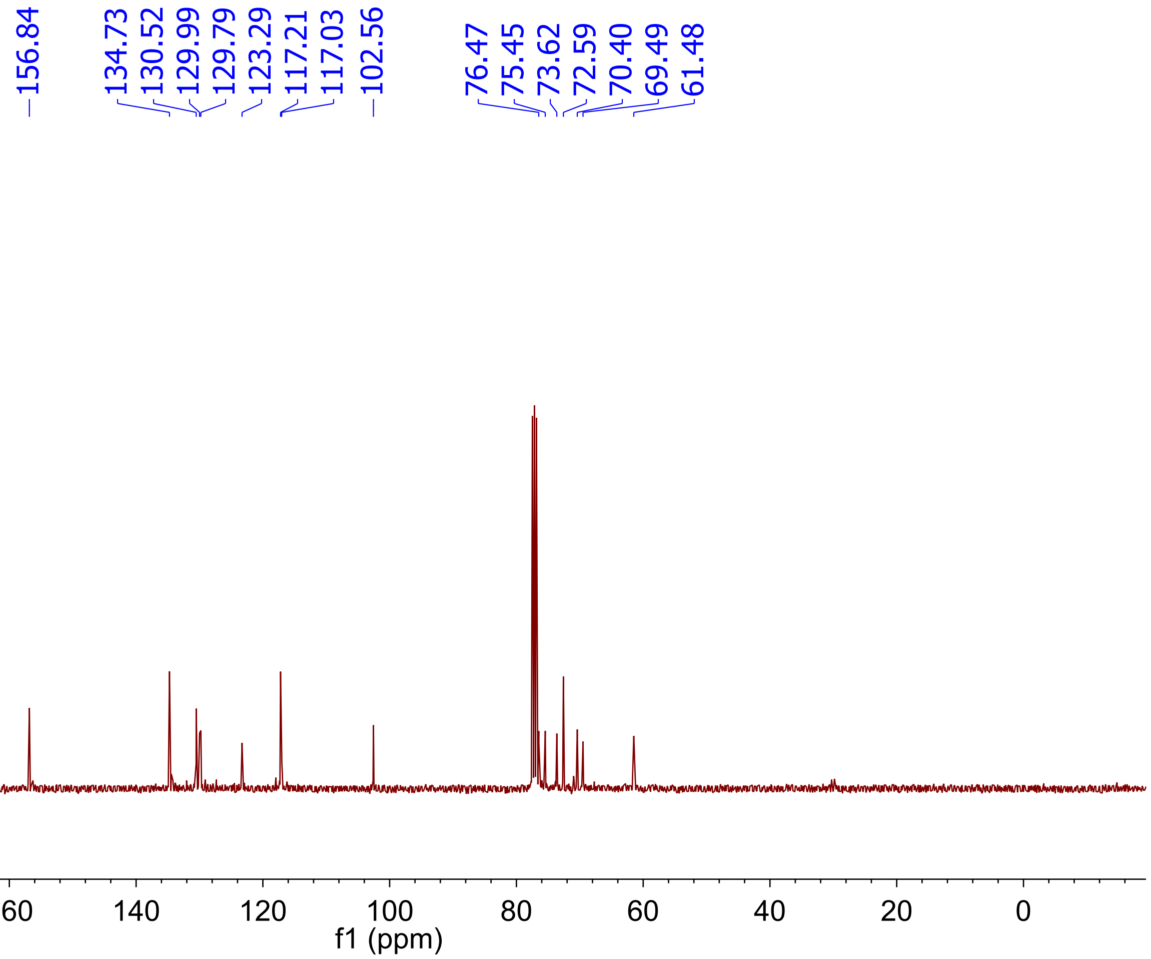

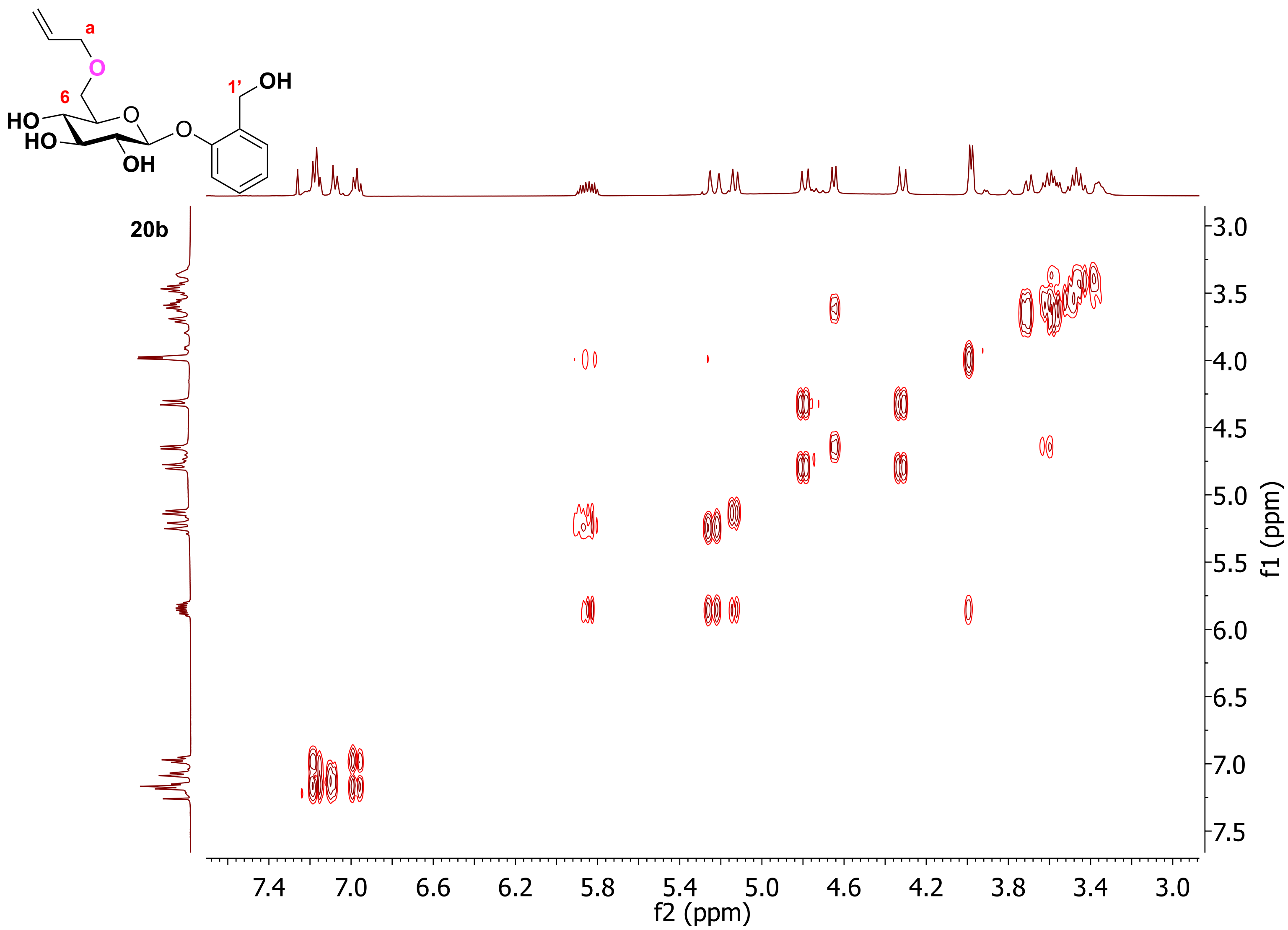
a
6
1'
OH
HO
HO
O
O
OH
20b
7.4
7.0
6.6
6.2
5.8
5.4
5.0
4.6
4.2
3.8
3.4
3.0
f2 (ppm)
3.0
3.5
4.0
4.5
5.0
5.5
6.0
6.5
7.0
7.5
f1 (ppm)

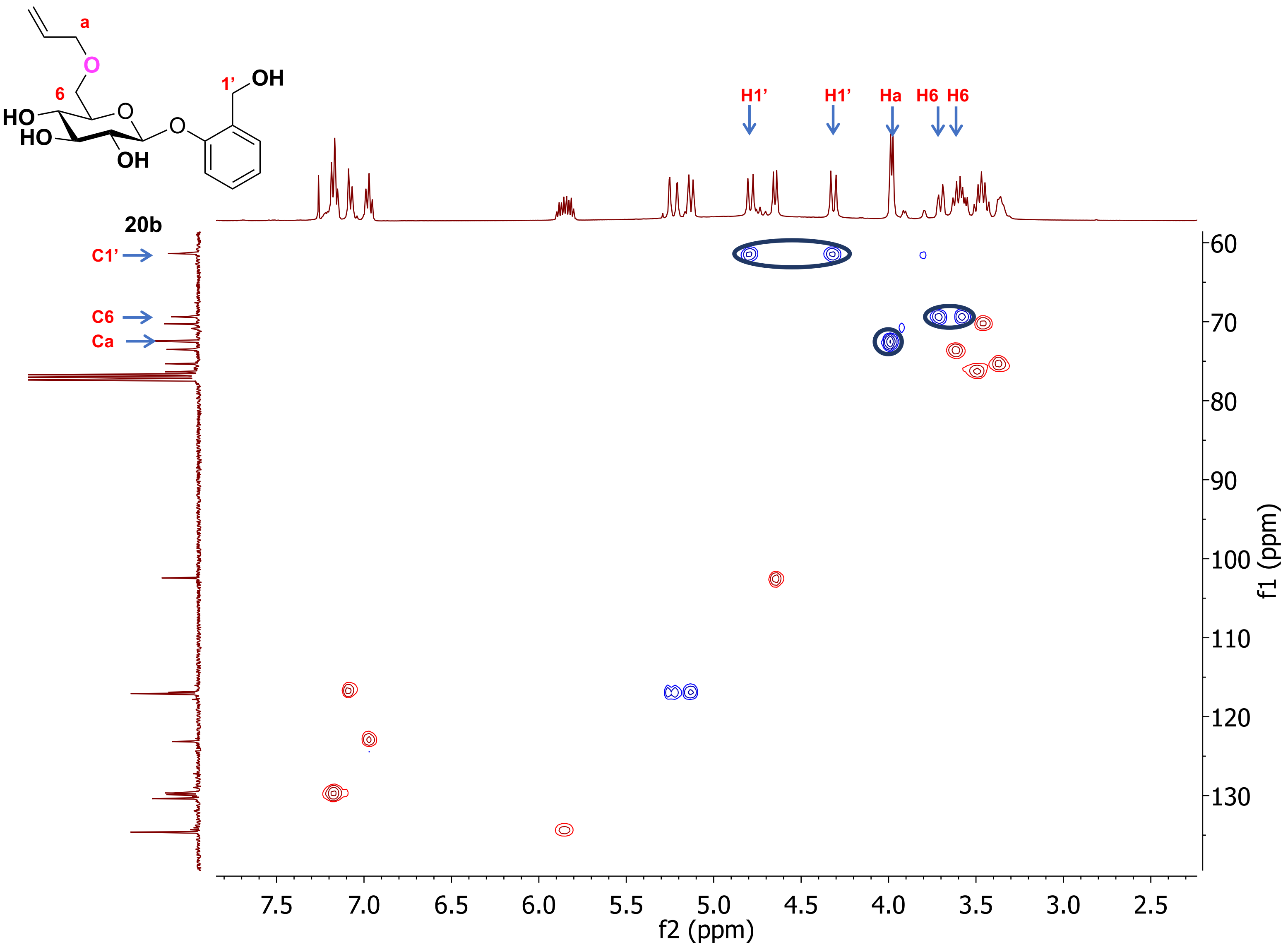
a
O
6
1'
OH
HO
HO
O
O
OH
20b
H1'
H1'
Ha
H6
H6
C1'
C6
Ca
60
70
80
90
100
110
120
130
f1 (ppm)
7.5
7.0
6.5
6.0
5.5
5.0
4.5
4.0
3.5
3.0
2.5
f2 (ppm)

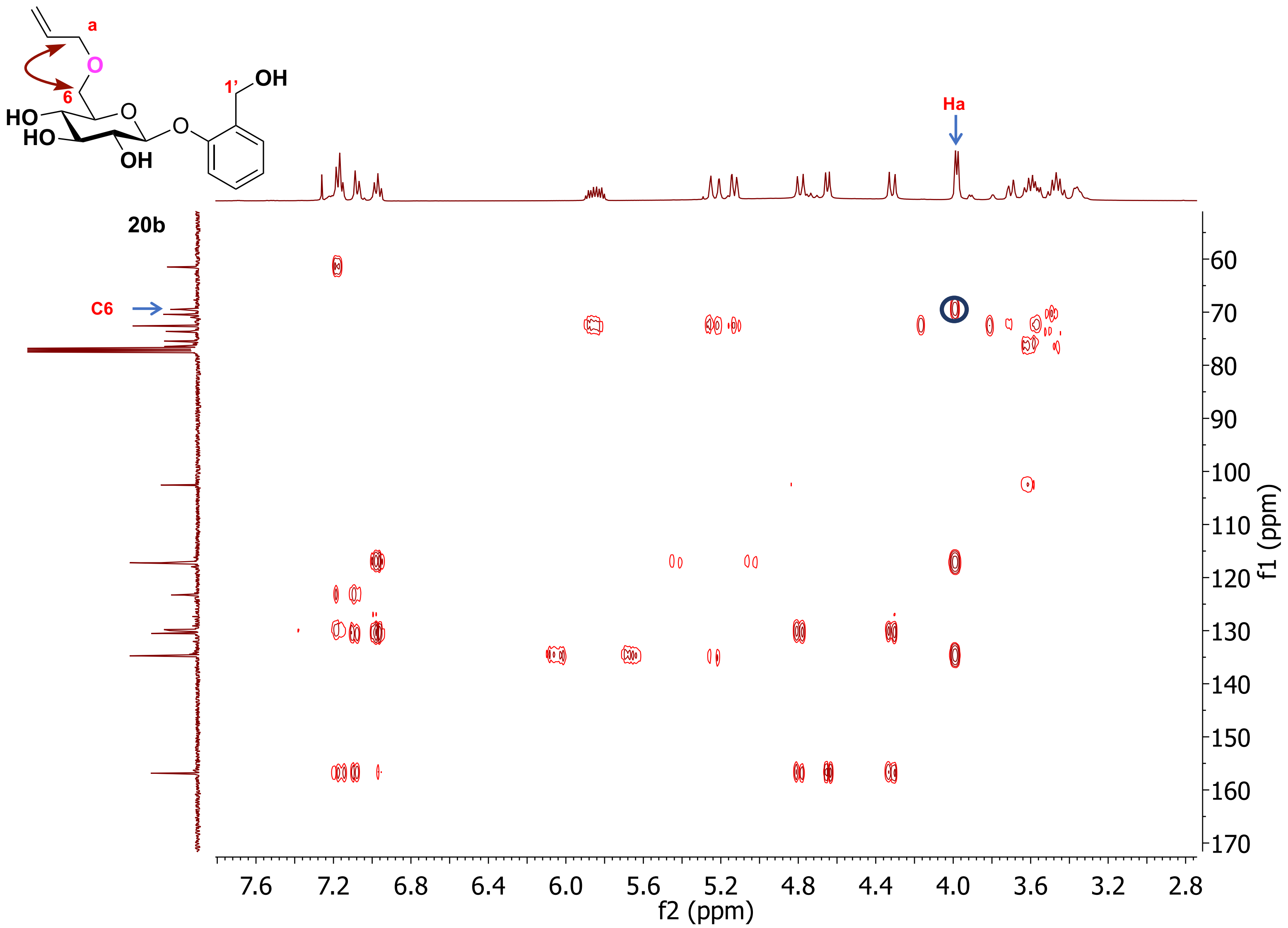

a
6
1'
OH
HO
HO
OH
O
O
Ha
20b
C6
f2 (ppm)
f1 (ppm)

MeOD, 400.13 MHz

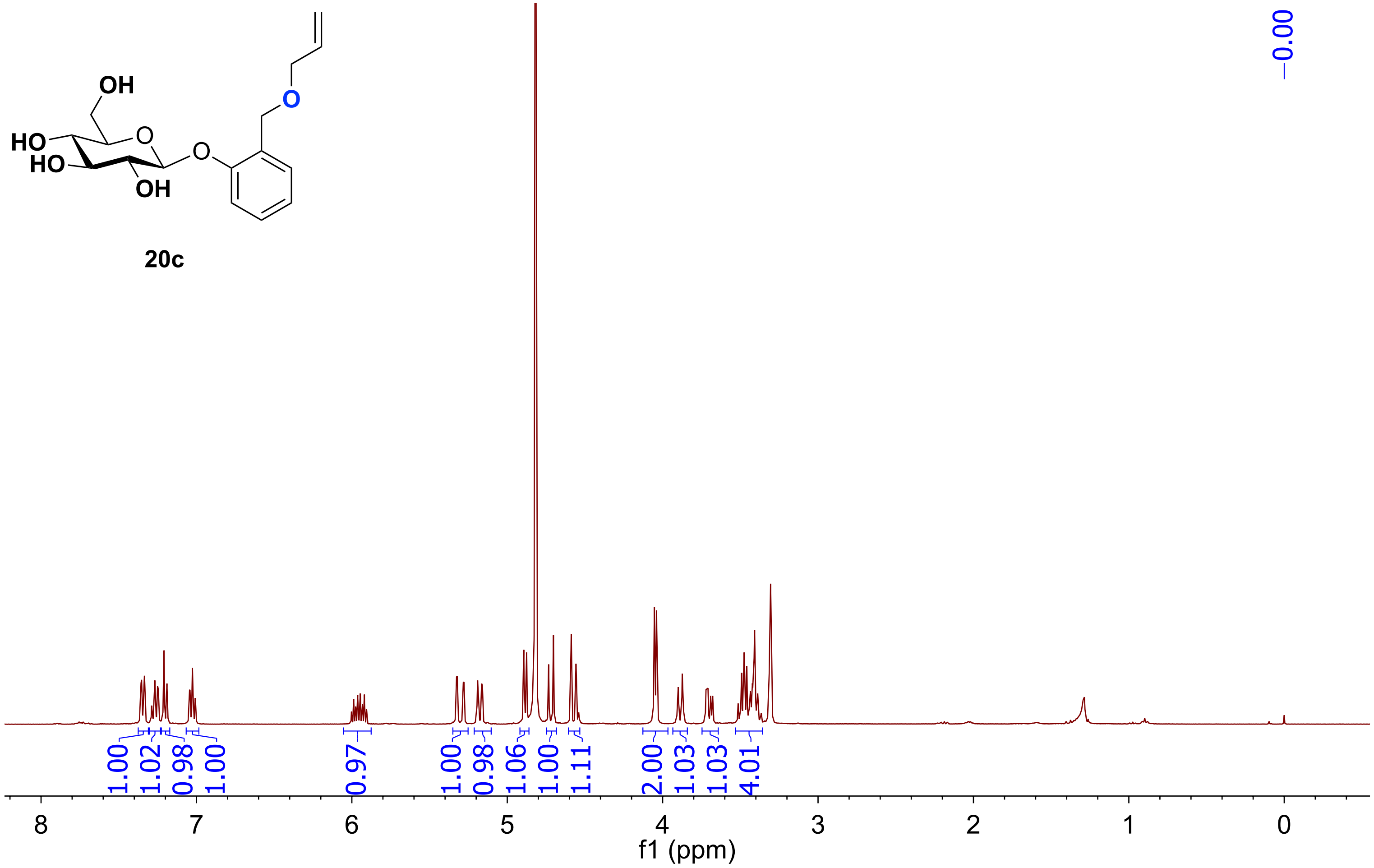

MeOD, 100.62 MHz

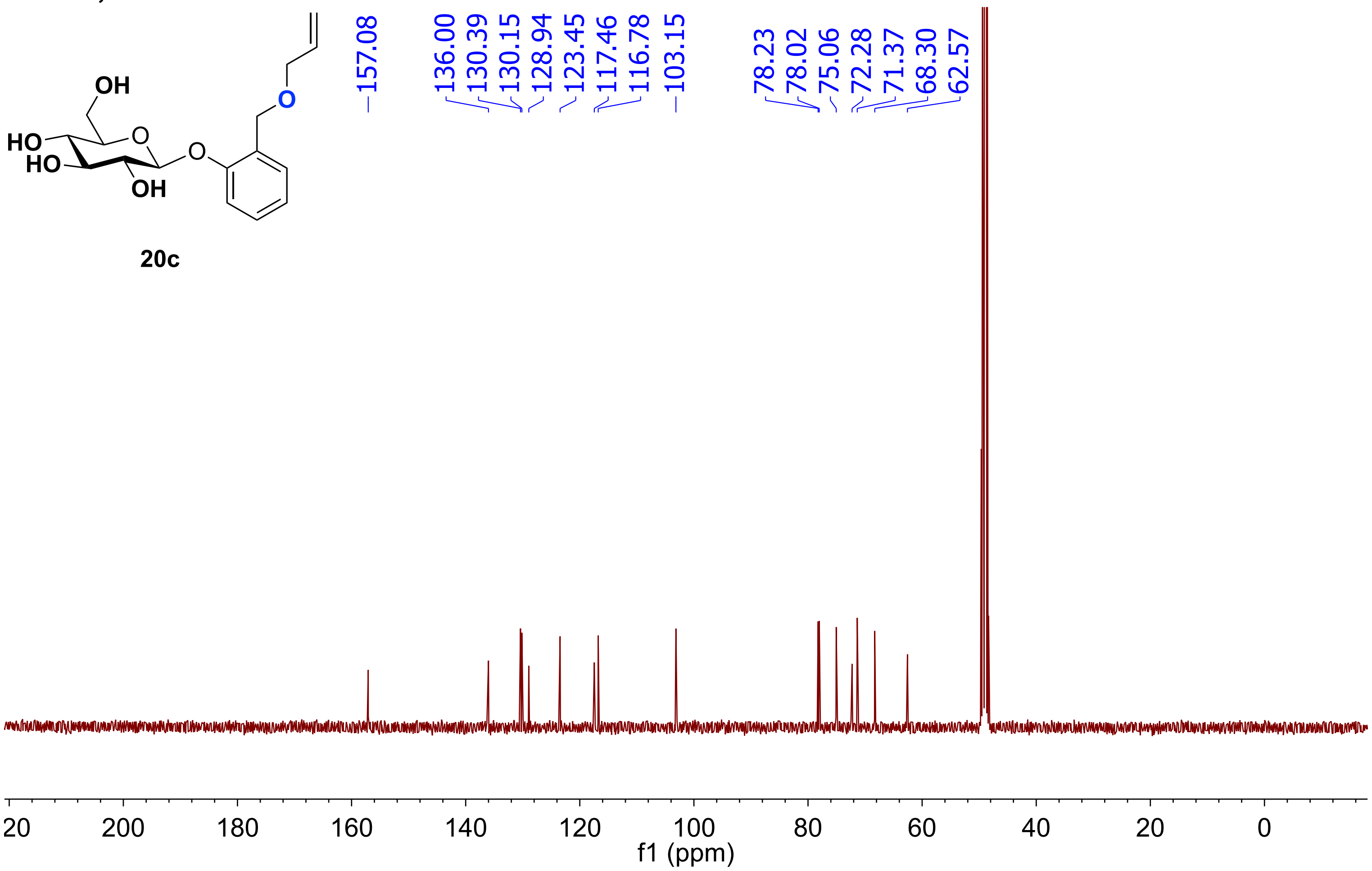

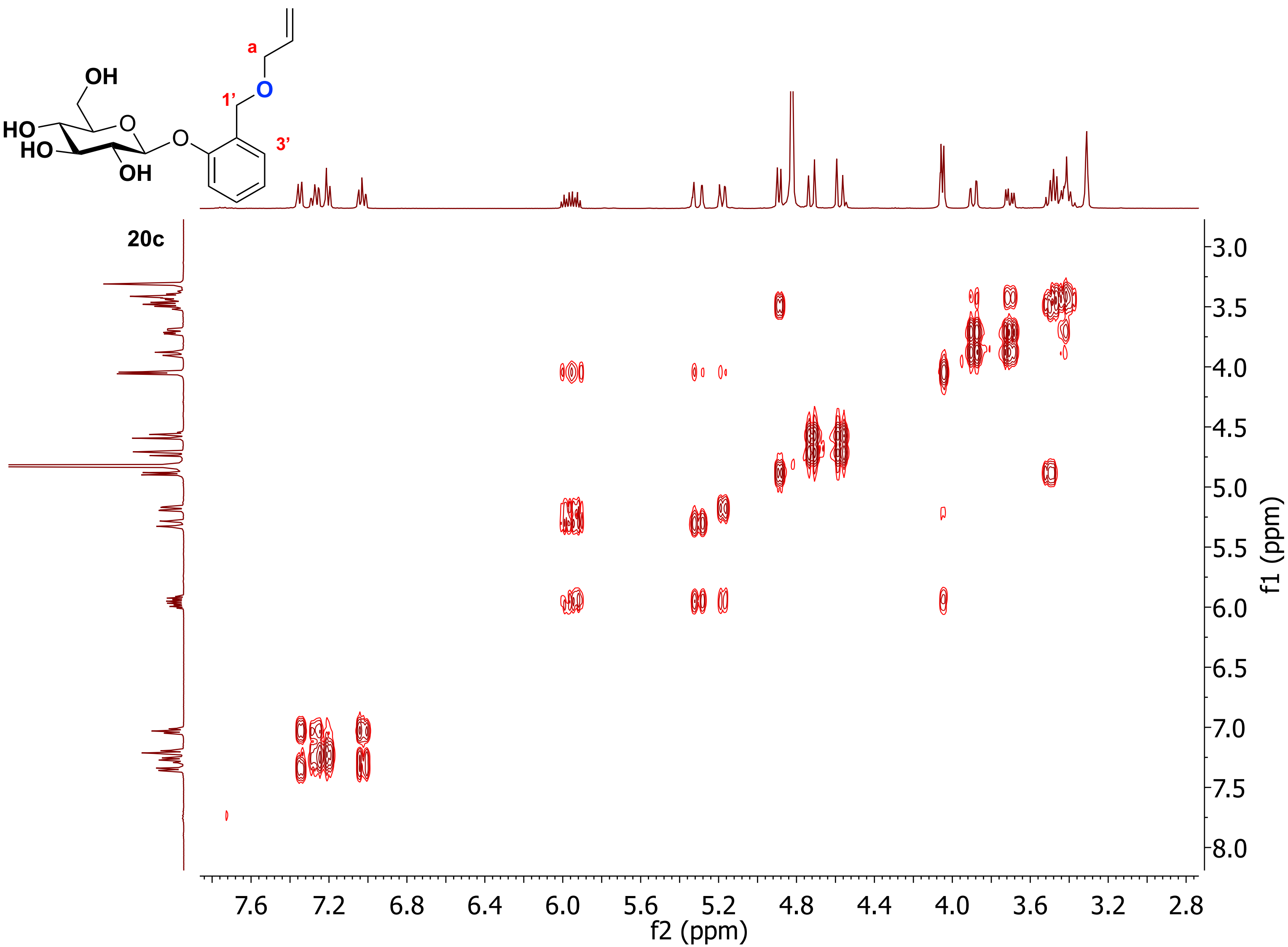
OH
HO
HO
O
O
OH
1'
O
a
3'
20c
f1 (ppm)
f2 (ppm)
3.0
3.5
4.0
4.5
5.0
5.5
6.0
6.5
7.0
7.5
8.0
7.6
7.2
6.8
6.4
6.0
5.6
5.2
4.8
4.4
4.0
3.6
3.2
2.8

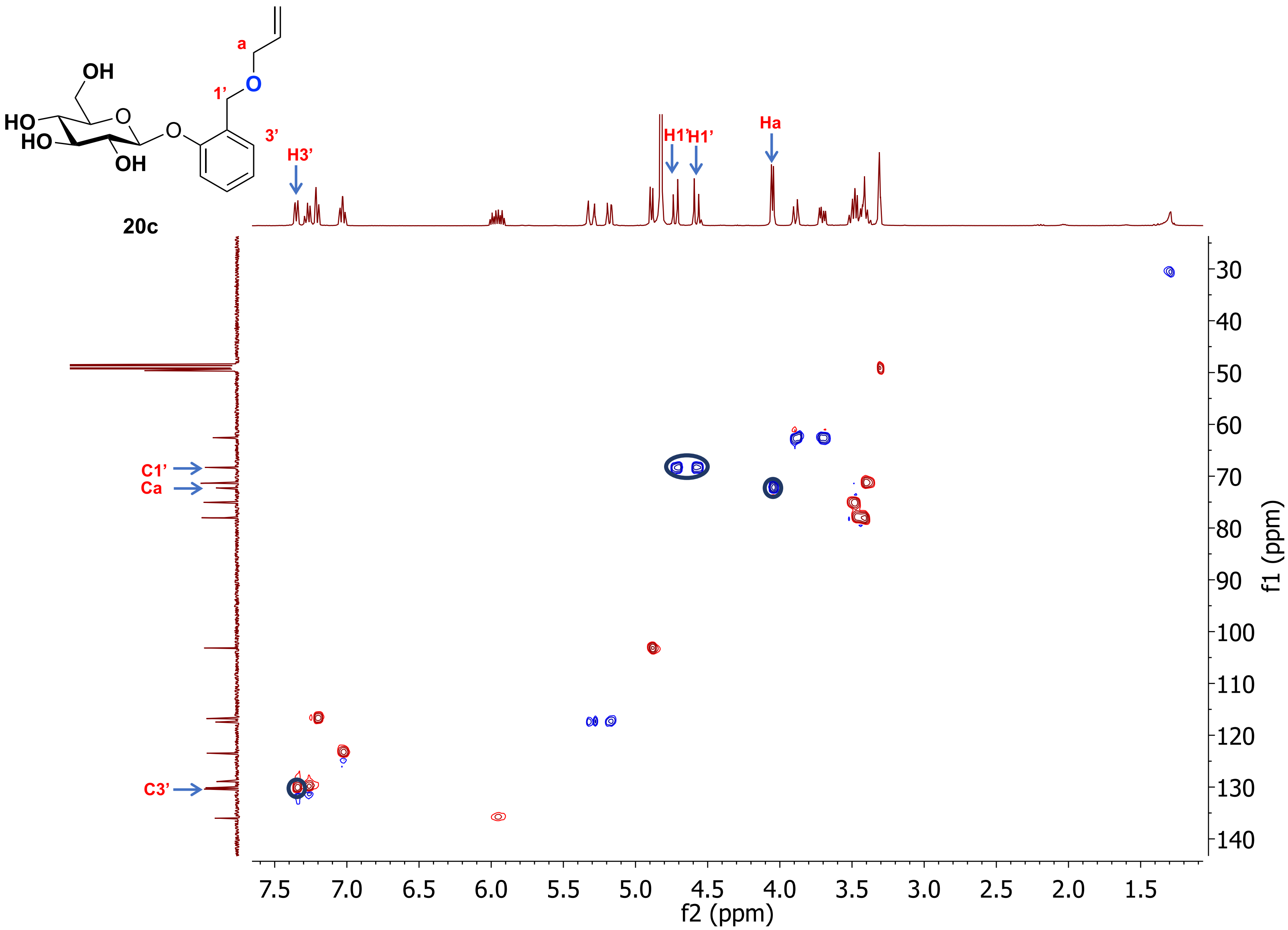

OH
HO
HO
OH
O
O
O
a
1'
3'
20c
H3'
H1'
H1'
Ha
C1'
Ca
C3'
7.5
7.0
6.5
6.0
5.5
5.0
4.5
4.0
3.5
3.0
2.5
2.0
1.5
f2 (ppm)
30
40
50
60
70
80
90
100
110
120
130
140
f1 (ppm)

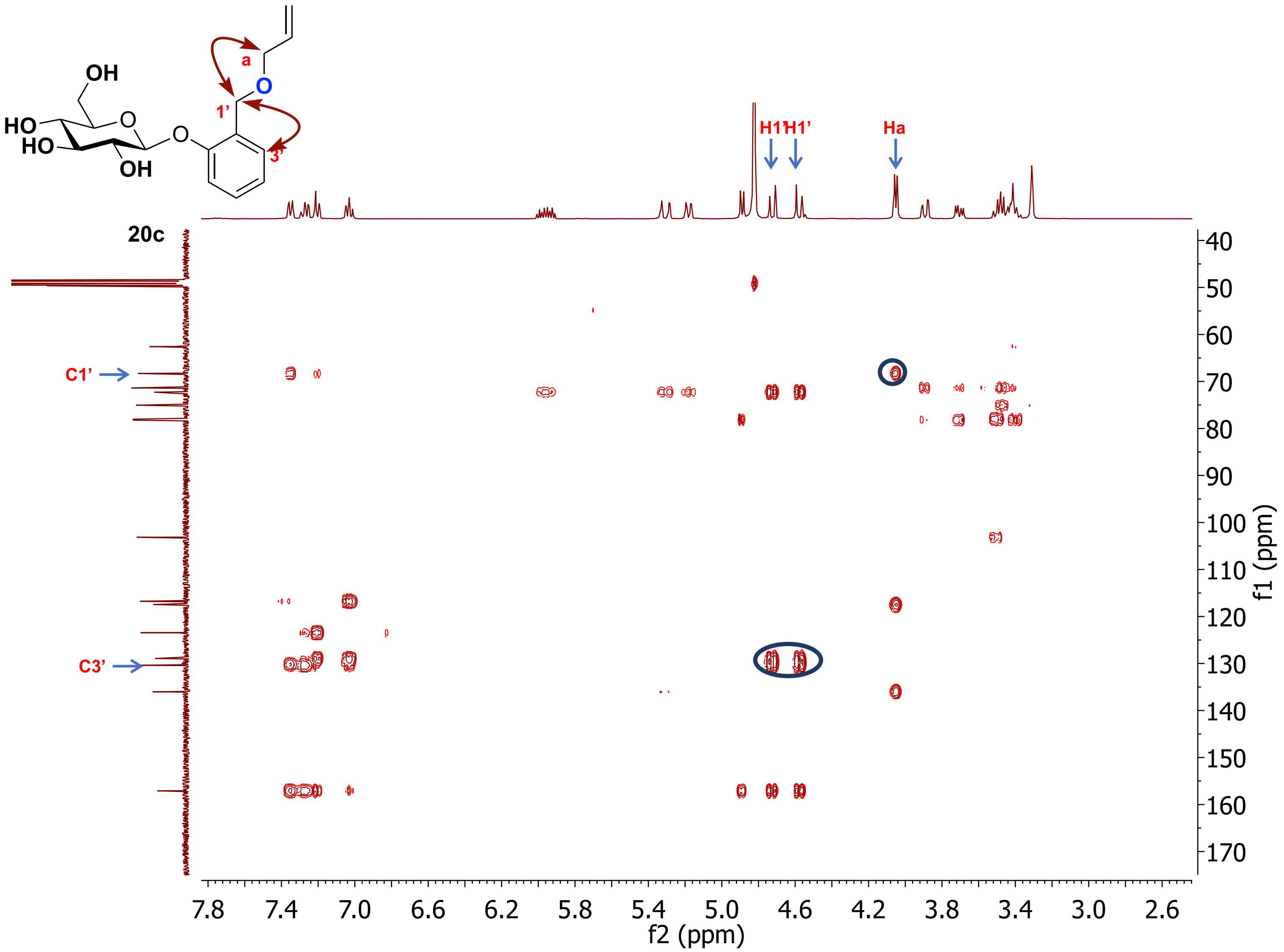

20c
a
1'
3'
C1'
C3'
H1'
H1'
Ha
f2 (ppm)
f1 (ppm)

MeOD, 400.13 MHz

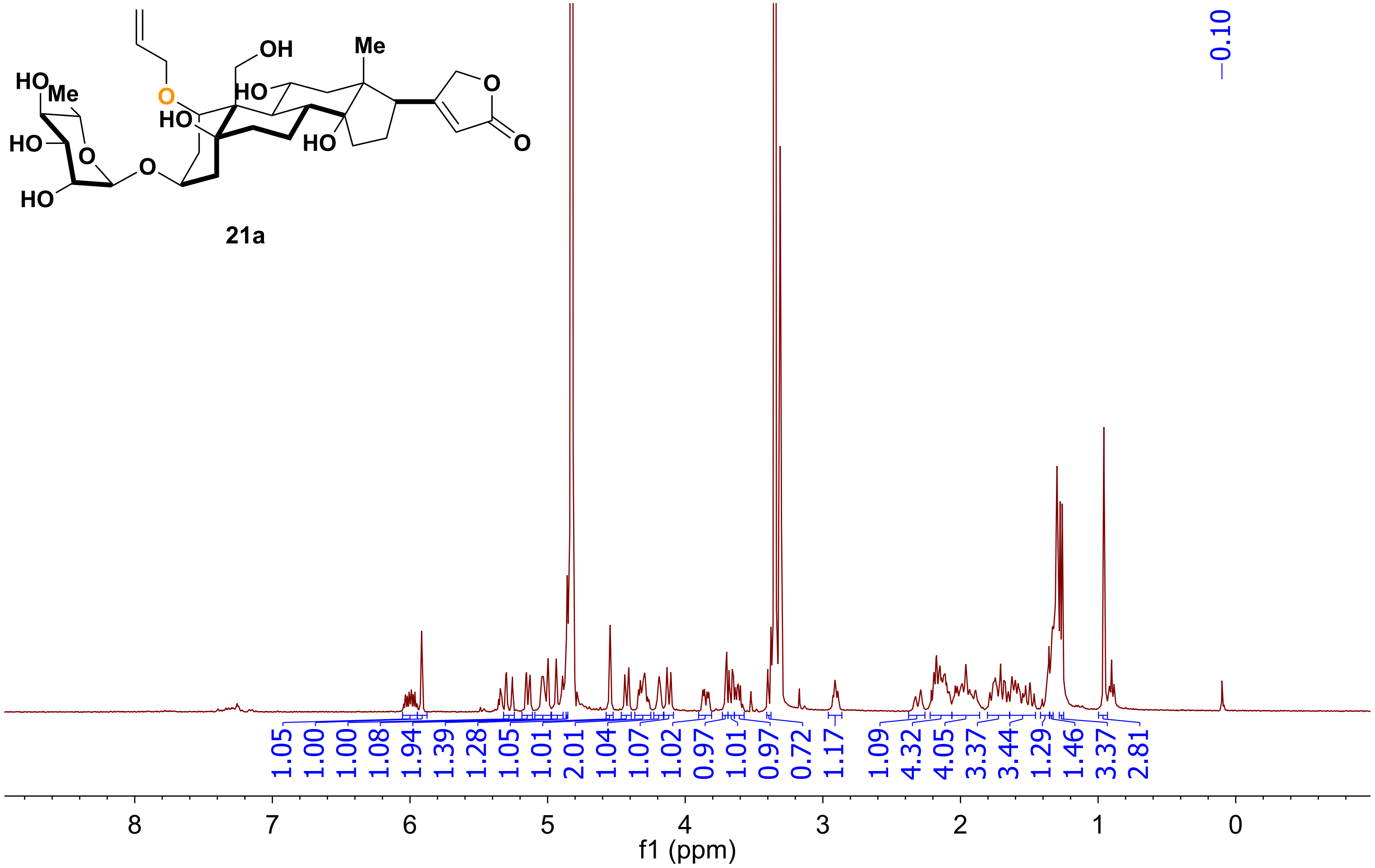

MeOD, 100.62 MHz

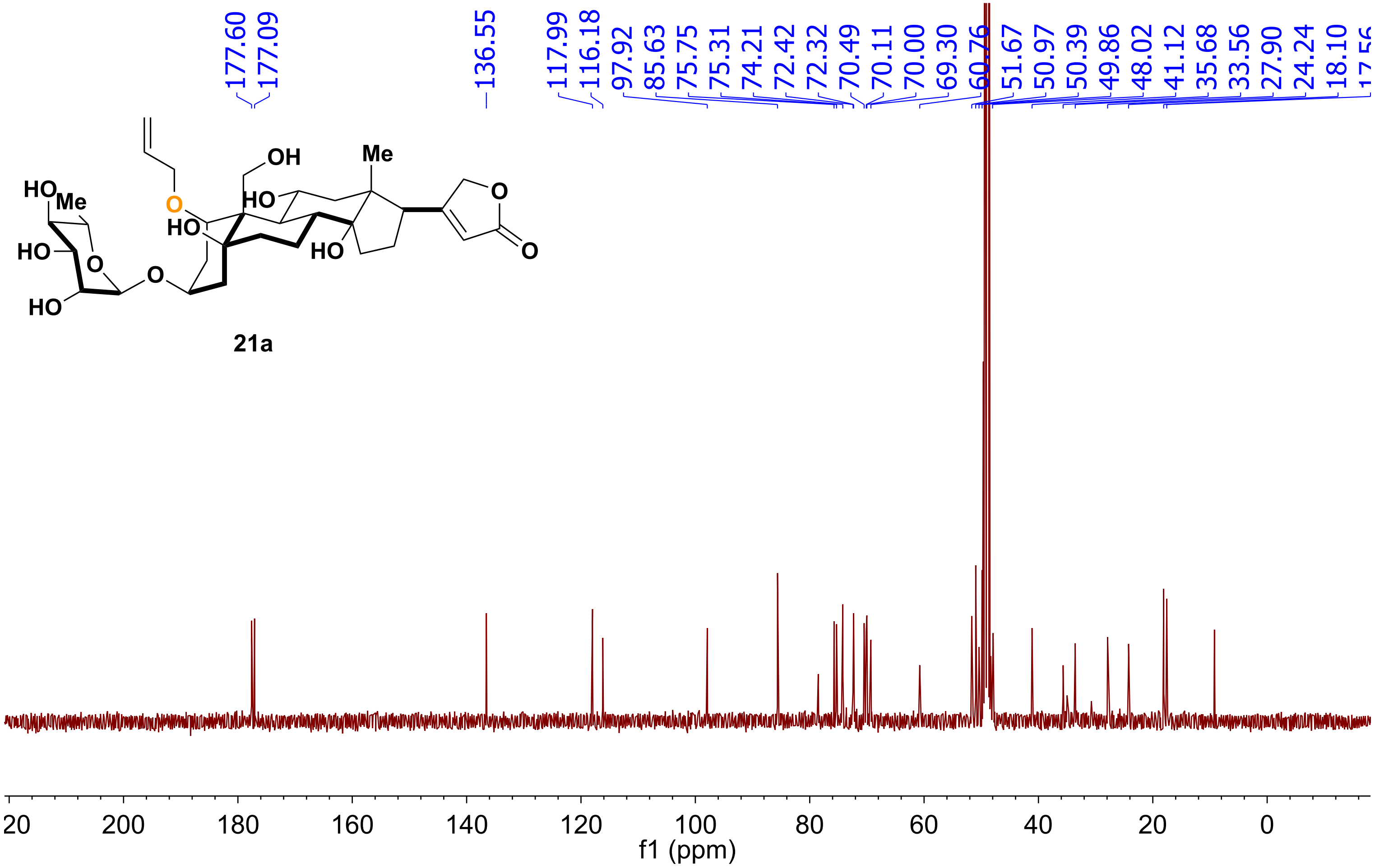

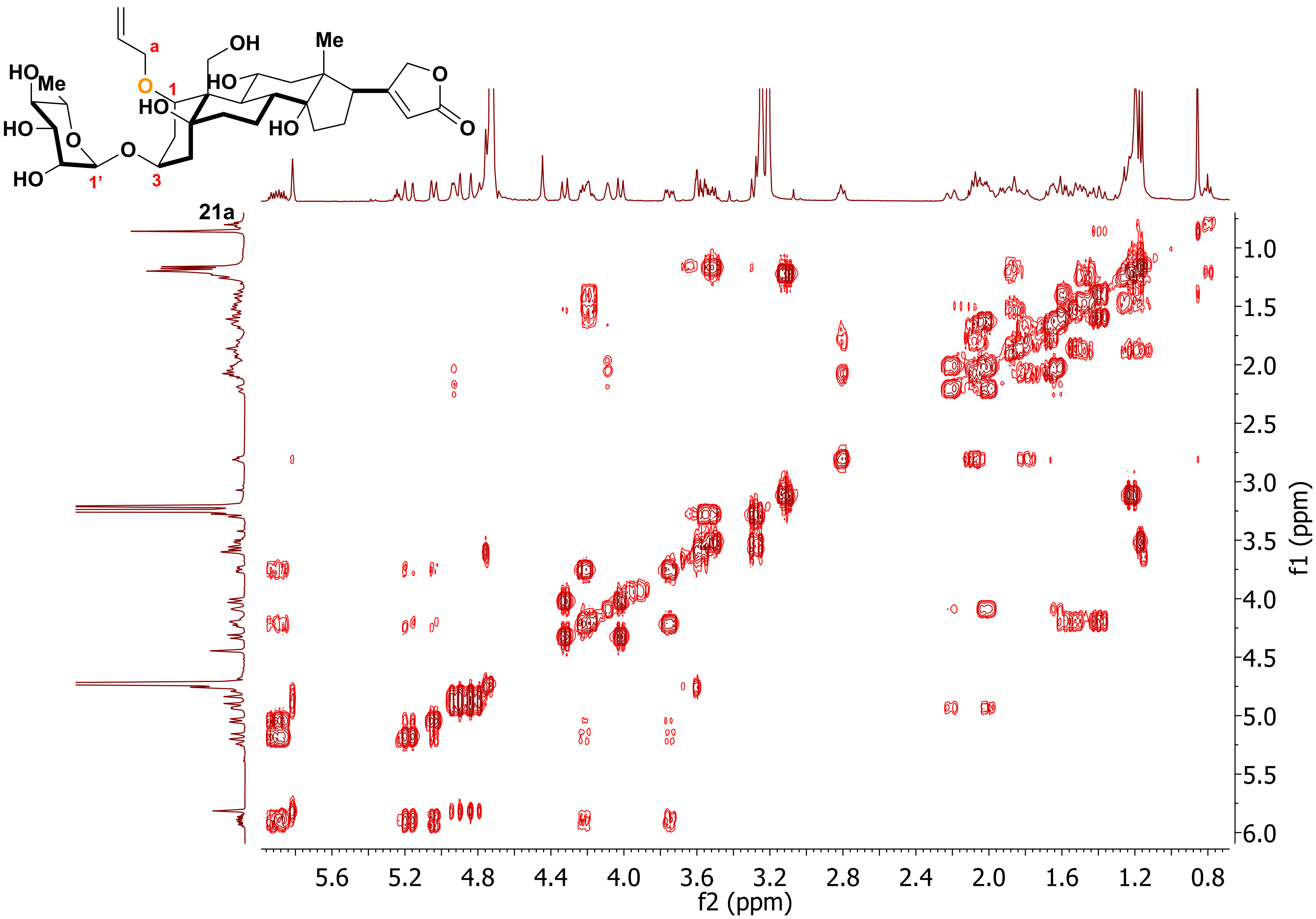

21a
a
1
3
1'
f2 (ppm)
f1 (ppm)

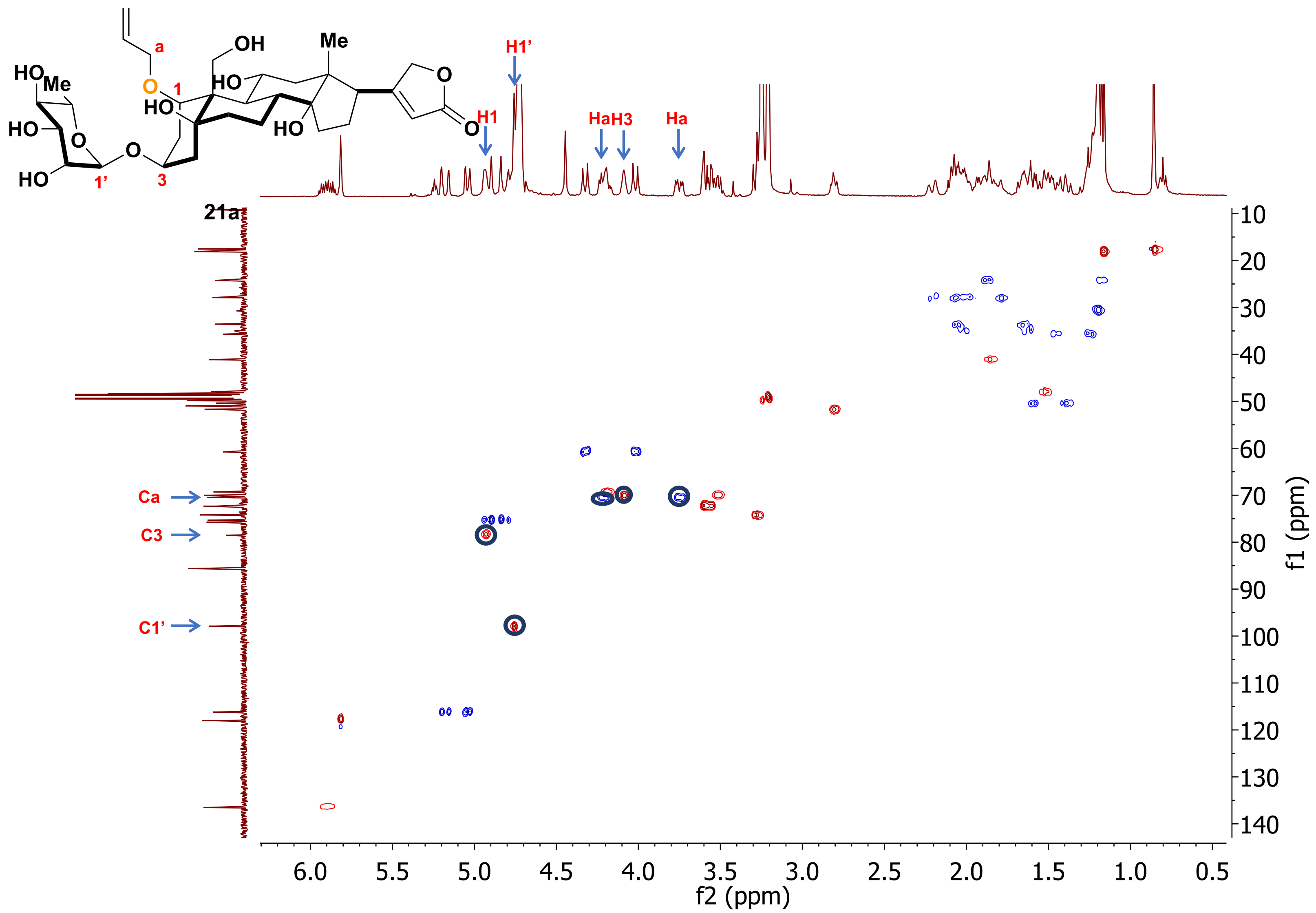

21a
H1'
H1
Ha
H3
Ha
Ca
C3
C1'
f2 (ppm)
f1 (ppm)

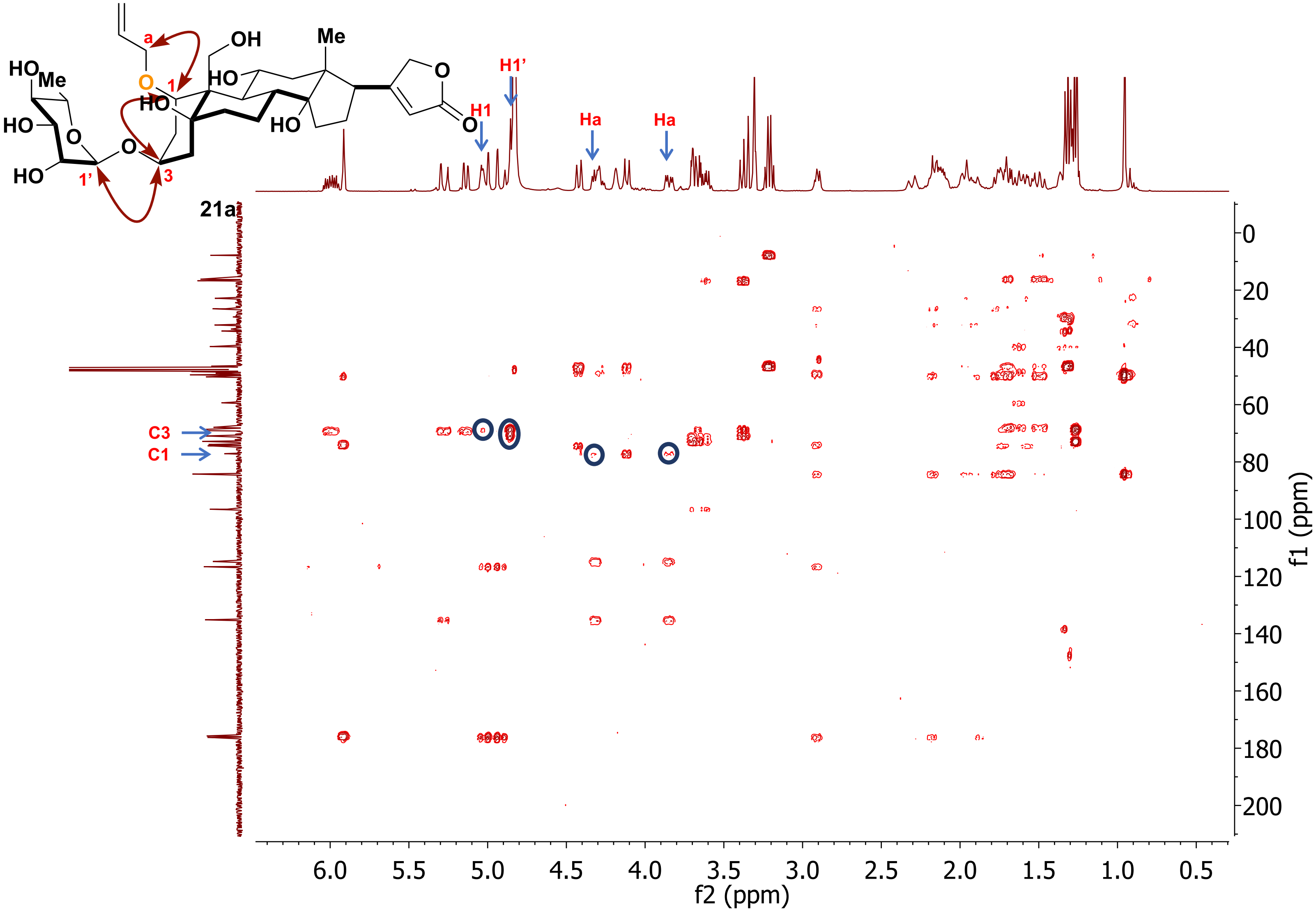
HO
Me
HO
HO
O
O
O
a
1
OH
HO
HO
Me
HO
O
O
3
1'
H1'
H1
Ha
Ha
21a
C3
C1
f2 (ppm)
f1 (ppm)

MeOD, 400.13 MHz

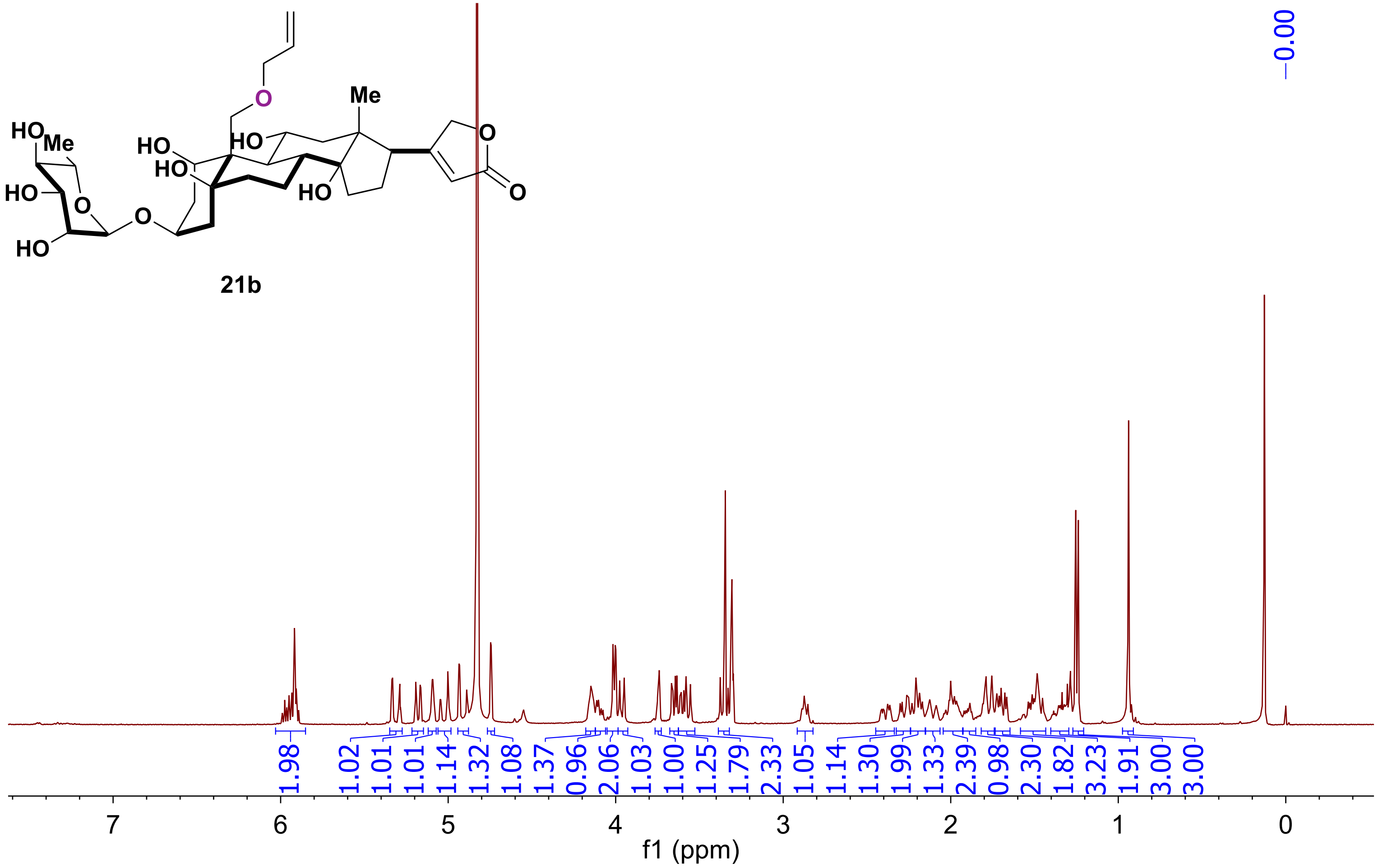

MeOD, 100.62 MHz

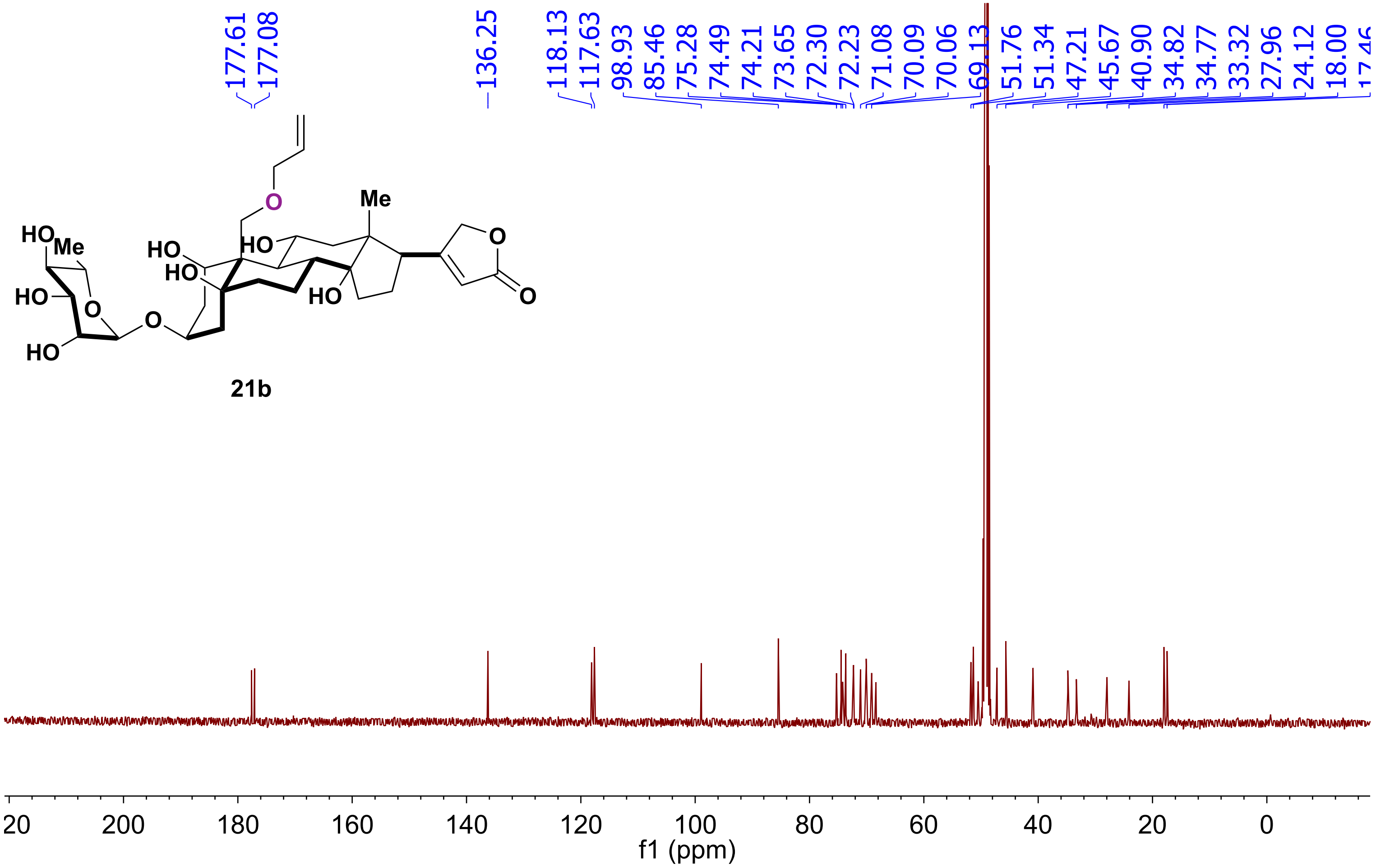

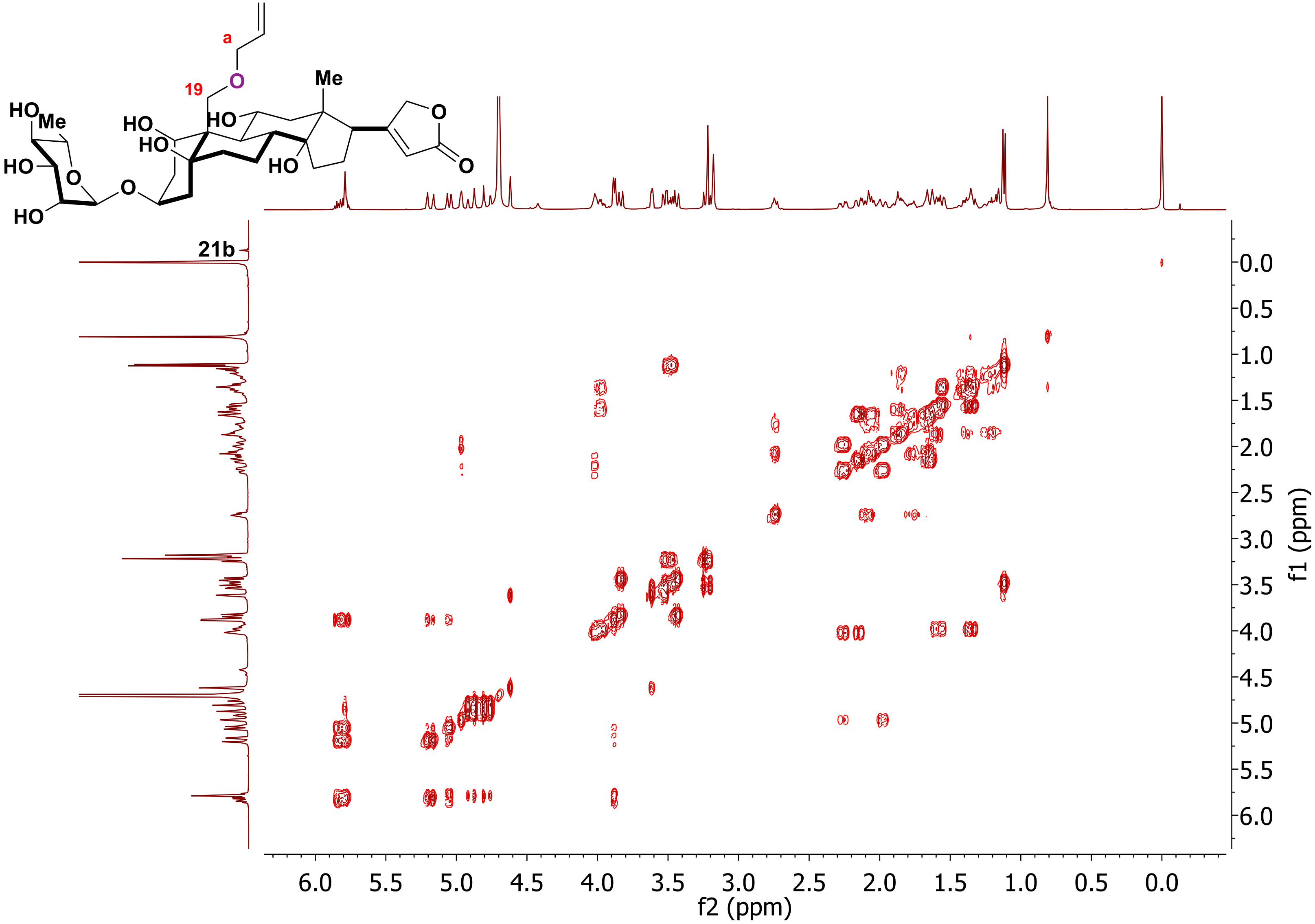
a
19
O
Me
HO
Me
HO
HO
HO
O
O
HO
HO
O
O
HO
21b
0.0
0.5
1.0
1.5
2.0
2.5
3.0
3.5
4.0
4.5
5.0
5.5
6.0
f1 (ppm)
6.0
5.5
5.0
4.5
4.0
3.5
3.0
2.5
2.0
1.5
1.0
0.5
0.0
f2 (ppm)

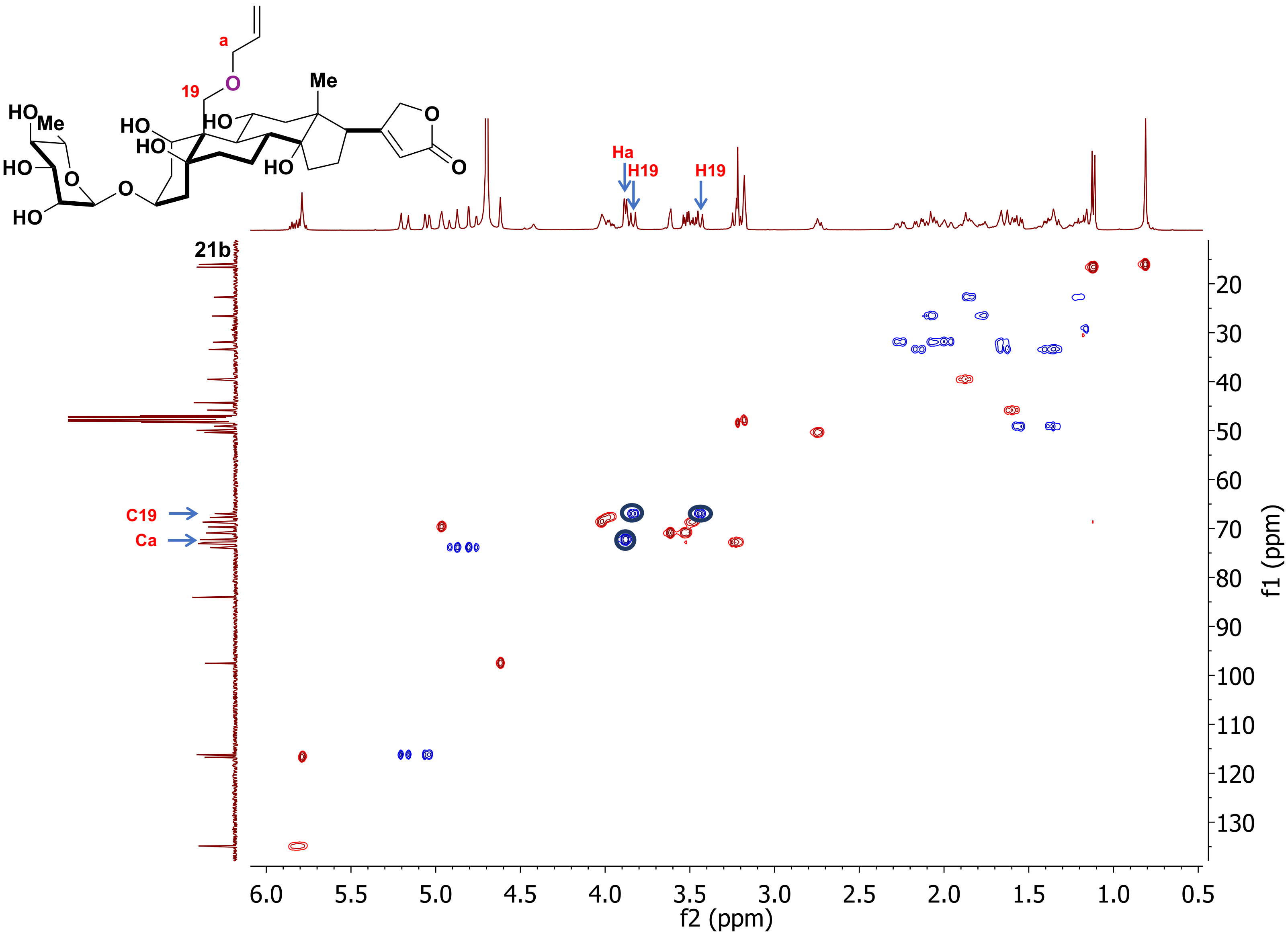

21b
HO
Me
HO
HO
HO
O
O
HO
HO
19
O
a
Me
HO
O
O
Ha
H19
H19
C19
Ca
f2 (ppm)
f1 (ppm)

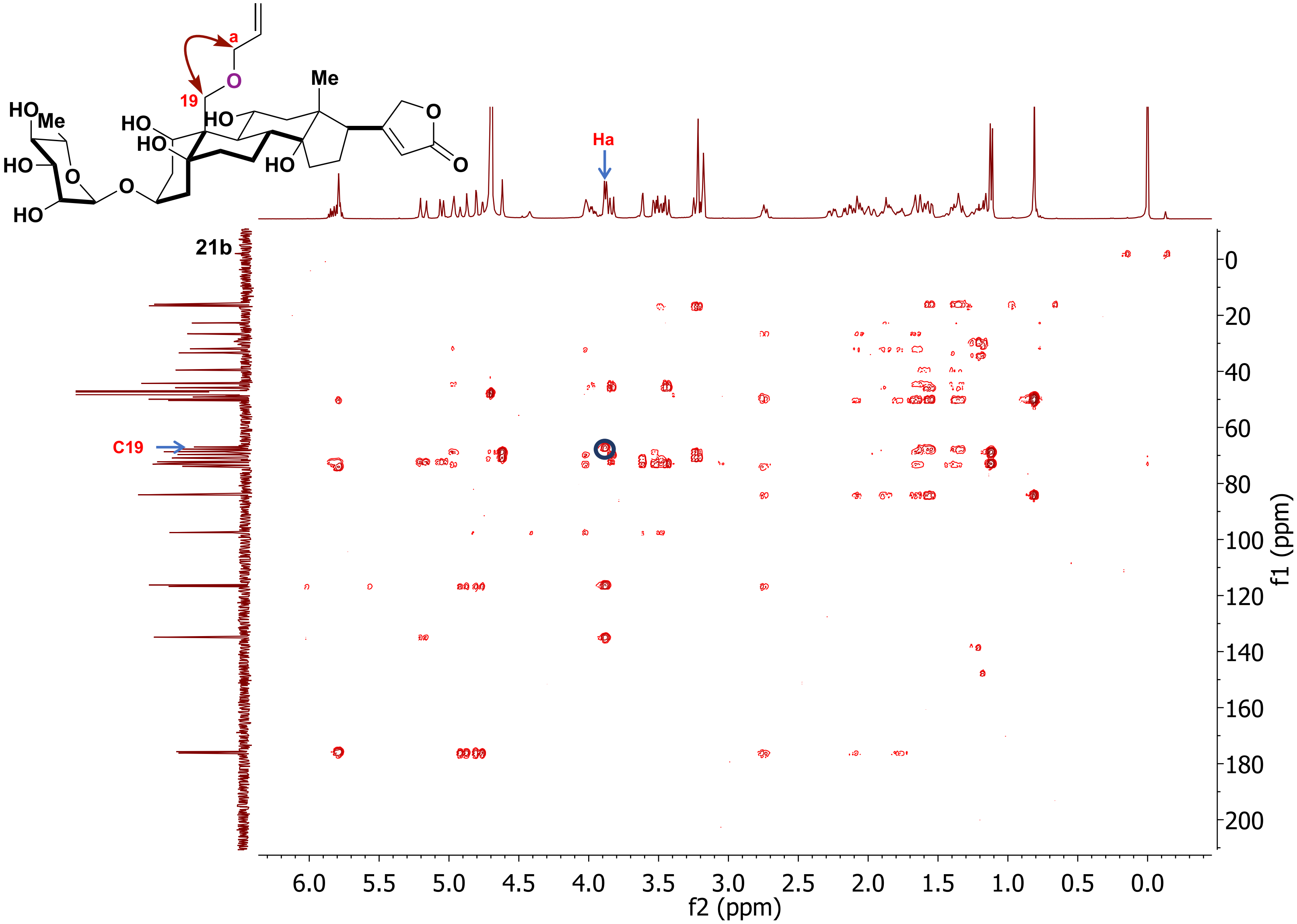

HO
Me
HO
O
O
HO
HO
HO
19
O
a
HO
Me
O
O
HO
Ha
21b
C19
0
20
40
60
80
100
120
140
160
180
200
f1 (ppm)
6.0
5.5
5.0
4.5
4.0
3.5
3.0
2.5
2.0
1.5
1.0
0.5
0.0
f2 (ppm)

MeOD, 400.13 MHz

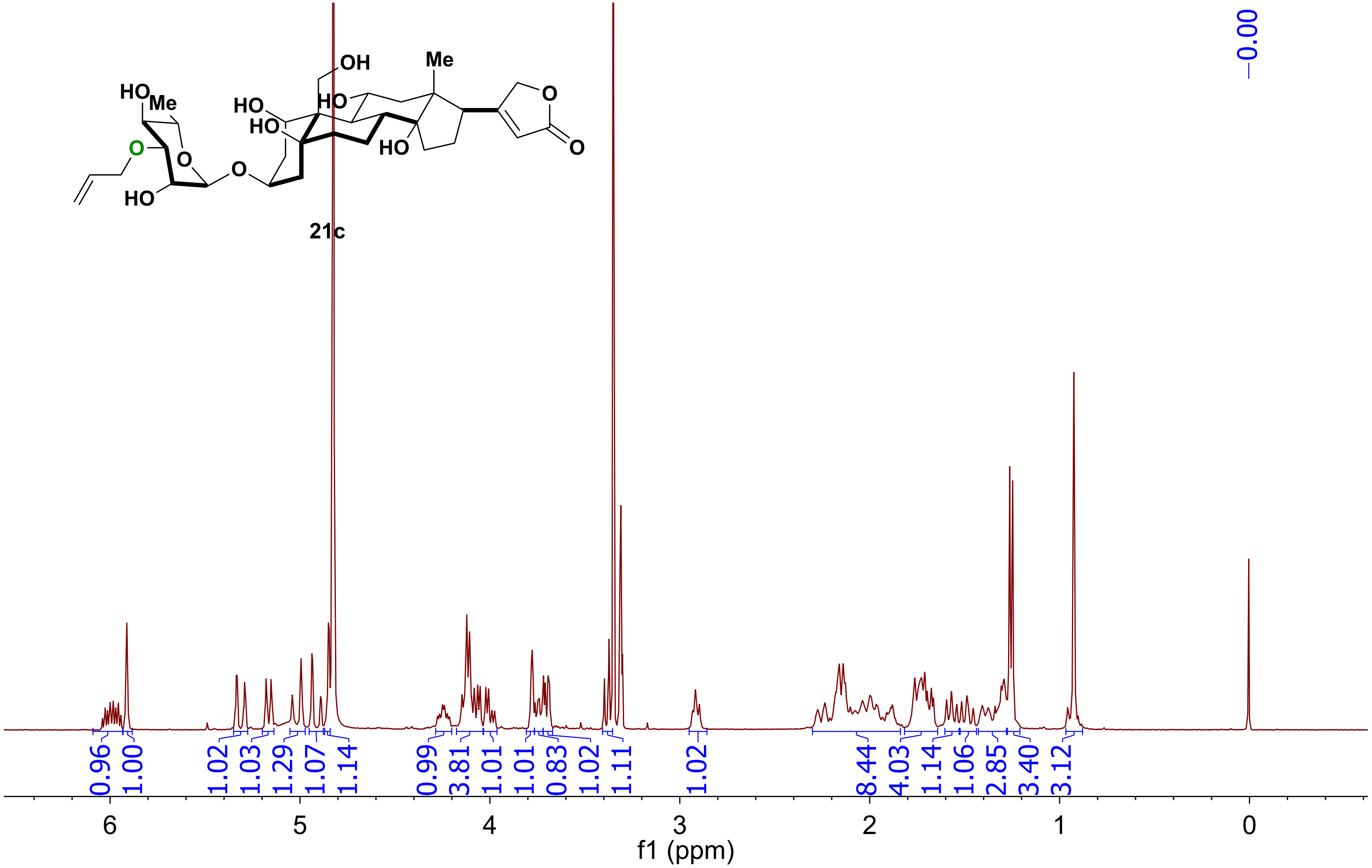

MeOD, 100.62 MHz

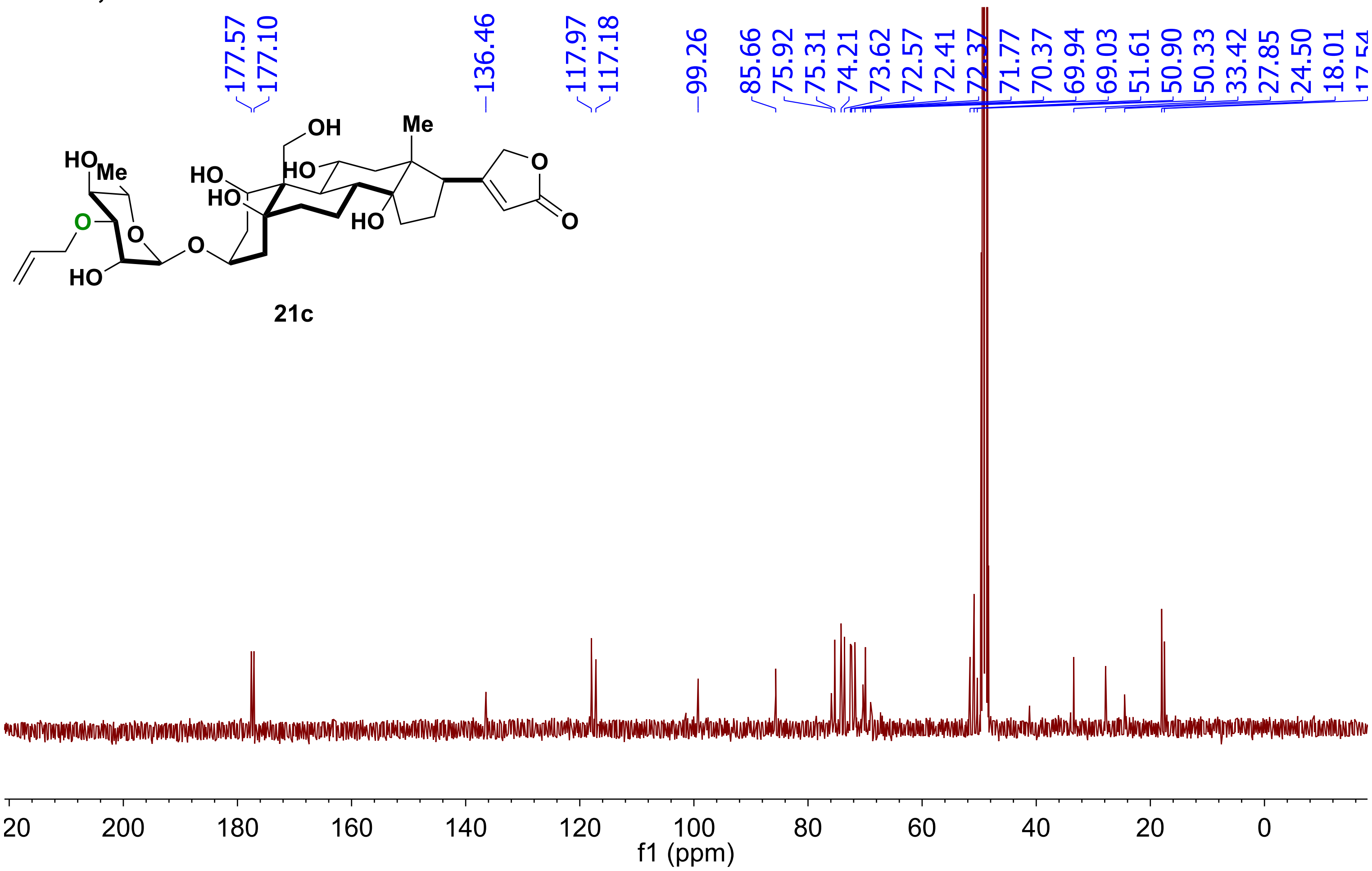

21c

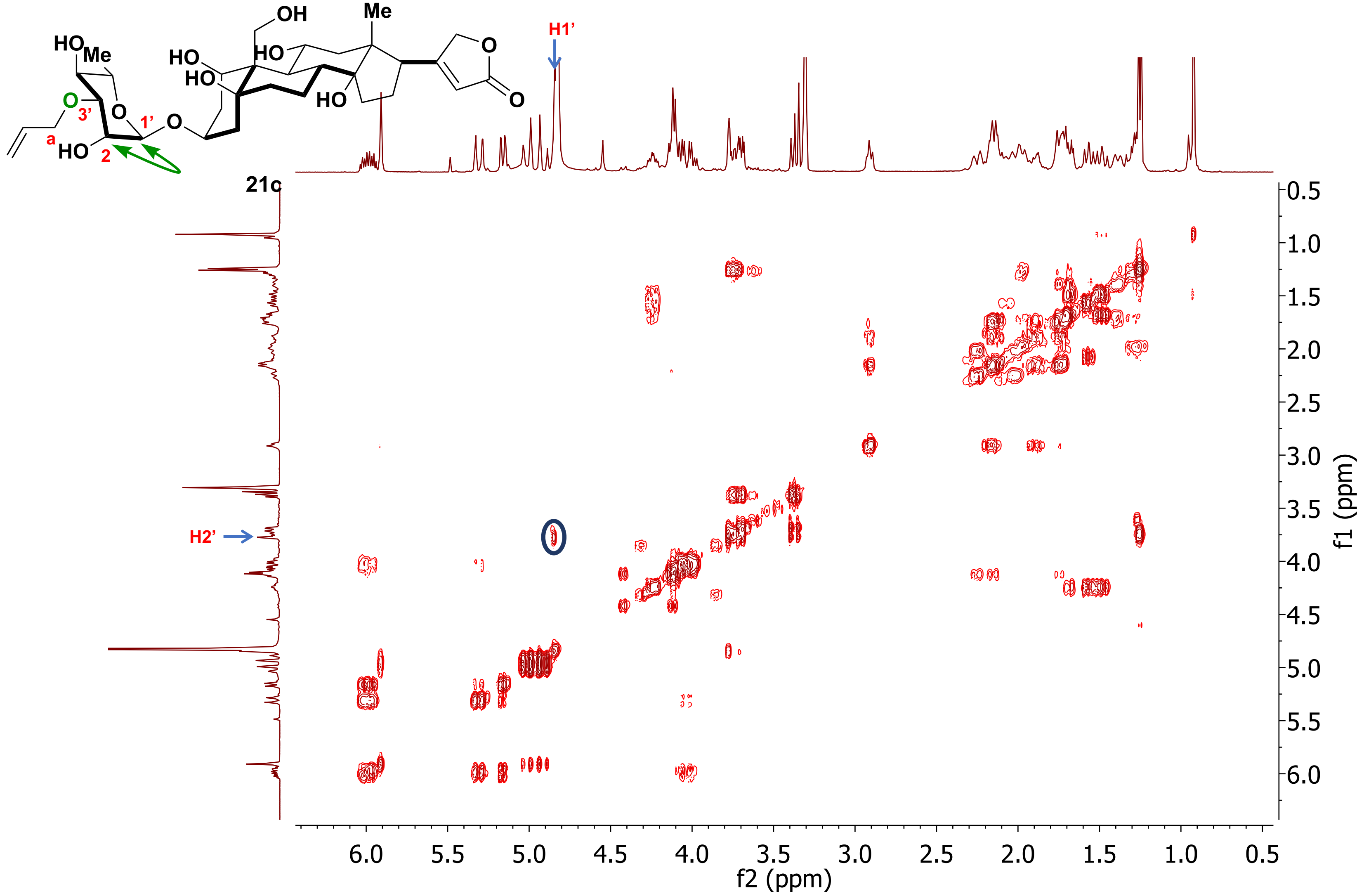

OH
Me
HO
Me
HO
HO
HO
O
O
3'
1'
O
O
O
a
HO
2
HO
21c
H1'
H2'
f1 (ppm)
f2 (ppm)

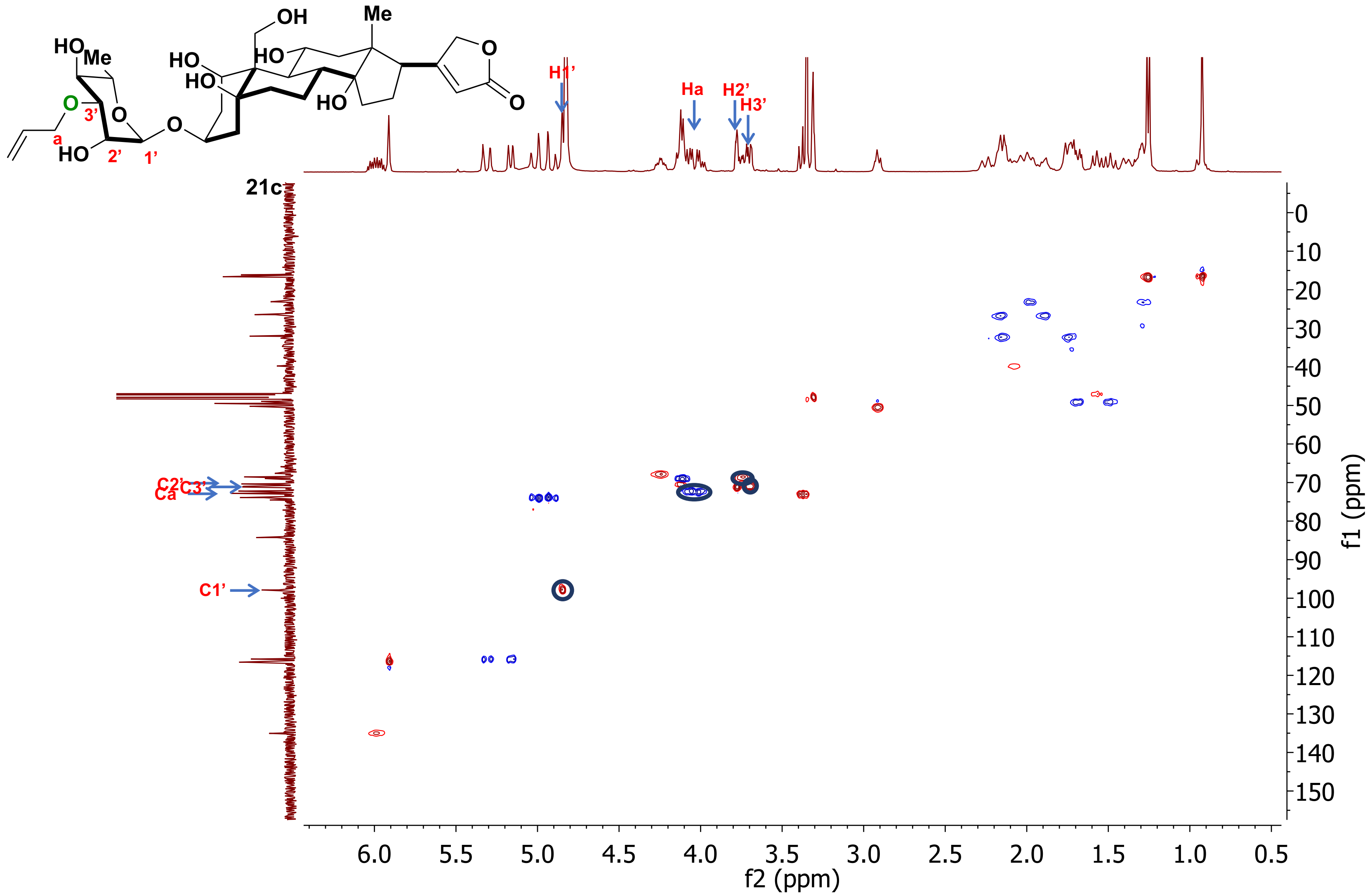

21c
H1'
Ha
H2'
H3'
C2' C3'
Ca
C1'
f2 (ppm)
f1 (ppm)

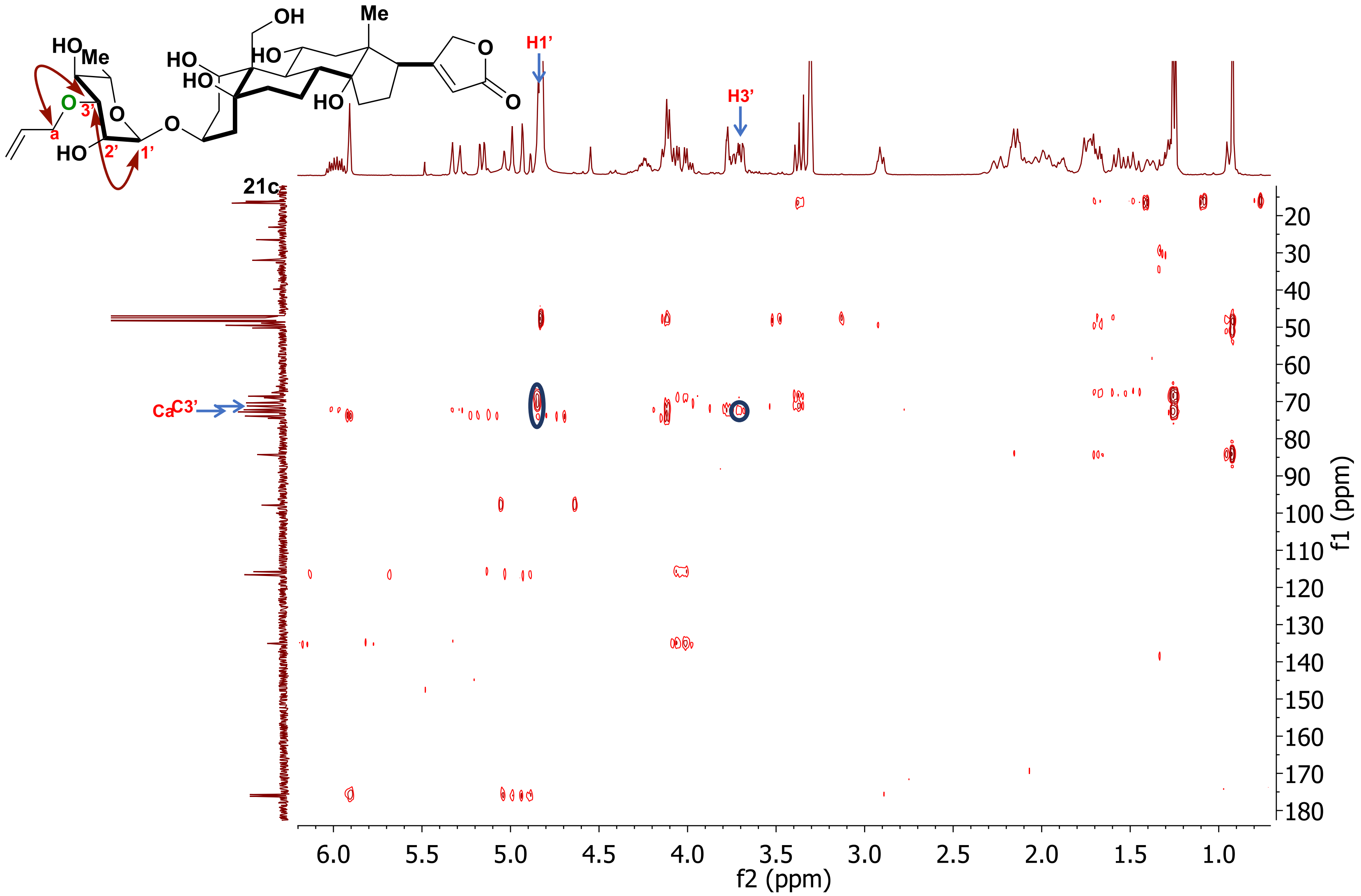

OH
Me
HO
Me
HO
HO
HO
O
O
3'
O
a
2'
1'
O
HO
HO
O
H1'
H3'
21c
Ca
C3'
f1 (ppm)
f2 (ppm)

MeOD, 400.13 MHz

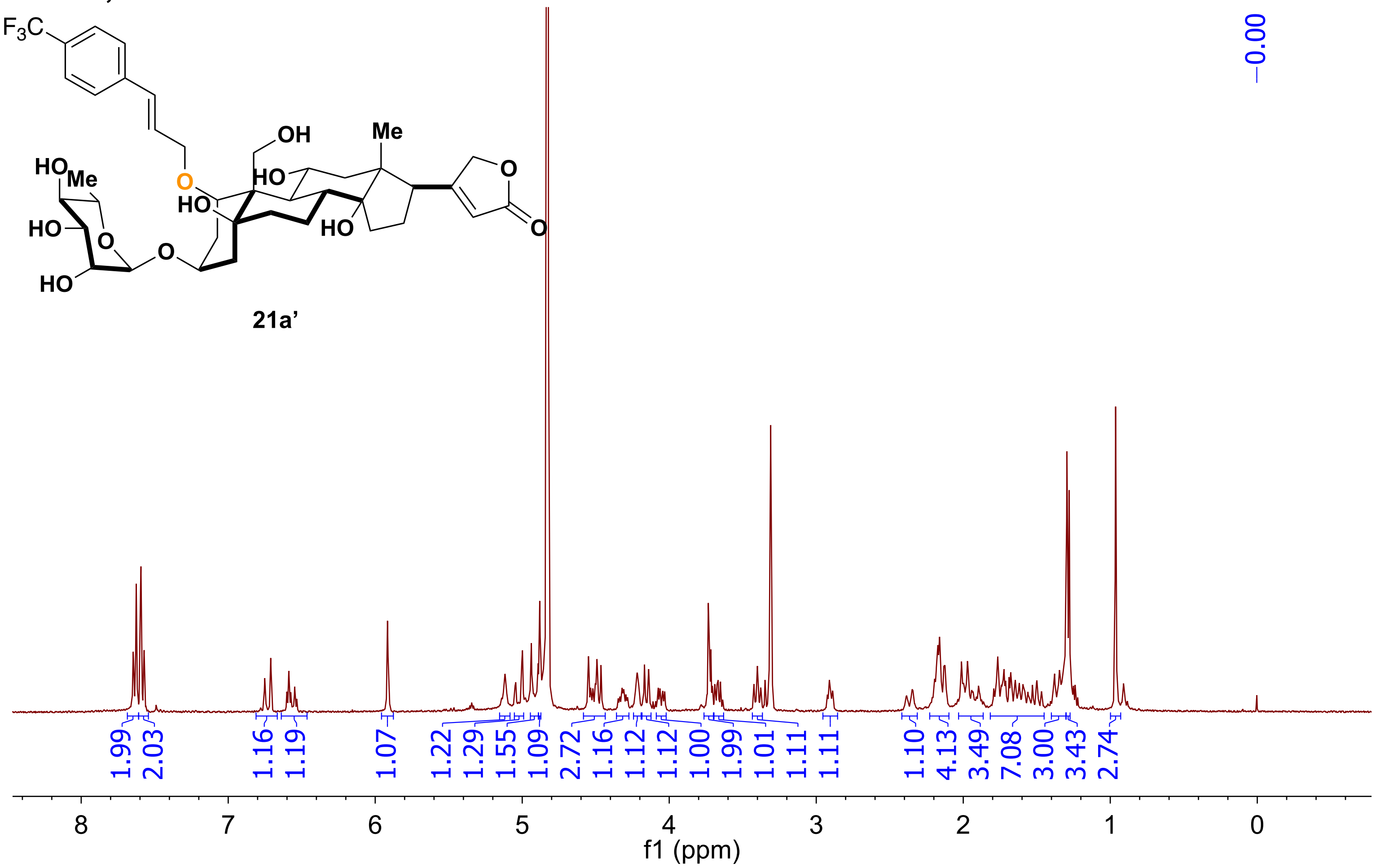

21a’

MeOD, 100.62 MHz

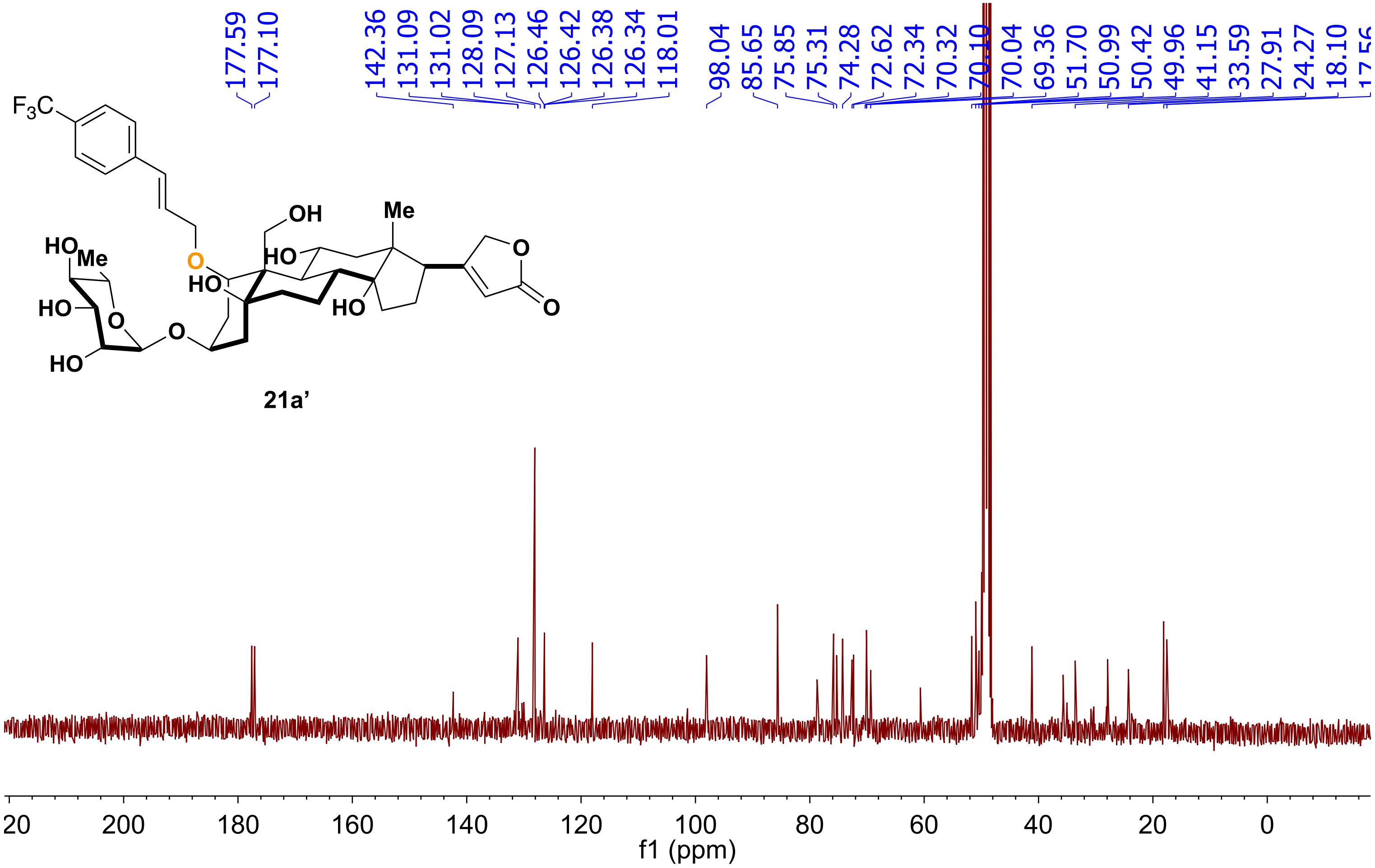

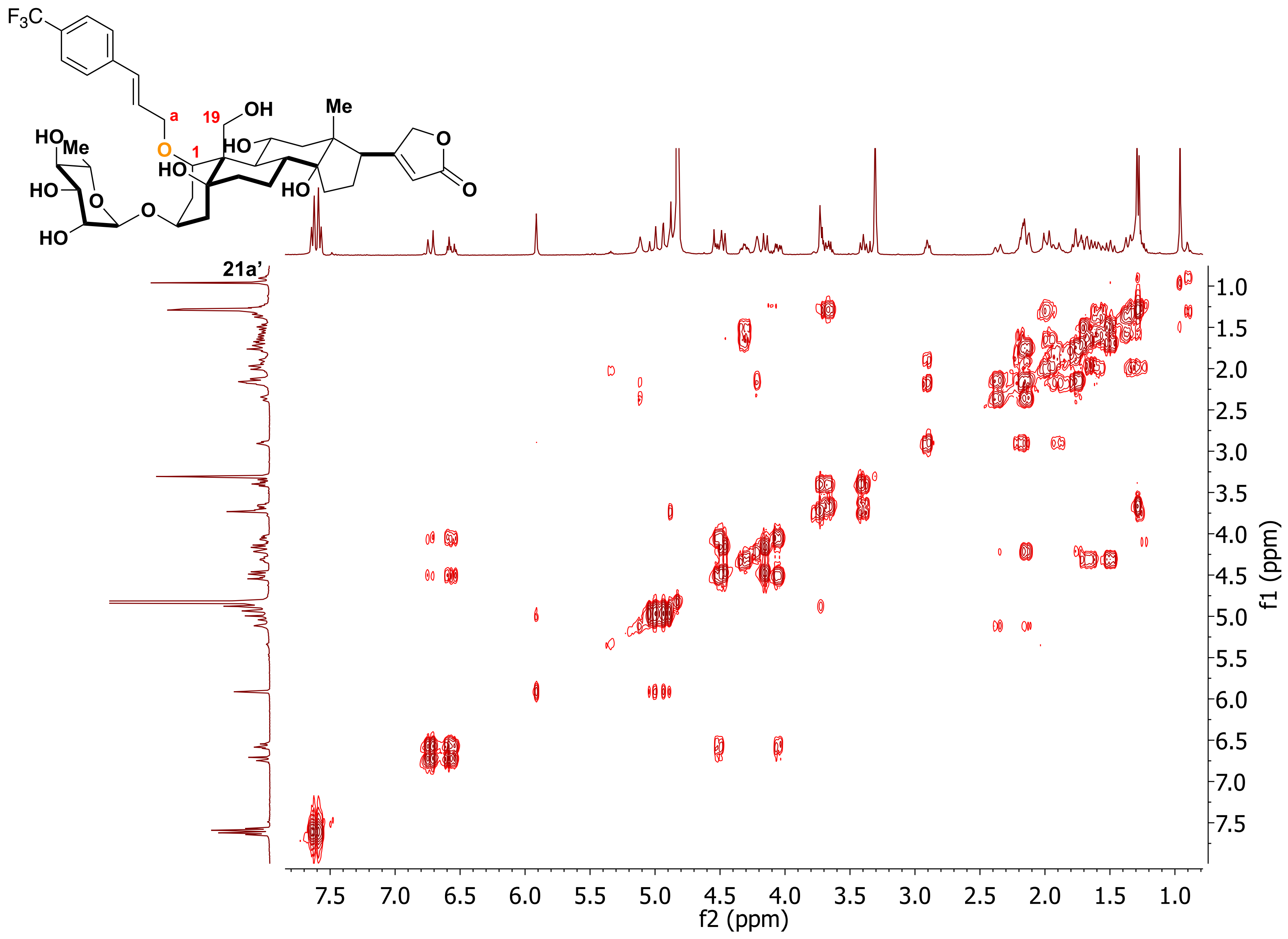
F3C
a
19
OH
Me
O
1
HO
HO
HO
Me
HO
O
O
HO
HO
O
O
21a'
f2 (ppm)
f1 (ppm)

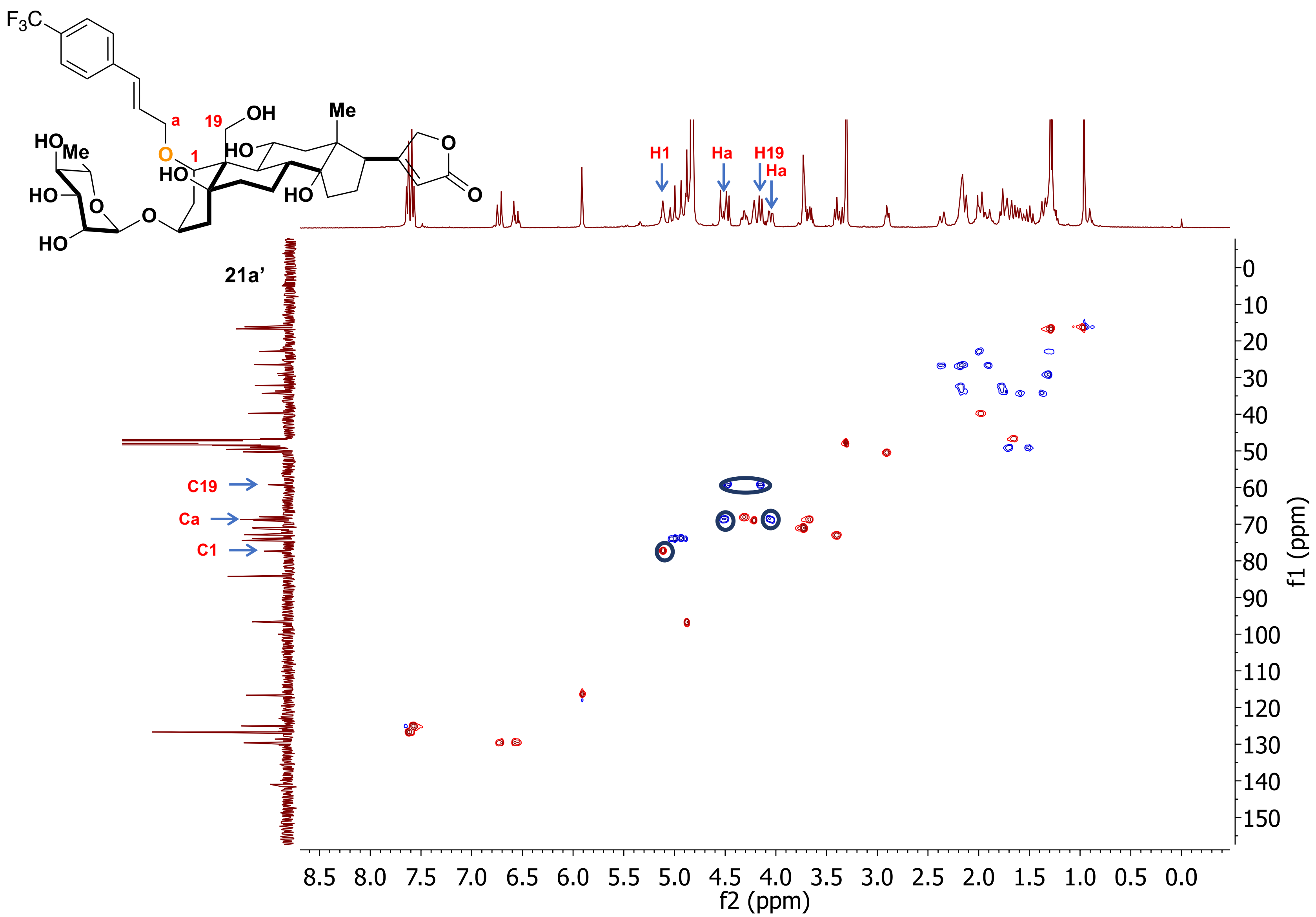
F3C
a
19
OH
Me
HO
Me
O
1
HO
HO
O
O
O
HO
HO
HO
O
21a'
H1
Ha
H19
Ha
C19
Ca
C1
f2 (ppm)
f1 (ppm)

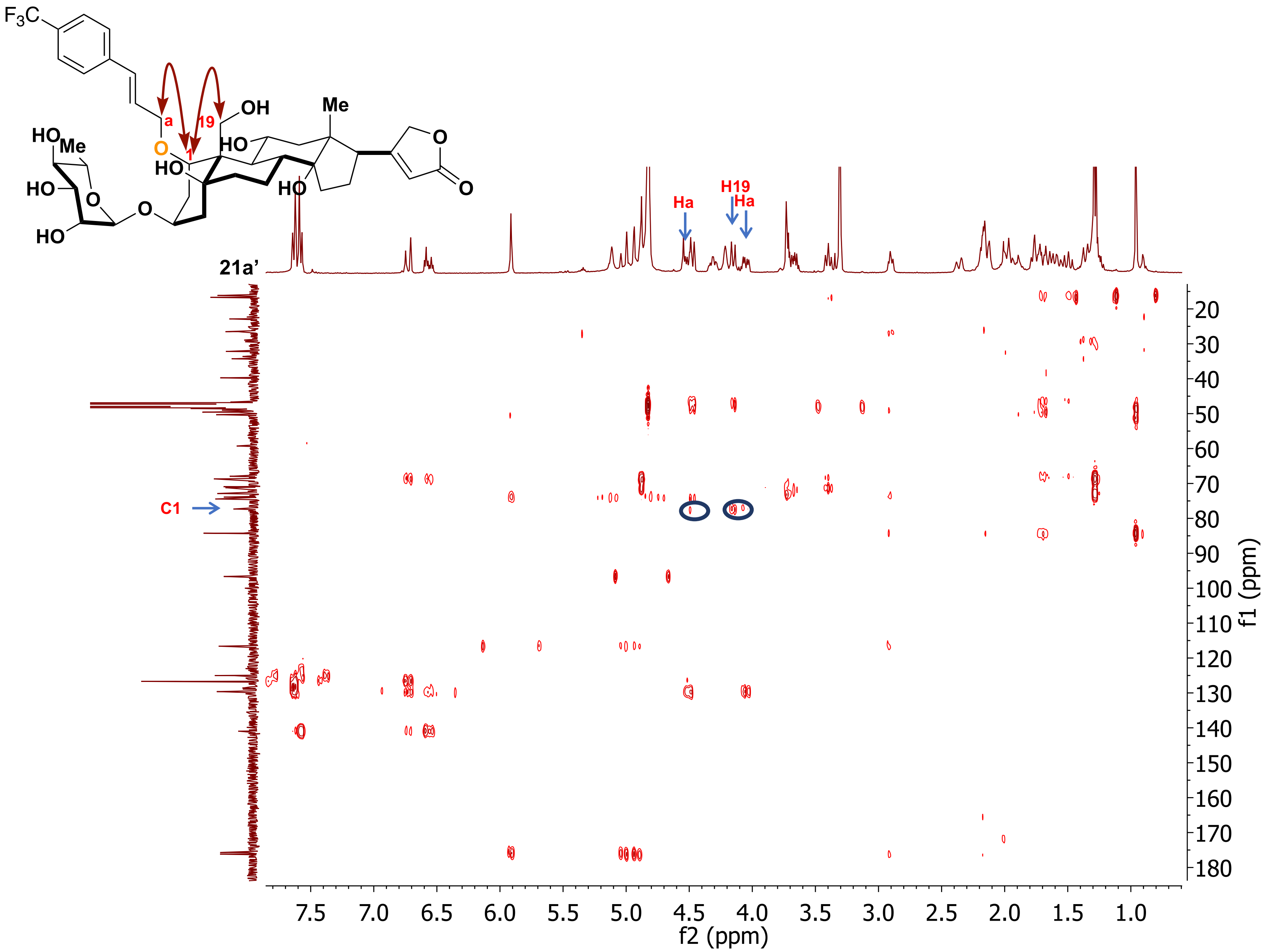
F3C
OH
Me
HO
Me
O
a
19
1
HO
HO
O
O
O
HO
HO
HO
O
21a'
Ha
H19
Ha
C1
f2 (ppm)
f1 (ppm)

MeOD, 400.13 MHz

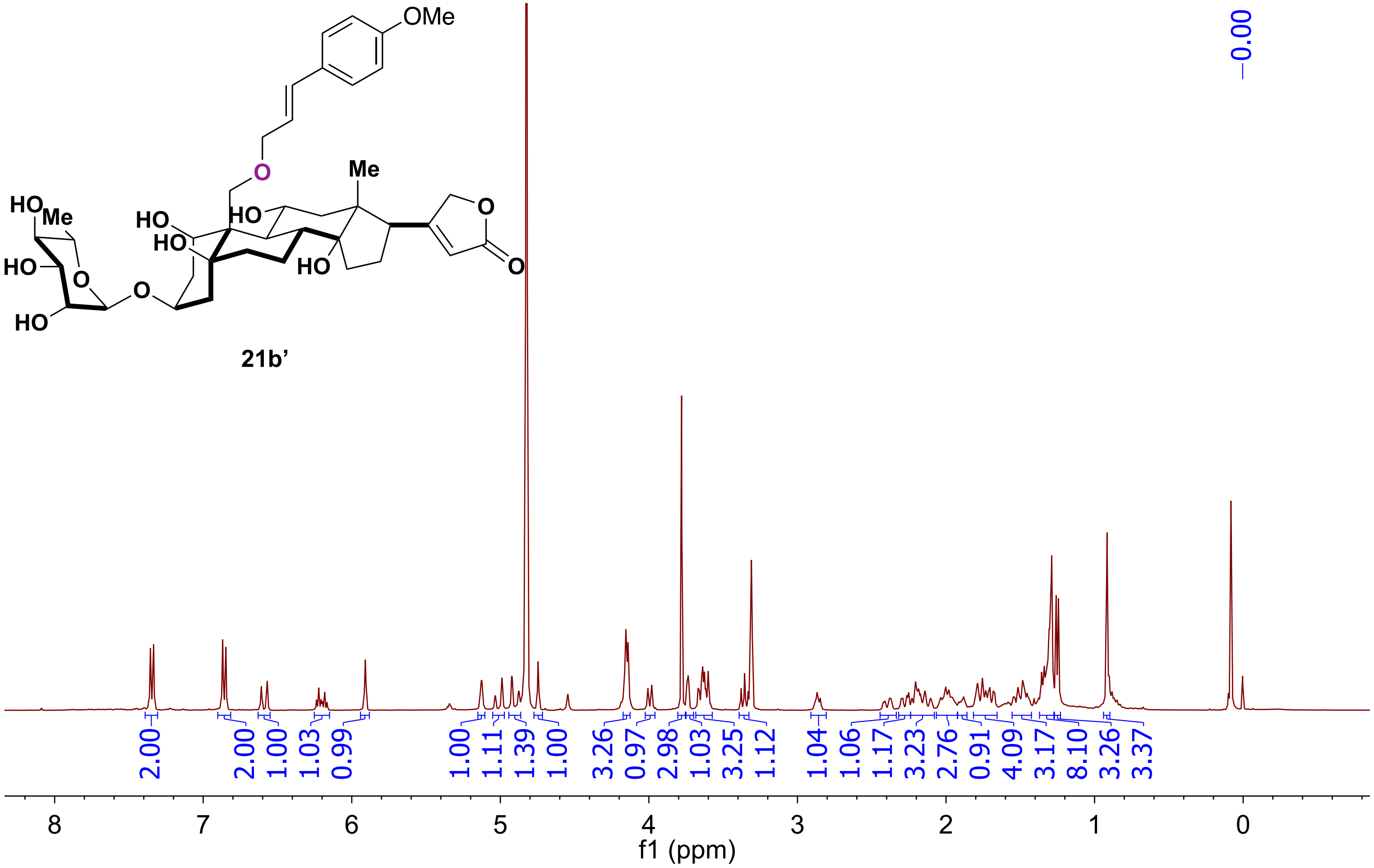

MeOD, 100.62 MHz

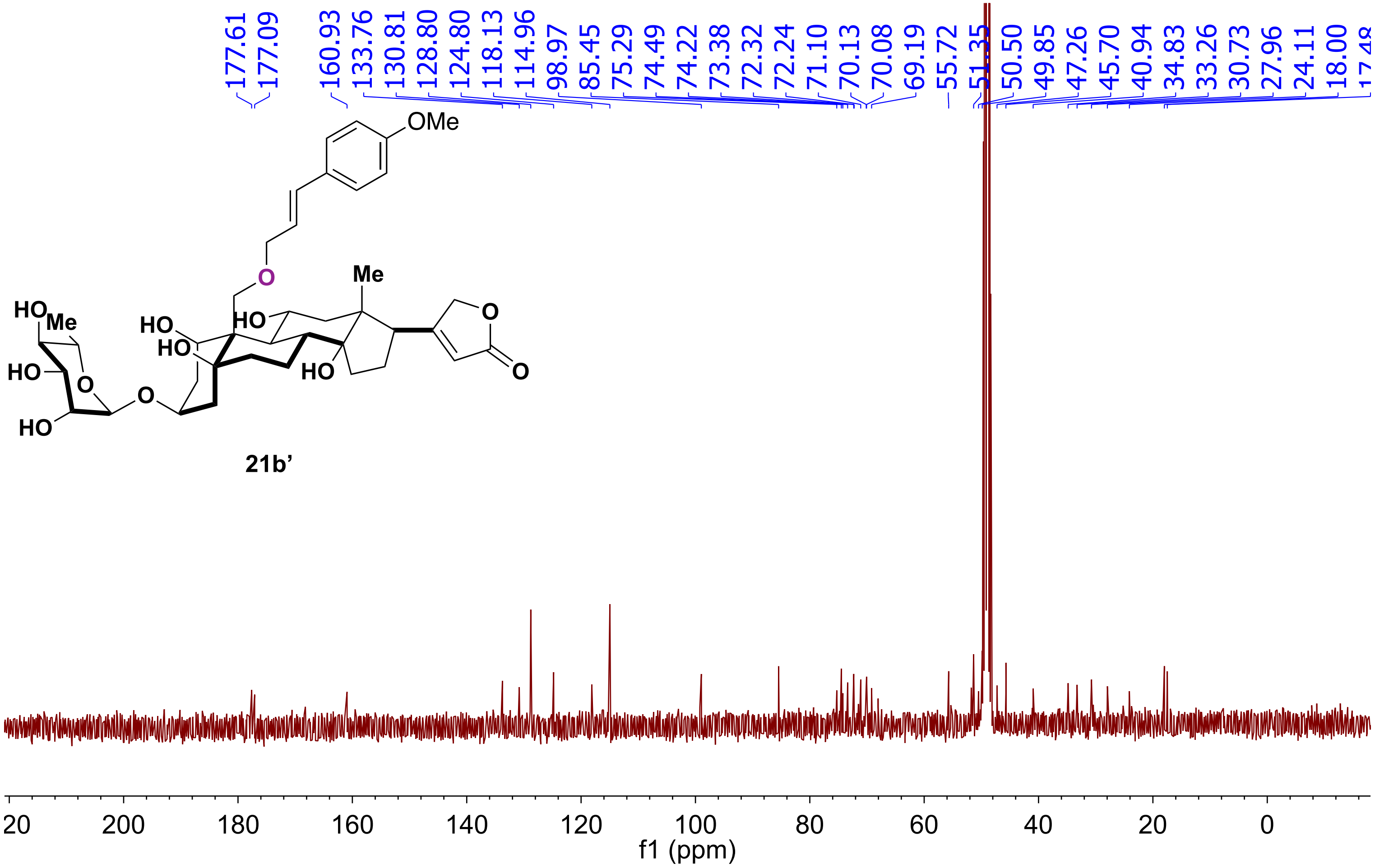

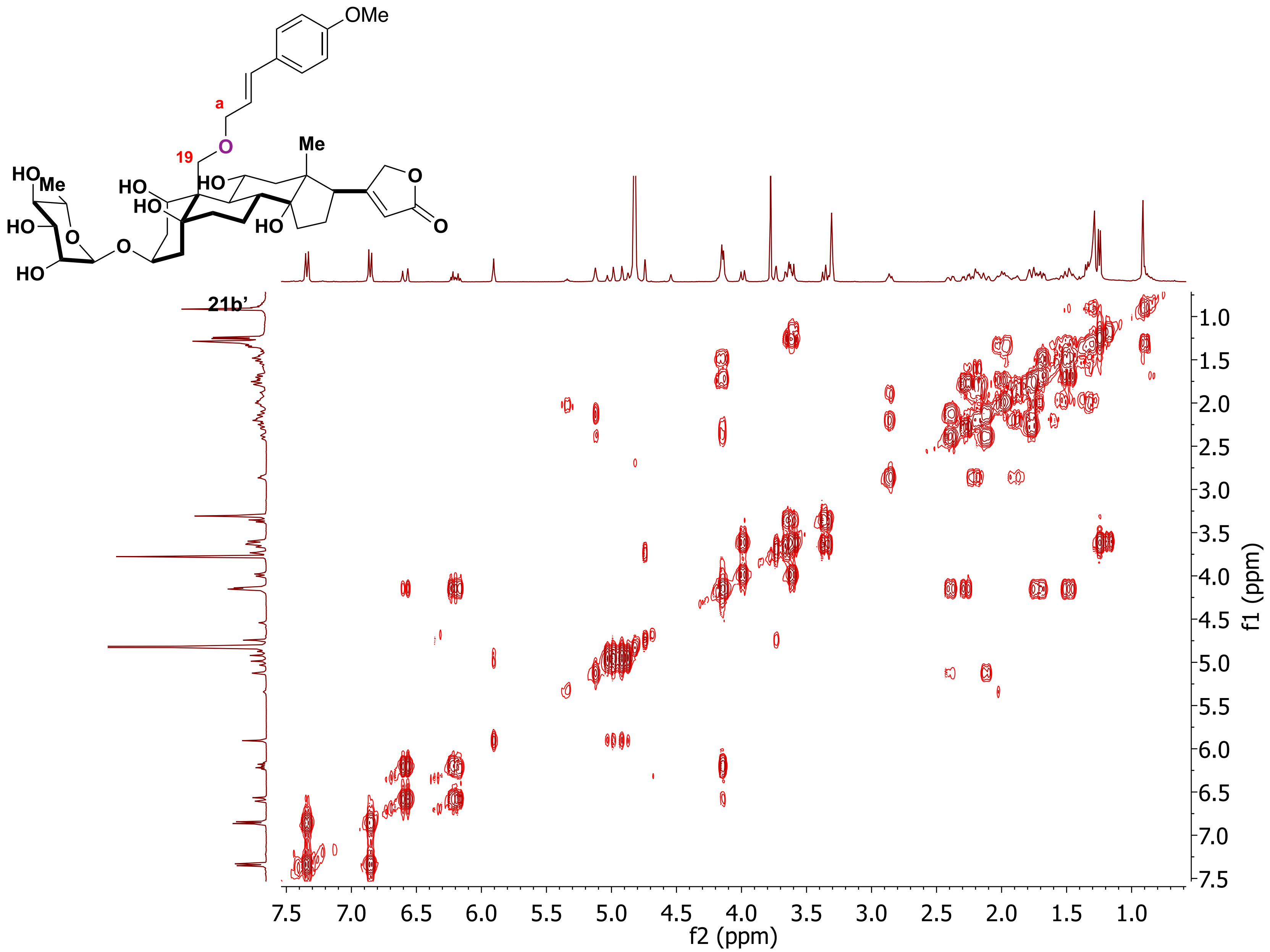
OMe
a
19
O
Me
HO
Me
HO
HO
HO
HO
O
O
HO
HO
O
O
21b'
f1 (ppm)
f2 (ppm)

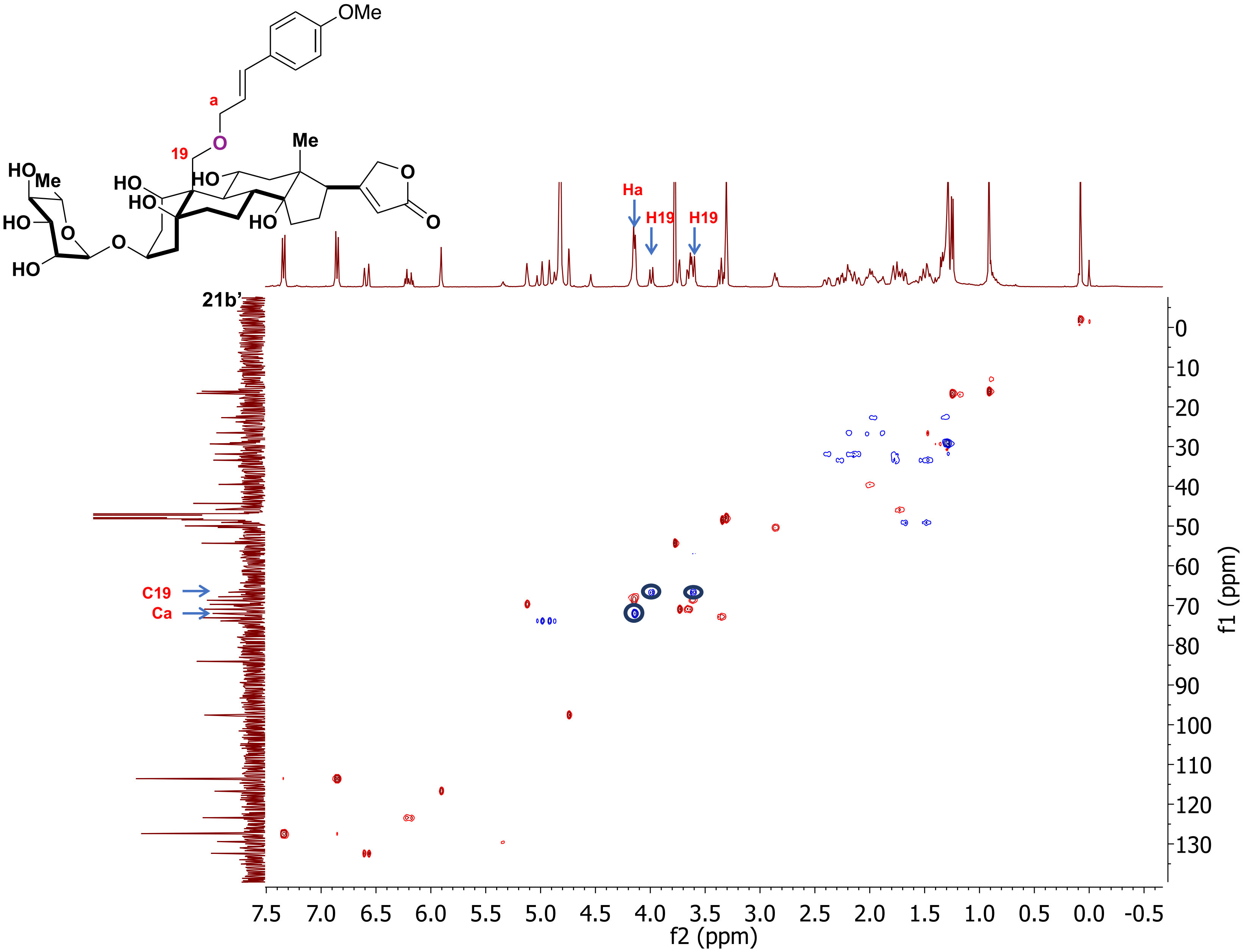

OMe
a
19
O
Me
HO
Me
HO
HO
HO
O
O
HO
HO
O
O
HO
Ha
H19
H19
21b'
C19
Ca
f2 (ppm)
f1 (ppm)

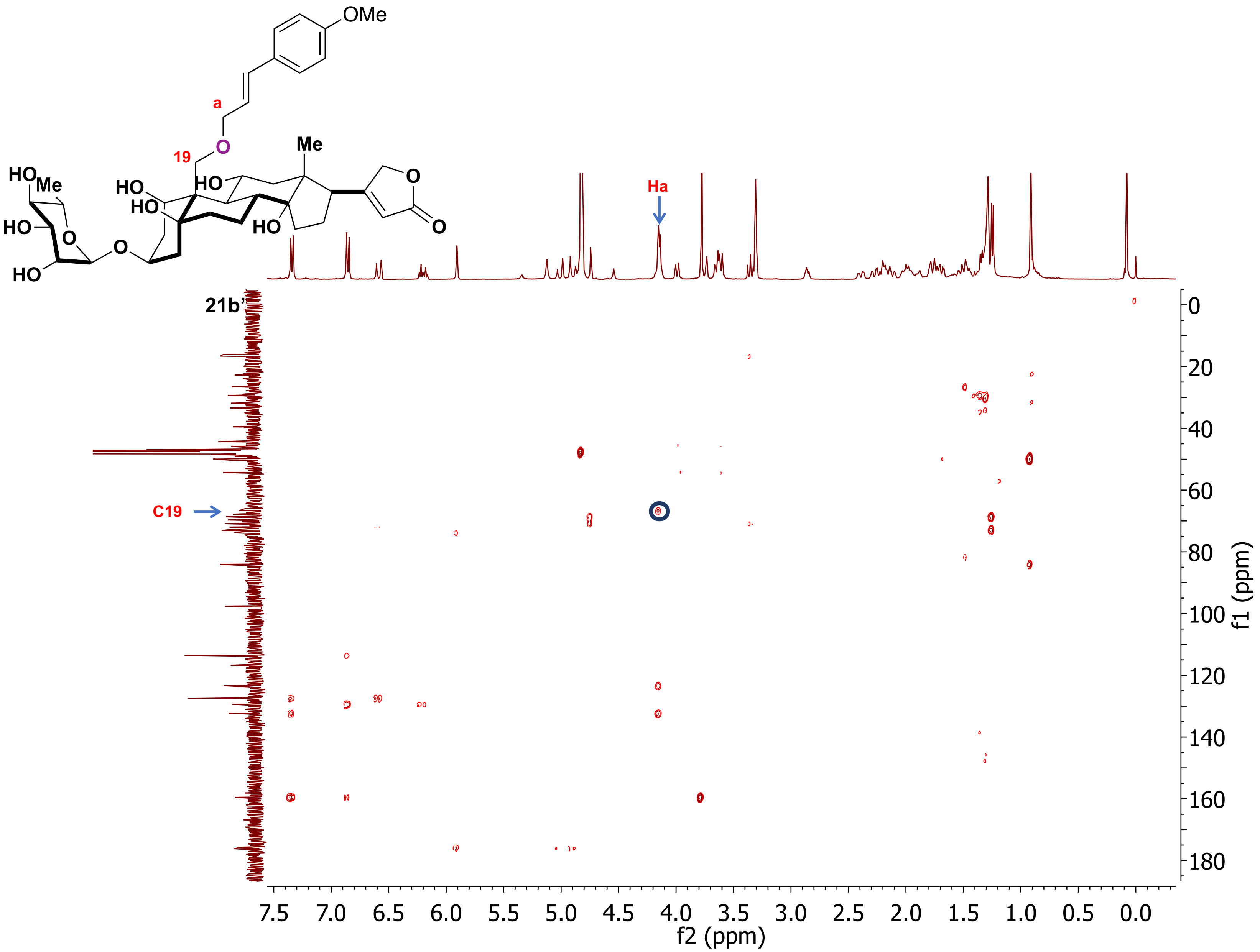
21b'
Ha
C19
a
19
f2 (ppm)
f1 (ppm)

MeOD, 400.13 MHz

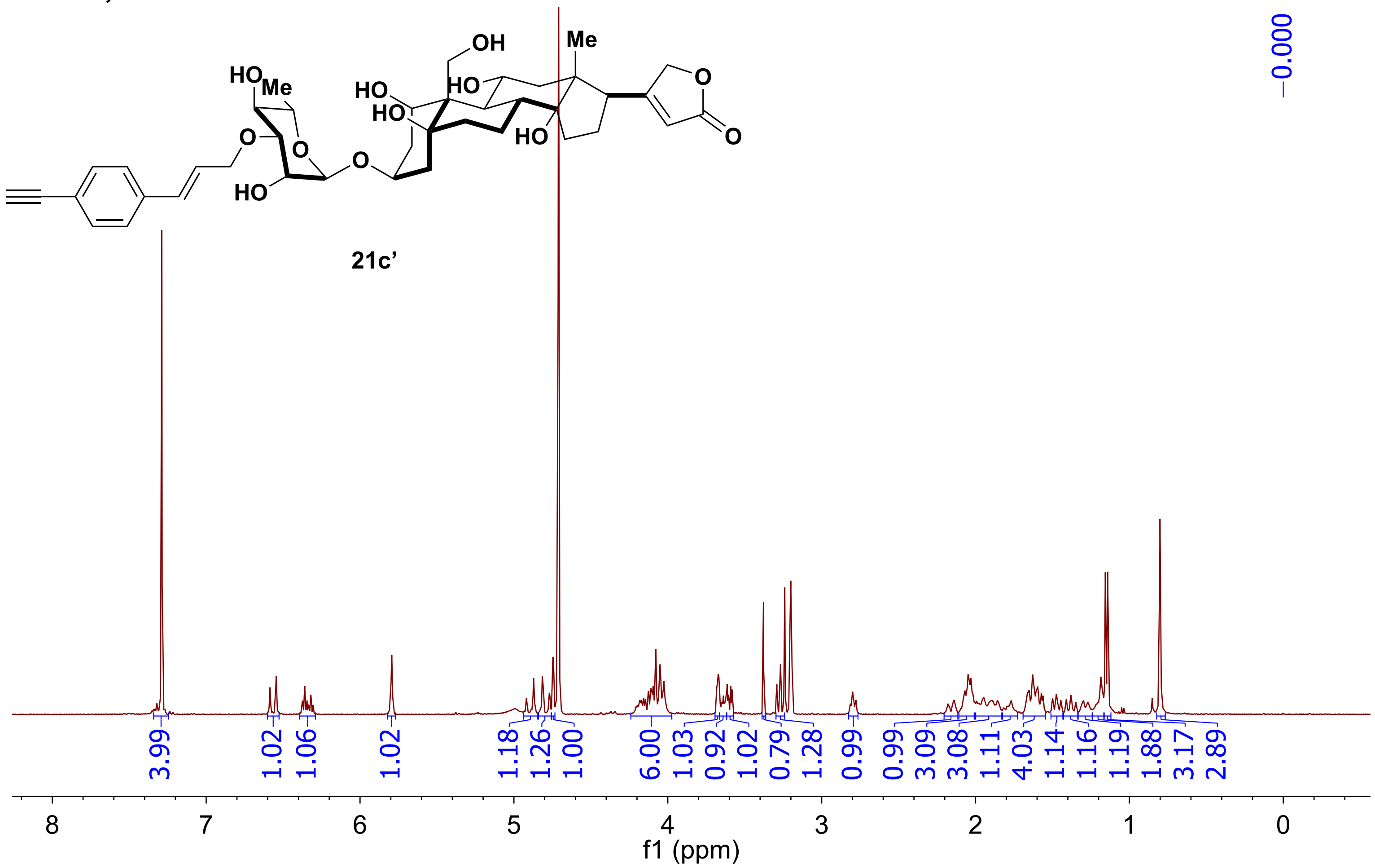

MeOD, 100.62 MHz

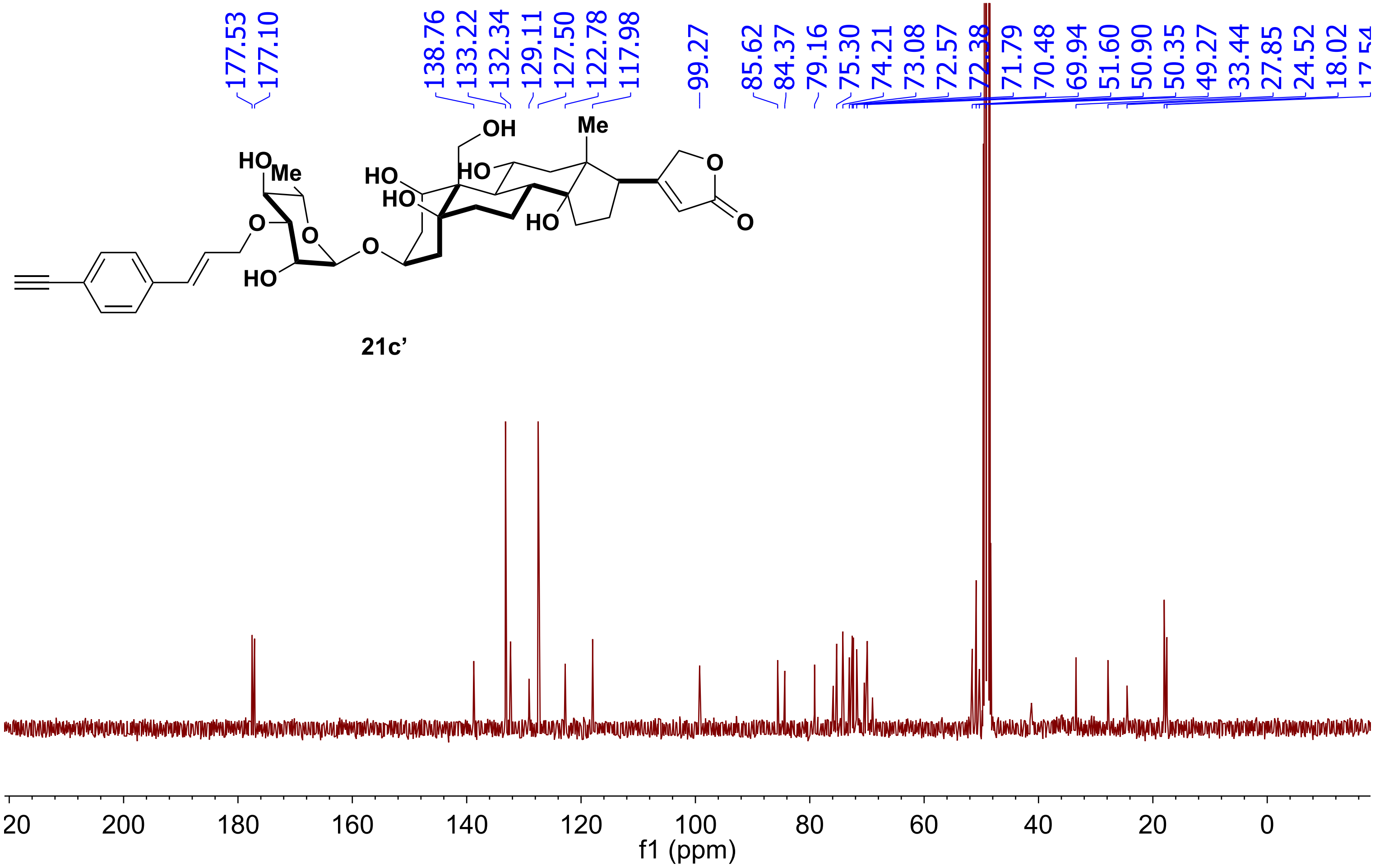

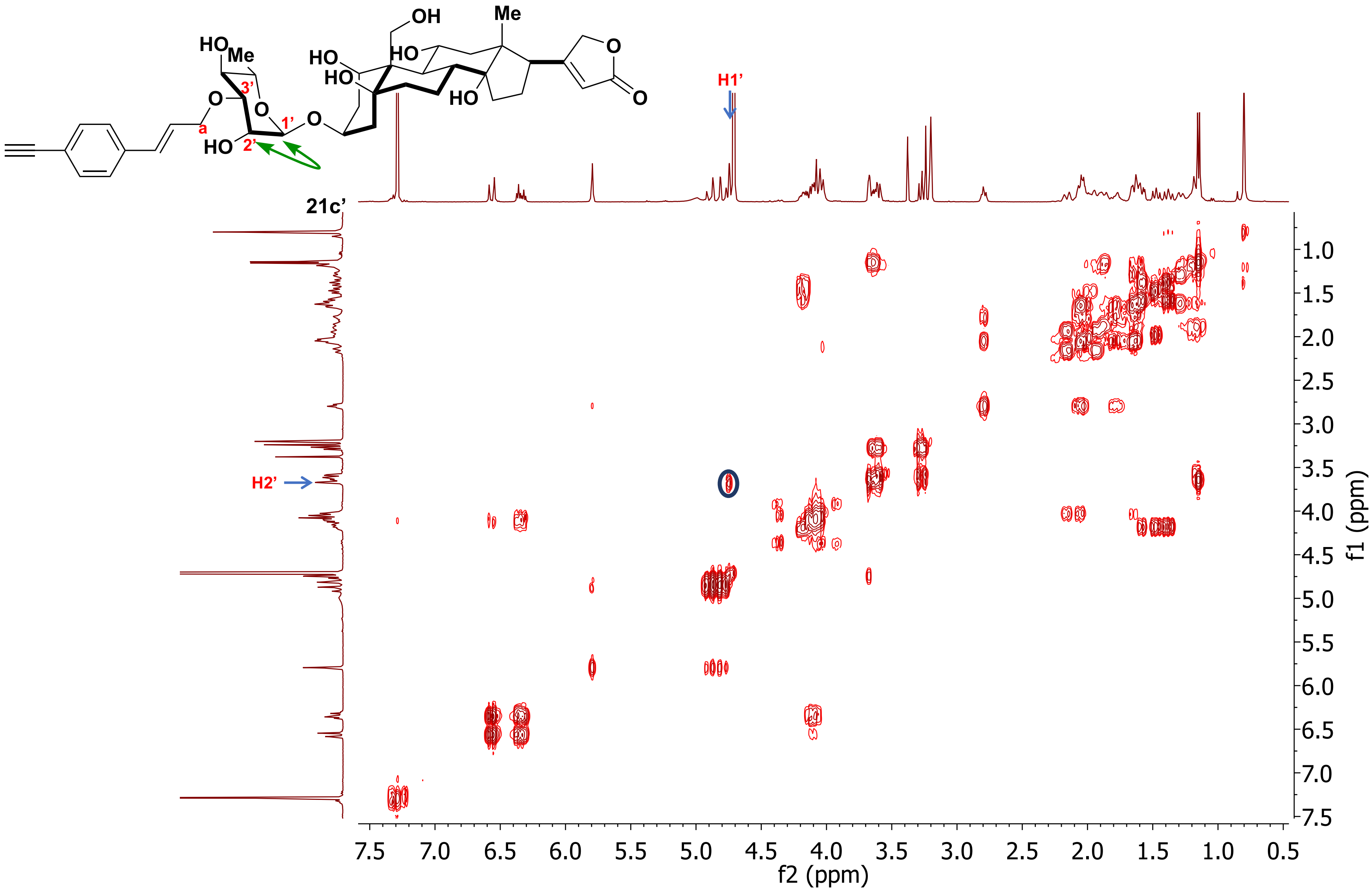

21c'
H1'
H2'
f2 (ppm)
f1 (ppm)

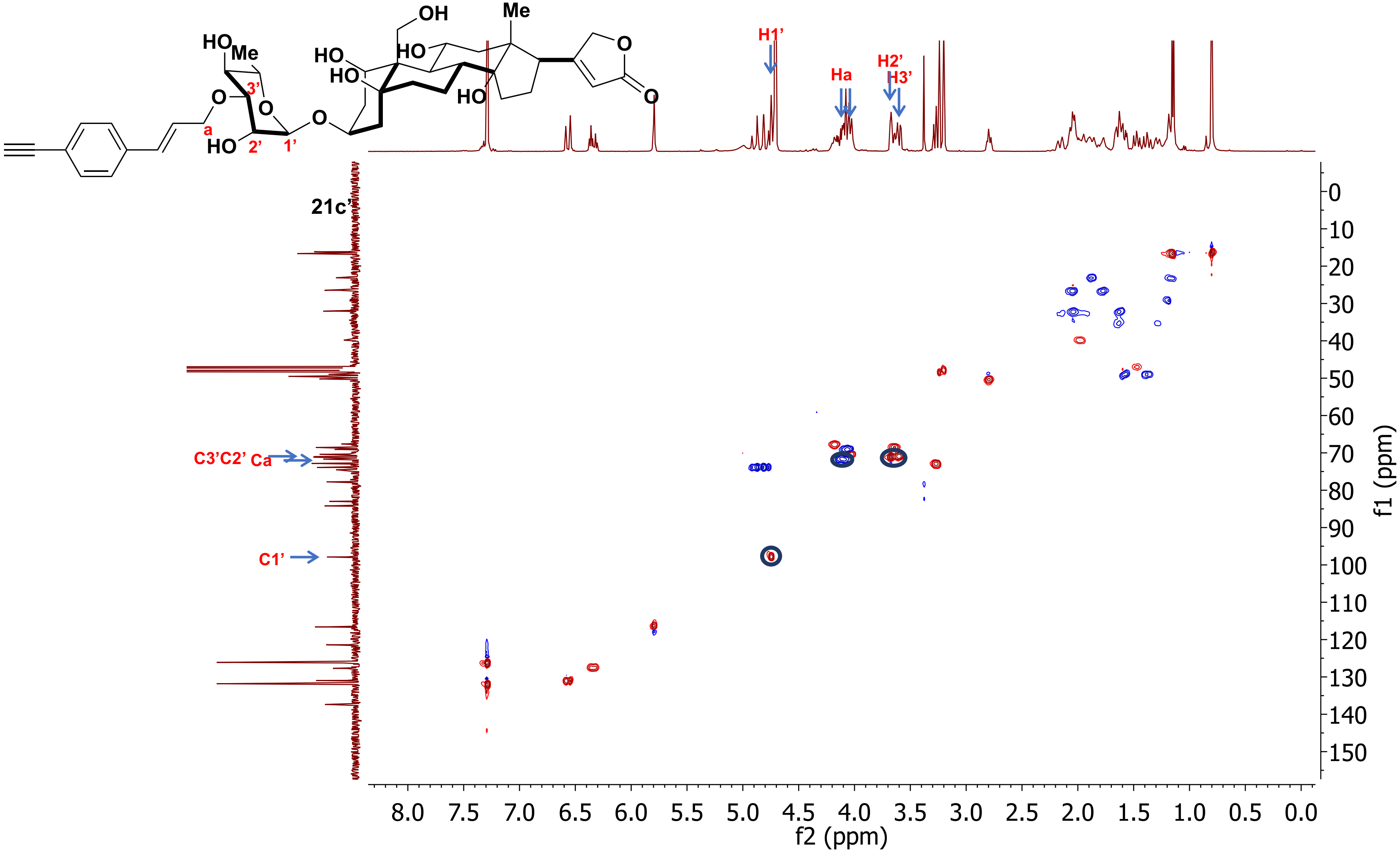

21c'
H1'
Ha
H2'
H3'
C3'C2' Ca
C1'
f2 (ppm)
f1 (ppm)

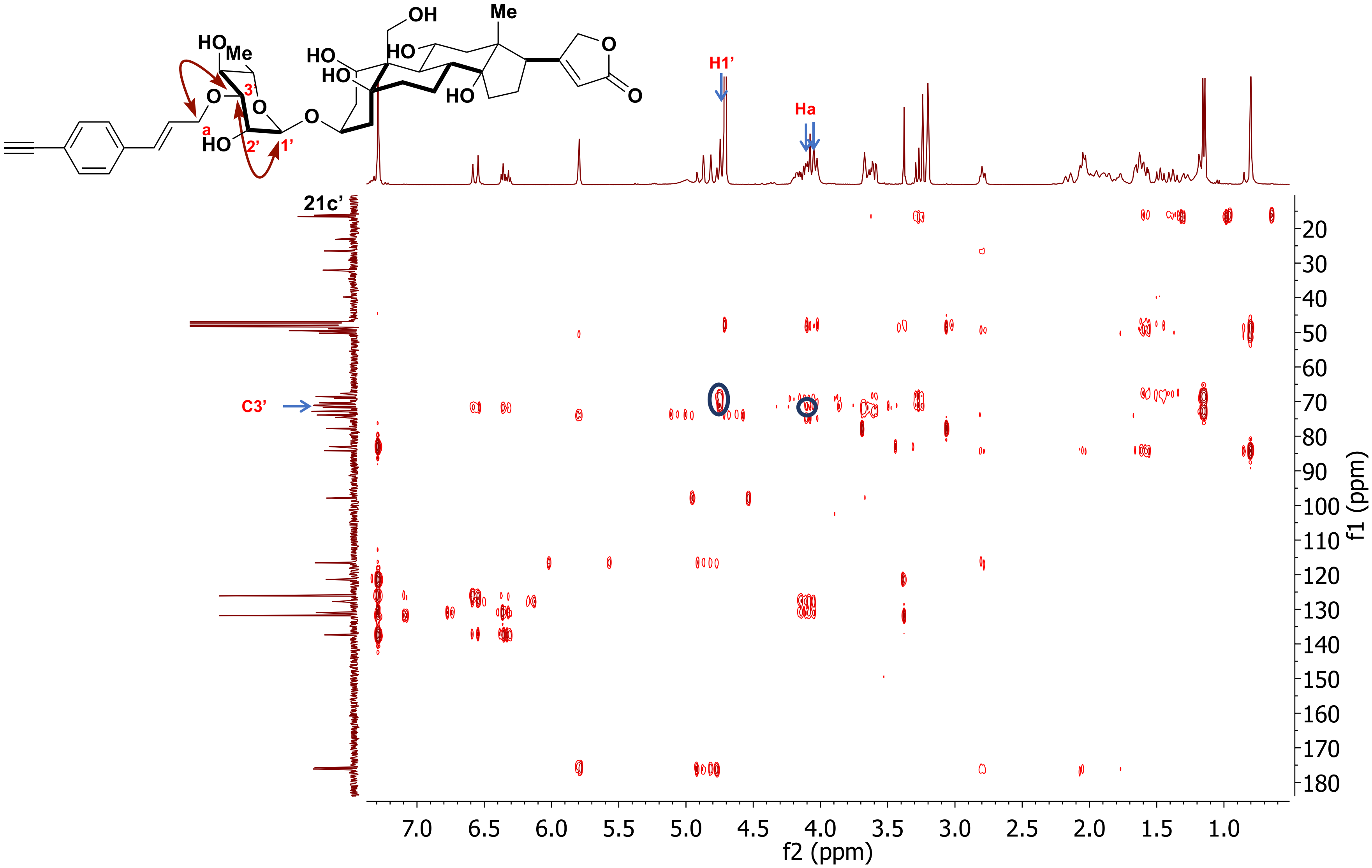

21c'
H1'
Ha
C3'
a
2'
1'
3'
f2 (ppm)
f1 (ppm)

MeOD, 400.13 MHz

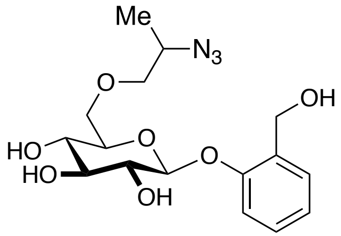

22a

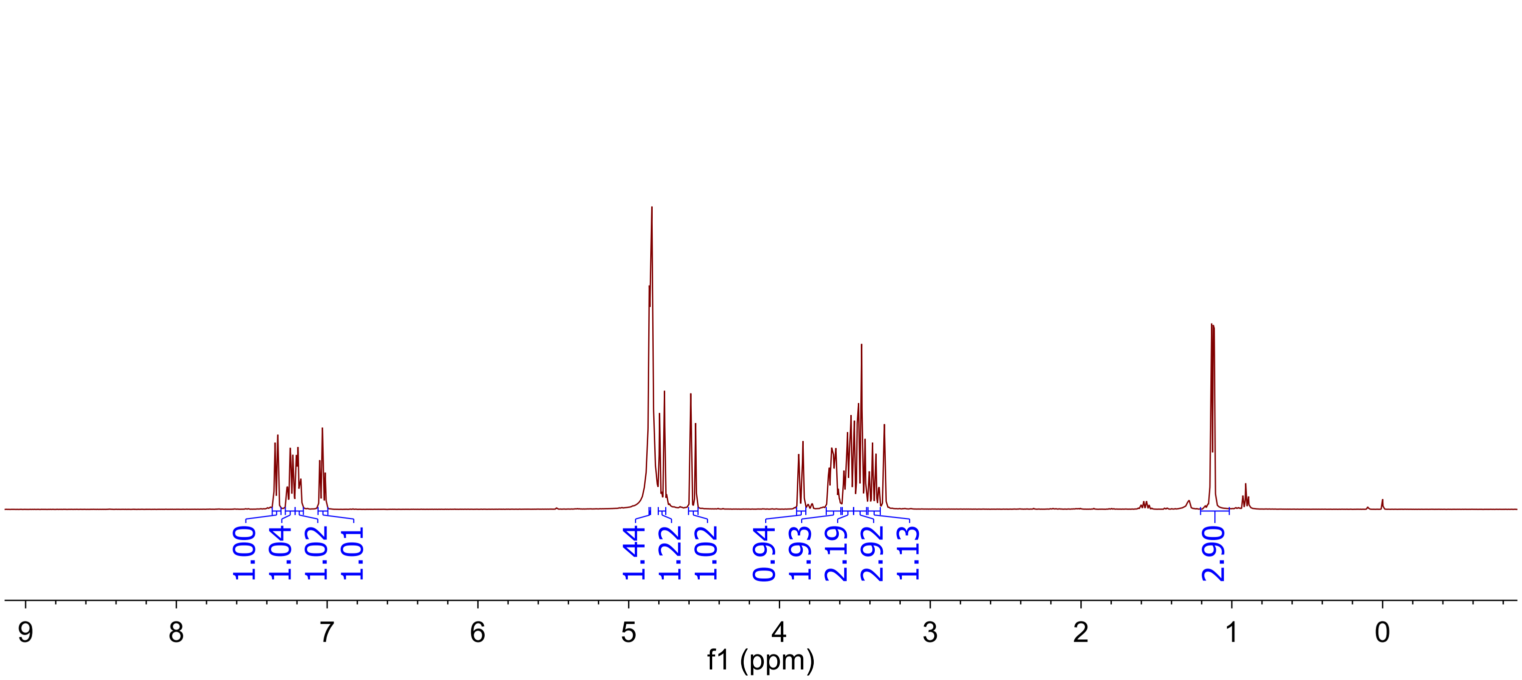

MeOD, 100.62 MHz

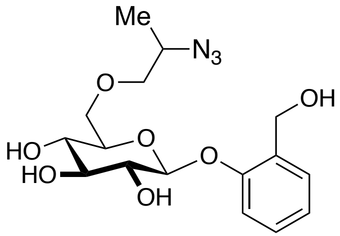

22a

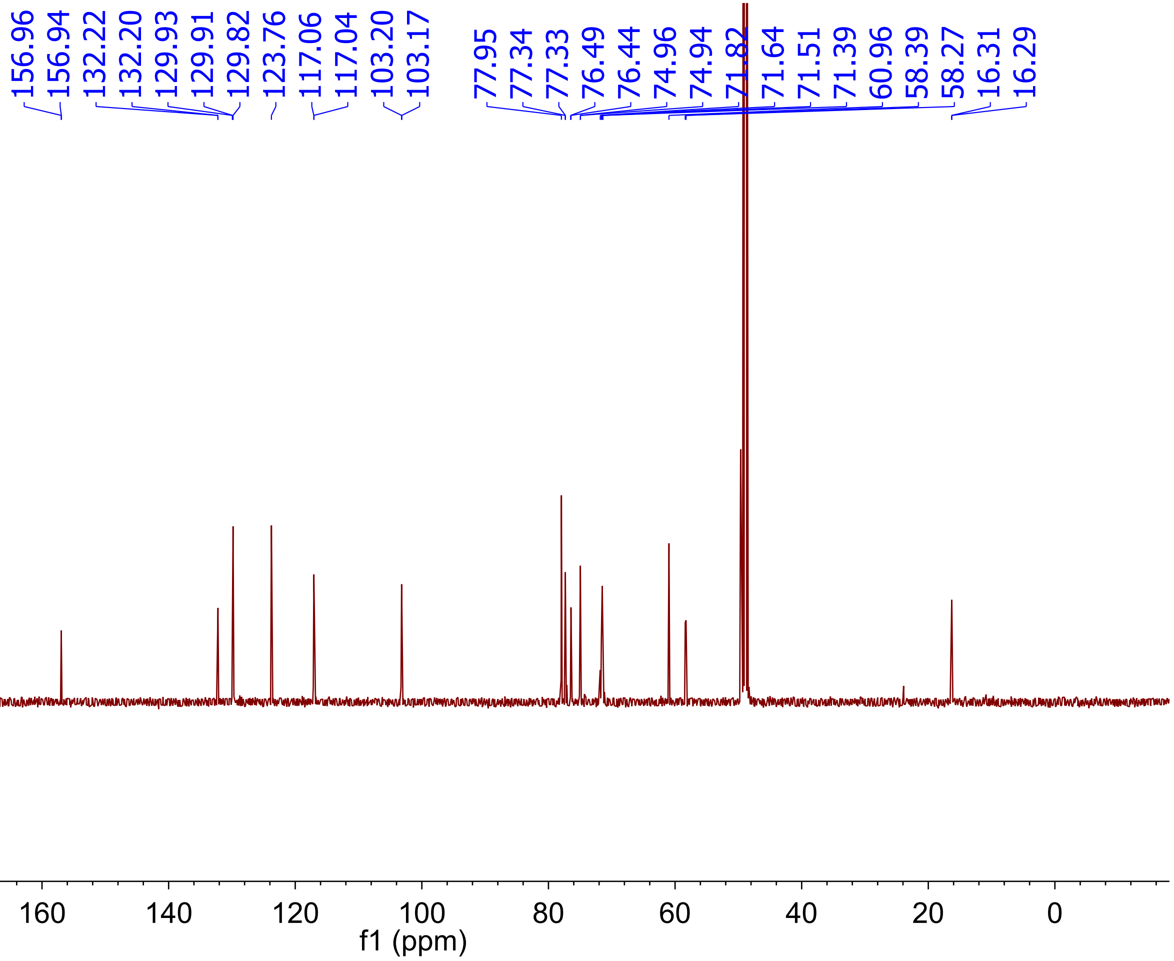

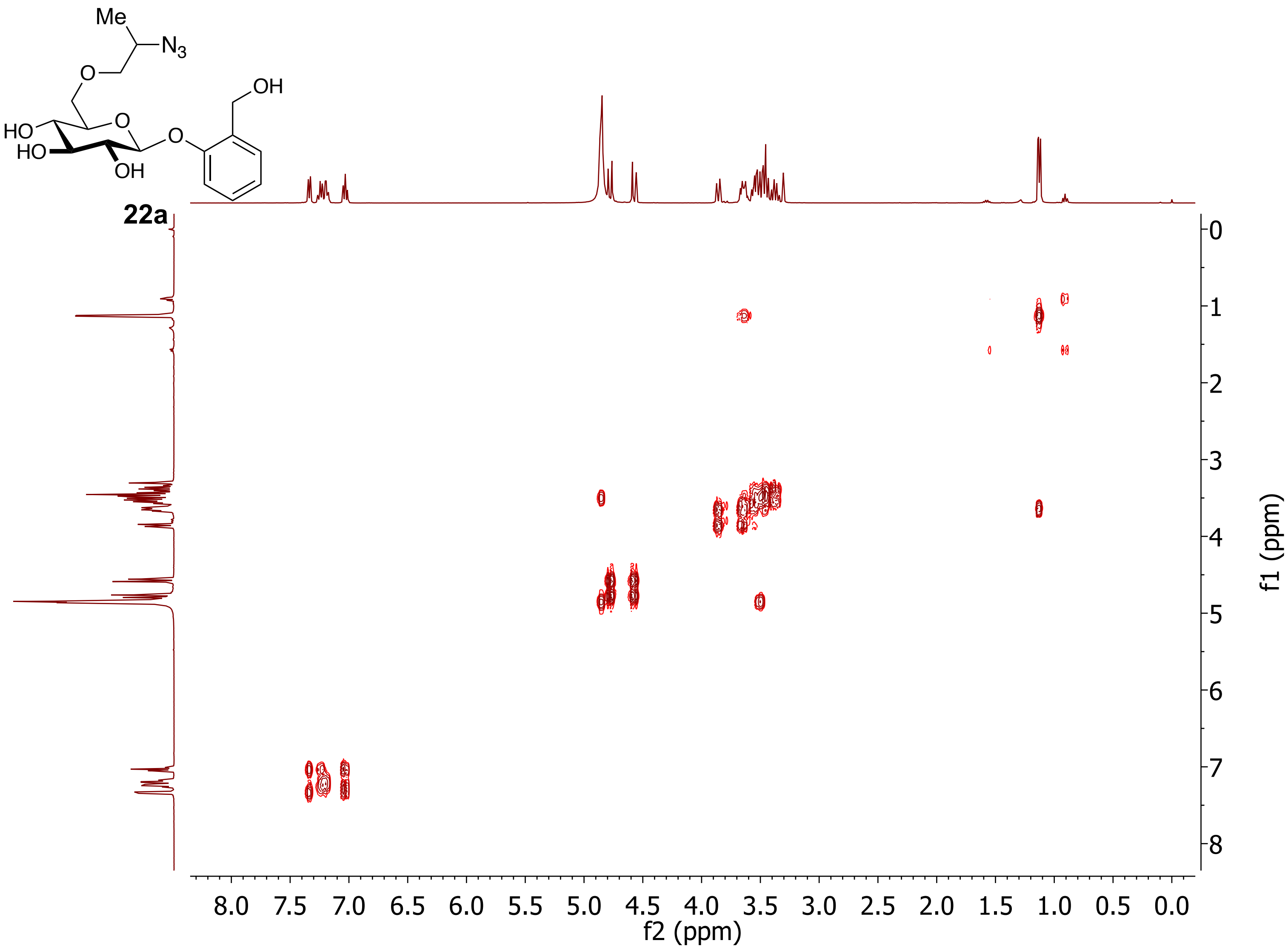

Me
N3
O
OH
HO
O
O
HO
OH
8.0 7.5 7.0 6.5 6.0 5.5 5.0 4.5 4.0 3.5 3.0 2.5 2.0 1.5 1.0 0.5 0.0
f2 (ppm)
0 1 2 3 4 5 6 7 8
f1 (ppm)

22a

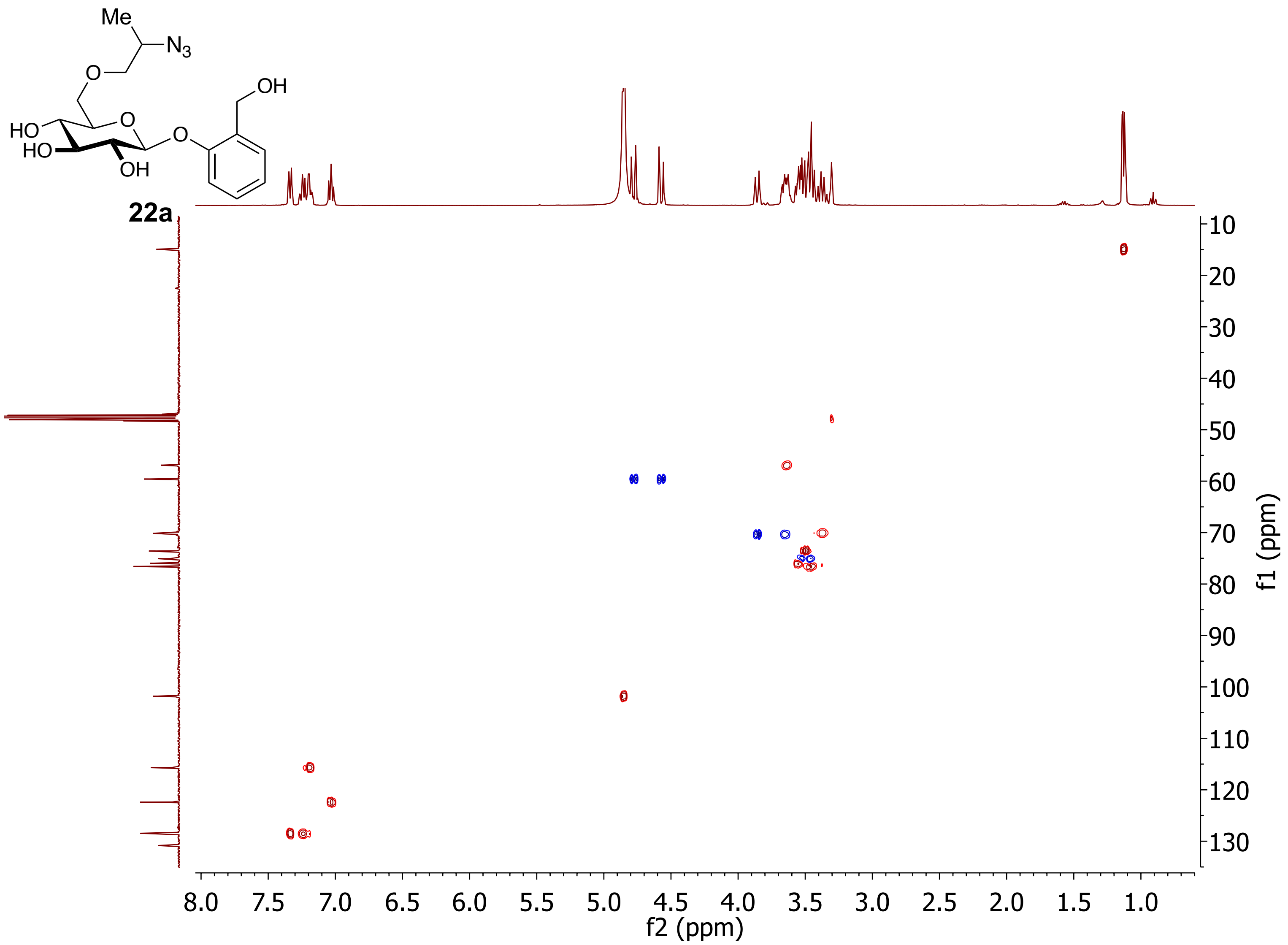
Me
N3
O
OH
HO
HO
O
O
OH
22a
f2 (ppm)
f1 (ppm)
8.0 7.5 7.0 6.5 6.0 5.5 5.0 4.5 4.0 3.5 3.0 2.5 2.0 1.5 1.0
10 20 30 40 50 60 70 80 90 100 110 120 130

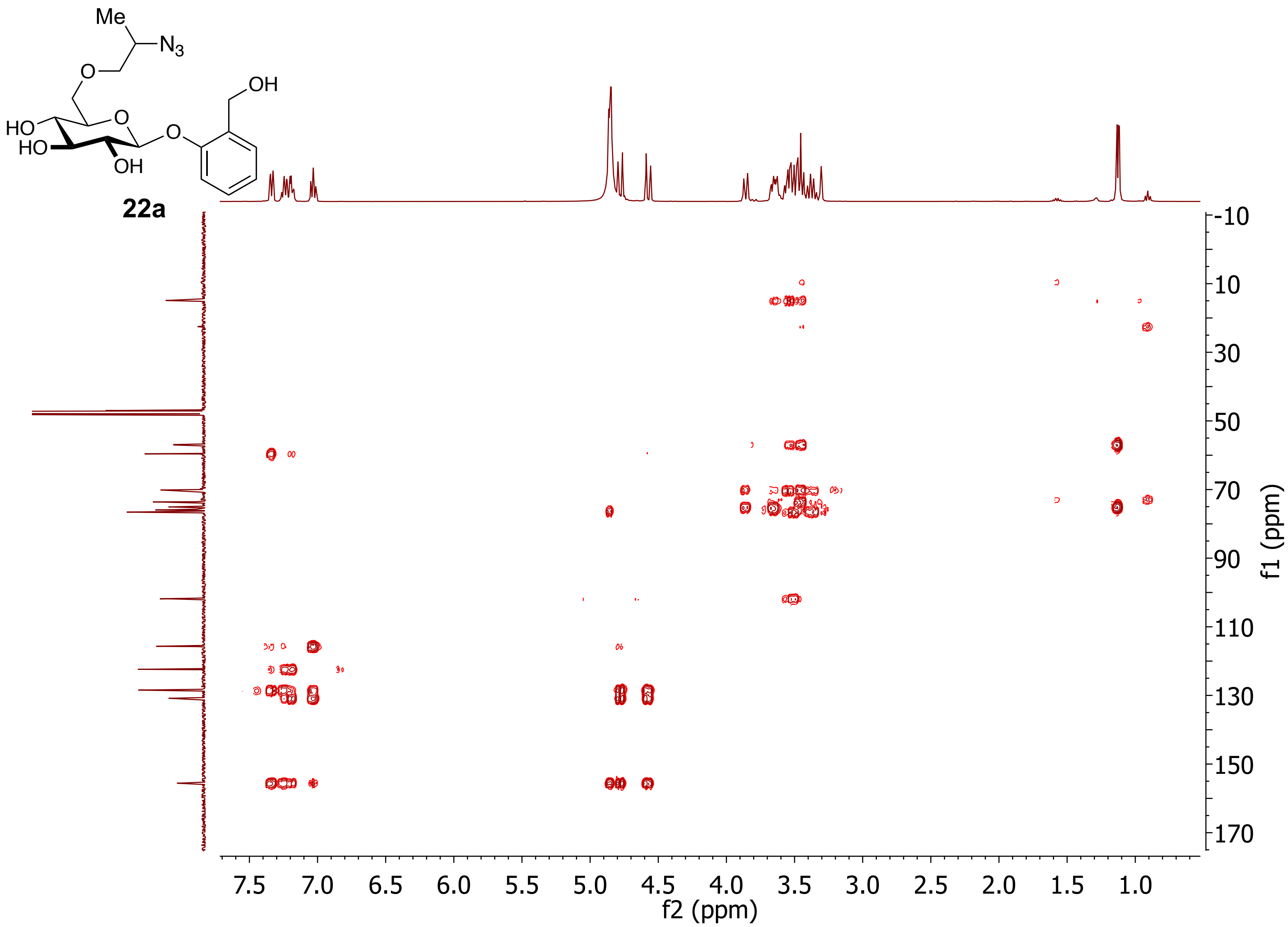

Me
N3
O
OH
HO
HO
O
O
OH
22a
f1 (ppm)
f2 (ppm)

MeOD, 400.13 MHz

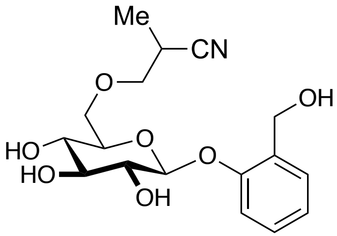

22b

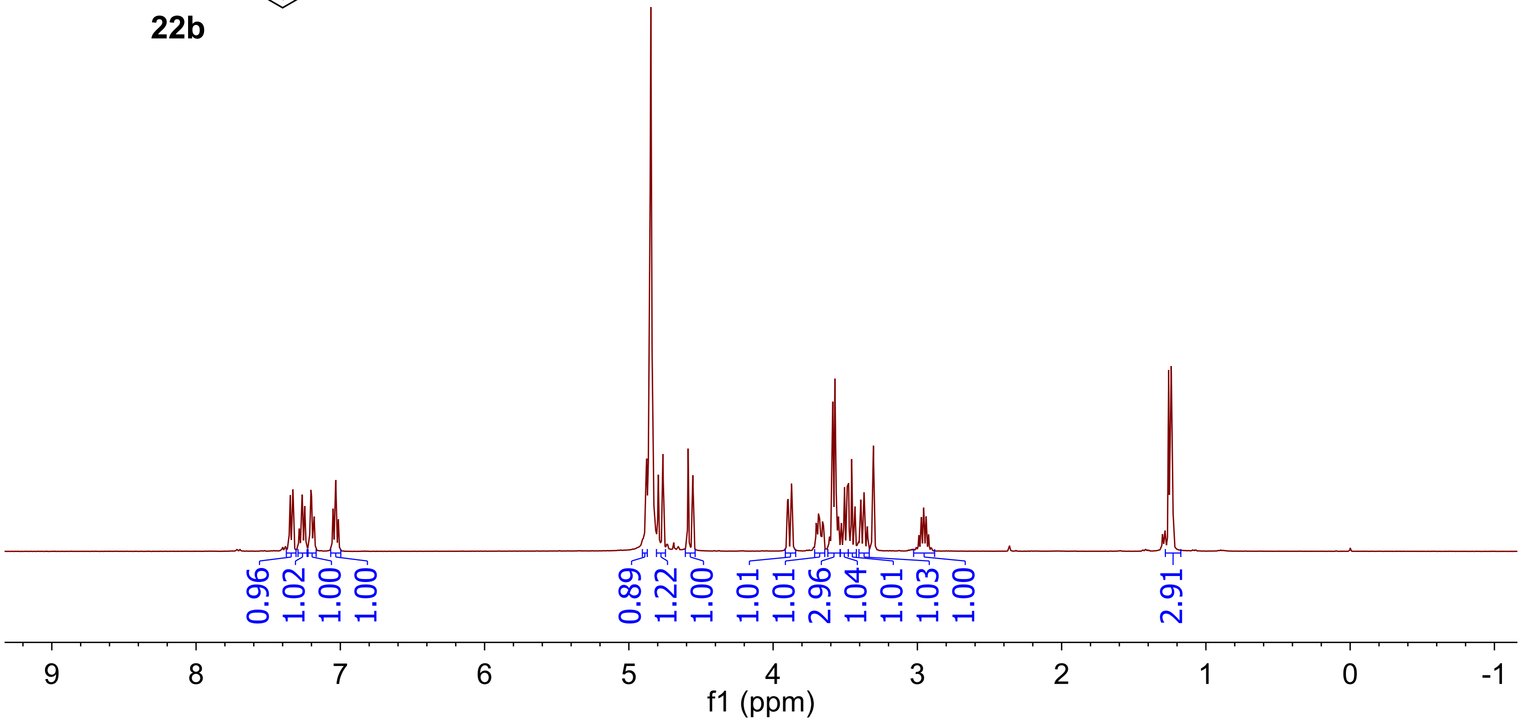

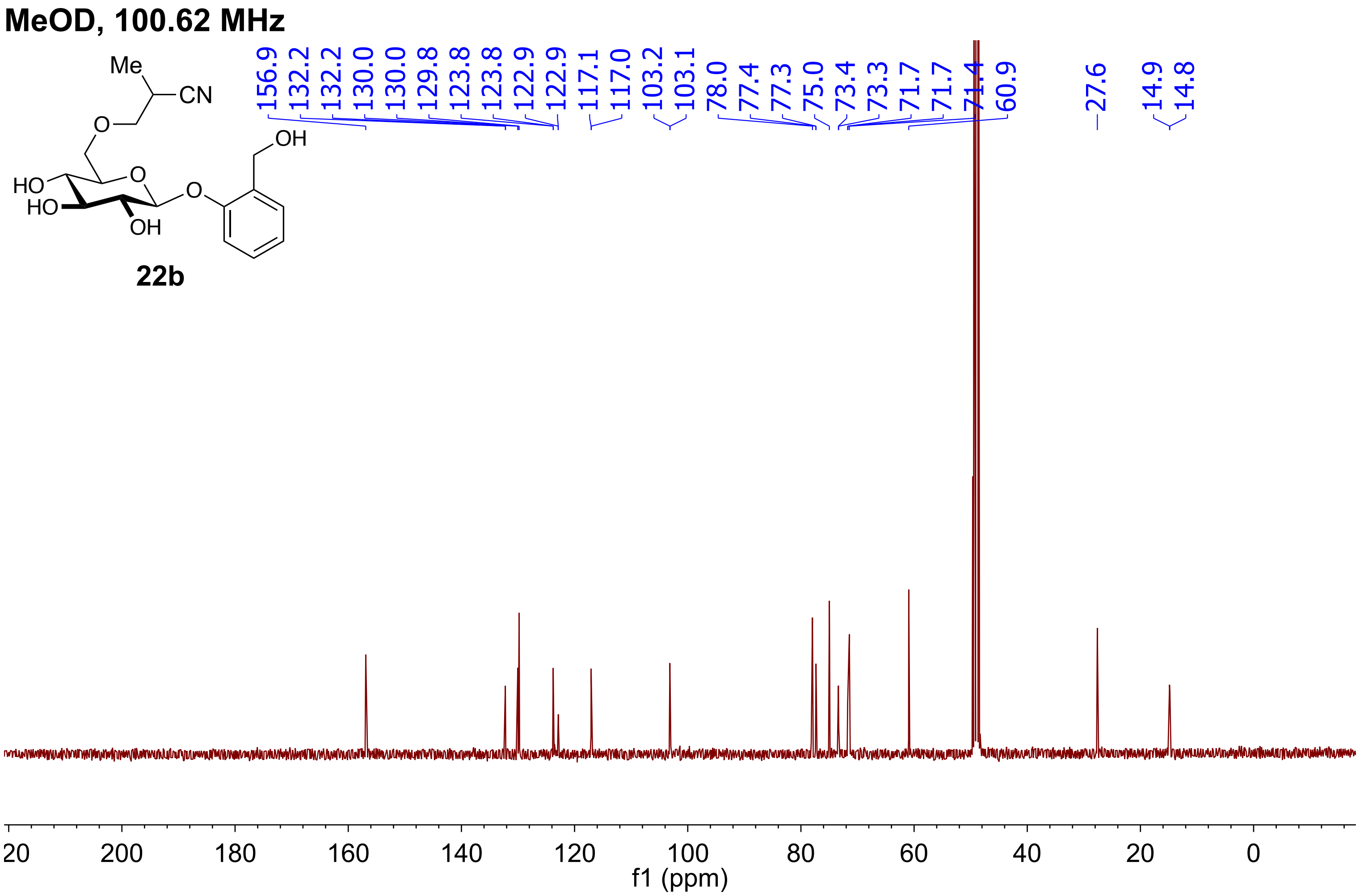
MeOD, 100.62 MHz
Me
CN
O
OH
HO
HO
O
O
OH
22b
156.9
132.2
132.2
130.0
130.0
129.8
123.8
123.8
122.9
122.9
117.1
117.0
103.2
103.1
78.0
77.4
77.3
75.0
73.4
73.3
71.7
71.7
71.4
60.9
27.6
14.9
14.8
20
200
180
160
140
120
100
80
60
40
20
0
f1 (ppm)

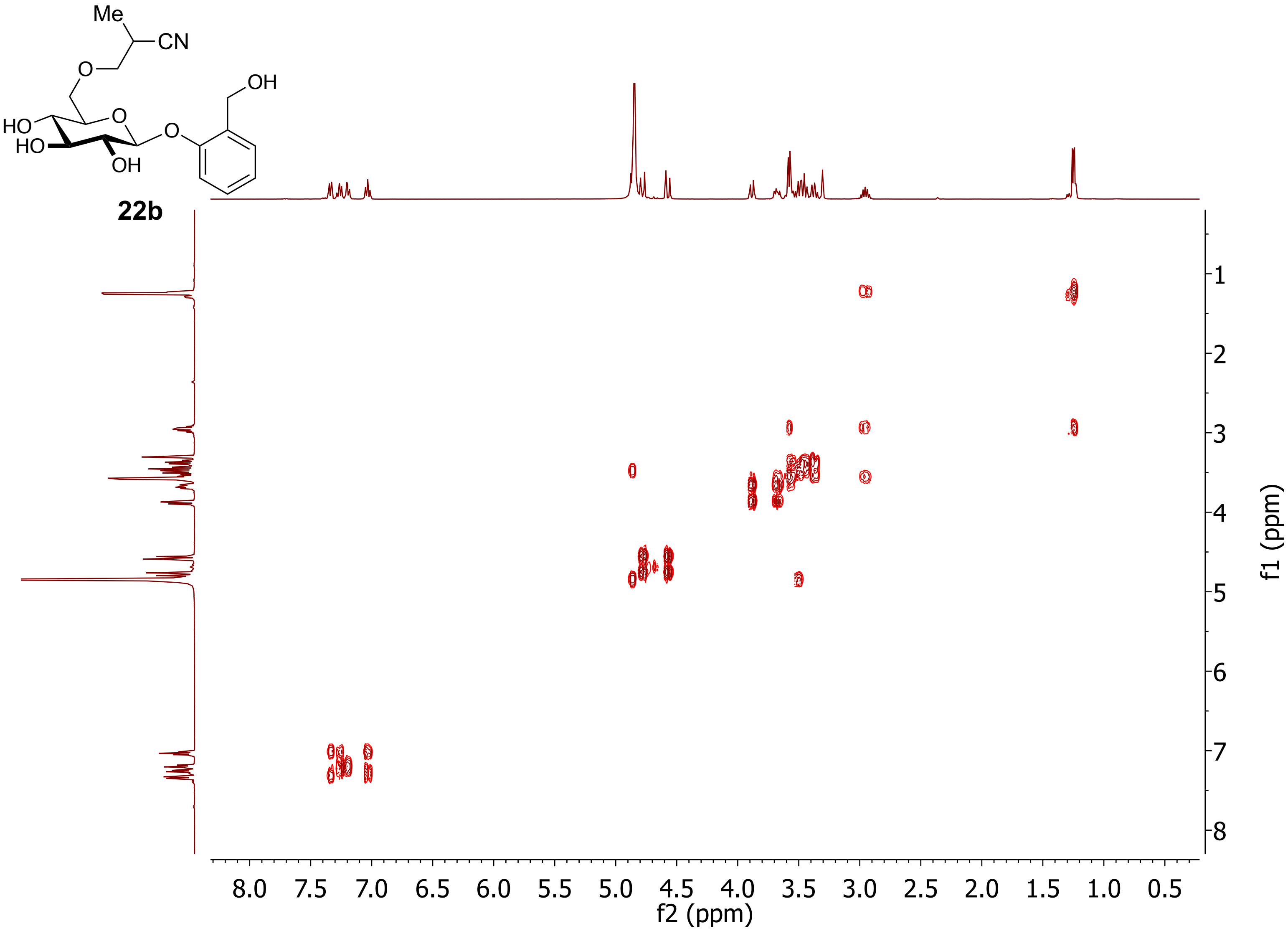
Me
CN
O
OH
HO
HO
O
O
OH
8.0
7.5
7.0
6.5
6.0
5.5
5.0
4.5
4.0
3.5
3.0
2.5
2.0
1.5
1.0
0.5
f2 (ppm)
1
2
3
4
5
6
7
8
f1 (ppm)

22b

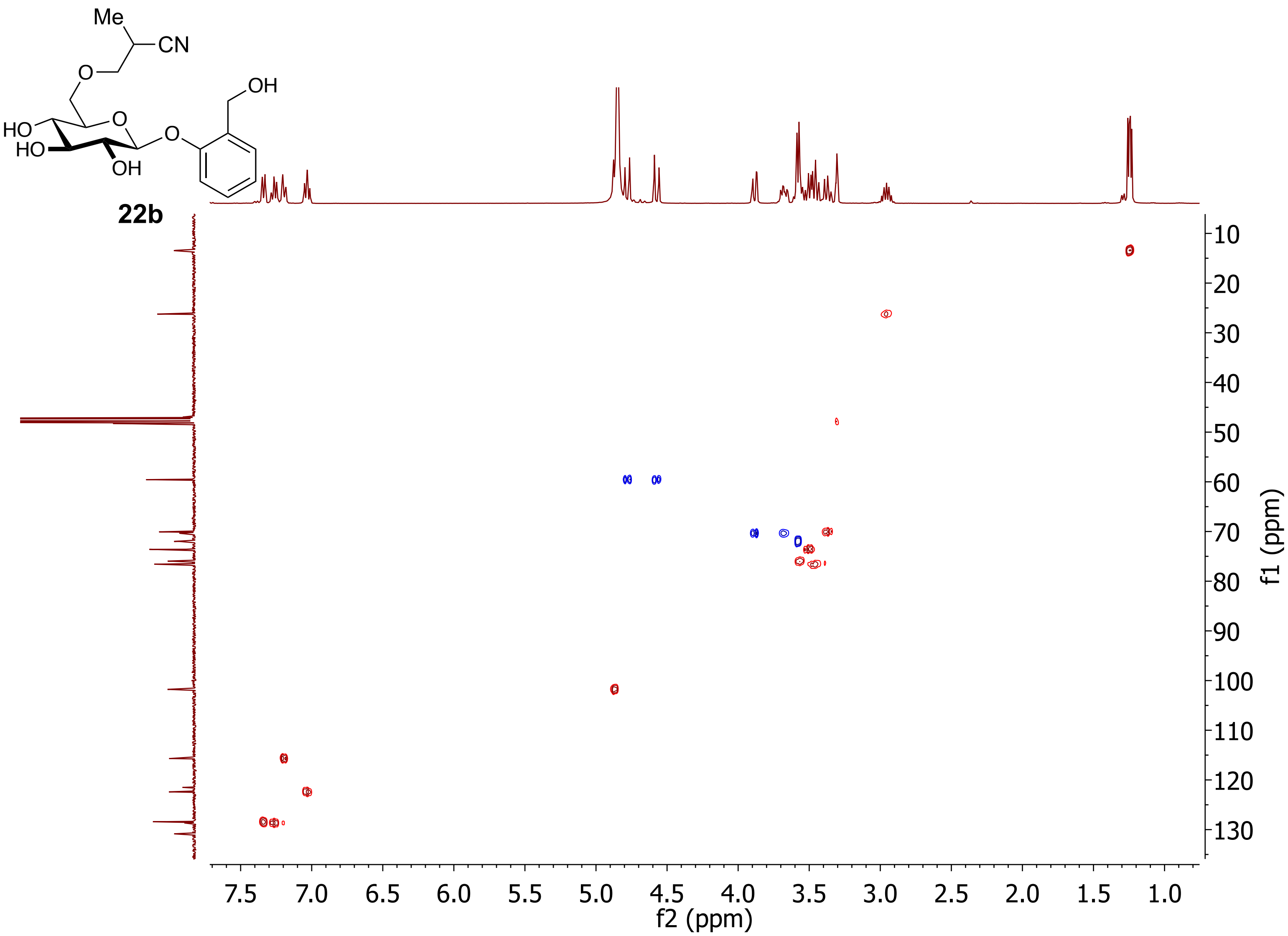
Me
CN
O
OH
HO
HO
O
O
OH
22b
7.5
7.0
6.5
6.0
5.5
5.0
4.5
4.0
3.5
3.0
2.5
2.0
1.5
1.0
f2 (ppm)
10
20
30
40
50
60
70
80
90
100
110
120
130
f1 (ppm)

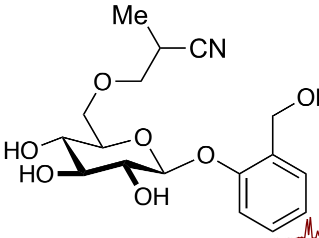
Me
CN
O
OH
HO
O
HO
O
OH

22b

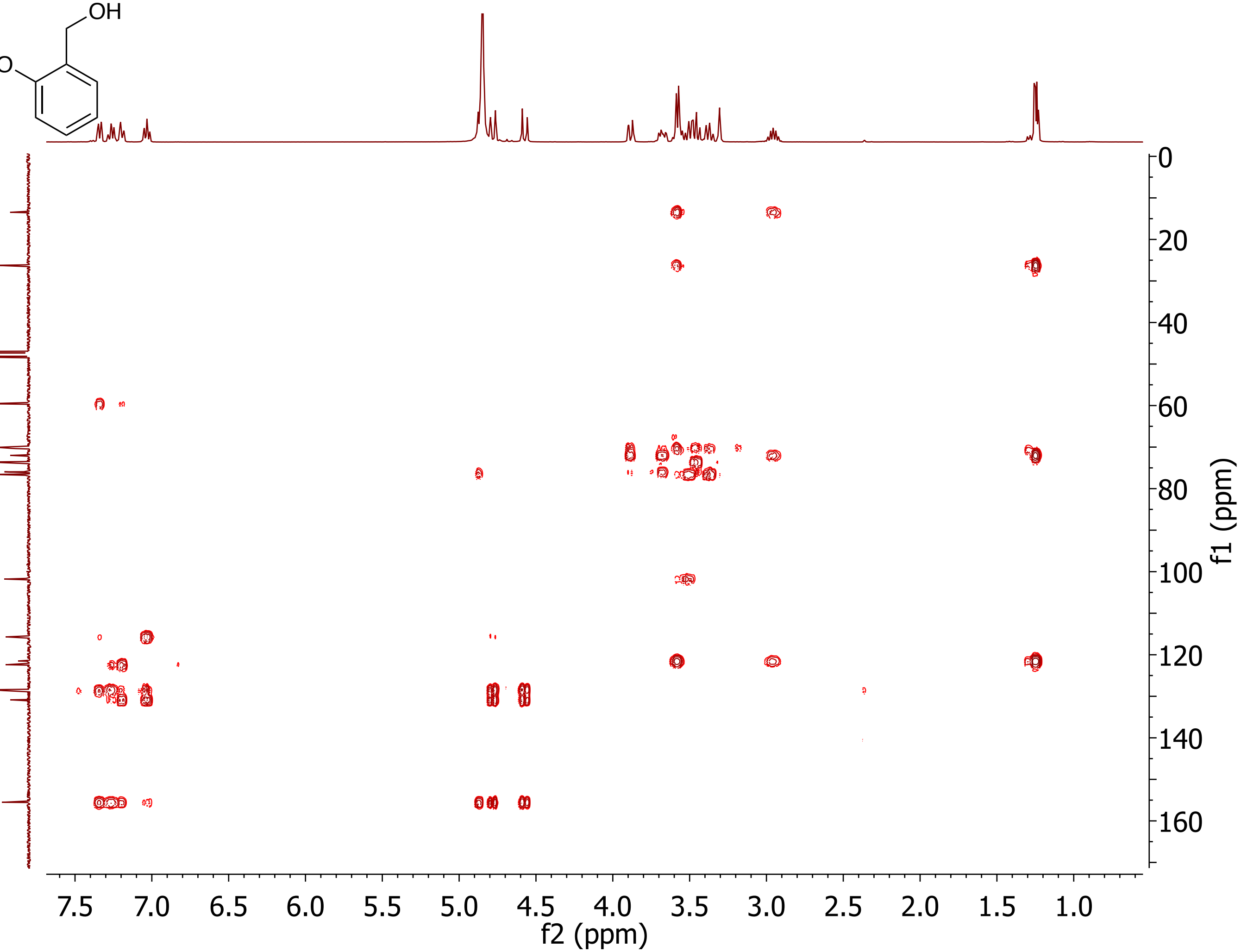
0
20
40
60
80
100
120
140
160
f1 (ppm)
7.5
7.0
6.5
6.0
5.5
5.0
4.5
4.0
3.5
3.0
2.5
2.0
1.5
1.0
f2 (ppm)

MeOD, 400.13 MHz

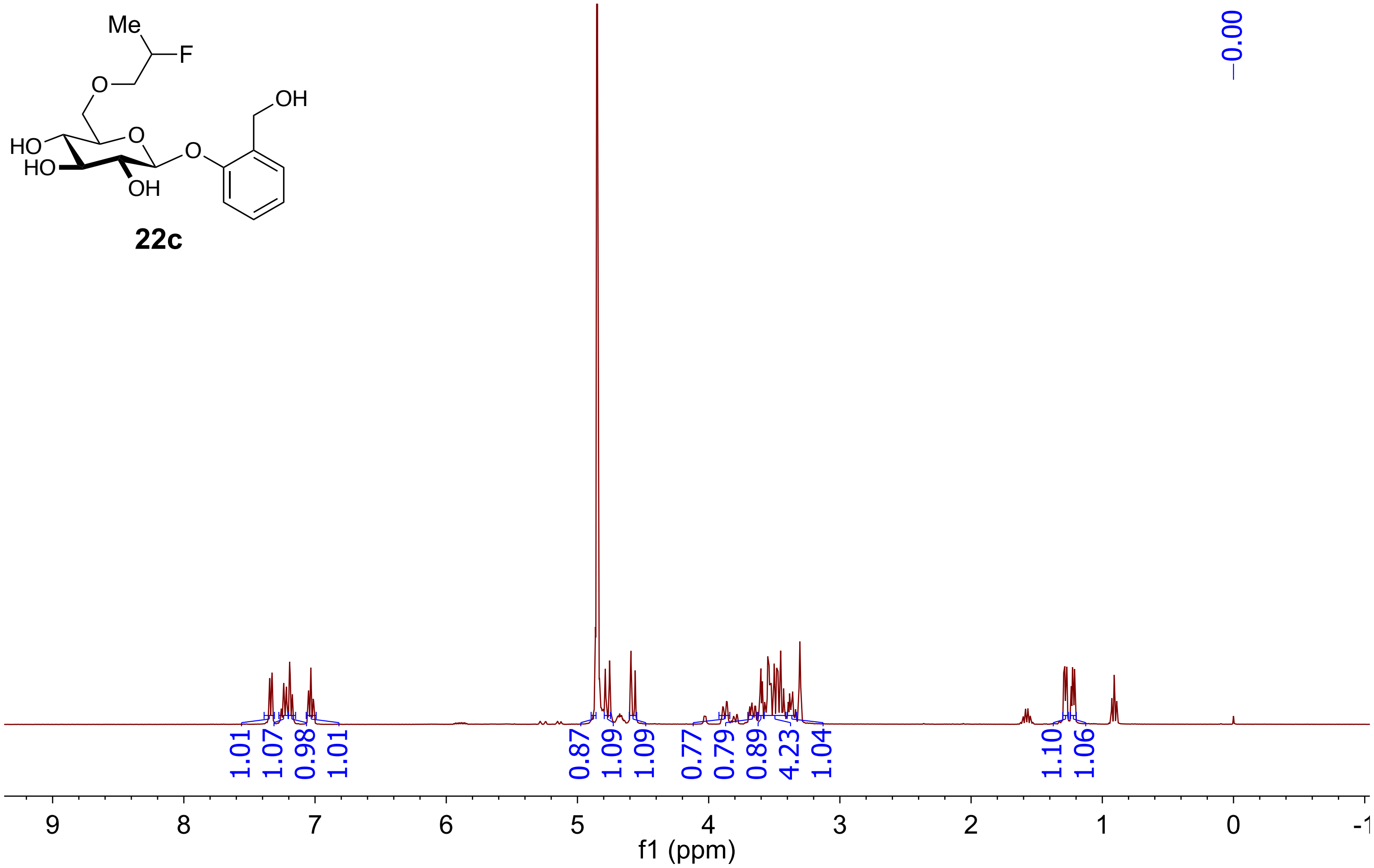

MeOD, 100.62 MHz

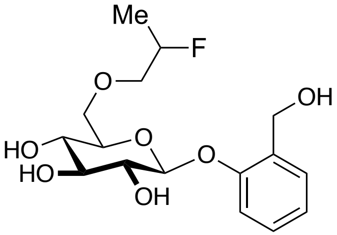

22c

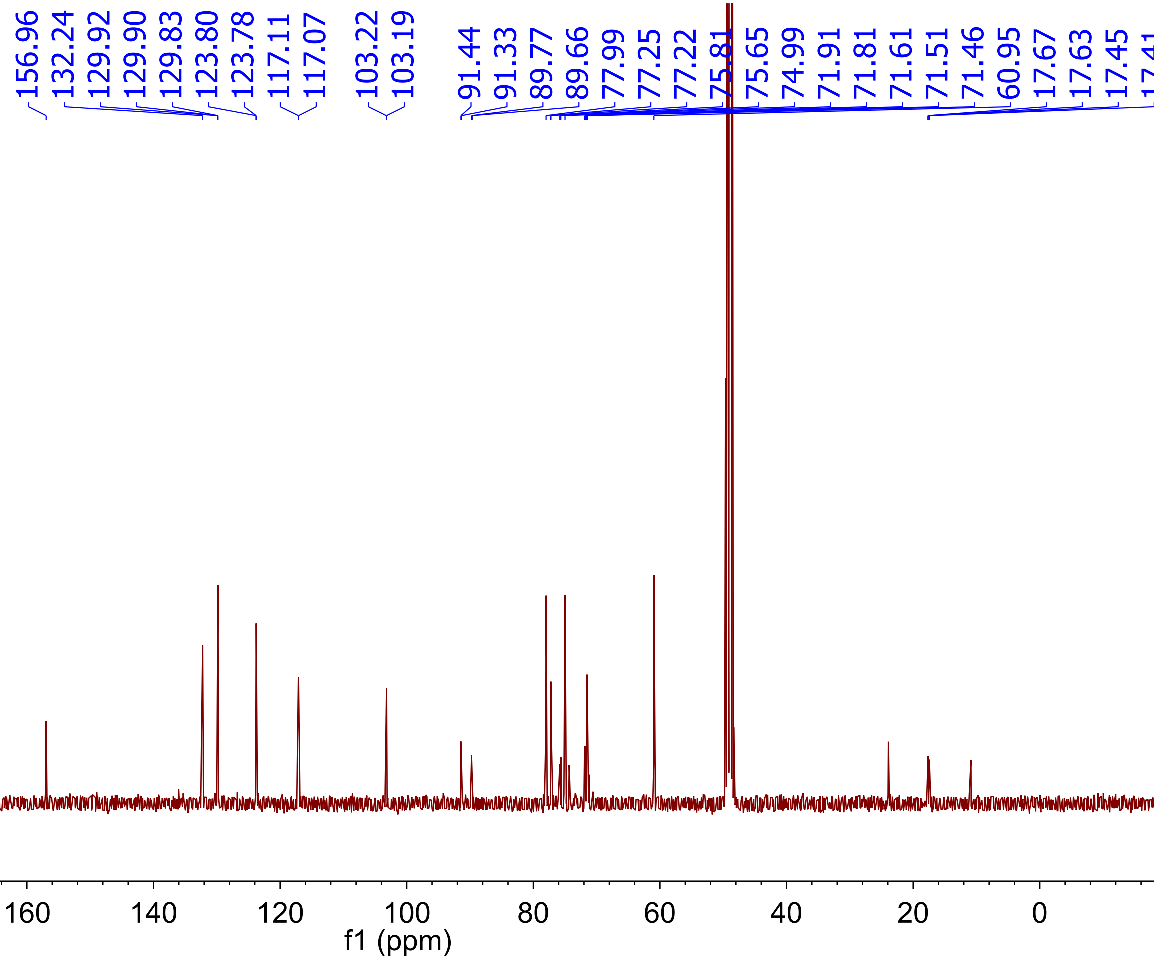

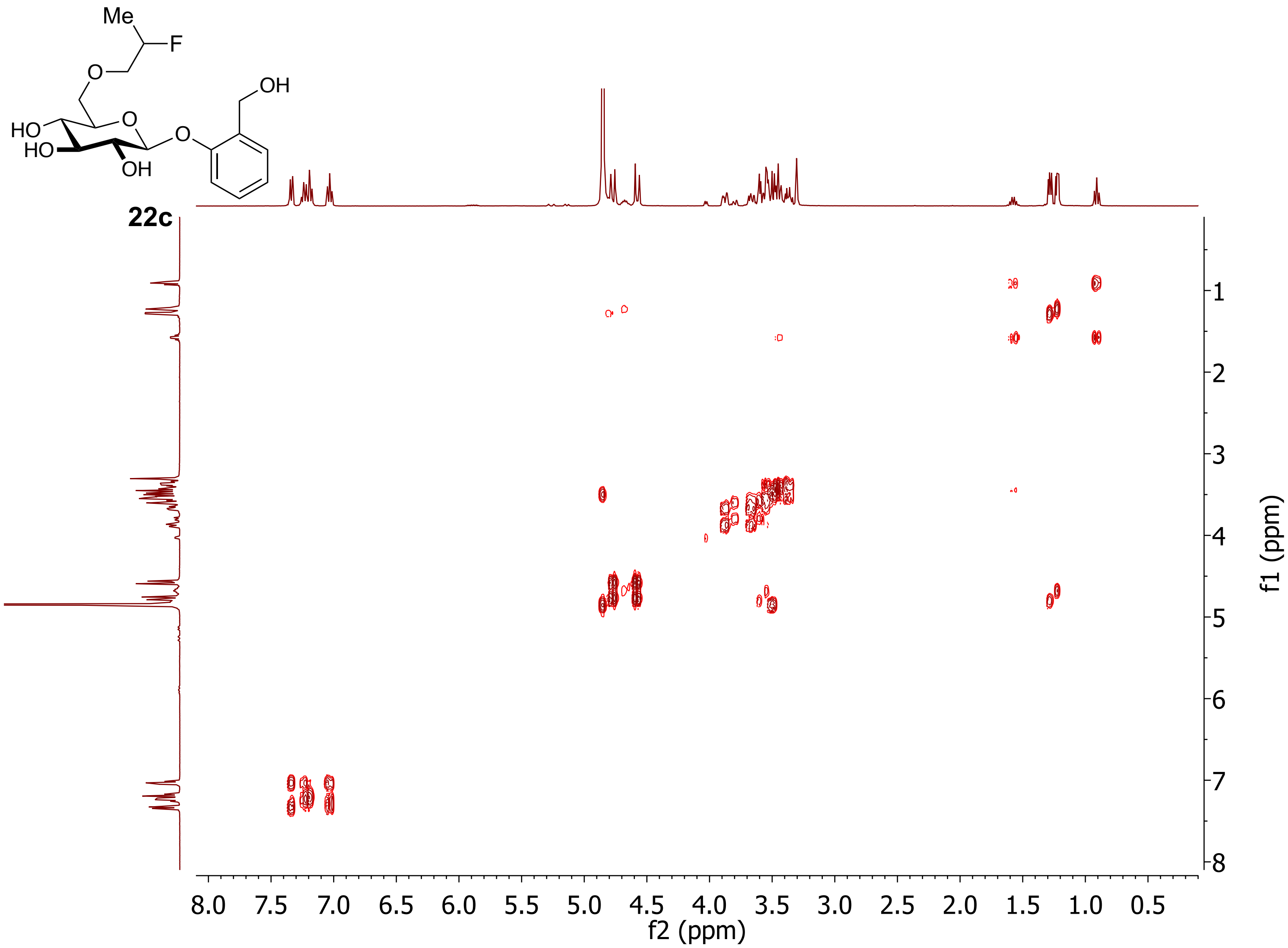

Me
F
O
OH
HO
HO
O
O
OH
22c
f1 (ppm)
f2 (ppm)
8.0
7.5
7.0
6.5
6.0
5.5
5.0
4.5
4.0
3.5
3.0
2.5
2.0
1.5
1.0
0.5
1
2
3
4
5
6
7
8

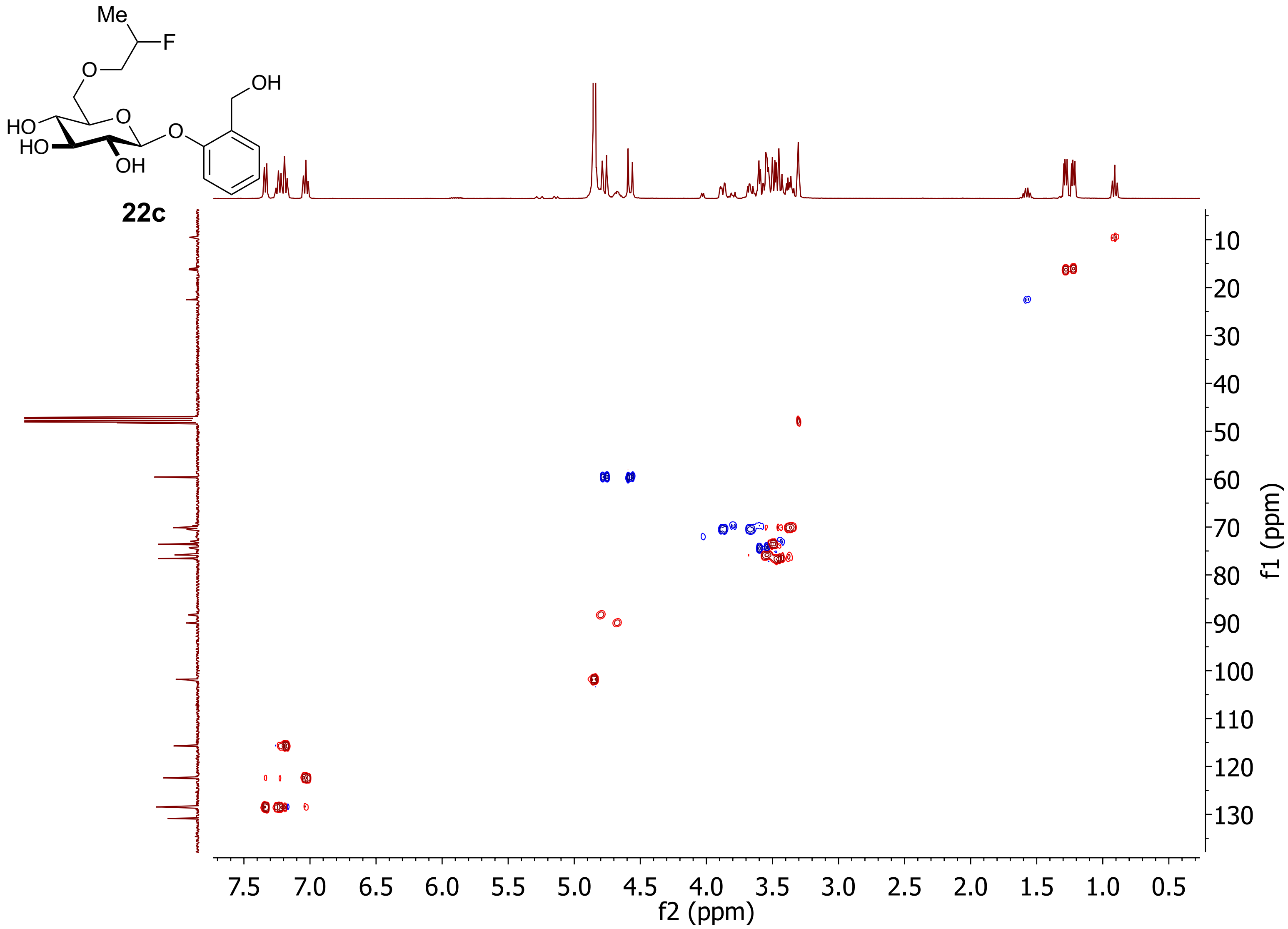

22c

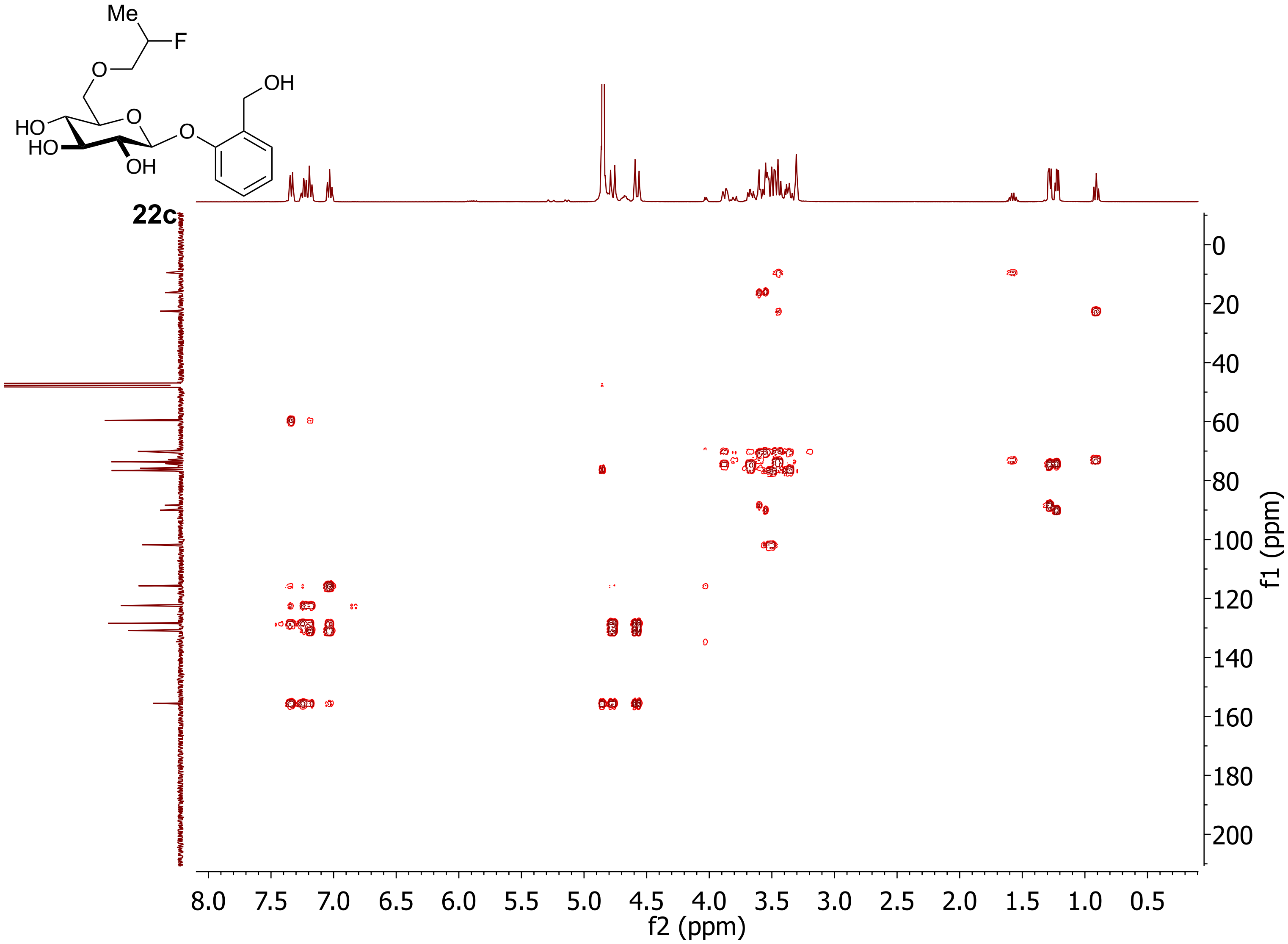
Me
F
O
OH
HO
O
O
HO
OH
22c
0
20
40
60
80
100
120
140
160
180
200
f1 (ppm)
8.0 7.5 7.0 6.5 6.0 5.5 5.0 4.5 4.0 3.5 3.0 2.5 2.0 1.5 1.0 0.5
f2 (ppm)

MeOD, 376.46 MHz

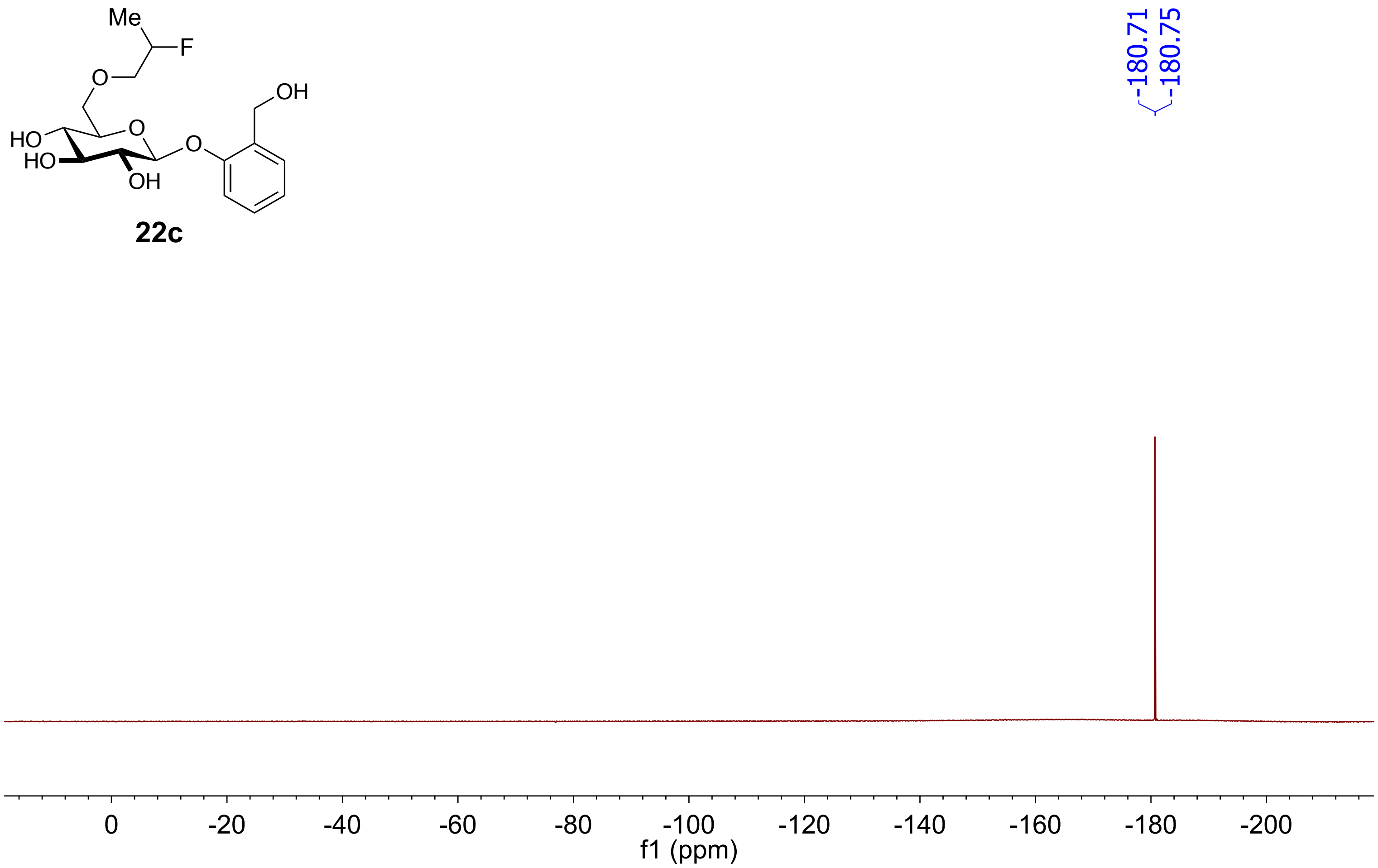

MeOD, 400.13 MHz

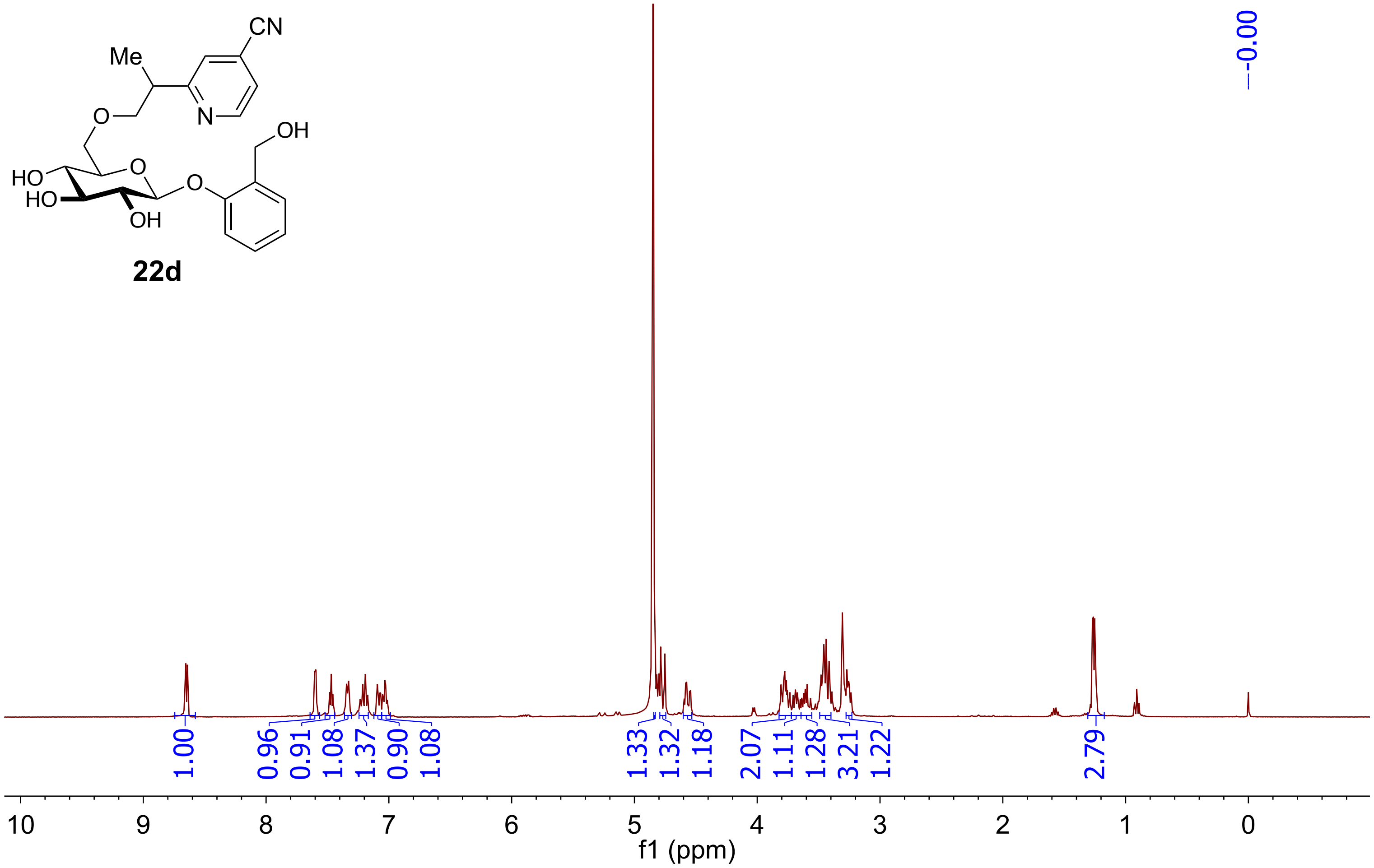

MeOD, 100.62 MHz

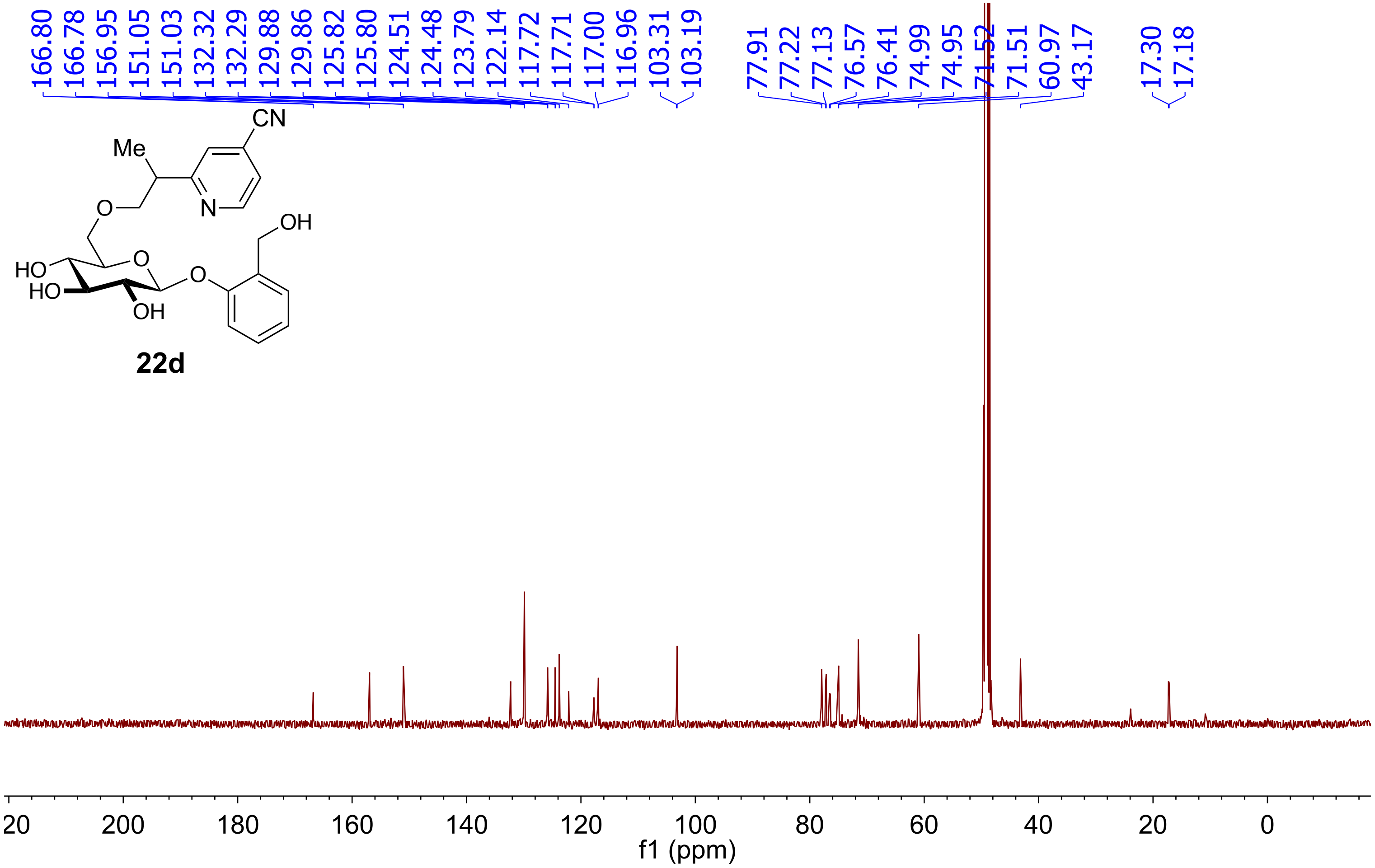

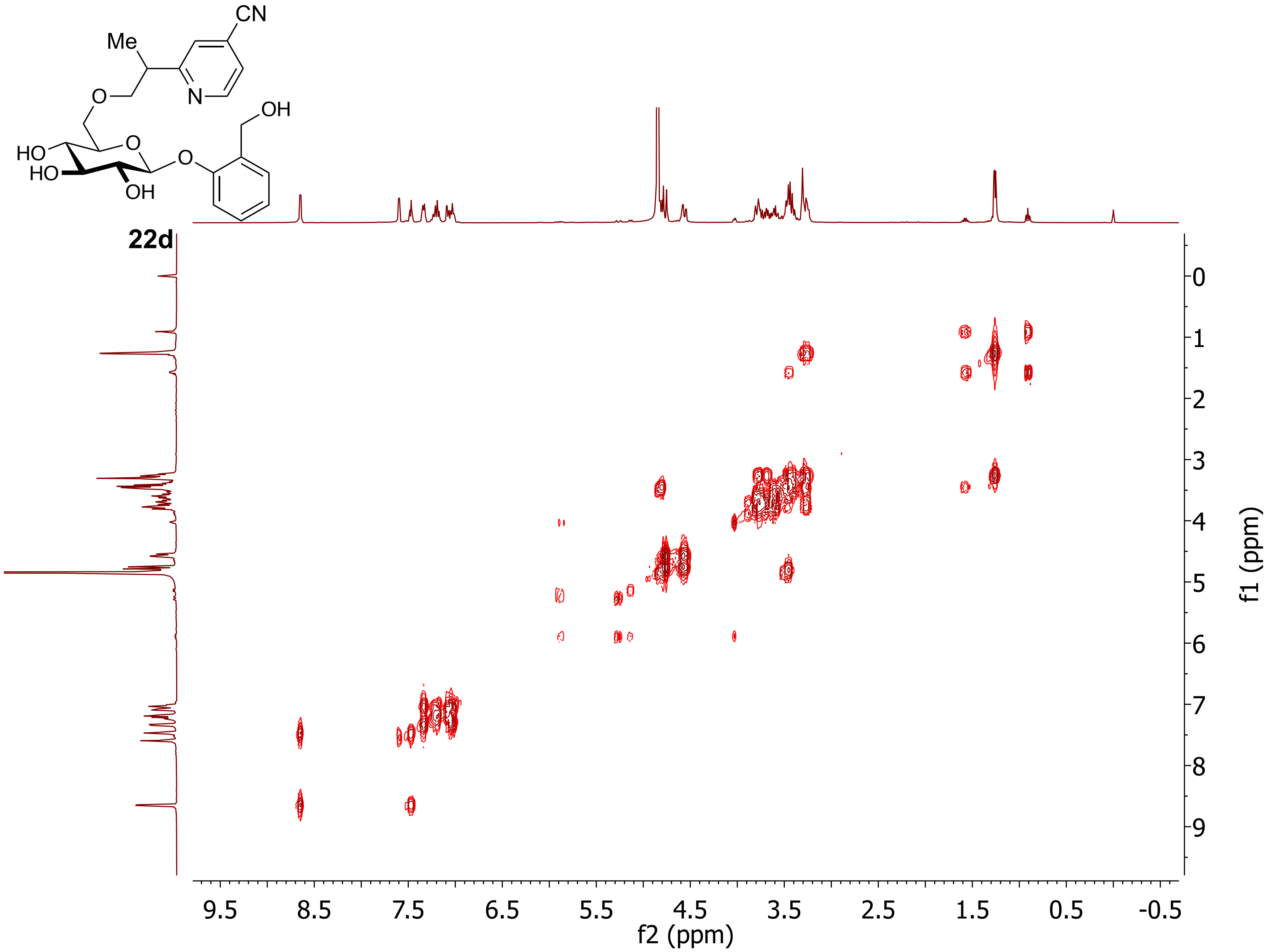
CN
Me
N
O
OH
HO
O
HO
O
OH
22d
9.5
8.5
7.5
6.5
5.5
4.5
3.5
2.5
1.5
0.5
-0.5
f2 (ppm)
0
1
2
3
4
5
6
7
8
9
f1 (ppm)

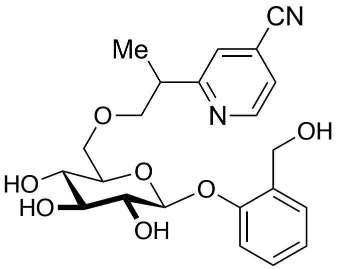

CN
Me
N
O
OH
HO
HO
O
O
OH

22d

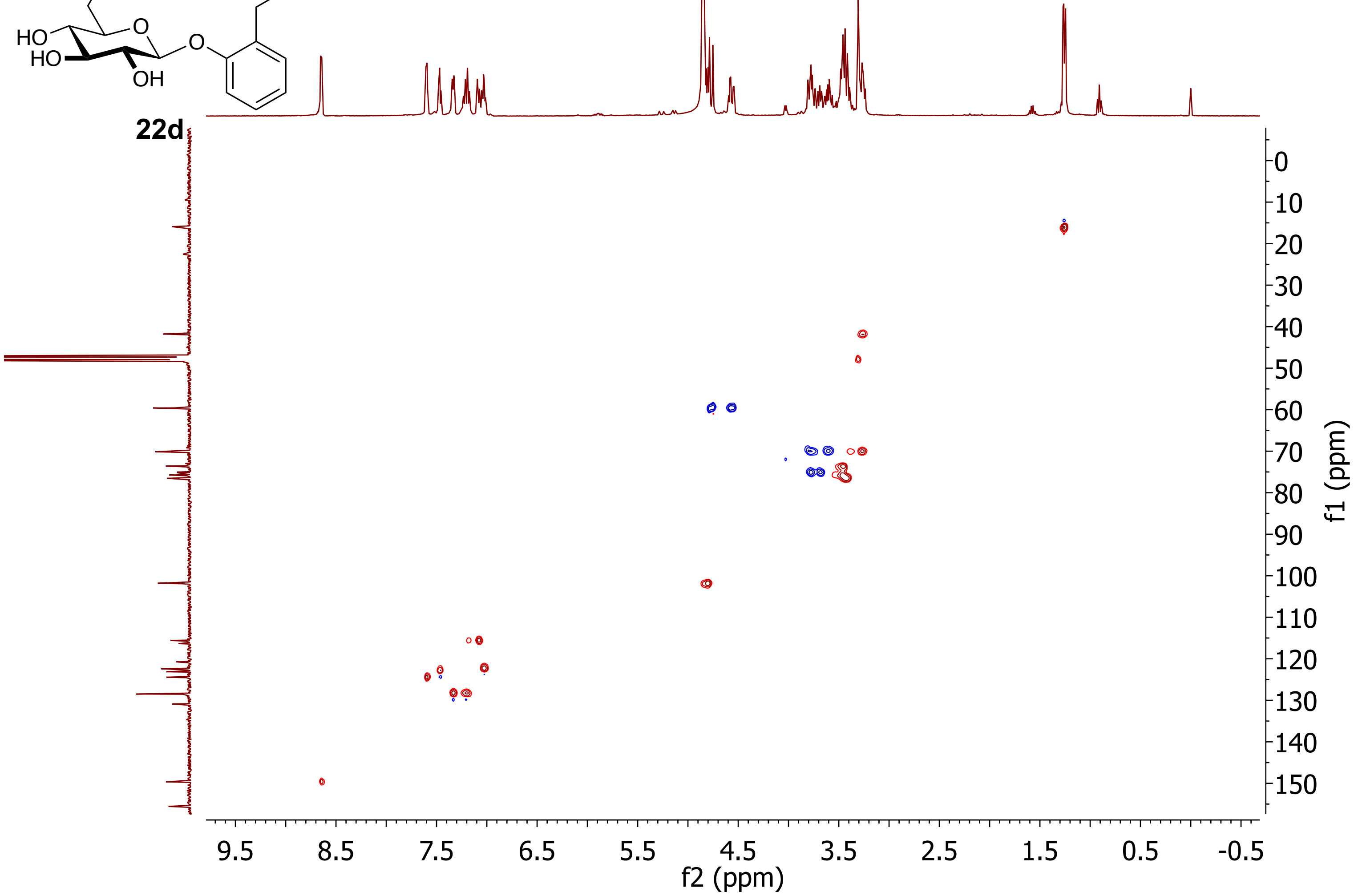

f2 (ppm)
f1 (ppm)
9.5
8.5
7.5
6.5
5.5
4.5
3.5
2.5
1.5
0.5
-0.5
0
10
20
30
40
50
60
70
80
90
100
110
120
130
140
150

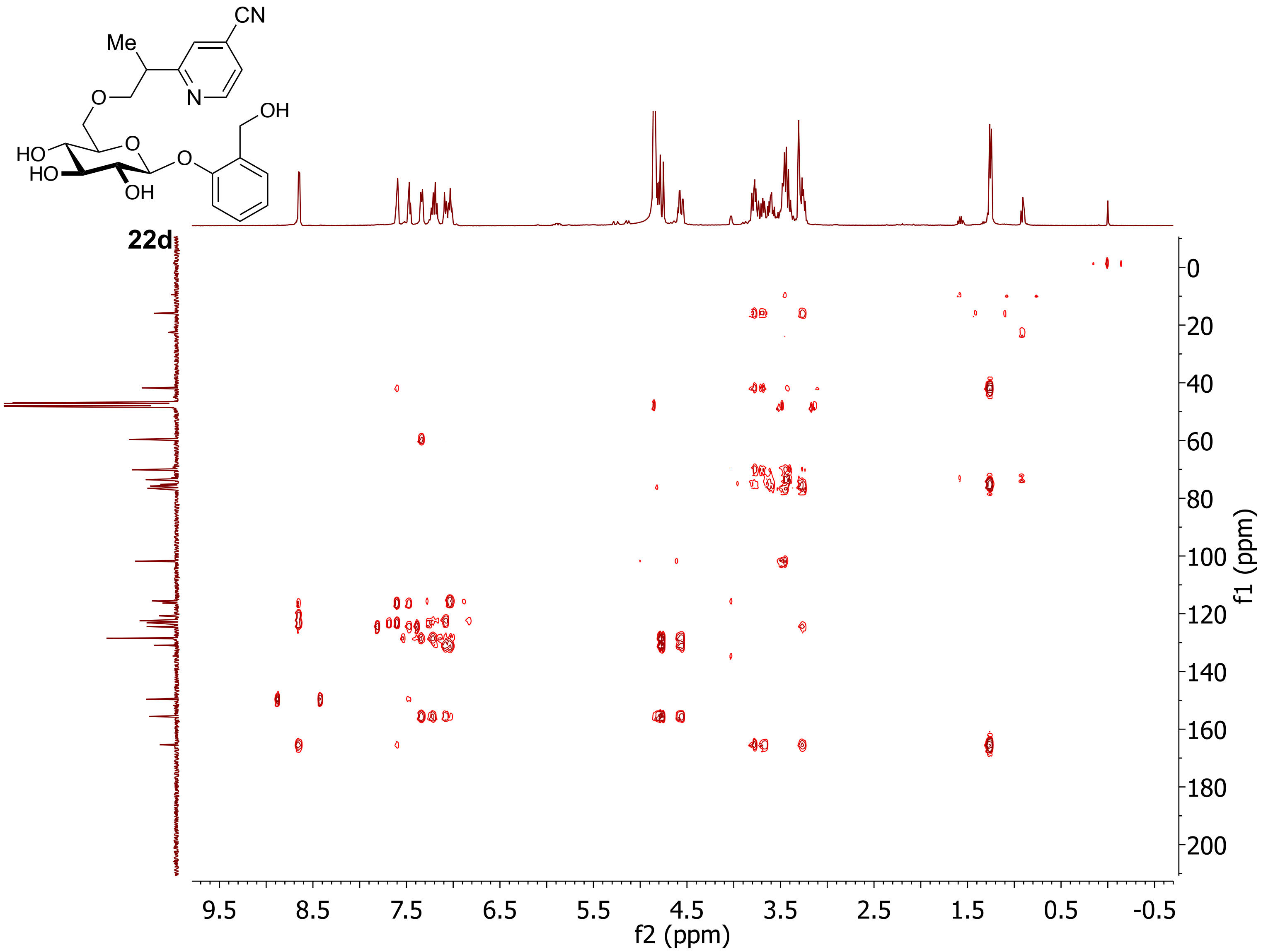
CN
Me
N
O
OH
HO
O
O
HO
OH
0
20
40
60
80
100
120
140
160
180
200
f1 (ppm)
9.5
8.5
7.5
6.5
5.5
4.5
3.5
2.5
1.5
0.5
-0.5
f2 (ppm)

22d

MeOD, 400.13 MHz

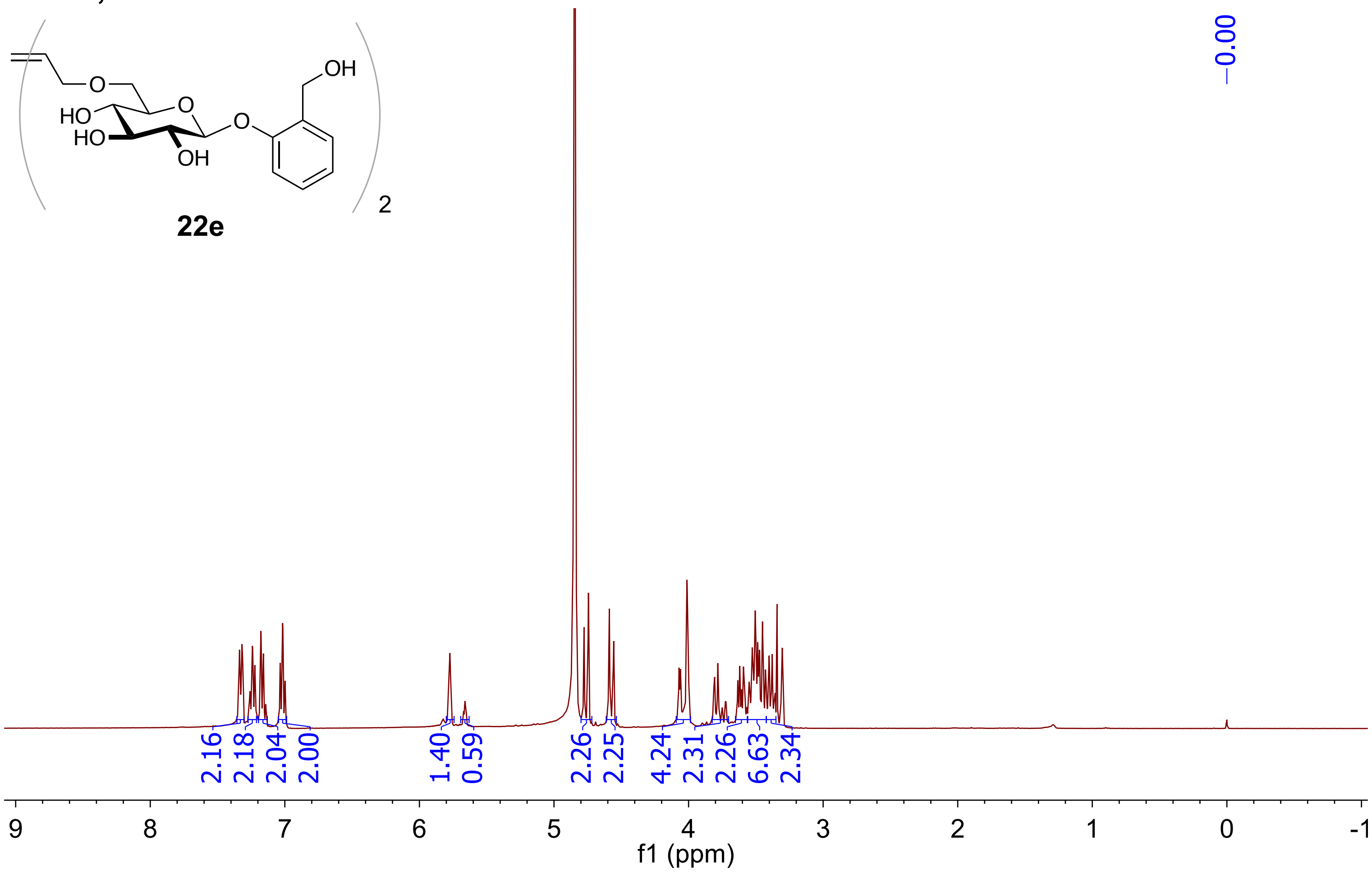

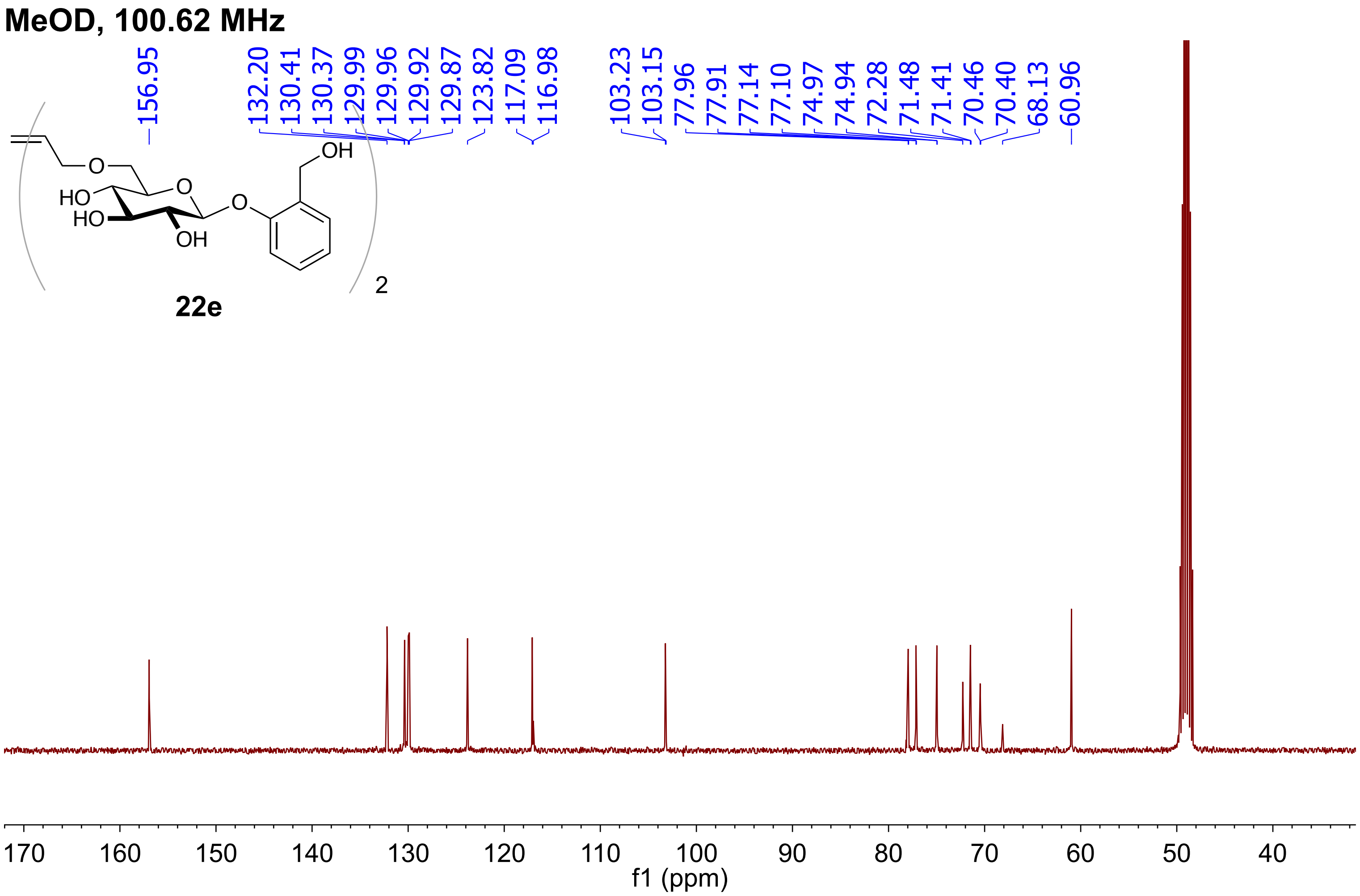
MeOD, 100.62 MHz
156.95
132.20
130.41
130.37
129.99
129.96
129.92
129.87
123.82
117.09
116.98
103.23
103.15
77.96
77.91
77.14
77.10
74.97
74.94
72.28
71.48
71.41
70.46
70.40
68.13
60.96
HO
HO
OH
O
O
O
OH
2
22e
170
160
150
140
130
120
110
100
90
80
70
60
50
40
f1 (ppm)

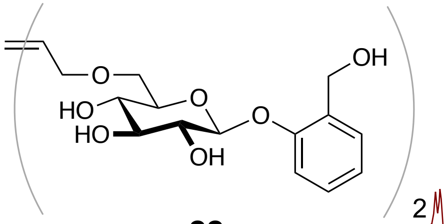
OH
O
HO
HO
OH
O
O
2

22e

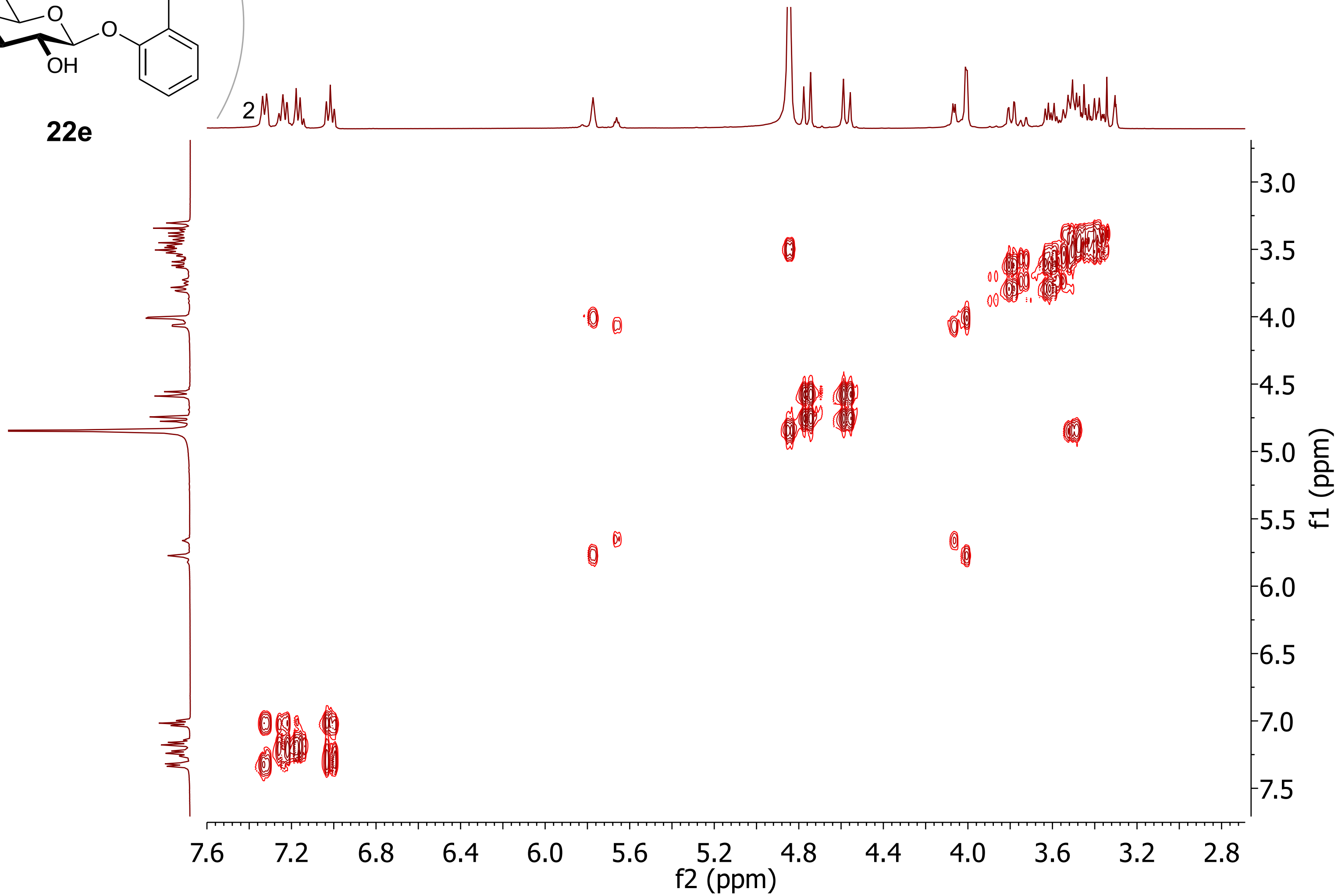
f2 (ppm)
f1 (ppm)
7.6
7.2
6.8
6.4
6.0
5.6
5.2
4.8
4.4
4.0
3.6
3.2
2.8
3.0
3.5
4.0
4.5
5.0
5.5
6.0
6.5
7.0
7.5

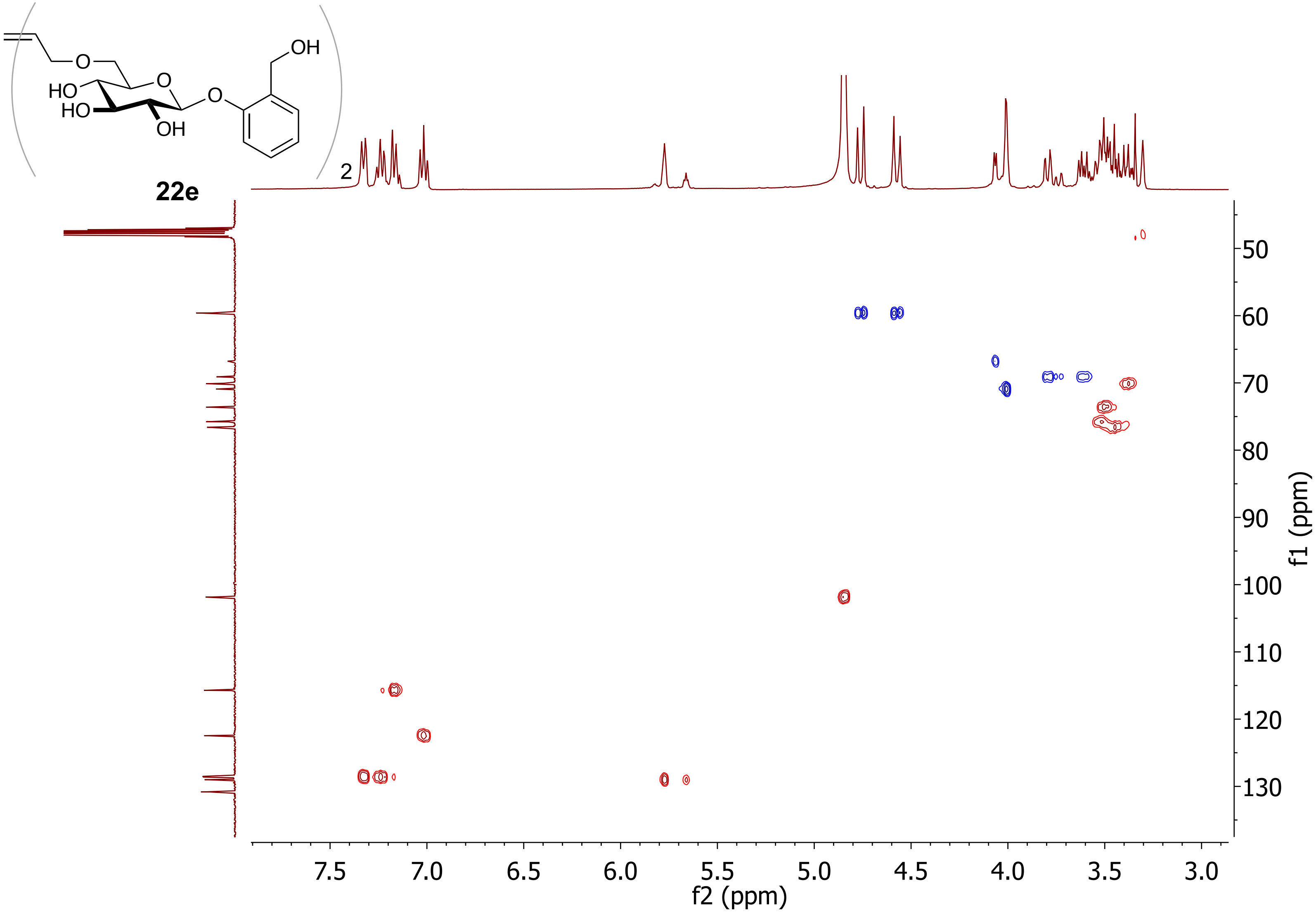
OH
O
O
O
HO
HO
OH
2
7.5
7.0
6.5
6.0
5.5
5.0
4.5
4.0
3.5
3.0
f2 (ppm)
50
60
70
80
90
100
110
120
130
f1 (ppm)

22e

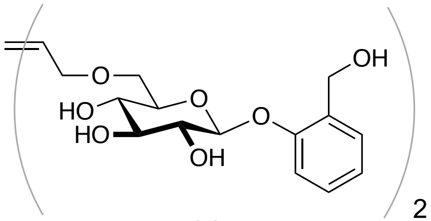

OH
O
O
HO
HO
OH
O
2

22e

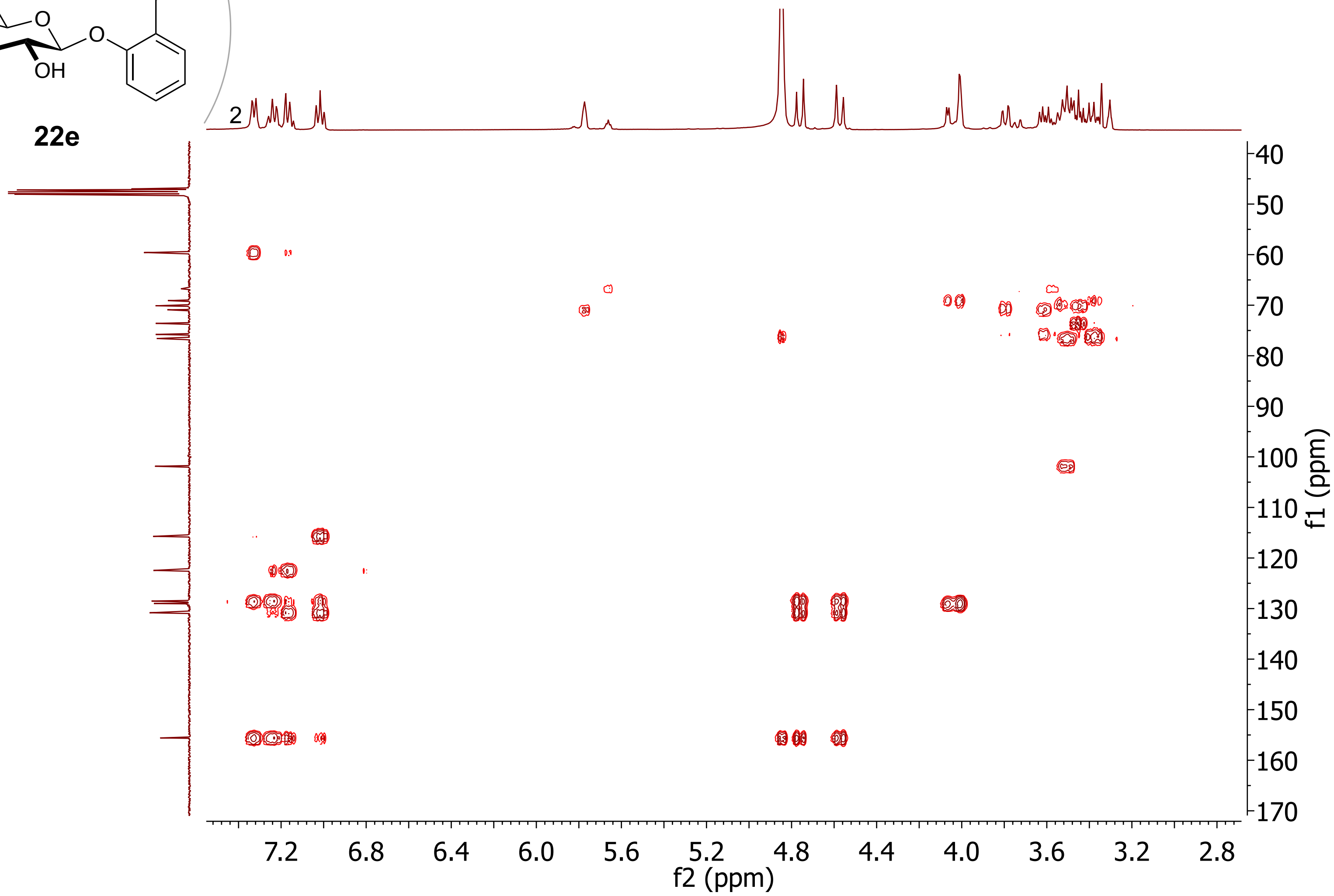

40
50
60
70
80
90
100
110
120
130
140
150
160
170
f1 (ppm)
7.2
6.8
6.4
6.0
5.6
5.2
4.8
4.4
4.0
3.6
3.2
2.8
f2 (ppm)

MeOD, 400.13 MHz

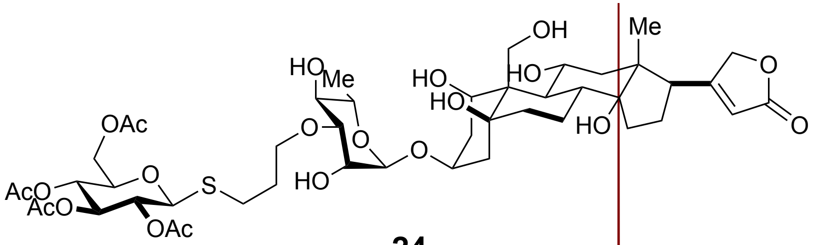

24

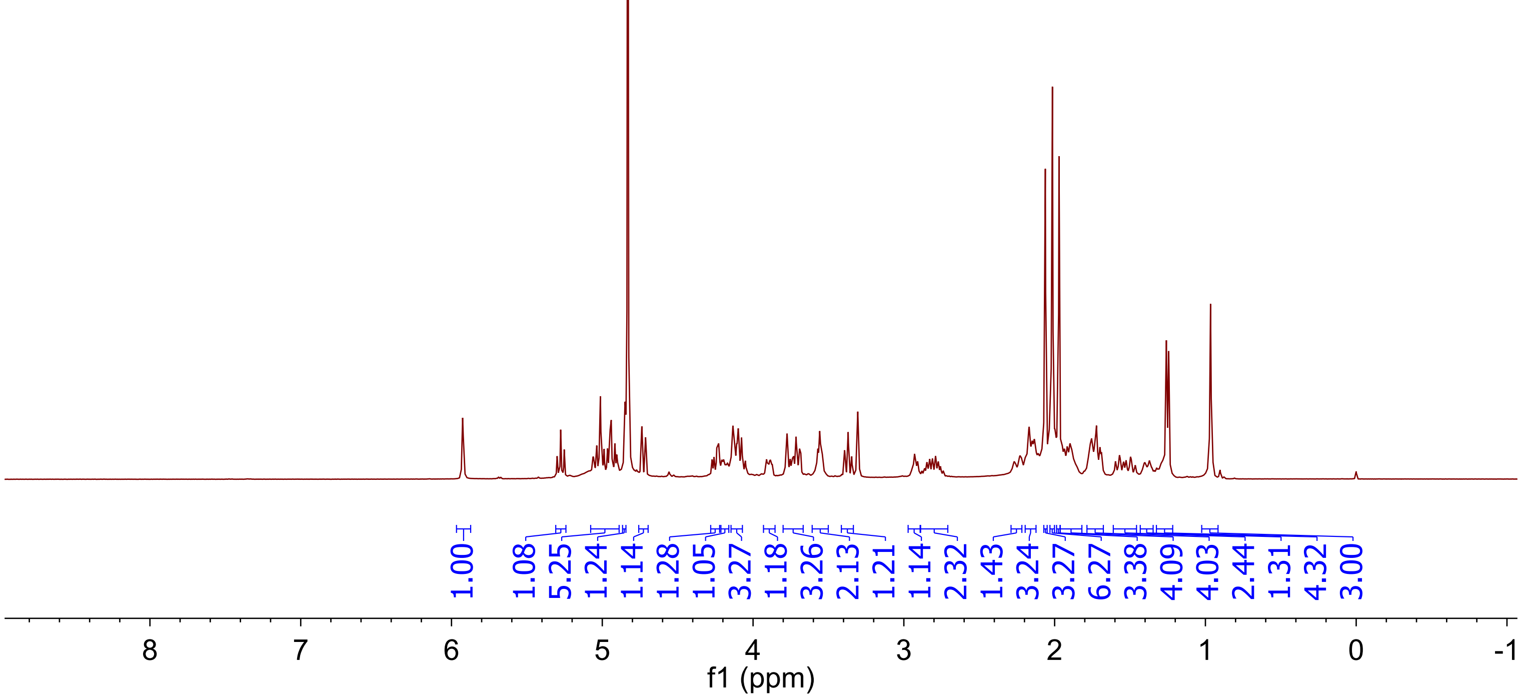

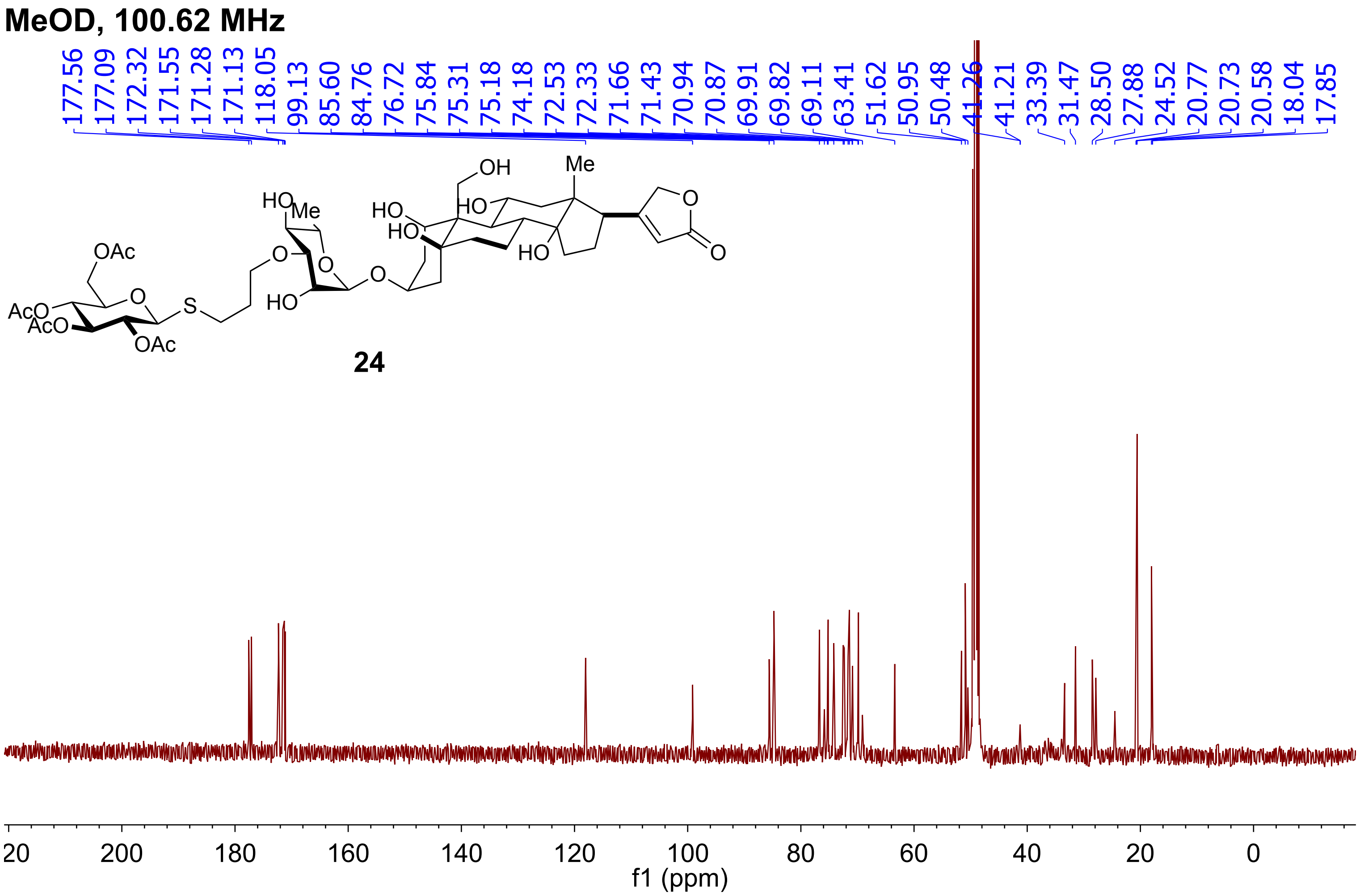

MeOD, 100.62 MHz
177.56
177.09
172.32
171.55
171.28
171.13
118.05
99.13
85.60
84.76
76.72
75.84
75.31
75.18
74.18
72.53
72.33
71.66
71.43
70.94
70.87
69.91
69.82
69.11
63.41
51.62
50.95
50.48
41.26
41.21
33.39
31.47
28.50
27.88
24.52
20.77
20.73
20.58
18.04
17.85
OAc
AcO
AcO
O
OAc
S
O
HO
Me
O
HO
O
OH
Me
HO
HO
HO
HO
O
O
24
f1 (ppm)

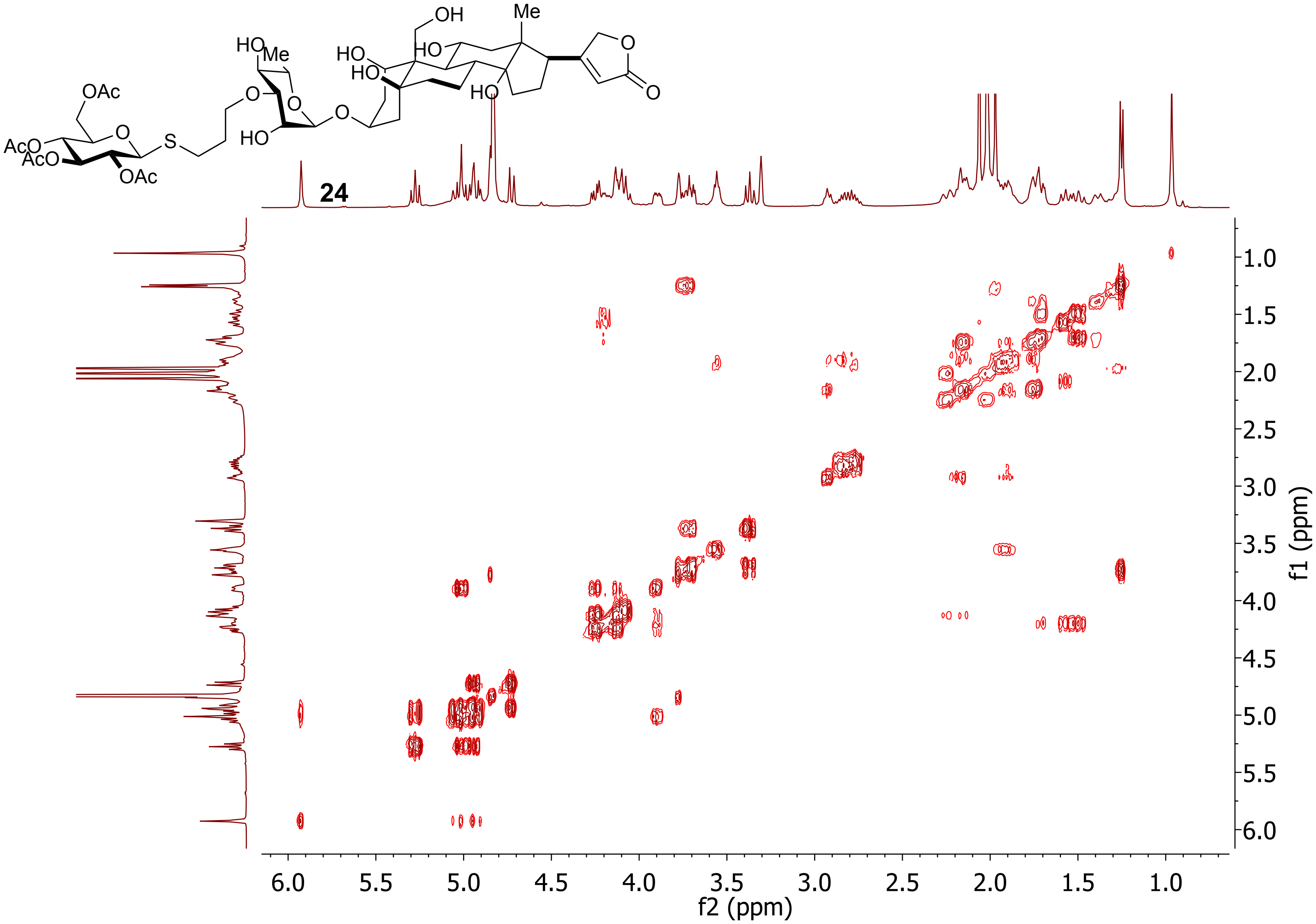
OAc
AcO
AcO
OAc
O
S
O
HO
Me
O
HO
O
OH
HO
HO
HO
Me
HO
O
O
24
6.0
5.5
5.0
4.5
4.0
3.5
3.0
2.5
2.0
1.5
1.0
f2 (ppm)
f1 (ppm)

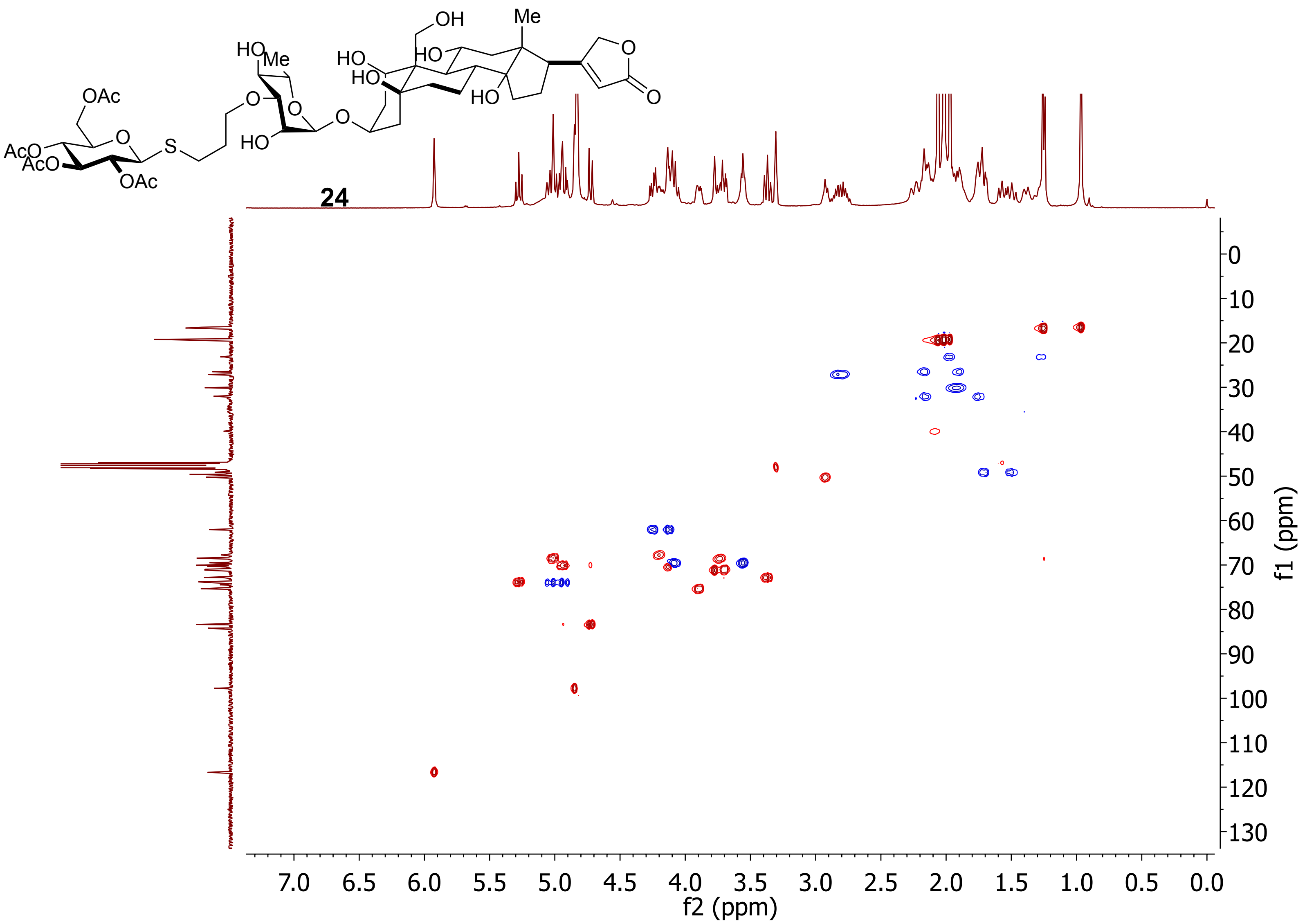
24
f2 (ppm)
f1 (ppm)

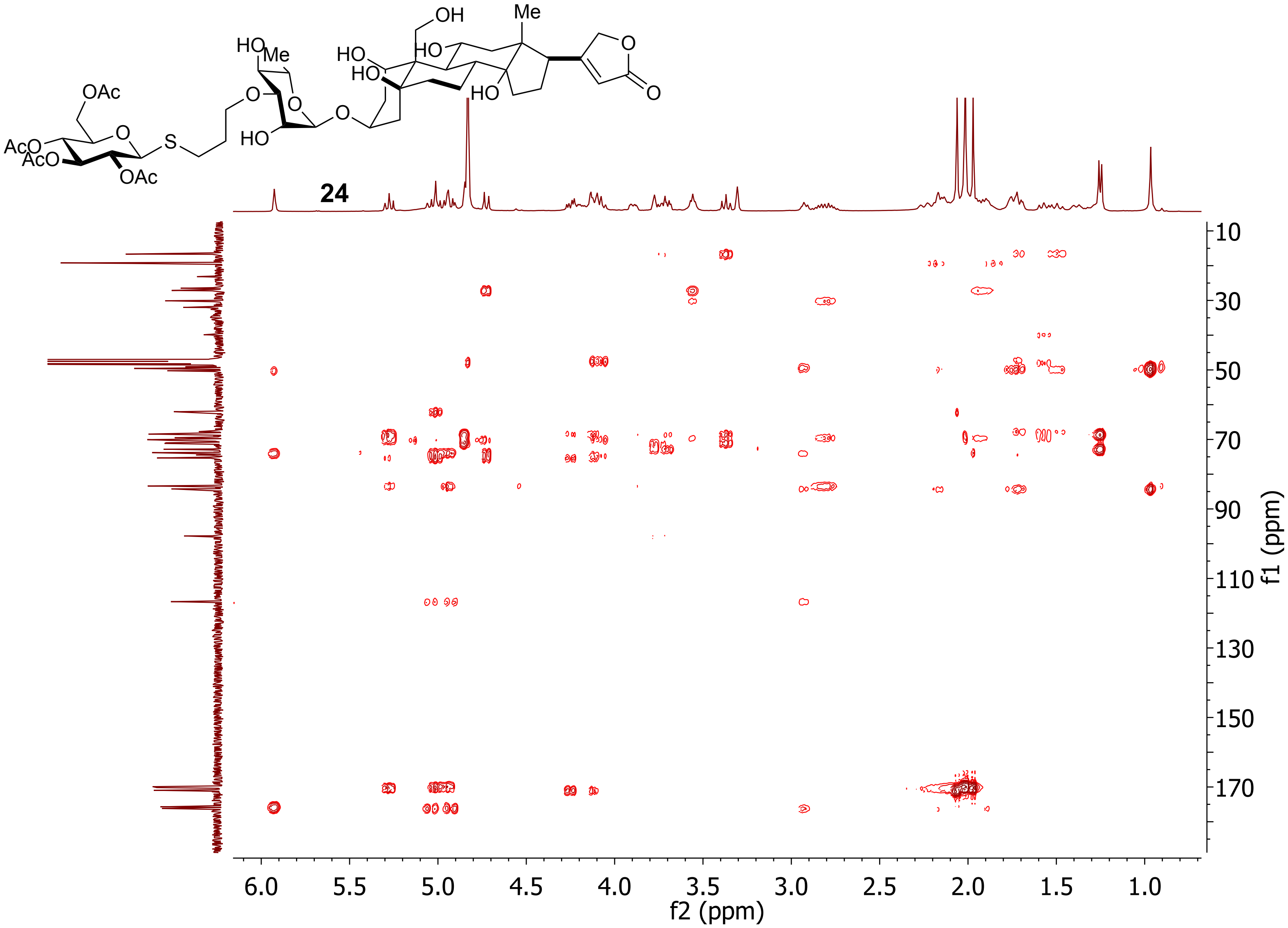

OAc
AcO
AcO
O
OAc
S
O
HO
Me
O
HO
O
OH
HO
HO
HO
Me
O
O
24
f1 (ppm)
10
30
50
70
90
110
130
150
170
6.0
5.5
5.0
4.5
4.0
3.5
3.0
2.5
2.0
1.5
1.0
f2 (ppm)

Supplementary References:

1. Kraus, J. M., Gits, H. C. & Silverman, R. B. High yielding allylation of a chiral secondary alcohol containing base sensitive functional groups. *Tetrahedron Lett.* **53**, 1319–1322 (2012).
2. Chung, M.-K., Orlova, G., Goddard, J. D., Schlaf, M., Harris, R., Beveridge, T. J., White, G. & Hallett, F. R. Regioselective Silylation of Sugars through Palladium Nanoparticle-Catalyzed Silane Alcoholysis. *J. Am. Chem. Soc.* **124**, 10508–10518 (2002).
3. Sinaÿ, P., James Pearce, A., Mallet, J.-M. & Sinay, P. One-Step Synthesis of Disaccharide Mimetics via Tandem Rearrangement of Unsaturated Disaccharides. *Heterocycles* **52**, 819–826 (2000).
4. Yoza, K., Amanokura, N., Ono, Y., Akao, T., Shinmori, H., Takeuchi, M., Shinkai, S. & Reinhoudt, D. N. Sugar-Integrated Gelators of Organic Solvents—Their Remarkable Diversity in Gelation Ability and Aggregate Structure. *Chem. Eur. J.* **5**, 2722–2729 (1999).
5. Dimitrijević, E., Cusimano, M. & Taylor, M. S. Synthesis of benzannulated heterocycles by twofold Suzuki–Miyaura couplings of cyclic diarylborinic acids. *Org. Biomol. Chem.* **12**, 1391–1394 (2014).
6. Mukherjee, M. M., Basu, N., Chaudhury, A. & Ghosh, R. Efficient one-pot per-O-acetylation–thioglycosidation of native sugars, 4,6-O-arylidenation and one-pot 4,6-O-benzylidenation–acetylation of S-/O-glycosides catalyzed by $Mg(OTf)_2$. *RSC Adv.* **6**, 109301–109314 (2016).
7. Singh, S., Scigelova, M., Critchley, P. & Crout, D. H. G. Trisaccharide synthesis by glycosyl transfer from p-nitrophenyl β-d-N-acetylgalactosaminide on to disaccharide acceptors catalysed by the β-N-acetylhexosaminidase from Aspergillus oryzae. *Carbohydr. Res.* **305**, 363–370 (1997).
8. Zhu, J., Wei, Y., Lin, D., Ou, C., Xie, L., Zhao, Y., Huang, W. & Zhu, J. One-pot synthesis of benzoxaborole derivatives from the palladium-catalyzed cross-coupling reaction of alkoxydiboron with unprotected o-bromobenzylalcohols. *Org. Biomol. Chem.* **13,** 11362–11368 (2015).
9. Schaus, S. E., Brandes, B. D., Larrow, J. F., Tokunaga, M., Hansen, K. B., Gould, A. E., Furrow, M. E. & Jacobsen, E. N. Highly Selective Hydrolytic Kinetic

Resolution of Terminal Epoxides Catalyzed by Chiral (salen)CoIIIComplexes. Practical Synthesis of Enantioenriched Terminal Epoxides and 1,2-Diols. *J. Am. Chem. Soc.* **124**, 1307–1315 (2002).

10. Ma, X. & Herzon, S. B. Intermolecular Hydropyridylation of Unactivated Alkenes. *J. Am. Chem. Soc.* **138**, 8718–8721 (2016).
11. Yang, Y., Cui, X.-K., Zhong, M. & Li, Z.-J. Study of carbohydrate–protein interactions using glyco-QDs with different fluorescence emission wavelengths. *Carbohydr. Res.* **361**, 189–194 (2012).
